# Dearomative di- and trifunctionalization of aryl sulfoxides via [5,5]-rearrangement

**DOI:** 10.1038/s41467-022-32426-6

**Published:** 2022-08-11

**Authors:** Mengjie Hu, Yanping Liu, Yuchen Liang, Taotao Dong, Lichun Kong, Ming Bao, Zhi-Xiang Wang, Bo Peng

**Affiliations:** 1 Key Laboratory of the Ministry of Education for Advanced Catalysis Materials, Zhejiang Normal University, Jinhua, China; 2 State Key Laboratory of Fine Chemicals, Dalian University of Technology, Dalian, China; 3 School of Chemical Sciences, University of the Chinese Academy of Sciences, Beijing, China

**Keywords:** Synthetic chemistry methodology, Reaction mechanisms

## Abstract

Aromatic [5,5]-rearrangement can in principle be an ideal protocol to access dearomative compounds. However, the lack of competent [5,5]-rearrangement impedes the advance of the protocol. In this Article, we showcase the power of [5,5]-rearrangement recently developed in our laboratory for constructing an intriguing dearomative sulfonium specie which features versatile and unique reactivities to perform nucleophilic 1,2- and 1,4-addition and cyclization, thus achieving dearomative di- and trifunctionalization of easily accessible aryl sulfoxides. Impressively, the dearomatization products can be readily converted to sulfur-removed cyclohexenones, naphthalenones, bicyclic cyclohexadienones, and multi-substituted benzenes. Mechanistic studies shed light on the key intermediates and the remarkable chemo-, regio- and stereoselectivities of the reactions.

## Abstract (web summary)

In contrast with well-established dearomatization protocols such as the Birch reduction, sigmatropic rearrangement-based dearomatizations are a less-explored but potentially effective route to saturated compounds. Here, the authors show a [5,5]-rearrangement-enabled dearomatization of aryl sulfoxides.

## Introduction

**Fig. 1:**
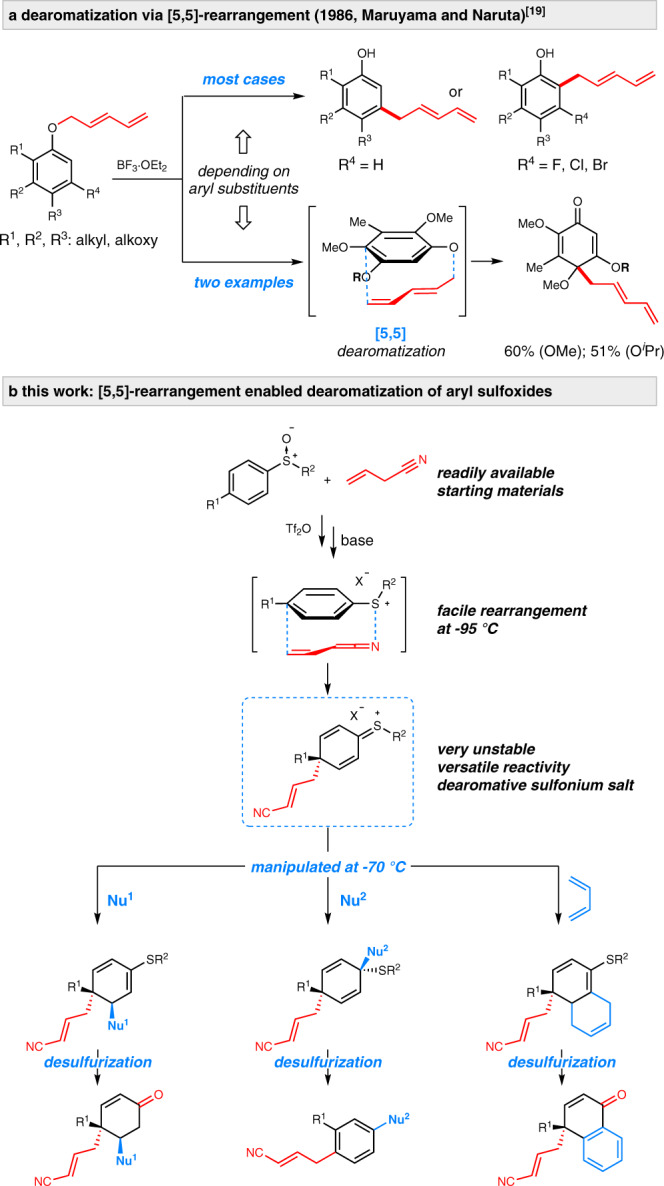
Background and this work. **a** Dearomatization via [5,5]-rearrangement by Maruyama and Naruta. **b** This work: [5,5]-rearrangement-enabled dearomatization of aryl sulfoxides.

Dearomatization represents a powerful strategy for converting readily available arenes to value-added alicyclic compounds^1–8^. In contrast with well-established dearomatization protocols such as the Birch reduction, oxidations of phenols, and transition-metal-mediated dearomatizations, etc., the sigmatropic rearrangement-based dearomatizations have not attracted much attention. There have been only sporadic examples of dearomatization via [2,3]- or [3,3]-rearrangement^9–16^, which are often limited to *ortho, ortho*′-disubstituted benzene derivatives^9–12^. Li and Lan showcased the potential of benzyne/sulfoxide mediated rearrangement in developing an unique dearomatization reaction^17,18^. In theory, [5,5]-rearrangement could enable *para*-functionalization of benzenes, thus affording *para*-functionalized alicyclic compounds and broadening the substrate scope. In 1986, Maruyama and Naruta attempted this idea by implementing Claisen-type rearrangement with *para*-substituted aryl pentadienyl ethers (Fig. 1a)^19^. In lieu of the expected dearomatization products formed via [5,5]-rearrangement, the reaction generally afforded *meta*- or *ortho*-alkylated benzenes. Nevertheless, switching to structurally well-defined substrates like 2,3,4,5-tetrasubstituted aryl pentadienyl ethers, they achieved the first two examples of [5,5]-rearrangement triggered dearomatization. Obviously, the high dependence of specific aryl substituents may severely impede the adoption of the dearomatization protocol. To the best of our knowledge, probably due to the lack of competent [5,5]-rearrangement protocols^19–24^, dearomatization reactions via such rearrangement still remain underexplored.

During the past few years, a great deal of research effort has been devoted to the development of [3,3]-rearrangements of sulfonium salts derived from aryl sulfoxides^25–30^. The reaction enabled the incorporation of an array of nucleophiles into the *ortho*-position of aryl sulfoxides while reducing sulfoxides to sulfides, thus providing a powerful synthetic tool for accessing 1,2-disubstituted arenes^31–58^. Not surprisingly, this type of rearrangements could be coupled with dearomatization processes^44–46,59–62^. For examples, Yorimitsu demonstrated an elegant dearomatization of phenols via their [3,3]-rearrangement with aryl/heteroaryl sulfoxides^44,45,59^. The [3,3]-rearrangement triggered dearomatization of benzothiophene *S*-oxides with phenols was also accomplished by Procter^46,60^. In addition, we applied the [3,3]-rearrangement to break the aromaticity of *ortho,ortho*′-disubstituted aryl sulfoxides and aryl iodanes using difluoro silyl enol ethers and α-stannyl nitriles as rearrangement partner, respectively^61,62^.

Unlike the well-studied [3,3]-rearrangements of aryl sulfoxides^25–30^, a [5,5]-rearrangement of aryl sulfoxides with allyl nitriles via an “assembly/deprotonation” sequence recently developed in our laboratory allows direct *para*- in lieu of *ortho*-C–H alkylation of aryl sulfoxides^63^. The rearrangement is dramatically accelerated by the congestion release of ketenimine moieties embodied in rearrangement precursors, thus the rearrangement could occur at very low temperature (−100 °C) and complete within a few seconds^63–66^. The effective [5,5]-rearrangement inspired us to quest its capability of unlocking aromaticity of arenes.

In this Article, we showcase the potential of the [5,5]-rearrangement for dearomatization of aryl sulfoxides and reveal the versatile reactivities of the rearrangement-enabled dearomatization intermediates (Fig. 1b). In contrast with the reported stable dearomatization product formed via [5,5]-rearrangement of pentadienyl ethers (Fig. 1a), the dearomative sulfonium species generated from our [5,5]-rearrangement are unstable at elevated temperature, but allowing for further manipulations at low temperature (−70 °C). The present study demonstrates that the in situ formed intermediate exhibits high electrophilicity and versatile reactivities towards a diverse of nucleophiles and cyclization partners. As a consequence, the protocol allows the installations of two different functionalities into aryl sulfoxides affording value-added alicyclic compounds which can be readily desulfurized to a wide variety of synthetic useful multi-substituted cyclohexenones, naphthalenones and benzenes etc.

## Results

### Development of the reaction

**Fig. 2:**
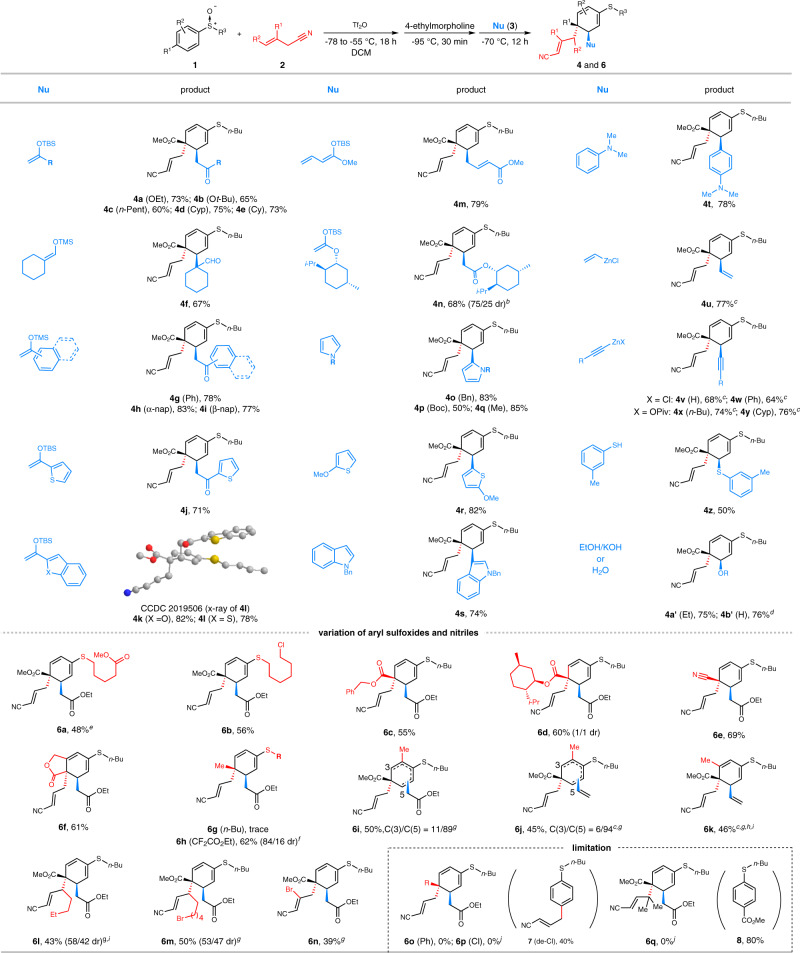
Dearomative 3,4-dual functionalization of aryl sulfoxides. Reactions were performed under optimal conditions given in Table 1, entry 15. Base (4-ethylmorpholine) and Nu were added in sequence (**4u**-**4y**, **4a′**, **4b′**, **6j** and **6k**) or in one portion (others). For cases given below dashed line, sily enol ether **3a** or vinyl zinc chloride **3t** were used as nucleophile. Unless otherwise noted, only one diastereoisomer was obtained. dr = diastereoisomeric ratio. ^[b]^ The absolute configuration of major isomer of **4n** is not confirmed. ^[c]^ Nu (3.0 equiv) was used. ^[d]^ 4-Ethylmorpholine (4.0 equiv) was used. ^[e]^ Tf_2_O, −55 °C, 36 h. ^[f]^ DIPEA instead of 4-ethylmorpholin was used. ^[g]^ Nu, −95 °C, 12 h. ^[h]^ **2a** (10 equiv) was used. ^[i]^ **6k** and **6l** were contaminated with small amounts of inseparable 1,2-addition products **6k′** and **6l′** with 86/14 rr and 83/17 rr, respectively. ^[j]^ In lieu of **6p** and **6q**, aryl sulfides **7** and **8** were obtained in 40% and 80% yields, respectively.

At the beginning, we examined the [5,5]-rearrangement of *para*-substituted aryl sulfoxide **1a** with allyl nitrile **2a** under the conditions we previously developed^63^ (Table 1). Silyl enol ether **3a** was employed as a nucleophile to trap the in situ formed dearomatization species. DABCO previously identified as superior base for deprotonative construction of rearrangement precursor proved unsuitable for the rearrangement of **1a** (entry 1). However, when switching to DIPEA, the one-pot three-step reaction afforded desired dual functionalized dearomatizaiton product **4a** in modest yield (40% NMR yield). Remarkably, the reaction exclusively produced 1,4-addition product **4a** with single diastereomer, exhibiting an excellent regio- and stereoselectivity. The relative configuration of **4a** was deduced by single-crystal X-ray analysis of its analog **4l** (Fig. 2). Among various organic bases (entries 1–6), 4-ethylmorpholine appeared to be optimal, affording **4a** in 70% NMR yield (entry 5). Afterwards, the investigation of reaction temperature (T^1^) and reaction time (t^1^) revealed that the rearrangement partners assembled at −55 °C for 18 h could increase the yield of **4a** to 79% (entries 7–10). Optimization of temperature (T^2^) and time (t^2^) for trapping dearomatization species with **3a** could not further enhance the efficiency of the reaction (entries 11–14). Comparing with the sequential addition (entry 9), the addition of base and nucleophile in one portion produced nearly the same yield of **4a** (entry 15). This result indicated that silyl enol ether **3a** could be tolerated in the stage of base promoted deprotonation/rearrangement. For simple operation, the base and nucleophile were added in one portion in the following study unless otherwise noted. It should be noted that the reaction under optimal conditions still furnished a slight amount of undesired *ortho*-cyanoalkylated product **5** (6% yield).

**Table 1:** Development of the Reaction^a^


Entry	Base	T^1^, t^1^	T^2^, t^2^	Yield^b^
1	DABCO	−50 °C, 18 h	−70 °C, 12 h	Trace
2	DIPEA	−50 °C, 18 h	−70 °C, 12 h	40
3	NEt_3_	−50 °C, 18 h	−70 °C, 12 h	63
4	DBU	−50 °C, 18 h	−70 °C, 12 h	38
5	4-ethylmorpholine	−50 °C, 18 h	−70 °C, 12 h	70
6	2-methylpyridine	−50 °C, 18 h	−70 °C, 12 h	34
7	4-ethylmorpholine	−50 °C, 12 h	−70 °C, 12 h	50
8	4-ethylmorpholine	−40 °C, 18 h	−70 °C, 12 h	30
9	4-ethylmorpholine	−55 °C, 18 h	−70 °C, 12 h	79
10	4-ethylmorpholine	−60 °C, 18 h	−70 °C, 12 h	66
11	4-ethylmorpholine	−55 °C, 18 h	−95 °C, 12 h	53
12	4-ethylmorpholine	−55 °C, 18 h	−80 °C, 12 h	71
13	4-ethylmorpholine	−55 °C, 18 h	−60 °C, 12 h	54
14	4-ethylmorpholine	−55 °C, 18 h	−70 °C, 6 h	72
15	4-ethylmorpholine	−55 °C, 18 h	−70 °C, 12 h	81(73)^c^

^a^Reactions were performed in a one-pot three-step manner: (1) **1a** (0.5 mmol), **2a** (3.0 equiv), Tf_2_O (1.5 equiv), T^1^ (°C), t^1^ (h); (2) base (2.5 equiv), −95 °C, 30 min; (3) **3a** (2.0 equiv), −95 °C to T^2^ (°C), t^2^ (h). Tf_2_O was added at −78 °C. Tf_2_O: triflic anhydride. DABCO: triethylenediamine. DIPEA: diisopropyl triethylamine. DBU: 1,8-diazabicyclo[5.4.0]undec-7-ene.

^b^NMR yields using mesitylene as internal standard and isolated yields given in parentheses.

^c^Base and Nu were added in one portion. *Ortho*-alkylated product **5** was obtained in 6% yield.

### Scope of the methodology

Next, the generality of the dearomative 3,4-dual functionalization reaction was investigated under the optimal conditions (Fig. 2). Gratifyingly, a wide variety of nucleophiles including silyl enol ethers (**4a**-**4n**), electron-rich arenes and heteroarenes (**4o**-**4t**), organozinc reagents (**4u**-**4y**) and heteroatom nucleophiles (**4z-4b′**) proved to be suitable for the reaction. As a result, carbonyl groups (**4a**-**4n**), arenes/heteroarenes (**4o**-**4t**), vinyl group (**4u**), alkynyl groups (**4v**-**4y**), sulfur group (**4z**), ethoxy group (**4a′**) and hydroxyl group (**4b′**) were smoothly anchored on the *meta*-position of aryl sulfoxides. It is impressive that the transformations consisted of three independent steps but still exhibited a high efficiency to afford 3,4-dual functionalized dearomatization products in generally good yields. Furthermore, in all cases, the reaction showcased excellent regio- and stereoselectivity producing single diastereomers. The X-ray diffraction analysis unambiguously demonstrated the relative configuration of **4l**. Other products were assigned by analogy to **4l**. Interestingly, the feasibility of stereoselective addition was proved by using chiral auxiliary modified silyl enol ether **3n** as nucleophile which produced **4n** with a modest diastereoselectivity (75/25 dr). It is worthy of noting that the multi-component reaction also demonstrated an excellent functional group (FG) compatibility. A wide range of FGs including aldehyde (**4f**), arenes/heteroarenes (**4o**-**4t**), vinyl group (**4u**), alkynyl groups (**4v**-**4y**), and heteroatoms (**4z**-**4b′**) were all tolerated in the reaction. The excellent FG compatibility can be attributed to the independent and precise control of each step of the reaction and the mild reaction conditions.

In addition, the scope of aryl sulfoxides and nitriles was studied under the optimal conditions (Fig. 2, below the dashed line). In addition to “*n*-Bu” group, the substituents on sulfur alkyl group such as ester (**6a**) and halide (**6b**) groups could also be well accommodated. The introduction of a chiral auxiliary group to *para*-ester group failed to induce any stereoselectivity wherein **6d** was obtained with 1/1 dr. To our delight, besides *para*-ester groups, the reaction of *para*-cyano substituted aryl suloxide afforded a good yield of **6e** (69% yield).

Remarkably, a structurally appealing bicyclic product **6f** was obtained in a good yield (61%). *para*-Methyl substituted aryl sulfoxide failed to furnish desired **6g**. However, switching S-substituent from “*n*-Bu” to “CF_2_CO_2_Et” could change the fate of the reaction to afford dearomatization product **6h** in a good yield (62%). We speculated that the electron-withdrawing group would increase the electrophilicity of activated sulfoxide and thus enhance the assembly of sulfoxide and allyl nitrile which has been studied previously in our laboratory^55^ Meanwhile, we do not believe the “fluoro alkyl” group could exert any special influence on the rearrangement process. The reaction of *ortho*-methyl substituted aryl sulfoxide gave rise to the formation of regioisomers **6i** in 50% yield with 11/89 rr. The use of vinyl zinc reagent as nucleophile slightly increased the regioselectivity affording **6j** with 6/94 rr. Impressively, despite steric hinderance, the 1,4-addition of vinyl zinc chloride still proceeded smoothly to give intriguing 3,4,5-densely-substituted **6k** albeit accompanied by a small amount of 1,2-addition product (7% yield). Remarkably, in addition to simple allyl nitrile (**2a**), γ-alkylated allyl nitriles (**2b** and **2c**) and β-bromo-allyl nitrile (**2d**) were all adopted by the reaction albeit with relatively low yields (**6l**, 43%; **6m**, 50% and **6n**, 39%) and poor diastereoselectivity (**6l**, 58/42 dr and **6m**, 53/47 dr). *para*-Ph or Cl-phenyl sulfoxides failed to produce any desired products **6o** and **6p**. Interestingly, dechlorination product **7** was obtained from the reaction of *para*-Cl phenyl sulfoxide. Probably due to steric hinderance, γ-dialkylated allyl nitrile **2e** could not lead to any desired **6q**. Instead, aryl sulfide **8** resulted from reduction of sulfoxide was obtained in 80% yield.

**Fig. 3:**
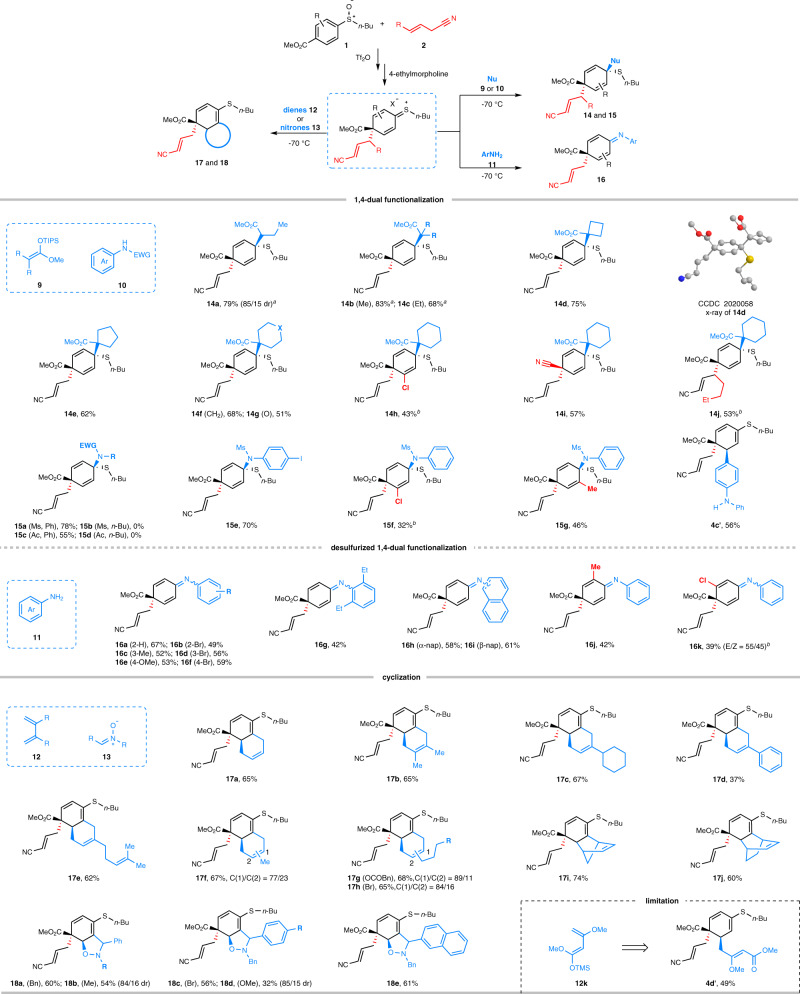
Dearomative 1,4-dual functionalization and dearomative cyclization of aryl sulfoxides. Reactions were performed on 0.5 mmol scale under optimal conditions. 5.0 equiv of dienes and 3.0 equiv of dipoles were used in the reaction. 4-ethylmorpholine and Nu were added in one portion (**14a**-**14c**, **14j**, **15a**-**15g** and **4c′**) or in sequence (others). Unless otherwise noted, only one diastereoisomer was obtained. dr = diastereoisomeric ratio. ^*a*^ TMS-protected enol silyl ethers were used. ^*b*^ Nu, −95 °C, 12 h.

In contrast with 1,4-addition of silyl enol ether **3a** (Fig. 2), the use of β-alkylated silyl enol ethers (**9**) and aryl amines (**10** and **11**) as nucleophile led to a complete regioselectivity switch achieving dearomative 1,4-dual functionalization of aryl sulfoxides (Fig. 3). In addition to β-alkyl silyl enol ether **9a**, β,β′-dialkyl silyl enol ethers **9b**-**9g** smoothly afforded **14b**-**14g** constructing two adjacent quaternary stereocenters. The relative configuration of **14d** was unambitiously confirmed by its X-ray structure that was also used for assigning the stereochemistry of other products. Similar to the 1,4-addition pattern shown in Fig. 2, the reaction proceeding via 1,2-addition also showcased excellent regio- and stereoselectivity. In addition to **1a**, *meta*-chloridephenyl sulfoxide and *para*-cyanophenyl sulfoxide were also suitable for the reaction albeit furnishing **14h** and **14i** in modest yields. Impressively, γ-alkylated allyl nitrile **2b** could overcome the steric hinderance to afford **14j** in a good yield. Furthermore, *N*-Ms or Ac protected aryl amines **10a**, **10c** and **10e** were found to be competent nucleophiles to access 1,4-dual functionalized products **15a**, **15c**, and **15e**, respectively. However, Ms or Ac protected aliphatic amines **10b** and **10d** proved unstuiable for the reaction. In contrast with protected aryl aimines, diphenylamine exclusively produced 1,4-addition product **4c′** with C–C bond formation. This result probably reflects a significant impact of steric and electronic effects of nucleophiles on the regioselectivity of the reaction.

Unlike secondary aryl amines, the reaction of primary aryl amines **11** led to quinone imines (**16**) with removal of sulfur moieties (Fig. 3). Among them, the reaction of unsymmetrical *ortho*-methyl phenyl sulfoxide proceeded with excellent stereoselectivity to give (*E*)-quinone imines (**16j**). In contrast, *meta*-chlorophenyl sulfoxide suffered poor stereoselectivity furnishing **16k** as a mixture of stereoisomers (*E*/*Z* = 55/45).

In addition to 1,4- and 1,2-addition, we further examined the feasibility of cyclization using in situ generated dearomative sulfonium intermediate as shown in Fig. 3. To our delight, a diverse of cyclization partners were successfully incorporated into aryl sulfoxides via consecutive dearomatization/cyclization process. Dienes **12** were used to trap dearomative sulfonium species via [4 + 2]-cyclization producing various bicyclic compounds **17**. It is remarkable that dienes bearing 2-cyclohexyl (**12c**), phenyl (**12d**) groups and alkene tethered alkyl chain (**12e**) afforded desired products **17c**-**17e** with excellent regioselectivity. In contrast, small portion of regioisomers were determined when employing 2-methyl, 2-bromoalkyl and 2-carbonate substituted dienes **12f**-**12h**. Not surprisingly, both cyclopentadiene **12i** and cyclohexadiene **12j** were well adopted by the reaction. In addition to dienes, nitrones **13** were also found to be competent for trapping the dearomative sulfonium intermediate via [3 + 2]-cyclization leading to structurally appealing hetero-bicyclic compounds **18**. Despite the remarkably high electrophilicity of dearomative sulfonium species, electron-rich diene **12k** failed to afford desired bicyclic product **17k** wherein 1,4-addition product **4d’** was obtained in a modest yield.

Overall, the [5,5]-rearrangement triggered dearomatization followed by either 1,4- and 1,2-addition and cyclization exhibited a remarkable synthetic efficiency of converting readily available aryl sulfoxides to valuable densely-substituted 1,3- and 1,4- cyclohexadienes and bicyclic compounds which can be difficult to make by other known methods.

### Transformation of products to value-added alicyclic compounds

**Fig. 4:**
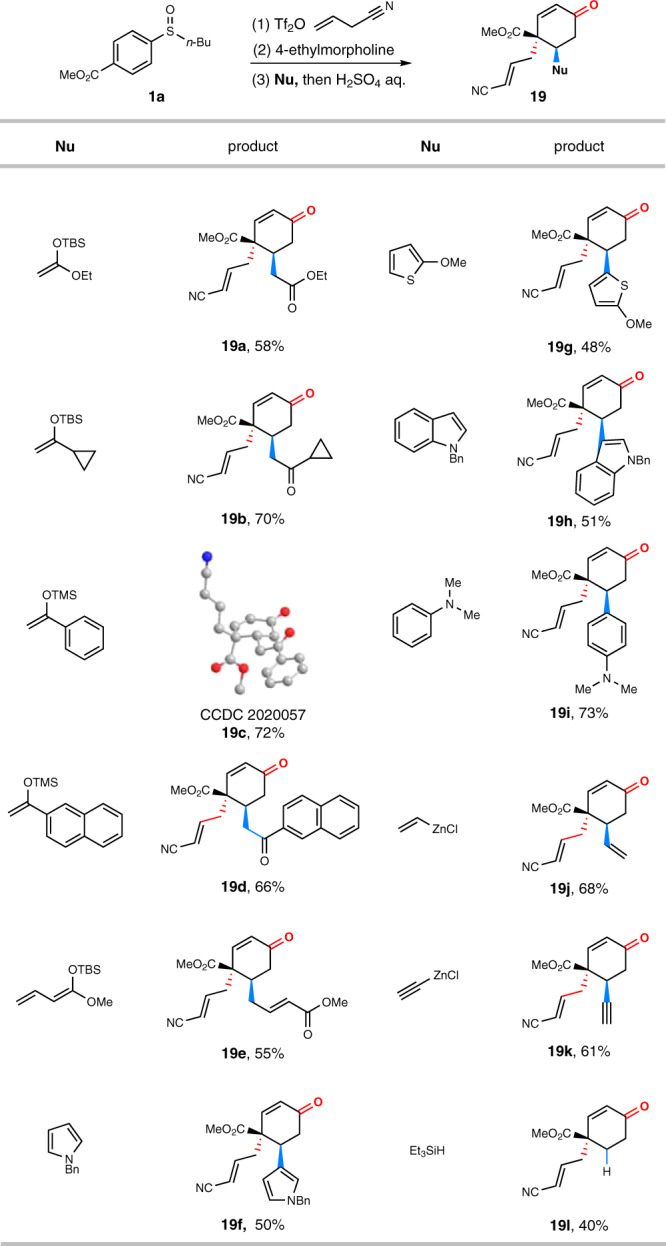
One-pot synthesis of cyclohexenones. The reaction was performed on 0.5 mmol scale under optimum conditions. After addition of Nu, the reaction mixture was treated with H_2_SO_4_ aq./EtOH at 65 °C for 10 h. In cases of **19a-19k**, only one diastereoisomer was obtained.

To demonstrate the utility of the reaction, we combined the dearomative 3,4-dual functionalization process with thioenol ether hydrolysis (Fig. 4). As a result, the protocol allowed the facile synthesis of a wide variety of cyclohexenones. Impressively, the whole transformation despite consisting of four independent stages still achieved intriguing densely-substituted cyclohexenones in respectful yields (40–73%). It is noteworthy that the final hydrolysis of thioenol ethers had no detrimental effect on dearomatization products. The stereochemistry of rearrangement products and their functional groups such as esters/ketones (**19a**-**19e**), alkene (**19j**), alkynyl group (**19k**), and arenes/heteroarenes (**19f**-**19i**) remained intact in the hydrolysis. In addition to excellent regio- and stereoselectivity and remarkable functional group compatibility, the use of readily available starting materials demonstrated the practicality of the protocol for producing polysubstituted cyclohexenones.

**Fig. 5:**
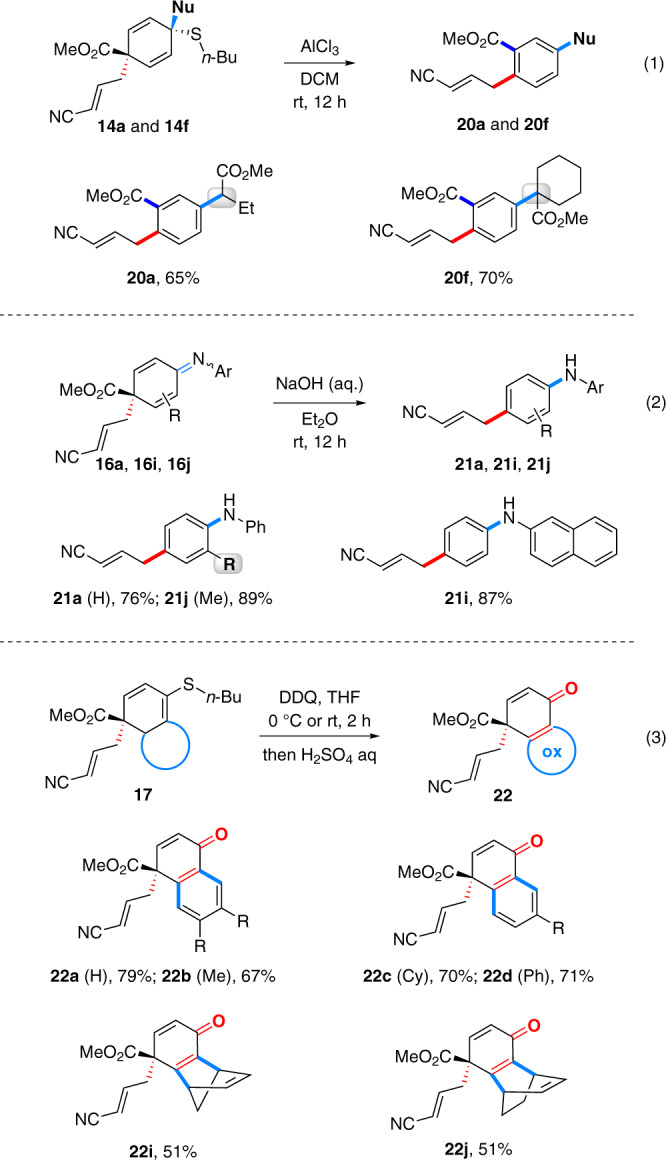
Elaboration of 1,4-cyclohexedienes 14 and 16 and bicyclic products 17. Conditions for eq 1: **14** (0.2 mmol), AlCl_3_ (0.5 equiv), DCM, rt, 12 h; Conditions for eq 2: **16** (0.2 mmol), NaOH (10% aq.)/Et_2_O, rt, 12 h; Conditions for eq 3: **17** (0.2 mmol), DDQ (2.0 equiv), THF, 0 °C or rt, 2 h; then H_2_SO_4_ aq., rt, 12 h.

The unique structures of 1,4-cyclohexediene products **14** and **16** and bicyclic product **17** prompted us to examine their reactivities. As illustrated in eqs 1 and 2 (Fig. 5), in presence of AlCl_3_, desulfurization of **14a** and **14f** was achieved to afford intriguing multi-substituted benzenes **20a** and **20f**, respectively wherein the ester group shifted from the *para* to *meta*-position (eq 1). Basic hydrolysis enabled de-esterification rearomatization of quinone imines **16a**, **16i** and **16j** producing valuable anilines **21a**, **21i**, and **21j**, respectively (eq 2). A consecutive DDQ oxidation and hydrolysis allows for the removal of sulfur moieties of dearomative cyclization products **17** leading to naphthalenones **22a**-**22d** and cyclohexadienones **22i** and **22j** (eq 3). As such, the versatile derivatizations of dearomatization products showcases their potential in synthetic applications.

### The overall mechanism of the reaction

**Fig. 6:**
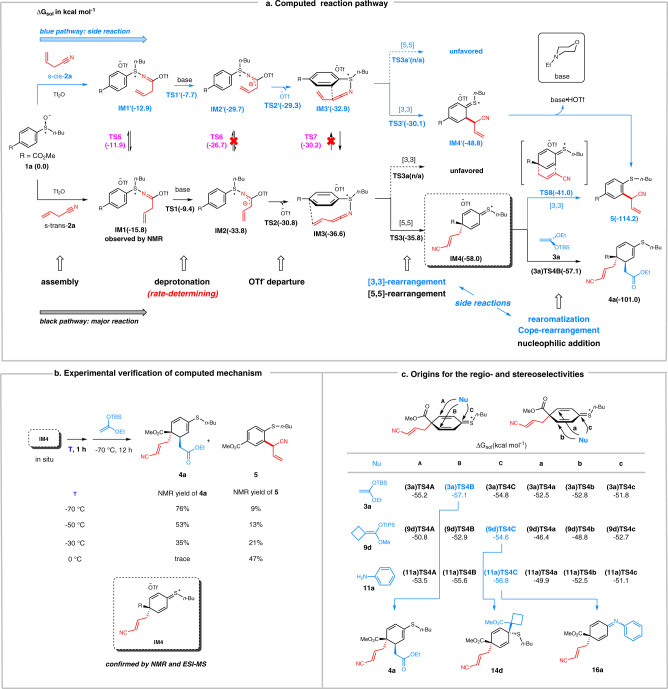
Mechanistic study results. **a** The overall mechanism of the reaction. **b** Understanding the formation of 5. **c** Understanding the regio- and stereoselectivities of the reaction.

To gain insights into the reaction mechanism, density functional theory (DFT) calculations (see SI 8.1 for computational details) were carried out to characterize the detailed pathways for the reaction of **1a** with **2a** and **3a** (Fig. 6a). **2a** has two conformers, namely, s-trans-conformer (s-trans-**2a**) and s-cis conformer (s-cis-**2a**). We first discuss the reaction pathway of s-trans-**2a**, colored in black. To begin with, the activator Tf_2_O activates **1a** to enable the formation of the salt **IM1** with a mechanism we reported previously^63^. The base 4-ethylmorpholine then deprotonates **IM1** via **TS1**, followed by OTf^-^ departure, generating the salt **IM3** as the rearrangement precursor. By crossing **TS3**, dearomative [5,5]-rearrangement takes place to convert **IM3** to **IM4**. Geometric optimizations to locate a transition state (namely, **TS3a**) for [3,3]-rearrangement repeatedly converged to [5,5]-rearrangement transition state **TS3**, which, along with the very low [5,5]-rearrangement barrier (0.8 kcal mol^−1^, **TS3** relative to **IM3**), indicates the strong preference of **IM3** to undergo of [5,5]-rearrangement. Finally, the nucleophile **3a** intercepts the rearrangement intermediate **IM4** via nucleophilic 1,4-addition (see below for more details), affording the product **4a**. Overall, the reaction is exergonic by 101.0 kcal mol^−1^, with a rate-determining barrier of 6.4 kcal mol^−1^ (**TS1** relative to **IM1**) for deprotonation. The energetics well explains why the reaction could proceed facilely.

The reaction pathway of s-cis-**2a**, colored in blue, is similar to that of s-trans-**2a**, except for a [3,3]-rearrangement for **IM3′** leading to **IM4′** and then **5**. Similar to the case **IM3**, geometric optimizations to locate a transition state (namely, **TS3a′**) for [5,5]-rearrangement repeatedly converged to [3,3]-rearrangement transition state **TS3′**. Thus, **IM3** and **IM3′** intrinsically prefer [5,5]- and [3,3]-rearrangement, respectively, which we attribute to the longer *trans*-ketenimine moiety in **IM3** than the *cis*-ketenimine moiety in **IM3′**. Comparing the two pathways, the intermediates and the transition states in the blue pathway are all above their counterparts in the black pathway. Notably, **TS3′** for [3,3]-rearrangement is 5.7 kcal mol^−1^ higher than **TS3** for [5,5]-rearrangement. Similar to our previous study^63^, we attribute the higher **TS3′** than **TS3** to the linear ketenimine group which brings up strain in the six-membered [3,3]-rearrangement transition state **TS3′**.

### Understanding the formation of 5

It is interesting that, as the reaction mainly afforded dearomative product **4a**, it meantime gave minor rearomatization product **5** (Table 1). On the basis of the reaction pathways of s-trans-**2a** and s-cis-**2a**, we attribute the formation of **5** to the kinetic competition between the two pathways. Thus, we located the transition states **TS5**-**TS8** for possible inter-conversions between the two pathways. **TS6** and **TS7** are 4.1 and 5.7 kcal mol^−1^ higher than **TS2** and **TS3**, respectively, thus **IM2** or **IM3** can be excluded as competition entrances. Because **TS5** for the conversion of **IM1** to **IM1′** is lower than **TS1** by 2.5 kcal mol^−1^, the two pathways compete at the deprotonation with an energetic difference of 1.7 kcal mol^−1^ (**TS1′** relative to **TS1**) in favor of the formation of **4a**. The energetic difference predicts an 80/1 ratio of **IM4**/**IM4′**. The formation of **IM4′** provides an entry to **5**. Alternatively, **IM4** can convert to the more stable **5** via Cope rearrangement via **TS8** when **3a** was not added. Therefore, two pathways contribute to the formation of **5**, which could be why the experimental yield of **5** (6%) is higher than the predicted yield according to the ratio of **IM4**/**IM4′**. Note that, because **TS8** is much higher than **(3a)TS4B** by 16.1 kcal mol^-1^, the Cope-rearrangement pathway could be excluded when **3a** was presented.

On the basis of the computed mechanism and the energetics, we reasoned the following: (i) **IM1** could be observed in the absence of base, because the reaction can stop at **IM1**. **IM4** could also be observed in the presence of base but absence of **3a**, because **IM4** is kinetically stable with a relatively high barrier of 17.0 kcal mol^−1^ (**TS8** relative to **IM4**). Indeed, at low temperature (−78 °C), we observed both **IM1** and **IM4** with NMR spectroscopy. It should be noted that the sample of **IM4** was pretreated with an acid (CF_3_COOH) prior to NMR measurement since we failed to correct baseline distortions in NMR spectra of the original sample. In addition, **IM4** was also detected by ESI-MS. (ii) In the absence of **3a**, the reaction could afford **5** through the competitive [3,3]-rearrangement and the feasible Cope rearrangement of **IM4** to **5**. To corroborate this, we carried out the control experiment (Fig. 6b). After in situ generation of **IM4**, we stopped the reaction for 1 h at a given temperature (T), and then continued the reaction for 12 hrs by adding **3a** at −70 °C. It was observed that as T increases, the yield of **4a** decreases, while that of **5** increases. The experimental results agree with (ii), because raising the temperature (T) favors **IM4** to undergo irreversible Cope-rearrangement to give **5** during the period of 1 h.

### Understanding the regio- and stereoselectivities of the reaction

In addition to **3a**, we further considered the nucleophilic additions of **9d** and **11a** to **IM4**. The pathways for the reactions of **9d** and **11a** with **IM4** to give **14d** and **16a**, respectively, are detailed in Supplementary Fig. 26 in SI 8. Figure 6c shows the results for the nucleophilic addition step. For each of the three nucleophiles, we considered six possible addition modes and located the corresponding six transition states. Note that addition modes A and B (or a and b) can be differentiated with respect to the S-nBu group and the stereochemistry of *para*-carbon. Due to the less steric effect of ester group than the cyanoalkyl group lying on the other side, the three nucleophiles all prefer approaching **IM4** from its upper face syn to the ester group, in agreement with our observed stereoselectivity of the reaction. The combinations of steric and electronic effects favor **3a** to undergo 1,4-addition giving **4a**, while **9d** and **11a** to undergo 1,2- additions to give **14d** and **16a**, respectively. The energetic results explain our experimentally obtained products.

## Discussion

In contrast with well-established dearomatization protocols such as the Birch reduction, sigmatropic rearrangement-based dearomatizations are a less-explored but potentially effective route to saturated compounds. Here, we show a [5,5]-rearrangement-enabled dearomatization of aryl sulfoxides. The key step of the reaction is the in situ formation of an intriguing dearomative sulfonium species via [5,5]-rearrangement of aryl sulfoxides with ally nitrile. This dearomative species is found to be unstable at evaluated temperature but could be manipulated at certain low temperature (−70 °C). Impressively, the intermediate exhibits versatile reactivities towards a wide variety of nucleophiles. As a result, the protocol allows for converting three readily available substrates to a diverse of polysubstiuted 1,3- and 1,4-cyclohexendienes, quinone imines and bicyclic compounds in a regio- and stereoselective manner. Simple elaboration of the dearomatization products produces valuable sulfur-removed cyclohexenones, naphthalenones, bicyclic cyclohexadienones, and multi-substituted benzenes. Mechanistic studies well explain the occurrence of the reaction with excellent chemo-, regio- and stereoselectivities. Further studies of the structurally unique dearomatization products and [5,5]-rearrangement triggered dearomatization reactions are underway.

## Methods

### Representative procedure for dearomative di- and trifunctionalization of aryl sulfoxides

To a mixture of aryl sulfoxide **1a** (120 mg, 0.5 mmol) and allyl nitrile **2a** (121 μL, 3.0 equiv) in DCM (3.0 mL) was added Tf_2_O (126 μL, 1.5 equiv) at −78 °C under N_2_ amosphere. The mixture was gradually warmed to −55 °C. After stirring for 18 h, the mixture was cooled to −95 °C. To the resulted mixture was added a mixture of 4-ethylmorpholine (157 μL, 2.5 equiv) and silyl enol ether **3a** (202 mg, 2.0 equiv) in DCM (2.0 mL) dropwise in 20 min using syringe pump. After stirring for 30 min, the mixture was gradually warmed to −70 °C and further stirred for 12 h. Then the mixture was passed through a short silica gel column and concentrated under vacuum. The obtained residue was further purified by flash chromatography on silica gel affording dearomatization product **4a**. For other procedures, see the Supplementary Information.

## Supplementary information

Supplementary Information

Description of Additional Supplementary Files

Supplementary data 1

## Data Availability

The X-ray crystallographic coordinates for structures of **4l**, **14d**, and **19c** reported in this study have been deposited in the Cambridge Crystallographic Data Center (CCDC) under deposition numbers CCDC 2019506(**4l**), 2020058(**14d**) and 2020057(**19c**). These data can be obtained free of charge from http://www.ccdc.cam.ac.uk/data_request/cif. Full experimental details, characterization data, and NMR spectra for all new compounds are available within this paper and its Supplementary Information. For the Cartesian coordinates and energies of the optimized structures, see Supplementary Data 1 File.

## References

[1] Wertjes WC, Southgate EH, Sarlah D (2018). Recent advances in chemical dearomatization of nonactivated arenes. Chem. Soc. Rev..

[2] Zheng C, You S-L (2016). Catalytic asymmetric dearomatization by transition-metal catalysis: a method for transformations of aromatic compounds. Chem.

[3] Wu W-T, Zhang L, You S-L (2016). Catalytic asymmetric dearomatization (CADA) reactions of phenol and aniline derivatives. Chem. Soc. Rev..

[4] Zhuo C-X, Zhang W, You S-L (2012). Catalytic asymmetric dearomatization reactions. Angew. Chem. Int. Ed..

[5] Huck CJ, Sarlah D (2020). Shaping molecular Landscapes: Recent advances, opportunities, and challenges in dearomatization. Chem.

[6] Zheng C, You S-L (2019). Catalytic asymmetric dearomatization (CADA) reaction-enabled total synthesis of indole-based natural products. Nat. Prod. Rep..

[7] Roche SP, Porco JA (2011). Dearomatization strategies in the synthesis of complex natural products. Angew. Chem. Int. Ed..

[8] You, S.-L., Ed. *Asymmetric Dearomatization Reactions*; (Wiley-VCH, 2016).

[9] McComas CC, Van Vranken DL (2003). Application of chiral lithium amide bases to the Thia-Sommelet dearomatization reaction. Tetrahedron Lett..

[10] Berger R, Ziller JW, Van Vranken DL (1998). Stereoselectivity of the Thia-Sommelet [2,3]-dearomatization. J. Am. Chem. Soc..

[11] Burdon MG, Moffatt JG (1965). Acid-catalyzed reactions of phenols with dimethyl sulfoxide and dicyclohexylcarbodiimide. J. Am. Chem. Soc..

[12] Hauser CR, Van Eenam DN (1957). Rearrangement of 2,4,6-trimethylbenzyltrimethylammonium ion by sodium amide to form an *exo*-methylenecyclohexadieneamine and its reactions. J. Am. Chem. Soc..

[13] Peruzzi MT, Lee SJ, Gagne MR (2017). Gold(I) catalyzed dearomative Claisen rearrangement of allyl, allenyl methyl, and propargyl aryl ethers. Org. Lett..

[14] Huang S, Kötzner L, Kanta De C, List B (2015). Catalytic asymmetric dearomatizing redox cross coupling of ketones with aryl hydrazines giving 1,4-diketones. J. Am. Chem. Soc..

[15] Linton EC, Kozlowski MC (2008). Catalytic enantioselective Meerwein-Eschenmoser Claisen rearrangement: asymmetric synthesis of allyl oxindoles. J. Am. Chem. Soc..

[16] Alshreimi AS (2020). Synthesis of spirocyclic 1-pyrrolines from nitrones and arynes through a dearomative [3,3’]-sigmatropic rearrangement. Angew. Chem. Int. Ed..

[17] Shi J (2021). Aryne 1,2,3,5-tetrasubstitution enabled by 3-silylaryne and allyl sulfoxide via an aromatic 1,3-silyl migration. J. Am. Chem. Soc..

[18] Shi J (2021). Benzyne 1,2,4-Trisubstitution and Dearomative 1,2,4- Trifunctionalization. J. Am. Chem. Soc..

[19] Maruyama K, Nagai N, Naruta Y (1986). Lewis acid mediated Claisen-type rearrangement of aryl dienyl ethers. J. Org. Chem..

[20] Hassan AA (2019). Synthesis and crystallographic evaluation of diazenyl- and hydrazothiazoles. [5.5] sigmatropic rearrangement and formation of thiazolium bromide dihydrate derivatives. J. Mol. Struct..

[21] Liu L (2017). Organocatalytic para-selective amination of phenols with iminoquinone monoacetals. Org. Lett..

[22] Bouillon ME, Meyer HH (2016). The 4.4’-benzidine rearrangement of 4-alkyl substituted hydrazobenzenes. Tetrahedron.

[23] Gao H, Ess DH, Yousufuddin M, Kürti L (2013). Transition-metal-free direct arylation: synthesis of halogenated 2-amino-2’-hydroxy-1,1’-biaryls and mechanism by DFT calculations. J. Am. Chem. Soc..

[24] Maruyama K, Nagai N, Naruta Y (1985). Lewis Acid Mediated Claisen-Type Rearrangement of Aryl Dienyl Ethers. Tetrahedron Lett..

[25] Yanagi T, Nogi K, Yorimitsu H (2018). Recent development of *ortho*-C–H functionalization of aryl sulfoxides through [3,3]-sigmatropic rearrangement. Tetrahedron Lett..

[26] Yorimitsu H (2017). Cascades of interrupted Pummerer reaction-sigmatropic rearrangement. Chem. Rec..

[27] Pulis AP, Procter DJ (2016). C−H coupling reactions directed by sulfoxides: teaching an old functional group new tricks. Angew. Chem. Int. Ed..

[28] Smith LHS, Coote SC, Sneddon HF, Procter DJ (2010). Beyond the Pummerer reaction: recent developments in thionium ion chemistry. Angew. Chem. Int. Ed..

[29] Kaiser D, Klose I, Oost R, Neuhaus J, Maulide N (2019). Bond-forming and -breaking reactions at sulfur(IV): sulfoxides, sulfonium salts, sulfur ylides, and sulfinate salts. Chem. Rev..

[30] Zhang L, Hu M, Peng B (2019). [3,3]- and [5,5]-sigmatropic rearrangements of aryl sulfoxides using an ‘assembly/deprotonation’ technology. Synlett.

[31] Eberhart AJ, Cicoira C, Procter DJ (2013). Nucleophilic *ortho*-allylation of pyrroles and pyrazoles: an accelerated Pummerer/thio-Claisen rearrangement sequence. Org. Lett..

[32] Eberhart AJ, Imbriglio JE, Procter DJ (2011). Nucleophilic *ortho*-allylation of aryl and heteroaryl sulfoxides. Org. Lett..

[33] Padwa A, Nara S, Wang Q (2006). Additive Pummerer reaction of heteroaromatic sulfilimines with carbon nucleophiles. Tetrahedron Lett..

[34] Akai S (2006). Regioselective, nucleophilic carbon–carbon bond formation at the C4-position of indoles initiated by the aromatic Pummerer-type reaction. Tetrahedron Lett..

[35] Akai S (2004). Highly regioselective nucleophilic carbon–carbon bond formation on furans and thiophenes initiated by Pummerer-type reaction. Org. Lett..

[36] Eberhart AJ (2016). Sulfoxide-directed metal-free cross-couplings in the expedient synthesis of benzothiophene-based components of materials. Chem. Sci..

[37] Eberhart AJ (2015). Sulfoxide-directed metal-free *ortho*-propargylation of aromatics and heteroaromatics. Chem. Eur. J..

[38] Eberhart AJ, Procter DJ (2013). Nucleophilic *ortho*-propargylation of aryl sulfoxides: an interrupted Pummerer/allenyl thio-Claisen rearrangement sequence. Angew. Chem. Int. Ed..

[39] Fernández-Salas JA, Eberhart AJ, Procter DJ (2016). Metal-free CH–CH-type cross-coupling of arenes and alkynes directed by a multifunctional sulfoxide group. J. Am. Chem. Soc..

[40] Meng X (2019). Synthesis of polysubstituted cyclic 1,2-diketones enabled by iterative sulfoxide-mediated arylation. Chem. Commun..

[41] Huang X, Patil M, Farès C, Thiel W, Maulide N (2013). Sulfur(IV)-mediated transformations: from ylide transfer to metal-free arylation of carbonyl compounds. J. Am. Chem. Soc..

[42] Huang X, Maulide N (2011). Sulfoxide-mediated α-arylation of carbonyl compounds. J. Am. Chem. Soc..

[43] Shrives HJ, Fernández-Salas JA, Hedtke C, Pulis AP, Procter DJ (2017). Regioselective synthesis of C3 alkylated and arylated benzothiophenes. Nat. Commun..

[44] Yanagi T (2016). Metal-free approach to biaryls from phenols and aryl sulfoxides by temporarily sulfur-tethered regioselective C–H/C–H coupling. J. Am. Chem. Soc..

[45] Okamoto K (2018). Sigmatropic dearomatization/defluorination strategy for C-F transformation: synthesis of fluorinated benzofurans from polyfluorophenols. Angew. Chem. Int. Ed..

[46] He Z (2018). Synthesis of C2 substituted benzothiophenes via an interrupted Pummerer/[3,3]-sigmatropic/1,2-migration cascade of benzothiophene S-Oxides. Angew. Chem. Int. Ed..

[47] Yanagi T, Nogi K, Yorimitsu H (2020). Sulfoxide-directed iterative assembly into oligoarenes. Synlett.

[48] Okamoto K, Nogi K, Shimokawa J, Yorimitsu H (2020). C–F arylation of polyfluorophenols by means of sigmatropic dearomatization/defluorination sequence. Chem. Eur. J..

[49] Yanagi T, Yorimitsu H (2021). Mechanistic Investigation of a Synthetic Route to Biaryls by the Sigmatropic Rearrangement of Arylsulfonium Species. Chem. Eur. J..

[50] Yanagi T, Nogi K, Yorimitsu H (2020). Construction of biaryls from aryl sulfoxides and anilines by means of a sigmatropic rearrangement. Chem. Eur. J..

[51] Yoshida A, Okamoto K, Yanagi T, Nogi K, Yorimitsu H (2020). Metal-free synthesis of biaryls from aryl sulfoxides and sulfonanilides via sigmatropic rearrangement. Tetrahedron.

[52] Kinoshita J (2020). A route to indoles via modified fischer indole intermediates from sulfonanilides and ketene dithioacetal monoxides. Asian J. Org. Chem..

[53] Luo F (2018). Reductive *ortho* C–H cyanoalkylation of aryl(heteroaryl) sulfoxides: a general approach to α-aryl(heteroaryl) nitriles. Org. Chem. Front..

[54] Shang L (2017). Redox-neutral α-arylation of alkyl nitriles with aryl sulfoxides: a rapid electrophilic rearrangement. J. Am. Chem. Soc..

[55] Hu M (2020). [3,3]-Sigmatropic rearrangement of aryl fluoroalkyl sulfoxides with alkyl nitriles. Eur. J. Org. Chem..

[56] Macé Y, Urban C, Pradet C, Blazejewski J, Magnier E (2009). Aromatic and benzylic C–H bond functionalization upon reaction between nitriles and perfluoroalkyl sulfoxides. Eur. J. Org. Chem..

[57] Pégot B, Urban C, Diter P, Magnier E (2013). Perfluoroalkylated bis(sulfilimine)s and bis(sulfoximine)s by a Ritter-type reaction. Eur. J. Org. Chem..

[58] Chen M (2021). *Z*-selective α-arylation of α,β-unsaturated nitriles via [3,3]-sigmatropic rearrangement. Angew. Chem. Int. Ed..

[59] Okamoto K, Nogi K (2020). & Yorim itsu, H. Regioselective difunctionalization of 2,6-difluorophenols triggered by sigmatropic dearomatization. Org. Lett..

[60] Yang K, Pulis AP, Perry GJP, Procter DJ (2018). Transition-metal-free synthesis of C3-arylated benzofurans from benzothiophenes and phenols. Org. Lett..

[61] Huang X (2020). Dearomatization of aryl sulfoxides: a switch between mono- and dual-difluoroalkylation. Chem. Sci..

[62] Zhao W (2019). Dearomative dual functionalization of aryl iodanes. Angew. Chem., Int. Ed..

[63] Zhang L (2019). Selective [5,5]-sigmatropic rearrangement by assembly of aryl sulfoxides with allyl nitriles. Angew. Chem. Int. Ed..

[64] Walters MA (1996). Ab initio investigation of the 3-Aza-Cope reaction. J. Org. Chem..

[65] Walters MA, Hoem AB, McDonough CS (1996). Rearrangements of substituted 3-Aza−1,2,5-hexatrienes. 3. the scope and versatility of an extremely mild 3-Aza-Cope reaction. J. Org. Chem..

[66] Walters MA (1994). Ab initio investigation of three 3-Aza-Claisen variations. J. Am. Chem. Soc..

