# Supplementary material for: Dearomative di- and trifunctionalization of aryl sulfoxides via [5,5]-rearrangement
Source: Nat Commun. 2022 Aug 11;13:4719. doi: 10.1038/s41467-022-32426-6 (PMC9372148; doi:10.1038/s41467-022-32426-6)
Supplement: Supplementary file 1 — Supplementary Information [file 41467_2022_32426_MOESM1_ESM.pdf]

Supplementary Information

Dearomative Di- and Trifunctionalization of Aryl Sulfoxides via [5,5]-Rearrangement

Mengjie Hu[1,2], Yanping Liu[1], Yuchen Liang[1], Taotao Dong[1], Lichun Kong[1], Ming Bao[2], Zhi-Xiang Wang[3]* & Bo Peng[1]*

[1]Key Laboratory of the Ministry of Education for Advanced Catalysis Materials, Zhejiang Normal University, Jinhua 321004, China.

[2]State Key Laboratory of Fine Chemicals, Dalian University of Technology, Dalian 116023, China.

[3]School of Chemical Sciences, University of the Chinese Academy of Sciences, Beijing 100049, China.

pengbo@zjnu.cn

zxwang@ucas.ac.cn

Table of Contents

Supplementary Methods S2

1 General information S2

2 General procedure for the synthesis of starting materials S3

3 Optimization of reaction conditions S12

4 General procedure for the dearomative 3,4-dual functionalization of aryl sulfoxides S13

5 General procedure for the dearomative 1,4-dual functionalization and dearomative cyclization of aryl sulfoxides S41

6 General procedure for the one pot synthesis of cyclohexenones S76

7 Elaboration of 1,4-cyclohexediene and bicyclic products....................S83

8 Density functional theory (DFT) calculations and in situ NMR studies S92

9 NMR spectra S100

Supplementary References....................S226

Supplementary Methods

1 General information

Unless otherwise indicated, all glassware was oven dried before use and all reactions were performed under an atmosphere of Nitrogen. All solvents were distilled from appropriate drying agents prior to use. All reagents were used as received from commercial suppliers. Reaction progress was monitored by thin layer chromatography (TLC) performed on glass plates coated with silica gel GF254 with 0.2 mm thickness. Chromatograms were visualized by fluorescence quenching with UV light at 254 nm or by staining using potassium permanganate or phosphomolybdic acid. Compound isolation was performed on chromatography column using silica gel 60 (160-200 mesh) or Biotage Isolera Prime flash column system. Neat infrared spectra were recorded using a NEXUS670 FT-IR spectrometer. Wavelengths (ν) are reported in cm^{-1}. High-resolution mass spectrometry (HRMS) analysis was carried out using a TOF MS instrument with ESI source. All ^{1}H NMR, ^{13}C NMR and ^{19}F NMR spectra were recorded on Bruker AV-400 or AV-600. Chemical shifts were reported in parts per million (ppm), and the residual solvent peak was used as an internal reference: proton (chloroform δ 7.26), carbon (chloroform δ 77.16). Multiplicity was indicated as follows: s (singlet), d (doublet), t (triplet), q (quartet), m (multiplet), dd (doublet of doublet). Splitting patterns that could not be interpreted or easily visualized were designated as multiplet (m). Coupling constants were reported in Hertz (Hz).

2 General procedure for the synthesis of starting materials

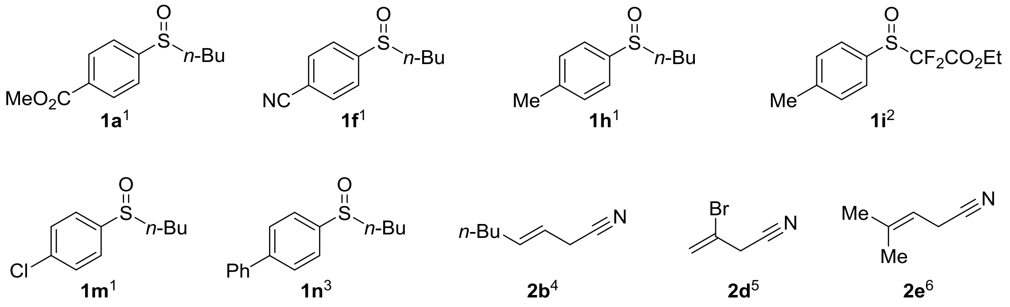

Supplementary Figure 1. The above aryl sulfoxides and nitriles are known compounds.

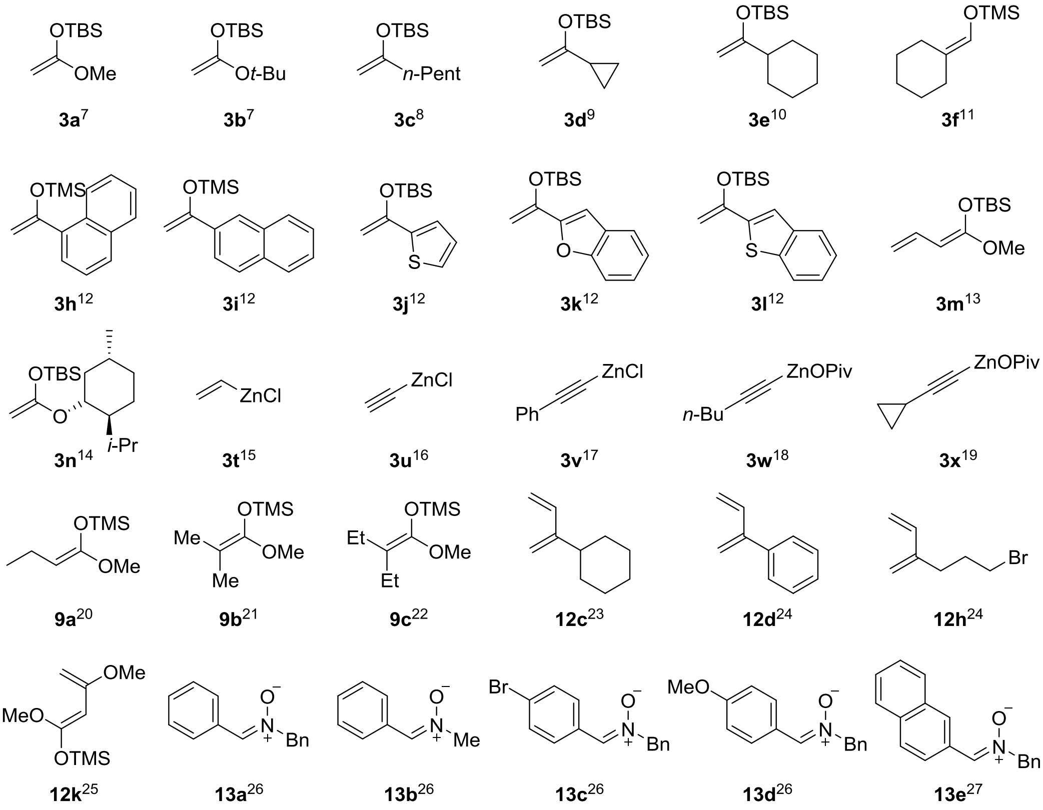

Supplementary Figure 2. The above nucleophiles are known compounds.

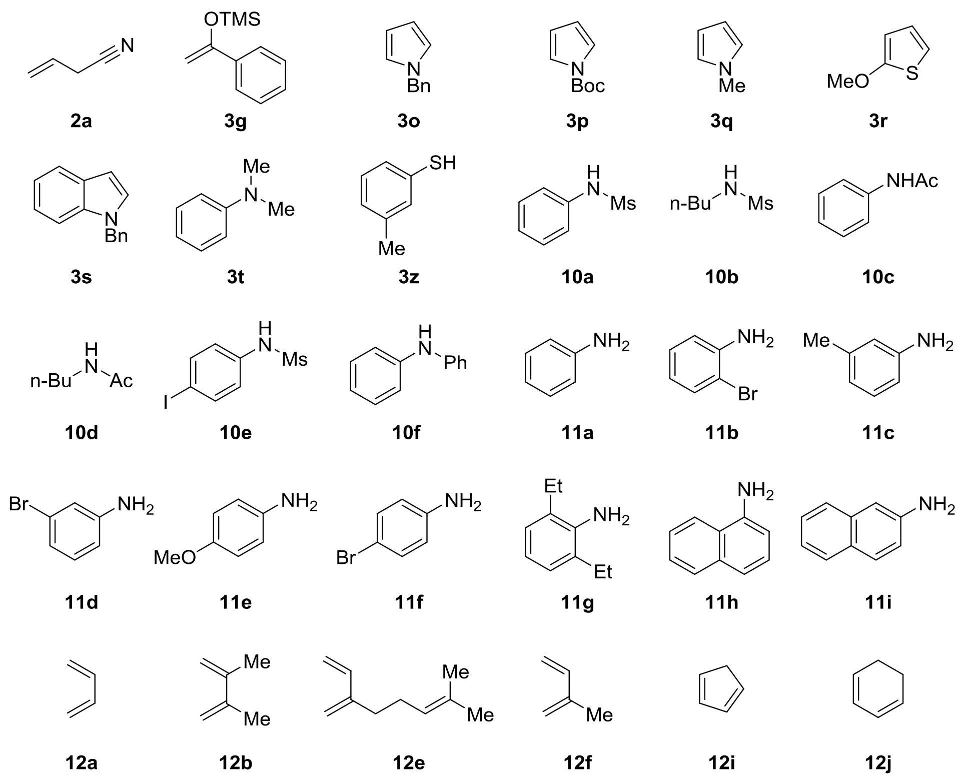

Supplementary Figure 3. The above nucleophiles are commercially available.

General procedure for the synthesis of aryl sulfoxide 1:

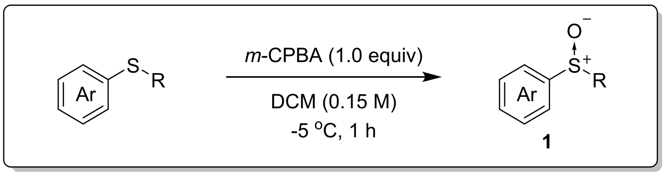

To a solution of aryl sulfide (5-12 mmol, 1.0 equiv) in DCM (0.3 M) was added a solution of *m*-CPBA (1.0 equiv) in DCM (0.3 M) dropwise at -5 °C. The resulting mixture was stirred at -5 °C for 1 h. Progress of the oxidation was checked by TLC. After completion of the reaction, a sat. aqueous $NaHCO_3$ solution was added to the reaction mixture and the resulting mixture was extracted with DCM. The combined organic layer was dried over Na_2SO_4 and concentrated under reduced pressure. The obtained residue was purified by silica gel chromatography eluting with an eluent (PE/EtOAc) to give the corresponding aryl sulfoxide **1**.

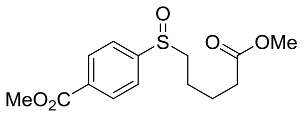

1b

Methyl 4-((5-methoxy-5-oxopentyl)sulfinyl)benzoate (1b)

Following the general procedure, the title compound was prepared

from 8 mmol of corresponding aryl sulfide and obtained as white solid, m.p. 66 – 68 ℃, 1.6 g, 65% yield. (Rf = 0.11, eluent: PE/EtOAc = 2/1)

^{1}H NMR (600 MHz, CDCl$_3$): δ 8.18 (d, *J* = 8.4 Hz, 2H), 7.68 (d, *J* = 8.4 Hz, 2H), 3.94 (s, 3H), 3.64 (s, 3H), 2.89 – 2.75 (m, 2H), 2.38 – 2.27 (m, 2H), 1.85 – 1.70 (m, 3H), 1.68 – 1.60 (m, 1H).

^{13}C NMR (151 MHz, CDCl$_3$): δ 173.4, 166.2, 149.1, 132.7, 130.5, 124.1, 56.7, 52.6, 51.8, 33.6, 24.0, 21.7.

IR (neat): 2950, 1719, 1594, 1434, 1277, 1216, 1041, 1012, 759, 721, 689 cm^{-1}.

HRMS (ESI-TOF) calcd. for $C_{14}H_{18}NaO_5S$ [M+Na]$^+$: 321.0767, found: 321.0768.

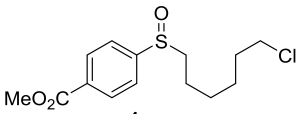

1c

Methyl 4-((6-chlorohexyl)sulfinyl)benzoate (1c)

Following the general procedure, the title compound was prepared from 7 mmol of corresponding aryl sulfide and obtained as white solid, m.p. 56 – 58 ℃, 1.6 g, 77% yield. (Rf = 0.23, eluent: PE/EtOAc = 2/1)

^{1}H NMR (600 MHz, CDCl$_3$): δ 8.17 (d, *J* = 8.4 Hz, 2H), 7.67 (d, *J* = 8.4 Hz, 2H), 3.93 (s, 3H), 3.49 (t, *J* = 6.6 Hz, 2H), 2.88 – 2.73 (m, 2H), 1.88 – 1.76 (m, 1H), 1.76 – 1.69 (m, 2H), 1.65 – 1.54 (m, 1H), 1.51 – 1.36 (m, 4H).

^{13}C NMR (151 MHz, CDCl$_3$): δ 166.1, 149.1, 132.6, 130.4, 124.1, 56.9, 52.6, 44.9, 32.2, 28.0, 26.5, 21.9.

IR (neat): 2935, 2863, 1716, 1594, 1432, 1295, 1084, 1030, 1014, 853, 719, 687 cm^{-1}.

HRMS (ESI-TOF) calcd. for $C_{14}H_{19}ClNaO_3S$ [M+Na]$^+$: 325.0616, found: 325.0633.

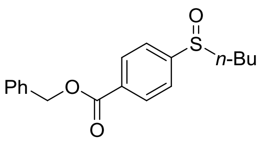

1d

Benzyl 4-(butylsulfinyl)benzoate (1d)

Following the general procedure, the title compound was prepared from 12 mmol of corresponding aryl sulfide and obtained as white solid, m.p. 47 – 49 ℃, 3.3 g, 86% yield. (Rf = 0.35, eluent: PE/EtOAc = 2/1)

^{1}H NMR (600 MHz, CDCl$_3$): δ 8.21 (d, *J* = 8.4 Hz, 2H), 7.68 (d, *J* = 8.4 Hz, 2H), 7.48 – 7.43 (m, 2H), 7.43 – 7.38 (m, 2H), 7.38 – 7.34 (m, 1H), 5.39 (s, 2H), 2.86 – 2.74 (m, 2H), 1.77 – 1.72 (m, 1H), 1.61 – 1.51 (m, 1H), 1.49 – 1.37 (m, 2H), 0.91 (t, *J* = 7.3 Hz, 3H).

^{13}C NMR (151 MHz, CDCl$_3$): δ 165.6, 149.5, 135.7, 132.6, 130.5, 128.8, 128.6, 128.5, 124.1, 67.3, 57.0, 24.0, 22.0, 13.8.

IR (neat): 2957, 2871, 1717, 1594, 1454, 1396, 1264, 1081, 1038, 759, 692 cm^{-1}.

HRMS (ESI-TOF) calcd. for $C_{18}H_{20}NaO_3S$ [M+Na]$^+$: 339.1025, found: 339.1025.

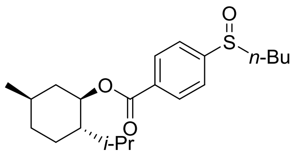

1e
(1/1 dr)

(1R,2S,5R)-2-isopropyl-5-methylcyclohexyl 4-(butylsulfinyl)benzoate (1e)

Following the general procedure, the title compound was prepared from 5 mmol of corresponding aryl sulfide and obtained as a mixture of two diastereoisomers (1/1 dr), white solid, m.p. 35 – 37 ℃, 1.3 g, 71% yield. (Rf = 0.53, eluent: PE/EtOAc = 2/1).

^{1}H NMR (600 MHz, CDCl$_3$): δ 8.17 (d, *J* = 8.0 Hz, 2H), 7.67 (d, *J* = 8.1 Hz, 2H), 4.99 – 4.90 (m, 1H), 2.86 – 2.73 (m, 2H), 2.14 – 2.07 (m, 1H), 1.98 – 1.89 (m, 1H), 1.79 – 1.70 (m, 3H), 1.61 – 1.52 (m, 3H), 1.50 – 1.37 (m, 2H), 1.17 – 1.06 (m, 2H), 0.95 – 0.89 (m, 10H), 0.78 (d, *J* = 6.9 Hz, 3H).

^{13}C NMR (151 MHz, CDCl$_3$): δ 165.2, 149.1, 133.3, 130.4, 124.0, 75.6, 57.02, 57.0, 47.3, 41.0, 34.4, 31.6, 26.64, 26.61, 24.0, 23.70, 23.67, 22.1, 22.0, 20.89, 20.87, 16.61, 16.59, 13.8.

IR (neat): 2957, 2871, 1716, 1595, 1457, 1397, 1370, 1274, 1172, 1116, 1087, 1042, 762 cm^{-1}.

HRMS (ESI-TOF) calcd. for $C_{21}H_{33}O_3S$ [M+H]$^+$: 365.2145, found: 365.2147.

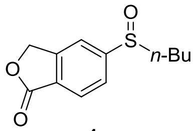

1g

5-(butylsulfinyl)isobenzofuran-1(3*H*)-one (1g)

Following the general procedure, the title compound was prepared from 6 mmol of corresponding aryl sulfide and obtained as white solid, m.p. 74 – 76 ℃, 1.2 g, 83% yield. (Rf = 0.17, eluent: PE/EtOAc = 2/1)

^{1}H NMR (600 MHz, CDCl$_3$): δ 8.02 (d, *J* = 7.9 Hz, 1H), 7.88 (s, 1H), 7.65 (d, *J* = 7.9 Hz, 1H), 5.39 (s, 2H), 2.91 – 2.84 (m, 1H), 2.83 – 2.76 (m, 1H), 1.83 – 1.76 (m, 1H), 1.62 – 1.53 (m, 1H), 1.52 – 1.38 (m, 2H), 0.92 (t, *J* = 7.3 Hz, 3H).

^{13}C NMR (151 MHz, CDCl$_3$): δ 169.9, 151.8, 147.5, 128.2, 126.5, 124.8, 118.3, 69.7, 57.1, 24.0, 21.9, 13.7.

IR (neat): 2960, 2933, 2873, 1763, 1612, 1453, 1420, 1353, 1316, 1265, 1212, 1038, 771 cm^{-1}.

HRMS (ESI-TOF) calcd. for $C_{12}H_{15}O_3S$ [M+H]$^+$: 239.0736, found: 239.0740.

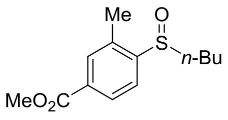

1j

Methyl 4-(butylsulfinyl)-3-methylbenzoate (1j)

Following the general procedure, the title compound was prepared from 9 mmol of corresponding aryl sulfide and obtained as colorless oil, 1.8 g, 78% yield. (Rf = 0.27, eluent: PE/EtOAc = 2/1)

^{1}H NMR (600 MHz, CDCl$_3$): δ 8.06 (d, *J* = 8.1 Hz, 1H), 7.97 (d, *J* = 8.3 Hz, 1H), 7.87 (s, 1H), 3.93 (s, 3H), 2.86 – 2.76 (m, 1H), 2.73 – 2.65 (m, 1H), 2.39 (s, 3H), 1.86 – 1.77 (m, 1H), 1.66 – 1.57 (m, 1H), 1.53 – 1.45 (m, 1H), 1.45 – 1.37 (m, 1H), 0.92 (t, *J* = 7.3 Hz, 3H).

^{13}C NMR (151 MHz, CDCl$_3$): δ 166.5, 147.7, 134.5, 132.2, 131.8, 128.2, 124.3, 55.0, 52.5, 24.4, 22.0, 18.3, 13.8.

IR (neat): 2958, 2873, 1725, 1598, 1568, 1437, 1400, 1286, 1251, 1196, 1077, 1037, 974 cm^{-1}.

HRMS (ESI-TOF) calcd. for $C_{13}H_{19}O_3S$ [M+H]$^+$: 255.1049, found: 255.1055.

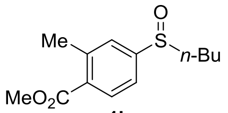

1k

Methyl 4-(butylsulfinyl)-2-methylbenzoate (1k)

Following the general procedure, the title compound was prepared from 7 mmol of corresponding aryl sulfide and obtained as white solid, m.p. 40 – 43 ℃, 1.3 g, 75% yield. (Rf = 0.25, eluent: PE/EtOAc = 2/1)

^{1}H NMR (600 MHz, CDCl$_3$): δ 7.98 (d, *J* = 8.1 Hz, 1H), 7.46 (s, 1H), 7.43 – 7.39 (m, 1H), 3.87 (s, 3H), 2.83 – 2.69 (m, 2H), 2.62 (s, 3H), 1.77 – 1.67 (m, 1H), 1.58 – 1.49 (m, 1H), 1.47 – 1.32 (m, 2H), 0.87 (t, *J* = 7.3 Hz, 3H).

^{13}C NMR (151 MHz, CDCl$_3$): δ 167.2, 147.9, 141.5, 131.9, 131.3, 126.7, 121.2, 56.8, 52.2, 24.0, 21.9, 21.8, 13.7.

IR (neat): 2952, 2871, 1713, 1594, 1563, 1435, 1381, 1250, 1099, 1076, 967, 772, 690 cm^{-1}.

HRMS (ESI-TOF) calcd. for $C_{13}H_{18}NaO_3S$ [M+Na]$^+$: 277.0869, found: 277.0876.

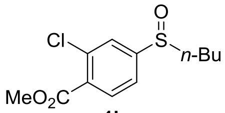
1l

Methyl 4-(butylsulfinyl)-2-chlorobenzoate (1l)

Following the general procedure, the title compound was prepared from 10 mmol of corresponding aryl sulfide and obtained as white solid, m.p. 54 – 55 ℃, 2.1 g, 75% yield. (Rf = 0.22, eluent: PE/EtOAc = 2/1)

^{1}H NMR (600 MHz, $CDCl_3$): δ 7.98 – 7.90 (m, 1H), 7.74 – 7.67 (m, 1H), 7.55 – 7.48 (m, 1H), 3.99 – 3.90 (m, 3H), 2.93 – 2.70 (m, 2H), 1.83 – 1.73 (m, 1H), 1.60 – 1.52 (m, 1H), 1.52 – 1.38 (m, 2H), 0.97 – 0.87 (m, 3H).

^{13}C NMR (151 MHz, $CDCl_3$): δ 165.5, 149.5, 135.1, 132.4, 132.1, 126.5, 122.1, 57.0, 52.9, 24.0, 22.0, 13.8.

IR (neat): 2954, 2870, 1730, 1585, 1433, 1373, 1261, 1238, 1120, 1030, 960, 839, 770 cm^{-1}.

HRMS (ESI-TOF) calcd. for $C_{12}H_{15}ClNaO_3S$ $[M+Na]^+$: 297.0323, found: 297.0326.

The procedure for the synthesis of (*E*)-9-bromonon-3-enenitrile (2c):

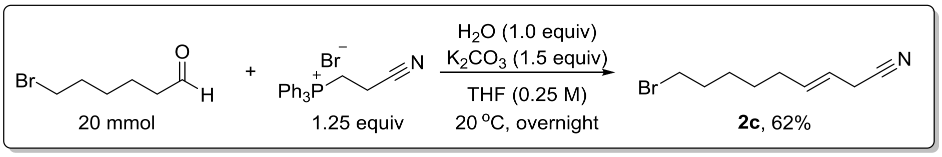

A mixture of 6-bromohexanal (3.6 g, 20.0 mmol), phosphonium salt (9.9 g, 1.25 equiv), K_2CO_3 (4.1 g, 1.5 equiv.), H_2O (360 μL, 1.0 equiv) in THF (80 mL) was stirred at rt for 12 h. After that, the reaction mixture was diluted with EtOAc and washed with brine. The organic layer was separated, dried over Na_2SO_4, and concentrated. The obtained residue was then purified by flash chromatography on silica gel affording the title compound (2.68 g, 62% yield) as light yellow oil. (Rf = 0.21, eluent: PE/EtOAc = 20/1)

^{1}H NMR (400 MHz, $CDCl_3$): δ 5.77 – 5.60 (m, 1H), 5.48 – 5.34 (m, 1H), 3.41 (t, *J* = 6.7 Hz, 2H), 3.09 (d, *J* = 6.9 Hz, 2H), 2.14 – 2.02 (m, 2H), 1.93 – 1.81 (m, 2H), 1.51 – 1.38 (m, *J* = 7.5, 6.3 Hz, 4H).

^{13}C NMR (101 MHz, $CDCl_3$): δ 135.8, 118.3, 117.3, 33.8, 32.6, 28.2, 27.8, 27.3, 15.7.

IR (neat): 3029, 2933, 2858, 2359, 2250, 1458, 1417, 1267, 1240, 1024, 906, 803 cm^{-1}.

HRMS (ESI-TOF) calcd. for $C_9H_{14}BrNNa$ $[M+Na]^+$: 238.0202, found: 238.0197.

General procedure for the synthesis of enol silyl ethers 9:

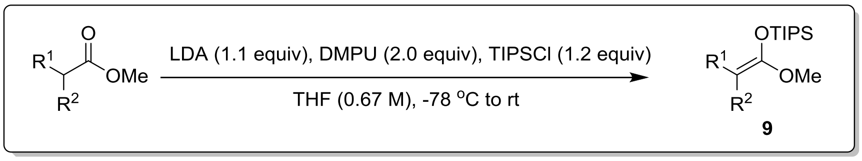

n-BuLi (2.5 M solution in hexane, 13.2 mL, 33mmol, 1.1 equiv) was added dropwise to a solution of *i*-Pr_2NH (5.0 mL, 36 mmol, 1.2 equiv) in THF (30 mL) at -78 ℃. The mixture was stirred at 0 ℃ for 15 mins and then cooled to -78 ℃. To the mixture was added corresponding ester (30 mmol, 1.0 equiv). The mixture was stirred at -78 ℃ for 30 min. After that, 1,3-dimethyl-3,4,5,6-tetrahydro-2(1*H*)-pyrimidinone (DMPU; 7.2 mL, 60 mmol, 2.0 equiv) and a solution of triisopropylsilyl chloride (7.7 mL, 36 mmol, 1.2 equiv) in THF (15 mL) were added to the mixture. The mixture was then warmed up to rt. After stirred overnight, the mixture was concentrated in *vacuo* to remove THF. The obtained residue was dissolved by petroleum ether and then washed by sat. aqueous $NaHCO_3$ solution. The organic layer was further washed with water, sat. aquoues $CuSO_4$ solution, water and brine in order. After drying over $MgSO_4$ and concentration, the crude product was purified by distillation under reduced pressure to afford the corresponding enol silyl ethers **9**.

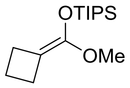

9d

(cyclobutylidene(methoxy)methoxy)triisopropylsilane (9d)

Following the general procedure, the title compound was purified by distillation (bp 106-108 ℃ at 3 Torr) and obtained as a colorless liquid (3.7 g, 13.5 mmol, 45%).

^{1}H NMR (600 MHz, $CDCl_3$): δ 3.61 (s, 3H), 2.85 – 2.78 (m, 2H), 2.66 – 2.60 (m, 2H), 1.92 (t, *J* = 7.8 Hz, 2H), 1.13 – 1.07 (m, 21H).

^{13}C NMR (151 MHz, $CDCl_3$): δ 148.7, 89.7, 56.6, 27.3, 27.0, 18.4, 17.9, 12.8.

IR (neat): 2943, 2865, 1708, 1463, 1251, 1132, 1105, 997, 881, 804 cm^{-1}.

HRMS (ESI-TOF) calcd. for $C_{15}H_{31}O_2Si$ $[M+H]^+$: 271.2088, found: 271.2073.

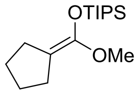

9e

(cyclopentylidene(methoxy)methoxy)triisopropylsilane (9e):

Following the general procedure, the title compound was purified by distillation (bp 115-116 ℃ at 3 Torr) and obtained as a colorless liquid (6.2 g, 21.9 mmol, 73%).

^{1}H NMR (600 MHz, $CDCl_3$): δ 3.60 (s, 3H), 2.27 – 2.15 (m, 4H), 1.62 – 1.56 (m, 4H), 1.18 – 1.08 (m, 21H).

^{13}C NMR (151 MHz, $CDCl_3$): δ 148.7, 100.5, 57.6, 28.8, 27.7, 27.3, 26.9, 18.0, 13.0.

IR (neat): 2942, 2865, 1707, 1463, 1215, 1174, 1082, 969, 881, 806 cm^{-1}.

HRMS (ESI-TOF) calcd. for $C_{16}H_{33}O_2Si$ $[M+H]^+$: 285.2244, found: 285.2248.

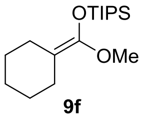

9f

(cyclohexylidene(methoxy)methoxy)triisopropylsilane (9f): Following the general procedure, the title compound was purified by distillation (bp 127-129 °C at 3 Torr) and obtained as a colorless liquid (7.2 g, 24.0 mmol, 80%).

^{1}H NMR (600 MHz, $CDCl_3$): δ 3.55 (s, 3H), 2.15 – 2.02 (m, 4H), 1.53 – 1.40 (m, 6H), 1.21 – 1.07 (m, 21H).

^{13}C NMR (151 MHz, $CDCl_3$): δ 148.9, 99.4, 58.9, 27.8, 27.2, 27.1, 27.0, 26.9, 18.0, 12.9.

IR (neat): 2922, 2866, 1692, 1463, 1257, 1164, 1060, 945, 881, 839 cm^{-1}.

HRMS (ESI-TOF) calcd. for $C_{17}H_{35}O_2Si$ $[M+H]^+$: 299.2401, found: 299.2407.

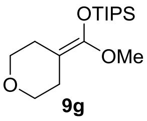

9g

Triisopropyl(methoxy(tetrahydro-4*H*-pyran-4-ylidene)methoxy)silane (9g): Following the general procedure, the title compound was purified by distillation (bp 133-134 °C at 3 Torr) and obtained as a colorless liquid (5.0 g, 16.5 mmol, 55%).

^{1}H NMR (600 MHz, $CDCl_3$): δ 3.63 – 3.56 (m, 4H), 3.54 (s, 3H), 2.19 (t, *J* = 5.4 Hz, 4H), 1.19 – 1.04 (m, 21H).

^{13}C NMR (151 MHz, $CDCl_3$): δ 150.0, 94.2, 68.8, 68.3, 58.9, 27.7, 27.4, 17.9, 12.9.

IR (neat): 2945, 2866, 1696, 1463, 1236, 1164, 1102, 1067, 882, 845, 804 cm^{-1}.

HRMS (ESI-TOF) calcd. for $C_{16}H_{33}O_3Si$ $[M+H]^+$: 301.2193, found: 301.2199.

The procedure for the synthesis of benzyl (4-methylenehex-5-en-1-yl) carbonate (12g):

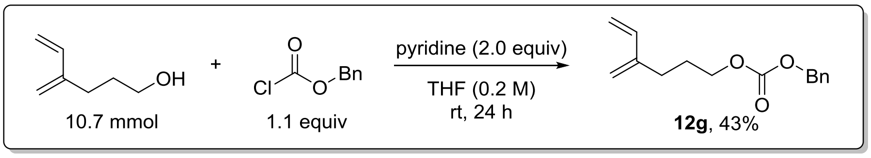

To a solution of 4-methylenehex-5-en-1-ol (1.2 g, 10.7 mmol) in THF (50 mL) were added pyridine (1.73 mL, 2.0 equiv) and benzyl chloroformate (2.0 g, 1.1 equiv). After stirring for 24 hours at room temperature, 1 N HCl was added and the aqueous phase was extracted with EtOAc. The organic phases were combined, washed with a sat. solution of $NaHCO_3$ and brine, dried over Na_2SO_4, and concentrated. The obtained residue was then purified by flash chromatography on silica gel affording the title compound (1.13 g, 43%) as colorless oil. (Rf = 0.63, eluent: PE/EtOAc = 10/1)

^{1}H NMR (400 MHz, $CDCl_3$): δ 7.48 – 7.29 (m, 5H), 6.38 (dd, *J* = 17.6, 10.8 Hz, 1H), 5.29 – 5.13 (m, 3H), 5.13 – 4.97 (m, 3H), 4.20 (t, *J* = 6.5 Hz, 2H), 2.38 – 2.26 (m, 2H), 1.97 – 1.82 (m, 2H).

^{13}C NMR (101 MHz, $CDCl_3$): δ 155.3, 145.0, 138.6, 135.4, 128.7, 128.6, 128.4, 116.4, 113.6, 69.6, 67.8, 27.4, 27.1.

IR (neat): 2961, 2359, 1741, 1595, 1498, 1456, 1397, 1245, 899, 790 cm^{-1}.

HRMS (ESI-TOF) calcd. for $C_{15}H_{18}NaO_3$ $[M+Na]^+$: 269.1148, found: 269.1147.

3 Optimization of reaction conditions

Supplementary Table 1. Optimization of reaction conditions

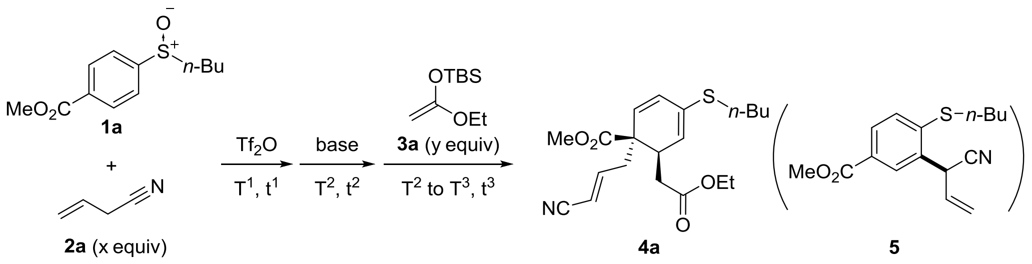

entry	base	T^1, t^1	T^2, t^2	T^3, t^3	x	y	yield (%)[a]
1	DIPEA	-50 °C, 18 h	-95 °C, 30 min	-70 °C, 12 h	3.0	2.0	40
2	DABCO	-50 °C, 18 h	-95 °C, 30 min	-70 °C, 12 h	3.0	2.0	trace
3	4-methylmorpholine	-50 °C, 18 h	-95 °C, 30 min	-70 °C, 12 h	3.0	2.0	33
4	4-ethylmorpholine	-50 °C, 18 h	-95 °C, 30 min	-70 °C, 12 h	3.0	2.0	70
5	2-methylpyridine	-50 °C, 18 h	-95 °C, 30 min	-70 °C, 12 h	3.0	2.0	34
6	Et_3N	-50 °C, 18 h	-95 °C, 30 min	-70 °C, 12 h	3.0	2.0	63
7	DBU	-50 °C, 18 h	-95 °C, 30 min	-70 °C, 12 h	3.0	2.0	38
8	4-ethylmorpholine	-40 °C, 18 h	-95 °C, 30 min	-70 °C, 12 h	3.0	2.0	30
9	4-ethylmorpholine	-60 °C, 18 h	-95 °C, 30 min	-70 °C, 12 h	3.0	2.0	66
10	4-ethylmorpholine	-55 °C, 18 h	-95 °C, 30 min	-70 °C, 12 h	3.0	2.0	79
11	4-ethylmorpholine	-55 °C, 12 h	-95 °C, 30 min	-70 °C, 12 h	3.0	2.0	50
12	4-ethylmorpholine	-55 °C, 18 h	-95 °C, 1.0 h	-70 °C, 12 h	3.0	2.0	70
13	4-ethylmorpholine	-55 °C, 18 h	-95 °C, 3.0 h	-70 °C, 12 h	3.0	2.0	69
14	4-ethylmorpholine	-55 °C, 18 h	-78 °C, 30 min	-70 °C, 12 h	3.0	2.0	74
15	4-ethylmorpholine	-55 °C, 18 h	-95 °C, 30 min	-60 °C, 12 h	3.0	2.0	54
16	4-ethylmorpholine	-55 °C, 18 h	-95 °C, 30 min	-80 °C, 12 h	3.0	2.0	71
17	4-ethylmorpholine	-55 °C, 18 h	-95 °C, 30 min	-95 °C, 12 h	3.0	2.0	53
18	4-ethylmorpholine	-55 °C, 18 h	-95 °C, 30 min	-70 °C, 6 h	3.0	2.0	72
19	4-ethylmorpholine	-55 °C, 18 h	-95 °C, 30 min	-70 °C, 12 h	2.5	2.0	67
20	4-ethylmorpholine	-55 °C, 18 h	-95 °C, 30 min	-70 °C, 12 h	3.0	2.5	79
21	4-ethylmorpholine	-55 °C, 18 h	-95 °C, 30 min	-70 °C, 12 h	3.0	1.5	54
22	4-ethylmorpholine	-55 °C, 18 h	-95 °C, 30 min	-70 °C, 12 h	3.0	2.0	81(73)[b]

[a]NMR yields using mesitylene as internal standard and isolated yields given in parentheses. [b]Base and Nu were added in one portion. *Ortho*-cyanoalkylated product **5** was obtained in 6% yield.

To a mixture of **1a** (120 mg, 0.5 mmol) and **2a** (121 μL, 1.5 mmol) in DCM (3.0 mL) was added Tf_2O (126 μL, 0.75 mmol) at -78 °C under N_2 atmosphere. The mixture was gradually warmed to T^1 (°C). After stirring for t^1 (hrs), the mixture was cooled to T^2 (°C). A solution of base (1.25 mmol) in DCM (1.0 mL) was added dropwise to the mixture in 10 min using syringe pump. After stirring for t^2 (hrs), to the mixture was added a solution of **3a** in DCM (1.0 mL) dropwise in 10 min using syringe pump. The mixture was gradually warmed to T^3 (°C) and stirred for t^3 (hrs). Then the mixture was passed through a short silica gel column, concentrated under vacuum and prepared for NMR analysis with mesitylene as internal standard. For entry 22, the obtained mixture was further purified by flash chromatography on silica gel giving product **4a** and *ortho*-alkylated product **5**.

4 General procedure for the dearomative 3,4-dual functionalization of aryl sulfoxides

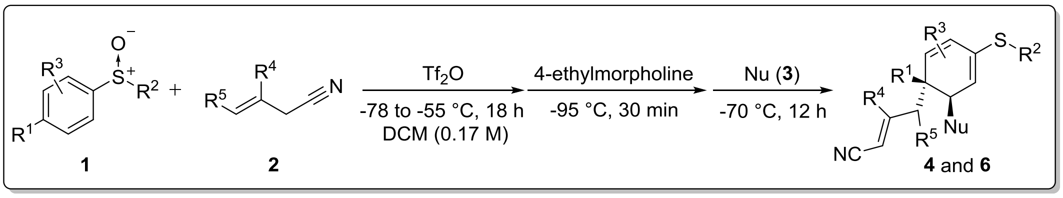

To a mixture of aryl sulfoxide **1** (0.5 mmol) and allyl nitrile **2** (1.5 mmol, 3.0 equiv) in DCM (3.0 mL) was added Tf_2O (126 μL, 0.75 mmol, 1.5 equiv) at -78 °C under N_2 amosphere. The mixture was gradually warmed to -55 °C. After stirring for 18 h, the mixture was cooled to -95 °C.

Subsequent procedures for the cases of **4a-4t**, **4z**, **6a-6i**, **6l-6q**: to the mixture of sulfoxide **1**, allyl nitrile **2** and Tf_2O was added a mixture of 4-ethylmorpholine (157 μL, 1.25 mmol, 2.5 equiv) and nucleophile **3a-3n**, **3o-3t**, or **3z** (1.0 mmol, 2.0 equiv) in DCM (2.0 mL) dropwise in 20 min using syringe pump. After stirring for 30 min, the mixture was gradually warmed to -70 °C and further stirred for 12 h. Then the mixture was passed through a short silica gel column and concentrated under vacuum. The obtained residue was further purified by flash chromatography on silica gel affording the title compound.

Subsequent procedures for the cases of **4u-4y**, **4a'**, **4b', 6j** and **6k**: to the mixture of sulfoxide **1,** allyl nitrile **2** and Tf_2O was added a solution of 4-ethylmorpholine (157 μL, 2.5 equiv) in DCM (1.0 mL) dropwise in 10 min using syringe pump. After stirring for 30 min, to the mixture was

added a solution of nucleophile **3u-3y**, **3a'** or **3b'** (1.5 mmol, 3.0 equiv) in THF dropwise in 10 min using syringe pump. The mixture was gradually warmed to -70 ℃ and further stirred for 12 h. Then the mixture was passed through a short silica gel column and concentrated under vacuum. The obtained residue was further purified by flash chromatography on silica gel affording the title compound.

Following the general procedure, **4a** and *ortho*-alkylated product **5** were obtained from the reaction of **1a** with **3a**.

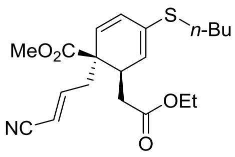

4a

Methyl (1*S*,6*R*)-4-(butylthio)-1-((*E*)-3-cyanoallyl)-6-(2-ethoxy-2-oxoethyl)cyclohexa-2,4-diene-1-carboxylate (4a):

The title compound was obtained as colorless oil, 137.8 mg, 73% yield. (Rf = 0.15, eluent: PE/EtOAc = 5/1).

^{1}H NMR (600 MHz, CDCl$_3$): δ 6.58 – 6.50 (m, 1H), 6.07 – 6.02 (m, 1H), 5.94 – 5.90 (m, 1H), 5.63 – 5.58 (m, 1H), 5.32 – 5.26 (m, 1H), 4.12 – 4.03 (m, 2H), 3.73 (s, 3H), 2.96 – 2.87 (m, 1H), 2.83 – 2.77 (m, 1H), 2.72 – 2.61 (m, 2H), 2.54 – 2.47 (m, 1H), 2.43 – 2.35 (m, 1H), 2.15 – 2.08 (m, 1H), 1.60 – 1.51 (m, 2H), 1.45 – 1.35 (m, 2H), 1.22 (t, *J* = 7.1 Hz, 3H), 0.90 (t, *J* = 7.4 Hz, 3H).

^{13}C NMR (151 MHz, CDCl$_3$): δ 173.4, 171.6, 150.9, 131.1, 127.8, 126.5, 119.4, 116.9, 102.9, 60.7, 52.5, 50.7, 40.5, 38.6, 35.1, 30.9, 30.7, 22.1, 14.3, 13.7.

IR (neat): 2956, 2872, 2224, 1726, 1211, 1176, 1027, 969, 754 cm^{-1}.

HRMS (ESI-TOF) calcd. for $C_{20}H_{27}NNaO_4S$ [M+Na]$^+$: 400.1553, found: 400.1570.

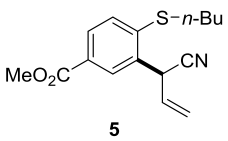

5

Methyl 4-(butylthio)-3-(1-cyanoallyl)benzoate (5):

The title compound was obtained as colorless oil, 8.7 mg, 6% yield. (Rf = 0.55, eluent: PE/EtOAc = 5/1).

^{1}H NMR (600 MHz, CDCl$_3$): δ 8.06 (s, 1 H), 7.96 (d, *J* = 8.3 Hz, 1H), 7.39 (d, *J* = 8.3 Hz, 1H), 5.91 – 5.82 (m, 1H), 5.60 (d, *J* = 16.9 Hz, 1H), 5.42 (d, *J* = 10.0 Hz, 1H), 5.13 (d, *J* = 5.1 Hz, 1H),

3.91 (s, 3H), 3.02 (t, *J* = 7.4 Hz, 2H), 1.72 – 1.65 (m, 2H), 1.52 – 1.44 (m, 2H), 0.95 (t, *J* = 7.4 Hz, 3H).

^{13}C NMR (151 MHz, CDCl$_3$): δ 166.3, 142.7, 133.4, 130.6, 130.0, 129.4, 128.0, 127.7, 119.3, 118.1, 52.4, 37.6, 33.4, 30.6, 22.1, 13.3.

IR (neat): 2957, 2930, 2237, 1721, 1600, 1506, 1435, 1407, 1292, 1252, 1117, 1053, 738 cm^{-1}.

HRMS (ESI-TOF) calcd. for $C_{16}H_{19}NO_2S$ [M+H]$^+$: 290.1209, found: 290.1217.

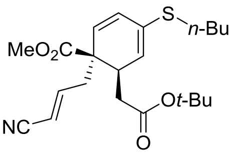

4b

Methyl (1*S*,6*R*)-6-(2-(*tert*-butoxy)-2-oxoethyl)-4-(butylthio)-1-((*E*)-3-cyanoallyl)cyclohexa-2,4-diene-1-carboxylate (4b):

Following the general procedure, the title compound was obtained as colorless oil, 131.8 mg, 65% yield. (Rf = 0.37, eluent: PE/EtOAc = 5/1).

^{1}H NMR (600 MHz, CDCl$_3$): δ 6.57 – 6.49 (m, 1H), 6.01 (d, *J* = 9.9 Hz, 1H), 5.93 – 5.87 (m, 1H), 5.64 – 5.57 (m, 1H), 5.28 (d, *J* = 16.2 Hz, 1H), 3.72 (s, 3H), 2.87 – 2.81 (m, 1H), 2.81 – 2.75 (m, 1H), 2.68 – 2.62 (m, 2H), 2.52 – 2.46 (m, 1H), 2.32 – 2.25 (m, 1H), 2.05 – 1.99 (m, 1H), 1.58 – 1.52 (m, 2H), 1.42 – 1.36 (m, 11H), 0.88 (t, *J* = 7.4 Hz, 3H).

^{13}C NMR (151 MHz, CDCl$_3$): δ 173.5, 170.8, 151.0, 130.9, 127.7, 126.4, 119.5, 116.9, 102.8, 80.9, 52.5, 50.6, 40.5, 38.7, 36.2, 30.9, 30.7, 28.1, 22.1, 13.7.

IR (neat): 2957, 2930, 2224, 1725, 1367, 1150, 969, 912 cm^{-1}.

HRMS (ESI-TOF) calcd. for $C_{22}H_{31}NNaO_4S$ [M+Na]$^+$: 428.1850, found: 428.1866.

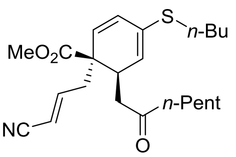

4c

Methyl (1*S*,6*R*)-4-(butylthio)-1-((*E*)-3-cyanoallyl)-6-(2-oxoheptyl)cyclohexa-2,4-diene-1-carboxylate (4c):

Following the general procedure, the title compound was obtained as colorless oil, 121.0 mg, 60% yield. (Rf = 0.34, eluent: PE/EtOAc = 5/1).

^{1}H NMR (600 MHz, CDCl$_3$): δ 6.58 – 6.50 (m, 1H), 6.03 (d, *J* = 9.9 Hz, 1H), 5.94 – 5.88 (m, 1H), 5.63 – 5.57 (m, 1H), 5.34 – 5.24 (m, 1H), 3.71 (s, 3H), 3.00 – 2.94 (m, 1H), 2.82 – 2.77 (m, 1H), 2.67 – 2.62 (m, 2H), 2.61 – 2.54 (m, 1H), 2.52 – 2.47 (m, 1H), 2.33 – 2.23 (m, 2H), 2.19 –

2.13 (m, 1H), 1.56 – 1.46 (m, 4H), 1.42 – 1.37 (m, 2H), 1.29 – 1.24 (m, 2H), 1.23 – 1.17 (m, 2H), 0.90 (t, *J* = 7.4 Hz, 3H), 0.85 (t, *J* = 7.2 Hz, 3H).

^{13}C NMR (151 MHz, CDCl$_3$): δ 208.9, 173.7, 151.1, 130.4, 127.8, 126.4, 120.2, 117.0, 102.8, 52.5, 50.7, 43.5, 43.2, 40.4, 37.7, 31.4, 30.9, 30.7, 23.5, 22.5, 22.1, 14.0, 13.8.

IR (neat): 2955, 2929, 2871, 2224, 1727, 1435, 1377, 1213, 1060, 970, 911 cm^{-1}.

HRMS (ESI-TOF) calcd. for $C_{23}H_{33}NNaO_3S$ [M+Na]$^+$: 426.2073, found: 426.2057.

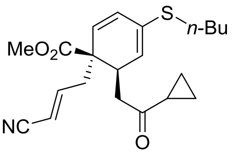

4d

Methyl (1*S*,6*R*)-4-(butylthio)-1-((*E*)-3-cyanoallyl)-6-(2-cyclopropyl-2-oxoethyl) cyclohexa-2,4-diene-1-carboxylate (4d):

Following the general procedure, the title compound was obtained as colorless oil, 139.9 mg, 75% yield. (Rf = 0.26, eluent: PE/EtOAc = 5/1).

^{1}H NMR (600 MHz, CDCl$_3$): δ 6.57 – 6.50 (m, 1H), 6.04 (d, *J* = 9.9 Hz, 1H), 5.93 – 5.89 (m, 1H), 5.61 – 5.56 (m, 1H), 5.28 (d, *J* = 16.2 Hz, 1H), 3.71 (s, 3H), 3.00 – 2.93 (m, 1H), 2.82 – 2.76 (m, 1H), 2.75 – 2.68 (m, 1H), 2.67 – 2.59 (m, 2H), 2.52 – 2.46 (m, 1H), 2.35 – 2.29 (m, 1H), 1.80 – 1.75 (m, 1H), 1.56 – 1.50 (m, 2H), 1.41 – 1.34 (m, 2H), 0.99 – 0.91 (m, 2H), 0.88 (t, *J* = 7.4 Hz, 3H), 0.85 – 0.81 (m, 2H).

^{13}C NMR (151 MHz, CDCl$_3$): δ 208.4, 173.7, 151.1, 130.2, 127.7, 126.4, 120.3, 116.9, 102.7, 52.5, 50.6, 43.9, 40.4, 37.8, 30.8, 30.7, 22.1, 21.0, 13.7, 11.04, 11.02.

IR (neat): 2955, 2929, 2223, 1728, 1695, 1386, 1197, 1082, 970, 907 cm^{-1}.

HRMS (ESI-TOF) calcd. for $C_{21}H_{27}NNaO_3S$ [M+Na]$^+$: 396.1604, found: 396.1587.

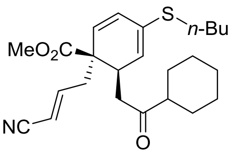

4e

Methyl (1*S*,6*R*)-4-(butylthio)-1-((*E*)-3-cyanoallyl)-6-(2-cyclohexyl-2-oxoethyl)cyclohexa-2,4-diene-1-carboxylate (4e):

Following the general procedure, the title compound was obtained as yellow oil, 151.7 mg, 73% yield. (Rf = 0.31, eluent: PE/EtOAc = 5/1).

^{1}H NMR (600 MHz, CDCl$_3$): δ 6.56 – 6.48 (m, 1H), 6.02 (d, *J* = 9.9 Hz, 1H), 5.92 – 5.87 (m, 1H), 5.56 (d, *J* = 6.3 Hz, 1H), 5.27 (d, *J* = 16.3 Hz, 1H), 3.69 (s, 3H), 3.00 – 2.93 (m, 1H), 2.81 –

2.75 (m, 1H), 2.66 – 2.59 (m, 3H), 2.51 – 2.45 (m, 1H), 2.21 – 2.10 (m, 2H), 1.73 – 1.69 (m, 3H), 1.61 (d, *J* = 11.8 Hz, 1H), 1.55 – 1.48 (m, 2H), 1.41 – 1.35 (m, 2H), 1.29 – 1.11 (m, 6H), 0.88 (t, *J* = 7.4 Hz, 3H).

^{13}C NMR (151 MHz, CDCl$_3$): δ 211.7, 173.7, 151.1, 130.2, 127.8, 126.4, 120.4, 116.9, 102.7, 52.5, 51.2, 50.7, 41.2, 40.4, 37.6, 30.8, 30.7, 28.5, 28.2, 25.8, 25.7, 25.5, 22.1, 13.7.

IR (neat): 2928, 2854, 2224, 1728, 1705, 1449, 1202, 969, 910 cm^{-1}.

HRMS (ESI-TOF) calcd. for $C_{24}H_{33}NNaO_3S$ [M+Na]$^+$: 438.2073, found: 438.2088.

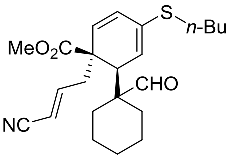

4f

Methyl (1*S*,2*S*)-5-(butylthio)-2-((*E*)-3-cyanoallyl)-1'-formyl-[1,1'-bi(cyclohexane)]-3,5-diene-2-carboxylate (4f):

Following the general procedure, the title compound was obtained as colorless oil, 134.4 mg, 67% yield. (Rf = 0.26, eluent: PE/EtOAc = 5/1).

^{1}H NMR (600 MHz, CDCl$_3$): δ 9.26 (s, 1H), 6.50 – 6.41 (m, 1H), 6.23 (d, *J* = 9.9 Hz, 1H), 5.91 (d, *J* = 9.9 Hz, 1H), 5.54 (d, *J* = 6.5 Hz, 1H), 5.25 (d, *J* = 16.2 Hz, 1H), 3.66 (s, 3H), 2.92 – 2.84 (m, 1H), 2.80 – 2.69 (m, 2H), 2.66 (d, *J* = 6.5 Hz, 1H), 2.52 – 2.45 (m, 1H), 1.90 (d, *J* = 13.2 Hz, 1H), 1.70 (d, *J* = 12.0 Hz, 1H), 1.64 – 1.59 (m, 2H), 1.56 – 1.35 (m, 7H), 1.29 – 1.24 (m, 1H), 1.05 – 0.98 (m, 2H), 0.93 (t, *J* = 7.3 Hz, 3H).

^{13}C NMR (151 MHz, CDCl$_3$): δ 203.5, 173.7, 150.9, 132.9, 130.3, 126.3, 116.9, 103.2, 56.4, 52.3, 49.5, 48.3, 43.5, 31.2, 31.1, 29.7, 27.4, 25.4, 22.8, 22.14, 22.09, 13.8.

IR (neat): 2926, 2855, 2223, 1723, 1631, 1451, 1196, 1058, 970, 912 cm^{-1}.

HRMS (ESI-TOF) calcd. for $C_{23}H_{31}NNaO_3S$ [M+Na]$^+$: 424.1917, found: 424.1957.

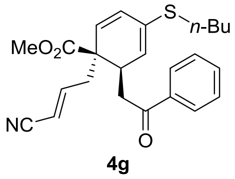

4g

Methyl (1*S*,6*R*)-4-(butylthio)-1-((*E*)-3-cyanoallyl)-6-(2-oxo-2-phenylethyl)cyclohexa-2,4-diene-1-carboxylate (4g):

Following the general procedure, the title compound was obtained as colorless oil, 159.6 mg, 78% yield. (Rf = 0.25, eluent: PE/EtOAc = 5/1).

^{1}H NMR (600 MHz, CDCl$_3$): δ 7.86 (d, *J* = 8.3 Hz, 2H), 7.57 – 7.52 (m, 1H), 7.45 – 7.40 (m, 2H), 6.61 – 6.54 (m, 1H), 6.11 (d, *J* = 9.9 Hz, 1H), 5.95 (d, *J* = 9.9 Hz, 1H), 5.65 (d, *J* = 5.6 Hz, 1H), 5.31 (d, *J* = 16.2 Hz, 1H), 3.70 (s, 3H), 3.24 – 3.15 (m, 2H), 2.89 – 2.83 (m, 1H), 2.68 (d, *J* = 13.7 Hz, 1H), 2.62 – 2.53 (m, 3H), 1.51 – 1.44 (m, 2H), 1.37 – 1.29 (m, 2H), 0.83 (t, *J* = 7.4 Hz, 3H).

^{13}C NMR (151 MHz, CDCl$_3$): δ 197.9, 173.7, 151.1, 136.7, 133.4, 130.6, 128.7, 128.2, 127.9, 126.5, 120.1, 117.0, 102.8, 52.6, 50.9, 40.5, 39.4, 38.1, 30.8, 30.6, 22.1, 13.7.

IR (neat): 2955, 2871, 2223, 1727, 1683, 1596, 1448, 1213, 969, 911 cm^{-1}.

HRMS (ESI-TOF) calcd. for $C_{24}H_{27}NNaO_3S$ [M+Na]$^+$: 432.1604, found: 432.1591.

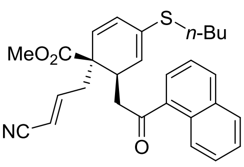

4h

Methyl (1*S*,6*R*)-4-(butylthio)-1-((*E*)-3-cyanoallyl)-6-(2-(naphthalen-1-yl)-2-oxoethyl)cyclohexa-2,4-diene-1-carboxylate (4h):

Following the general procedure, the title compound was obtained as colorless oil, 190.6 mg, 83% yield. (Rf = 0.20, eluent: PE/EtOAc = 5/1).

^{1}H NMR (600 MHz, CDCl$_3$): δ 8.56 (d, *J* = 8.6 Hz, 1H), 7.97 (d, *J* = 8.2 Hz, 1H), 7.86 (d, *J* = 8.1 Hz, 1H), 7.74 (d, *J* = 7.1 Hz, 1H), 7.62 – 7.56 (m, 1H), 7.55 – 7.50 (m, 1H), 7.49 – 7.44 (m, 1H), 6.64 – 6.55 (m, 1H), 6.13 (d, *J* = 9.9 Hz, 1H), 6.00 – 5.94 (m, 1H), 5.71 (d, *J* = 5.9 Hz, 1H), 5.33 (d, *J* = 16.2 Hz, 1H), 3.73 (s, 3H), 3.35 – 3.24 (m, 2H), 2.92 – 2.84 (m, 1H), 2.82 – 2.76 (m, 1H), 2.65 – 2.54 (m, 3H), 1.55 – 1.46 (m, 2H), 1.38 – 1.29 (m, 2H), 0.82 (t, *J* = 7.4 Hz, 3H).

^{13}C NMR (151 MHz, CDCl$_3$): δ 202.0, 173.7, 151.0, 135.6, 134.0, 133.0, 131.0, 130.1, 128.6, 128.1, 128.0, 127.9, 126.6, 126.5, 125.6, 124.4, 119.6, 117.0, 102.8, 52.6, 50.9, 42.7, 40.5, 38.5, 30.8, 30.6, 22.1, 13.6.

IR (neat): 2955, 2224, 1728, 1690, 1244, 1096, 968, 908 cm^{-1}.

HRMS (ESI-TOF) calcd. for $C_{28}H_{29}NNaO_3S$ [M+Na]$^+$: 482.1760, found: 482.1745.

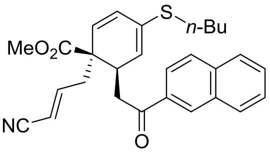

4i

Methyl (1*S*,6*R*)-4-(butylthio)-1-((*E*)-3-cyanoallyl)-6-(2-(naphthalen-2-yl)-2-oxoethyl)cyclohexa-2,4-diene-1-carboxylate (4i):

Following the general procedure, the title compound was obtained as colorless oil, 176.8 mg, 77% yield. (Rf = 0.26, eluent: PE/EtOAc = 5/1).

^{1}H NMR (600 MHz, $CDCl_3$): δ 8.35 (s, 1H), 7.98 – 7.92 (m, 2H), 7.89 – 7.83 (m, 2H), 7.63 – 7.51 (m, 2H), 6.65 – 6.55 (m, 1H), 6.17 (d, *J* = 9.9 Hz, 1H), 6.04 – 5.97 (m, 1H), 5.69 (d, *J* = 6.3 Hz, 1H), 5.33 (d, *J* = 16.2 Hz, 1H), 3.73 (s, 3H), 3.40 – 3.33 (m, 1H), 3.32 – 3.22 (m, 1H), 2.94 – 2.86 (m, 1H), 2.84 – 2.78 (m, 1H), 2.62 – 2.54 (m, 3H), 1.50 – 1.41 (m, 2H), 1.34 – 1.26 (m, 2H), 0.79 (t, *J* = 7.4 Hz, 3H).

^{13}C NMR (151 MHz, $CDCl_3$): δ197.8, 173.8, 151.1, 135.7, 134.1, 132.5, 130.7, 130.1, 129.7, 128.7, 128.6, 127.9, 127.8, 126.9, 126.6, 123.7, 120.0 117.0, 102.8, 52.6, 50.9, 40.5, 39.4, 38.3, 30.8, 30.5, 22.0, 13.6.

IR (neat): 2954, 2928, 2223, 1728, 1677, 1595, 1466, 1182, 1122, 968 cm^{-1}.

HRMS (ESI-TOF) calcd. for $C_{28}H_{29}NNaO_3S$ $[M+Na]^+$: 482.1760, found: 482.1747.

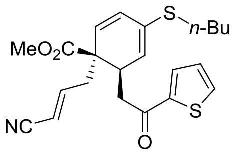

4j

Methyl (1*S*,6*R*)-4-(butylthio)-1-((*E*)-3-cyanoallyl)-6-(2-oxo-2-(thiophen-2-yl)ethyl)cyclohexa-2,4-diene-1-carboxylate (4j):

Following the general procedure, the title compound was obtained as yellow oil, 147.4 mg, 71% yield. (Rf = 0.23, eluent: PE/EtOAc = 5/1).

^{1}H NMR (600 MHz, $CDCl_3$): δ 7.64 – 7.56 (m, 2H), 7.11 – 7.06 (m, 1H), 6.60 – 6.52 (m, 1H), 6.10 (d, *J* = 9.9 Hz, 1H), 5.99 – 5.91 (m, 1H), 5.58 (d, *J* = 6.1 Hz, 1H), 5.30 (d, *J* = 16.2 Hz, 1H), 3.70 (s, 3H), 3.18 – 3.05 (m, 2H), 2.88 – 2.80 (m, 1H), 2.63 – 2.52 (m, 4H), 1.50 – 1.42 (m, 2H), 1.36 – 1.29 (m, 2H), 0.82 (t, *J* = 7.4 Hz, 3H).

^{13}C NMR (151 MHz, $CDCl_3$): δ 190.8, 173.6, 150.9, 144.1, 134.1, 132.4, 130.9, 128.3, 127.8, 126.5, 119.4, 116.9, 102.8, 52.6, 50.8, 40.4, 39.9, 38.3, 30.8, 30.6, 22.1, 13.7.

IR (neat): 2955, 2929, 2224, 1727, 1657, 1414, 1214, 968, 910 cm^{-1}.

HRMS (ESI-TOF) calcd. for $C_{22}H_{25}NNaO_3S_2$ $[M+Na]^+$: 438.1168, found: 438.1187.

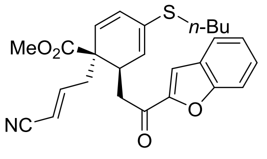

4k

Methyl (1*S*,6*R*)-6-(2-(benzofuran-2-yl)-2-oxoethyl)-4-(butylthio)-1-((*E*)-3-cyanoallyl)cyclohexa-2,4-diene-1-carboxylate (4k):

Following the general procedure, the title compound was obtained as yellow oil, 184.2 mg, 82% yield. (Rf = 0.20, eluent: PE/EtOAc = 5/1).

^{1}H NMR (600 MHz, $CDCl_3$): δ 7.67 (d, *J* = 7.7 Hz, 1H), 7.53 (d, *J* = 8.3 Hz, 1H), 7.48 – 7.41 (m, 2H), 7.31 – 7.26 (m, 1H), 6.61 – 6.52 (m, 1H), 6.14 (d, *J* = 9.9 Hz, 1H), 6.00 – 5.93 (m, 1H), 5.61 (d, *J* = 4.9 Hz, 1H), 5.31 (d, *J* = 16.2 Hz, 1H), 3.73 (s, 3H), 3.24 – 3.13 (m, 2H), 2.90 – 2.80 (m, 1H), 2.72 – 2.64 (m, 1H), 2.60 – 2.51 (m, 3H), 1.49 – 1.40 (m, 2H), 1.33 – 1.25 (m, 2H), 0.79 (t, *J* = 7.3 Hz, 3H).

^{13}C NMR (151 MHz, $CDCl_3$): δ 188.8, 173.5, 155.6, 152.2, 150.9, 131.1, 128.5, 127.8, 126.9, 126.5, 124.0, 123.4, 119.1, 116.9, 113.4, 112.5, 102.8, 52.6, 50.8, 40.4, 39.6, 37.9, 30.7, 30.5, 22.0, 13.6.

IR (neat): 2955, 2224, 1728, 1677, 1555, 1435, 1257, 1137, 968, 910, 752 cm^{-1}.

HRMS (ESI-TOF) calcd. for $C_{26}H_{27}NNaO_4S$ $[M+Na]^+$: 472.1553, found: 472.1553.

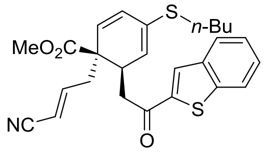

4l

Methyl (1*S*,6*R*)-6-(2-(benzo[b]thiophen-2-yl)-2-oxoethyl)-4-(butylthio)-1-((*E*)-3-cyanoallyl)cyclohexa-2,4-diene-1-carboxylate (4l):

Following the general procedure, the title compound was obtained as white solid, m.p. 109 – 112 ℃, 181.4 mg, 77% yield. (Rf = 0.25, eluent: PE/EtOAc = 5/1).

^{1}H NMR (600 MHz, $CDCl_3$): δ 7.91 – 7.81 (m, 3H), 7.49 – 7.43 (m, 1H), 7.42 – 7.36 (m, 1H), 6.64 – 6.54 (m, 1H), 6.15 (d, *J* = 9.9 Hz, 1H), 5.99 (d, *J* = 9.9 Hz, 1H), 5.60 (d, *J* = 5.8 Hz, 1H), 5.32 (d, *J* = 16.2 Hz, 1H), 3.74 (s, 3H), 3.29 – 3.17 (m, 2H), 2.92 – 2.84 (m, 1H), 2.77 – 2.70 (m, 1H), 2.63 – 2.53 (m, 3H), 1.49 – 1.41 (m, 2H), 1.33 – 1.26 (m, 2H), 0.79 (t, *J* = 7.4 Hz, 3H).

^{13}C NMR (151 MHz, $CDCl_3$): δ 192.3, 173.5, 150.9, 143.4, 142.5, 139.0, 131.1, 129.7, 127.8, 127.6, 126.5, 126.1, 125.1, 123.0, 119.0, 116.9, 102.8, 52.6, 50.8, 40.4, 39.7, 38.3, 30.7, 30.4, 22.0, 13.6.

IR (neat): 3046, 2951, 2923, 2861, 2220, 1733, 1644, 1511, 1438, 1183, 1139, 976 cm^{-1}.

HRMS (ESI-TOF) calcd. for $C_{26}H_{27}NNaO_3S_2$ $[M+Na]^+$: 488.1325, found: 488.1318.

Supplementary Table 2. Crystal data and structure refinement for **4l**

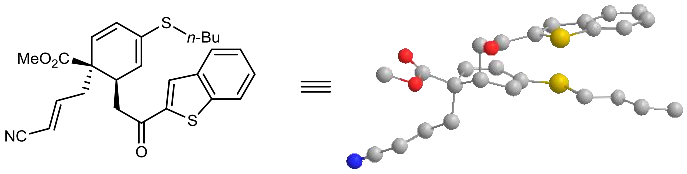

4l

Bond precision	c-c = 0.0034 Å		Wavelength=0.71073
Cell	a=9.3087 (7)	b=9.2669 (6)	c=29.066 (2)
	α=90	β=96.877 (2)	γ=90
Temperature	294 K		
	Calculated	Reported	
Volume	2489.3 (3)	2489.3 (3)	
Space group	P 21/c	P2(1)/c	
Hall group	-P 2ybc		
Moiety formula	C_{26} H_{27} N O_3 S_2	C_{26} H_{27} N O_3 S_2	
Sum formula	C_{26} H_{27} N O_3 S_2	C_{26} H_{27} N O_3 S_2	
Mr	465.61	465.61	
Dx,g cm^{-3}	1.242	1.242	
Z	4	4	
Mu (mm^{-1})	0.241	0.241	
F000	984.0	984.0	
F000'	985.37		
h,k,lmax	12,12,37	12,12,37	
Nref	5673	5642	
Tmin,Tmax	0.949,0.974	0.949,0.974	
Tmin'	0.942		

Correction method= # Reported T Limits: Tmin=0.949 Tmax=0.974

AbsCorr = EMPIRICAL

Data completeness= 0.995 Theta(max)= 27.460

R(reflections)= 0.0495(3790) wR2(reflections)= 0.1294(5642)

S = 1.036 Npar= 289

The single crystal of **4l** was obtained by recrystallization in a solution of DCM/Hexane at 4 ℃.

Crystallographic data (excluding structural factors) for this compound **4l** has been deposited at the

Cambridge Crystallographic Data Centre under the deposition number CCDC 2019506.

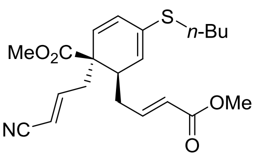

4m

Methyl (1*S*,6*R*)-4-(butylthio)-1-((*E*)-3-cyanoallyl)-6-((*E*)-4-methoxy-4-oxobut-2-en-1-yl)cyclohexa-2,4-diene-1-carboxylate (4m):

Following the general procedure, the title compound was obtained as colorless oil, 153.7 mg, 79% yield. (Rf = 0.28, eluent: PE/EtOAc = 5/1).

^{1}H NMR (600 MHz, CDCl$_3$): δ 6.86 – 6.78 (m, 1H), 6.59 – 6.47 (m, 1H), 6.07 (d, *J* = 9.9 Hz, 1H), 5.97 – 5.90 (m, 1H), 5.74 (d, *J* = 15.5 Hz, 1H), 5.47 (d, *J* = 6.2 Hz, 1H), 5.28 (d, *J* = 16.2 Hz, 1H), 3.77 – 3.67 (m, 6H), 2.81 – 2.75 (m, 1H), 2.72 – 2.62 (m, 2H), 2.57 – 2.44 (m, 2H), 2.32 – 2.24 (m, 1H), 2.04 – 1.97 (m, 1H), 1.60 – 1.53 (m, 2H), 1.44 – 1.36 (m, 2H), 0.90 (t, *J* = 7.4 Hz, 3H).

^{13}C NMR (151 MHz, CDCl$_3$): δ 173.5, 166.6, 150.8, 146.0, 131.2, 128.0, 126.5, 123.1, 119.5, 116.9, 102.9, 52.5, 51.6, 51.1, 41.8, 41.0, 33.4, 31.0, 30.8, 22.1, 13.7.

IR (neat): 2954, 2224, 1719, 1654, 1434, 1270, 1209, 970, 913 cm^{-1}.

HRMS (ESI-TOF) calcd. for $C_{21}H_{27}NNaO_4S$ $[M+Na]^+$: 412.1553, found: 412.1571.

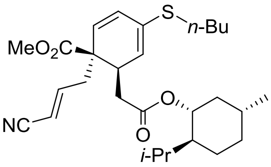

4n
(75/25 dr)

Methyl (1*S*,6*R*)-4-(butylthio)-1-((*E*)-3-cyanoallyl)-6-(2-(((1*R*,2*S*,5*R*)-2-isopropyl-5-methylcyclohexyl)oxy)-2-oxoethyl)cyclohexa-2,4-diene-1-carboxylate (4n):

Following the general procedure, the title compound was obtained as a mixture of two diastereoisomers (75/25 dr), colorless oil, 165.8 mg, 68% yield. (Rf = 0.18, eluent: PE/EtOAc = 10/1).

^{1}H NMR (400 MHz, CDCl$_3$): δ 6.56 (dt, *J* = 15.8, 7.7 Hz, 1H), 6.05 (d, *J* = 9.9 Hz, 1H), 5.99 – 5.90 (m, 1H), 5.65 – 5.55 (m, 1H), 5.30 (d, *J* = 16.2 Hz, 1H), 4.72 – 4.60 (m, 1H), 3.82 – 3.71 (m, 3H), 2.96 – 2.88 (m, 1H), 2.85 – 2.77 (m, 1H), 2.71 – 2.62 (m, 2H), 2.57 – 2.49 (m, 1H), 2.44 –

2.34 (m, 1H), 2.13 – 2.05 (m, 1H), 1.97 – 1.90 (m, 1H), 1.83 – 1.72 (m, 1H), 1.68 – 1.54 (m, 4H), 1.48 – 1.28 (m, 4H), 0.96 – 0.82 (m, 12H), 0.74 (d, J = 7.0 Hz, 3H).

^{13}C NMR (101 MHz, CDCl$_3$): δ 173.5, 171.4, 171.2, 151.0, 131.4, 127.8, 126.5, 119.2, 118.9, 117.0, 102.9, 74.8, 74.7, 52.6, 52.4, 50.79, 50.74, 47.05, 46.99, 45.3, 41.07, 41.03, 40.6, 38.9, 38.7, 35.45, 34.3, 31.53, 31.50, 31.0, 30.8, 30.7, 26.5, 26.4, 23.6, 23.5, 22.8, 22.21, 22.15, 20.9, 20.8, 16.5, 16.4, 13.8.

IR (neat): 2955, 2871, 2361, 2225, 1729, 1455, 1370, 1249, 1219, 1177, 1057, 982 cm^{-1}.

HRMS (ESI-TOF) calcd. for $C_{28}H_{41}NNaO_4S$ $[M+Na]^+$: 510.2649, found: 510.2657.

Diastereoisomer ratio (75/25 dr) is determined by HPLC (Chirapak AD-H, hexane/*i*-PrOH = 90/10, flow rate = 1.0 mL/min, 254 nm): major isomer: t_R = 8.13 min, minor isomer: t_R = 13.59 min.

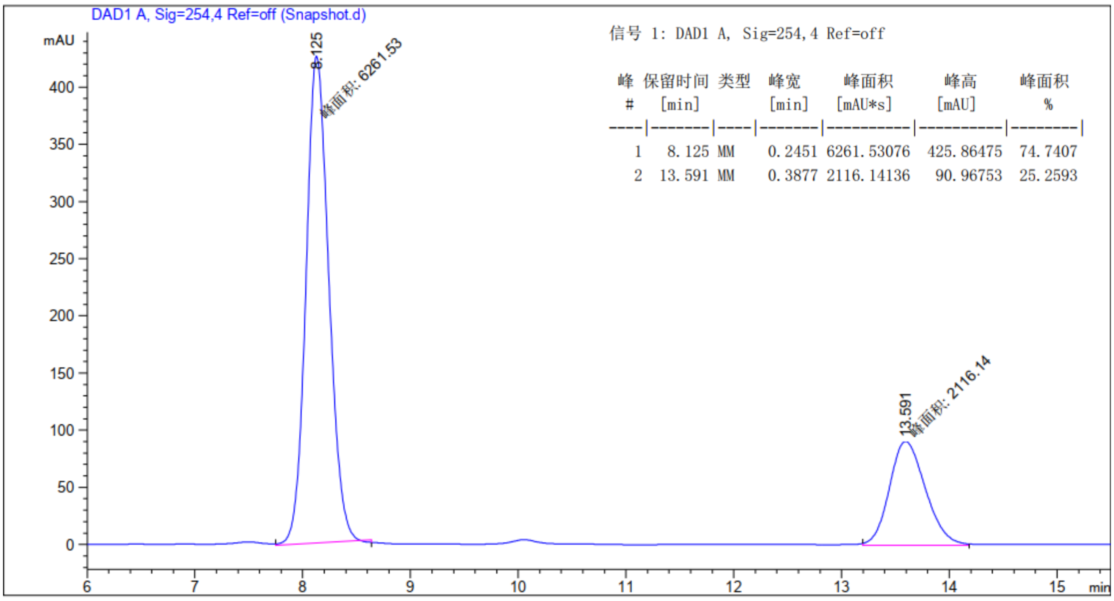

Supplementary Figure 4. HPLC Spectrum of **4n**

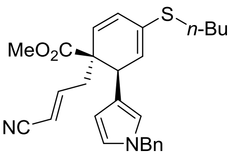

4o

Methyl (1*S*,6*S*)-6-(1-benzyl-1*H*-pyrrol-3-yl)-4-(butylthio)-1-((*E*)-3-cyanoallyl)cyclohexa-2,4-diene-1-carboxylate (4o):

Following the general procedure, the title compound was obtained as yellow oil, 185.2 mg, 83% yield. (Rf = 0.31, eluent: PE/EtOAc = 5/1).

^{1}H NMR (600 MHz, CDCl$_3$): δ 7.35 – 7.30 (m, 2H), 7.29 – 7.25 (m, 1H), 6.95 (d, *J* = 7.3 Hz, 2H), 6.63 – 6.56 (m, 2H), 6.15 – 6.12 (m, 1H), 6.09 (d, *J* = 10.0 Hz, 1H), 6.03 – 5.97 (m, 2H), 5.29 (d, *J* = 16.2 Hz, 1H), 5.16 (d, *J* = 16.5 Hz, 1H), 5.04 (d, *J* = 16.5 Hz, 1H), 4.99 (d, *J* = 6.3 Hz, 1H), 3.48 (d, *J* = 6.3 Hz, 1H), 3.45 (s, 3H), 2.81 – 2.75 (m, 1H), 2.61 – 2.55 (m, 1H), 2.54 – 2.49 (m, 2H), 1.53 – 1.46 (m, 2H), 1.40 – 1.33 (m, 2H), 0.89 (t, *J* = 7.4 Hz, 3H).

^{13}C NMR (151 MHz, CDCl$_3$): δ 173.4, 150.7, 138.9, 128.84, 128.76, 128.74, 127.6, 126.3, 126.2, 122.2, 119.5, 117.0, 108.7, 107.6, 102.9, 52.4, 51.7, 50.7, 42.4, 40.3, 30.9, 30.8, 22.1, 13.7.

IR (neat): 2954, 2928, 2870, 2223, 1727, 1681, 1435, 1202, 968 cm^{-1}.

HRMS (ESI-TOF) calcd. for $C_{27}H_{30}N_2NaO_2S$ $[M+Na]^+$: 469.1920, found: 469.1907.

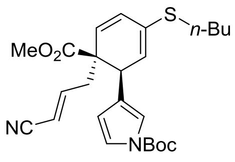

4p

***tert*-Butyl 3-((1*S*,6*S*)-3-(butylthio)-6-((*E*)-3-cyanoallyl)-6-(methoxycarbonyl)cyclohexa-2,4-dien-1-yl)-1*H*-pyrrole-1-carboxylate (4p):**

Following the general procedure, the title compound was obtained as yellow oil, 114.1 mg, 50% yield. (Rf = 0.40, eluent: PE/EtOAc = 5/1).

^{1}H NMR (600 MHz, CDCl$_3$): δ 7.12 – 7.08 (m, 1H), 6.67 – 6.59 (m, 1H), 6.16 (d, *J* = 10.0 Hz, 1H), 6.12 – 6.10 (m, 1H), 6.00 – 5.96 (m, 1H), 5.94 (t, *J* = 3.4 Hz, 1H), 5.77 – 5.73 (m, 1H), 5.35 – 5.30 (m, 1H), 4.72 (d, *J* = 6.4 Hz, 1H), 3.49 (s, 3H), 2.92 – 2.86 (m, 1H), 2.70 – 2.60 (m, 3H), 1.61 (s, 9H), 1.57 – 1.53 (m, 2H), 1.42 – 1.38 (m, 2H), 0.89 (t, *J* = 7.4 Hz, 3H).

^{13}C NMR (151 MHz, CDCl$_3$): δ 173.5, 151.0, 149.5, 132.5, 129.5, 127.9, 126.3, 121.6, 119.6, 117.1, 112.1, 110.2, 102.7, 83.9, 52.4, 51.4, 42.2, 40.1, 31.1, 30.9, 28.2, 22.1, 13.8.

IR (neat): 2956, 2930, 2872, 2224, 1731, 1319, 1118, 1066, 969, 912 cm^{-1}.

HRMS (ESI-TOF) calcd. for $C_{25}H_{32}N_2NaO_4S$ $[M+Na]^+$: 479.1975, found: 479.1970.

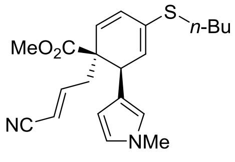

4q

Methyl (1*S*,6*S*)-4-(butylthio)-1-((*E*)-3-cyanoallyl)-6-(1-methyl-1*H*-pyrrol-3-yl)cyclohexa-2,4-diene-1-carboxylate (4q):

Following the general procedure, the title compound was obtained as

yellow oil, 157.3 mg, 85% yield. (Rf = 0.26, eluent: PE/EtOAc = 5/1).

^{1}H NMR (600 MHz, CDCl$_3$): δ 6.67 – 6.59 (m, 1H), 6.47 – 6.40 (m, 1H), 6.08 – 6.00 (m, 3H), 5.96 – 5.91 (m, 1H), 5.52 (d, *J* = 6.3 Hz, 1H), 5.36 (d, *J* = 16.2 Hz, 1H), 3.65 (d, *J* = 6.3 Hz, 1H), 3.54 (s, 3H), 3.40 (s, 3H), 2.93 – 2.86 (m, 1H), 2.74 – 2.66 (m, 3H), 1.61 – 1.55 (m, 2H), 1.44 – 1.37 (m, 2H), 0.91 (t, *J* = 7.4 Hz, 3H).

^{13}C NMR (151 MHz, CDCl$_3$): δ 173.4, 150.8, 129.2, 128.6, 127.5, 126.2, 122.5, 119.4, 117.0, 108.4, 107.2, 103.0, 52.4, 51.8, 42.5, 40.8, 33.9, 31.1, 30.9, 22.1, 13.8.

IR (neat): 2954, 2871, 2222, 1725, 1434, 1206, 1089, 969, 912 cm^{-1}.

HRMS (ESI-TOF) calcd. for $C_{21}H_{26}N_2NaO_2S$ [M+Na]$^+$: 393.1609, found: 393.1611.

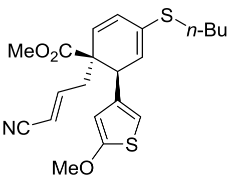

4r

Methyl (1*S*,6*R*)-4-(butylthio)-1-((*E*)-3-cyanoallyl)-6-(5-methoxythiophen-3-yl)cyclohexa-2,4-diene-1-carboxylate (4r):

Following the general procedure, the title compound was obtained as yellow oil, 165.3 mg, 82% yield. (Rf = 0.30, eluent: PE/EtOAc = 5/1).

^{1}H NMR (600 MHz, CDCl$_3$): δ 6.63 – 6.55 (m, 1H), 6.38 (d, *J* = 3.9 Hz, 1H), 6.04 (d, *J* = 0.7 Hz, 2H), 5.91 (d, *J* = 3.9 Hz, 1H), 5.56 (d, *J* = 6.4 Hz, 1H), 5.37 – 5.30 (m, 1H), 3.78 (s, 3H), 3.74 (d, *J* = 6.3 Hz, 1H), 3.55 (s, 3H), 2.91 – 2.85 (m, 1H), 2.77 – 2.65 (m, 3H), 1.64 – 1.58 (m, 2H), 1.46 – 1.37 (m, 2H), 0.91 (t, *J* = 7.4 Hz, 3H).

^{13}C NMR (151 MHz, CDCl$_3$): δ 172.8, 165.7, 150.9, 130.4, 127.8, 126.4, 125.9, 124.0, 119.5, 117.0, 102.9, 102.8, 60.0, 53.0, 52.3, 45.3, 41.5, 30.89, 30.82, 22.1, 13.8.

IR (neat): 2954, 2926, 2223, 1728, 1498, 1431, 1202, 969, 910 cm^{-1}.

HRMS (ESI-TOF) calcd. for $C_{21}H_{25}NNaO_3S_2$ [M+Na]$^+$: 426.1168, found: 426.1166.

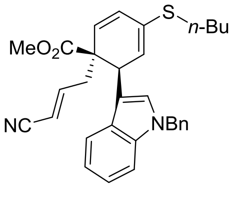

4s

Methyl (1*S*,6*S*)-6-(1-benzyl-1*H*-indol-3-yl)-4-(butylthio)-1-((*E*)-3-cyanoallyl)cyclohexa-2,4-diene-1-carboxylate (4s):

Following the general procedure, the title compound was obtained as yellow oil, 183.6 mg, 74% yield. (Rf = 0.35, eluent: PE/EtOAc = 5/1).

¹H NMR (600 MHz, CDCl$_3$): δ 7.60 – 7.55 (m, 1H), 7.28 – 7.23 (m, 3H), 7.18 – 7.14 (m, 1H), 7.12 – 7.08 (m, 2H), 7.04 (s, 1H), 7.01 – 6.96 (m, 2H), 6.72 – 6.64 (m, 1H), 6.14 – 6.10 (m, 1H), 6.06 (d, *J* = 10.0 Hz, 1H), 5.72 (d, *J* = 6.2 Hz, 1H), 5.39 (d, *J* = 16.2 Hz, 1H), 5.24 (d, *J* = 3.1 Hz, 2H), 4.05 (d, *J* = 6.2 Hz, 1H), 3.08 (s, 3H), 3.03 – 2.97 (m, 1H), 2.88 – 2.82 (m, 1H), 2.76 – 2.67 (m, 2H), 1.61 – 1.55 (m, 2H), 1.45 – 1.37 (m, 2H), 0.89 (t, *J* = 7.4 Hz, 3H).

¹³C NMR (151 MHz, CDCl$_3$): δ 173.4, 151.4, 137.6, 136.2, 128.8, 128.3, 128.2, 127.8, 127.6, 127.1, 126.51, 126.47, 121,79, 121.77, 119.41, 119.38, 117.2, 110.9, 109.9, 102.8, 52.3, 52.0, 50.2, 42.1, 40.8, 31.08, 31.00, 22.1, 13.8.

IR (neat): 2953, 2222, 1726, 1685, 1465, 1453, 1335, 1200, 1176, 968, 908 cm^{-1}.

HRMS (ESI-TOF) calcd. for $C_{31}H_{32}N_2NaO_2S$ $[M+Na]^+$: 519.2077, found: 519.2067.

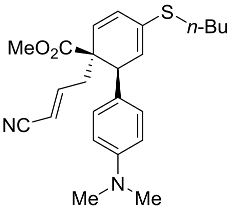

4t

Methyl (1*S*,2*S*)-5-(butylthio)-2-((*E*)-3-cyanoallyl)-4'-(dimethylamino)-1,2-dihydro-[1,1'-biphenyl]-2-carboxylate (4t):

Following the general procedure, the title compound was obtained as yellow oil, 160.0 mg, 78% yield. (Rf = 0.26, eluent: PE/EtOAc = 5/1).

¹H NMR (600 MHz, CDCl$_3$): δ 7.03 – 6.97 (m, 2H), 6.68 – 6.60 (m, 1H), 6.60 – 6.54 (m, 2H), 6.09 – 6.04 (m, 1H), 5.96 (d, *J* = 9.9 Hz, 1H), 5.56 (d, *J* = 6.1 Hz, 1H), 5.36 (d, *J* = 16.2 Hz, 1H), 3.48 (d, *J* = 6.2 Hz, 1H), 3.35 (s, 3H), 2.95 – 2.86 (m, 7H), 2.78 – 2.68 (m, 3H), 1.63 – 1.57 (m, 2H), 1.47 – 1.38 (m, 2H), 0.91 (t, *J* = 7.4 Hz, 3H).

¹³C NMR (151 MHz, CDCl$_3$): δ 173.2, 151.3, 150.1, 129.6, 129.2, 127.7, 126.5, 124.5, 121.1, 117.2, 112.2, 102.7, 52.8, 52.0, 49.4, 42.7, 40.6, 31.1, 31.0, 22.2, 13.8.

IR (neat): 2952, 2222, 1728, 1609, 1518, 1195, 969, 911, 817 cm^{-1}.

HRMS (ESI-TOF) calcd. for $C_{24}H_{30}N_2NaO_2S$ $[M+Na]^+$: 433.1920, found: 433.1916.

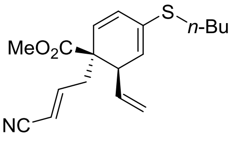

4u

Methyl (1*S*,6*R*)-4-(butylthio)-1-((*E*)-3-cyanoallyl)-6-vinylcyclohexa-2,4-diene-1-carboxylate (4u):

Following the general procedure, vinylzinc chloride (**3u**, 0.35 M in THF,

4.3 mL, 3.0 equiv) was used as nucleophile. The title compound was obtained as colorless oil, 122.1 mg, 77% yield. (Rf = 0.48, eluent: PE/EtOAc = 5/1).

^{1}H NMR (600 MHz, CDCl$_3$): δ 6.60 – 6.52 (m, 1H), 6.00 (d, *J* = 10.0 Hz, 1H), 5.95 – 5.90 (m, 1H), 5.64 – 5.55 (m, 1H), 5.40 – 5.36 (m, 1H), 5.33 – 5.27 (m, 1H), 5.06 – 4.99 (m, 1H), 4.97 – 4.92 (m, 1H), 3.65 (s, 3H), 2.96 – 2.90 (m, 1H), 2.82 – 2.76 (m, 1H), 2.73 – 2.64 (m, 2H), 2.56 – 2.51 (m, 1H), 1.60 – 1.55 (m, 2H), 1.44 – 1.38 (m, 2H), 0.90 (t, *J* = 7.4 Hz, 3H).

^{13}C NMR (151 MHz, CDCl$_3$): δ 173.2, 151.0, 134.0, 130.4, 127.7, 126.3, 118.7, 117.5, 117.0, 102.8, 52.2, 51.3, 48.4, 40.5, 31.0, 30.8, 22.1, 13.8.

IR (neat): 2955, 2224, 1728, 1434, 1249, 1211, 1101, 970, 911 cm^{-1}.

HRMS (ESI-TOF) calcd. for $C_{18}H_{23}NNaO_2S$ [M+Na]$^+$: 340.1342, found: 340.1347.

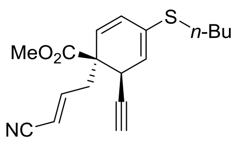

4v

Methyl (1*S*,6*S*)-4-(butylthio)-1-((*E*)-3-cyanoallyl)-6-ethynylcyclohexa-2,4-diene-1-carboxylate (4v):

Following the general procedure, ethynylzinc chloride (**3v**, 0.22 M in THF, 6.8 mL, 3 equiv) was used as nucleophile. The title compound was obtained as yellow oil, 107.1 mg, 68% yield. (Rf = 0.19, eluent: PE/EtOAc = 10/1).

^{1}H NMR (600 MHz, CDCl$_3$): δ 6.60 – 6.52 (m, 1H), 6.11 (d, *J* = 10.0 Hz, 1H), 6.02 – 5.97 (m, 1H), 5.46 – 5.42 (m, 1H), 5.36 – 5.30 (m, 1H), 3.77 (s, 3H), 3.42 – 3.37 (m, 1H), 2.77 – 2.68 (m, 3H), 2.56 – 2.49 (m, 1H), 2.11 (d, *J* = 2.5 Hz, 1H), 1.63 – 1.58 (m, 2H), 1.46 – 1.41 (m, 2H), 0.92 (t, *J* = 7.4 Hz, 3H).

^{13}C NMR (151 MHz, CDCl$_3$): δ 172.4, 150.2, 132.4, 127.7, 126.7, 116.8, 114.5, 103.3, 80.3, 71.1, 52.7, 51.0, 34.0, 34.7, 31.0, 30.7, 22.2, 13.8.

IR (neat): 3293, 2956, 2872, 2225, 1729, 1434, 1212, 910 cm^{-1}.

HRMS (ESI-TOF) calcd. for $C_{18}H_{21}NNaO_2S$ [M+Na]$^+$: 338.1185, found: 338.1186.

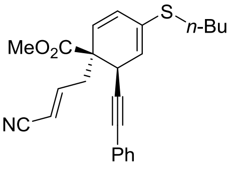

4w

Methyl (1*S*,6*S*)-4-(butylthio)-1-((*E*)-3-cyanoallyl)-6-(phenylethynyl)cyclohexa-2,4-diene-1-carboxylate (4w):

Following the general procedure, (phenylethynyl)zinc chloride (**3w**, 0.32 M in THF, 4.7 mL, 3 equiv) was used as nucleophile. The title compound was obtained as colorless oil, 125.2 mg, 64% yield. (Rf = 0.40, eluent: PE/EtOAc = 5/1).

^{1}H NMR (600 MHz, $CDCl_3$): δ 7.32 – 7.29 (m, 2H), 7.27 – 7.21 (m, 3H), 6.63 – 6.55 (m, 1H), 6.12 (d, *J* = 10.0 Hz, 1H), 6.03 – 5.98 (m, 1H), 5.52 (d, *J* = 6.0 Hz, 1H), 5.35 (d, *J* = 16.2 Hz, 1H), 3.77 (s, 3H), 3.60 (d, *J* = 6.0 Hz, 1H), 2.80 – 2.70 (m, 3H), 2.61 – 2.54 (m, 1H), 1.64 – 1.59 (m, 2H), 1.47 – 1.40 (m, 2H), 0.92 (t, *J* = 7.4 Hz, 3H).

^{13}C NMR (151 MHz, $CDCl_3$): δ 172.5, 150.4, 131.9, 131.7, 128.21, 128.15, 127.7, 126.5, 122.9, 116.8, 115.1, 103.1, 85.4, 83.2, 52.6, 51.2, 40.0, 35.6, 30.9, 30.7, 22.1, 13.7.

IR (neat): 2954, 2929, 2871, 2224, 1730, 1633, 1597, 1489, 1434, 1202, 966, 910, 756 cm^{-1}.

HRMS (ESI-TOF) calcd. for $C_{24}H_{25}NNaO_2S$ $[M+Na]^+$: 414.1489, found: 414.1500.

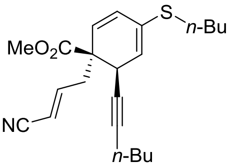

4x

Methyl (1*S*,6*S*)-4-(butylthio)-1-((*E*)-3-cyanoallyl)-6-(hex-1-yn-1-yl)cyclohexa-2,4-diene-1-carboxylate (4x):

Following the general procedure, hex-1-yn-1-yl(oxo)(pivaloyl)zinc (**3x**, 0.45 M in THF, 3.3 mL, 3 equiv) was used as nucleophile. The title compound was obtained as yellow oil, 137.3 mg, 74% yield. (Rf = 0.5, eluent: PE/EtOAc = 5/1).

^{1}H NMR (600 MHz, $CDCl_3$): δ 6.59 – 6.52 (m, 1H), 6.06 (d, *J* = 10.0 Hz, 1H), 5.99 – 5.94 (m, 1H), 5.46 (d, *J* = 6.0 Hz, 1H), 5.31 (d, *J* = 16.2 Hz, 1H), 3.74 (s, 3H), 3.38 – 3.32 (m, 1H), 2.75 – 2.66 (m, 3H), 2.53 – 2.47 (m, 1H), 2.10 – 2.05 (m, 2H), 1.62 – 1.56 (m, 2H), 1.44 – 1.29 (m, 6H), 0.91 (t, *J* = 7.4 Hz, 3H), 0.86 (t, *J* = 7.2 Hz, 3H).

^{13}C NMR (151 MHz, $CDCl_3$): δ 172.7, 150.7, 131.0, 127.7, 126.5, 116.9, 116.8, 102.9, 83.7, 75.7, 52.5, 51.2, 40.2, 35.2, 31.02, 30.95, 30.8, 22.1, 21.9, 18.5, 13.8, 13.7.

IR (neat): 3467, 2956, 2930, 2872, 2224, 1730, 1693, 1209, 968, 911 cm^{-1}.

HRMS (ESI-TOF) calcd. for $C_{22}H_{29}NNaO_2S$ $[M+Na]^+$: 394.1811, found: 394.1817.

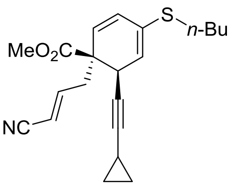

4y

Methyl (1*S*,6*S*)-4-(butylthio)-1-((*E*)-3-cyanoallyl)-6-(cyclopropylethynyl)cyclohexa-2,4-diene-1-carboxylate (4y):

Following the general procedure, (cyclopropylethynyl)(oxo)(pivaloyl)zinc (**3y**, 0.42 M in THF, 3.6 mL, 3 equiv) was used as nucleophile. The title compound was obtained as yellow oil, 135.0 mg, 76% yield. (Rf = 0.33, eluent: PE/EtOAc = 5/1).

^{1}H NMR (600 MHz, CDCl$_3$): δ 6.59 – 6.51 (m, 1H), 6.03 (d, *J* = 10.0 Hz, 1H), 5.98 – 5.93 (m, 1H), 5.42 (d, *J* = 6.0 Hz, 1H), 5.30 (d, *J* = 16.2 Hz, 1H), 3.74 (s, 3H), 3.30 (d, *J* = 4.9 Hz, 1H), 2.75 – 2.66 (m, 3H), 2.53 – 2.46 (m, 1H), 1.63 – 1.54 (m, 2H), 1.45 – 1.39 (m, 2H), 1.17 – 1.06 (m, 1H), 0.92 (t, *J* = 7.4 Hz, 3H), 0.72 – 0.64 (m, 2H), 0.58 – 0.47 (m, 2H).

^{13}C NMR (151 MHz, CDCl$_3$): δ 172.7, 150.7, 131.1, 127.6, 126.5, 116.9, 116.3, 103.0, 86.8, 70.9, 52.5, 51.3, 40.1, 35.2, 31.0, 30.8, 22.1, 13.8, 8.5, 8.4, -0.4.

IR (neat): 3462, 2955, 2930, 2872, 2224, 1434, 1203, 968, 912 cm^{-1}.

HRMS (ESI-TOF) calcd. for $C_{21}H_{25}NNaO_2S$ $[M+Na]^+$: 378.1498, found: 378.1496.

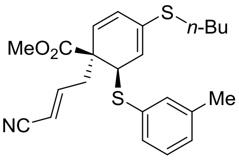

4z

Methyl (1*R*,6*R*)-4-(butylthio)-1-((*E*)-3-cyanoallyl)-6-(m-tolylthio)cyclohexa-2,4-diene-1-carboxylate (4z):

Following the general procedure, the title compound was obtained as yellow oil, 103.3 mg, 50% yield. (Rf = 0.40, eluent: PE/EtOAc = 5/1).

^{1}H NMR (600 MHz, CDCl$_3$): δ 7.21 – 7.17 (m, 2H), 7.15 – 7.10 (m, 1H), 7.07 (d, *J* = 7.6 Hz, 1H), 6.55 – 6.47 (m, 1H), 6.08 (d, *J* = 9.9 Hz, 1H), 5.74 – 5.69 (m, 1H), 5.52 (d, *J* = 6.4 Hz, 1H), 5.27 (d, *J* = 16.2 Hz, 1H), 3.91 – 3.87 (m, 1H), 3.68 (s, 3H), 2.73 – 2.69 (m, 1H), 2.68 – 2.62 (m, 2H), 2.58 – 2.52 (m, 1H), 2.31 (s, 3H), 1.60 – 1.54 (m, 2H), 1.44 – 1.39 (m, 2H), 0.92 (t, *J* = 7.4 Hz, 3H).

^{13}C NMR (151 MHz, CDCl$_3$): δ 172.1, 150.4, 138.3, 135.6, 132.7, 132.3, 131.9, 129.1, 128.4, 128.0, 126.3, 116.8, 115.9, 103.0, 52.8, 52.4, 52.2, 42.0, 30.8, 30.7, 22.2, 21.3, 13.7.

IR (neat): 2954, 2871, 2223, 1730, 1591, 1434, 1204, 1080, 966, 910, 780 cm^{-1}.

HRMS (ESI-TOF) calcd. for $C_{23}H_{27}NNaO_2S_2$ [M+Na]$^+$: 436.1375, found: 436.1382.

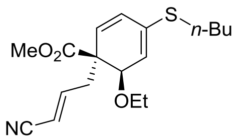

4a'

Methyl (1*S*,6*R*)-4-(butylthio)-1-((*E*)-3-cyanoallyl)-6-ethoxycyclohexa-2,4-diene-1-carboxylate (4a'):

Following a procedure similar to the general procedure, a solution of KOH (3.5 M in EtOH, 0.5 mL) was used as nucleophile. The title compound was obtained as colorless oil, 125.7 mg, 75% yield. (Rf = 0.32, eluent: PE/EtOAc = 5/1).

^{1}H NMR (600 MHz, CDCl$_3$): δ 6.58 – 6.48 (m, 1H), 6.18 (d, *J* = 9.9 Hz, 1H), 5.99 – 5.93 (m, 1H), 5.58 (d, *J* = 5.7 Hz, 1H), 5.31 (d, *J* = 16.2 Hz, 1H), 3.85 – 3.82 (m, 1H), 3.75 (s, 3H), 3.59 – 3.53 (m, 1H), 3.39 – 3.33 (m, 1H), 2.79 – 2.70 (m, 2H), 2.58 – 2.53 (m, 1H), 2.52 – 2.46 (m, 1H), 1.65 – 1.59 (m, 2H), 1.47 – 1.40 (m, 2H), 1.06 (t, *J* = 7.0 Hz, 3H), 0.92 (t, *J* = 7.4 Hz, 3H).

^{13}C NMR (151 MHz, CDCl$_3$): δ 172.7, 150.5, 136.2, 129.1, 125.4, 116.9, 112.3, 102.9, 75.9, 64.4, 52.5, 52.5, 39.9, 30.6, 30.4, 22.2, 15.6, 13.8.

IR (neat): 2957, 2931, 2224, 1732, 1633, 1595, 1436, 1276, 1111, 1031, 972, 762 cm^{-1}.

HRMS (ESI-TOF) calcd. for $C_{18}H_{25}NNaO_3S$ [M+Na]$^+$: 358.1447, found: 358.1452.

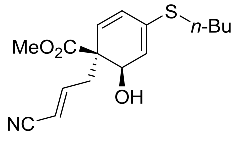

4b'

Methyl (1*S*,6*R*)-4-(butylthio)-1-((*E*)-3-cyanoallyl)-6-hydroxycyclohexa-2,4-diene-1-carboxylate (4b'):

Following a procedure similar to the general procedure, 4-ethylmorpholine (251 μL, 4.0 equiv) was used as base in the reaction, a solution of H_2O (1.0 M in THF, 3.0 mL) was used as nucleophile. The title compound was obtained as colorless oil, 116.7 mg, 76% yield. (Rf = 0.33, eluent: PE/EtOAc = 2/1).

^{1}H NMR (600 MHz, CDCl$_3$): δ 6.63 – 6.50 (m, 1H), 6.11 (d, *J* = 9.9 Hz, 1H), 6.01 – 5.92 (m, 1H), 5.56 (d, *J* = 5.8 Hz, 1H), 5.38 – 5.28 (m, 1H), 4.18 (d, *J* = 5.6 Hz, 1H), 3.77 (s, 3H), 2.78 – 2.71 (m, 2H), 2.61 – 2.47 (m, 2H), 1.66 – 1.59 (m, 2H), 1.47 – 1.40 (m, 2H), 0.92 (t, *J* = 7.4 Hz, 3H).

^{13}C NMR (151 MHz, CDCl$_3$): δ 172.9, 150.3, 135.3, 128.6, 125.8, 116.8, 114.9, 103.1, 69.7, 53.4, 52.7, 39.6, 30.6, 30.3, 22.2, 13.7.

IR (neat): 3467, 2955, 2871, 2223, 1729, 1435, 1201, 1040, 968, 753 cm^{-1}.

HRMS (ESI-TOF) calcd. for $C_{16}H_{21}NNaO_3S$ [M+Na]$^+$: 330.1134, found: 330.1142.

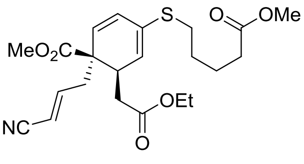

6a

Methyl (1*S*,6*R*)-1-((*E*)-3-cyanoallyl)-6-(2-ethoxy-2-oxoethyl)-4-((5-methoxy-5-oxopentyl)thio)cyclohexa-2,4-diene-1-carboxylate (6a): Following a procedure similar to the general procedure, the reaction of **1b** with **2a** and **3a** was conducted under modified conditions (Tf$_2$O, -55 ℃, 36 h), **6a** were obtained as yellow oil, 104.4 mg, 48% yield. (Rf = 0.24, eluent: PE/EtOAc = 5/1).

^{1}H NMR (600 MHz, CDCl$_3$): δ 6.59 – 6.52 (m, 1H), 6.06 (d, *J* = 9.9 Hz, 1H), 5.96 – 5.91 (m, 1H), 5.64 (d, *J* = 5.9 Hz, 1H), 5.32 (d, *J* = 16.2 Hz, 1H), 4.14 – 4.07 (m, 2H), 3.74 (s, 3H), 3.66 (s, 3H), 2.95 – 2.89 (m, 1H), 2.84 – 2.78 (m, 1H), 2.74 – 2.65 (m, 2H), 2.55 –2.49 (m, 1H), 2.43 – 2.37 (m, 1H), 2.33 (t, *J* = 7.3 Hz, 2H), 2.16 – 2.10 (m, 1H), 1.76 – 1.70 (m, 2H), 1.67 – 1.62 (m, 2H), 1.26 – 1.22 (m, 3H).

^{13}C NMR (151 MHz, CDCl$_3$): δ 173.7, 173.4, 171.6, 150.9, 130.8, 128.0, 126.4, 120.0, 117.0, 103.0, 60.8, 52.6, 51.7, 50.7, 40.6, 38.6, 35.1, 33.6, 30.9, 28.1, 24.2, 14.3.

IR (neat): 2952, 2225, 1726, 1436, 1209, 1175, 1026, 970, 909 cm^{-1}.

HRMS (ESI-TOF) calcd. for $C_{22}H_{29}NNaO_6S$ [M+Na]$^+$: 458.1608, found: 458.1612.

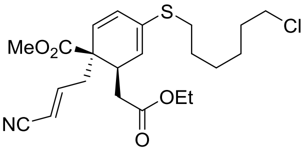

6b

Methyl (1*S*,6*R*)-4-((6-chlorohexyl)thio)-1-((*E*)-3-cyanoallyl)-6-(2-ethoxy-2-oxoethyl)cyclohexa-2,4-diene-1-carboxylate (6b): Following the general procedure, **6b** were obtained from the reaction

of **1c** with **2a** and **3a**. The title compound was obtained as colorless oil, 123.0 mg, 56% yield. (Rf = 0.25, eluent: PE/EtOAc = 5/1).

^{1}H NMR (600 MHz, $CDCl_3$): δ 6.58 – 6.50 (m, 1H), 6.05 (d, *J* = 9.9 Hz, 1H), 5.95 – 5.88 (m, 1H), 5.61 (d, *J* = 6.3 Hz, 1H), 5.30 (d, *J* = 16.2 Hz, 1H), 4.13 – 4.06 (m, 2H), 3.73 (s, 3H), 3.52 (t, *J* = 6.6 Hz, 2H), 2.94 – 2.87 (m, 1H), 2.83 – 2.77 (m, 1H), 2.72 – 2.63 (m, 2H), 2.54 – 2.48 (m, 1H), 2.42 – 2.36 (m, 1H), 2.15 – 2.09 (m, 1H), 1.78 – 1.73 (m, 2H), 1.63 – 1.57 (m, 2H), 1.46 – 1.39 (m, 4H), 1.22 (t, *J* = 7.1 Hz, 3H).

^{13}C NMR (151 MHz, $CDCl_3$): δ 173.4, 171.6, 150.9, 131.0, 127.9, 126.4, 119.5, 116.9, 102.9, 60.8, 52.6, 50.7, 45.0, 40.6, 38.6, 35.1, 32.5, 31.1, 28.4, 28.2, 26.5, 14.3.

IR (neat): 2933, 2857, 2224, 1726, 1434, 1213, 1177, 1025, 969 cm^{-1}.

HRMS (ESI-TOF) calcd. for $C_{22}H_{30}ClNNaO_4S$ $[M+Na]^+$: 462.1476, found: 462.1469.

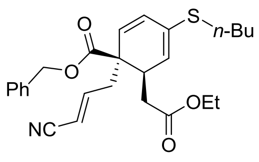

6c

Benzyl (1*S*,6*R*)-4-(butylthio)-1-((*E*)-3-cyanoallyl)-6-(2-ethoxy-2-oxoethyl)cyclohexa-2,4-diene-1-carboxylate (6c):

Following the general procedure, **6c** were obtained from the reaction of **1d** with **2a** and **3a**. The title compound was obtained as colorless oil, 124.6 mg, 55% yield. (Rf = 0.50, eluent: PE/EtOAc = 5/1).

^{1}H NMR (600 MHz, $CDCl_3$): δ 7.41 – 7.34 (m, 5H), 6.47 – 6.39 (m, 1H), 6.07 (d, *J* = 9.9 Hz, 1H), 5.96 – 5.90 (m, 1H), 5.65 – 5.59 (m, 1H), 5.21 (d, *J* = 12.0 Hz, 1H), 5.13 (d, *J* = 12.0 Hz, 1H), 5.07 (d, *J* = 16.2 Hz, 1H), 4.11 – 4.05 (m, 2H), 2.96 – 2.90 (m, 1H), 2.82 – 2.77 (m, 1H), 2.70 – 2.62 (m, 2H), 2.50 – 2.44 (m, 1H), 2.39 – 2.33 (m, 1H), 2.09 – 2.02 (m, 1H), 1.59 – 1.53 (m, 2H), 1.43 – 1.37 (m, 2H), 1.22 (t, *J* = 7.1 Hz, 3H), 0.90 (t, *J* = 7.4 Hz, 3H).

^{13}C NMR (151 MHz, $CDCl_3$): δ 172.7, 171.6, 150.6, 135.2, 131.2, 128.94, 128.92, 128.91, 127.8, 126.6, 119.4, 116.9, 102.9, 67.3, 60.7, 50.5, 40.5, 38.6, 35.0, 30.9, 30.7, 22.1, 14.3, 13.7.

IR (neat): 2958, 2929, 2224, 1726, 1372, 1176, 1096, 967, 910, 697 cm^{-1}.

HRMS (ESI-TOF) calcd. for $C_{26}H_{31}NNaO_4S$ $[M+Na]^+$: 476.1866, found: 476.1869.

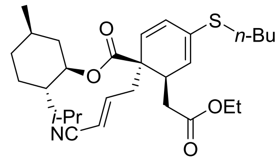

6d
(1/1 dr)

(1*R*,2*S*,5*R*)-2-isopropyl-5-methylcyclohexyl (1*S*,6*R*)-4-(butylthio)-1-((*E*)-3-cyanoallyl)-6-(2-ethoxy-2-oxoethyl)cyclohexa-2,4-diene-1-carboxylate (6d):

Following the general procedure, **6d** were obtained from the reaction of **1e** with **2a** and **3a**. The title compound was obtained as a mixture of two diastereoisomers (1/1 dr), colorless oil, 150.4 mg, 60% yield. (Rf = 0.32, eluent: PE/EtOAc = 10/1).

^{1}H NMR (600 MHz, CDCl$_3$): δ 6.56 – 6.48 (m, 1H), 6.12 – 6.04 (m, 1H), 5.94 – 5.89 (m, 1H), 5.67 – 5.60 (m, 1H), 5.34 – 5.24 (m, 1H), 4.74 – 4.65 (m, 1H), 4.16 – 4.04 (m, 2H), 2.95 – 2.87 (m, 1H), 2.87 – 2.78 (m, 1H), 2.71 – 2.63 (m, 2H), 2.54 – 2.46 (m, 1H), 2.45 – 2.35 (m, 1H), 2.22 – 2.16 (m, 0.5H), 2.15 – 2.10 (m, 0.5H), 2.02 – 1.94 (m, 1H), 1.85 – 1.78 (m, 1H), 1.71 – 1.66 (m, 2H), 1.59 – 1.53 (m, 2H), 1.52 – 1.46 (m, 1H), 1.44 – 1.37 (m, 3H), 1.25 – 1.21 (m, 3H), 1.08 – 1.00 (m, 1H), 0.93 – 0.86 (m, 11H), 0.75 – 0.70 (m, 3H).

^{13}C NMR (151 MHz, CDCl$_3$): δ 172.6, 172.4, 171.8, 171.6, 150.88, 150.81, 131.20, 131.15, 128.2, 128.0, 126.6, 126.4, 119.61, 119.55, 116.9, 103.0, 102.8, 76.13, 76.08, 60.68, 60.65, 50.6, 50.4, 46.93, 46.88, 41.0, 40.8, 40.6, 40.5, 38.8, 38.5, 35.3, 35.1, 34.20, 34.15, 31.54, 31.51, 30.9, 30.74, 30.70, 26.2, 26.0, 22.94, 22.86, 22.13, 22.09, 22.06, 21.00, 20.96, 15.8, 15.6, 14.34, 14.31, 13.8.

IR (neat): 2955, 2870, 2223, 1720, 1633, 1456, 1371, 1209, 1179, 1095, 1036, 963 cm^{-1}.

HRMS (ESI-TOF) calcd. for $C_{29}H_{43}NNaO_4S$ [M+Na]$^+$: 524.2805, found: 524.2805.

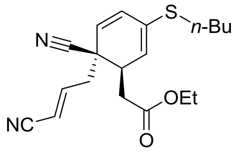

6e

Ethyl 2-((1*R*,6*S*)-3-(butylthio)-6-cyano-6-((*E*)-3-cyanoallyl)cyclohexa-2,4-dien-1-yl)acetate (6e):

Following the general procedure, **6e** were obtained from the reaction of **1f** with **2a** and **3a**. The title compound was obtained as yellow oil, 118.7 mg, 69% yield. (Rf = 0.44, eluent: PE/EtOAc = 2/1).

^{1}H NMR (600 MHz, CDCl$_3$): δ 6.78 – 6.67 (m, 1H), 6.06 – 5.97 (m, 1H), 5.67 (d, J = 9.6 Hz, 1H), 5.57 (d, J = 5.6 Hz, 1H), 5.49 (d, J = 16.2 Hz, 1H), 4.22 – 4.09 (m, 2H), 2.94 – 2.87 (m, 1H),

2.80 – 2.73 (m, 2H), 2.72 – 2.65 (m, 2H), 2.59 – 2.51 (m, 2H), 1.60 – 1.5 (m, 2H), 1.45 – 1.37 (m, 2H), 1.26 (t, *J* = 7.1 Hz, 3H), 0.91 (t, *J* = 7.4 Hz, 3H).

^{13}C NMR (151 MHz, $CDCl_3$): δ 171.1, 147.9, 131.5, 128.2, 124.0, 120.0, 118.4, 116.4, 105.3, 61.1, 40.9, 39.5, 37.5, 36.1, 30.9, 30.5, 22.1, 14.3, 13.7.

IR (neat): 2958, 2872, 2226, 1726, 1376, 1178, 1024, 967, 912 cm^{-1}.

HRMS (ESI-TOF) calcd. for $C_{19}H_{24}N_2NaO_2S$ $[M+Na]^+$: 367.1451, found: 367.1451.

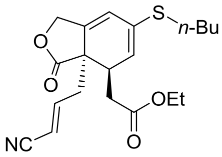

6f

Ethyl 2-((3a*R*,4*R*)-6-(butylthio)-3a-((*E*)-3-cyanoallyl)-3-oxo-1,3,3a,4-tetrahydroisobenzofuran-4-yl)acetate (6f):

Following the general procedure, **6f** were obtained from the reaction of **1g** with **2a** and **3a**. The title compound was obtained as yellow oil, 114.5 mg, 61% yield. (Rf = 0.13, eluent: PE/EtOAc = 5/1).

^{1}H NMR (400 MHz, $CDCl_3$): δ 6.72 – 6.56 (m, 1H), 5.91 (s, 1H), 5.55 (d, *J* = 6.2 Hz, 1H), 5.41 (d, *J* = 16.2 Hz, 1H), 4.91 – 4.80 (m, 2H), 4.14 – 4.03 (m, 2H), 2.96 – 2.87 (m, 1H), 2.82 – 2.64 (m, 3H), 2.58 – 2.48 (m, 1H), 2.47 – 2.39 (m, 1H), 2.36 – 2.26 (m, 1H), 1.63 – 1.52 (m, 2H), 1.47 – 1.37 (m, 2H), 1.21 (t, *J* = 7.1 Hz, 3H), 0.91 (t, *J* = 7.3 Hz, 3H).

^{13}C NMR (101 MHz, $CDCl_3$): δ 175.9, 171.1, 148.2, 134.7, 131.7, 120.0, 117.9, 116.5, 104.4, 69.2, 60.9, 48.9, 37.6, 35.8, 34.2, 31.0, 30.6, 22.1, 14.2, 13.7.

IR (neat): 2960, 2932, 2222, 1774, 1731, 1462, 1375, 1178, 1144, 1095, 1029, 964 cm^{-1}.

HRMS (ESI-TOF) calcd. for $C_{20}H_{26}NO_4S$ $[M+H]^+$: 376.1577, found: 376.1586.

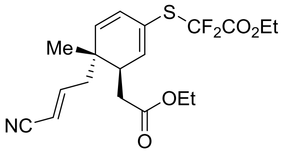

6h
(84/16 dr)

Ethyl 2-(((3*R*,4*R*)-4-((*E*)-3-cyanoallyl)-3-(2-ethoxy-2-oxoethyl)-4-methylcyclohexa-1,5-dien-1-yl)thio)-2,2-difluoroacetate (6h):

Following the general procedure, DIPEA (218 μL, 2.5 equiv) instead of 4-ethylmorpholine (157 μL, 2.5 equiv) was used, **6h** were obtained from the reaction of **1i** with **2a** and **3a**. The title compound was obtained as a mixture of two diastereoisomers (84/16 dr), yellow oil, 123.7 mg, 62% yield. (Rf = 0.26, eluent: PE/EtOAc = 5/1).

^{1}H NMR (600 MHz, CDCl$_3$): δ 6.72 – 6.61 (m, 1H), 6.33 (d, *J* = 5.2 Hz, 0.84H), 6.20 (d, *J* = 3.4 Hz, 0.16H), 6.04 – 5.94 (m, 1H), 5.54 (d, *J* = 9.7 Hz, 0.16H), 5.46 (d, *J* = 9.7 Hz, 0.84H), 5.41 – 5.31 (m, 1H), 4.36 – 4.29 (m, 2H), 4.18 – 4.07 (m, 2H), 2.89 – 2.84 (m, 0.16H), 2.72 – 2.65 (m, 0.84H), 2.54 – 2.40 (m, 2H), 2.35 – 2.25 (m, 1H), 2.21 – 2.14 (m, 0.84H), 1.98 – 1.91 (m, 0.16H), 1.38 – 1.32 (m, 3H), 1.27 – 1.21 (m, 3H), 1.12 (s, 0.48H), 1.00 (s, 2.52H).

^{13}C NMR (151 MHz, CDCl$_3$): δ 172.0, 171.9, 161.5 (t, *J* = 32.3 Hz), 151.9, 151.7, 142.8, 142.7, 136.4, 135.6, 127.8, 127.6, 121.8, 119.90 (t, *J* = 353.5 Hz), 119.86 (t, *J* = 365.7 Hz), 117.2, 102.90, 102.88, 63.80, 63.78, 61.1, 60.9, 43.2, 42.4, 40.7, 37.8, 37.1, 36.7, 32.79, 32.75, 24.6, 20.6, 14.26, 14.24, 14.00.

^{19}F NMR (377 MHz, CDCl$_3$): δ -81.76 (d, *J* = 218.0 Hz, 0.84F), -81.83 (s, 0.32F), -82.42 (d, *J* = 218.0 Hz, 0.84F).

IR (neat): 2983, 2933, 2224, 1766, 1732, 1632, 1466, 1372, 1292, 1176, 1107, 1018, 978 cm^{-1}.

HRMS (ESI-TOF) calcd. for $C_{19}H_{24}F_2NO_4S$ [M+H]$^+$: 400.1389, found: 400.1392.

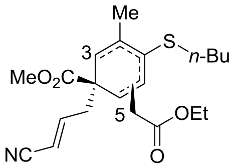

6i
C(3)/C(5) = 11/89

Methyl (1*R*,6*R*)-4-(butylthio)-1-((*E*)-3-cyanoallyl)-6-(2-ethoxy-2-oxoethyl)-5-methylcyclohexa-2,4-diene-1-carboxylate (minor): **Methyl (1*R*,6*R*)-4-(butylthio)-1-((*E*)-3-cyanoallyl)-6-(2-ethoxy-2-oxoethyl)-3-methylcyclohexa-2,4-diene-1-carboxylate** (major):

Following a procedure similar to the general procedure, the reaction of **1j** with **2a** and **3a** was conducted under modified conditions (Nu, -95 ℃, 12 h), **6i** were obtained as a mixture of two regiosiomers (**C(3)/C(5)** = 11/89), yellow oil, 97.8 mg, 50% yield. (Rf = 0.36, eluent: PE/EtOAc = 5/1).

^{1}H NMR (600 MHz, CDCl$_3$): δ 6.62 – 6.48 (m, 1H), 6.11 (d, *J* = 9.9 Hz, 0.11H), 5.93 (d, *J* = 9.7 Hz, 0.11H), 5.80 (s, 0.89H), 5.52 (d, *J* = 6.4 Hz, 0.89H), 5.32 – 5.22 (m, 1H), 4.13 – 4.02 (m, 2H), 3.74 – 3.67 (m, 3H), 2.89 – 2.82 (m, 1H), 2.81 – 2.73 (m, 1H), 2.66 – 2.59 (m, 2H), 2.50 – 2.42 (m, 1H), 2.40 – 2.32 (m, 1H), 2.13 – 2.05 (m, 1H), 1.97 (s, 0.33H), 1.89 (s, 2.67H), 1.63 – 1.54 (m, 1.78H), 1.47 – 1.38 (m, 2.22H), 1.25 – 1.18 (m, 3H), 0.96 – 0.86 (m, 3H).

^{13}C NMR (151 MHz, CDCl$_3$): δ 173.8, 173.5, 171.9, 171.8, 151.6, 151.1, 137.8, 134.6, 133.1, 127.8, 125.6, 123.9, 123.0, 118.3, 117.1, 102.6, 102.4, 60.8, 60.7, 52.50, 52.45, 51.3, 50.7, 45.4, 40.7, 39.9, 38.5, 35.1, 33.9, 32.3 32.3, 31.1, 30.1, 27.0, 22.3, 21.7, 20.9, 19.6, 14.3, 14.2, 13.8.

IR (neat): 2957, 2224, 1732, 1633, 1561, 1436, 1373, 1178, 1098, 1032, 971 cm^{-1}.

HRMS (ESI-TOF) calcd. for $C_{21}H_{30}NO_4S$ [M+H]$^+$: 392.1890, found: 392.1896.

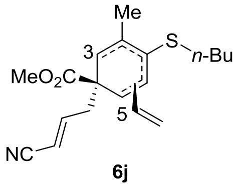

6j
C(3)/C(5) = 6/94

Methyl (1*R*,6*R*)-4-(butylthio)-1-((*E*)-3-cyanoallyl)-5-methyl-6-vinylcyclohexa-2,4-diene-1-carboxylate (minor):

Methyl (1*R*,6*R*)-4-(butylthio)-1-((*E*)-3-cyanoallyl)-3-methyl-6-vinylcyclohexa-2,4-diene-1-carboxylate (major):

Following a procedure similar to the general procedure, the reaction of **1j** with **2a** and vinylzinc chloride (**3u**, 0.35 M in THF, 4.3 mL, 3.0 equiv) was conducted under modified conditions (Nu, -95 ℃, 12 h), **6j** were obtained as a mixture of two regiosiomers (**C(3)**/**C(5)** = 6/94), yellow oil, 74.5 mg, 45% yield. (Rf = 0.45, eluent: PE/EtOAc = 5/1).

^{1}H NMR (600 MHz, CDCl$_3$): δ 6.62 – 6.54 (m, 1H), 6.11 (d, *J* = 9.9 Hz, 0.06H), 5.93 (d, *J* = 10.0 Hz, 0.06H), 5.78 (s, 0.94H), 5.64 – 5.55 (m, 1H), 5.34 – 5.25 (m, 1.94H), 5.01 (d, *J* = 16.4 Hz, 1H), 4.97 – 4.91 (m, 1H), 3.67 – 3.63 (m, 3H), 2.91 – 2.87 (m, 1H), 2.80 – 2.75 (m, 1H), 2.66 (t, *J* = 7.4 Hz, 2H), 2.53 – 2.48 (m, 1H), 1.94 – 1.90 (m, 3H), 1.65 – 1.59 (m, 2H), 1.47 – 1.40 (m, 2H), 0.92 (t, *J* = 7.4 Hz, 3H).

^{13}C NMR (151 MHz, CDCl$_3$): δ 173.6, 151.7, 151.0, 134.4, 134.1, 133.0, 125.1, 123.1, 117.6, 117.3, 117.2, 102.6, 102.4, 55.2, 52.1, 51.4, 48.5, 40.6, 40.3, 32.5, 32.2, 31.3, 30.2, 22.4, 21.8, 19.9, 19.7, 13.8.

IR (neat): 2956, 2930, 2224, 1732, 1683, 1634, 1435, 1249, 1207, 971, 924 cm^{-1}.

HRMS (ESI-TOF) calcd. for $C_{19}H_{26}NO_2S$ [M+H]$^+$: 332.1679, found: 332.1683.

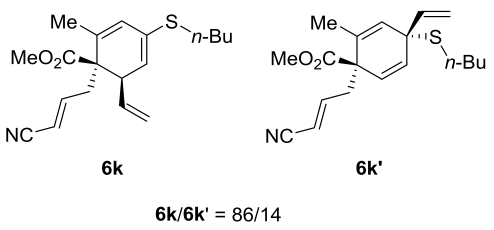

Methyl (1*R*,6*R*)-4-(butylthio)-1-((*E*)-3-cyanoallyl)-2-methyl-6-vinylcyclohexa-2,4-diene-1-carboxylate (6k): Methyl (1R,4R)-4-(butylthio)-1-((E)-3-cyanoallyl)-2-methyl-4-vinylcyclohexa-2,5-diene-1-carboxylate (6k'):

Following a procedure similar to the general procedure, the reaction of **1k** with **2a** (10.0 equiv) and vinylzinc chloride (**3u**, 0.35 M in THF, 4.3 mL, 3.0 equiv) was conducted under modified conditions (Nu, -95 ℃, 12 h), **6j** were obtained as a mixture of **6k** and **6k'** (**6k**/**6k'** = 86/14), yellow oil, 87.8 mg, 53% yield. (Rf = 0.40, eluent: PE/EtOAc = 5/1).

^{1}H NMR (600 MHz, $CDCl_3$): δ 6.65 – 6.51 (m, 1H), 6.12 (d, *J* = 9.9 Hz, 0.14H), 5.94 (d, *J* = 9.9 Hz, 0.14H), 5.79 (s, 0.86H), 5.66 – 5.54 (m, 1H), 5.37 – 5.24 (m, 2H), 5.08 – 5.00 (m, 1.14H), 4.97 – 4.93 (m, 0.86H), 3.70 – 3.64 (m, 3H), 2.92 – 2.88 (m, 0.86H), 2.83 – 2.74 (m, 1H), 2.70 – 2.64 (m, 2H), 2.55 – 2.48 (m, 1H), 1.97 – 1.89 (m, 3H), 1.68 – 1.60 (m, 1.72H), 1.51 – 1.38 (m, 2.28H), 0.97 – 0.88 (m, 3H).

^{13}C NMR (151 MHz, $CDCl_3$): δ 173.7, 173.4, 151.7, 151.0, 136.4, 134.5, 134.2, 133.2, 133.1, 127.9, 125.1, 123.2, 118.2, 117.7, 117.3, 117.2, 117.0, 102.7, 102.4, 55.23, 52.25, 52.2, 51.7, 51.4, 48.5, 40.6, 40.4, 32.5, 32.3, 31.3, 30.3, 22.4, 21.8, 20.0, 19.8, 13.8.

IR (neat): 2956, 2872, 1734, 1633, 1435, 1381, 1249, 1199, 1027, 998, 972, 921 cm^{-1}.

HRMS (ESI-TOF) calcd. for $C_{19}H_{26}NO_2S$ $[M+H]^+$: 332.1679, found: 332.1682.

Following a procedure similar to the general procedure, the reaction of **1a** with **2b** and **3a** was conducted under modified conditions (Nu, -95 ℃, 12 h), **6l** and **6l'** were obtained in 43% and 9% yields, respectively.

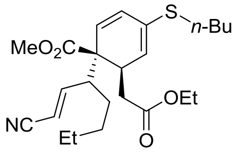

6l
(58/42 dr)

Methyl (1*R*,6*R*)-4-(butylthio)-1-((*S*,*E*)-1-cyanohept-1-en-3-yl)-6-(2-ethoxy-2-oxoethyl)cyclohexa-2,4-diene-1-carboxylate (6l):

The title compound was obtained as a mixture of two diastereoisomers (58/42 dr), yellow oil, 93.2 mg, 43% yield. (Rf = 0.34, eluent: PE/EtOAc = 5/1).

^{1}H NMR (400 MHz, CDCl$_3$): δ 6.80 – 6.70 (m, 0.58H), 6.40 – 6.30 (m, 0.42H), 6.28 – 6.22 (m, 0.58H), 6.00 – 5.85 (m, 1.42H), 5.62 – 5.56 (m, 0.58H), 5.41 – 5.37 (m, 0.42H), 5.36 – 5.22 (m, 1H), 4.15 – 4.05 (m, 2H), 3.79 – 3.72 (m, 3H), 2.98 – 2.88 (m, 1H), 2.73 – 2.50 (m, 3H), 2.48 – 2.36 (m, 1H), 2.14 – 2.07 (m, 0.42H), 2.05 – 1.99 (m, 0.58H), 1.60 – 1.50 (m, 2H), 1.47 – 1.38 (m, 2H), 1.31 – 1.09 (m, 8H), 1.05 – 0.96 (m, 1H), 0.95 – 0.89 (m, 3H), 0.86 – 0.80 (m, 3H).

^{13}C NMR (101 MHz, CDCl$_3$): δ 173.6, 172.8, 171.7, 171.6, 157.0, 156.1, 131.8, 131.7, 129.3, 126.9, 126.1, 125.8, 120.0, 119.7, 117.3, 117.2, 102.3, 100.2, 60.8, 60.7, 54.7, 53.2, 52.5, 52.3, 52.1, 50.4, 37. 8, 37.0, 36.2, 35.1, 31.0, 30.9, 30.8, 30.7, 30.4, 29.9, 29.8, 28.8, 22.4, 22.3, 22.2, 22.1, 14.3, 13.94, 13.93, 13.8, 13.8.

IR (neat): 2957, 2930, 2872, 2218, 1733, 1626, 1458, 1373, 1212, 1176, 972 cm^{-1}.

HRMS (ESI-TOF) calcd. for $C_{24}H_{35}NNaO_4S$ [M+Na]$^+$: 456.2179, found: 456.2184.

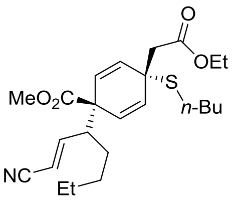

6l'

Methyl (1*r*,4*S*)-4-(butylthio)-1-((*S*,*E*)-1-cyanohept-1-en-3-yl)-4-(2-ethoxy-2-oxoethyl)cyclohexa-2,5-diene-1-carboxylate (6l'):

The title compound was obtained as colorless oil, 19.5 mg, 9% yield. (Rf = 0.26, eluent: PE/EtOAc = 5/1).

^{1}H NMR (600 MHz, CDCl$_3$): δ 6.45 (dd, *J* = 16.2, 10.4 Hz, 1H), 6.06 – 5.78 (m, 4H), 5.32 (d, *J* = 16.2 Hz, 1H), 4.03 (q, *J* = 7.1 Hz, 2H), 3.67 (s, 3H), 2.66 – 2.53 (m, 3H), 2.39 (t, *J* = 7.3 Hz, 2H), 1.57 – 1.51 (m, 2H), 1.50 – 1.45 (m, 1H), 1.42 – 1.35 (m, 2H), 1.31 – 1.19 (m, 7H), 1.12 – 1.04 (m, 1H), 0.92 – 0.84 (m, 6H).

^{13}C NMR (101 MHz, CDCl$_3$): δ 172.5, 168.9, 155.0, 133.5, 132.7, 126.6, 126.3, 116.9, 102.7, 60.7, 52.6, 51.9, 51.4, 46.5, 45.5, 31.3, 29.9, 29.3, 29.0, 22.5, 22.3, 14.2, 14.0, 13.9.

IR (neat): 2957, 2930, 2859, 2218, 1732, 1630, 1465, 1434, 1236, 1154, 1029, 974 cm^{-1}.

HRMS (ESI-TOF) calcd. for $C_{24}H_{35}NNaO_4S$ [M+Na]$^+$: 456.2179, found: 456.2186.

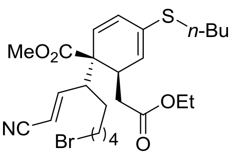

6m
(53/47 dr)

Methyl (1*R*,6*R*)-1-((*S*,*E*)-8-bromo-1-cyanooct-1-en-3-yl)-4-(butylthio)-6-(2-ethoxy-2-oxoethyl)cyclohexa-2,4-diene-1-carboxylate (6m):

Following a procedure similar to the general procedure, the reaction of **1a** with **2c** and **3a** was conducted under modified conditions (Nu, -95 ℃, 12 h), **6m** were obtained as a mixture of two diastereoisomers (53/47 dr), yellow oil, 131.6 mg, 50% yield. (Rf = 0.32, eluent: PE/EtOAc = 5/1).

^{1}H NMR (400 MHz, $CDCl_3$): δ 6.80 – 6.66 (m, 0.53H), 6.37 – 6.28 (m, 0.47H), 6.22 (d, *J* = 9.9 Hz, 0.47H), 5.97 – 5.82 (m, 1.53H), 5.55 (d, *J* = 6.3 Hz, 0.53H), 5.39 – 5.23 (m, 1.47H), 4.14 – 4.03 (m, 2H), 3.77 – 3.71 (m, 3H), 3.41 – 3.31 (m, 2H), 2.96 – 2.84 (m, 1H), 2.73 – 2.49 (m, 3H), 2.46 – 2.33 (m, 1H), 2.11 – 1.96 (m, 1H), 1.82 – 1.70 (m, 2H), 1.61 – 1.51 (m, 2H), 1.46 – 1.25 (m, 6H), 1.24 – 1.13 (m, 4H), 1.09 – 1.00 (m, 1H), 0.93 – 0.87 (m, 3H).

^{13}C NMR (101 MHz, $CDCl_3$): δ 173.5, 172.6, 171.6, 171.5, 156.6, 155.8, 131.8, 131.6, 129.0, 126.9, 126.1, 125.4, 119.3, 119.2, 117.15, 117.07, 102.4, 100.3, 60.72, 60.67, 54.5, 53.0, 52.5, 52.4, 51.9, 50.2, 37.7, 36.9, 36.1, 35.0, 33.7, 32.40, 32.37, 30.9, 30.8, 30.6, 30.5, 30.0, 28.9, 27.8, 27.7, 27.3, 26.7, 22.2, 22.1, 14.3, 13.8, 13.7.

IR (neat): 2932, 2859, 2222, 1727, 1628, 1435, 1370, 1249, 1210, 1175, 1030, 975 cm^{-1}.

HRMS (ESI-TOF) calcd. for $C_{25}H_{36}BrNNaO_4S$ $[M+Na]^+$: 548.1441, found: 548.1433.

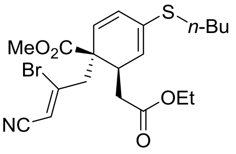

6n

Methyl (1*S*,6*R*)-1-((*Z*)-2-bromo-3-cyanoallyl)-4-(butylthio)-6-(2-ethoxy-2-oxoethyl)cyclohexa-2,4-diene-1-carboxylate (6n):

Following a procedure similar to the general procedure, the reaction of **1a** with **2d** and **3a** was conducted under modified conditions (Nu, -95 ℃, 12 h), **6n** were obtained as yellow oil, 89.1 mg, 39% yield. (Rf = 0.24, eluent: PE/EtOAc = 5/1).

^{1}H NMR (400 MHz, $CDCl_3$): δ 6.08 (d, *J* = 9.9 Hz, 1H), 6.00 – 5.94 (m, 1H), 5.76 (s, 1H), 5.64 (d, *J* = 6.3 Hz, 1H), 4.10 (q, *J* = 7.1 Hz, 2H), 3.78 (s, 3H), 3.31 (d, *J* = 13.9 Hz, 1H), 3.01 (d, *J* = 13.9 Hz, 1H), 2.95 – 2.87 (m, 1H), 2.73 – 2.61 (m, 2H), 2.46 – 2.38 (m, 1H), 2.13 – 2.04 (m, 1H), 1.59 – 1.53 (m, 2H), 1.46 – 1.38 (m, 2H), 1.24 (t, *J* = 7.2 Hz, 3H), 0.92 (t, *J* = 7.3 Hz, 3H).

^{13}C NMR (101 MHz, CDCl$_3$): δ 173.1, 171.5, 144.6, 131.6, 126.8, 126.4, 118.8, 115.7, 105.7, 60.9, 52.8, 50.7, 46.4, 39.0, 34.8, 30.9, 30.6, 22.2, 14.3, 13.8.

IR (neat): 2956, 2931, 2225, 1728, 1611, 1435, 1372, 1252, 1202, 1178, 1097, 1029 cm^{-1}.

HRMS (ESI-TOF) calcd. for $C_{20}H_{26}BrNNaO_4S$ $[M+Na]^+$: 478.0658, found: 478.0657.

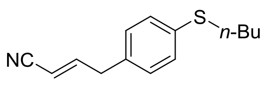

7

(*E*)-4-(4-(butylthio)phenyl)but-2-enenitrile (7):

Following the general procedure, **7** were obtained from the reaction of **1n** with **2a** and **3a**. The title compound was obtained as colorless oil, 46.3 mg, 40% yield. (Rf = 0.31, eluent: PE/EtOAc = 10/1).

^{1}H NMR (600 MHz, CDCl$_3$): δ 7.31 – 7.27 (m, 2H), 7.05 (d, *J* = 8.2 Hz, 2H), 6.88 – 6.82 (m, 1H), 5.29 – 5.25 (m, 1H), 3.53 – 3.47 (m, 2H), 2.96 – 2.86 (m, 2H), 1.67 – 1.55 (m, 2H), 1.48 – 1.41 (m, 2H), 0.92 (t, *J* = 7.4 Hz, 3H).

^{13}C NMR (101 MHz, CDCl$_3$): δ 154.0, 136.2, 133.5, 129.4, 117.4, 101.1, 39.0, 33.4, 31.3, 22.1, 13.8.

Compound **7** is a known compound and the ^{1}H NMR and ^{13}C NMR are consistent with the reported spectra[5].

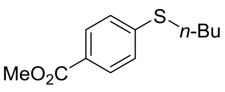

8

Methyl 4-(butylthio)benzoate (8):

Following the general procedure, **8** were obtained from the reaction of **1a** with **2e** and **3a**. The title compound was obtained as colorless oil, 89.7 mg, 80% yield. (Rf = 0.35, eluent: PE/EtOAc = 20/1).

^{1}H NMR (400 MHz, CDCl$_3$): δ 7.94 (d, *J* = 8.8 Hz, 2H), 7.30 (d, *J* = 8.9 Hz, 2H), 3.92 (s, 3H), 3.00 (t, *J* = 7.4 Hz, 2H), 1.72 – 1.65 (m, 2H), 1.55 – 1.44 (m, 2H), 0.96 (t, *J* = 7.4 Hz, 3H).

^{13}C NMR (101 MHz, CDCl$_3$): δ 167.0, 144.6, 130.0, 126.6, 126.3, 52.2, 31.8, 30.9, 22.2, 13.8.

Compound **8** is a known compound and the ^{1}H NMR and ^{13}C NMR are consistent with the reported spectra[28].

5 General procedure for the dearomative 1,4-dual functionalization and dearomative cyclization of aryl sulfoxides

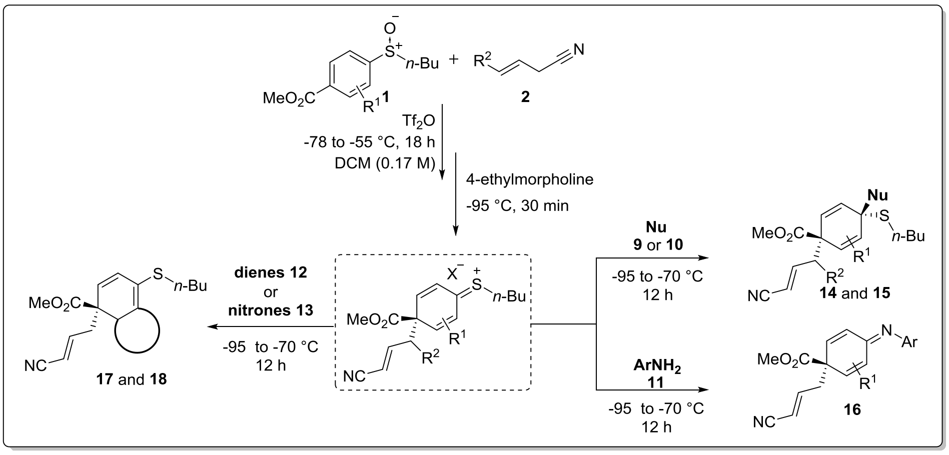

To a mixture of aryl sulfoxide **1** (0.5 mmol) and allyl nitrile **2** (1.5 mmol) in DCM (3.0 mL) was added Tf_2O (126 μL, 0.75 mmol) at -78 ℃ under N_2 atmosphere. The mixture was gradually warmed to -55 ℃. After stirring for 18 h, the mixture was cooled to -95 ℃.

Subsequent procedures for the cases of **14a-14c**, **14j**, **15a-15g**, **4c'**: to the mixture of sulfoxide **1**, allyl nitrile **2** and Tf_2O was added a mixture of 4-ethylmorpholine (157 μL, 1.25 mmol, 2.5 equiv) and nucleophile **9a-9c** (1.0 mmol, 2.0 equiv) or **10a-10f** (1.0 mmol, 2.0 equiv) in DCM (2.0 mL) dropwise in 20 min using syringe pump. After stirring for 30 min, the mixture was gradually warmed to -70 ℃ and further stirred for 12 h. Then the mixture was passed through a short silica gel column and concentrated under vacuum. The obtained residue was further purified by flash chromatography on silica gel affording the title compound.

Subsequent procedures for the cases of **14d-14i**, **16a-16k**, **17a-17j**, **18a-18e**, **4d'**: to the mixture of sulfoxide **1**, allyl nitrile **2** and Tf_2O was added a solution of 4-ethylmorpholine (157 μL, 2.5 equiv) in DCM (1.0 mL) dropwise in 10 min using syringe pump. After stirring for 30 min, to the mixture was added a solution of nucleophile **9d-9g** (1.0 mmol, 2.0 equiv), **11a-11i** (1.0 mmol, 2.0 equiv), **12a-12k** (1.5 mmol, 3.0 equiv) or **13a-13e** (2.5 mmol, 5.0 equiv) in DCM (1.0 mL) dropwise in 10 min using syringe pump. The mixture was gradually warmed to -70 ℃ and further stirred for 12 h. Then the mixture was passed through a short silica gel column and concentrated under vacuum. The obtained residue was further purified by flash chromatography on silica gel affording the title compound.

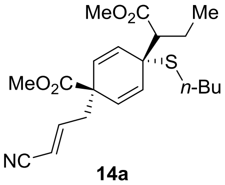

14a
(85/15 dr)

Methyl (1*S*,4*r*)-4-(butylthio)-1-((*E*)-3-cyanoallyl)-4-((*S*)-1-methoxy-1-oxobutan-2-yl)cyclohexa-2,5-diene-1-carboxylate (14a):

Following the general procedure, the title compound was obtained as a mixture of two diastereoisomers (85/15 dr), colorless oil, 154.5 mg, 79% yield. (Rf = 0.17, eluent: PE/EtOAc = 10/1).

^{1}H NMR (600 MHz, $CDCl_3$): δ 6.57 – 6.46 (m, 1H), 6.04 – 5.99 (m, 1H), 5.83 – 5.76 (m, 2H), 5.67 – 5.63 (m, 0.85H), 5.45 – 5.41 (m, 0.15H), 5.36 – 5.30 (m, 0.85H), 5.25 – 5.20 (m, 0.15H), 3.73 (s, 0.45H), 3.64 (s, 2.55H), 3.62 (s, 2.55H), 3.45 (s, 0.45H), 2.71 – 2.61 (m, 0.60H), 2.58 – 2.52 (m, 1.70H), 2.47 – 2.41 (m, 1H), 2.24 – 2.18 (m, 1.70H), 1.58 – 1.43 (m, 4H), 1.40 – 1.29 (m, 2H), 0.89 – 0.86 (m, 0.45H), 0.82 (t, *J* = 7.4 Hz, 2.55H), 0.79 – 0.74 (m, 3H).

^{13}C NMR (151 MHz, $CDCl_3$): δ 173.3, 173.0, 172.9, 172.3, 150.7, 149.9, 132.0, 131.6, 130.2, 128.4, 127.5, 127.3, 126.0, 116.6, 116.5, 103.4, 102.6, 56.3, 52.6, 52.2, 51.43, 51.36, 50.2, 50.0, 47.4, 44.6, 43.4, 42.7, 30.9, 30.7, 29.1, 23.4, 22.2, 22.1, 22.0, 13.7, 13.6, 12.3, 12.2.

IR (neat): 2954, 2874, 2224, 1727, 1433, 1231, 1159, 969, 915, 854 cm^{-1}.

HRMS (ESI-TOF) calcd. for $C_{21}H_{29}NNaO_4S$ $[M+Na]^+$: 414.1710, found: 414.1713.

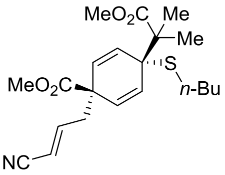

14b

Methyl (1*r*,4*r*)-4-(butylthio)-1-((*E*)-3-cyanoallyl)-4-(1-methoxy-2-methyl-1-oxopropan-2-yl)cyclohexa-2,5-diene-1-carboxylate (14b):

Following the general procedure, the title compound was obtained as colorless oil, 162.3 mg, 83% yield. (Rf = 0.21, eluent: PE/EtOAc = 5/1).

^{1}H NMR (600 MHz, $CDCl_3$): δ 6.62 – 6.54 (m, 1H), 5.98 – 5.92 (m, 2H), 5.86 – 5.82 (m, 2H), 5.39 – 5.33 (m, 1H), 3.68 (s, 3H), 3.66 (s, 3H), 2.61 – 2.57 (m, 2H), 2.13 (t, *J* = 7.3 Hz, 2H), 1.54 – 1.46 (m, 2H), 1.39 – 1.32 (m, 2H), 1.24 (s, 6H), 0.86 (t, *J* = 7.3 Hz, 3H).

^{13}C NMR (151 MHz, $CDCl_3$): δ 175.0, 172.5, 149.9, 130.5, 127.5, 116.7, 103.6, 54.0, 52.7, 51.9, 50.2, 47.1, 43.7, 31.0, 29.6, 22.4, 22.2, 13.9.

IR (neat): 2957, 2871, 2222, 1729, 1718, 1434, 1263, 980, 912 cm^{-1}.

HRMS (ESI-TOF) calcd. for $C_{21}H_{29}NNaO_4S$ $[M+Na]^+$: 414.1710, found: 414.1724.

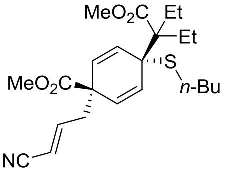

14c

Methyl (1r,4*r*)-4-(butylthio)-1-((*E*)-3-cyanoallyl)-4-(3-(methoxycarbonyl)pentan-3-yl)cyclohexa-2,5-diene-1-carboxylate (14c):

Following the general procedure, the title compound was obtained as colorless oil, 142.5mg, 68% yield. (Rf = 0.27, eluent: PE/EtOAc = 5/1).

^{1}H NMR (600 MHz, CDCl$_3$): δ 6.65 – 6.53 (m, 1H), 5.94 (d, *J* = 10.4 Hz, 2H), 5.79 (d, *J* = 10.4 Hz, 2H), 5.35 (d, *J* = 16.2 Hz, 1H), 3.72 – 3.64 (m, 6H), 2.60 – 2.54 (m, 2H), 2.07 (t, *J* = 7.3 Hz, 2H), 1.97 – 1.88 (m, 2H), 1.83 – 1.73 (m, 2H), 1.53 – 1.45 (m, 2H), 1.39 – 1.30 (m, 2H), 0.91 – 0.82 (m, 9H).

^{13}C NMR (151 MHz, CDCl$_3$): δ 174.0, 172.5, 149.9, 130.9, 126.5, 116.7, 103.6, 57.5, 54.9, 52.7, 51.5, 47.0, 44.0, 30.8, 29.6, 24.4, 22.5, 13.9, 10.2.

IR (neat): 2953, 2224, 1722, 1432, 1224, 1127, 969, 914, 840 cm^{-1}.

HRMS (ESI-TOF) calcd. for $C_{23}H_{33}NNaO_4S$ $[M+Na]^+$: 442.2023, found: 442.2026.

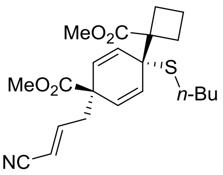

14d

Methyl (1*r*,4*r*)-4-(butylthio)-1-((*E*)-3-cyanoallyl)-4-(1-(methoxycarbonyl)cyclobutyl)cyclohexa-2,5-diene-1-carboxylate (14d):

Following the general procedure, the title compound was obtained as white solid, m.p. 93 – 95 ℃, 151.2 mg, 75% yield. (Rf = 0.23, eluent: PE/EtOAc = 5/1).

^{1}H NMR (600 MHz, CDCl$_3$): δ 6.66 – 6.56 (m, 1H), 5.96 – 5.90 (m, 2H), 5.89 – 5.83 (m, 2H), 5.36 (d, *J* = 16.2 Hz, 1H), 3.76 – 3.63 (m, 6H), 2.64 – 2.56 (m, 2H), 2.44 – 2.30 (m, 4H), 2.18 (t, *J* = 7.3 Hz, 2H), 1.81 – 1.72 (m, 2H), 1.59 – 1.49 (m, 2H), 1.44 – 1.34 (m, 2H), 0.88 (t, *J* = 7.3 Hz, 3H).

^{13}C NMR (151 MHz, CDCl$_3$): δ 175.3, 172.6, 149.9, 129.8, 127.3, 116.7, 103.6, 55.3, 52.8, 52.3, 52.1, 47.4, 44.0, 31.1, 29.5, 27.3, 22.5, 15.8, 13.9.

IR (neat): 2951, 2847, 2224, 1730, 1714, 1633, 1436, 1263, 1202, 1106, 985, 915, 873 cm^{-1}.

HRMS (ESI-TOF) calcd. for $C_{22}H_{29}NNaO_4S$ $[M+Na]^+$: 426.1710, found: 426.1709.

Supplementary Table 3. Crystal data and structure refinement for **14d**

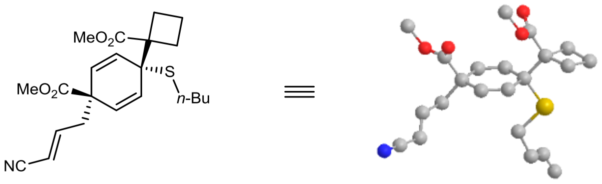

14d

Bond precision:	c-c = 0.0063 Å	Wavelength=0.71073	
Cell:	a=6.1246(6)	b=10.3701(11)	c=18.213(2)
	α=94.290(3)	β=98.555(3)	γ=104.375(3)
Temperature:	295 K		

	Calculated	Reported
Volume	1100.5(2)	1100.5(2)
Space group	P -1	P-1
Hall group	-P 1	
Moiety formula	C_{22} H_{29} N O_4 S	C_{22} H_{29} N O_4 S
Sum formula	C_{22} H_{29} N O_4 S	C_{22} H_{29} N O_4 S
Mr	403.52	403.52
Dx,g cm^{-3}	1.218	1.218
Z	2	2
Mu (mm^{-1})	0.173	0.173
F000	432.0	432.0
F000'	432.44	
h,k,lmax	7,13,23	7,13,23
Nref	5039	4935
Tmin,Tmax	0.949,0.979	0.949,0.979
Tmin'	0.933	

Correction method= # Reported T Limits: Tmin=0.949 Tmax=0.979
AbsCorr = EMPIRICAL

Data completeness= 0.979 Theta(max)= 27.480

R(reflections)= 0.0736(2695) wR2(reflections)= 0.2525(4935)

S = 1.084 Npar= 253

The single crystal of **14d** was obtained by recrystallization in a solution of DCM/Hexane at rt. Crystallographic data (excluding structural factors) for this compound **14d** has been deposited at the Cambridge Crystallographic Data Centre under the deposition number CCDC 2020058.

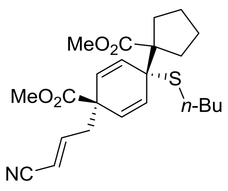

14e

Methyl (1*r*,4*r*)-4-(butylthio)-1-((*E*)-3-cyanoallyl)-4-(1-(methoxycarbonyl)cyclopentyl)cyclohexa-2,5-diene-1-carboxylate (14e):

Following the general procedure, the title compound was obtained as white solid, m.p. 96 – 98 ℃, 129.3 mg, 62% yield. (Rf = 0.32, eluent: PE/EtOAc = 5/1).

^{1}H NMR (600 MHz, $CDCl_3$): δ 6.64 – 6.51 (m, 1H), 5.91 (d, *J* = 10.3 Hz, 2H), 5.80 (d, *J* = 10.3 Hz, 2H), 5.34 (d, *J* = 16.2 Hz, 1H), 3.68 (s, 3H), 3.61 (s, 3H), 2.61 – 2.52 (m, 2H), 2.27 – 2.17 (m, 2H), 2.13 (t, *J* = 7.3 Hz, 2H), 1.93 – 1.83 (m, 2H), 1.63 – 1.54 (m, 2H), 1.54 – 1.42 (m, 4H), 1.40 – 1.31 (m, 2H), 0.86 (t, *J* = 7.3 Hz, 3H).

^{13}C NMR (151 MHz, $CDCl_3$): δ 174.9, 172.5, 149.9, 131.0, 126.7, 116.7, 103.5, 63.1, 53.6, 52.7, 52.1, 47.2, 44.1, 31.7, 31.0, 29.6, 24.8, 22.4, 13.9.

IR (neat): 2954, 2870, 2222, 1729, 1713, 1633, 1434, 1261, 1193, 979, 910 cm^{-1}.

HRMS (ESI-TOF) calcd. for $C_{23}H_{31}NNaO_4S$ $[M+Na]^+$: 440.1866, found: 440.1869.

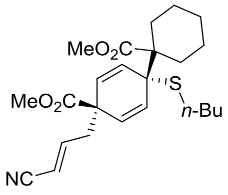

14f

Dimethyl (1'*r*,4'*r*)-1'-(butylthio)-4'-((*E*)-3-cyanoallyl)-[1,1'-bi(cyclohexane)]-2',5'-diene-1,4'-dicarboxylate (14f):

Following the general procedure, the title compound was obtained as white solid, m.p. 89 – 91 ℃, 146.6 mg, 68% yield. (Rf = 0.27, eluent: PE/EtOAc = 5/1).

^{1}H NMR (600 MHz, $CDCl_3$): δ 6.62 – 6.53 (m, 1H), 5.90 – 5.85 (m, 2H), 5.84 – 5.78 (m, 2H), 5.35 (d, *J* = 16.2 Hz, 1H), 3.69 (s, 3H), 3.68 (s, 3H), 2.60 – 2.55 (m, 2H), 2.23 (d, *J* = 12.4 Hz, 2H), 2.09 (t, *J* = 7.3 Hz, 2H), 1.62 (d, *J* = 13.6 Hz, 2H), 1.57 – 1.46 (m, 3H), 1.39 – 1.31 (m, 4H), 1.23 – 1.12 (m, 2H), 1.12 – 1.02 (m, 1H), 0.86 (t, *J* = 7.3 Hz, 3H).

^{13}C NMR (151 MHz, $CDCl_3$): δ 173.2, 172.5, 149.9, 130.5, 127.2, 116.7, 103.6, 55.8, 54.5, 52.7, 51.6, 47.1, 43.8, 31.0, 30.1, 29.3, 25.5, 24.0, 22.4, 13.9.

IR (neat): 2935, 2849, 2222, 1729, 1716, 1434, 1214, 979 cm^{-1}.

HRMS (ESI-TOF) calcd. for $C_{24}H_{33}NNaO_4S$ $[M+Na]^+$: 454.2023, found: 454.2041.

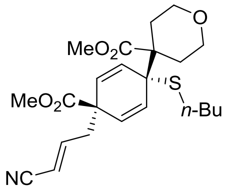

14g

Methyl 4-((1*r*,4*r*)-1-(butylthio)-4-((*E*)-3-cyanoallyl)-4-(methoxycarbonyl)cyclohexa-2,5-dien-1-yl)tetrahydro-2*H*-pyran-4-carboxylate (14g):

Following the general procedure, the title compound was obtained as white solid, m.p. 100 – 102 ℃, 110.5 mg, 51% yield. (Rf = 0.11, eluent: PE/EtOAc = 5/1).

^{1}H NMR (600 MHz, $CDCl_3$): δ 6.61 – 6.52 (m, 1H), 5.86 (s, 4H), 5.36 (d, *J* = 16.2 Hz, 1H), 3.89 – 3.82 (m, 2H), 3.74 (s, 3H), 3.69 (s, 3H), 3.26 (t, *J* = 11.7 Hz, 2H), 2.59 (d, *J* = 7.7 Hz, 2H), 2.16 – 2.06 (m, 4H), 1.83 – 1.74 (m, 2H), 1.55 – 1.47 (m, 2H), 1.40 – 1.42 (m, 2H), 0.90 – 0.83 (m, 3H).

^{13}C NMR (151 MHz, $CDCl_3$): δ 172.5, 172.2, 149.7, 129.9, 127.9, 116.7, 103.7, 66.0, 53.84, 53.80, 52.8, 52.0, 47.2, 43.7, 31.0, 30.2, 29.3, 22.4, 13.9.

IR (neat): 2948, 2848, 2765, 2226, 1730, 1635, 1440, 1260, 1201, 1101, 916, 797 cm^{-1}.

HRMS (ESI-TOF) calcd. for $C_{23}H_{31}NNaO_5S$ $[M+Na]^+$: 456.1815, found: 456.1816.

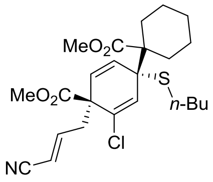

14h

Dimethyl (1'*S*,4'*R*)-1'-(butylthio)-3'-chloro-4'-((*E*)-3-cyanoallyl)-[1,1'-bi(cyclohexane)]-2',5'-diene-1,4'-dicarboxylate (14h):

Following a procedure similar to the general procedure, the reaction of **1l** with **2a** and **9f** was conducted under modified conditions (Nu, -95 ℃, 12 h), **14h** were obtained as yellow oil, 100.0 mg, 43% yield. (Rf = 0.27, eluent: PE/EtOAc = 5/1).

^{1}H NMR (600 MHz, $CDCl_3$): δ 6.64 – 6.54 (m, 1H), 6.15 (s, 1H), 5.91 (d, *J* = 10.0 Hz, 1H), 5.67 (d, *J* = 10.0 Hz, 1H), 5.44 (d, *J* = 16.2 Hz, 1H), 3.74 (s, 3H), 3.71 (s, 3H), 3.01 – 2.94 (m, 1H), 2.87 – 2.79 (m, 1H), 2.36 – 2.28 (m, 1H), 2.24 – 2.18 (m, 1H), 2.18 – 2.11 (m, 2H), 1.70 – 1.60 (m, 2H), 1.60 – 1.48 (m, 3H), 1.41 – 1.28 (m, 4H), 1.25 – 1.16 (m, 2H), 1.13 – 1.05 (m, 1H), 0.87 (t, *J* = 7.3 Hz, 3H).

^{13}C NMR (151 MHz, $CDCl_3$): δ 172.9, 170.2, 150.1, 131.2, 131.0, 130.8, 126.3, 116.7, 103.8, 57.2, 56.4, 53.1, 52.6, 51.7, 40.5, 30.8, 30.68, 30.62, 29.4, 25.4, 23.98, 23.96, 22.1, 13.9.

IR (neat): 2932, 2215, 1735, 1453, 1434, 1307, 1222, 1134, 970 cm^{-1}.

HRMS (ESI-TOF) calcd. for $C_{24}H_{33}ClNO_4S$ [M+H]$^+$: 466.1813, found: 466.1809.

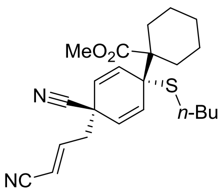

14i

Methyl (1'*r*,4'*r*)-1'-(butylthio)-4'-cyano-4'-((*E*)-3-cyanoallyl)-[1,1'-bi(cyclohexane)]-2',5'-diene-1-carboxylate (14i):

Following the general procedure, the title compound was obtained as colorless oil, 113.5 mg, 57% yield. (Rf = 0.25, eluent: PE/EtOAc = 5/1).

^{1}H NMR (600 MHz, CDCl$_3$): δ 6.68 (dt, *J* = 15.8, 7.7 Hz, 1H), 6.04 (d, *J* = 10.1 Hz, 2H), 5.72 (d, *J* = 10.1 Hz, 2H), 5.51 (d, *J* = 16.2 Hz, 1H), 3.74 (s, 3H), 2.67 – 2.57 (m, 2H), 2.36 – 2.26 (m, 2H), 2.03 (t, *J* = 7.3 Hz, 2H), 1.72 – 1.66 (m, 2H), 1.61 – 1.55 (m, 1H), 1.52 – 1.40 (m, 4H), 1.38 – 1.30 (m, 2H), 1.24 – 1.17 (m, 2H), 1.14 – 1.06 (m, 1H), 0.86 (t, *J* = 7.3 Hz, 3H).

^{13}C NMR (151 MHz, CDCl$_3$): δ 172.8, 147.0, 132.8, 123.3, 118.9, 116.1, 105.6, 56.0, 54.0, 52.0, 44.0, 35.5, 30.8, 30.0, 29.4, 25.4, 23.9, 22.4, 13.8.

IR (neat): 2930, 2860, 2362, 2252, 2227, 1720, 1636, 1453, 1306, 1218, 1133, 967 cm^{-1}.

HRMS (ESI-TOF) calcd. for $C_{23}H_{30}N_2NaO_2S$ [M+Na]$^+$: 421.1920, found: 421.1919.

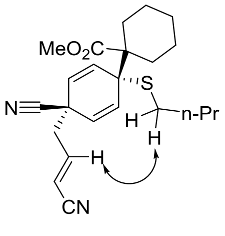

14i 2D-NOESY

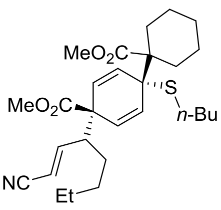

14j

Dimethyl (1'*S*,4'*r*)-1'-(butylthio)-4'-((*S*,*E*)-1-cyanohept-1-en-3-yl)-[1,1'-bi(cyclohexane)]-2',5'-diene-1,4'-dicarboxylate (14j):

Following a procedure similar to the general procedure, the reaction of **1a** with **2b** and **9f** was conducted under modified conditions (Nu, -95 ℃, 12 h), **14j** were obtained as yellow oil, 129.2 mg, 53% yield. (Rf = 0.35, eluent: PE/EtOAc = 5/1).

^{1}H NMR (400 MHz, CDCl$_3$): δ 6.46 – 6.32 (m, 1H), 5.98 – 5.74 (m, 4H), 5.29 (d, *J* = 16.2 Hz, 1H), 3.66 (s, 3H), 3.62 (s, 3H), 2.63 – 2.45 (m, 1H), 2.27 – 2.18 (m, 4H), 1.58 – 1.01 (m, 18H), 0.88 – 0.74 (m, 6H).

^{13}C NMR (151 MHz, CDCl$_3$): δ 173.1, 172.5, 154.7, 132.4, 131.5, 126.9, 126.7, 116.7, 102.6, 56.1, 54.6, 52.3, 52.0, 51.4, 50.9, 31.0, 30.0, 29.8, 29.1, 28.8, 25.4, 23.9, 22.3, 22.2, 13.87, 13.82.

IR (neat): 2933, 2850, 2223, 1729, 1430, 1203, 1134, 1032, 985, 802 cm^{-1}.

HRMS (ESI-TOF) calcd. for $C_{28}H_{41}NNaO_4S$ [M+Na]$^+$: 510.2649, found: 510.2645.

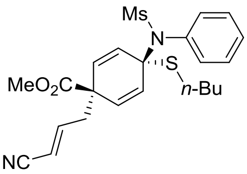

15a

Methyl (1*r*,4*r*)-4-(butylthio)-1-((*E*)-3-cyanoallyl)-4-(*N*-phenylmethylsulfonamido)cyclohexa-2,5-diene-1-carboxylate (15a):

Following the general procedure, the title compound was obtained as colorless oil, 179.5 mg, 78% yield. (Rf = 0.32, eluent: PE/EtOAc = 2/1).

^{1}H NMR (600 MHz, CDCl$_3$): δ 7.34 – 7.30 (m, 2H), 7.13 – 7.08 (m, 1H), 7.09 (d, *J* = 7.8 Hz, 2H), 6.52 – 6.43 (m, 1H), 5.85 (d, *J* = 9.9 Hz, 1H), 5.49 (d, *J* = 6.3 Hz, 1H), 5.29 (d, *J* = 16.1 Hz, 1H), 5.23 (d, *J* = 9.9 Hz, 1H), 5.16 (d, *J* = 6.3 Hz, 1H), 3.93 (s, 3H), 2.87 (s, 3H), 2.80 – 2.70 (m, 2H), 2.68 – 2.58 (m, 2H), 1.65 – 1.60 (m, 2H), 1.51 – 1.42 (m, 2H), 0.96 (t, *J* = 7.3 Hz, 3H).

^{13}C NMR (151 MHz, CDCl$_3$): δ 172.4, 150.0, 137.6, 135.6, 132.2, 129.3, 128.8, 128.5, 124.7, 116.7, 111.2, 103.3, 58.4, 52.8, 52.0, 42.5, 40.2, 30.6, 30.5, 22.2, 13.8.

IR (neat): 2957, 2867, 2224, 1732, 1633, 1492, 1437, 1333, 1155, 1034, 971, 906 cm^{-1}.

HRMS (ESI-TOF) calcd. for $C_{23}H_{29}N_2O_4S_2$ [M+H]$^+$: 461.1563, found: 461.1562.

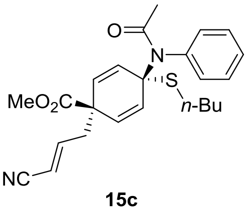

15c

Methyl (1*r*,4*r*)-4-(butylthio)-1-((*E*)-3-cyanoallyl)-4-(*N*-phenylacetamido)cyclohexa-2,5-diene-1-carboxylate (15c):

Following the general procedure, the title compound was obtained as colorless oil, 116.7 mg, 55% yield. (Rf = 0.36, eluent: PE/EtOAc = 1/1).

^{1}H NMR (600 MHz, CDCl$_3$): δ 7.32 – 7.19 (m, 4H), 6.93 – 6.78 (m, 1H), 6.57 – 6.48 (m, 1H), 5.89 (d, *J* = 9.9 Hz, 1H), 5.78 (d, *J* = 6.4 Hz, 1H), 5.36 – 5.24 (m, 3H), 3.82 – 3.77 (m, 3H), 2.76 –

2.69 (m, 1H), 2.66 – 2.59 (m, 3H). 1.65 (s, 3H), 1.56 – 1.48 (m, 2H), 1.44 – 1.36 (m, 2H), 0.92 (t, *J* = 7.3 Hz, 3H).

^{13}C NMR (151 MHz, CDCl$_3$): δ 172.9, 170.5, 150.6, 139.6, 136.4, 130.7, 130.2, 129.1, 128.8, 128.5, 127.8, 125.1, 116.9, 111.6, 103.0, 53.1, 52.6, 51.5, 41.7, 30.7, 30.5, 23.2, 22.2, 13.7.

IR (neat): 2956, 2872, 2224, 1731, 1667, 1595, 1496, 1438, 1373, 1316, 1053, 970, 912 cm^{-1}.

HRMS (ESI-TOF) calcd. for $C_{24}H_{29}N_2O_3S$ [M+H]$^+$: 425.1893, found: 425.1888.

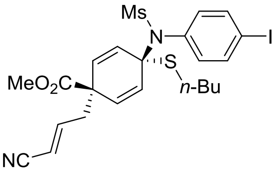

15e

Methyl (1*r*,4*r*)-4-(butylthio)-1-((*E*)-3-cyanoallyl)-4-(*N*-(4-iodophenyl)methylsulfonamido)cyclohexa-2,5-diene-1-carboxylate (15e):

Following the general procedure, the title compound was obtained as colorless oil, 205.1 mg, 70% yield. (Rf = 0.33, eluent: PE/EtOAc = 2/1).

^{1}H NMR (600 MHz, CDCl$_3$): δ 7.59 (d, *J* = 7.0 Hz, 2H), 6.83 (d, *J* = 7.1 Hz, 2H), 6.52 – 6.43 (m, 1H), 5.89 (d, *J* = 9.9 Hz, 1H), 5.46 (d, *J* = 6.4 Hz, 1H), 5.34 – 5.28 (m, 2H), 5.12 (d, *J* = 6.4 Hz, 1H), 3.91 (s, 3H), 2.87 (s, 3H), 2.79 – 2.71 (m, 2H), 2.67 – 2.59 (m, 2H), 1.66 – 1.59 (m, 2H), 1.51 – 1.43 (m, 2H), 0.96 (t, *J* = 7.3 Hz, 3H).

^{13}C NMR (101 MHz, CDCl$_3$): δ 172.4, 149.8, 138.1, 137.8, 135.5, 133.9, 128.9, 125.0, 116.7, 110.7, 103.4, 95.6, 58.5, 52.8, 52.0, 42.4, 40.2, 30.6, 30.5, 22.2, 13.8.

IR (neat): 2956, 2930, 2224, 1732, 1633, 1568, 1482, 1437, 1334, 1204, 1155, 1009, 971 cm^{-1}.

HRMS (ESI-TOF) calcd. for $C_{23}H_{27}IN_2NaO_4S_2$ [M+Na]$^+$: 609.0349, found: 609.0332.

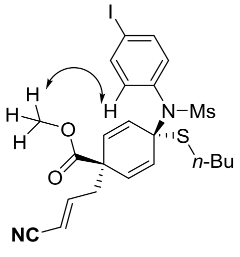

15e 2D-NOESY

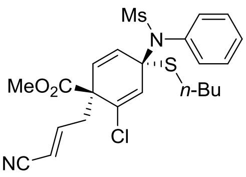

15f

Methyl (1*R*,4*S*)-4-(butylthio)-2-chloro-1-((*E*)-3-cyanoallyl)-4-(*N*-phenylmethylsulfonamido)cyclohexa-2,5-diene-1-carboxylate (15f):

Following a procedure similar to the general procedure, the reaction of **1l** with **2a** and **10a** was conducted under modified conditions (Nu, -95 ℃, 12 h), **15f** were obtained as colorless oil, 79.2 mg, 32% yield. (Rf = 0.27, eluent: PE/EtOAc = 2/1).

^{1}H NMR (400 MHz, CDCl$_3$): δ 7.53 – 7.46 (m, 2H), 7.37 – 7.27 (m, 3H), 6.83 – 6.72 (m, 1H), 5.59 (d, *J* = 1.1 Hz, 1H), 5.43 – 5.29 (m, 2H), 5.17 (d, *J* = 6.4 Hz, 1H), 3.90 (s, 3H), 2.89 – 2.79 (m, 4H), 2.73 – 2.64 (m, 1H), 2.60 (t, *J* = 7.3 Hz, 2H), 1.56 – 1.46 (m, 2H), 1.42 – 1.34 (m, 2H), 0.93 (t, *J* = 7.3 Hz, 3H).

^{13}C NMR (101 MHz, CDCl$_3$): δ 169.3, 150.0, 136.9, 135.8, 133.7, 132.9, 129.4, 128.3, 125.5, 117.0, 111.1, 103.3, 59.9, 58.4, 53.1, 40.2, 39.5, 30.8, 30.5, 22.1, 13.7.

IR (neat): 2957, 2927, 2223, 1739, 1624, 1492, 1430, 1341, 1254, 1206, 1154, 969 cm^{-1}.

HRMS (ESI-TOF) calcd. for $C_{23}H_{27}ClN_2NaO_4S_2$ $[M+Na]^+$: 517.0993, found: 517.0991.

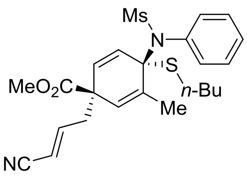

15g

Methyl (1*S*,4*S*)-4-(butylthio)-1-((*E*)-3-cyanoallyl)-3-methyl-4-(*N*-phenylmethylsulfonamido)cyclohexa-2,5-diene-1-carboxylate (15g):

Following the general procedure, **15g** were obtained from the reaction of aryl **1j** with **2a** and **10a**. The title compound was obtained as colorless oil, 109.1 mg, 46% yield. (Rf = 0.37, eluent: PE/EtOAc = 2/1).

^{1}H NMR (600 MHz, CDCl$_3$): δ 7.33 – 7.26 (m, 3H), 7.11 (d, *J* = 7.7 Hz, 2H), 6.52 – 6.44 (m, 1H), 5.61 (s, 1H), 5.42 (d, *J* = 6.6 Hz, 1H), 5.28 (d, *J* = 16.2 Hz, 1H), 5.10 (d, *J* = 6.6 Hz, 1H), 3.92 (s, 3H), 2.88 (s, 3H), 2.83 – 2.71 (m, 2H), 2.60 (d, *J* = 7.8 Hz, 2H), 1.73 – 1.66 (m, 2H), 1.56 – 1.47 (m, 2H), 1.29 (s, 3H), 0.99 (t, *J* = 7.3 Hz, 3H).

^{13}C NMR (151 MHz, CDCl$_3$): δ 172.8, 150.7, 141.4, 135.8, 132.6, 132.3, 129.0, 128.3, 124.1, 116.9, 110.3, 102.8, 58.2, 52.7, 52.0, 42.3, 40.2, 30.7, 30.0, 22.4, 18.6, 13.8.

IR (neat): 2957, 2872, 2224, 1732, 1628, 1561, 1491, 1437, 1329, 1154, 1038, 968, 908 cm^{-1}.

HRMS (ESI-TOF) calcd. for $C_{24}H_{31}N_2O_4S_2$ $[M+H]^+$: 475.1720, found: 475.1723.

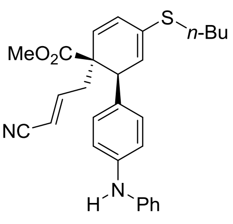

4c'

Methyl (1*S*,2*S*)-5-(butylthio)-2-((*E*)-3-cyanoallyl)-4'-(phenylamino)-1,2-dihydro-[1,1'-biphenyl]-2-carboxylate (4c'):

Following the general procedure, the title compound was obtained as yellow oil, 128.3 mg, 56% yield. (Rf = 0.25, eluent: PE/EtOAc = 5/1).

^{1}H NMR (600 MHz, CDCl$_3$): δ 7.27 – 7.22 (m, 2H), 7.07 – 7.01 (m, 4H), 6.96 – 6.89 (m, 3H), 6.68 – 6.60 (m, 1H), 6.10 – 6.06 (m, 1H), 5.99 (d, *J* = 10.0 Hz, 1H), 5.57 (d, *J* = 6.1 Hz, 1H), 5.37 (d, *J* = 16.2 Hz, 1H), 3.51 (d, *J* = 6.2 Hz, 1H), 3.38 (s, 3H), 2.97 – 2.89 (m, 1H), 2.80 – 2.71 (m, 3H), 1.68 – 1.58 (m, 2H), 1.48 – 1.40 (m, 2H), 0.92 (t, *J* = 7.4 Hz, 3H).

^{13}C NMR (151 MHz, CDCl$_3$): δ 173.1, 151.1, 142.8, 142.7, 129.9, 129.8, 129.41, 129.38, 127.8, 126.5, 121.2, 120.5, 118.0, 117.2, 117.1, 102.9, 52.8, 52.1, 49.4, 42.7, 31.0, 30.9, 22.1, 13.8.

IR (neat): 3375, 2953, 2927, 2223, 1726, 1595, 1513, 1495, 1312, 1196, 969, 909, 746 cm^{-1}.

HRMS (ESI-TOF) calcd. for $C_{28}H_{30}N_2NaO_2S$ [M+Na]$^+$: 481.1920, found: 481.1954.

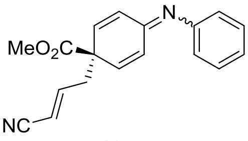

16a

Methyl 1-((*E*)-3-cyanoallyl)-4-(phenylimino)cyclohexa-2,5-dienecarboxylate (16a):

Following the general procedure, the title compound was obtained as yellow oil, 97.9 mg, 67% yield. (Rf = 0.36, eluent: PE/EtOAc = 2/1).

^{1}H NMR (600 MHz, CDCl$_3$): δ 7.34 – 7.30 (m, 2H), 7.12 – 7.08 (m, 1H), 6.81 (d, *J* = 8.1 Hz, 2H), 6.63 (dd, *J* = 10.1, 1.5 Hz, 1H), 6.52 – 6.44 (m, 3H), 6.36 (dd, *J* = 10.4, 2.5 Hz, 1H), 5.40 (d, *J* = 16.3 Hz, 1H), 3.75 (s, 3H), 2.81 – 2.72 (m, 2H).

^{13}C NMR (151 MHz, CDCl$_3$): δ 170.8, 155.5, 149.8, 148.5, 139.0, 137.1, 132.1, 129.0, 124.3, 122.3, 120.3, 116.6, 104.2, 53.4, 51.2, 41.9.

IR (neat): 3051, 2953, 2224, 1729, 1588, 1481, 1433, 1230, 967, 909 cm^{-1}.

HRMS (ESI-TOF) calcd. for $C_{18}H_{16}N_2NaO_2$ [M+Na]$^+$: 315.1104, found: 315.1104.

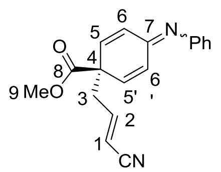

16a

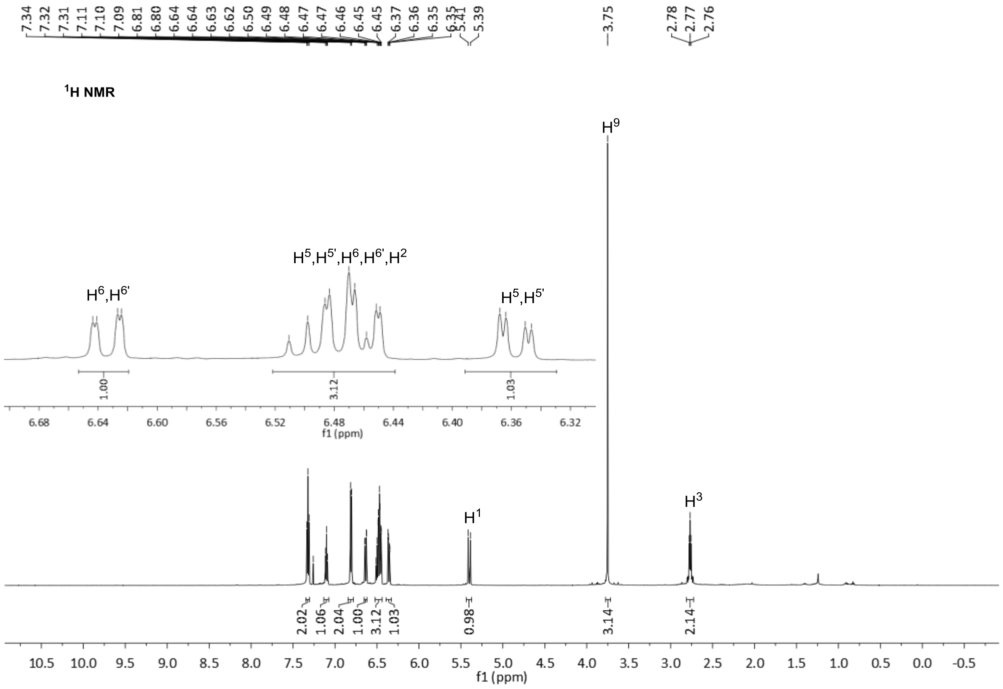

Supplementary Figure 5. ^{1}H NMR spectrum of **16a**

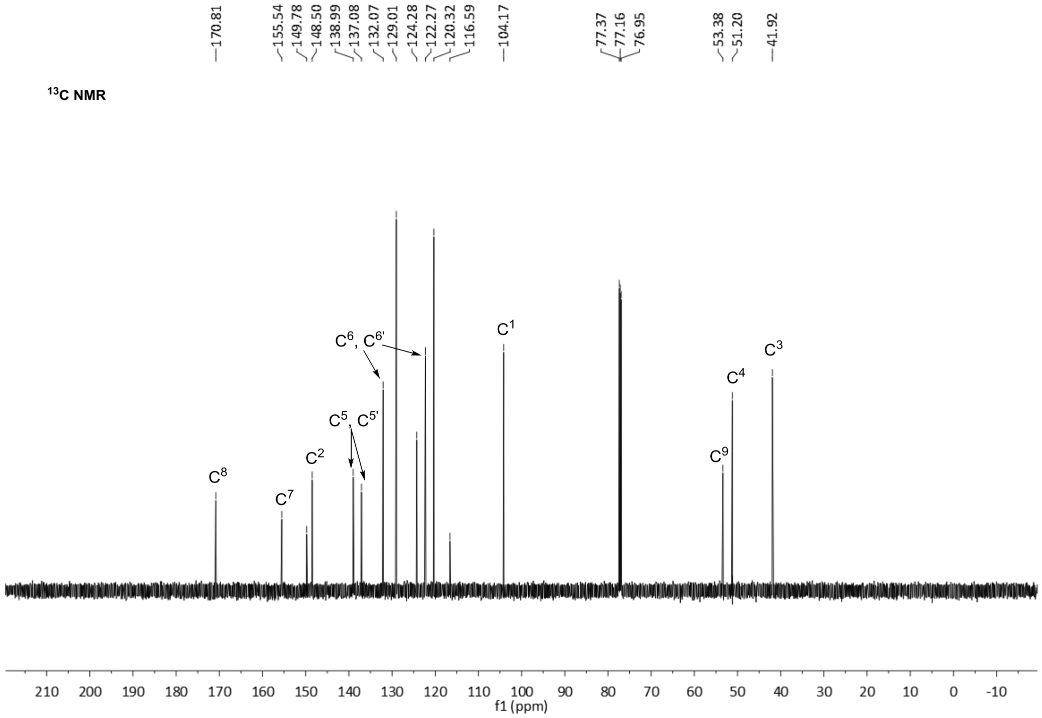

Supplementary Figure 6. ^{13}C NMR spectrum of **16a**

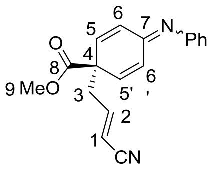

16a

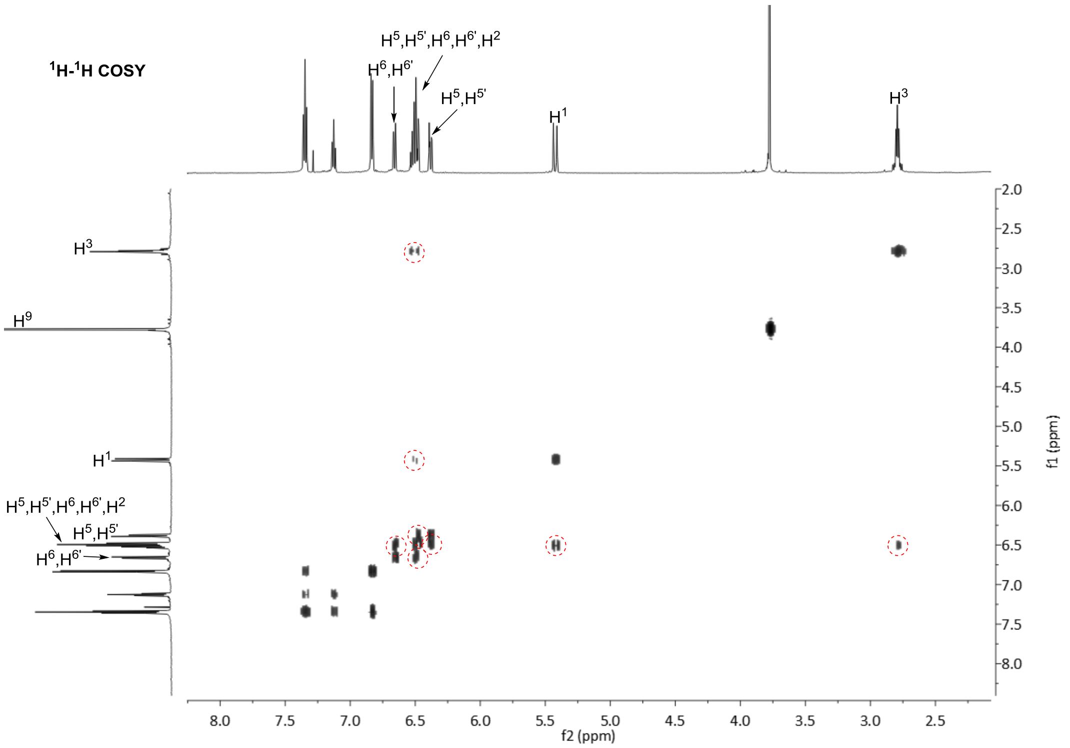

Supplementary Figure 7. ^{1}H-^{1}H COSY spectrum of **16a**

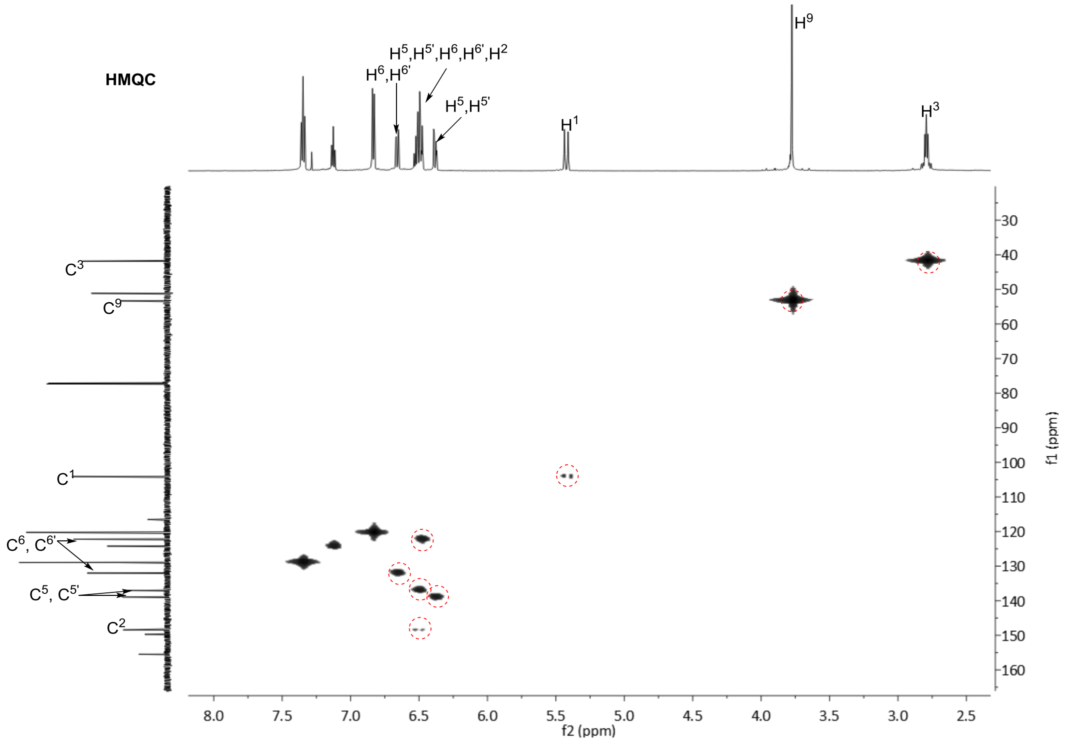

Supplementary Figure 8. HMQC spectrum of **16a**

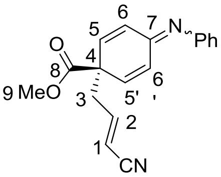

16a

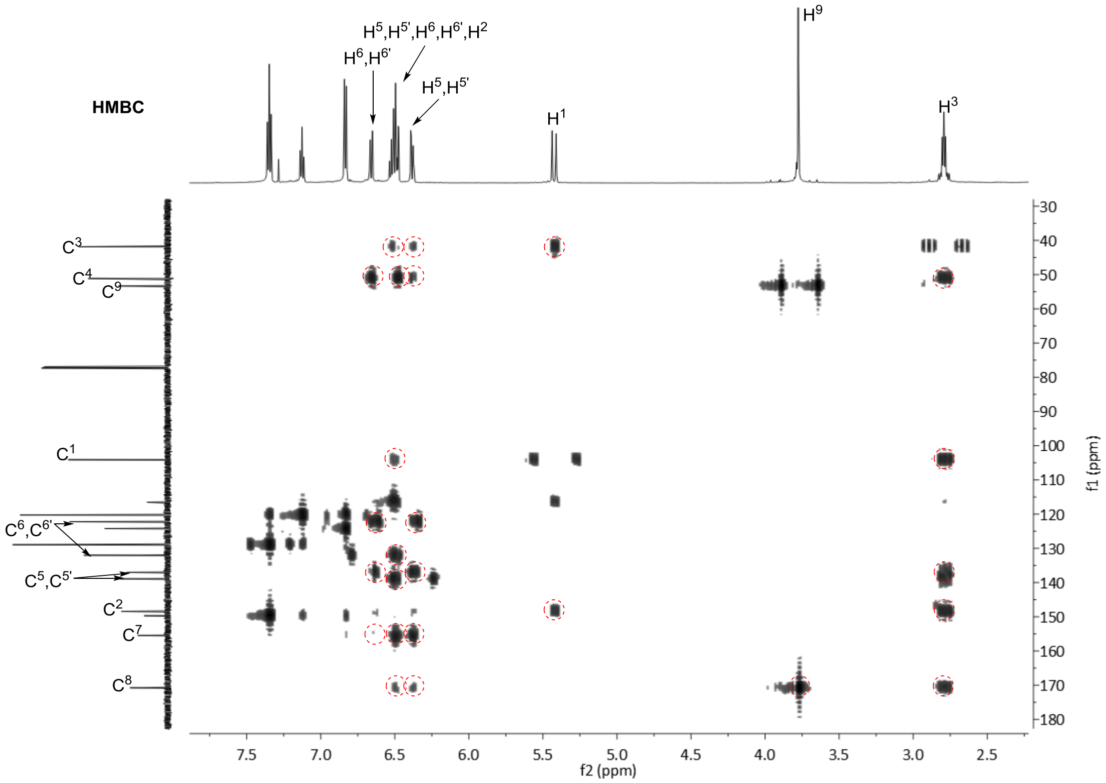

Supplementary Figure 9. HMBC spectrum of **16a**

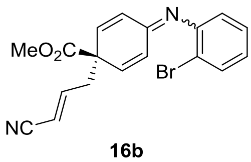

16b

Methyl 4-((2-bromophenyl)imino)-1-((*E*)-3-cyanoallyl)cyclohexa-2,5-dienecarboxylate (16b):

Following the general procedure, the title compound was obtained as yellow oil, 90.7 mg, 49% yield. (Rf = 0.10, eluent: PE/EtOAc = 5/1).

^{1}H NMR (600 MHz, $CDCl_3$): δ 7.58 (dd, *J* = 8.0, 1.1 Hz, 1H), 7.28 – 7.25 (m, 1H), 6.97 (td, *J* = 7.8, 1.5 Hz, 1H), 6.78 (dd, *J* = 7.8, 1.5 Hz, 1H), 6.73 (dd, *J* = 10.1, 1.8 Hz, 1H), 6.57 (dd, *J* = 10.1, 2.6 Hz, 1H), 6.52 – 6.44 (m, 1H), 6.42 (dd, *J* = 10.3, 2.6 Hz, 1H), 6.24 (dd, *J* = 10.3, 1.8 Hz, 1H), 5.40 (d, *J* = 16.2 Hz, 1H), 3.76 (s, 3H), 2.85 – 2.74 (m, 2H).

^{13}C NMR (151 MHz, $CDCl_3$): δ 170.6, 156.9, 148.4, 148.3, 139.8, 138.1, 133.1, 131.5, 128.0, 125.4, 122.6, 121.2, 116.5, 114.5, 104.4, 53.5, 51.4, 41.8.

IR (neat): 2920, 2851, 2223, 1730, 1658, 1588, 1459, 1433, 1232, 1023, 966, 832 cm^{-1}.

HRMS (ESI-TOF) calcd. for $C_{18}H_{15}BrN_2NaO_2$ $[M+Na]^+$: 393.0209, found: 393.0220.

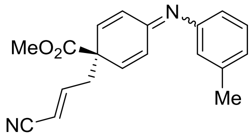

16c

Methyl 1-((*E*)-3-cyanoallyl)-4-(m-tolylimino)cyclohexa-2,5-dienecarboxylate (16c):

Following the general procedure, the title compound was obtained as yellow oil, 79.6 mg, 52% yield. (Rf = 0.40, eluent: PE/EtOAc = 2/1).

^{1}H NMR (600 MHz, $CDCl_3$): δ 7.23 – 7.17 (m, 1H), 6.92 (d, *J* = 7.5 Hz, 1H), 6.66 – 6.58 (m, 3H), 6.53 – 6.43 (m, 3H), 6.35 (dd, *J* = 10.4, 2.3 Hz, 1H), 5.40 (d, *J* = 16.2 Hz, 1H), 3.75 (s, 3H), 2.81 – 2.72 (m, 2H), 2.33 (s, 3H).

^{13}C NMR (151 MHz, $CDCl_3$): δ 170.9, 155.4, 149.8, 148.5, 138.9, 138.8, 136.9, 132.1, 128.8, 125.0, 122.4, 120.9, 117.3, 116.6, 104.2, 53.4, 51.2, 42.0, 21.5.

IR (neat): 2919, 2849, 2224, 1730, 1657, 1584, 1433, 1229, 1184, 967, 832, 779 cm^{-1}.

HRMS (ESI-TOF) calcd. for $C_{19}H_{18}N_2NaO_2$ $[M+Na]^+$: 329.1260, found: 329.1269.

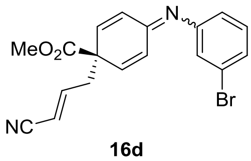

16d

Methyl 4-((3-bromophenyl)imino)-1-((*E*)-3-cyanoallyl)cyclohexa-2,5-dienecarboxylate (16d):

Following the general procedure, the title compound was obtained as colorless oil, 103.6 mg, 56% yield. (Rf = 0.24, eluent: PE/EtOAc = 5/1).

^{1}H NMR (600 MHz, $CDCl_3$): δ 7.23 (d, *J* = 8.0 Hz, 1H), 7.21 – 7.17 (m, 1H), 6.99 – 6.95 (m, 1H), 6.74 (d, *J* = 7.7 Hz, 1H), 6.61 (d, *J* = 10.3 Hz, 1H), 6.53 – 6.45 (m, 2H), 6.42 (s, 2H), 5.41 (d, *J* = 16.2 Hz, 1H), 3.76 (s, 3H), 2.82 – 2.73 (m, 2H).

^{13}C NMR (151 MHz, $CDCl_3$): δ 170.7, 156.1, 151.4, 148.3, 139.9, 137.8, 131.8, 130.5, 127.1, 123.1, 122.8, 122.1, 119.1, 116.6, 104.4, 53.5, 51.3, 42.0.

IR (neat): 2921, 2849, 2224, 1731, 1582, 1465, 1232, 968, 833 cm^{-1}.

HRMS (ESI-TOF) calcd. for $C_{18}H_{15}BrN_2NaO_2$ $[M+Na]^+$: 393.0209, found: 393.0210.

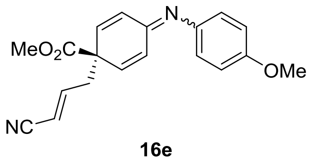

16e

Methyl 1-((*E*)-3-cyanoallyl)-4-((4-methoxyphenyl)imino)cyclohexa-2,5-dienecarboxylate (16e):

Following the general procedure, the title compound was obtained as yellow oil, 85.4 mg, 53% yield. (Rf = 0.32, eluent: PE/EtOAc = 2/1).

^{1}H NMR (600 MHz, $CDCl_3$): δ 6.89 – 6.85 (m, 2H), 6.82 – 6.76 (m, 2H), 6.62 (dd, *J* = 10.1, 1.7 Hz, 1H), 6.56 (dd, *J* = 10.4, 1.8 Hz, 1H), 6.52 – 6.42 (m, 2H), 6.35 (dd, *J* = 10.4, 2.6 Hz, 1H), 5.40 (d, *J* = 16.2 Hz, 1H), 3.79 (s, 3H), 3.75 (s, 3H), 2.80 – 2.72 (m, 2H).

^{13}C NMR (151 MHz, $CDCl_3$): δ 170.9, 156.9, 155.6, 148.6, 142.7, 138.7, 136.7, 132.2, 122.3, 121.9, 116.6, 114.3, 104.1, 55.6, 53.4, 51.3, 42.0.

IR (neat): 2952, 2835, 2223, 1729, 1657, 1602, 1499, 1235, 1104, 1030, 967, 830 cm^{-1}.

HRMS (ESI-TOF) calcd. for $C_{19}H_{18}N_2NaO_3$ $[M+Na]^+$: 345.1210, found: 345.1225.

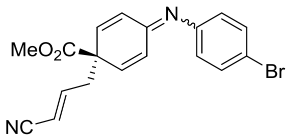

16f

Methyl 4-((4-bromophenyl)imino)-1-((*E*)-3-cyanoallyl)cyclohexa-2,5-diene carboxylate (16f):

Following the general procedure, the title compound was obtained as yellow oil, 109.2 mg, 59% yield. (Rf = 0.45, eluent: PE/EtOAc = 2/1).

^{1}H NMR (600 MHz, $CDCl_3$): δ 7.44 (d, *J* = 8.5 Hz, 2H), 6.70 (d, *J* = 8.5 Hz, 2H), 6.62 (dd, *J* = 10.1, 1.3 Hz, 1H), 6.53 – 6.38 (m, 4H), 5.41 (d, *J* = 16.2 Hz, 1H), 3.77 (s, 3H), 2.83 – 2.73 (m, 2H).

^{13}C NMR (151 MHz, $CDCl_3$): δ 170.7, 155.9, 148.9, 148.4, 139.6, 137.5, 132.1, 132.0, 122.2, 122.1, 117.4, 116.6, 104.4, 53.5, 51.3, 41.9.

IR (neat): 2953, 2224, 1729, 1684, 1590, 1488, 1212, 1069, 965, 832 cm^{-1}.

HRMS (ESI-TOF) calcd. for $C_{18}H_{15}BrN_2NaO_2$ $[M+Na]^+$: 393.0209, found: 393.0245.

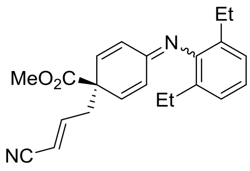

16g

Methyl 1-((*E*)-3-cyanoallyl)-4-((2,6-diethylphenyl)imino)cyclohexa-2,5-dienecarboxylate (16g):

Following the general procedure, the title compound was obtained as colorless oil, 73.1 mg, 42% yield. (Rf = 0.16, eluent: PE/EtOAc = 5/1).

^{1}H NMR (600 MHz, $CDCl_3$): δ 7.09 – 7.04 (m, 2H), 7.04 – 6.98 (m, 1H), 6.77 (d, *J* = 9.6 Hz, 1H), 6.50 (dd, *J* = 10.1, 2.5 Hz, 1H), 6.48 – 6.41 (m, 1H), 6.29 (dd, *J* = 10.3, 2.5 Hz, 1H), 6.09 (dd, *J* = 10.3, 1.7 Hz, 1H), 5.38 (d, *J* = 16.2 Hz, 1H), 3.76 (s, 3H), 2.84 – 2.70 (m, 2H), 2.40 – 2.22 (m, 4H), 1.15 – 1.04 (m, 6H).

^{13}C NMR (151 MHz, $CDCl_3$): δ 170.9, 156.1, 148.3, 146.6, 138.5, 136.9, 132.39, 132.32, 131.6, 126.1, 126.0, 123.9, 122.9, 116.4, 104.3, 53.4, 51.4, 41.8, 24.8, 24.7, 14.1, 14.0.

IR (neat): 2969, 2873, 2220, 1731, 1654, 1585, 1432, 1234, 1031, 967, 836 cm^{-1}.

HRMS (ESI-TOF) calcd. for $C_{22}H_{24}N_2NaO_2$ $[M+Na]^+$: 371.1730, found: 371.1735.

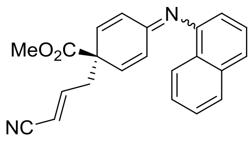

16h

Methyl 1-((*E*)-3-cyanoallyl)-4-(naphthalen-1-ylimino)cyclohexa-2,5-diene-1-carboxylate (16h):

Following the general procedure, the title compound was obtained as yellow oil, 99.2 mg, 58% yield. (Rf = 0.30, eluent: PE/EtOAc = 2/1).

^{1}H NMR (600 MHz, CDCl$_3$): δ 7.84 (d, *J* = 7.8 Hz, 1H), 7.76 (d, *J* = 8.0 Hz, 1H), 7.62 (d, *J* = 8.2 Hz, 1H), 7.53 – 7.45 (m, 2H), 7.45 – 7.40 (m, 1H), 6.83 (d, *J* = 10.2 Hz, 1H), 6.78 (d, *J* = 7.2 Hz, 1H), 6.56 (dd, *J* = 10.1, 1.7 Hz, 1H), 6.54 – 6.47 (m, 1H), 6.38 – 6.26 (m, 2H), 5.40 (d, *J* = 16.2 Hz, 1H), 3.77 (s, 3H), 2.84 – 2.73 (m, 2H).

^{13}C NMR (151 MHz, CDCl$_3$): δ 170.9, 156.6, 148.5, 146.5, 139.0, 137.3, 134.2, 132.1, 128.0, 126.6, 126.5, 126.0, 125.7, 124.4, 123.8, 122.8, 116.6, 114.4, 104.3, 53.5, 51.3, 41.9.

IR (neat): 2954, 2926, 2225, 1733, 1659, 1591, 1506, 1434, 1385, 1234, 1015, 968 cm^{-1}.

HRMS (ESI-TOF) calcd. for $C_{22}H_{19}N_2O_2$ [M+H]$^+$: 343.1441, found: 343.1435.

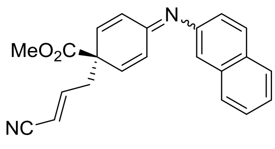

16i

Methyl 1-((*E*)-3-cyanoallyl)-4-(naphthalen-2-ylimino)cyclohexa-2,5-dienecarboxylate (16i):

Following the general procedure, the title compound was obtained as yellow solid, m.p. 84 – 87 ℃, 104.4 mg, 61% yield. (Rf = 0.27, eluent: PE/EtOAc = 2/1).

^{1}H NMR (600 MHz, CDCl$_3$): δ 7.85 – 7.79 (m, 2H), 7.77 (d, *J* = 8.1 Hz, 1H), 7.49 – 7.44 (m, 1H), 7.43 – 7.38 (m, 1H), 7.18 (s, 1H), 7.06 (d, *J* = 8.5 Hz, 1H), 6.69 (d, *J* = 10.1 Hz, 1H), 6.55 – 6.46 (m, 3H), 6.36 (d, *J* = 10.3 Hz, 1H), 5.41 (d, *J* = 16.2 Hz, 1H), 3.77 (s, 3H), 2.84 – 2.72 (m, 2H).

^{13}C NMR (151 MHz, CDCl$_3$): δ 170.8, 155.8, 148.5, 147.6, 139.1, 137.2, 133.9, 132.1, 130.9, 129.0, 127.8, 127.5, 126.6, 124.9, 122.3, 121.4, 116.6, 116.2, 104.2, 53.4, 51.3, 41.9.

IR (neat): 3052, 2945, 2219, 1728, 1591, 1577, 1500, 1428, 1227, 1082, 968 cm^{-1}.

HRMS (ESI-TOF) calcd. for $C_{22}H_{18}N_2NaO_2$ [M+Na]$^+$: 365.1260, found: 365.1269.

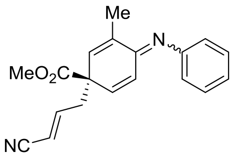

16j

Methyl 1-((*E*)-3-cyanoallyl)-3-methyl-4-(phenylimino)cyclohexa-2,5-diene-1-carboxylate (16j):

Following the general procedure, **16j** were obtained from the reaction of **1j** with **2a** and **11a**. The title compound was obtained as yellow oil, 64.3 mg, 42% yield. (Rf = 0.23, eluent: PE/EtOAc = 5/1).

^{1}H NMR (600 MHz, $CDCl_3$): δ 7.35 – 7.30 (m, 2H), 7.11 – 7.06 (m, 1H), 6.82 – 6.73 (m, 2H), 6.53 – 6.45 (m, 1H), 6.42 (d, *J* = 10.8 Hz, 1H), 6.35 – 6.28 (m, 2H), 5.43 – 5.35 (m, 1H), 3.75 (s, 3H), 2.82 – 2.71 (m, 2H), 2.14 – 2.07 (m, 3H).

^{13}C NMR (151 MHz, $CDCl_3$): δ 171.4, 156.2, 150.6, 149.1, 138.2, 137.7, 133.5, 129.0, 123.8, 122.4, 120.1, 116.8, 103.9, 53.3, 51.0, 42.1, 18.4.

IR (neat): 2957, 2225, 1733, 1663, 1585, 1500, 1483, 1434, 1231, 1123, 968, 865 cm^{-1}.

HRMS (ESI-TOF) calcd. for $C_{19}H_{19}N_2O_2$ $[M+H]^+$: 307.1441, found: 307.1441.

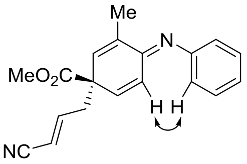

16j 2D-NOESY

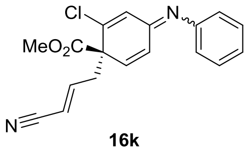

16k
(*E*/*Z* = 55/45)

Methyl (*S*,*E*)-2-chloro-1-((*E*)-3-cyanoallyl)-4-(phenylimino)cyclohexa-2,5-diene-1-carboxylate (16k):

Following a procedure similar to the general procedure, the reaction of **1l** with **2a** and **11a** was conducted under modified conditions (Nu, -95 ℃, 12 h), **16k** were obtained as a mixture of two stereoisomers (E/Z = 55/45), yellow oil, 63.6 mg, 39% yield. (Rf = 0.42, eluent: PE/EtOAc = 2/1).

^{1}H NMR (600 MHz, $CDCl_3$): δ 7.41 – 7.31 (m, 2H), 7.18 – 7.10 (m, 1H), 6.90 – 6.77 (m, 2.45H), 6.70 – 6.64 (m, 1.1H), 6.52 (d, *J* = 9.9 Hz, 0.45H), 6.46 – 6.35 (m, 1H), 6.28 (d, *J* = 9.8 Hz, 0.55H), 6.13 (d, *J* = 10.0 Hz, 0.45H), 5.47 (d, *J* = 16.2 Hz, 1H), 3.78 (s, 3H), 3.20 – 3.05 (m, 1H), 3.02 – 2.88 (m, 1H).

^{13}C NMR (151 MHz, CDCl$_3$): δ 168.83, 168.81, 155.7, 155.1, 149.6, 149.4, 147.9, 145.3, 141.7, 137.7, 136.0, 132.5, 132.3, 129.3, 129.1, 124.8, 124.7, 122.9, 122.3, 120.28, 120.25, 116.7, 104.5, 57.0, 56.8, 53.9, 38.7, 38.6.

IR (neat): 2921, 2226, 1743, 1651, 1590, 1482, 1434, 1233, 1025, 970, 910 cm^{-1}.

HRMS (ESI-TOF) calcd. for $C_{18}H_{16}ClN_2O_2$ $[M+H]^+$: 327.0895, found: 327.0895.

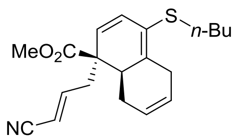

17a

Methyl (1*S*,8a*S*)-4-(butylthio)-1-((*E*)-3-cyanoallyl)-1,5,8,8a-tetrahydronaphthalene-1-carboxylate (17a):

Following a procedure similar to the general procedure, buta-1,3-diene (**12a**, 5.0 equiv) dissovled in THF (1.25 mL) was used as nucleophile. The title compound was obtained as yellow oil, 111.6 mg, 65% yield. (Rf = 0.28, eluent: PE/EtOAc = 10/1).

^{1}H NMR (600 MHz, CDCl$_3$): δ 6.58 (dt, *J* = 15.8, 7.7 Hz, 1H), 6.08 (d, *J* = 9.9 Hz, 1H), 5.85 (d, *J* = 9.9 Hz, 1H), 5.69 – 5.63 (m, 1H), 5.60 – 5.54 (m, 1H), 5.30 (d, *J* = 16.2 Hz, 1H), 3.73 (s, 3H), 3.61 – 3.54 (m, 1H), 2.82 – 2.76 (m, 1H), 2.73 – 2.68 (m, 1H), 2.65 – 2.57 (m, 2H), 2.57 – 2.49 (m, 2H), 2.23 – 2.14 (m, 1H), 1.77 – 1.70 (m, 1H), 1.49 – 1.43 (m, 2H), 1.41 – 1.34 (m, 2H), 0.87 (t, *J* = 7.3 Hz, 3H).

^{13}C NMR (151 MHz, CDCl$_3$): δ 173.7, 151.0, 141.6, 127.4, 126.5, 126.3, 124.2, 120.1, 117.1, 102.8, 52.3, 50.1, 46.3, 42.5, 33.8, 33.0, 31.9, 29.8, 21.8, 13.8.

IR (neat): 3031, 2961, 2910, 2222, 1727, 1636, 1590, 1438, 1263, 1218, 1143, 1050, 968 cm^{-1}.

HRMS (ESI-TOF) calcd. for $C_{20}H_{25}NNaO_2S$ $[M+Na]^+$: 366.1498, found: 366.1493.

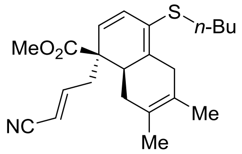

17b

Methyl (1*S*,8a*S*)-4-(butylthio)-1-((*E*)-3-cyanoallyl)-6,7-dimethyl-1,5,8,8a-tetrahydronaphthalene-1-carboxylate (17b):

Following the general procedure, the title compound was obtained as colorless oil, 120.8 mg, 65% yield. (Rf = 0.34, eluent: PE/EtOAc = 10/1).

^{1}H NMR (600 MHz, CDCl$_3$): δ 6.59 (dt, *J* = 15.8, 7.8 Hz, 1H), 6.07 (d, *J* = 9.9 Hz, 1H), 5.82 (d, *J* = 9.9 Hz, 1H), 5.31 (d, *J* = 16.2 Hz, 1H), 3.75 (s, 3H), 3.40 (d, *J* = 17.5 Hz, 1H), 2.79 – 2.67 (m,

2H), 2.66 – 2.50 (m, 4H), 2.22 – 2.15 (m, 1H), 1.65 (s, 3H), 1.62 – 1.58 (m, 1H), 1.54 (s, 3H), 1.47 – 1.41 (m, 2H), 1.40 – 1.33 (m, 2H), 0.86 (t, J = 7.2 Hz, 3H).

^{13}C NMR (151 MHz, CDCl$_3$): δ 173.9, 151.2, 142.8, 127.6, 125.6, 125.3, 123.8, 118.9, 117.1, 102.7, 52.4, 50.0, 46.7, 42.7, 39.2, 35.5, 33.1, 31.9, 21.8, 18.9, 18.8, 13.9.

IR (neat): 2956, 2929, 2872, 2225, 1725, 1634, 1600, 1435, 1293, 1260, 1219, 1114, 973 cm^{-1}.

HRMS (ESI-TOF) calcd. for $C_{22}H_{29}NNaO_2S$ [M+Na]$^+$: 394.1811, found: 394.1805.

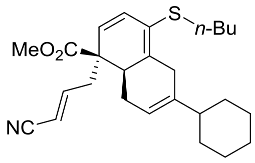

17c

Methyl (1*S*,8a*S*)-4-(butylthio)-1-((*E*)-3-cyanoallyl)-6-cyclohexyl-1,5,8,8a-tetrahydronaphthalene-1-carboxylate (17c):

Following the general procedure, the title compound was obtained as yellow oil, 142.6 mg, 67% yield. (Rf = 0.33, eluent: PE/EtOAc = 10/1).

^{1}H NMR (600 MHz, CDCl$_3$): δ 6.58 (dt, J = 15.9, 7.7 Hz, 1H), 6.06 (d, J = 9.9 Hz, 1H), 5.81 (d, J = 9.9 Hz, 1H), 5.34 – 5.28 (m, 1H), 5.28 – 5.24 (m, 1H), 3.73 (s, 3H), 3.51 (d, J = 17.4 Hz, 1H), 2.78 – 2.68 (m, 2H), 2.64 – 2.55 (m, 2H), 2.54 – 2.47 (m, 2H), 2.19 – 2.10 (m, 1H), 1.86 – 1.79 (m, 1H), 1.78 – 1.64 (m, 6H), 1.50 – 1.41 (m, 2H), 1.41 – 1.33 (m, 2H), 1.30 – 1.21 (m, 2H), 1.20 – 1.10 (m, 3H), 0.87 (t, J = 7.3 Hz, 3H).

^{13}C NMR (151 MHz, CDCl$_3$): δ 173.8, 151.1, 143.3, 142.7, 127.4, 123.9, 119.4, 117.9, 117.1, 102.7, 52.3, 50.1, 46.7, 45.0, 42.6, 35.1, 33.0, 32.3, 32.1, 31.9, 29.2, 26.81, 26.76, 26.4, 21.9, 13.9.

IR (neat): 2926, 2852, 2226, 1727, 1634, 1436, 1261, 1222, 1196, 1110, 906 cm^{-1}.

HRMS (ESI-TOF) calcd. for $C_{26}H_{35}NNaO_2S$ [M+Na]$^+$: 448.2281, found: 448.2268.

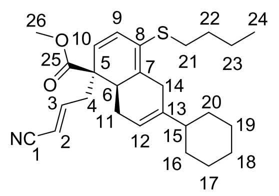

17c

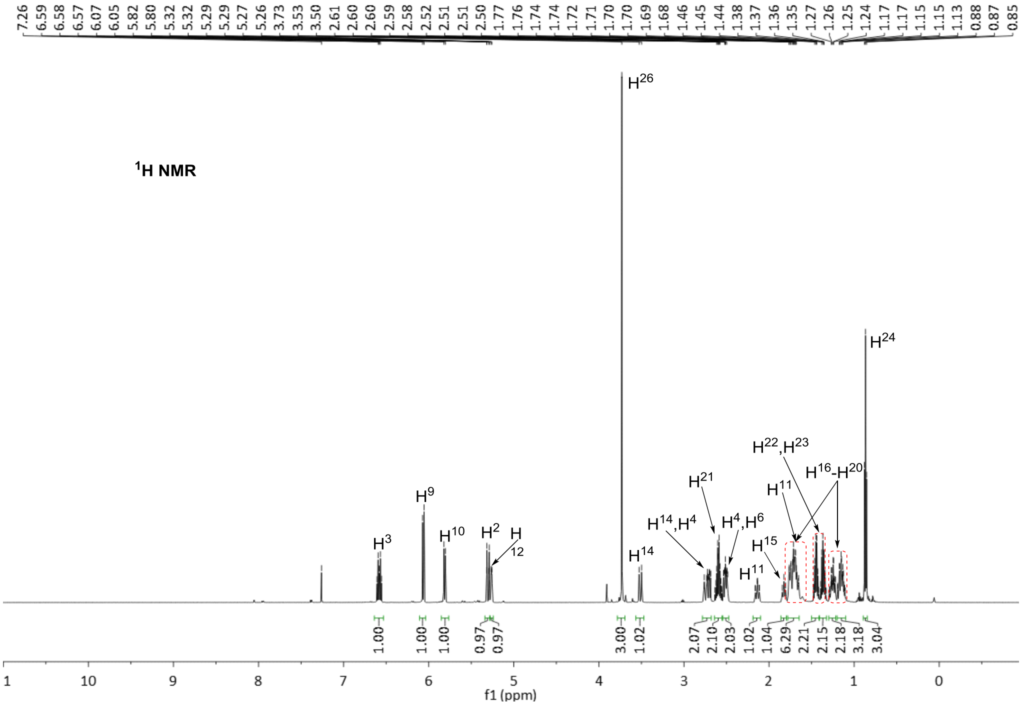

Supplementary Figure 10. ^{1}H NMR spectrum of **17c**

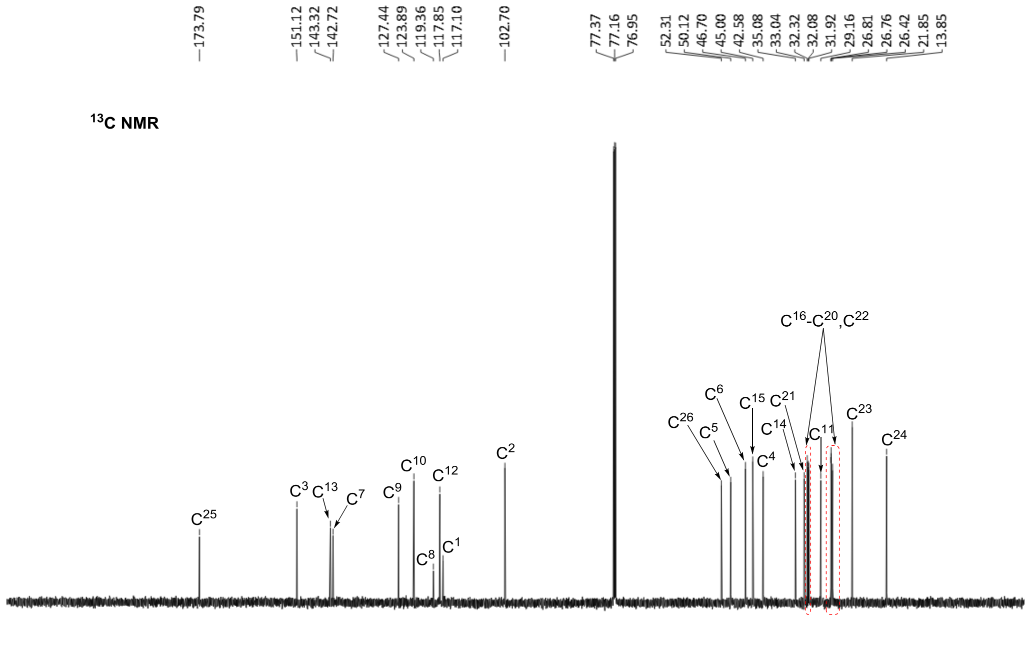

Supplementary Figure 11. ^{13}C NMR spectrum of **17c**

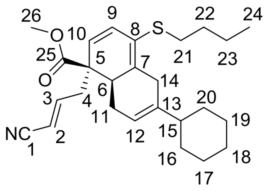

17c

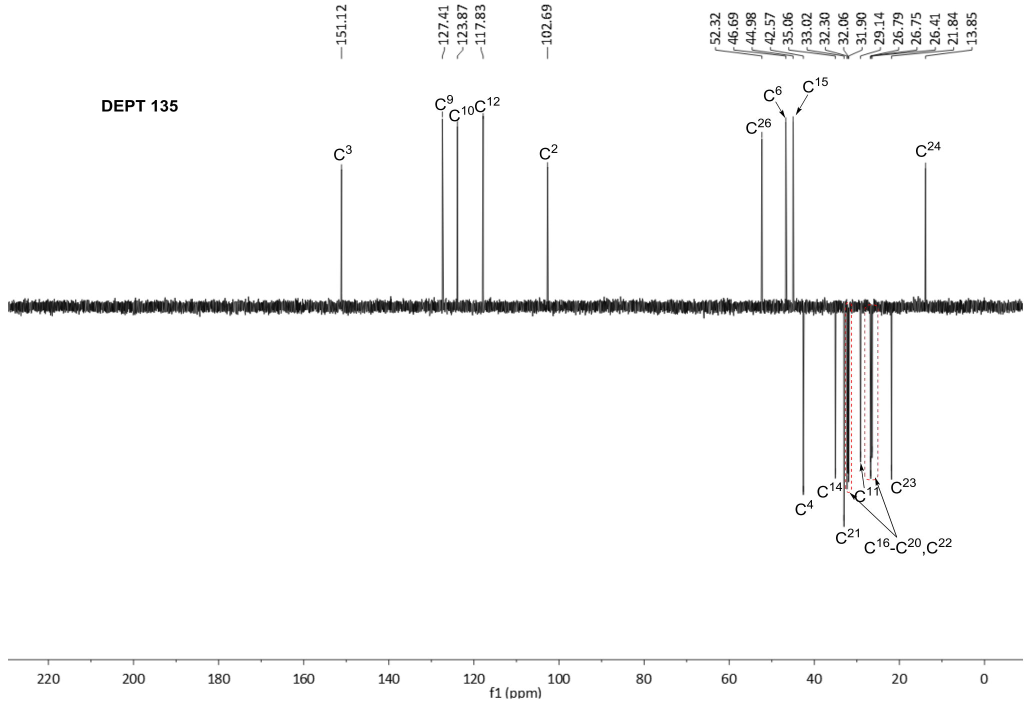

Supplementary Figure 12. DEPT 135 spectrum of **17c**

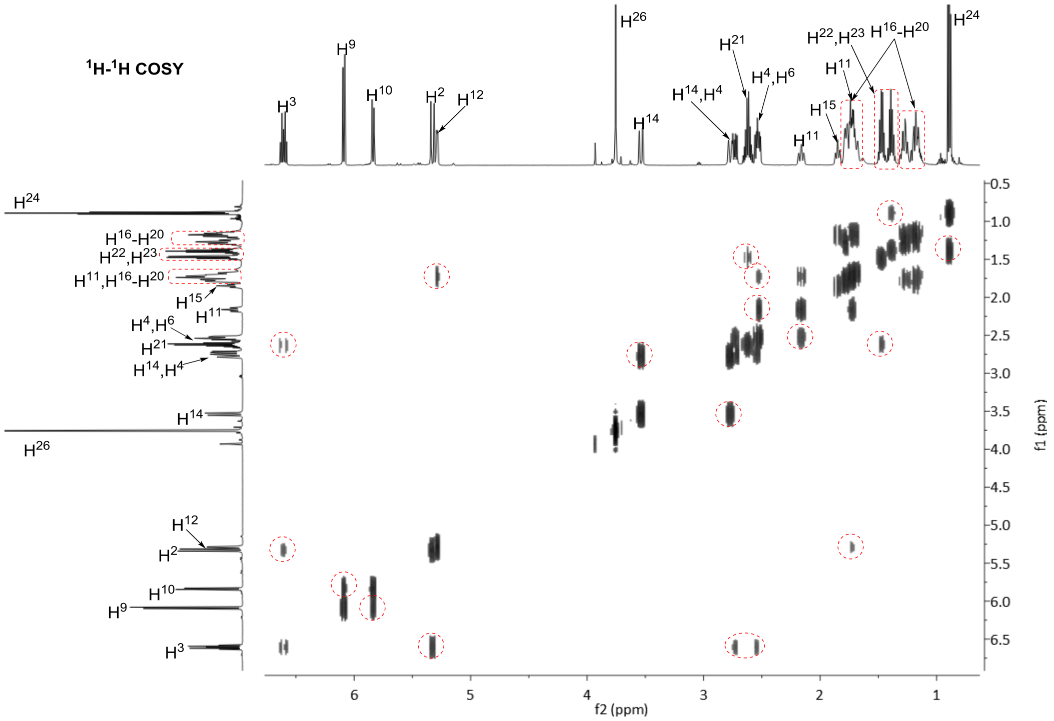

Supplementary Figure 13. ^{1}H-^{1}H COSY spectrum of **17c**

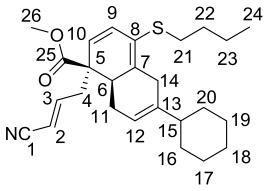

17c

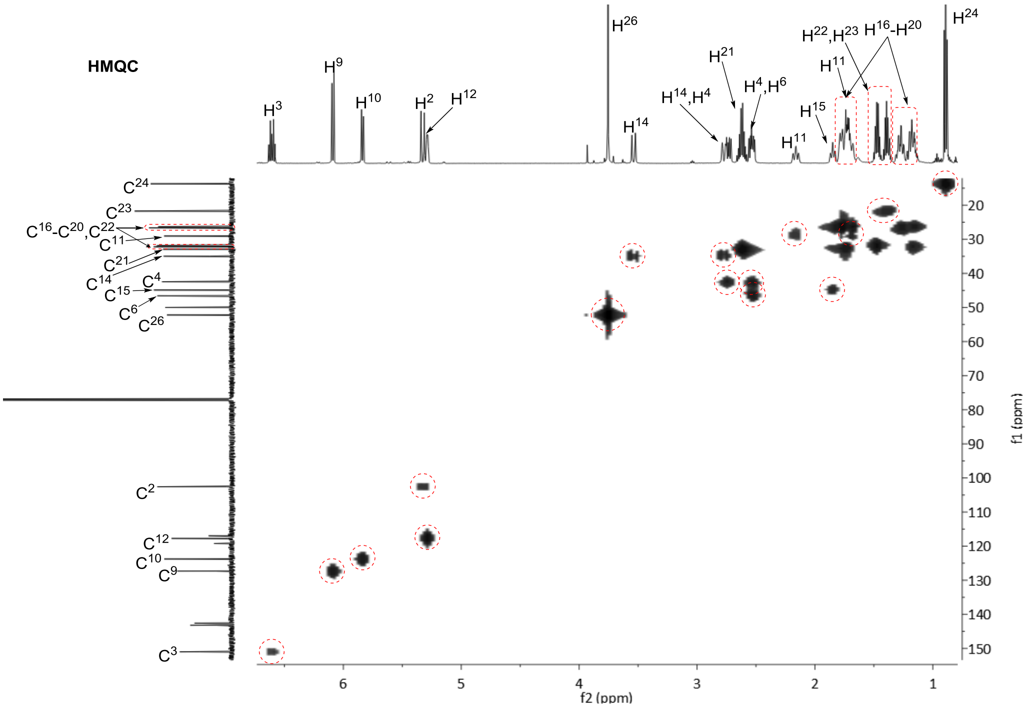

Supplementary Figure 14. HMQC spectrum of **17c**

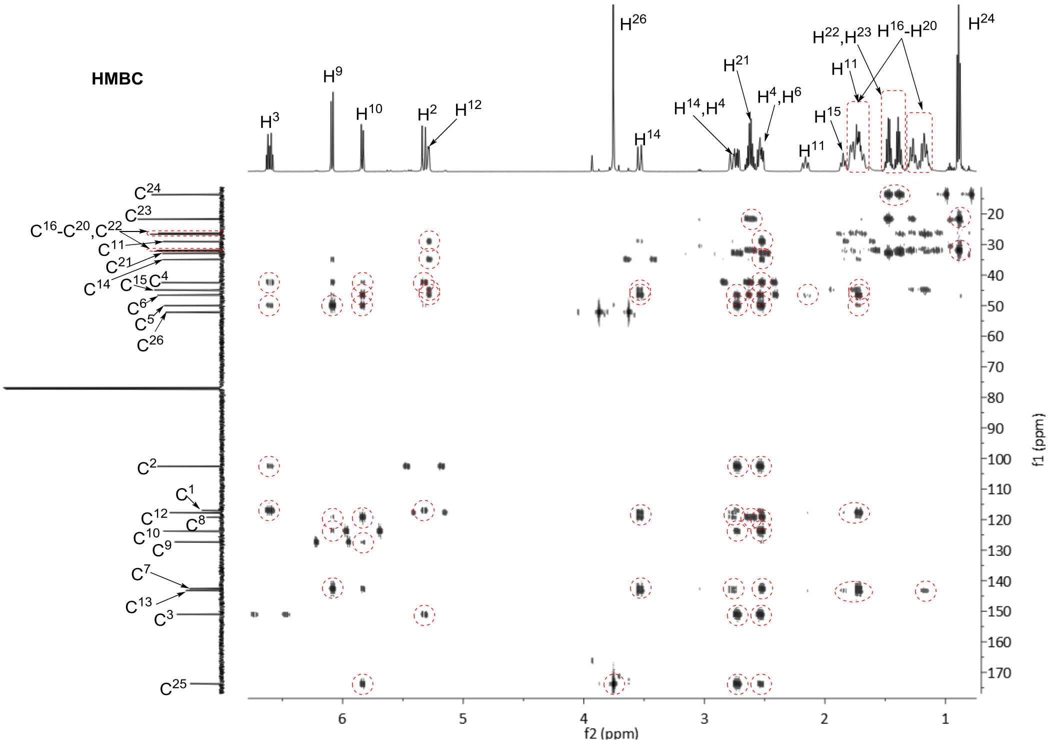

Supplementary Figure 15. HMBC spectrum of **17c**

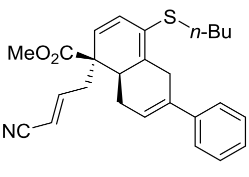

17d

Methyl (1*S*,8a*S*)-4-(butylthio)-1-((*E*)-3-cyanoallyl)-6-phenyl-1,5,8,8a-tetrahydronaphthalene-1-carboxylate (17d):

Following the general procedure, the title compound was obtained as yellow oil, 77.6 mg, 37% yield. (Rf = 0.33, eluent: PE/EtOAc = 10/1).

^{1}H NMR (600 MHz, CDCl$_3$): δ 7.28 – 7.22 (m, 4H), 7.22 – 7.17 (m, 1H), 6.58 (dt, *J* = 15.8, 7.7 Hz, 1H), 6.12 (d, *J* = 9.9 Hz, 1H), 6.05 – 5.99 (m, 1H), 5.90 (d, *J* = 9.9 Hz, 1H), 5.30 (d, *J* = 16.2 Hz, 1H), 3.78 – 3.69 (m, 4H), 2.95 (d, *J* = 18.8 Hz, 1H), 2.77 – 2.71 (m, 1H), 2.71 – 2.66 (m, 1H), 2.65 – 2.57 (m, 3H), 2.57 – 2.52 (m, 1H), 2.12 – 2.05 (m, 1H), 1.49 – 1.42 (m, 2H), 1.39 – 1.32 (m, 2H), 0.83 (t, *J* = 7.3 Hz, 3H).

^{13}C NMR (151 MHz, CDCl$_3$): δ 173.7, 150.9, 141.3, 140.8, 136.9, 128.5, 127.4, 127.2, 125.2, 124.4, 123.5, 120.5, 117.0, 102.9, 52.5, 50.2, 45.9, 42.6, 33.6, 33.0, 32.1, 32.0, 21.8, 13.8.

IR (neat): 2955, 2913, 2225, 1730, 1634, 1494, 1435, 1259, 1197, 1107, 969, 906 cm^{-1}.

HRMS (ESI-TOF) calcd. for $C_{26}H_{29}NNaO_2S$ [M+Na]$^+$: 442.1811, found: 442.1799.

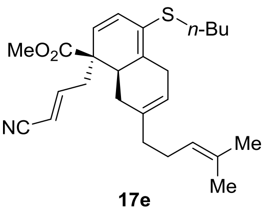

17e

Methyl (1*S*,8a*S*)-4-(butylthio)-1-((*E*)-3-cyanoallyl)-7-(4-methylpent-3-en-1-yl)-1,5,8,8a-tetrahydronaphthalene-1-carboxylate (17e):

Following the general procedure, the title compound was obtained as yellow oil, 131.9 mg, 62% yield. (Rf = 0.34, eluent: PE/EtOAc = 10/1).

^{1}H NMR (600 MHz, CDCl$_3$): δ 6.59 (dt, *J* = 15.8, 7.7 Hz, 1H), 6.08 (d, *J* = 9.9 Hz, 1H), 5.83 (d, *J* = 9.8 Hz, 1H), 5.34 – 5.28 (m, 2H), 5.13 – 5.06 (m, 1H), 3.74 (s, 3H), 3.49 (d, *J* = 17.3 Hz, 1H), 2.79 – 2.69 (m, 2H), 2.65 – 2.58 (m, 2H), 2.55 – 2.49 (m, 2H), 2.19 – 2.07 (m, 3H), 2.03 – 1.97 (m, 2H), 1.73 – 1.66 (m, 4H), 1.63 – 1.58 (s, 3H), 1.49 – 1.43 (m, 2H), 1.41 – 1.35 (m, 2H), 0.88 (t, *J* = 7.3 Hz, 3H).

^{13}C NMR (151 MHz, CDCl$_3$): δ 173.8, 151.1, 142.1, 138.0, 132.0, 127.5, 124.0, 119.9, 119.7, 117.1, 102.8, 52.3, 50.1, 46.6, 42.6, 37.0, 36.8, 33.1, 32.0, 29.2, 26.7, 25.9, 21.8, 17.9, 13.8.

IR (neat): 2956, 2926, 2872, 2224, 1727, 1633, 1600, 1435, 1291, 1261, 1195, 1108, 970 cm^{-1}.

HRMS (ESI-TOF) calcd. for $C_{26}H_{35}NNaO_2S$ [M+Na]$^+$: 448.2281, found: 448.2267.

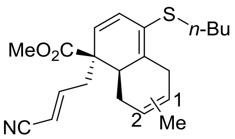

17f
C(1)/C(2) = 77/23

Methyl (1*S*,8a*S*)-4-(butylthio)-1-((*E*)-3-cyanoallyl)-6-methyl-1,5,8,8a-tetrahydronaphthalene-1-carboxylate (major):

Methyl (1*S*,8a*S*)-4-(butylthio)-1-((*E*)-3-cyanoallyl)-7-methyl-1,5,8,8a-tetrahydronaphthalene-1-carboxylate (minor):

Following the general procedure, the title compound was obtained as a mixture of two regiosiomers (**C(1)**/**C(2)** = 77/23), yellow oil, 119.7 mg, 67% yield. (Rf = 0.29, eluent: PE/EtOAc = 10/1).

^{1}H NMR (600 MHz, CDCl$_3$): δ 6.62 – 6.54 (m, 1H), 6.12 – 6.02 (m, 1H), 5.88 – 5.79 (m, 1H), 5.39 – 5.23 (m, 2H), 3.75 (s, 0.69H), 3.72 (s, 2.31H), 3.54 – 3.39 (m, 1H), 2.79 – 2.67 (m, 2H), 2.65 – 2.47 (m, 4H), 2.21 – 2.06 (m, 1H), 1.72 – 1.58 (m, 4H), 1.49 – 1.41 (m, 2H), 1.40 – 1.33 (m, 2H), 0.90 – 0.83 (m, 3H),

^{13}C NMR (151 MHz, CDCl$_3$): δ 173.75, 173.73, 151.06, 151.04, 141.96, 141.90, 134.1, 133.7, 127.46, 127.43, 124.02, 120.4, 120.3, 119.8, 119.6, 117.1, 102.74, 102.72, 52.34, 52.29, 50.11, 50.05, 46.36, 45.9, 42.6, 38.3, 34.3, 33.2, 33.04, 33.00, 31.9, 31.8, 29.1, 23.1, 22.8, 21.75, 21.72, 13.80, 13.78.

IR (neat): 2956, 2928, 2872, 2224, 1729, 1633, 1435, 1378, 1260, 1222, 1195, 1108, 971 cm^{-1}.

HRMS (ESI-TOF) calcd. for $C_{21}H_{27}NNaO_2S$ [M+Na]$^+$: 380.1655, found: 380.1643.

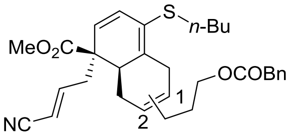

17g
C(1)/C(2) = 89/11

Methyl (1*S*,8a*S*)-4-(butylthio)-1-((*E*)-3-cyanoallyl)-6-(3-(2-phenylacetoxy)propyl)-1,5,8,8a-tetrahydronaphthalene-1-carboxylate (major):

Methyl (1*S*,8a*S*)-4-(butylthio)-1-((*E*)-3-cyanoallyl)-7-(3-(2-phenylacetoxy)propyl)-1,5,8,8a-tetrahydronaphthalene-1-carboxylate (minor):

Following the general procedure, the title compound was obtained as a mixture of two regiosiomers (**C(1)/C(2)** = 89/11), yellow oil, 182.0 mg, 68% yield. (Rf = 0.31, eluent: PE/EtOAc = 5/1).

^{1}H NMR (400 MHz, CDCl$_3$): δ 7.42 – 7.30 (m, 5H), 6.63 – 6.52 (m, 1H), 6.12 – 6.02 (m, 1H), 5.89 – 5.79 (m, 1H), 5.42 – 5.25 (m, 2H), 5.18 – 5.12 (m, 2H), 4.18 – 4.06 (m, 2H), 3.77 – 3.69 (m, 3H), 3.57 – 3.43 (m, 1H), 2.81 – 2.65 (m, 2H), 2.65 – 2.44 (m, 4H), 2.21 – 2.02 (m, 2.67H), 2.01 – 1.94 (m, 0.33H), 1.85 – 1.60 (m, 3H), 1.50 – 1.31 (m, 4H), 0.90 – 0.82 (m, 3H).

^{13}C NMR (101 MHz, CDCl$_3$): δ 173.6, 155.3, 155.2, 150.9, 141.6, 141.5, 136.7, 136.3, 135.3, 128.68, 128.61, 128.4, 127.4, 127.2, 124.1, 120.8, 120.7, 119.93, 119.87, 117.0, 102.7, 69.6, 67.7, 67.7, 52.34, 52.30, 50.08, 50.02, 46.3, 45.8, 42.5, 36.4, 33.1, 32.94, 32.91, 32.6, 31.91, 31.85, 29.0, 26.7, 21.7, 13.8.

IR (neat): 2955, 2873, 2361, 2223, 1732, 1634, 1456, 1435, 1398, 1254, 1107, 969 cm^{-1}.

HRMS (ESI-TOF) calcd. for $C_{31}H_{37}NNaO_5S$ [M+Na]$^+$: 558.2285, found: 558.2280.

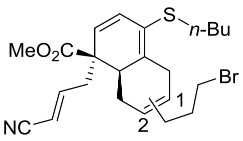

17h

C(1)/C(2) = 84/16

Methyl (1*S*,8a*S*)-6-(3-bromopropyl)-4-(butylthio)-1-((*E*)-3-cyanoallyl)-1,5,8,8a-tetrahydronaphthalene-1-carboxylate (major):

Methyl (1*S*,8a*S*)-7-(3-bromopropyl)-4-(butylthio)-1-((*E*)-3-cyanoallyl)-1,5,8,8a-tetrahydronaphthalene-1-carboxylate (minor):

Following the general procedure, the title compound was obtained as a mixture of two regiosiomers (**C(1)/C(2)** = 84/16), yellow oil, 151.0 mg, 65% yield. (Rf = 0.29, eluent: PE/EtOAc = 10/1).

^{1}H NMR (600 MHz, CDCl$_3$): δ 6.62 – 6.54 (m, 1H), 6.11 – 6.04 (m, 1H), 5.88 – 5.80 (m, 1H), 5.44 – 5.42 (m, 0.16H), 5.40 – 5.26 (m, 1.84H), 3.75 (s, 0.48H), 3.73 (s, 2.52H), 3.56 – 3.45 (m, 1H), 3.41 – 3.29 (m, 2H), 2.80 – 2.67 (m, 2H), 2.65 – 2.56 (m, 2H), 2.55 – 2.46 (m, 2H), 2.19 – 2.02 (m, 3H), 2.01 – 1.81 (m, 2H), 1.74 – 1.66 (m, 0.84H), 1.57 – 1.53 (m, 0.16H), 1.48 – 1.40 (m, 2H), 1.40 – 1.32 (m, 2H), 0.90 – 0.84 (m, 3H).

^{13}C NMR (151 MHz, CDCl$_3$): δ 173.7, 151.0, 150.9, 141.6, 141.3, 136.2, 135.9, 127.4, 127.2, 124.14, 124.10, 121.25, 121.24, 119.94, 119.90, 117.04, 117.02, 102.81, 102.78, 52.41, 52.36, 50.09, 50.03, 46.3, 45.8, 42.6, 36.4, 35.4, 35.1, 33.34, 33.29, 33.2, 32.9, 32.7, 32.0, 31.9, 30.72, 30.67, 29.1, 21.8, 21.7, 13.8.

IR (neat): 2988, 2971, 2900, 2361, 2343, 2225, 1733, 1406, 1382, 1250, 1066, 902 cm^{-1}.

HRMS (ESI-TOF) calcd. for $C_{23}H_{30}BrNNaO_2S$ [M+Na]$^+$: 486.1073, found: 486.1057.

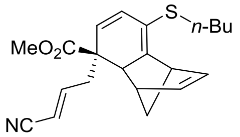

17i

Methyl (1*R*,4*S*,4a*S*,5*S*)-8-(butylthio)-5-((*E*)-3-cyanoallyl)-1,4,4a,5-tetrahydro-1,4-methanonaphthalene-5-carboxylate (17i):

Following the general procedure, the title compound was obtained as white solid, m.p. 84 – 87 ℃, 131.5 mg, 74% yield. (Rf = 0.25, eluent: PE/EtOAc = 10/1).

^{1}H NMR (400 MHz, CDCl$_3$): δ 6.76 – 6.65 (m, 1H), 6.20 – 6.14 (m, 1H), 6.09 (d, *J* = 9.6 Hz, 1H), 5.89 – 5.84 (m, 1H), 5.45 (d, *J* = 16.3 Hz, 1H), 5.19 (d, *J* = 9.6 Hz, 1H), 3.72 – 3.67 (m, 1H), 3.52 (s, 3H), 3.08 – 3.04 (m, 1H), 3.04 – 2.97 (m, 1H), 2.97 – 2.94 (m, 1H), 2.71 – 2.62 (m, 1H), 2.54 – 2.44 (m, 2H), 1.84 (d, *J* = 8.2 Hz, 1H), 1.55 – 1.35 (m, 5H), 0.91 (t, *J* = 7.1 Hz, 3H).

^{13}C NMR (101 MHz, CDCl$_3$): δ 173.1, 151.8, 148.4, 136.2, 134.8, 132.3, 123.6, 117.1, 115.9, 103.4, 54.7, 52.1, 50.6, 49.2, 47.7, 43.0, 41.9, 33.0, 31.8, 21.7, 13.8.

IR (neat): 2955, 2872, 2223, 1721, 1635, 1598, 1434, 1248, 1225, 1022, 976 cm^{-1}.

HRMS (ESI-TOF) calcd. for $C_{21}H_{25}NNaO_2S$ [M+Na]$^+$: 378.1498, found: 378.1487.

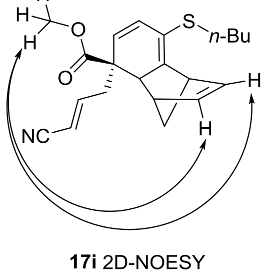

17i 2D-NOESY

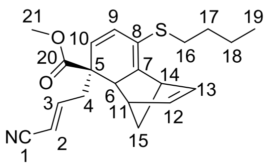

17i

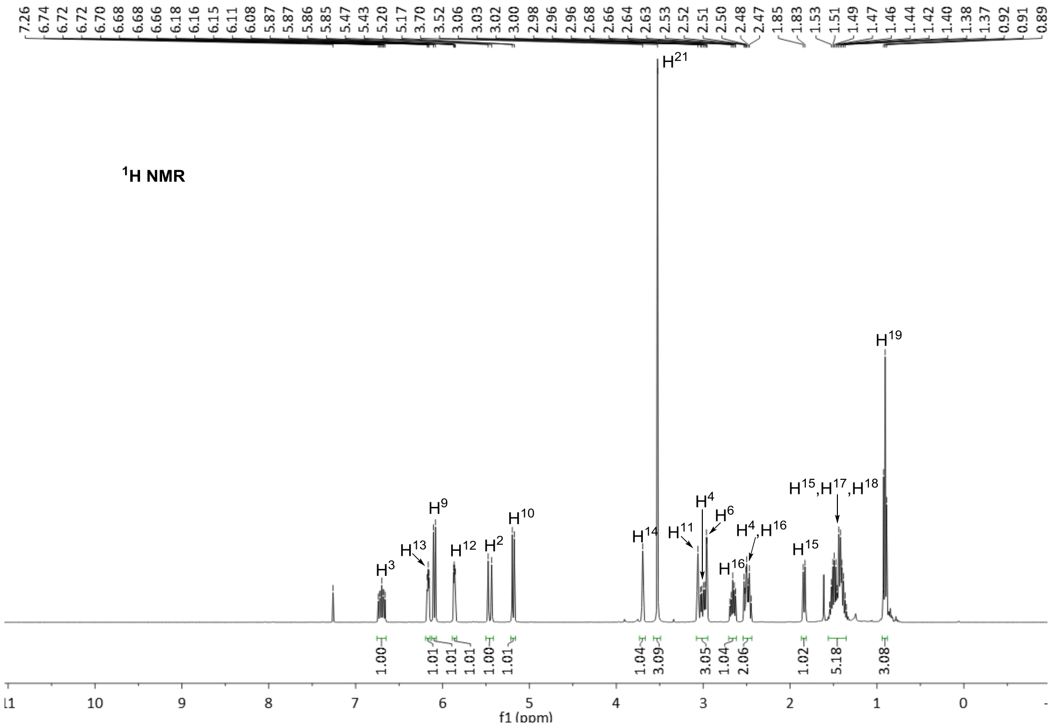

Supplementary Figure 16. ^{1}H NMR spectrum of **17i**

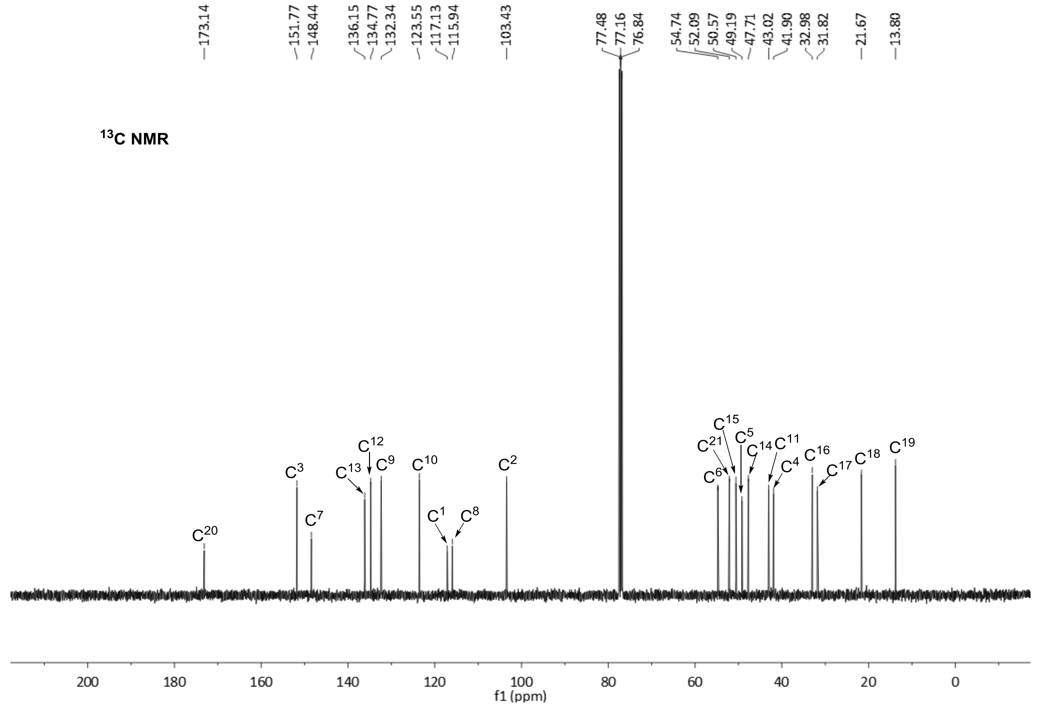

Supplementary Figure 17. ^{13}C NMR spectrum of **17i**

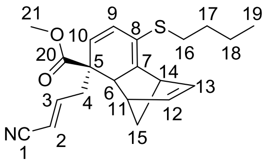

17i

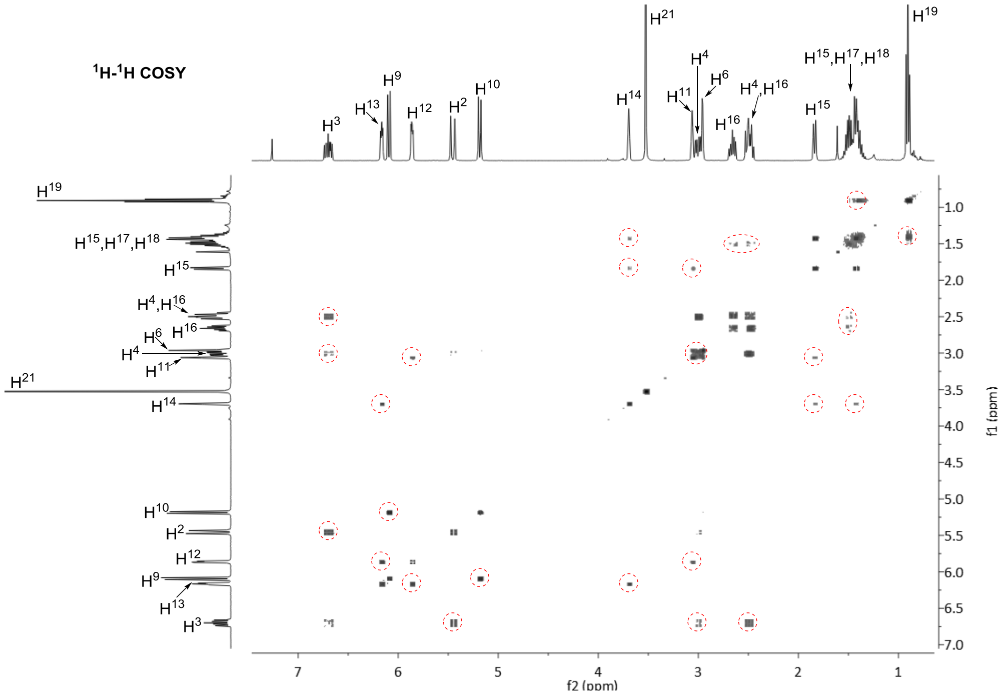

Supplementary Figure 18. ^{1}H-^{1}H COSY spectrum of **17i**

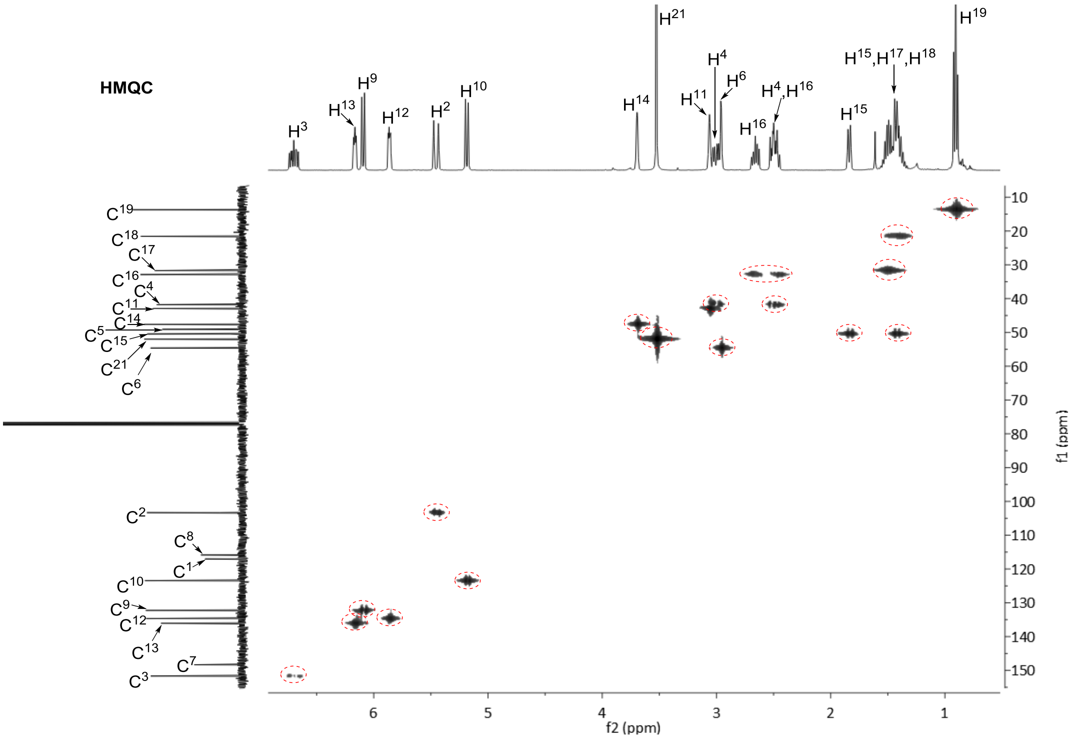

Supplementary Figure 19. HMQC spectrum of **17i**

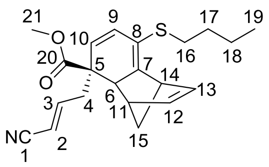

17i

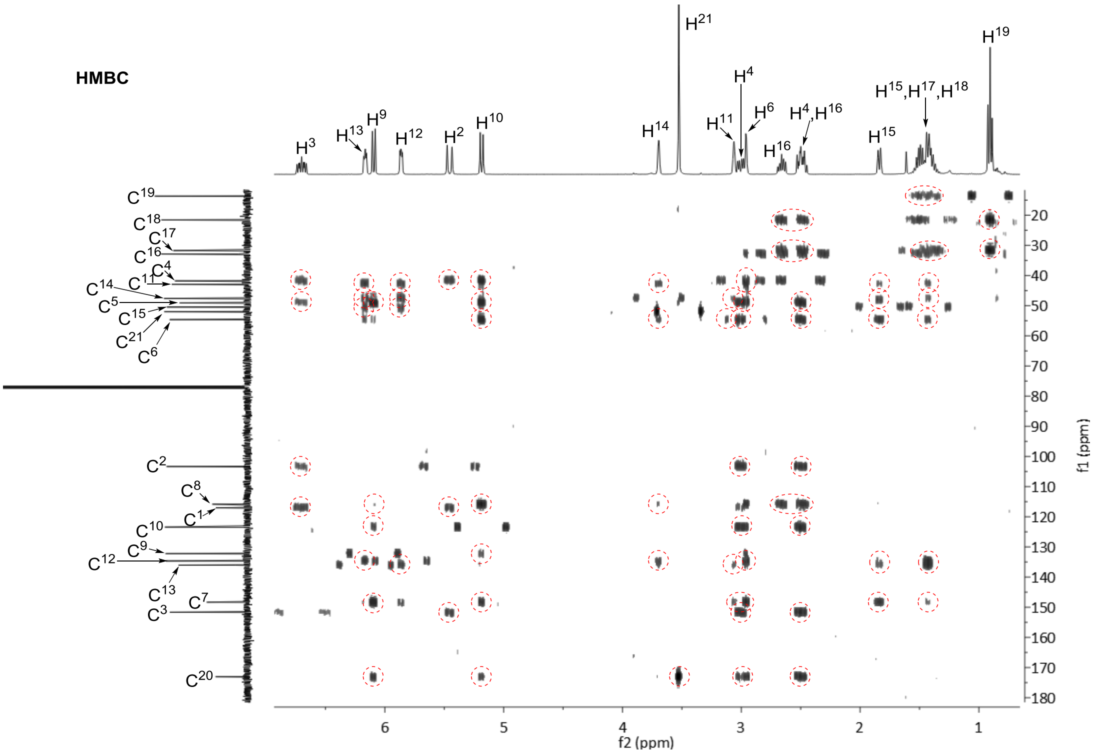

Supplementary Figure 20. HMBC spectrum of **17i**

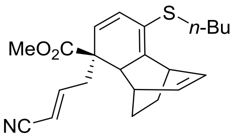

17j

Methyl (1*R*,4*S*,4a*S*,5*S*)-8-(butylthio)-5-((*E*)-3-cyanoallyl)-1,4,4a,5-tetrahydro-1,4-ethanonaphthalene-5-carboxylate (17j):

Following the general procedure, the title compound was obtained as white solid, m.p. 72 – 74 ℃, 107.9 mg, 60% yield. (Rf = 0.23, eluent: PE/EtOAc = 10/1).

^{1}H NMR (600 MHz, $CDCl_3$): δ 6.71 – 6.62 (m, 1H), 6.28 – 6.21 (m, 1H), 6.10 (d, *J* = 9.7 Hz, 1H), 6.06 – 5.98 (m, 1H), 5.45 (d, *J* = 16.3 Hz, 1H), 5.24 (d, *J* = 9.7 Hz, 1H), 3.90 (d, *J* = 6.6 Hz, 1H), 3.53 (s, 3H), 2.99 – 2.91 (m, 1H), 2.81 (d, *J* = 5.7 Hz, 1H), 2.77 (s, 1H), 2.67 – 2.60 (m, 1H), 2.54 – 2.44 (m, 2H), 1.61 – 1.47 (m, 4H), 1.47 – 1.35 (m, 4H), 0.91 (t, *J* = 7.3 Hz, 3H).

^{13}C NMR (151 MHz, CDCl$_3$): δ 172.6, 151.9, 147.6, 133.8, 132.9, 132.4, 123.9, 117.1, 116.1, 103.3, 52.7, 52.2, 48.7, 40.6, 36.5, 33.3, 31.7, 31.5, 27.6, 22.9, 21.7, 13.8.

IR (neat): 2956, 2915, 2848, 2223, 1721, 1632, 1473, 1434, 1251, 1220, 1184, 997 cm^{-1}.

HRMS (ESI-TOF) calcd. for $C_{22}H_{27}NNaO_2S$ [M+Na]$^+$: 392.1655, found: 392.1648.

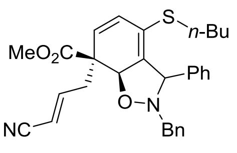

18a

Methyl (7*S*,7a*S*)-2-benzyl-4-(butylthio)-7-((*E*)-3-cyanoallyl)-3-phenyl-2,3,7,7a-tetrahydrobenzo[d]isoxazole-7-carboxylate (18a): Following the general procedure, the title compound was obtained as yellow oil, 150.1 mg, 60% yield. (Rf = 0.31, eluent: PE/EtOAc = 5/1).

^{1}H NMR (400 MHz, CDCl$_3$): δ 7.40 – 7.22 (m, 10H), 6.78 – 6.66 (m, 1H), 6.24 (d, *J* = 9.5 Hz, 1H), 5.58 (d, *J* = 9.5 Hz, 1H), 5.40 (d, *J* = 16.4 Hz, 1H), 4.94 (s, 1H), 4.79 (s, 1H), 4.15 – 3.97 (m, 2H), 3.43 (s, 3H), 2.99 – 2.88 (m, 1H), 2.74 – 2.64 (m, 1H), 2.58 – 2.39 (m, 2H), 1.31 – 1.15 (m, 4H), 0.80 (t, *J* = 7.0 Hz, 3H).

^{13}C NMR (101 MHz, CDCl$_3$): δ 169.7, 150.7, 144.5, 138.9, 136.6, 131.6, 129.2, 129.0, 128.5, 128.0, 127.7, 127.5, 127.4, 122.4, 117.0, 103.7, 82.2, 70.1, 52.5, 50.3, 40.0, 32.1, 31.9, 22.7, 21.8, 13.7.

IR (neat): 2956, 2928, 2361, 2223, 1731, 1675, 1636, 1496, 1454, 1434, 1226, 1076, 971 cm^{-1}.

HRMS (ESI-TOF) calcd. for $C_{30}H_{33}N_2O_3S$ [M+H]$^+$]: 501.2206, found: 501.2208.

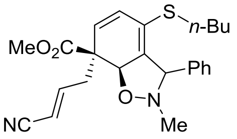

18b
(84/16 dr)

Methyl (7*S*,7a*S*)-4-(butylthio)-7-((*E*)-3-cyanoallyl)-2-methyl-3-phenyl-2,3,7,7a-tetrahydrobenzo[d]isoxazole-7-carboxylate (18b): Following the general procedure, the title compound was obtained as a mixture of two diastereoisomers (84/16 dr), yellow oil, 114.6 mg, 54% yield. (Rf = 0.17, eluent: PE/EtOAc = 5/1).

^{1}H NMR (400 MHz, CDCl$_3$): δ 7.51 – 7.16 (m, 5H), 6.84 – 6.63 (m, 1H), 6.20 (d, *J* = 9.6 Hz, 0.84H), 6.09 (d, *J* = 12.4 Hz, 0.16H), 5.82 (d, *J* = 7.3 Hz, 0.16H), 5.61 – 5.42 (m, 1.84H), 4.96 – 4.88 (m, 0.84H), 4.75 – 4.35 (m, 0.84H), 4.29 – 4.21 (m, 0.16H), 3.82 (d, *J* = 7.6 Hz, 0.16H), 3.67

– 3.42 (m, 3H), 3.21 – 3.14 (m, 0.16H), 3.08 – 2.98 (m, 0.84H), 2.85 – 2.62 (m, 3.36H), 2.56 (s, 0.48H), 2.53 – 2.27 (m, 2H), 2.22 – 2.15 (m, 0.16H), 1.31 – 1.04 (m, 4H), 0.83 – 0.72 (m, 3H).

^{13}C NMR (101 MHz, CDCl$_3$): δ 169.67, 168.68, 151.3, 150.9, 150.7, 139.9, 131.5, 129.02, 128.95, 128.5, 128.4, 128.1, 127.70, 127.68, 127.0, 126.4, 124.3, 122.6, 117.0, 103.8, 103.2, 82.0, 75.7, 66.7, 65.8, 54.9, 52.6, 52.4, 50.2, 41.9, 40.0, 36.5, 32.1, 31.9, 31.4, 30.0, 22.0, 21.8, 13.7, 13.6.

IR (neat): 2956, 2929, 2871, 2223, 1730, 1634, 1455, 1434, 1275, 1226, 1205, 1031, 971 cm^{-1}.

HRMS (ESI-TOF) calcd. for $C_{24}H_{29}N_2O_3S$ [M+H]$^+$: 425.1893, found: 425.1895.

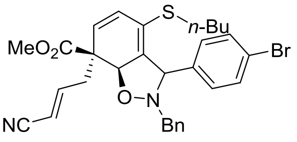

18c

Methyl (7*S*,7a*S*)-2-benzyl-3-(4-bromophenyl)-4-(butylthio)-7-((*E*)-3-cyanoallyl)-2,3,7,7a-tetrahydrobenzo[*d*]isoxazole-7-carboxylate (18c): Following the general procedure, the title compound was obtained as yellow oil, 162.3 mg, 56% yield. (Rf = 0.20, eluent: PE/EtOAc = 5/1).

^{1}H NMR (400 MHz, CDCl$_3$): δ 7.37 (d, *J* = 8.3 Hz, 2H), 7.33 – 7.19 (m, 7H), 6.74 – 6.63 (m, 1H), 6.21 (d, *J* = 9.5 Hz, 1H), 5.57 (d, *J* = 9.5 Hz, 1H), 5.39 (d, *J* = 16.3 Hz, 1H), 4.92 (s, 1H), 4.70 (s, 1H), 4.12 – 4.01 (m, 1H), 4.00 – 3.92 (m, 1H), 3.42 (s, 3H), 2.97 – 2.85 (m, 1H), 2.73 – 2.62 (m, 1H), 2.57 – 2.38 (m, 2H), 1.30 – 1.12 (m, 4H), 0.79 (t, *J* = 6.9 Hz, 3H).

^{13}C NMR (101 MHz, CDCl$_3$): δ 169.6, 150.5, 143.6, 138.0, 136.2, 131.3, 131.0, 130.7, 129.2, 128.6, 127.8, 127.6, 122.8, 121.5, 117.0, 103.8, 82.1, 69.3, 52.6, 50.2, 40.0, 32.1, 32.0, 27.0, 21.8, 13.8.

IR (neat): 2954, 2928, 2359, 2223, 1727, 1634, 1486, 1454, 1433, 1224, 1181, 1071, 1011 cm^{-1}.

HRMS (ESI-TOF) calcd. for $C_{30}H_{32}BrN_2O_3S$ [M+H]$^+$: 579.1312, found: 579.1310.

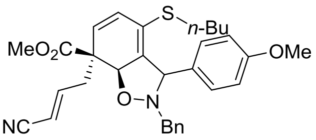

18d
(85/15 dr)

Methyl (7*S*,7a*S*)-2-benzyl-4-(butylthio)-7-((*E*)-3-cyanoallyl)-3-(4-methoxyphenyl)-2,3,7,7a-tetrahydrobenzo[*d*]isoxazole-7-carboxylate (18d):

Following the general procedure, the title compound was obtained as a mixture of two diastereoisomers (85/15 dr), yellow oil, 84.9 mg, 32% yield. (Rf = 0.12, eluent: PE/EtOAc = 5/1).

^{1}H NMR (400 MHz, CDCl$_3$): δ 7.35 – 7.22 (m, 7H), 6.87 (d, *J* = 8.7 Hz, 0.30H), 6.81 (d, *J* = 8.8 Hz, 1.70H), 6.78 – 6.64 (m, 1H), 6.23 (d, *J* = 9.6 Hz, 0.85H), 6.11 (d, *J* = 9.5 Hz, 0.15H), 5.57 (d, *J* = 9.5 Hz, 0.85H), 5.48 – 5.37 (m, 1.15H), 4.96 – 4.86 (m, 1H), 4.72 (s, 0.85H), 4.38 (s, 0.15H), 4.14 – 3.94 (m, 2H), 3.81 (s, 0.45H), 3.78 (s, 2.55H), 3.59 (s, 0.45H), 3.48 (s, 2.55H), 2.98 – 2.90 (m, 0.85H), 2.82 – 2.75 (m, 0.30H), 2.72 – 2.64 (m, 0.85H), 2.60 – 2.32 (m, 2H), 1.33 – 1.18 (m, 4H), 0.87 – 0.78 (m, 3H).

^{13}C NMR (101 MHz, CDCl$_3$): δ 169.9, 169.8, 159.5, 159.1, 151.1, 150.8, 136.7, 131.6, 130.8, 130.5, 130.3, 130.2, 129.9, 129.2, 128.4, 128.3, 127.6, 127.5, 127.2, 126.5, 122.3, 117.2, 117.0, 113.9, 113.4, 103.6, 103.2, 82.6, 82.4, 69.7, 59.3, 55.4, 52.6, 52.5, 50.3, 49.9, 40.7, 40.0, 32.4, 32.1, 32.0, 31.5, 29.8, 21.83, 21.77, 13.7.

IR (neat): 2955, 2929, 2358, 2224, 1730, 1611, 1512, 1456, 1434, 1246, 1178, 1031, 911 cm^{-1}.

HRMS (ESI-TOF) calcd. for $C_{31}H_{34}N_2NaO_4S$ $[M+Na]^+$: 553.2131, found: 553.2140.

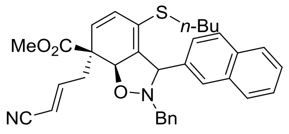

18e

Methyl (7*S*,7a*S*)-2-benzyl-4-(butylthio)-7-((*E*)-3-cyanoallyl)-3-(naphthalen-2-yl)-2,3,7,7a-tetrahydrobenzo[d]isoxazole-7-carboxylate (18e): Following the general procedure, the title compound was obtained as yellow oil, 168.0 mg, 56% yield. (Rf = 0.32, eluent: PE/EtOAc = 5/1).

^{1}H NMR (400 MHz, CDCl$_3$): δ 7.85 – 7.73 (m, 4H), 7.54 – 7.42 (m, 3H), 7.38 – 7.26 (m, 5H), 6.80 – 6.67 (m, 1H), 6.25 (d, *J* = 9.5 Hz, 1H), 5.60 (d, *J* = 9.5 Hz, 1H), 5.43 (d, *J* = 16.3 Hz, 1H), 5.05 – 4.85 (m, 2H), 4.19 – 4.02 (m, 2H), 3.39 (s, 3H), 3.02 – 2.91 (m, 1H), 2.77 – 2.67 (m, 1H), 2.52 – 2.34 (m, 2H), 1.23 – 1.14 (m, 1H), 1.07 – 0.98 (m, 2H), 0.90 – 0.84 (m, 1H), 0.60 (t, *J* = 7.1 Hz, 3H).

^{13}C NMR (101 MHz, CDCl$_3$): δ 169.8, 150.7, 133.2, 133.0, 131.6, 129.2, 128.5, 128.1, 127.8, 127.7, 127.6, 127.5, 127.0, 126.0, 125.9, 122.8, 117.0, 103.7, 82.3, 70.2, 52.7, 50.3, 40.0, 32.2, 32.0, 27.0, 21.7, 13.5.

IR (neat): 2954, 2928, 2359, 2223, 1727, 1675, 1632, 1497, 1508, 1433, 1225, 968 cm^{-1}.

HRMS (ESI-TOF) calcd. for $C_{34}H_{34}N_2NaO_3S$ $[M+Na]^+$: 573.2182, found: 573.2180.

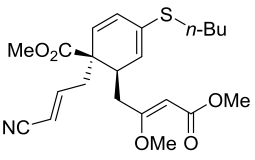

4d'

Methyl (1*S*,6*R*)-4-(butylthio)-1-((*E*)-3-cyanoallyl)-6-((*Z*)-2,4-dimethoxy-4-oxobut-2-en-1-yl)cyclohexa-2,4-diene-1-carboxylate (4d'):

Following the general procedure, the title compound was obtained as yellow oil, 102.8 mg, 49% yield. (Rf = 0.30, eluent: PE/EtOAc = 5/1).

^{1}H NMR (400 MHz, CDCl$_3$): δ 6.58 (dt, *J* = 15.8, 7.7 Hz, 1H), 6.07 (d, *J* = 9.9 Hz, 1H), 5.96 (d, *J* = 9.8 Hz, 1H), 5.39 (d, *J* = 6.2 Hz, 1H), 5.30 (d, *J* = 16.2 Hz, 1H), 5.01 (s, 1H), 3.75 (s, 3H), 3.64 (s, 3H), 3.59 (s, 3H), 3.19 – 3.09 (m, 1H), 2.88 – 2.76 (m, 2H), 2.68 – 2.60 (m, 2H), 2.55 – 2.43 (m, 2H), 1.60 – 1.51 (m, 2H), 1.46 – 1.36 (m, 2H), 0.91 (t, *J* = 7.3 Hz, 3H).

^{13}C NMR (101 MHz, CDCl$_3$): δ 173.6, 173.0, 167.6, 151.5, 130.6, 127.9, 126.4, 120.1, 117.1, 102.7, 92.3, 55.6, 52.5, 51.1, 50.9, 40.9, 40.4, 31.2, 31.0, 30.9, 22.1, 13.8.

IR (neat): 2954, 2224, 1710, 1621, 1435, 1375, 1275, 1192, 1134, 1052, 970, 823 cm^{-1}.

HRMS (ESI-TOF) calcd. for $C_{22}H_{29}NNaO_5S$ $[M+Na]^+$: 442.1659, found: 442.1652.

6 General procedure for the one pot synthesis of cyclohexenones

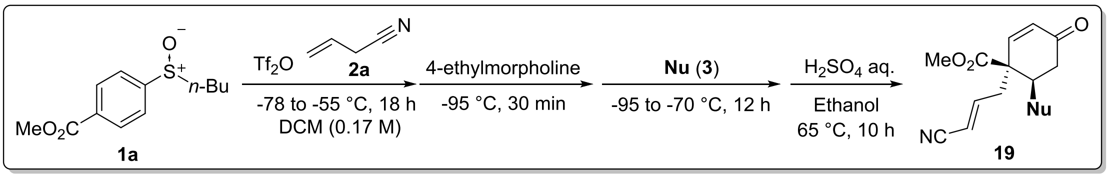

To a mixture of aryl sulfoxide **1a** (120 mg, 0.5 mmol) and allyl nitrile **2a** (121 µL, 1.5 mmol) in DCM (3.0 mL) was added Tf_2O (126 µL, 0.75 mmol) at -78 ℃ under N_2 atmosphere. The mixture was gradually warmed to -55 ℃. After stirring for 18 h, the mixture was cooled to -95 ℃. A mixture of 4-ethylmorpholine (157 µL, 1.25 mmol) and nucleophile **3** (1.0 mmol) in DCM (2.0 mL) was added to the mixture dropwise in 20 min using syringe pump. After stirring for 30 min, the mixture was gradually warmed to -70 ℃ and then stirred for 12 h. After that, to the mixture was added a mixture of H_2SO_4 aq. (1 M, 3 ml) and EtOH (3 mL). Then the resulted mixture was stirrred at 65 ℃ for 10 h. (Air was purged into the reactor to remove in situ formed butanethiol.) After cooling to rt, the mixture was diluted with EtOAc and washed with $NaHCO_3$ aq. and brine. The organic layer was separated, dried over Na_2SO_4 and concentrated. The obtained residue was further purified by flash chromatography on silica gel affording **19**.

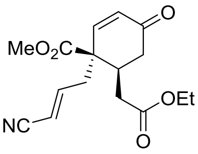

19a

Methyl (1*R*,6*S*)-1-((*E*)-3-cyanoallyl)-6-(2-ethoxy-2-oxoethyl)-4-oxocyclohex-2-ene-1-carboxylate (19a):

Following the general procedure, the title compound was obtained as colorless oil, 88.5 mg, 58% yield. (Rf = 0.19, eluent: PE/EtOAc = 2/1).

^{1}H NMR (600 MHz, $CDCl_3$): δ 6.76 (d, *J* = 10.3 Hz, 1H), 6.67 – 6.54 (m, 1H), 6.15 (d, *J* = 10.2 Hz, 1H), 5.48 (d, *J* = 16.2 Hz, 1H), 4.13 (q, *J* = 6.9 Hz, 2H), 3.76 (s, 3H), 2.97 – 2.88 (m, 1H), 2.72 (d, *J* = 9.8 Hz, 1H), 2.69 – 2.50 (m, 3H), 2.48 – 2.42 (m, 1H), 2.24 – 2.16 (m, 1H), 1.25 (t, *J* = 7.1 Hz, 3H).

^{13}C NMR (151 MHz, $CDCl_3$): δ 196.5, 171.14, 174.05, 148.8, 147.1, 130.9, 116.5, 104.8, 61.3, 53.1, 50.9, 40.4, 39.5, 37.9, 36.0, 14.3.

IR (neat): 2955, 2224, 1725, 1680, 1221, 1197, 1025, 969, 914 cm^{-1}.

HRMS (ESI-TOF) calcd. for $C_{16}H_{19}NNaO_5$ $[M+Na]^+$: 328.1155, found: 328.1158.

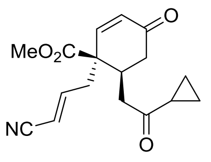

19b

Methyl (1*R*,6*S*)-1-((*E*)-3-cyanoallyl)-6-(2-cyclopropyl-2-oxoethyl)-4-oxocyclohex-2-ene-1-carboxylate (19b):

Following the general procedure, the title compound was obtained as yellow solid, m.p. 70 – 73 ℃, 105.4 mg, 70% yield. (Rf = 0.17, eluent: PE/EtOAc = 2/1).

^{1}H NMR (600 MHz, CDCl$_3$): δ 6.79 (d, *J* = 10.3 Hz, 1H), 6.65 – 6.56 (m, 1H), 6.13 (d, *J* = 10.3 Hz, 1H), 5.45 (d, *J* = 16.2 Hz, 1H), 3.77 (s, 3H), 2.93 – 2.84 (m, 2H), 2.70 – 2.58 (m, 2H), 2.57 – 2.50 (m, 2H), 2.47 – 2.39 (m, 1H), 1.89 – 1.79 (m, 1H), 1.08 – 0.98 (m, 2H), 0.93 – 0.87 (m, 2H).

^{13}C NMR (151 MHz, CDCl$_3$): δ 207.6, 196.9, 171.5, 149.0, 147.3, 130.8, 116.5, 104.6, 53.1, 50.9, 44.5, 40.7, 39.8, 36.4, 21.3, 11.6, 11.4.

IR (neat): 2953, 2920, 2220, 1731, 1677, 1391, 1223, 1199, 1062, 968, 806 cm^{-1}.

HRMS (ESI-TOF) calcd. for $C_{17}H_{19}NNaO_4$ $[M+Na]^+$: 324.1206, found: 324.1208.

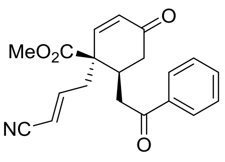

19c

Methyl (1*R*,6*S*)-1-((*E*)-3-cyanoallyl)-4-oxo-6-(2-oxo-2-phenylethyl)cyclohex-2-ene carboxylate (19c):

Following the general procedure, the title compound was obtained as white solid, m.p. 125 – 128 ℃, 121.4 mg, 72% yield. (Rf = 0.30, eluent: PE/EtOAc = 2/1).

^{1}H NMR (600 MHz, CDCl$_3$): δ 7.94 – 7.87 (m, 2H), 7.62 – 7.55 (m, 1H), 7.50 – 7.44 (m, 2H), 6.85 (d, *J* = 10.3 Hz, 1H), 6.67 – 6.59 (m, 1H), 6.17 (d, *J* = 10.3 Hz, 1H), 5.46 (d, *J* = 16.2 Hz, 1H), 3.77 (s, 3H), 3.14 – 3.02 (m, 2H), 3.01 – 2.92 (m, 2H), 2.71 – 2.60 (m, 2H), 2.54 – 2.47 (m, 1H).

^{13}C NMR (151 MHz, CDCl$_3$): δ 197.0, 196.8, 171.6, 149.0, 147.4, 136.5, 133.8, 130.8, 128.9, 128.1, 116.5, 104.7, 53.1, 51.1, 40.8, 39.9, 39.6, 36.7.

IR (neat): 2954, 2921, 2217, 1731, 1671, 1630, 1432, 1295, 966, 750 cm^{-1}.

HRMS (ESI-TOF) calcd. for $C_{20}H_{19}NNaO_4$ $[M+Na]^+$: 360.1206, found: 360.1204.

Supplementary Table 4. Crystal data and structure refinement for **19c**

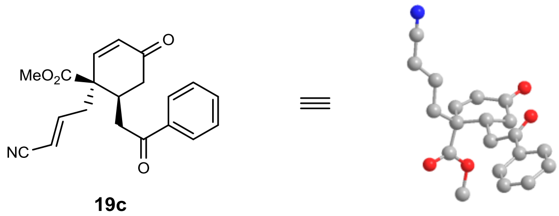

19c

Bond precision:	c-c = 0.0033 Å		Wavelength=0.71073
Cell:	a=7.8409(6)	b=22.6541(19)	c=9.7975(8)
	α=90	β=92.452(4)	γ=90
Temperature:	295 K		

	Calculated	Reported
Volume	1738.7(2)	1738.7(2)
Space group	P 21/n	P2(1)/n
Hall group	-P 2yn	
Moiety formula	C_{20} H_{19} N O_4	C_{20} H_{19} N O_4
Sum formula	C_{20} H_{19} N O_4	C_{20} H_{19} N O_4
Mr	337.36	337.36
Dx,g cm^{-3}	1.289	1.289
Z	4	4
Mu (mm^{-1})	0.090	0.090
F000	712.0	712.0
F000'	712.36	
h,k,lmax	10,29,12	10,29,12
Nref	3991	3973
Tmin,Tmax	0.973,0.989	0.973,0.989
Tmin'	0.965	

Correction method= # Reported T Limits: Tmin=0.973 Tmax=0.989

AbsCorr = EMPIRICAL

Data completeness= 0.995 Theta(max)= 27.500

R(reflections)= 0.0558(2676) wR2(reflections)= 0.1890(3973)

S = 1.058 Npar= 226

The single crystal of **19c** was obtained by recrystallization in a solution of DCM/Hexane at rt. Crystallographic data (excluding structural factors) for this compound **19c** has been deposited at the Cambridge Crystallographic Data Centre under the deposition number CCDC 2020057.

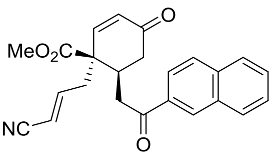

19d

Methyl (1*R*,6*S*)-1-((*E*)-3-cyanoallyl)-6-(2-(naphthalen-2-yl)-2-oxoethyl)-4-oxocyclohex-2-ene-1-carboxylate (19d):

Following the general procedure, the title compound was obtained as white solid, m.p. 146 – 147 ℃, 127.8 mg, 66% yield. (Rf = 0.17, eluent: PE/EtOAc = 2/1).

^{1}H NMR (600 MHz, CDCl$_3$): δ 8.39 (s, 1H), 7.96 (d, *J* = 8.2 Hz, 2H), 7.91 – 7.84 (m, 2H), 7.64 – 7.52 (m, 2H), 6.87 (d, *J* = 10.3 Hz, 1H), 6.69 – 6.60 (m, 1H), 6.18 (d, *J* = 10.3 Hz, 1H), 5.47 (d, *J* = 16.2 Hz, 1H), 3.79 (s, 3H), 3.24 – 3.07 (m, 3H), 3.01 – 2.94 (m, 1H), 2.74 – 2.63 (m, 2H), 2.59 – 2.50 (m, 1H).

^{13}C NMR (151 MHz, CDCl$_3$): δ 196.9, 196.8, 171.7, 149.0, 147.5, 135.8, 133.8, 132.5, 130.8, 129.9, 129.7, 128.9, 128.8, 127.9, 127.1, 123.6, 116.5, 104.7, 53.1, 51.1, 40.8, 34.0, 39.6, 36.8.

IR (neat): 2971, 2935, 2217, 1731, 1666, 1630, 1214, 1043, 962, 854 cm^{-1}.

HRMS (ESI-TOF) calcd. for $C_{24}H_{21}NNaO_4$ [M+Na]$^+$: 410.1363, found: 410.1361.

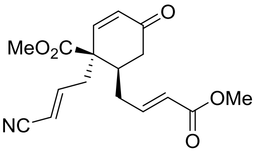

19e

Methyl (1*R*,6*R*)-1-((*E*)-3-cyanoallyl)-6-((*E*)-4-methoxy-4-oxobut-2-en-1-yl)-4-oxocyclohex-2-ene-1-carboxylate (19e):

In oder to improve the selectivity of the reaction, the crude dearomatization product was passed through a short silica gel column and concentrated under vacuum. Then the mixture was treated with acid and was further purified following the general procedure. The title compound was obtained as colorless oil, 87.2 mg, 55% yield. (Rf = 0.17, eluent: PE/EtOAc = 2/1).

^{1}H NMR (600 MHz, CDCl$_3$): δ 6.82 (d, *J* = 10.3 Hz, 1H), 6.79 – 6.71 (m, 1H), 6.63 – 6.54 (m, 1H), 6.15 (d, *J* = 10.3 Hz, 1H), 5.82 (d, *J* = 15.6 Hz, 1H), 5.48 (d, *J* = 16.2 Hz, 1H), 3.78 (s, 3H), 3.72 (s, 3H), 2.99 – 2.91 (m, 1H), 2.75 – 2.66 (m, 1H), 2.60 – 2.44 (m, 2H), 2.39 – 2.31 (m, 2H), 2.12 – 2.02 (m, 1H).

^{13}C NMR (151 MHz, CDCl$_3$): δ 196.4, 171.2, 166.3, 148.9, 147.2, 144.5, 130.9, 124.3, 116.4, 104.7, 53.1, 51.8, 51.3, 41.0, 40.7, 38.7, 33.6.

IR (neat): 2953, 2224, 1716, 1677, 1435, 1214, 1157, 1038, 968 cm^{-1}.

HRMS (ESI-TOF) calcd. for $C_{17}H_{19}NNaO_5$ $[M+Na]^+$: 340.1155, found: 340.1156.

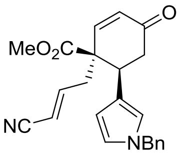

19f

Methyl (1*S*,6*S*)-6-(1-benzyl-1*H*-pyrrol-3-yl)-1-((*E*)-3-cyanoallyl)-4-oxocyclohex-2-ene-1-carboxylate (19f):

Following the general procedure, the title compound was obtained as yellow oil, 93.5 mg, 50% yield. (Rf = 0.33, eluent: PE/EtOAc = 2/1).

^{1}H NMR (600 MHz, $CDCl_3$): δ 7.35 – 7.30 (m, 2H), 7.28 (d, *J* = 7.3 Hz, 1H), 6.97 – 6.90 (m, 3H), 6.66 – 6.62 (m, 1H), 6.54 – 6.47 (m, 1H), 6.17 (d, *J* = 10.4 Hz, 1H), 6.06 (t, *J* = 3.2 Hz, 1H), 5.89 – 5.85 (m, 1H), 5.32 (d, *J* = 16.2 Hz, 1H), 5.12 – 4.99 (m, 2H), 3.60 (s, 3H), 3.38 (t, *J* = 6.1 Hz, 1H), 2.83 – 2.76 (m, 1H), 2.61 – 2.49 (m, 2H), 2.47 – 2.41 (m, 1H).

^{13}C NMR (151 MHz, $CDCl_3$): δ 196.6, 171.4, 149.4, 147.4, 138.1, 130.6, 130.2, 129.1, 128.0, 126.2, 122.6, 116.6, 108.2, 107.9, 104.0, 52.8, 52.2, 50.9, 41.9, 40.7, 39.3.

IR (neat): 3029, 2950, 2223, 1727, 1679, 1452, 1225, 1078, 964, 911 cm^{-1}.

HRMS (ESI-TOF) calcd. for $C_{23}H_{22}N_2NaO_3$ $[M+Na]^+$: 397.1523, found: 397.1532.

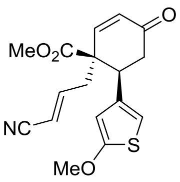

19g

Methyl (1*S*,6*R*)-1-((*E*)-3-cyanoallyl)-6-(5-methoxythiophen-3-yl)-4-oxocyclohex-2-ene-1-carboxylate (19g):

Following a procedure similar to the synthesis of **19e**, the title compound was obtained as yellow oil, 79.5 mg, 48% yield. (Rf = 0.33, eluent: PE/EtOAc = 2/1).

^{1}H NMR (600 MHz, $CDCl_3$): δ 6.78 (d, *J* = 10.3 Hz, 1H), 6.67 – 6.59 (m, 1H), 6.41 (d, *J* = 3.9 Hz, 1H), 6.24 (d, *J* = 10.3 Hz, 1H), 5.98 (d, *J* = 3.9 Hz, 1H), 5.50 (d, *J* = 16.3 Hz, 1H), 3.83 (s, 3H), 3.70 (s, 3H), 3.53 – 3.46 (m, 1H), 3.15 – 3.07 (m, 1H), 3.03 – 2.95 (m, 1H), 2.78 – 2.67 (m, 2H).

^{13}C NMR (151 MHz, $CDCl_3$): δ 196.8, 170.6, 165.8, 149.7, 147.1, 131.5, 126.8, 124.3, 116.6, 104.6, 102.9, 60.2, 53.0, 52.6, 44.1, 41.8, 40.4.

IR (neat): 2918, 2848, 2223, 1728, 1678, 1557, 1499, 1431, 1201, 964 cm^{-1}.

HRMS (ESI-TOF) calcd. for $C_{17}H_{17}NNaO_4S$ $[M+Na]^+$: 354.0770, found: 354.0773.

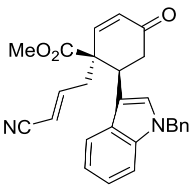

19h

Methyl (1*S*,6*S*)-6-(1-benzyl-1*H*-indol-3-yl)-1-((*E*)-3-cyanoallyl)-4-oxocyclohex-2-ene-1-carboxylate (19h):

Following the general procedure, the title compound was obtained as yellow solid, m.p. 82 – 83 ℃, 108.2 mg, 51% yield. (Rf = 0.33, eluent: PE/EtOAc = 2/1).

^{1}H NMR (600 MHz, CDCl$_3$): δ 7.50 (d, *J* = 7.9 Hz, 1H), 7.33 – 7.24 (m, 4H), 7.22 – 7.17 (m, 1H), 7.16 – 7.12 (m, 1H), 7.04 (d, *J* = 7.1 Hz, 2H), 6.90 (d, *J* = 10.3 Hz, 1H), 6.83 (s, 1H), 6.72 – 6.63 (m, 1H), 6.27 (d, *J* = 10.3 Hz, 1H), 5.42 (d, *J* = 16.3 Hz, 1H), 5.31 (d, *J* = 16.0 Hz, 1H), 5.21 (d, *J* = 15.9 Hz, 1H), 3.86 – 3.80 (m, 1H), 3.40 (s, 3H), 3.19 – 3.11 (m, 1H), 3.07 – 2.99 (m, 1H), 2.84 – 2.77 (m, 1H), 2.72 – 2.64 (m, 1H).

^{13}C NMR (151 MHz, CDCl$_3$): δ 197.9, 171.2, 150.0, 147.6, 137.2, 136.1, 131.1, 128.9, 127.9, 127.7, 126.8, 126.1, 122.5, 119.8, 118.6, 116.7, 113.4, 110.1, 104.2, 53.2, 52.6, 50.1, 41.56, 41.3, 39.6.

IR (neat): 3055, 2949, 2251, 2227, 1723, 1676, 1631, 1541, 1216, 1175, 918, 810 cm^{-1}.

HRMS (ESI-TOF) calcd. for $C_{27}H_{24}N_2NaO_3$ $[M+Na]^+$: 447.1679, found: 447.1685.

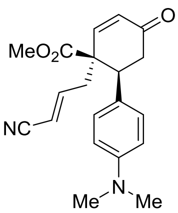

19i

Methyl (1*S*,2*S*)-2-((*E*)-3-cyanoallyl)-4'-(dimethylamino)-5-oxo-1,2,5,6-tetrahydro-[1,1'-biphenyl]-2-carboxylate (19i):

Following a procedure similar to the synthesis of **19e**, the title compound was obtained as yellow oil, 123.4 mg, 73% yield. (Rf = 0.23, eluent: PE/EtOAc = 2/1).

^{1}H NMR (600 MHz, CDCl$_3$): δ 6.89 (d, *J* = 8.7 Hz, 2H), 6.74 – 6.61 (m, 4H), 6.25 (d, *J* = 10.2 Hz, 1H), 5.48 (d, *J* = 16.3 Hz, 1H), 3.65 (s, 3H), 3.29 – 3.24 (m, 1H), 3.23 – 3.16 (m, 1H), 2.95 – 2.87 (m, 7H), 2.62 – 2.51 (m, 2H).

^{13}C NMR (151 MHz, CDCl$_3$): δ 198.5, 170.6, 150.5, 150.2, 147.2, 131.4, 128.9, 125.4, 116.8, 112.3, 104.3, 52.76, 52.71, 47.9, 40.9, 40.4, 40.0.

IR (neat): 2951, 2223, 1729, 1678, 1612, 1522, 1352, 1202, 966, 908, 825 cm^{-1}.

HRMS (ESI-TOF) calcd. for $C_{20}H_{22}N_2NaO_3$ [M+Na]$^+$: 361.1523, found: 361.1522.

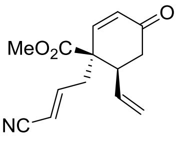

19j

Methyl (1*S*,6*S*)-1-((*E*)-3-cyanoallyl)-4-oxo-6-vinylcyclohex-2-ene-1-carboxylate (19j):

In lieu of the addition of base and nucleophile in one portion described in the gereneral procedure, base and nucleophile were added sequentially to the mixture. Specifically, a solution of 4-ethylmorpholine (157μL, 2.5 equiv) in DCM (1.0 mL) was added to the mixture dropwise in 10 min using syringe pump. After stirring for 30 min, to the mixture was added vinylzinc chloride (**3u**, 0.35 M in THF, 4.3 mL, 3.0 equiv) dropwise in 10 min using syringe pump. The mixture was gradually warmed to -70 ℃ and stirred for 12 h. Then the mixture was passed through a short silica gel column and concentrated under vacuum. As a result, the title compound was obtained as colorless oil, 83.3 mg, 68% yield. (Rf = 0.45, eluent: PE/EtOAc = 2/1).

^{1}H NMR (600 MHz, CDCl$_3$): δ 6.76 (d, *J* = 10.3 Hz, 1H), 6.64 – 6.56 (m, 1H), 6.14 (d, *J* = 10.3 Hz, 1H), 5.72 – 5.63 (m, 1H), 5.49 – 5.42 (m, 1H), 5.20 – 5.08 (m, 2H), 3.72 (s, 3H), 2.98 – 2.90 (m, 1H), 2.85 – 2.79 (m, 1H), 2.72 – 2.61 (m, 2H), 2.55 – 2.49 (m, 1H).

^{13}C NMR (151 MHz, CDCl$_3$): δ 197.0, 170.8, 149.5, 147.1, 135.3, 131.0, 119.1, 116.6, 104.5, 52.9, 51.0, 46.7, 40.3, 39.9.

IR (neat): 2954, 2920, 2223, 1727, 1678, 1434, 1219, 1104, 970, 928, 809 cm^{-1}.

HRMS (ESI-TOF) calcd. for $C_{14}H_{15}NNaO_3$ [M+Na]$^+$: 268.0944, found: 268.0953.

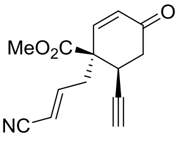

19k

Methyl (1*S*,6*S*)-1-((*E*)-3-cyanoallyl)-6-ethynyl-4-oxocyclohex-2-ene-1-carboxylate (19k):

Following a procedure similar to the synthesis of **19j**, ethynylzinc chloride (**3v**,

0.22 M in THF, 6.8 mL, 3.0 equiv) was used as nucleophile. The title compound was obtained as white solid, m.p. 97 – 90 ℃, 74.1 mg, 61% yield. (Rf = 0.45, eluent: PE/EtOAc = 2/1).

^{1}H NMR (600 MHz, CDCl$_3$): δ 6.76 (d, *J* = 10.3 Hz, 1H), 6.67 – 6.58 (m, 1H), 6.16 (d, *J* = 10.3 Hz, 1H), 5.55 – 5.46 (m, 1H), 3.80 (s, 3H), 3.17 – 3.10 (m, 1H), 3.01 – 2.90 (m, 2H), 2.84 – 2.76 (m, 1H), 2.74 – 2.67 (m, 1H), 2.24 (d, *J* = 2.5 Hz, 1H).

^{13}C NMR (151 MHz, CDCl$_3$): δ 195.3, 170.2, 148.8, 146.1, 131.1, 116.5, 104.9, 80.8, 73.3, 53.2, 51.1, 40.4, 39.5, 34.2.

IR (neat): 3267, 2952, 2854, 2224, 1726, 1688, 1219, 1174, 972, 869 cm^{-1}.

HRMS (ESI-TOF) calcd. for $C_{14}H_{13}NNaO_3$ [M+Na]$^+$: 266.0788, found: 266.0789

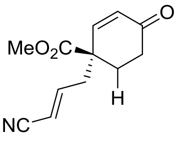

19l

Methyl (*S*,*E*)-1-(3-cyanoallyl)-4-oxocyclohex-2-ene-1-carboxylate (19l): Following the general procedure, triethylsilane (116.3 mg, 2.0 equiv) was used as nucleophile. The title compound was obtained as colorless oil, 43.8 mg, 40% yield. (Rf = 0.30, eluent: PE/EtOAc = 2/1).

^{1}H NMR (600 MHz, CDCl$_3$): δ 6.79 (d, *J* = 10.2 Hz, 1H), 6.66 – 6.56 (m, 1H), 6.06 (d, *J* = 10.2 Hz, 1H), 5.45 (d, *J* = 16.2 Hz, 1H), 3.77 (d, *J* = 7.6 Hz, 3H), 2.73 – 2.63 (m, 2H), 2.58 – 2.41 (m, 3H), 2.04 – 1.94 (m, 1H).

^{13}C NMR (151 MHz, CDCl$_3$): δ 197.4, 172.5, 148.9, 148.2, 130.4, 116.5, 104.3, 53.1, 47.5, 41.9, 34.4, 30.8.

IR (neat): 2972, 2881, 2226, 1734, 1677, 1436, 1272, 1086, 1045, 972 cm^{-1}.

HRMS (ESI-TOF) calcd. for $C_{12}H_{13}NNaO_3$ [M+Na]$^+$: 242.0788, found: 242.0810.

7 Elaboration of 1,4-cyclohexediene and bicyclic products

The procedure for the synthesis of methyl (*E*)-2-(3-cyanoallyl)-5-(1-methoxy-1-oxobutan-2-yl)benzoate (20a):

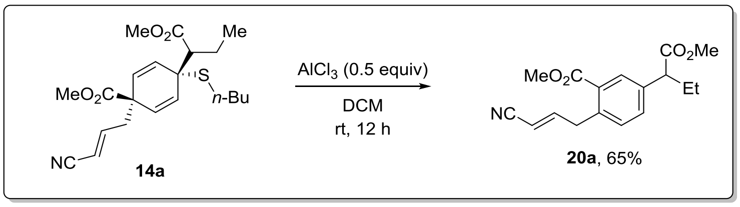

To a solution of **14a** (78.3 mg, 0.2 mmol) in DCM (2 mL) was added $AlCl_3$ (13.3 mg, 0.1 mmol) at rt. The resulting mixture was stirred at rt for 12 h. Then the mixture was passed through a short silica gel column and concentrated under vacuum. The obtained residue was further purified by flash chromatography on silica gel affording **20a** as colorless oil, 39.2 mg, 65% yield. (Rf = 0.17, eluent: PE/EtOAc = 10/1).

^{1}H NMR (400 MHz, $CDCl_3$): δ 7.92 (d, *J* = 2.0 Hz, 1H), 7.45 (dd, *J* = 7.9, 2.1 Hz, 1H), 7.17 (d, *J* = 7.9 Hz, 1H), 6.90 (dt, *J* = 16.3, 6.5 Hz, 1H), 5.28 – 5.20 (m, 1H), 3.91 – 3.88 (m, 5H), 3.67 (s, 3H), 3.49 (t, *J* = 7.7 Hz, 1H), 2.17 – 2.07 (m, 1H), 1.85 – 1.75 (m, 1H), 0.89 (t, *J* = 7.4 Hz, 3H).

^{13}C NMR (101 MHz, $CDCl_3$): δ 174.1, 167.1, 154.3, 138.7, 137.3, 132.4, 131.9, 131.1, 129.5, 117.6, 100.6, 52.9, 52.3, 52.2, 37.8, 26.8, 12.2.

IR (neat): 2954, 2223, 1719, 1631, 1435, 1273, 1197, 1166, 1081, 974 cm^{-1}.

HRMS (ESI-TOF) calcd. for $C_{17}H_{19}NNaO_4$ $[M+Na]^+$: 324.1206, found: 324.1208.

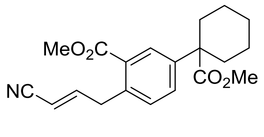

20f

Methyl (*E*)-2-(3-cyanoallyl)-5-(1-(methoxycarbonyl)cyclohexyl)benzoate (20f):

Following a procedure similar to the synthesis of **20a**, the title compound was obtained as colorless oil, 47.8 mg, 70% yield. (Rf = 0.27, eluent: PE/Acetone = 5/1).

^{1}H NMR (400 MHz, $CDCl_3$): δ 8.00 (d, *J* = 2.0 Hz, 1H), 7.55 – 7.49 (m, 1H), 7.16 (d, *J* = 8.1 Hz, 1H), 6.95 – 6.84 (m, 1H), 5.24 (d, *J* = 16.4 Hz, 1H), 3.93 – 3.84 (m, 5H), 3.64 (s, 3H), 2.49 (d, *J* = 13.2 Hz, 2H), 1.75 – 1.61 (m, 5H), 1.53 – 1.43 (m, 2H), 1.32 – 1.25 (m, 1H).

^{13}C NMR (151 MHz, $CDCl_3$): δ 175.3, 167.3, 154.3, 143.4, 136.8, 131.7, 130.6, 129.4, 129.0, 117.6, 100.5, 52.3, 50.7, 37.8, 34.7, 25.5, 23.6.

IR (neat): 2933, 2859, 2223, 1726, 1632, 1500, 1453, 1434, 1261, 1132, 1082, 973, 790 cm^{-1}.

HRMS (ESI-TOF) calcd. for $C_{20}H_{23}NNaO_4$ $[M+Na]^+$: 364.1519, found: 364.1515.

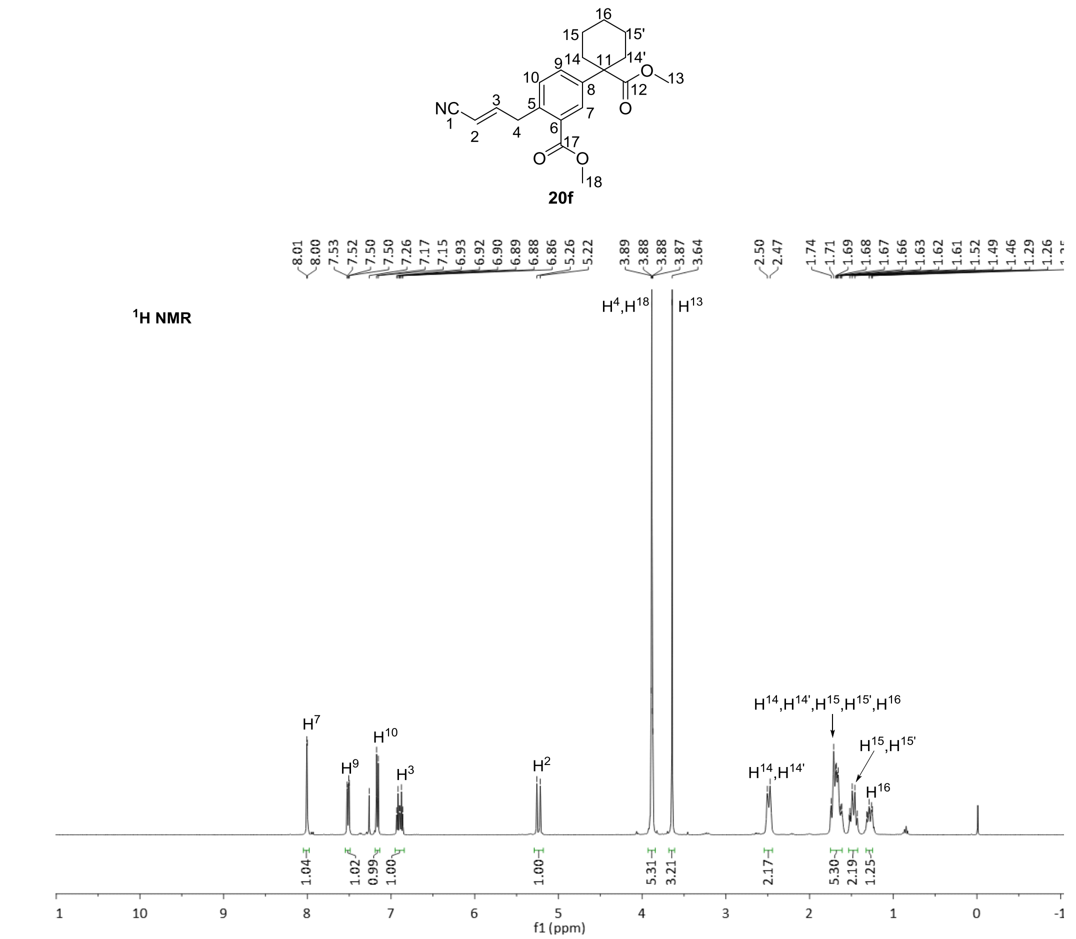

Supplementary Figure 21. ^{1}H NMR spectrum of **20f**

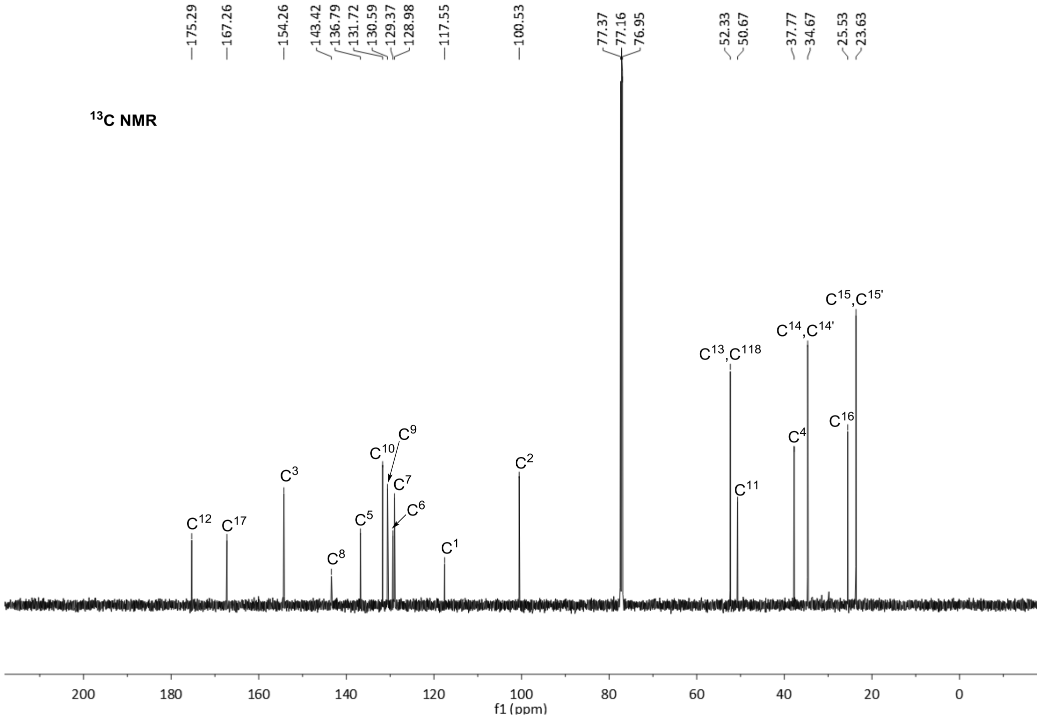

Supplementary Figure 22. ^{13}C NMR spectrum of **20f**

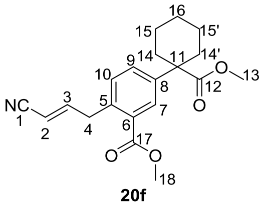

20f

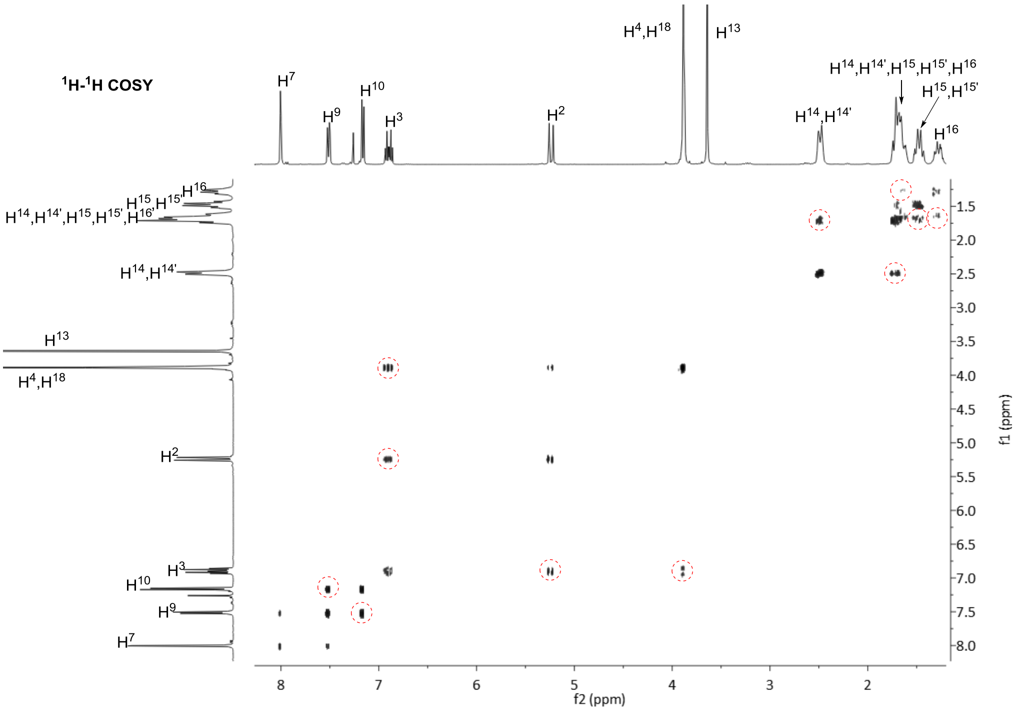

Supplementary Figure 23. ^{1}H-^{1}H COSY spectrum of **20f**

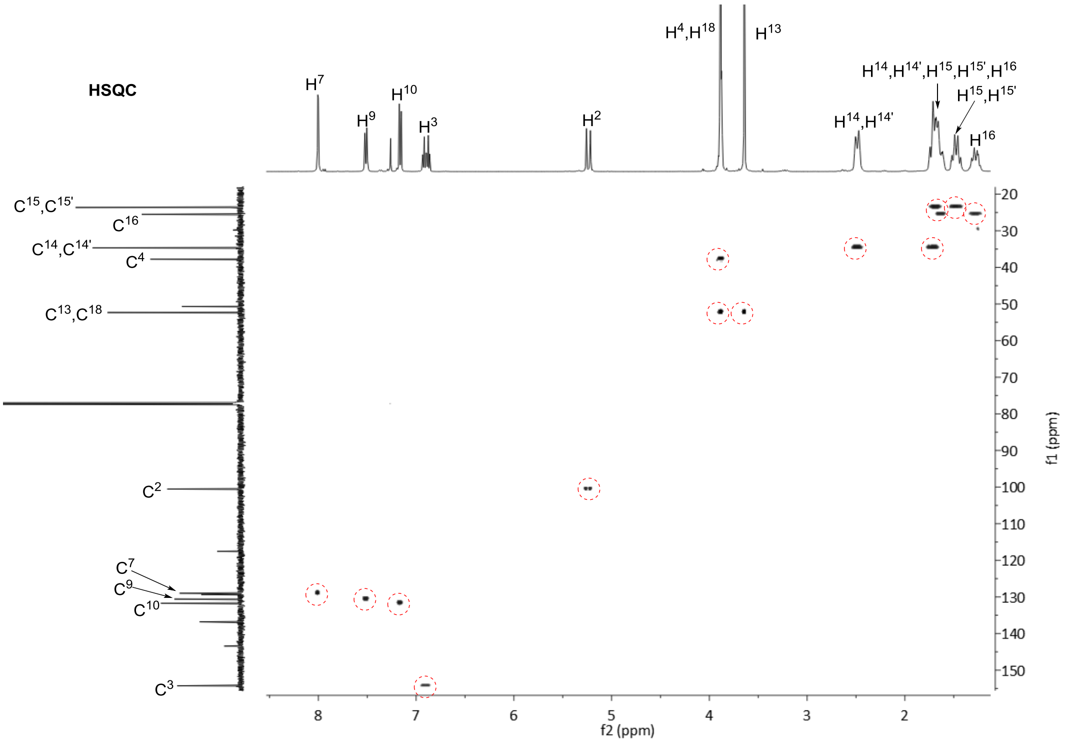

Supplementary Figure 24. HSQC spectrum of **20f**

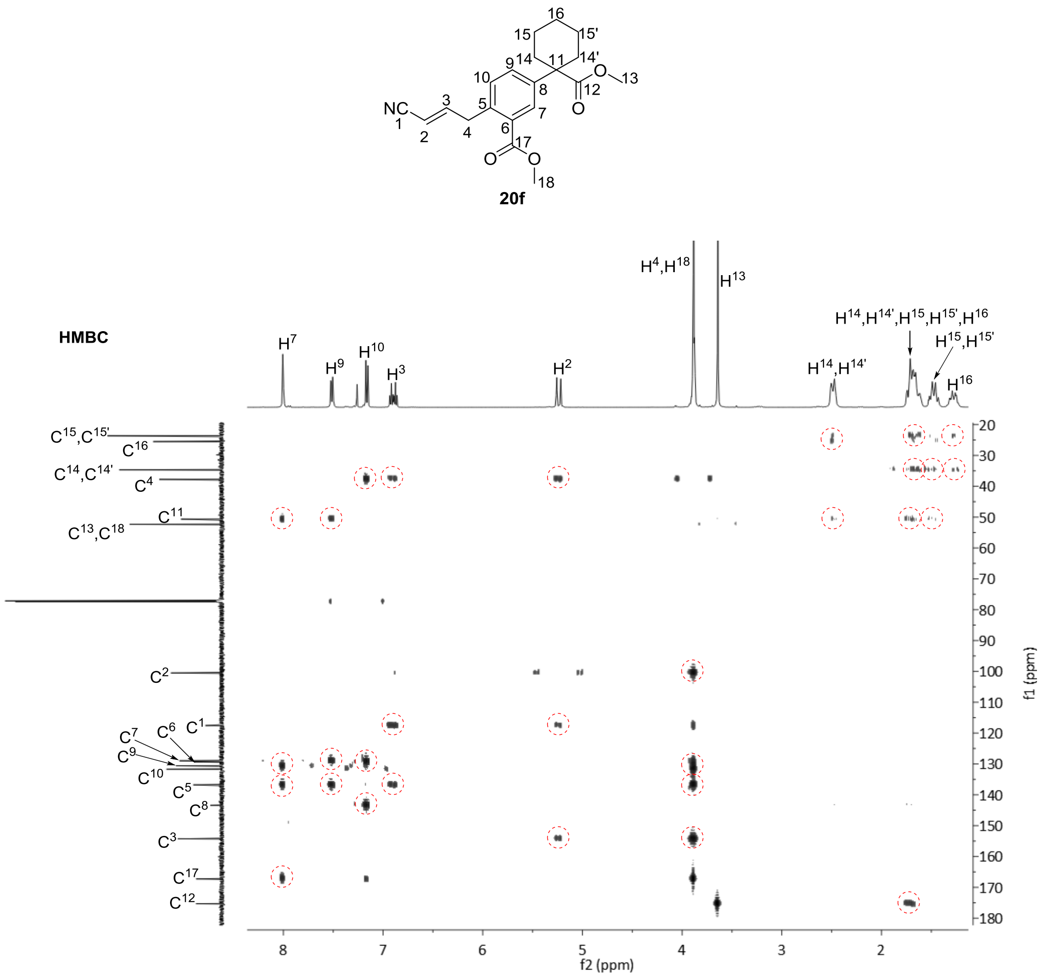

Supplementary Figure 25. HMBC spectrum of **20f**

The procedure for the synthesis of (*E*)-4-(4-(argioamino)phenyl)but-2-enenitrile (21a):

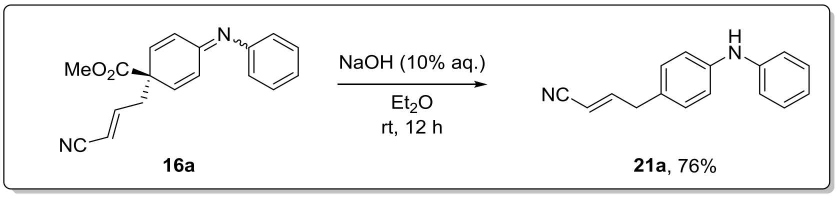

To a solution of **16a** (58.4 mg, 0.2 mmol) in Et_2O (2 mL) was added NaOH (10% aq., 2 mL) at rt. The resulting mixture was stirred at rt for 12 h. After that, the mixture was diluted with H_2O and then extracted with Et_2O. The combined organic phase was dried over Na_2SO_4, concentrated under reduced pressure and purified by flash chromatography on silica gel. The title compound **21a** was obtained as yellow oil, 35.6 mg, 76% yield. (Rf = 0.36, eluent: PE/EtOAc = 5/1)

^{1}H NMR (600 MHz, $CDCl_3$): δ 7.26 – 7.21 (m, 2H), 7.05 – 6.98 (m, 6H), 6.92 (t, *J* = 7.4 Hz, 1H), 6.88 – 6.81 (m, 1H), 5.69 (s, 1H), 5.29 – 5.22 (m, 1H), 3.48 – 3.41 (m, 2H).

^{13}C NMR (151 MHz, $CDCl_3$): δ 154.7, 143.0, 142.4, 129.9, 129.5, 128.2, 121.3, 118.1, 118.0, 117.6, 100.6, 38.8.

IR (neat): 3395, 2890, 2215, 1600, 1521, 1496, 1446, 1335, 1310, 1235, 1176, 972, 845 cm^{-1}.

HRMS (ESI-TOF) calcd. for $C_{16}H_{15}N_2$ $[M+H]^+$: 235.1230, found: 235.1224.

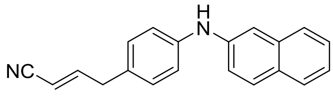

21i

(*E*)-4-(4-(naphthalen-2-ylamino)phenyl)but-2-enenitrile (21i): Following a procedure similar to the synthesis of **21a**, the title compound was obtained as yellow solid, m.p. 56 – 58 ℃, 49.5 mg, 87% yield. (Rf = 0.41, eluent: PE/EtOAc = 5/1).

^{1}H NMR (600 MHz, $CDCl_3$): δ 7.76 (d, *J* = 8.7 Hz, 2H), 7.66 (d, *J* = 8.2 Hz, 1H), 7.47 – 7.40 (m, 2H), 7.36 – 7.30 (m, 1H), 7.25 – 7.20 (m, 1H), 7.16 – 7.11 (m, 2H), 7.07 (d, *J* = 8.4 Hz, 2H), 6.89 (dt, *J* = 16.2, 6.5 Hz, 1H), 5.91 (s, 1H), 5.36 – 5.27 (m, 1H), 3.53 – 3.46 (m, 2H).

^{13}C NMR (151 MHz, $CDCl_3$): δ 154.6, 142.2, 140.7, 134.7, 130.0, 129.4, 128.7, 127.8, 126.6, 126.6, 123.7, 120.1, 118.6, 117.6, 111.9, 100.6, 38.8.

IR (neat): 3354, 3047, 2922, 2226, 1628, 1601, 1529, 1313, 1238, 1177, 967 cm^{-1}.

HRMS (ESI-TOF) calcd. for $C_{20}H_{16}N_2Na$ $[M+Na]^+$: 307.1206, found: 307.1200.

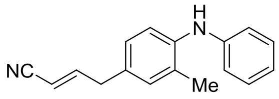

21j

(*E*)-4-(3-methyl-4-(phenylamino)phenyl)but-2-enenitrile (21j): Following a procedure similar to the synthesis of **21a**, the title compound was obtained as yellow solid, m.p. 73 – 75 ℃, 44.2 mg, 89% yield. (Rf = 0.40, eluent: PE/EtOAc = 5/1).

^{1}H NMR (600 MHz, $CDCl_3$): δ 7.30 – 7.25 (m, 2H), 7.21 (d, *J* = 8.1 Hz, 1H), 6.99 – 6.95 (m, 3H), 6.95 – 6.84 (m, 3H), 5.39 (s, 1H), 5.31 (d, *J* = 16.3 Hz, 1H), 3.51 – 3.45 (m, 2H), 2.26 (s, 3H).

^{13}C NMR (151 MHz, $CDCl_3$): δ 154.7, 143.8, 140.5, 131.5, 129.5, 129.3, 128.8, 127.2, 120.8, 119.1, 117.7, 100.5, 38.9, 18.0.

IR (neat): 3377, 2917, 2225, 1630, 1593, 1517, 1489, 1416, 1310, 1076, 969 cm^{-1}.

HRMS (ESI-TOF) calcd. for $C_{17}H_{16}N_2Na$ $[M+Na]^+$: 271.1206, found: 271.1215.

The procedure for the synthesis of methyl (*S*,*E*)-1-(3-cyanoallyl)-4-oxo-1,4-dihydronaphthalene-1-carboxylate (22a):

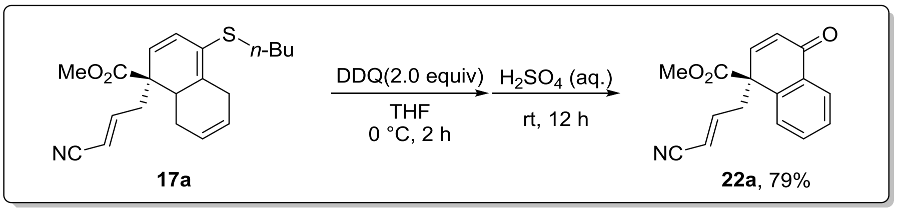

To a solution of **17a** (68.7 mg, 0.2 mmol) in THF (2.0 mL) was added a solution of DDQ (90.8 mg, 2.0 equiv) in THF (1.0 mL) dropwise in 10 min using syringe pump at 0 ℃. The resulting mixture was stirred at 0 ℃ for 2 h. After that, to the mixture was added H_2SO_4 aq. (1 M, 3 ml). Then the resulted mixture was stirrred at rt for 12 h. The mixture was then diluted with EtOAc and washed with $NaHCO_3$ aq. and brine. The combined organic phase was dried over Na_2SO_4, concentrated under reduced pressure and purified by flash chromatography on silica gel. The title compound **22a** was obtained as white solid, m.p. 90 – 95 ℃, 42.2 mg, 79% yield. (Rf = 0.14, eluent: PE/EtOAc = 3/1).

^{1}H NMR (400 MHz, CDCl$_3$): δ 8.27 – 8.18 (m, 1H), 7.67 – 7.60 (m, 1H), 7.56 – 7.47 (m, 2H), 6.87 (d, *J* = 10.2 Hz, 1H), 6.64 (d, *J* = 10.2 Hz, 1H), 6.21 (dt, *J* = 16.3, 7.6 Hz, 1H), 5.34 – 5.24 (m, 1H), 3.67 (s, 3H), 3.18 – 3.12 (m, 2H).

^{13}C NMR (101 MHz, CDCl$_3$): δ 183.6, 171.2, 148.4, 145.4, 139.8, 133.5, 131.7, 130.8, 128.9, 127.6, 126.4, 116.4, 104.4, 53.7, 51.7, 42.6.

IR (neat): 2951, 2223, 1727, 1655, 1634, 1596, 1456, 1429, 1392, 1303, 1227, 1156, 974 cm^{-1}.

HRMS (ESI-TOF) calcd. for $C_{16}H_{13}NNaO_3$ $[M+Na]^+$: 290.0788, found: 290.0781.

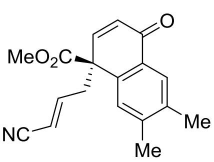

22b

Methyl (*S*,*E*)-1-(3-cyanoallyl)-6,7-dimethyl-4-oxo-1,4-dihydronaphthalene-1-carboxylate Methyl (22b):

Following a procedure similar to the synthesis of **22a**, the title compound was obtained as yellow oil, 39.6 mg, 67% yield. (Rf = 0.23, eluent: PE/EtOAc = 3/1).

^{1}H NMR (600 MHz, CDCl$_3$): δ 7.97 (s, 1H), 7.21 (s, 1H), 6.80 (d, *J* = 10.1 Hz, 1H), 6.59 (d, *J* = 10.2 Hz, 1H), 6.19 (dt, *J* = 16.2, 7.6 Hz, 1H), 5.32 – 5.25 (m, 1H), 3.66 (s, 3H), 3.18 – 3.07 (m, 2H), 2.38 – 2.32 (m, 6H).

^{13}C NMR (151 MHz, CDCl$_3$): δ 183.8, 171.5, 148.7, 145.0, 143.5, 138.0, 137.4, 130.9, 129.6, 128.2, 127.1, 116.6, 104.1, 53.7, 51.3, 42.5, 20.6, 19.7.

IR (neat): 2961, 2922, 2220, 1729, 1660, 1607, 1455, 1414, 1236, 1163, 1025, 961, 840 cm^{-1}.

HRMS (ESI-TOF) calcd. for $C_{18}H_{17}NNaO_3$ [M+Na]$^+$: 318.1101, found: 318.1089.

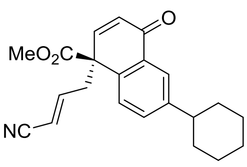

22c

Methyl (*S,E*)-1-(3-cyanoallyl)-6-cyclohexyl-4-oxo-1,4-dihydronaphthalene-1-carboxylate Methyl (22c):

Following a procedure similar to the synthesis of **22a**, the title compound was obtained as white solid, m.p. 118 – 121 ℃, 48.9 mg, 70% yield. (Rf = 0.30, eluent: PE/EtOAc = 3/1).

^{1}H NMR (600 MHz, CDCl$_3$): δ 8.06 (d, *J* = 2.0 Hz, 1H), 7.47 – 7.44 (m, 1H), 7.40 (d, *J* = 8.1 Hz, 1H), 6.84 (d, *J* = 10.2 Hz, 1H), 6.61 (d, *J* = 10.2 Hz, 1H), 6.21 (dt, *J* = 16.4, 7.5 Hz, 1H), 5.29 (d, *J* = 16.3 Hz, 1H), 3.66 (s, 3H), 3.17 – 3.08 (m, 2H), 2.63 – 2.55 (m, 1H), 1.93 – 1.83 (m, 4H), 1.79 – 1.73 (m, 1H), 1.50 – 1.36 (m, 4H), 1.30 – 1.25 (m, 1H).

^{13}C NMR (151 MHz, CDCl$_3$): δ 183.9, 171.3, 149.0, 148.7, 145.3, 137.2, 132.5, 131.4, 130.8, 126.4, 125.5, 116.5, 104.2, 53.7, 51.5, 44.3, 42.7, 34.2, 34.2, 26.8, 26.1.

IR (neat): 2924, 2850, 2224, 1737, 1660, 1636, 1603, 1430, 1386, 1306, 1224, 1162, 1053 cm^{-1}.

HRMS (ESI-TOF) calcd. for $C_{22}H_{23}NNaO_3$ [M+Na]$^+$: 372.1570, found: 372.1561.

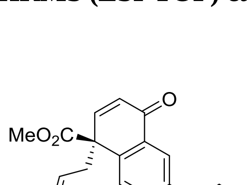

22d

Methyl (*S,E*)-1-(3-cyanoallyl)-4-oxo-6-phenyl-1,4-dihydronaphthalene-1-carboxylate (22d):

Following a procedure similar to the synthesis of **22a**, the title compound was obtained as yellow solid, m.p. 94 – 96 ℃, 48.7 mg, 71% yield. (Rf = 0.20, eluent: PE/EtOAc = 3/1).

^{1}H NMR (400 MHz, CDCl$_3$): δ 8.30 (d, *J* = 8.2 Hz, 1H), 7.78 – 7.73 (m, 1H), 7.69 (d, *J* = 1.7 Hz, 1H), 7.64 – 7.59 (m, 2H), 7.53 – 7.48 (m, 2H), 7.47 – 7.43 (m, 1H), 6.89 (d, *J* = 10.2 Hz, 1H), 6.66 (d, *J* = 10.2 Hz, 1H), 6.28 (dt, *J* = 16.2, 7.6 Hz, 1H), 5.32 (d, *J* = 16.3 Hz, 1H), 3.70 (s, 3H), 3.28 – 3.14 (m, 2H).

^{13}C NMR (151 MHz, CDCl$_3$): δ 183.4, 171.2, 148.4, 146.3, 145.3, 140.3, 139.4, 130.8, 130.4, 129.2, 128.8, 128.2, 127.8, 127.5, 124.8, 116.5, 104.4, 53.8, 51.8, 42.8.

IR (neat): 2924, 2852, 2225, 1734, 1665, 1607, 1432, 1304, 1230, 1162, 967, 905 cm^{-1}.

HRMS (ESI-TOF) calcd. for $C_{22}H_{17}NNaO_3$ [M+Na]$^+$: 366.1101, found: 366.1094.

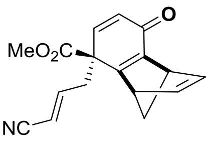

22i

Methyl (1*R*,4*S*,5*S*)-5-((*E*)-3-cyanoallyl)-8-oxo-1,4,5,8-tetrahydro-1,4-methanonaphthalene-5-carboxylate (22i):

Following a procedure similar to the synthesis of **22a**, THF/H_2O=10/1 (2 mL) instead of THF (2 mL) was used as solvent, the title compound was obtained as colorless oil, 28.7 mg, 51% yield. (Rf = 0.19, eluent: PE/EtOAc = 2/1).

^{1}H NMR (400 MHz, CDCl$_3$): δ 6.86 – 6.78 (m, 1H), 6.75 – 6.68 (m, 2H), 6.42 – 6.29 (m, 2H), 5.45 (d, *J* = 16.2 Hz, 1H), 4.17 – 4.11 (m, 1H), 3.76 – 3.72 (m, 1H), 3.67 (s, 3H), 3.06 (d, *J* = 7.4 Hz, 2H), 2.17 (d, *J* = 6.8 Hz, 1H), 2.03 (d, *J* = 6.8 Hz, 1H).

^{13}C NMR (101 MHz, CDCl$_3$): δ 181.0, 172.1, 168.9, 151.2, 148.2, 143.6, 142.7, 141.3, 132.3, 116.3, 104.3, 72.0, 53.4, 52.7, 52.4, 47.4, 39.2.

IR (neat): 2951, 2225, 1733, 1651, 1622, 1594, 1434, 1400, 1292, 1231, 1023, 969, 840 cm^{-1}.

HRMS (ESI-TOF) calcd. for $C_{17}H_{15}NNaO_3$ [M+Na]$^+$: 304.0944, found: 304.0945.

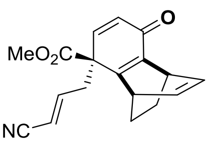

22j

Methyl (1*R*,4*S*,5*S*)-5-((*E*)-3-cyanoallyl)-8-oxo-1,4,5,8-tetrahydro-1,4-ethanonaphthalene-5-carboxylate (22j):

Following a procedure similar to the synthesis of **22i**, DDQ was added at rt and stirrred at rt for 2 h. The title compound was obtained as colorless oil, 30.1 mg, 51% yield. (Rf = 0.22, eluent: PE/EtOAc = 2/1).

^{1}H NMR (400 MHz, $CDCl_3$): δ 6.64 (d, *J* = 10.0 Hz, 1H), 6.43 – 6.36 (m, 2H), 6.36 – 6.24 (m, 2H), 5.46 (d, *J* = 16.3 Hz, 1H), 4.46 – 4.40 (m, 1H), 3.83 – 3.77 (m, 1H), 3.67 (s, 3H), 3.18 – 3.09 (m, 1H), 2.91 – 2.82 (m, 1H), 1.52 – 1.40 (m, 2H), 1.34 – 1.23 (m, 2H).

^{13}C NMR (101 MHz, $CDCl_3$): δ 180.6, 169.7, 158.2, 148.6, 143.7, 142.3, 135.0, 133.1, 131.6, 116.4, 104.2, 53.5, 53.0, 39.0, 38.2, 33.2, 24.9, 24.7.

IR (neat): 2953, 2924, 2360, 2225, 1732, 1656, 1630, 1592, 1434, 1407, 1339, 1230, 1151 cm^{-1}.

HRMS (ESI-TOF) calcd. for $C_{18}H_{17}NNaO_3$ $[M+Na]^+$: 318.1101, found: 318.1102.

8 Density functional theory (DFT) calculations and in situ NMR studies

8.1 Density functional theory (DFT) calculations

Computational Details

All structures were optimized at M062X[29-31] /6-31G(d,p) level with solvent effects accounted by the SMD[32] solvent model, using the experimental solvent (dichloromethane). Harmonic frequency analysis calculations were subsequently performed to verify the optimized geometries to be minima (no imaginary frequency) or transition states (TSs, having unique one imaginary frequency). The energies were then improved by M062X/6-311++G(d,p)//M062X/6-31G(d,p) single-point calculations with solvent effects accounted by the SMD solvent model, using the experimental solvent (dichloromethane). The refined energies were then corrected to enthalpies and free energies at experimental temperature (178.15K) and 1 atm, using the M062X/6-31G(d,p) harmonic frequencies. Total energies and Cartesian coordinates of all optimized structures are given below in this Supplementary Information. All standard DFT calculations were carried out using Gaussian 09 program.[33]

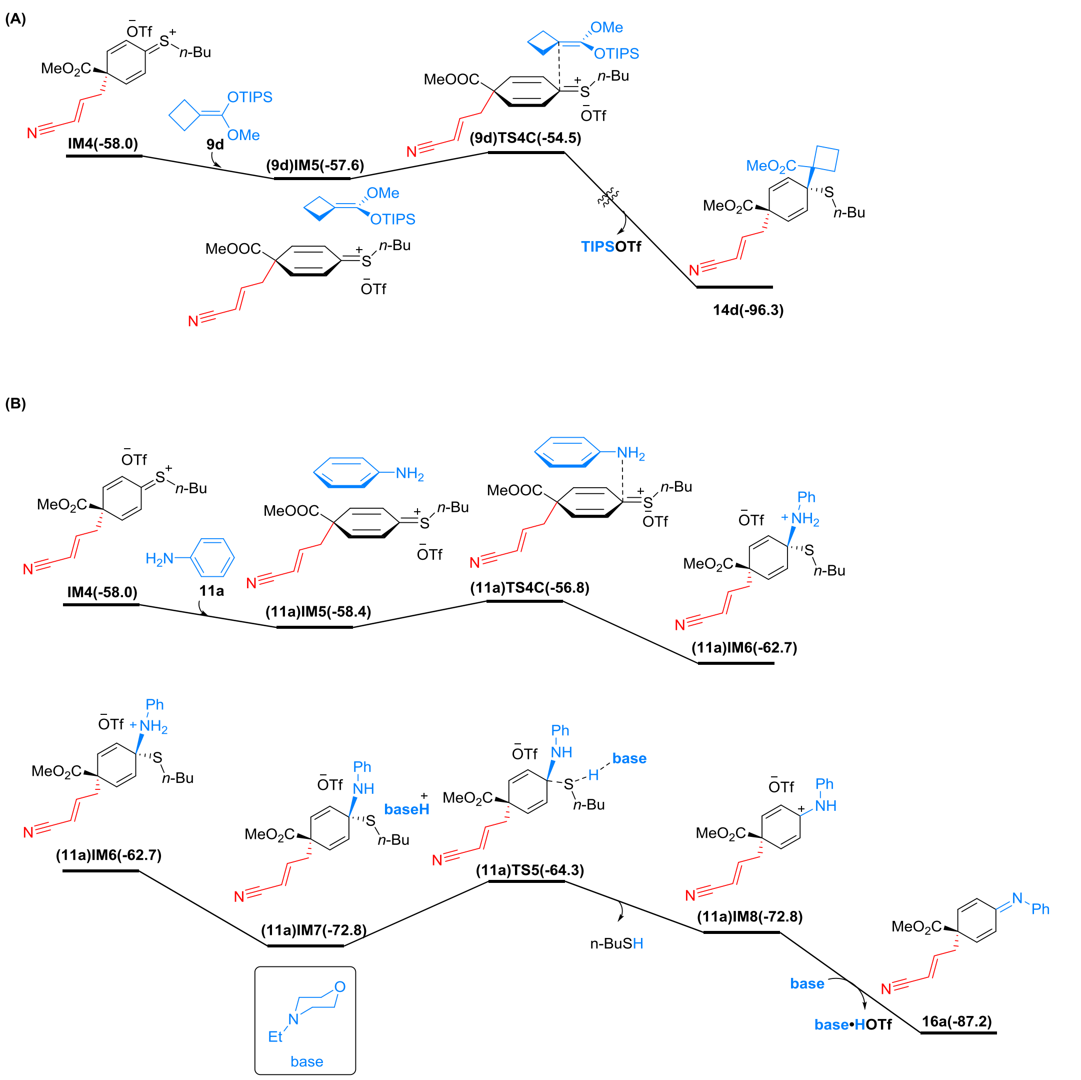

Supplementary Figure 26. Profile of the reactions of **IM4** with **9d** to form **14d** (**A**) and with **11a** to form **16a** (**B**).

8.2 Identification of IM1

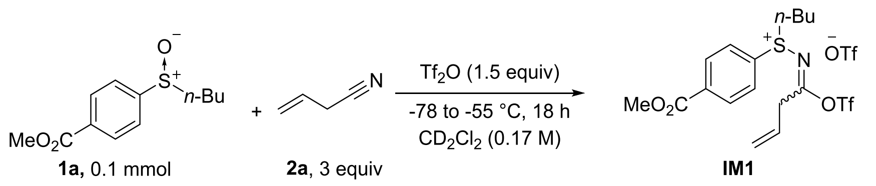

To an NMR tube were added **1a** (24 mg, 0.1 mmol), **2a** (24 μL, 0.3 mmol) and CD_2Cl_2 (0.6 mL). The mixture was cooled to -78 ℃ for 5 min. Once Tf_2O (25 μL, 0.15 mmol) was added to the mixture, the NMR tube was shaken for 15 min under -78 ℃. The temperature was then gradually warmed to -55 ℃ and maintained for 18 h. After that, the sample was measured by NMR spectroscopy. The ^{1}H and ^{13}C-NMR spectra of **IM1** were given below.

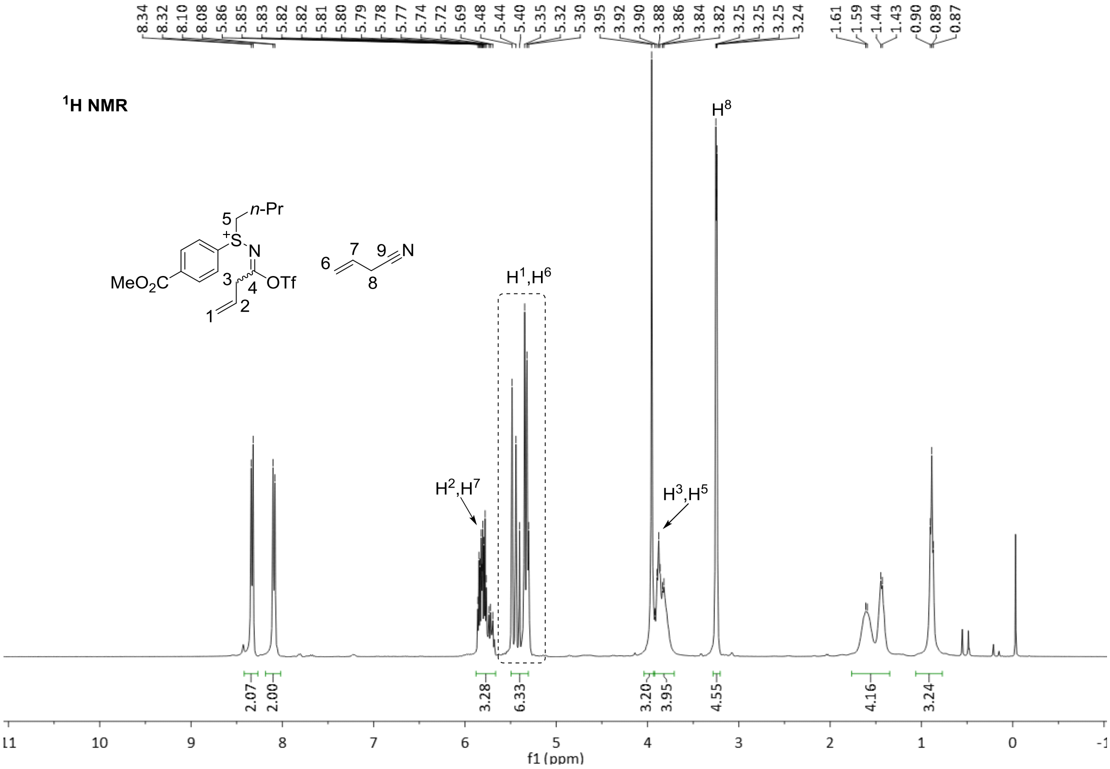

Supplementary Figure 27. ^{1}H NMR spectrum of **IM1**

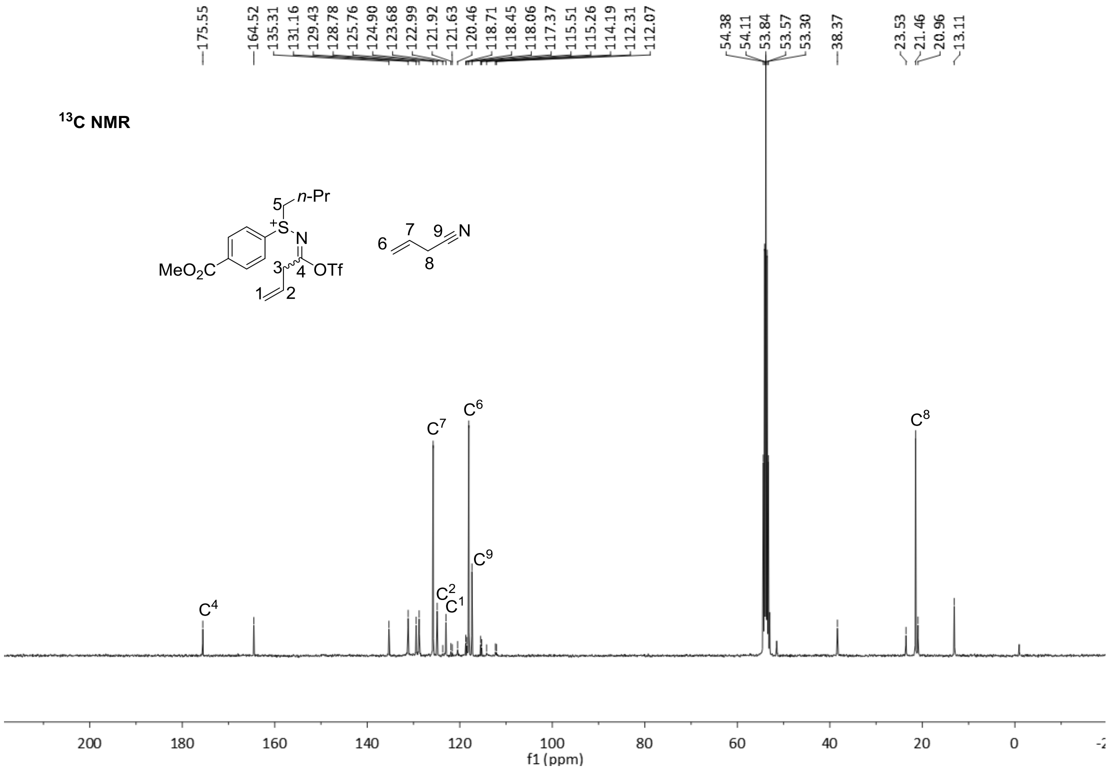

Supplementary Figure 28. ^{13}C NMR spectrum of **IM1**

8.3 Identification of IM4

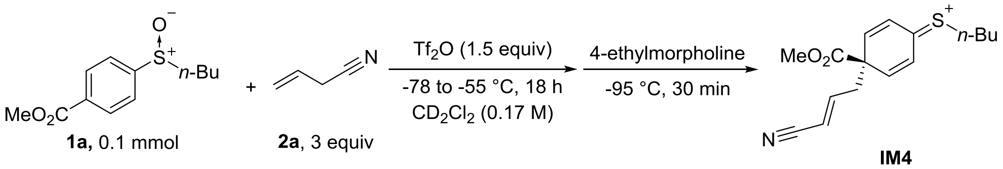

To an NMR tube were added **1a** (24 mg, 0.1 mmol), **2a** (24 μL, 0.3 mmol) and CD_2Cl_2 (0.6 mL). The mixture was cooled to -78 ℃ for 5 min. Once Tf_2O (25 μL, 0.15 mmol) was added to the mixture, the NMR tube was shaken for 15 min under -78 ℃. The temperature was then gradually warmed to -55 ℃ and maintained for 18 h. Then the mixture was cooled to -95 ℃, a solution of 4-ethylmorpholine (31.4 μL, 0.25 mmol) in CD_2Cl_2 (0.2 mL) was added to the mixture dropwise in 1 min. After shaking for 30 min, the sample was measured by HRMS. The target molecular weight was detected by ESI-MS as depicted below. However, due to the difficutlities in shimming field for NMR analysis, the sample was then treated with CF_3COOH (37 μL, 0.5 mmol) at -95 ℃. Then the sample was measured by NMR spectroscopic. NMR spectra of the acidified intermediate are given below.

The HRMS and NMR spectra of **IM4** are given below:

HRMS (ESI-TOF) calcd. for $[C_{16}H_{20}NO_2S]^+$: 290.1209, found: 290.1211.

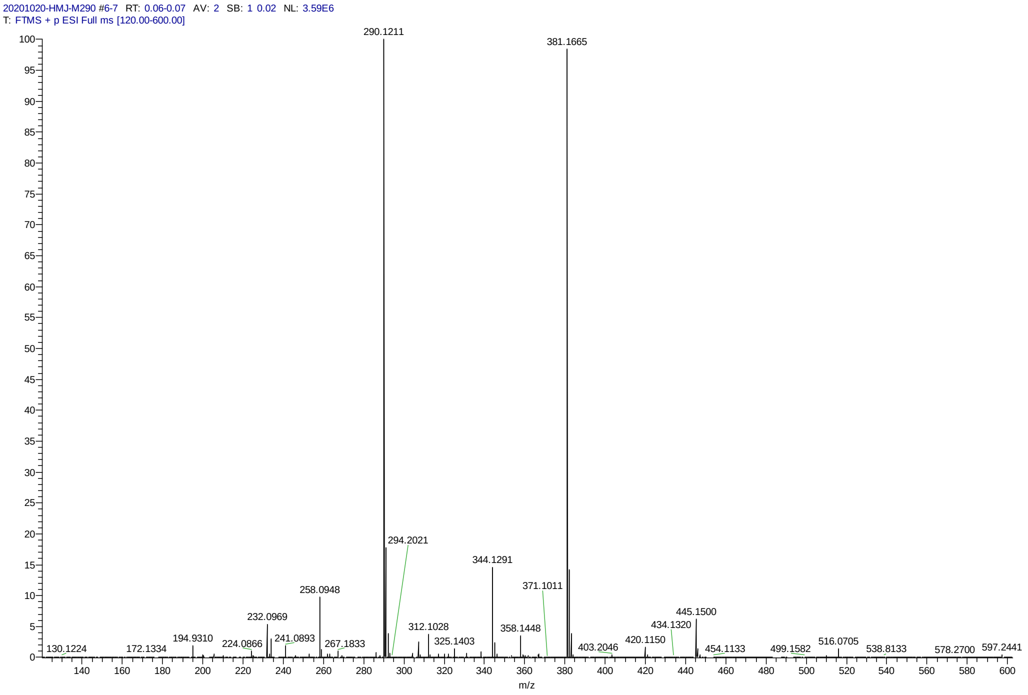

Supplementary Figure 29. HRMS spectrum of **IM4**

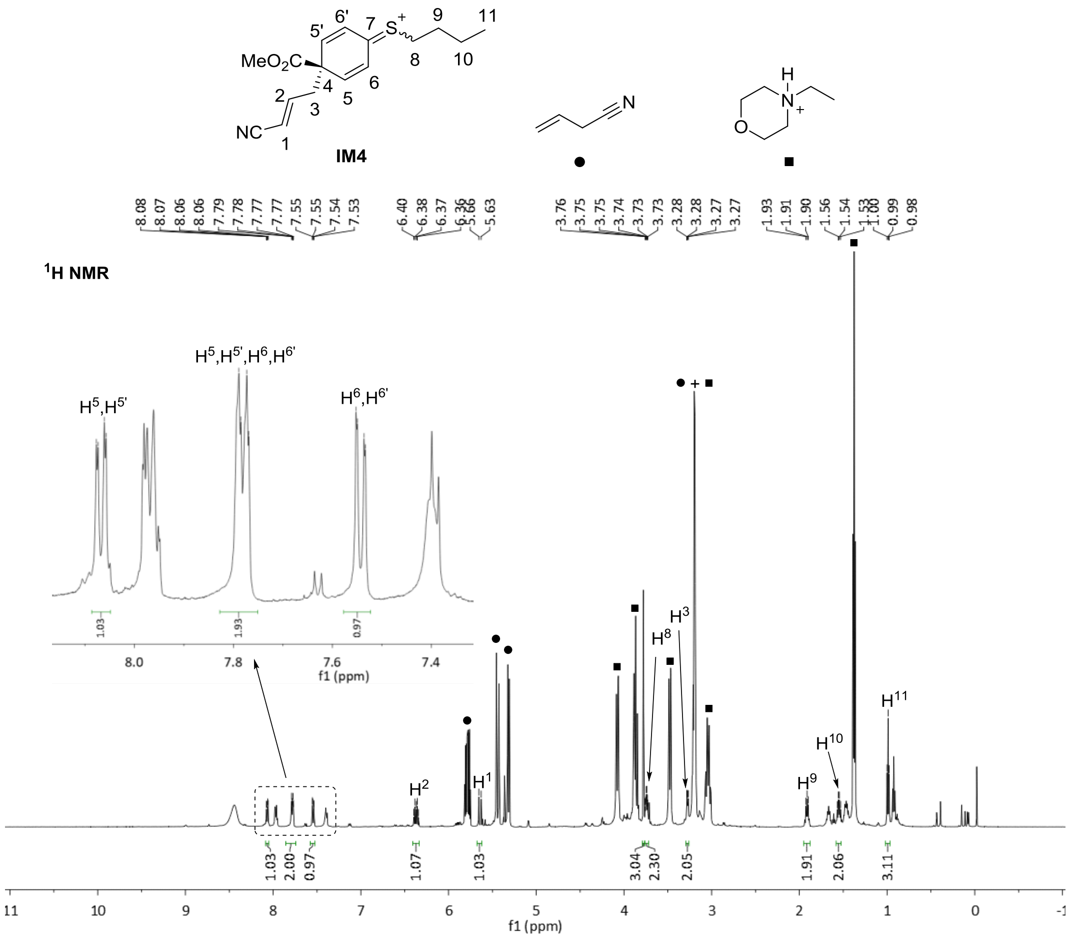

Supplementary Figure 30. ^{1}H NMR spectrum of **IM4**

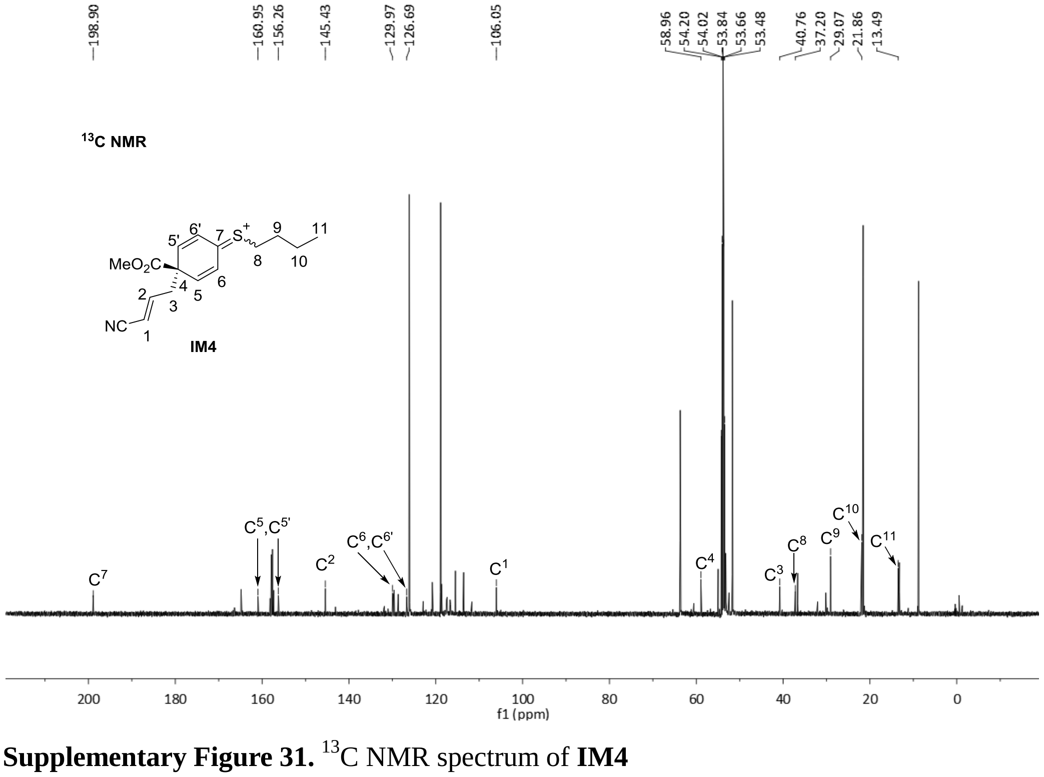

Supplementary Figure 31. ^{13}C NMR spectrum of **IM4**

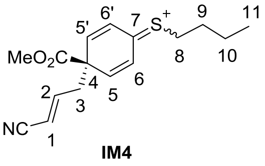

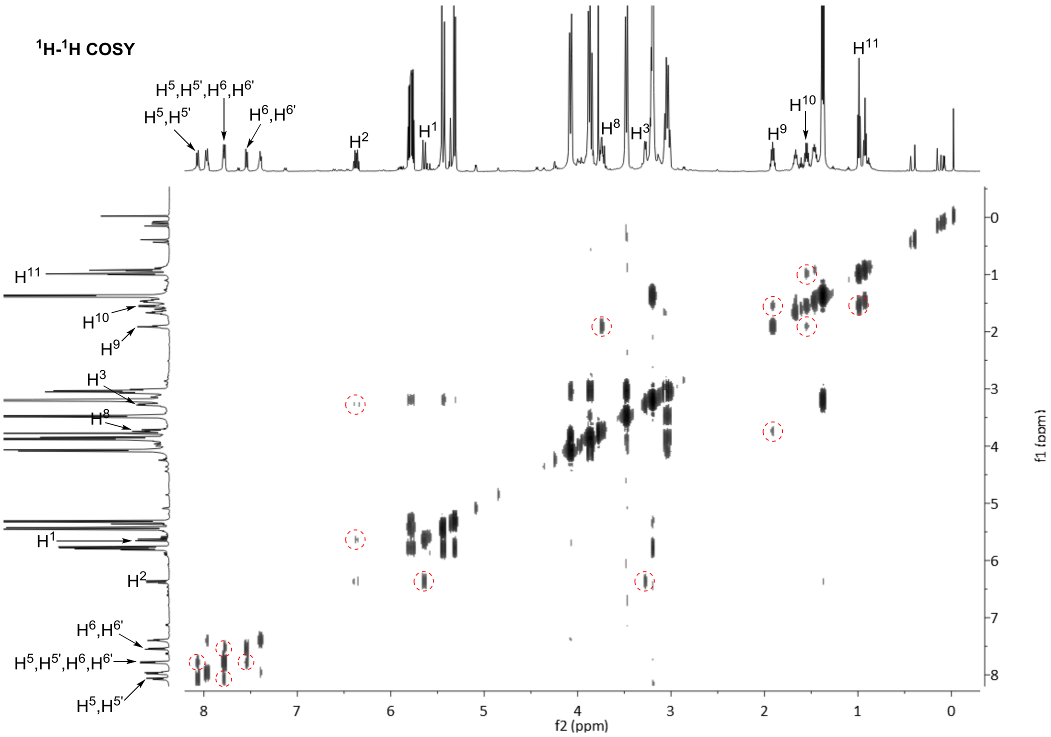

Supplementary Figure 32. ^{1}H-^{1}H COSY spectrum of **IM4**

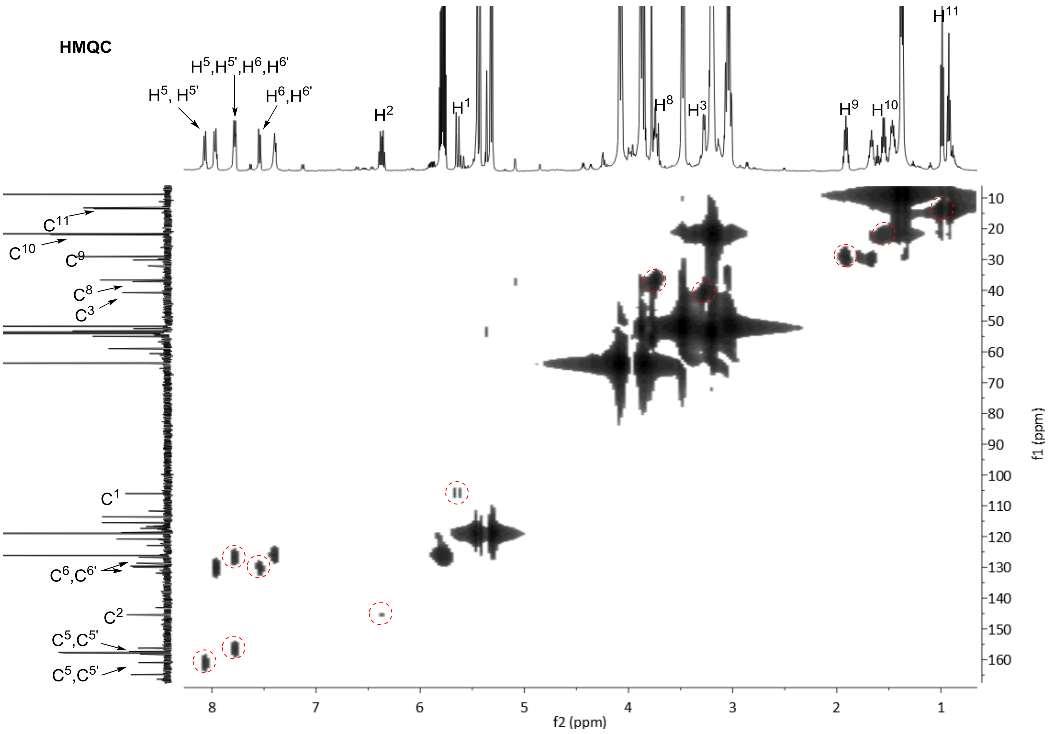

Supplementary Figure 33. HMQC spectrum of **IM4**

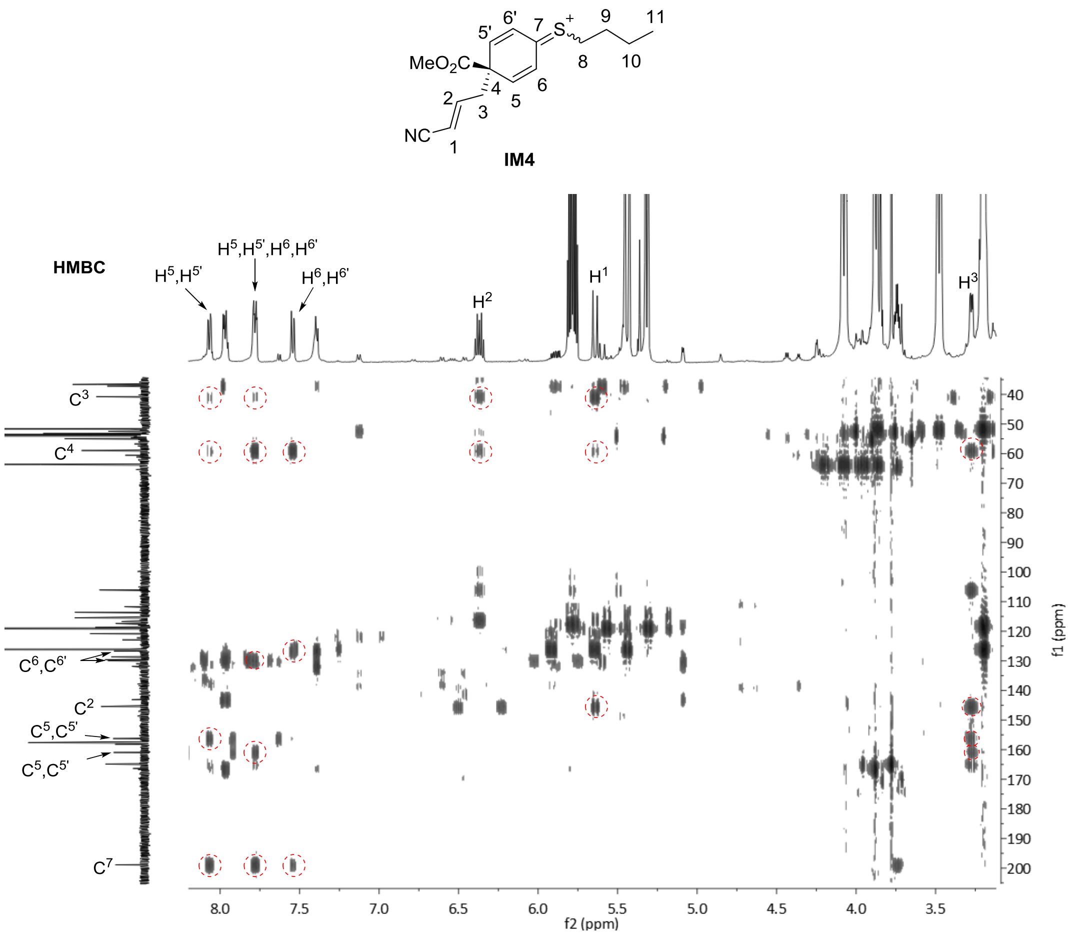

Supplementary Figure 34. HMBC spectrum of **IM4**

8.4 Study of stability of IM4

To a mixture of **1a** (120 mg, 0.5 mmol) and **2a** (121 μL, 1.5 mmol) in DCM (3.0 mL) was added Tf_2O (126 μL, 0.75 mmol) at -78 ℃ under N_2 atmosphere. The mixture was gradually warmed to -55 ℃. After stirring for 18 h, the mixture was cooled to -95 ℃. A solution of 4-ethylmorpholine (157 μL, 1.25 mmol) in DCM (1.0 mL) was added dropwise to the mixture in 10 min using syringe pump; After stirring for 30 min, the mixture was warmed to T (℃) and maintained for 1 h. To the mixture was added a solution of **3a** (202 mg, 1.0 mmol) in DCM (1.0 mL) dropwise in 10 min using syringe pump at -95 ℃. The mixture was gradually warmed to -70 ℃ and then stirred for 12 h. Then the mixture was passed through a short silica gel, concentrated under vacuum and prepared for NMR analysis with mesitylene as internal standard.

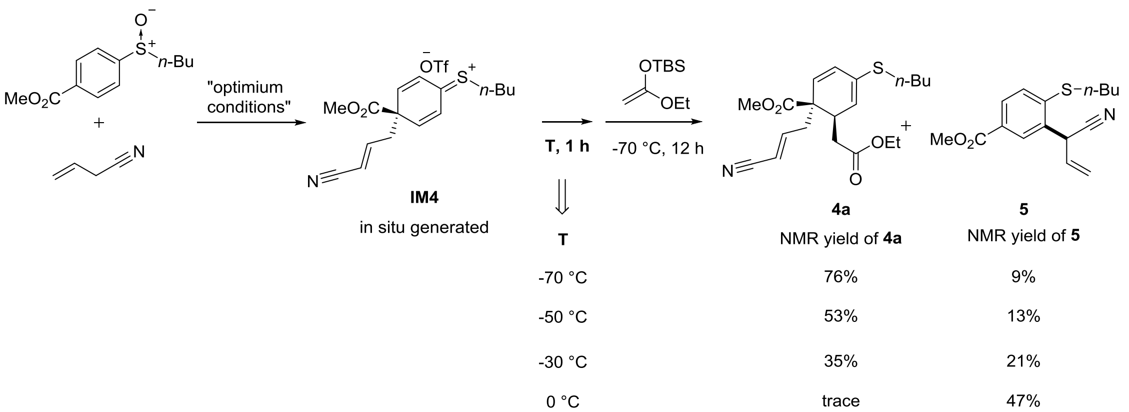

IM4
in situ generated

T	NMR yield of **4a** (**4a**)	NMR yield of **5** (**5**)
-70 °C	76%	9%
-50 °C	53%	13%
-30 °C	35%	21%
0 °C	trace	47%

9 NMR spectra

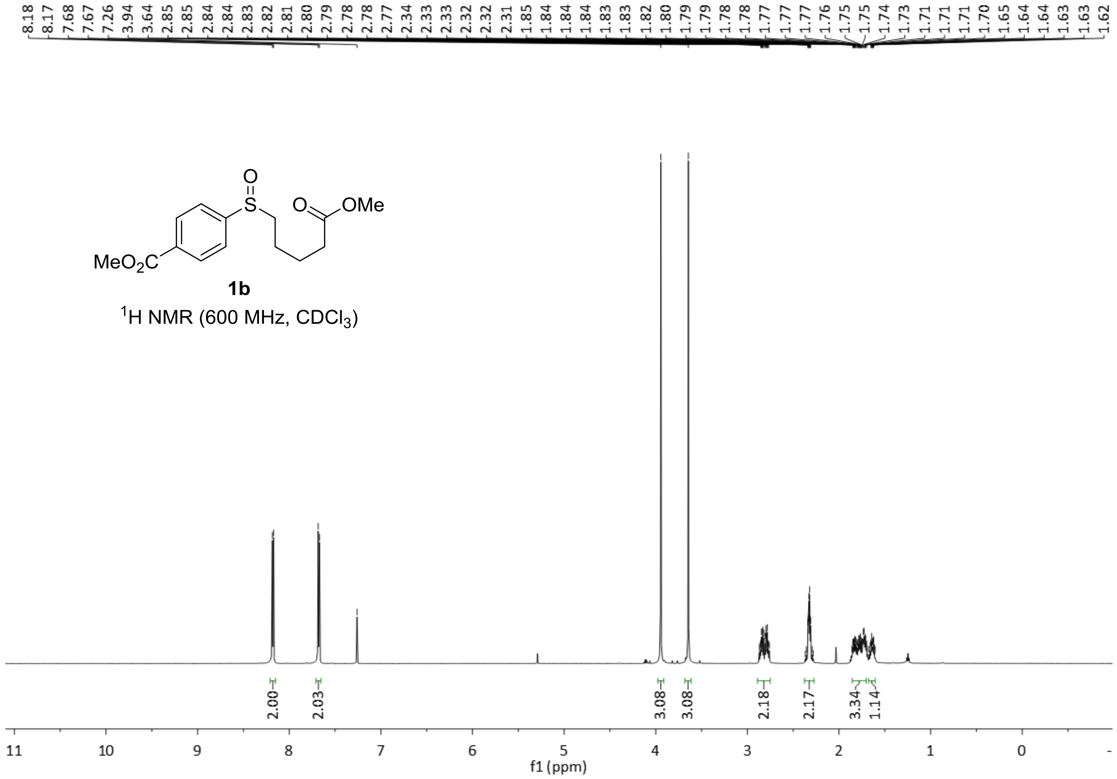

Supplementary Figure 35. ^{1}H NMR spectrum of **1b**

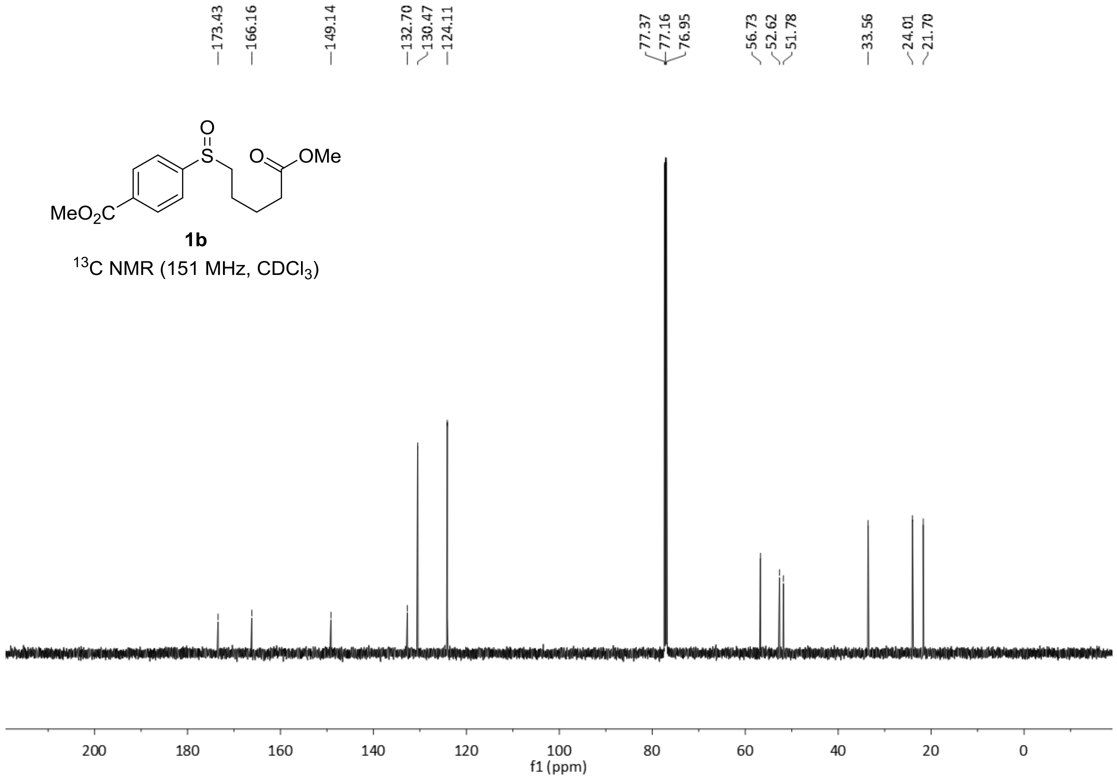

Supplementary Figure 36. ^{13}C NMR spectrum of **1b**

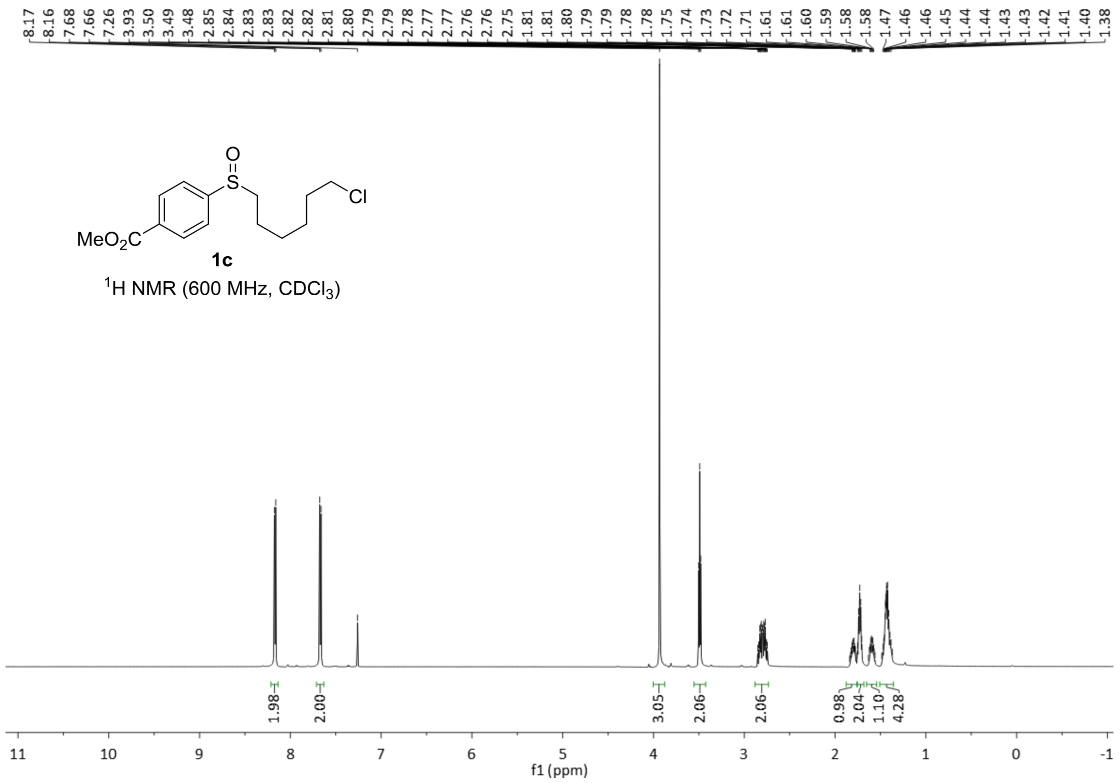

Supplementary Figure 37. ^{1}H NMR spectrum of **1c**

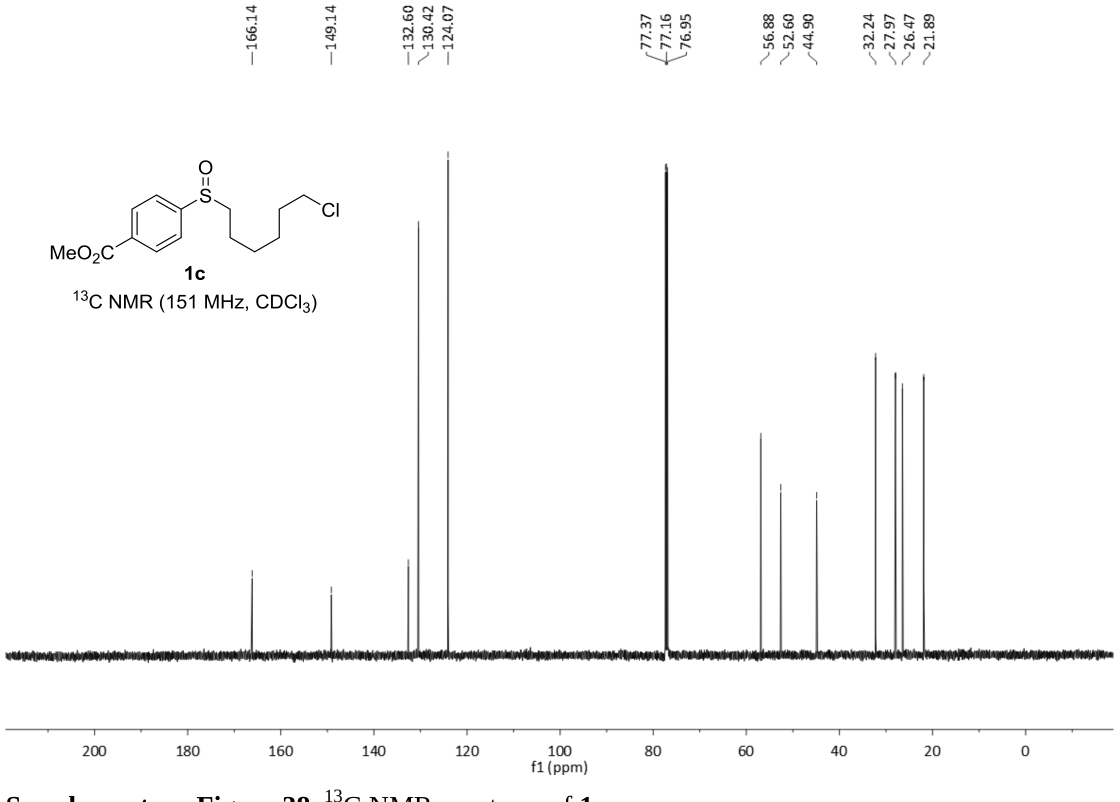

Supplementary Figure 38. ^{13}C NMR spectrum of **1c**

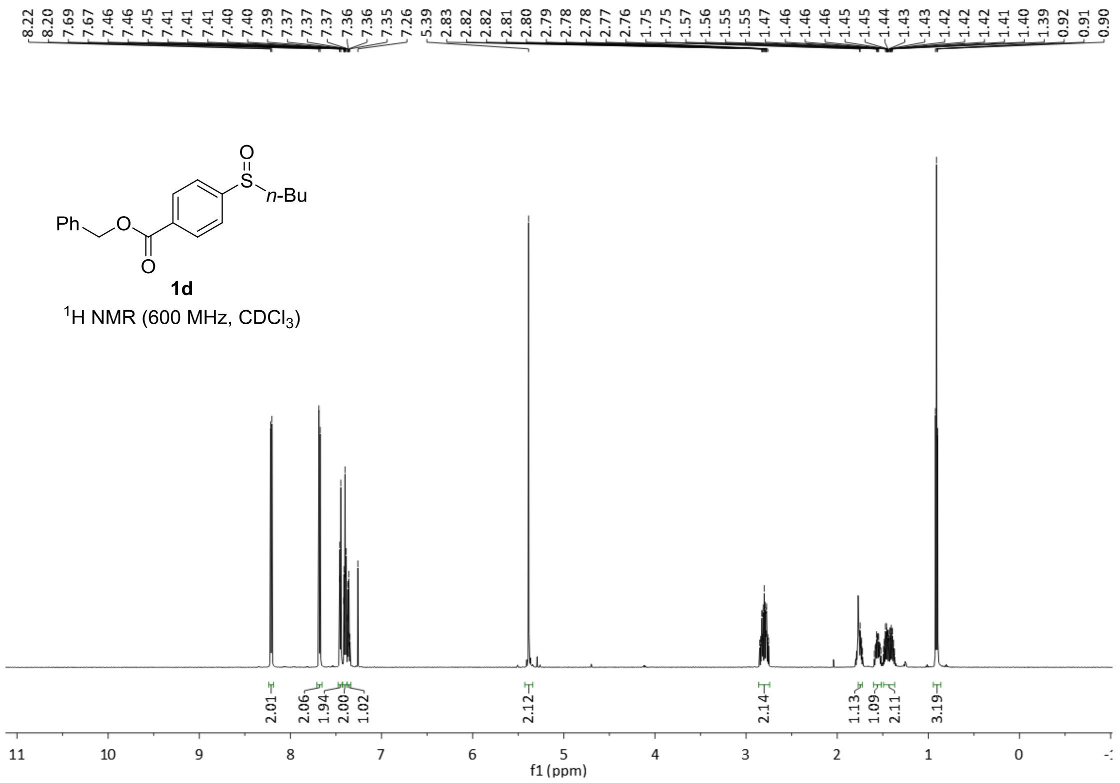

Supplementary Figure 39. ^{1}H NMR spectrum of **1d**

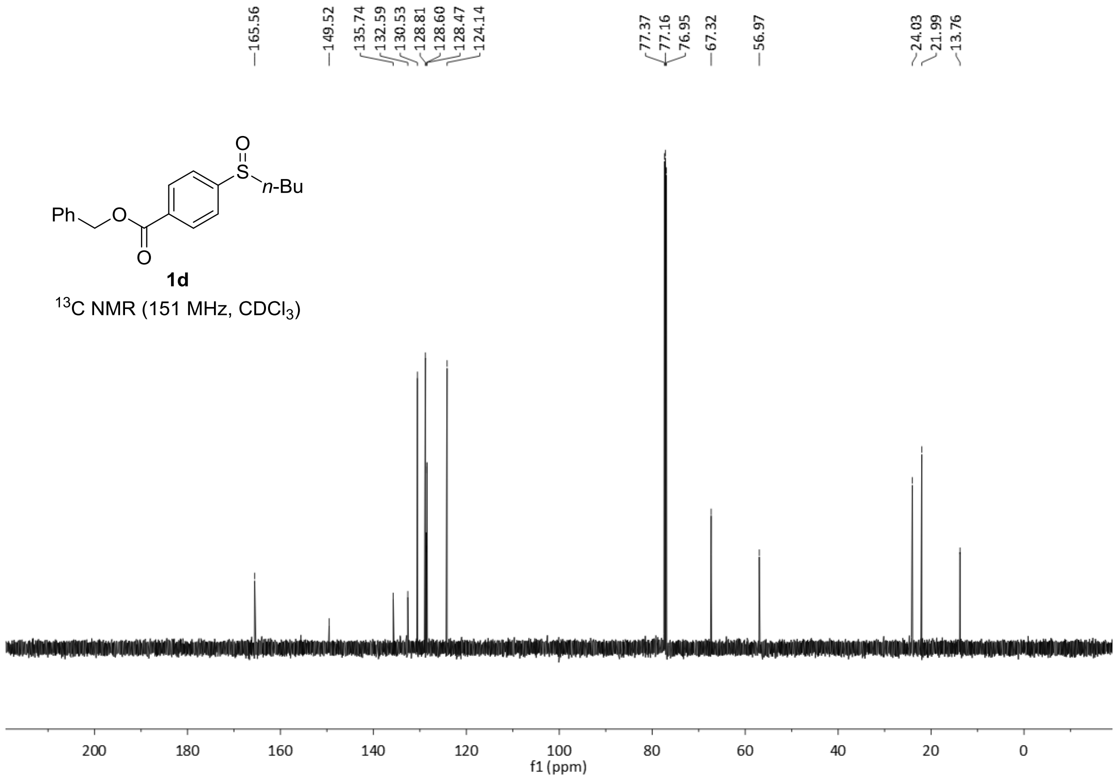

Supplementary Figure 40. ^{13}C NMR spectrum of **1d**

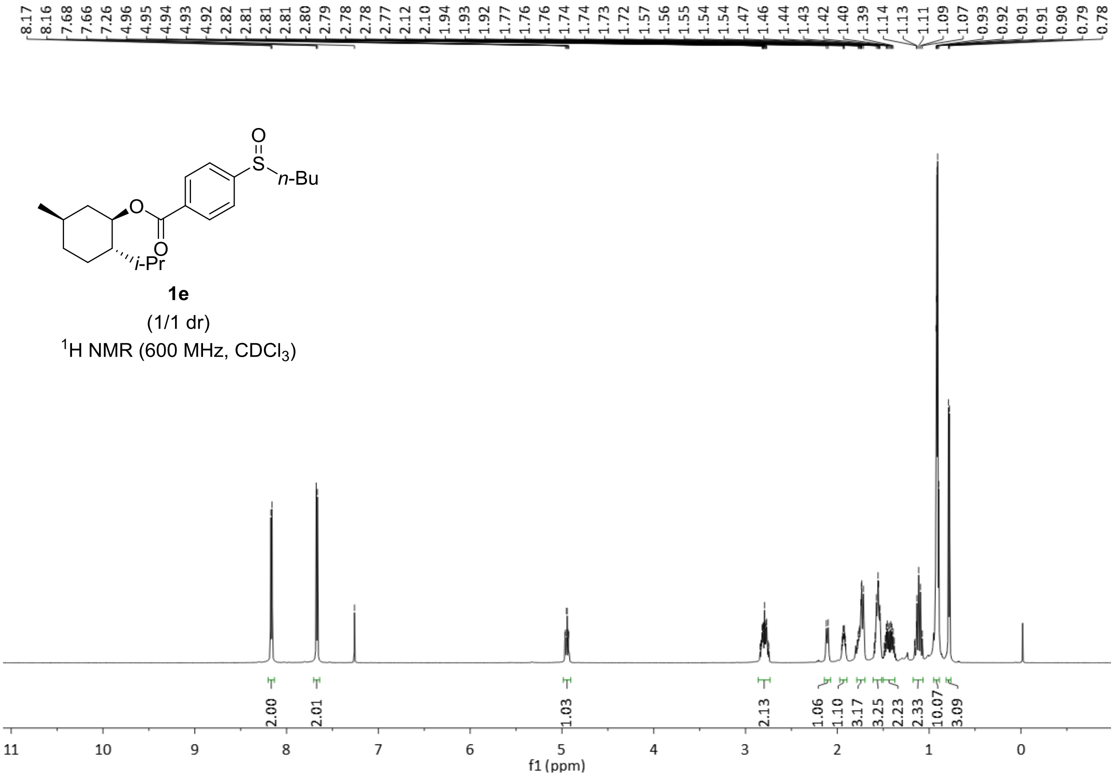

Supplementary Figure 41. ^{1}H NMR spectrum of **1e**

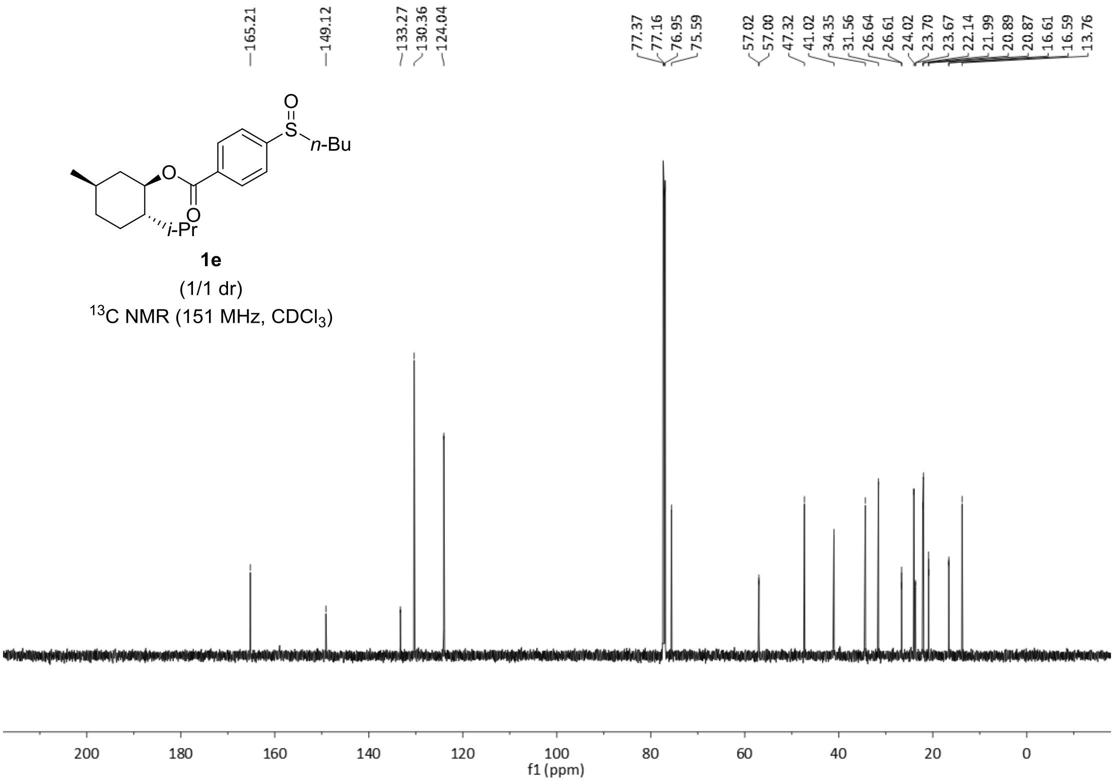

Supplementary Figure 42. ^{13}C NMR spectrum of **1e**

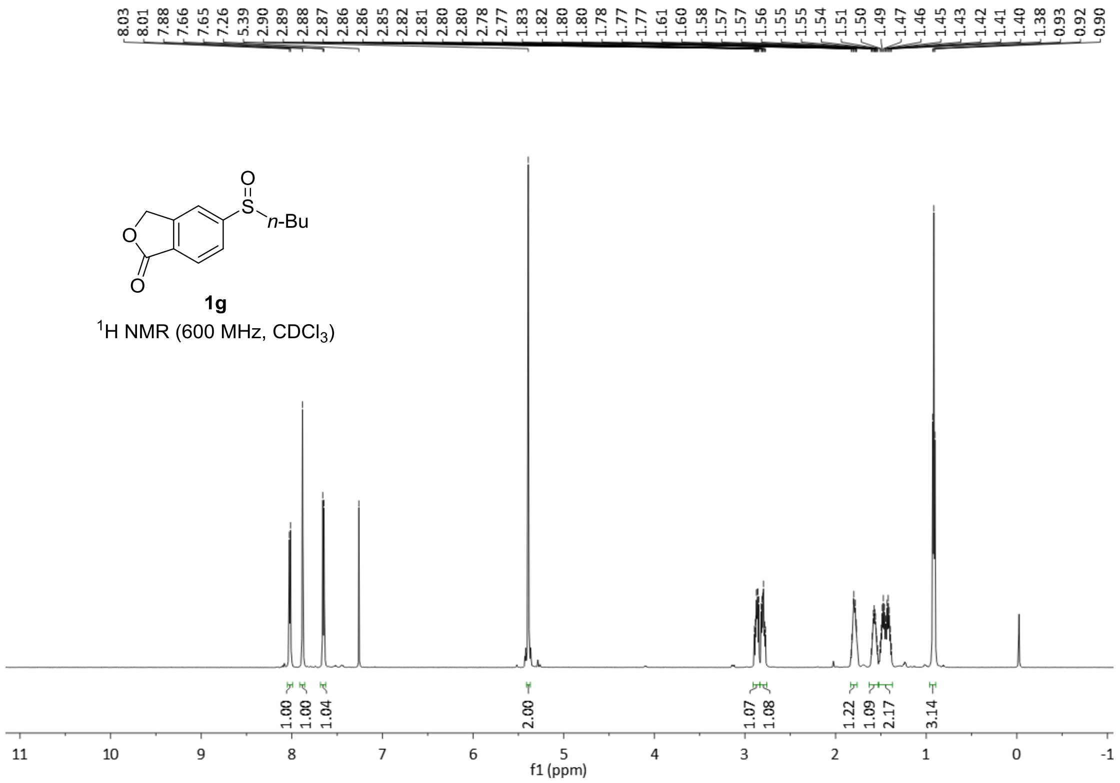

Supplementary Figure 43. ^{1}H NMR spectrum of **1g**

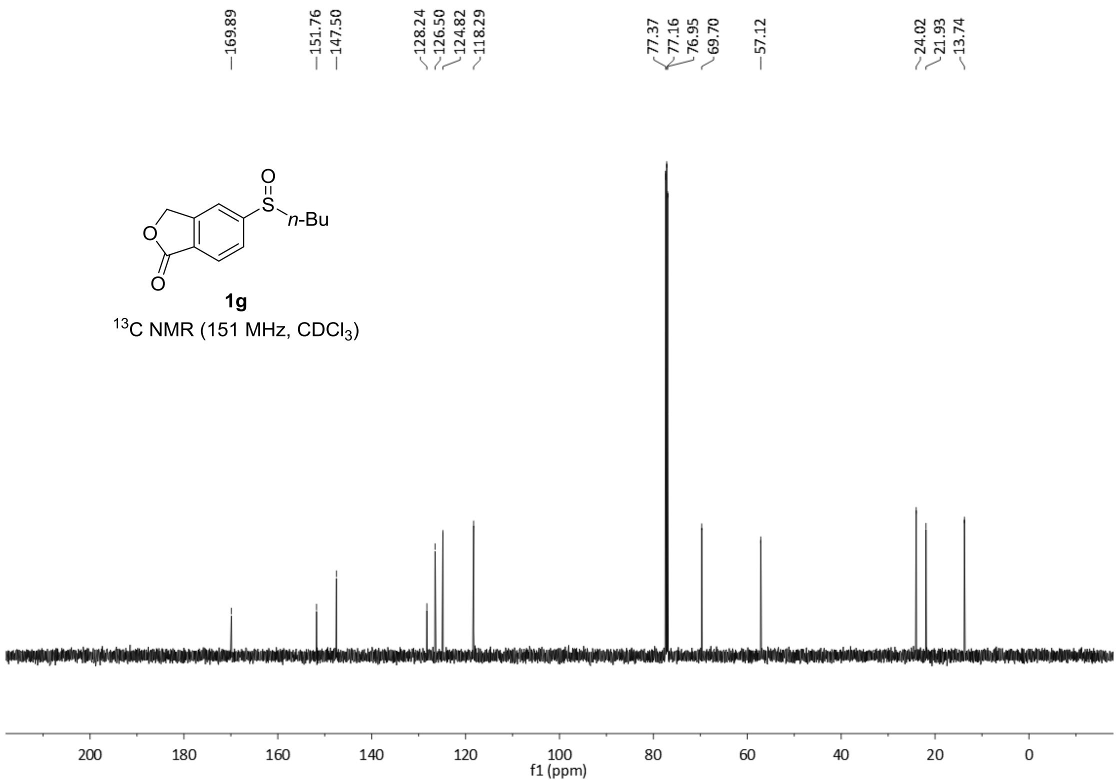

Supplementary Figure 44. ^{13}C NMR spectrum of **1g**

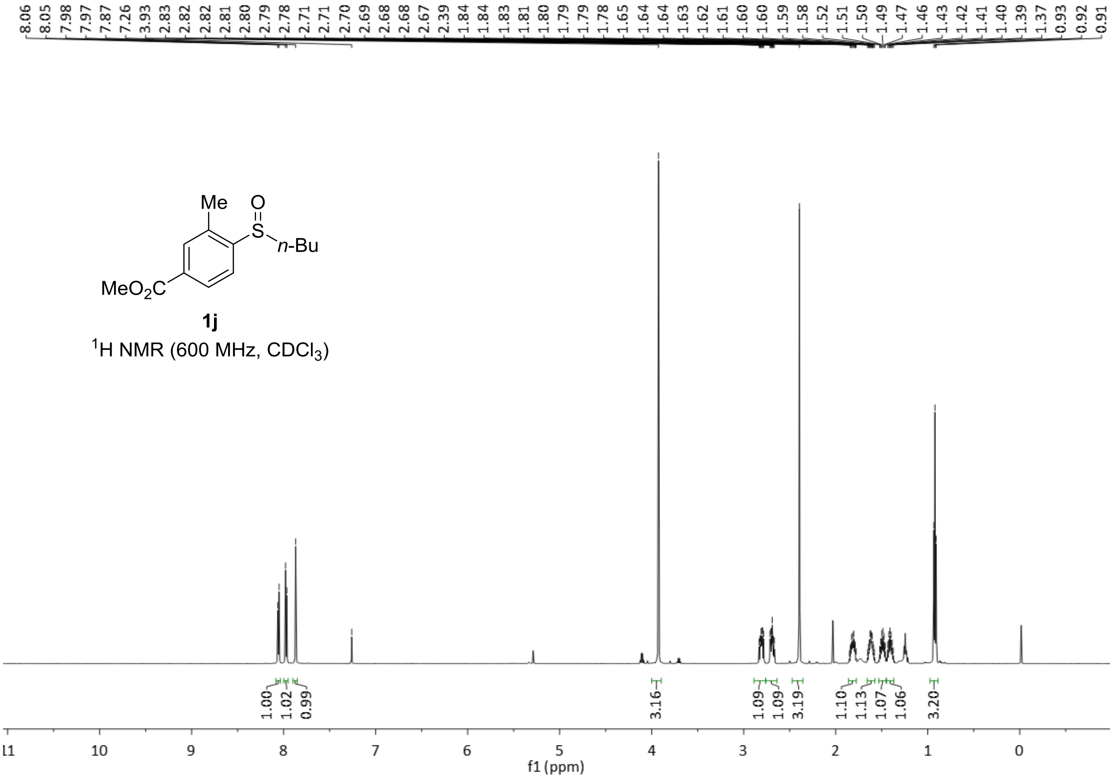

Supplementary Figure 45. ^{1}H NMR spectrum of **1j**

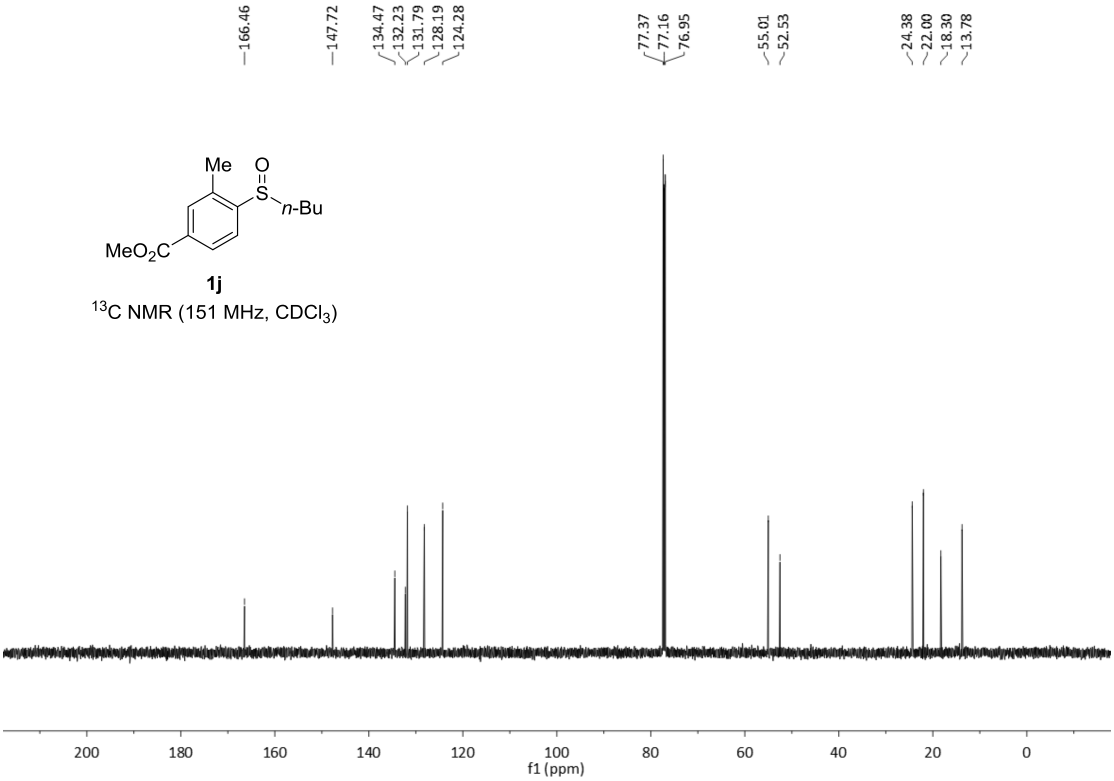

Supplementary Figure 46. ^{13}C NMR spectrum of **1j**

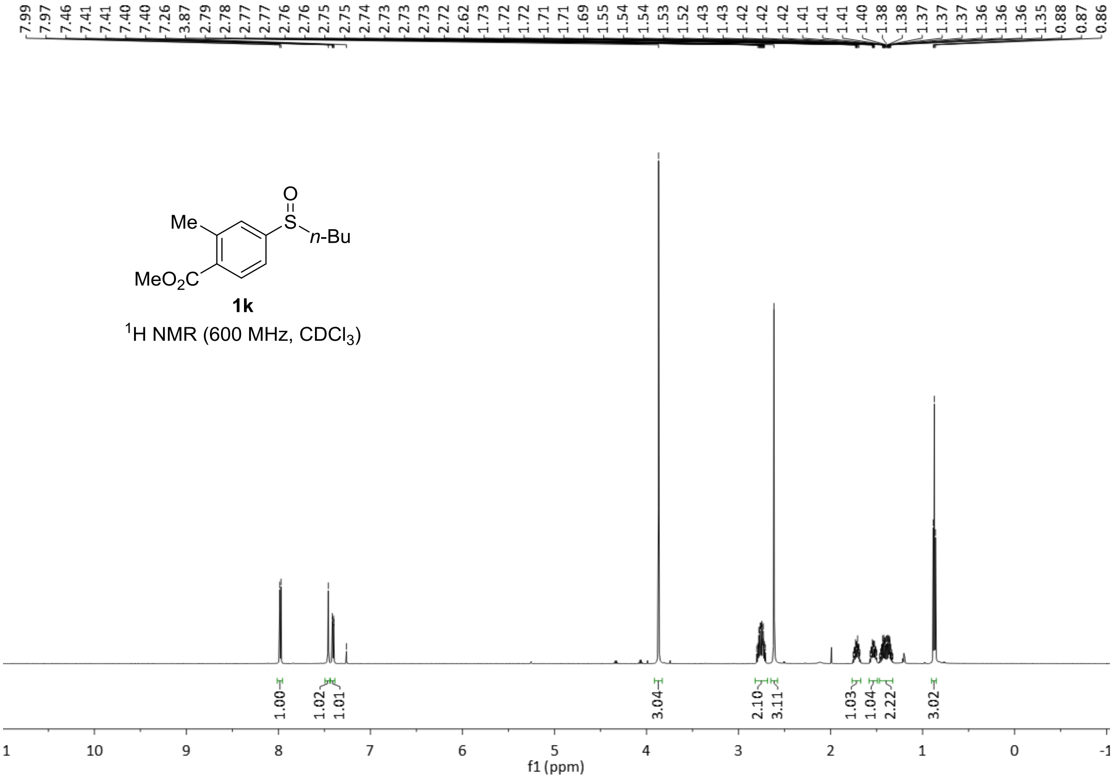

Supplementary Figure 47. ^{1}H NMR spectrum of **1k**

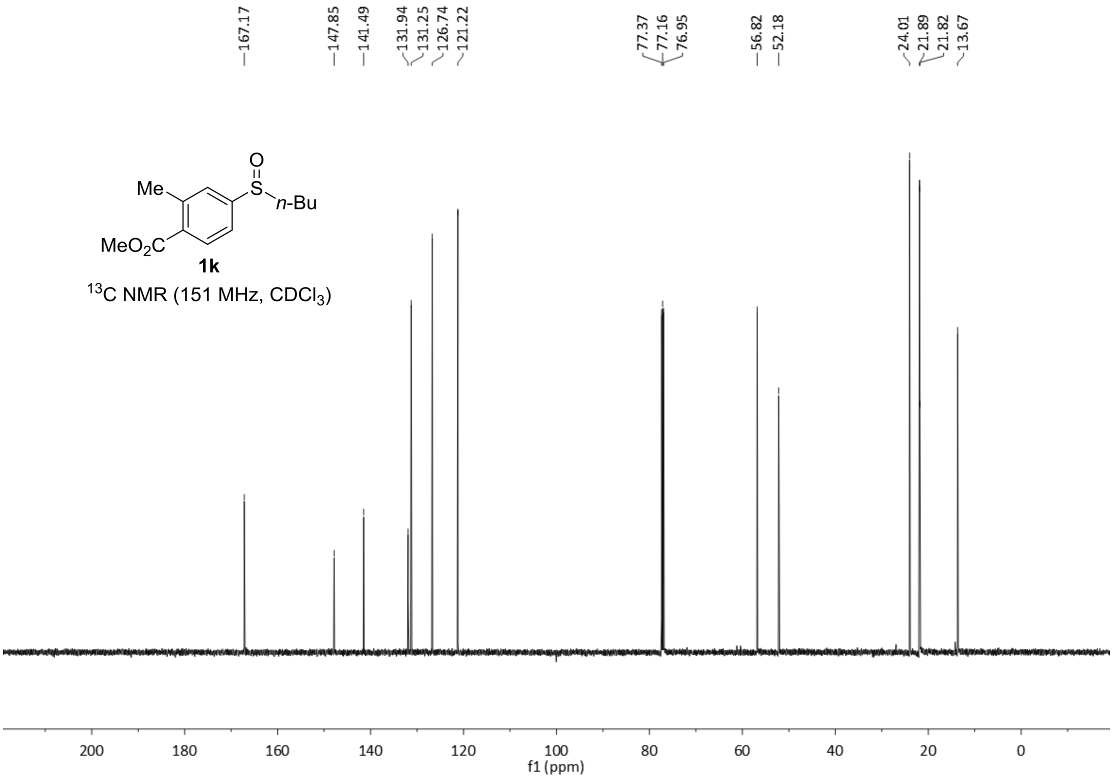

Supplementary Figure 48. ^{13}C NMR spectrum of **1k**

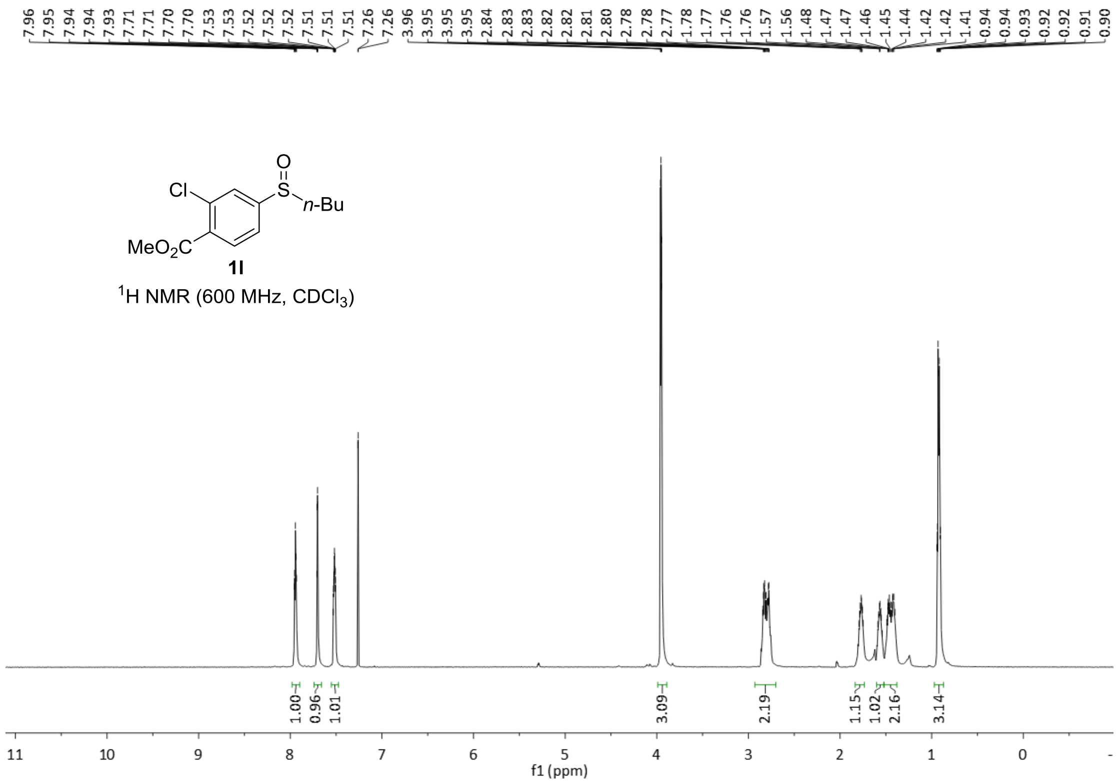

Supplementary Figure 49. ^{1}H NMR spectrum of **1l**

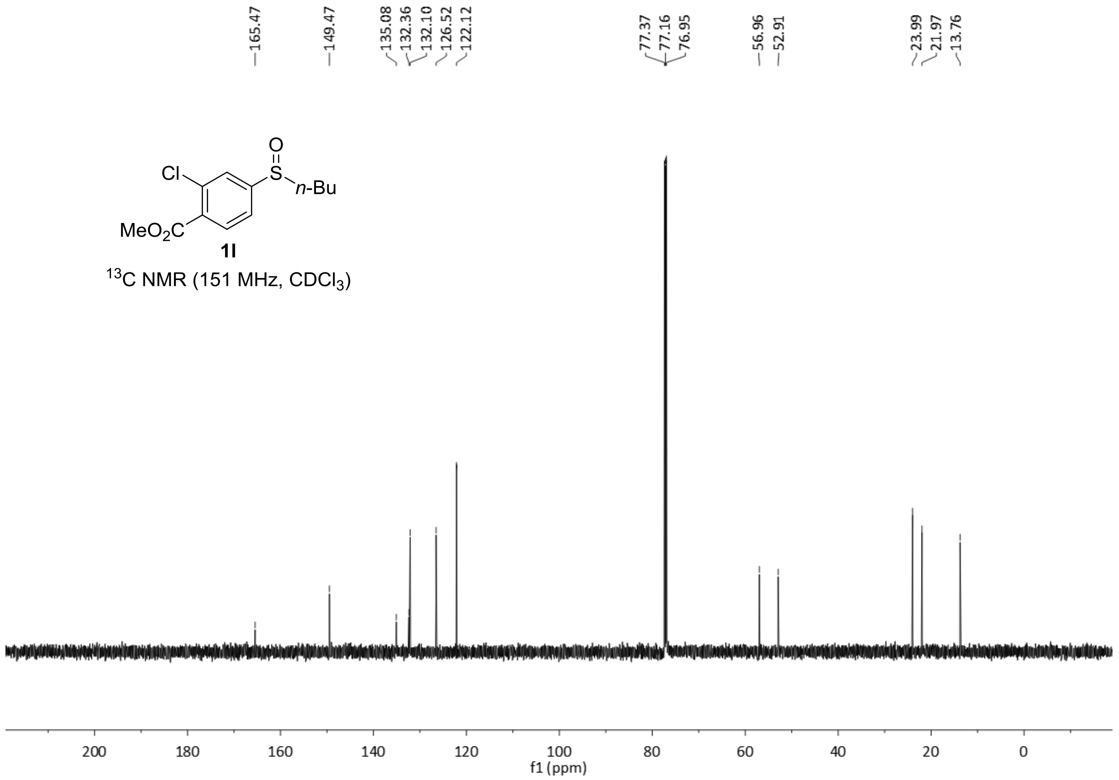

Supplementary Figure 50. ^{13}C NMR spectrum of **1l**

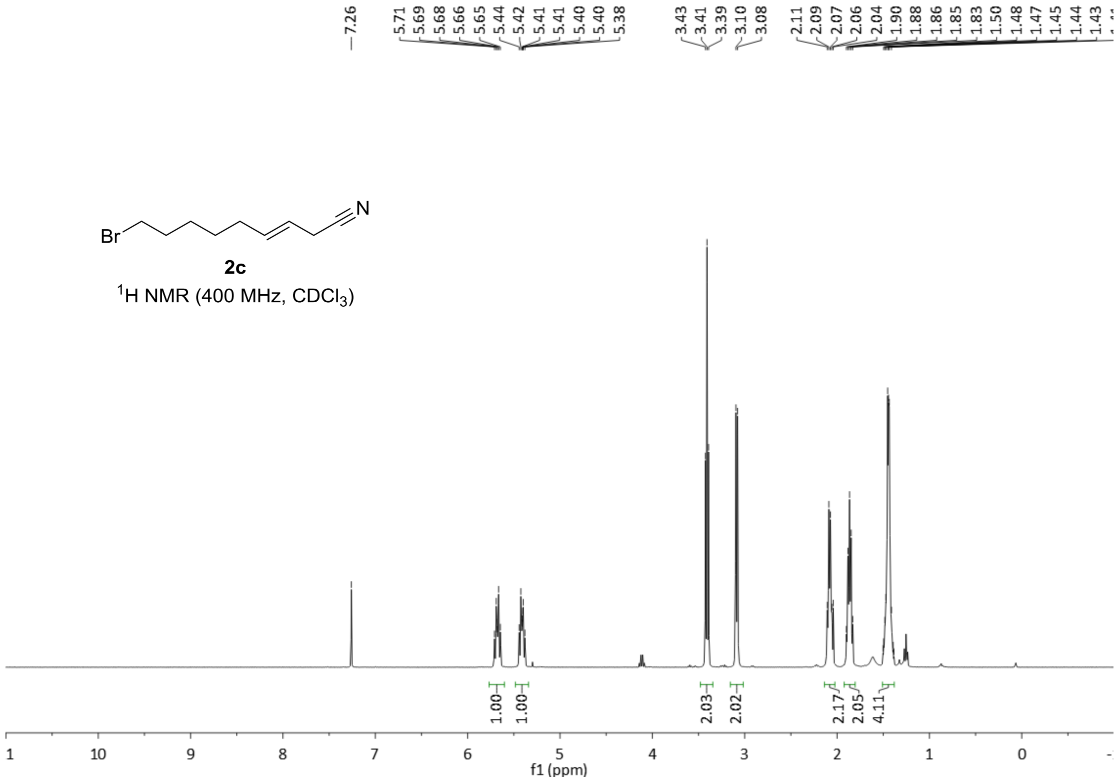

Supplementary Figure 51. ^{1}H NMR spectrum of **2c**

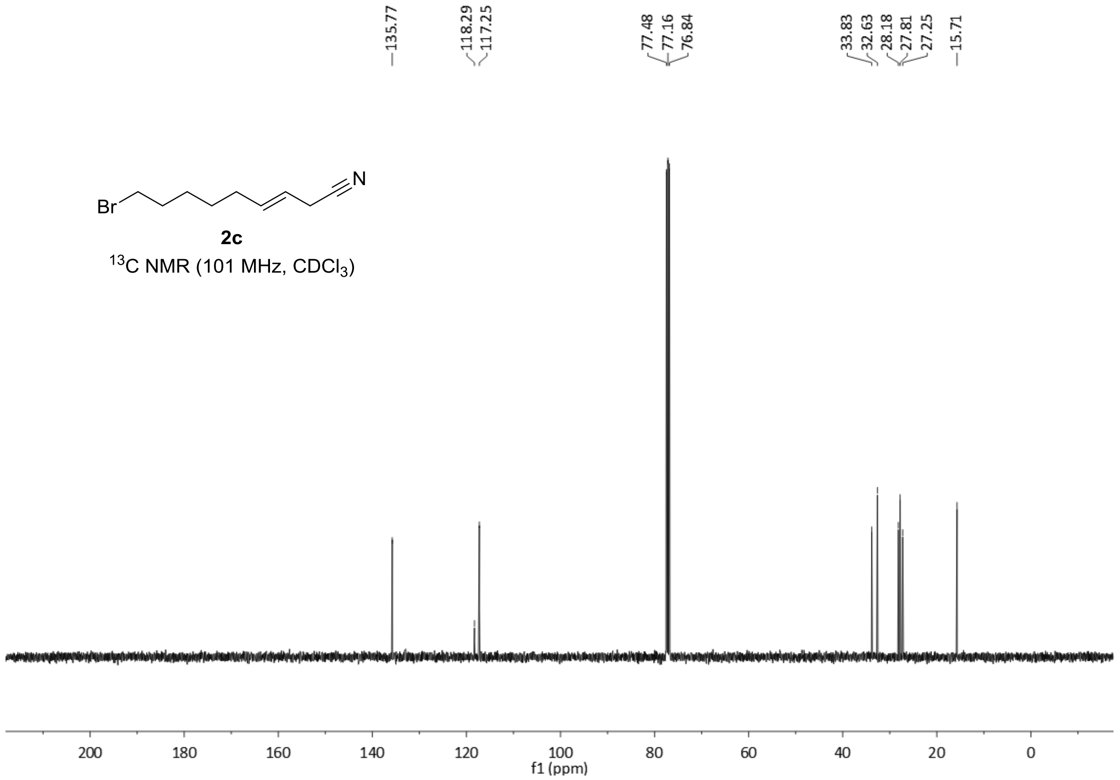

Supplementary Figure 52. ^{13}C NMR spectrum of **2c**

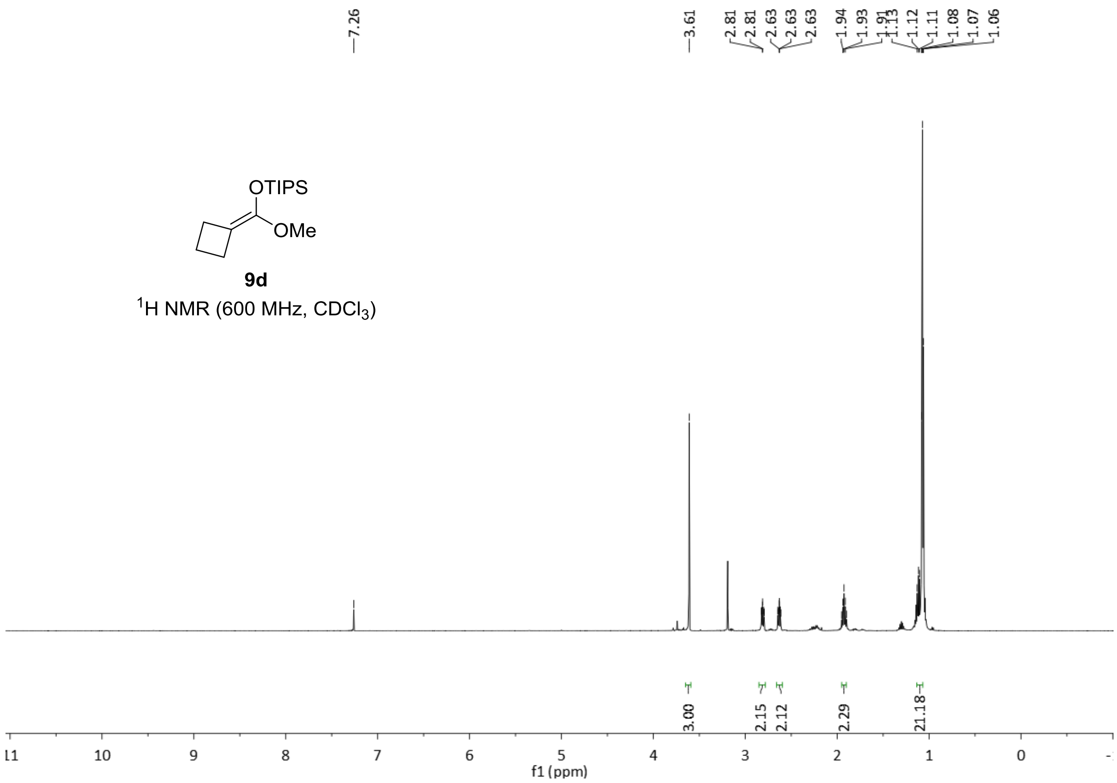

Supplementary Figure 53. ^{1}H NMR spectrum of **9d**

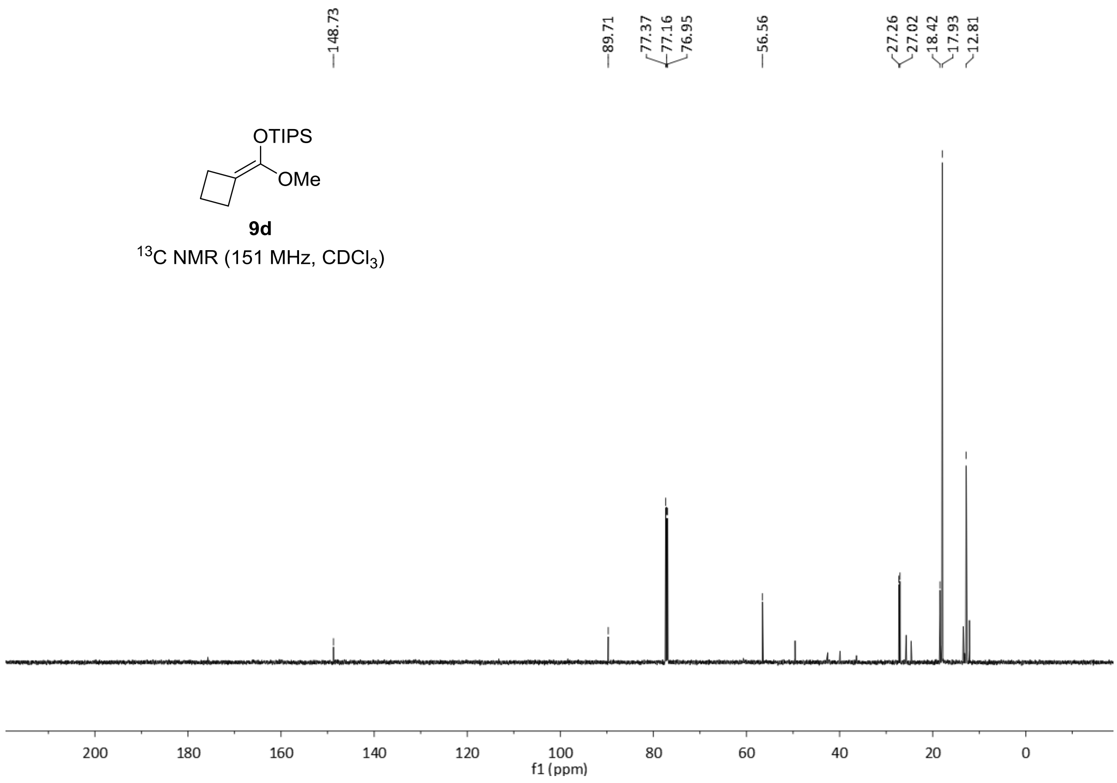

Supplementary Figure 54. ^{13}C NMR spectrum of **9d**

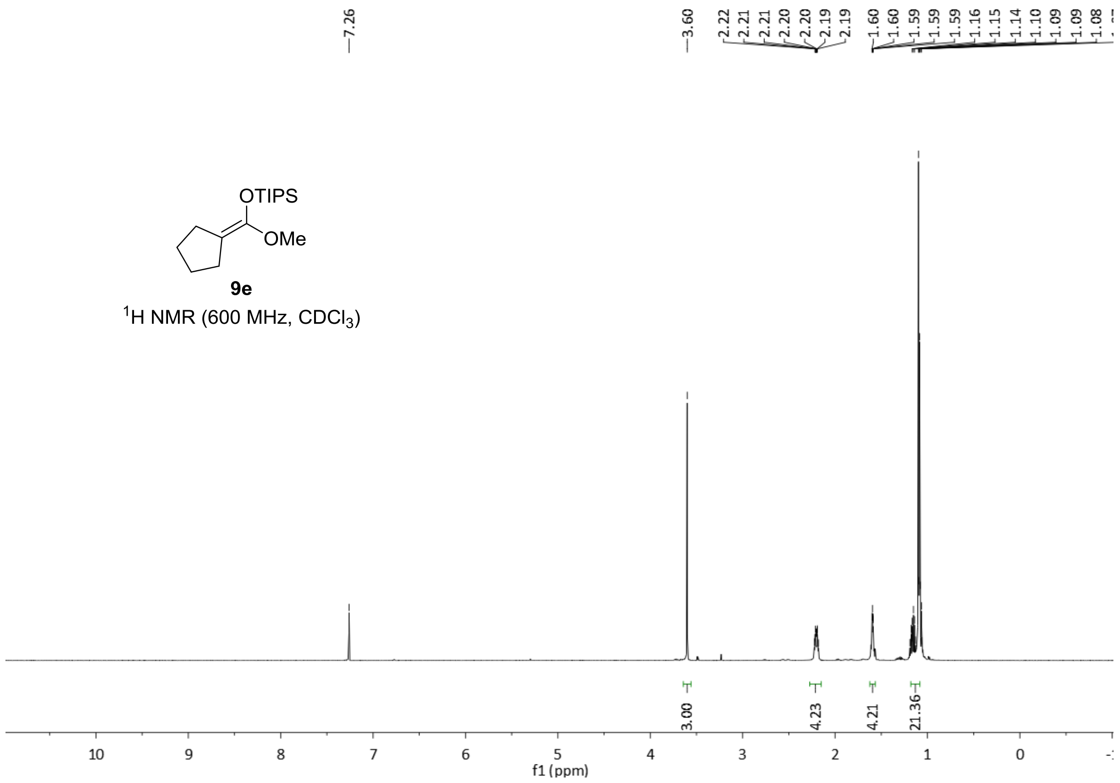

Supplementary Figure 55. ^{1}H NMR spectrum of **9e**

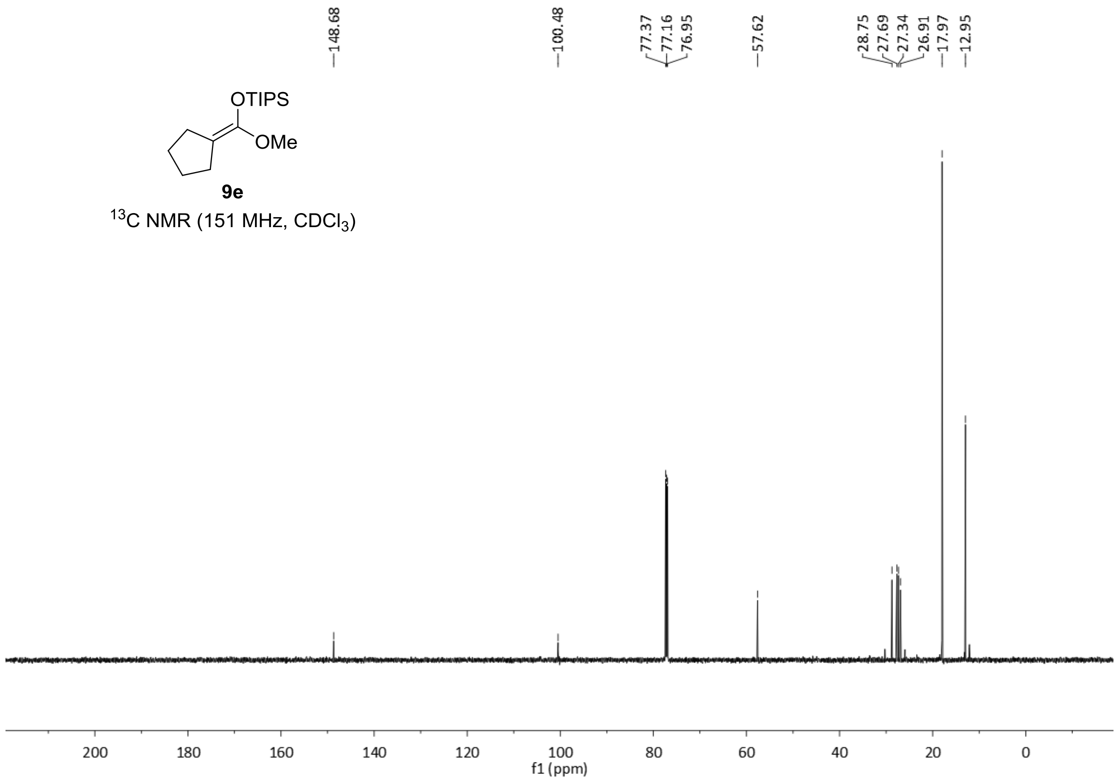

Supplementary Figure 56. ^{13}C NMR spectrum of **9e**

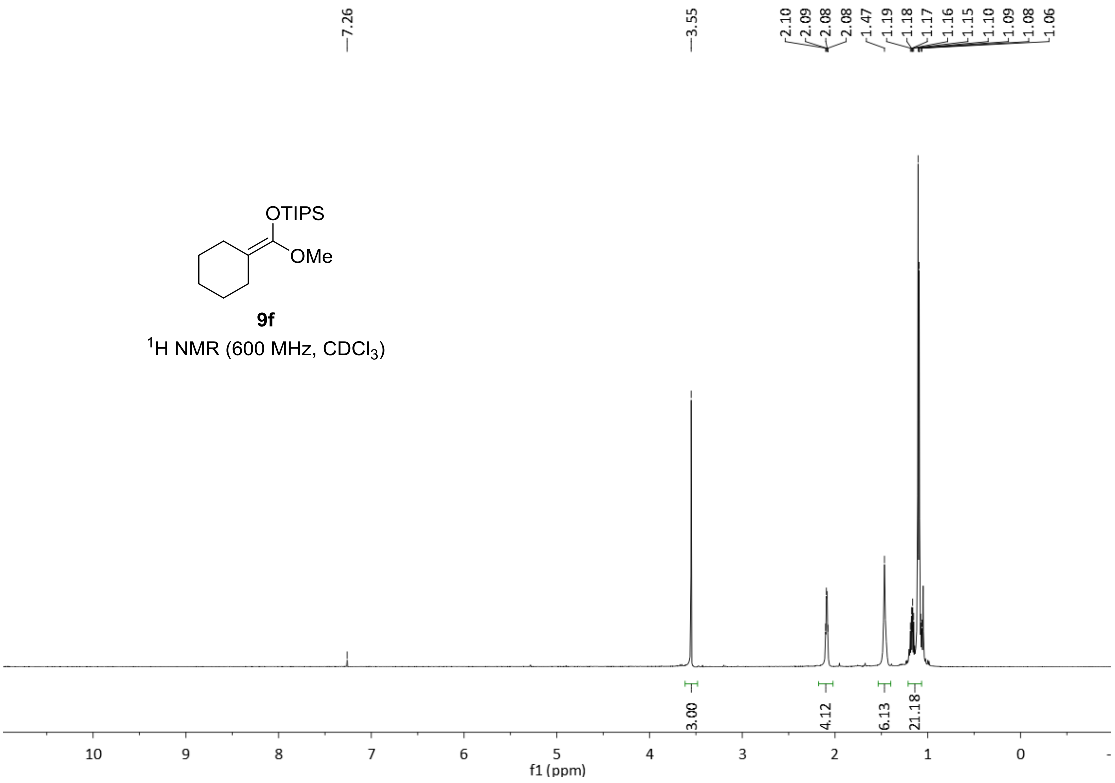

Supplementary Figure 57. ^{1}H NMR spectrum of **9f**

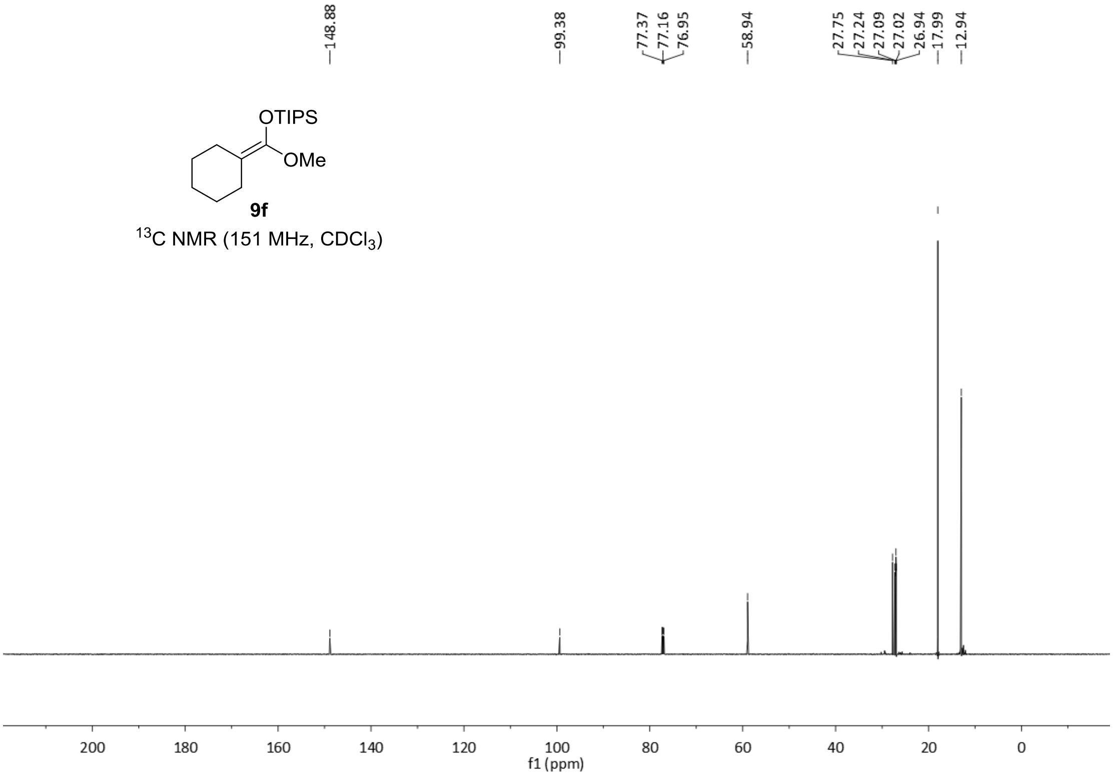

Supplementary Figure 58. ^{13}C NMR spectrum of **9f**

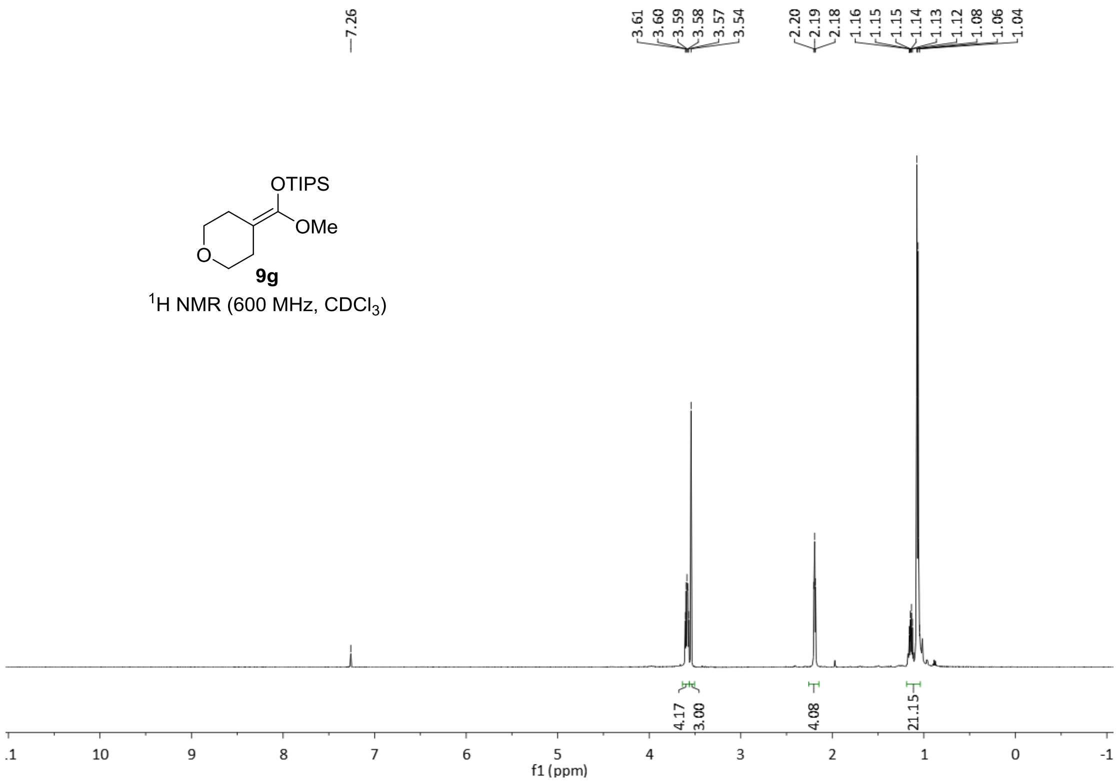

Supplementary Figure 59. ^{1}H NMR spectrum of **9g**

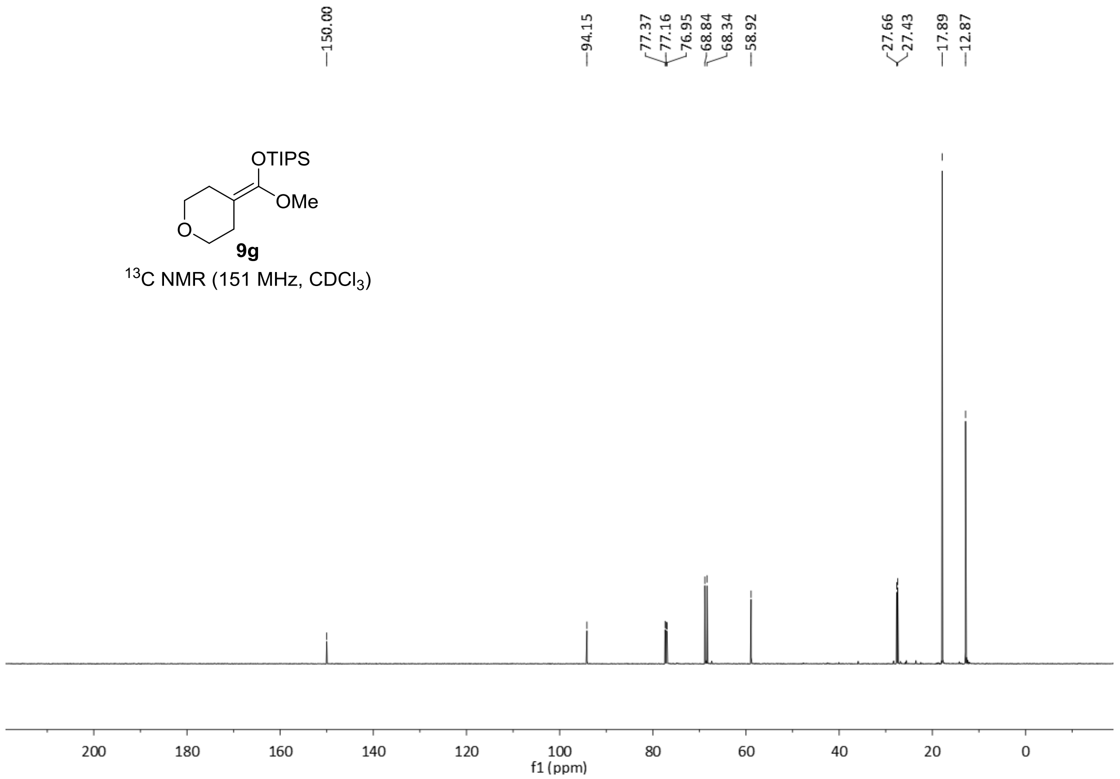

Supplementary Figure 60. ^{13}C NMR spectrum of **9g**

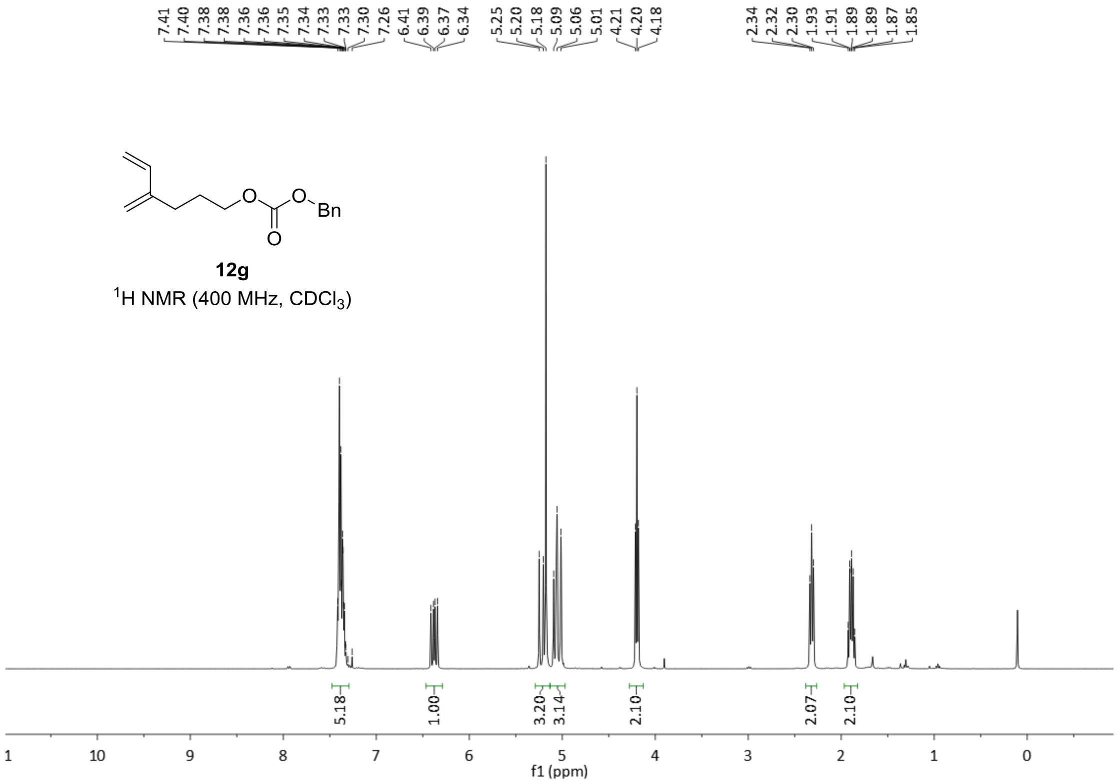

Supplementary Figure 61. ^{1}H NMR spectrum of **12g**

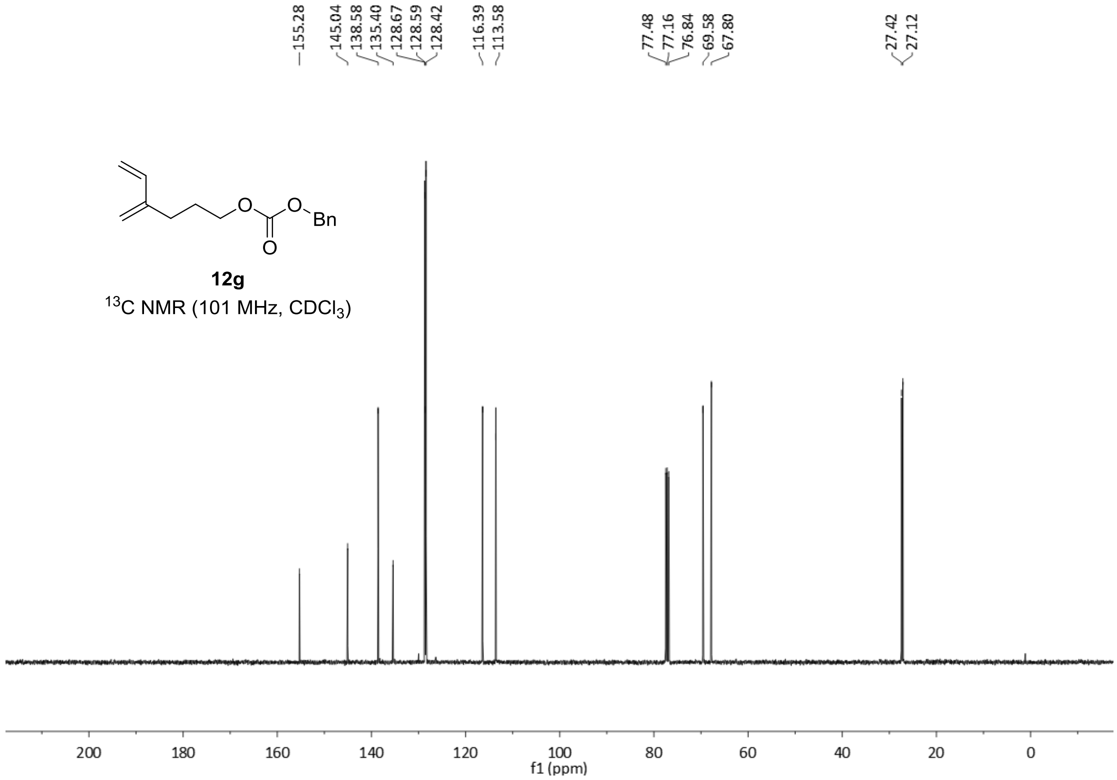

Supplementary Figure 62. ^{13}C NMR spectrum of **12g**

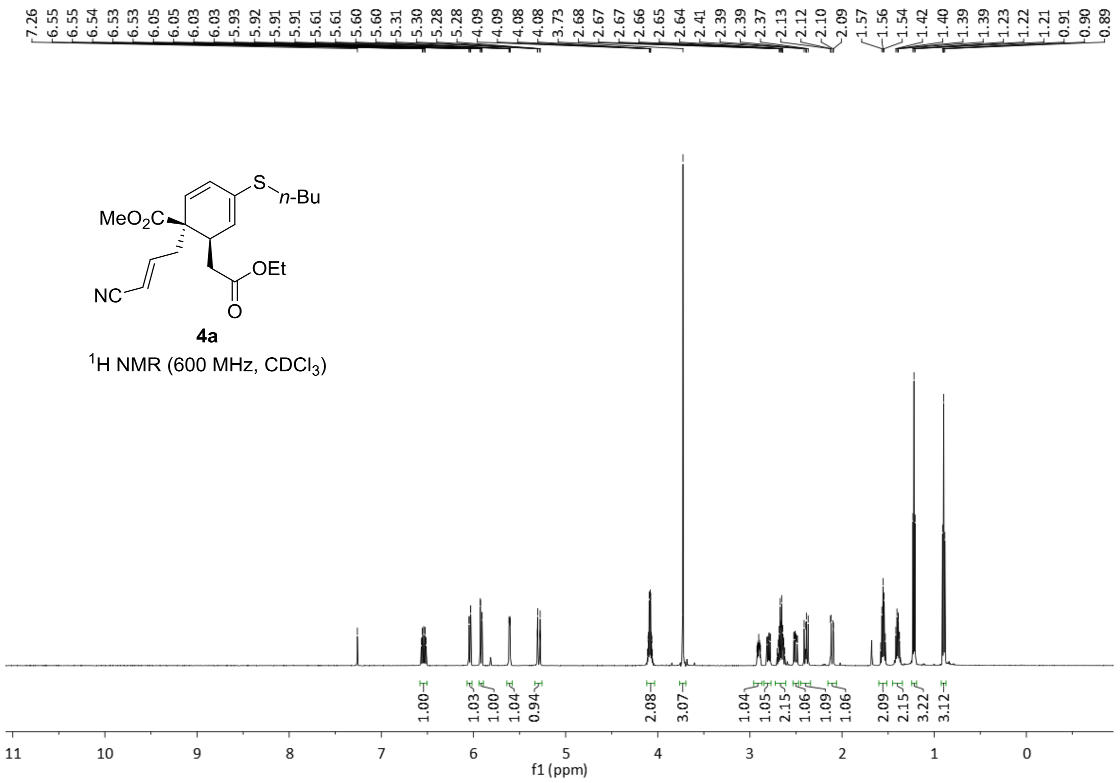

Supplementary Figure 63. ^{1}H NMR spectrum of **4a**

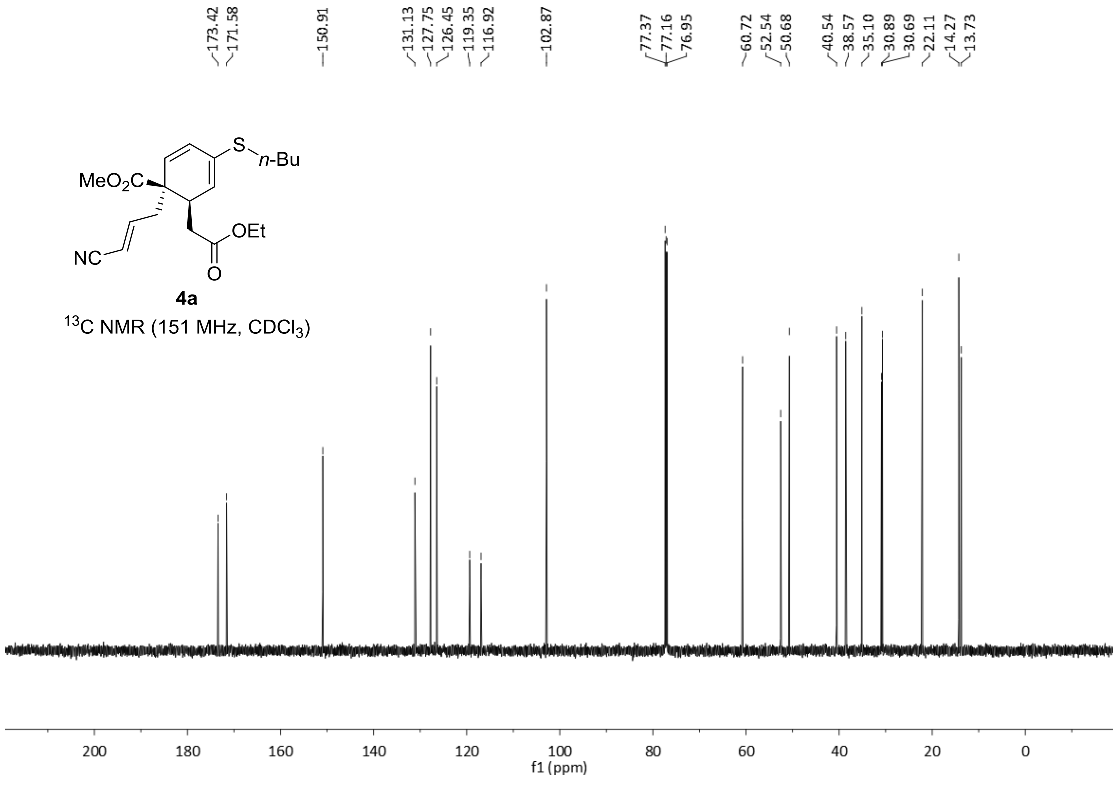

Supplementary Figure 64. ^{13}C NMR spectrum of **4a**

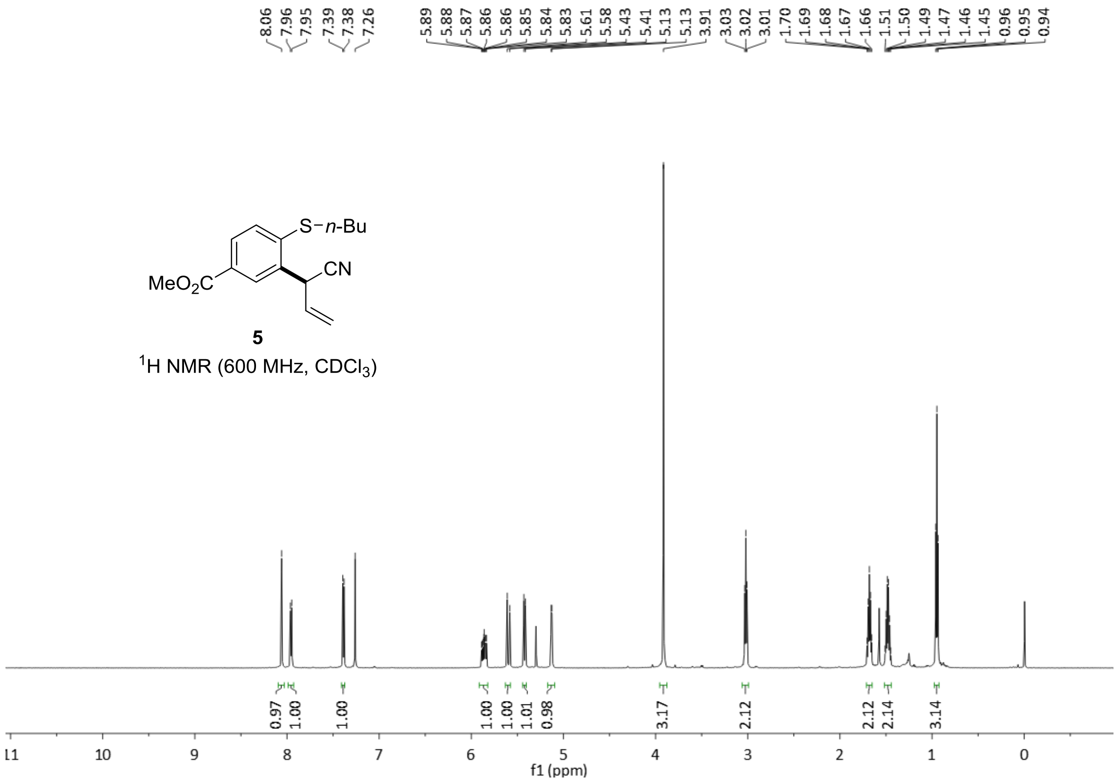

Supplementary Figure 65. ^{1}H NMR spectrum of **5**

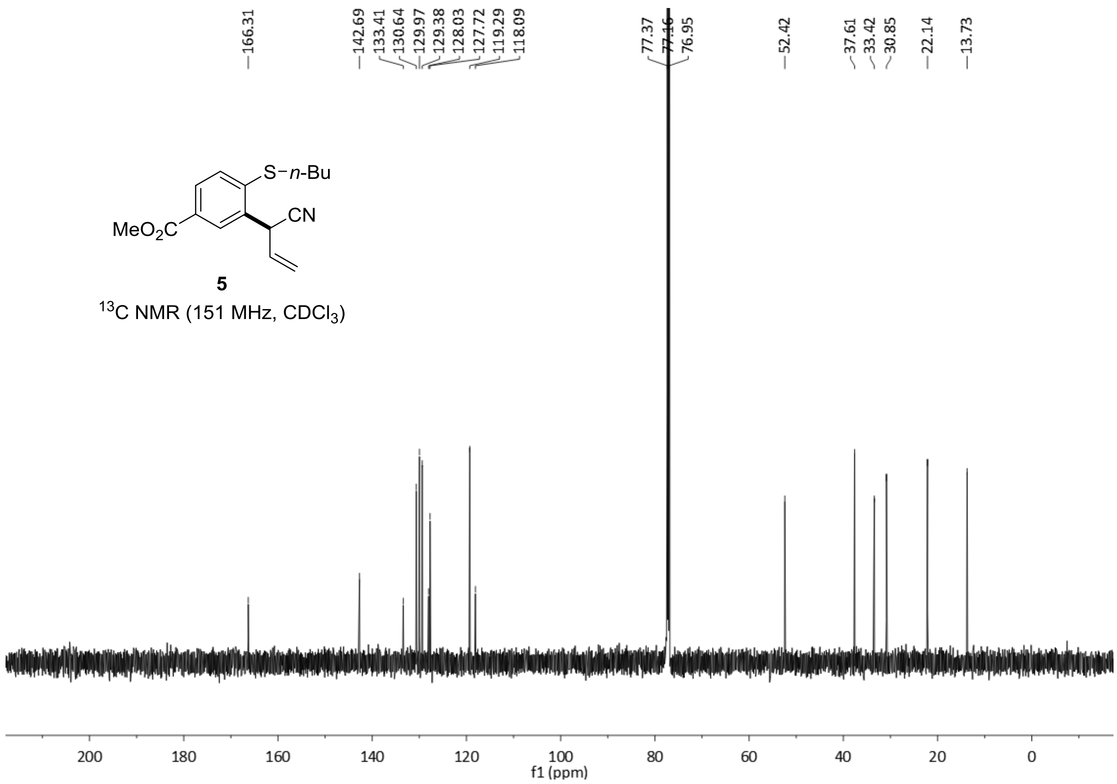

Supplementary Figure 66. ^{13}C NMR spectrum of **5**

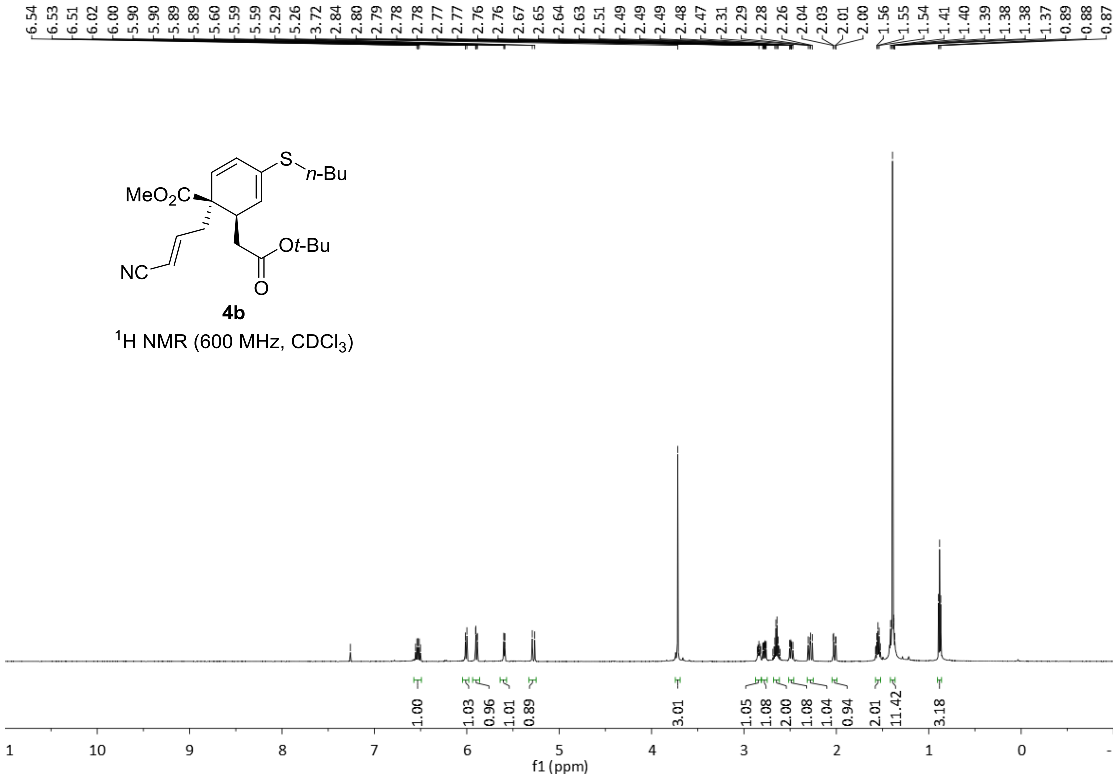

Supplementary Figure 67. ^{1}H NMR spectrum of **4b**

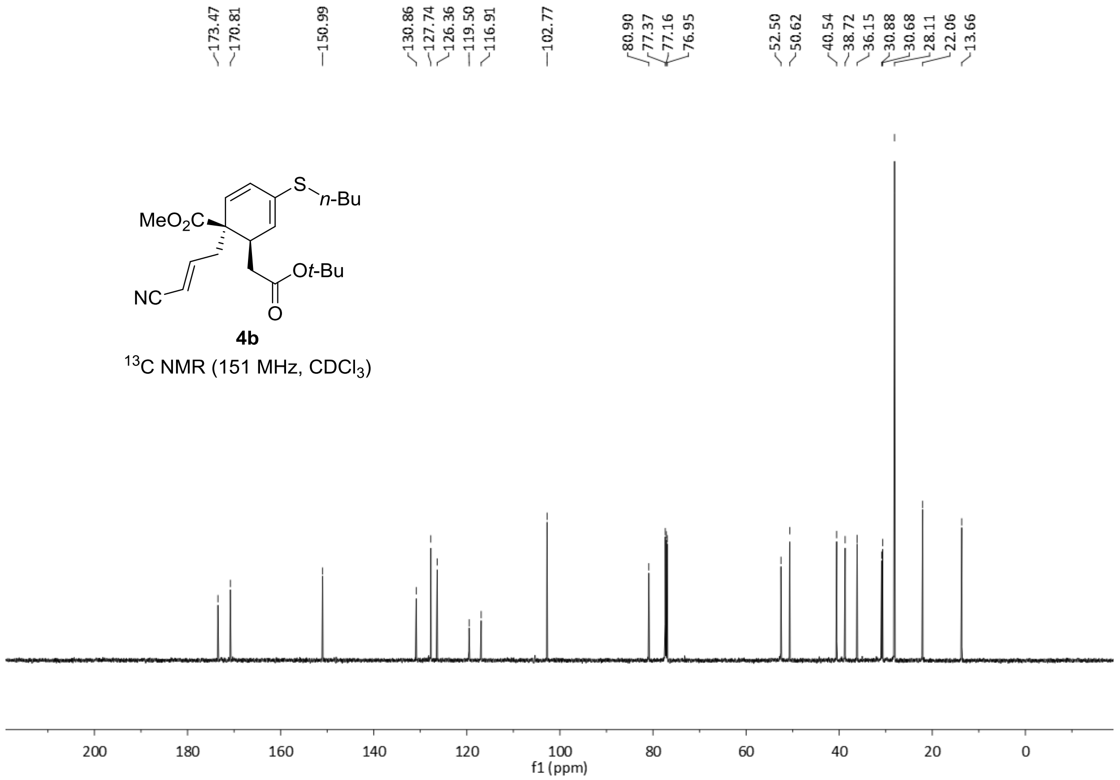

Supplementary Figure 68. ^{13}C NMR spectrum of **4b**

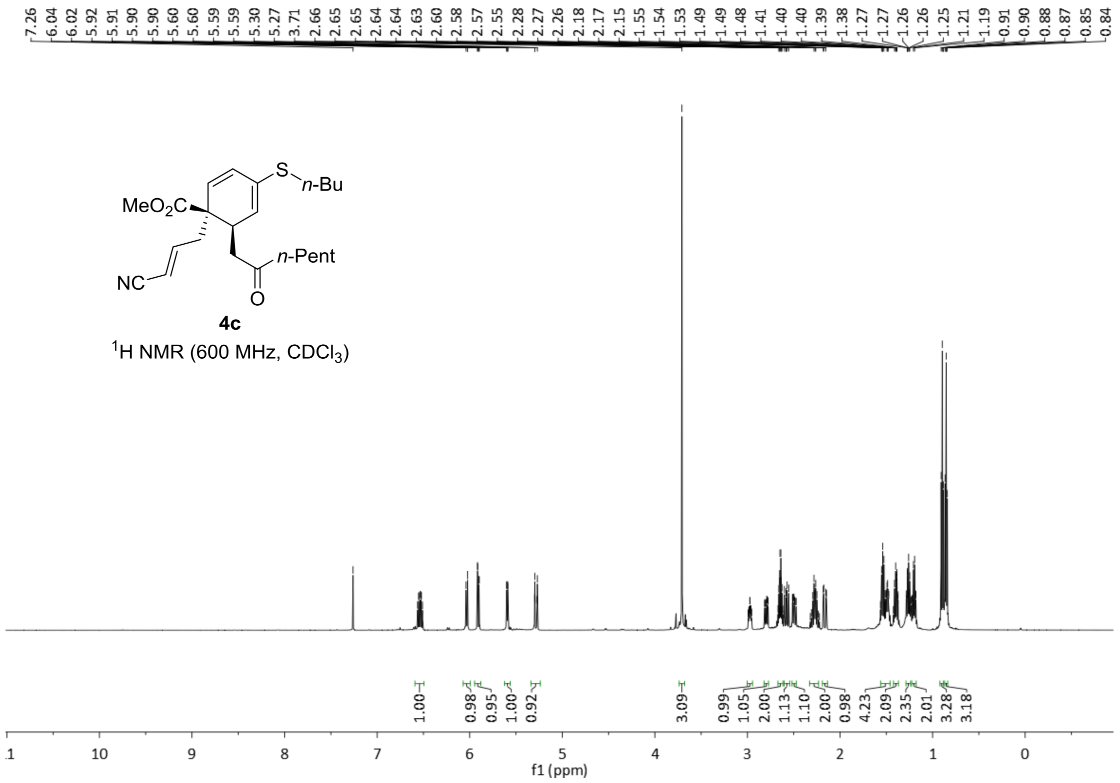

Supplementary Figure 69. ^{1}H NMR spectrum of **4c**

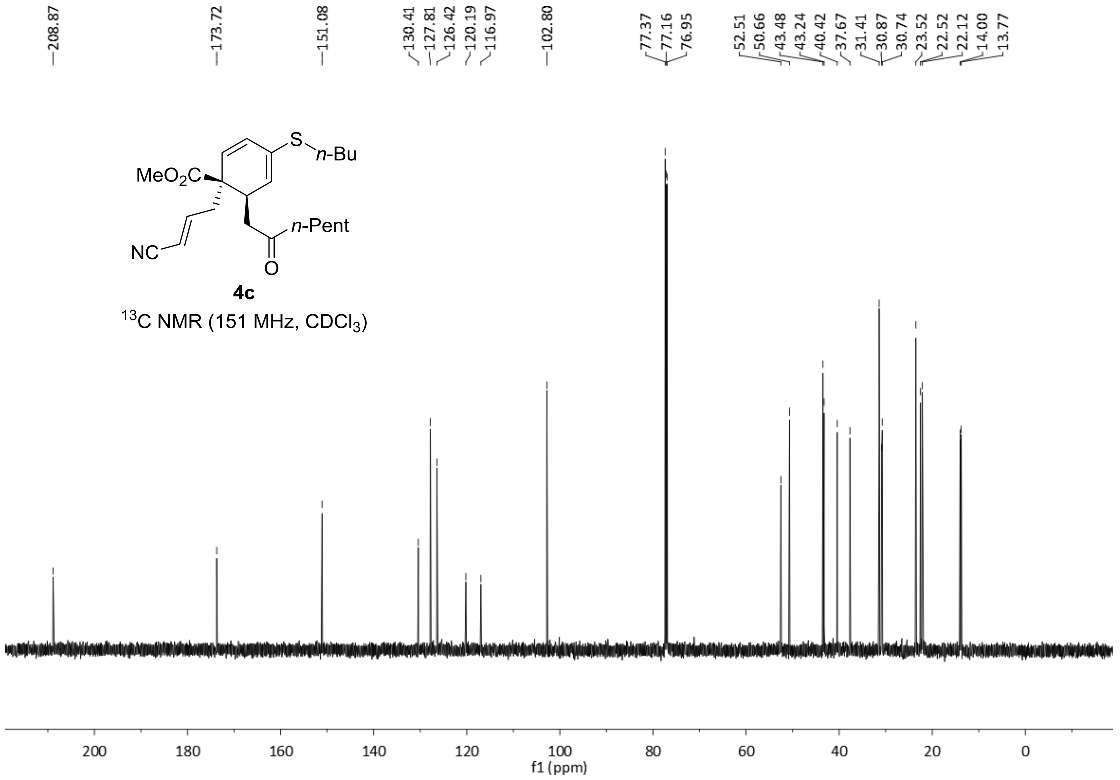

Supplementary Figure 70. ^{13}C NMR spectrum of **4c**

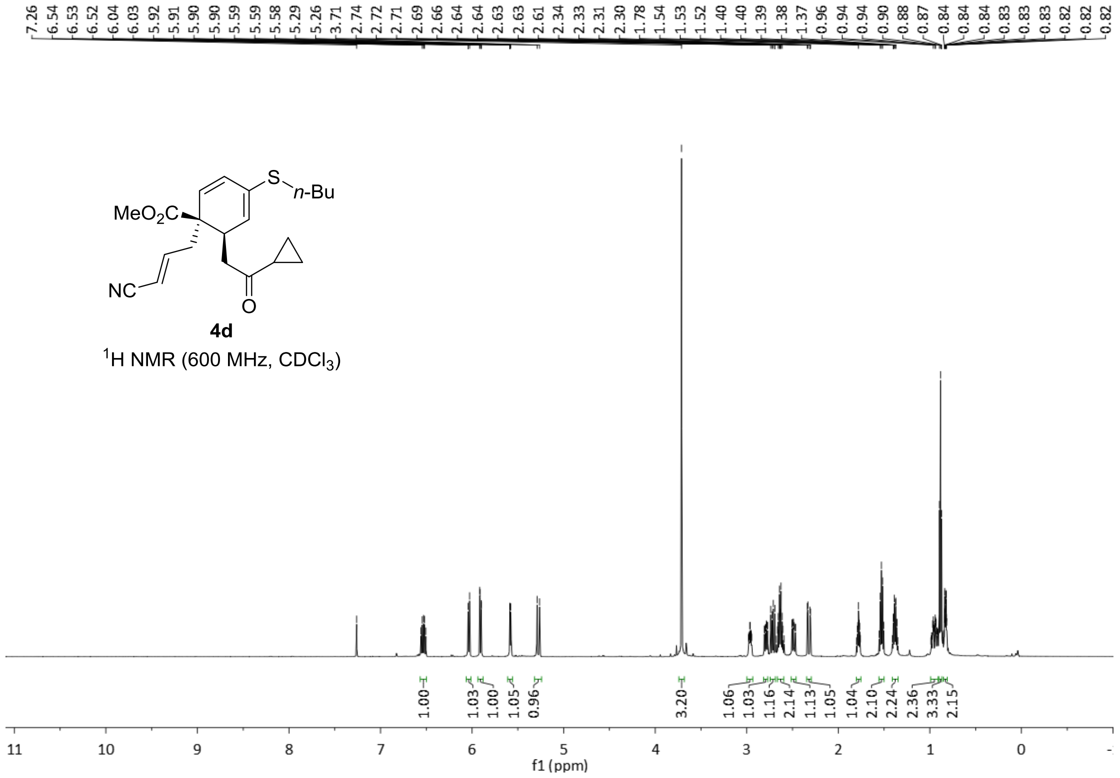

Supplementary Figure 71. ^{1}H NMR spectrum of **4d**

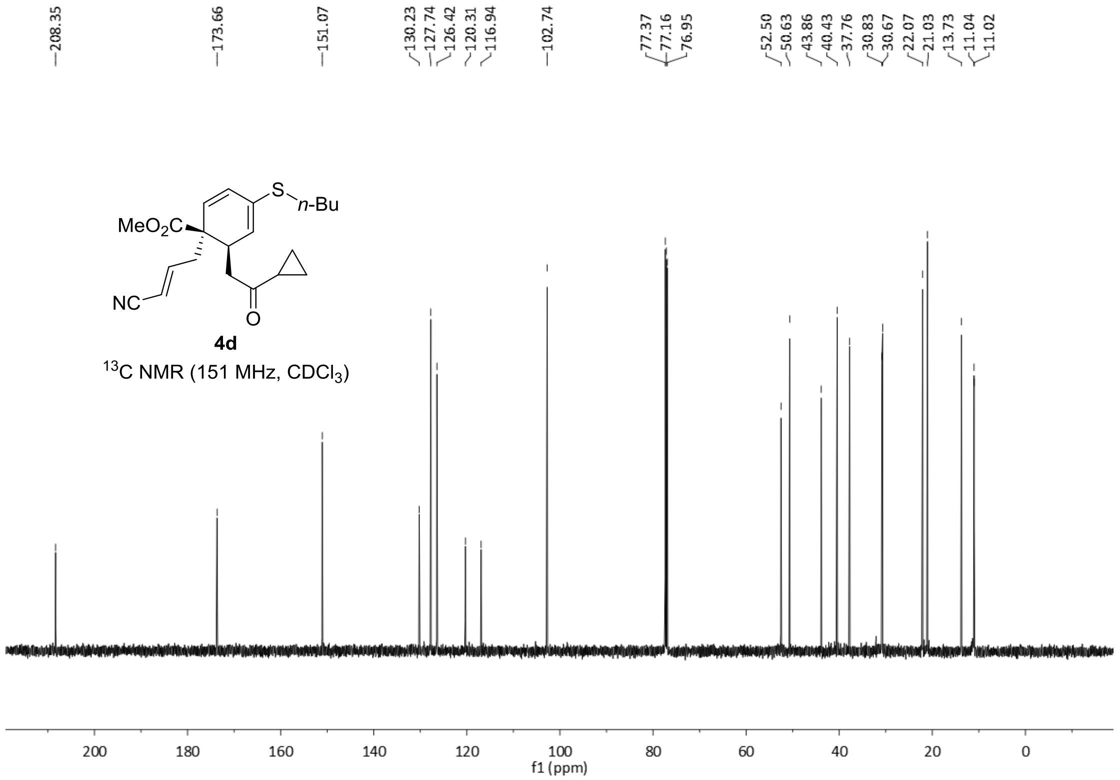

Supplementary Figure 72. ^{13}C NMR spectrum of **4d**

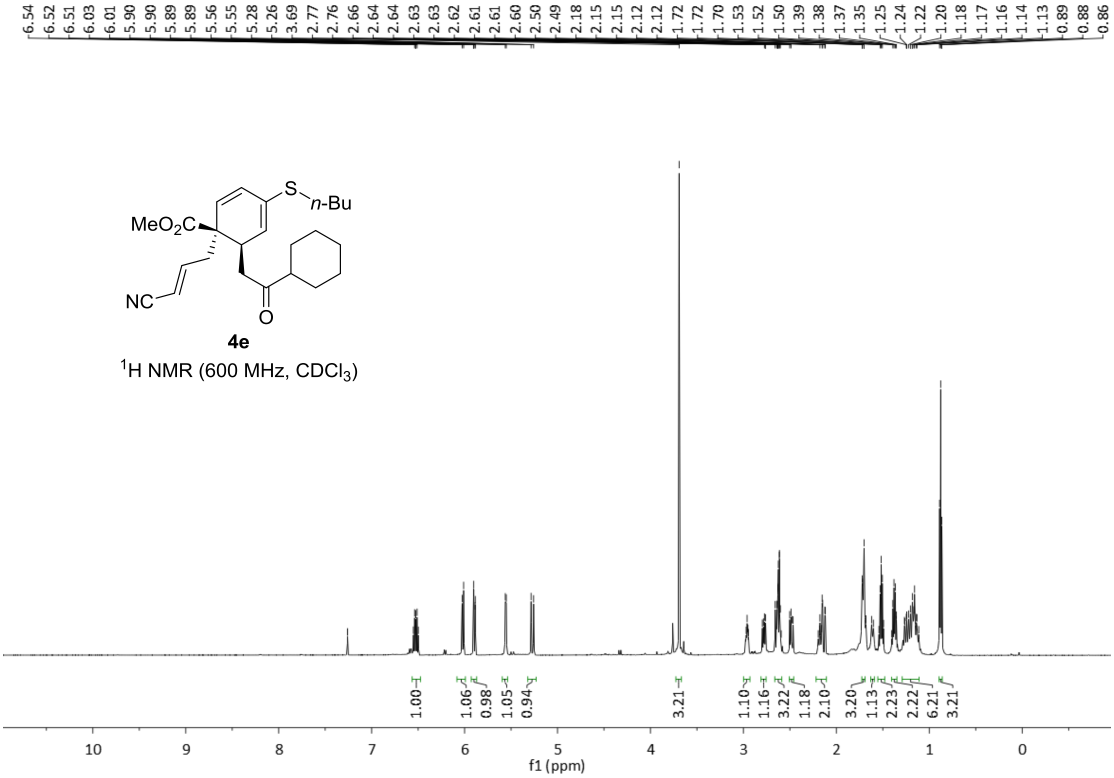

Supplementary Figure 73. ^{1}H NMR spectrum of **4e**

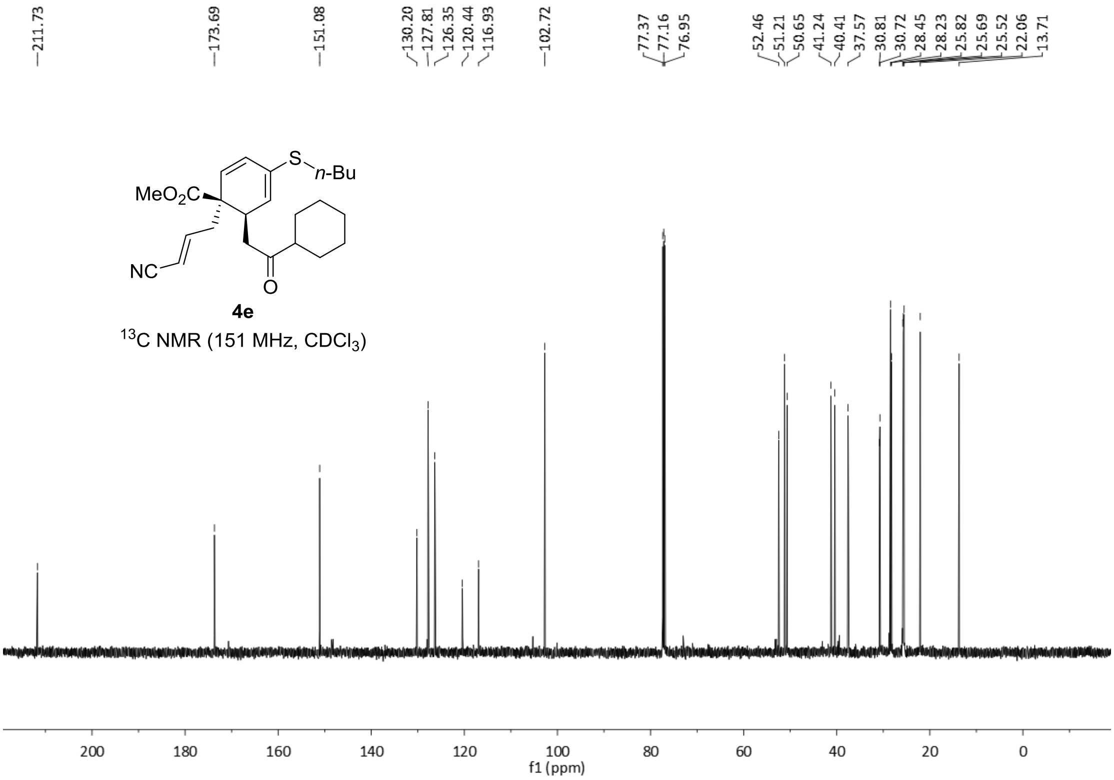

Supplementary Figure 74. ^{13}C NMR spectrum of **4e**

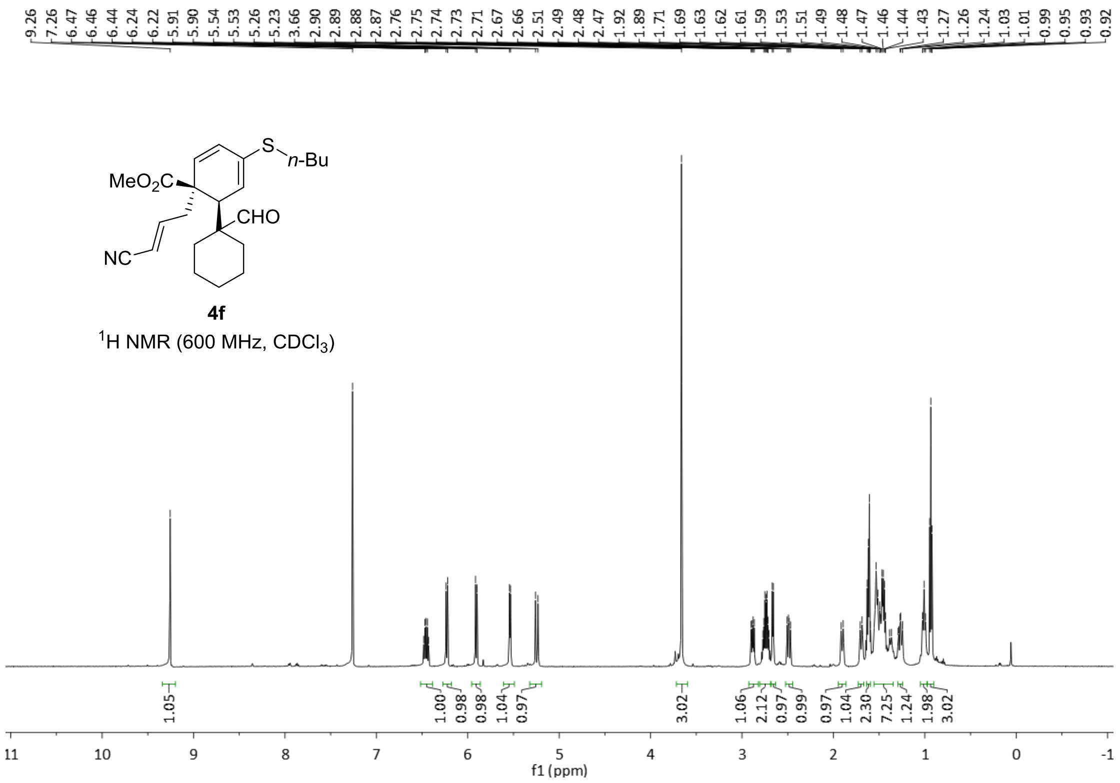

Supplementary Figure 75. ^{1}H NMR spectrum of **4f**

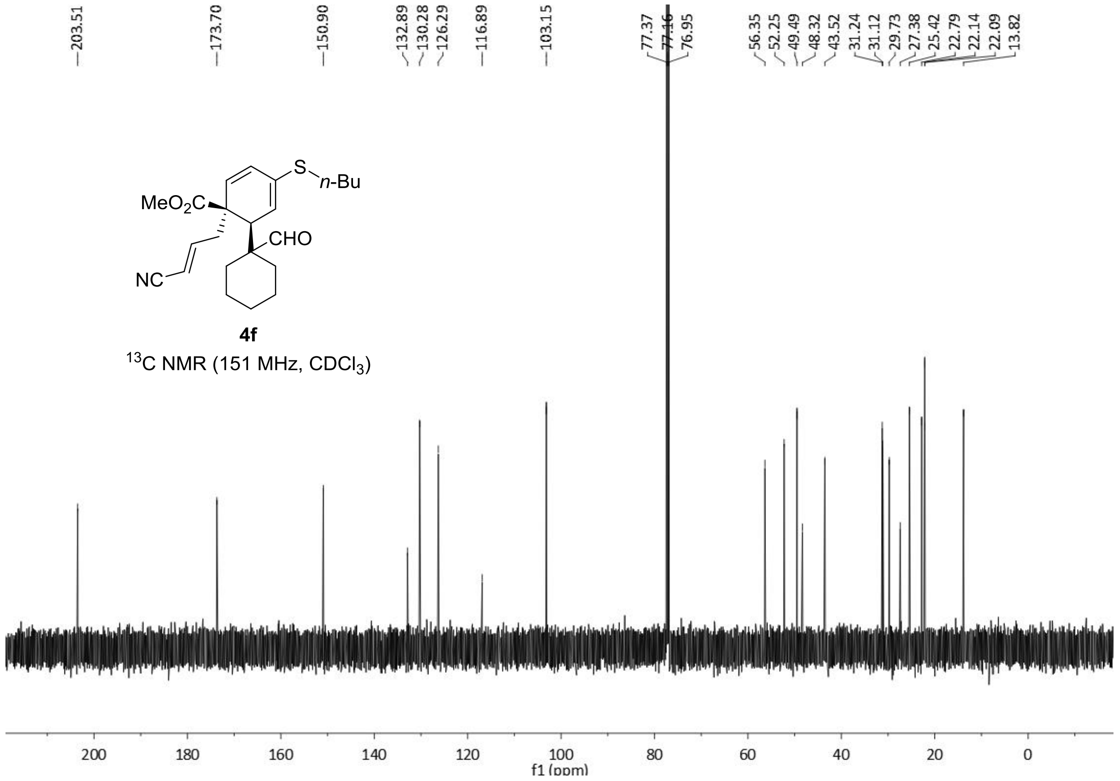

Supplementary Figure 76. ^{13}C NMR spectrum of **4f**

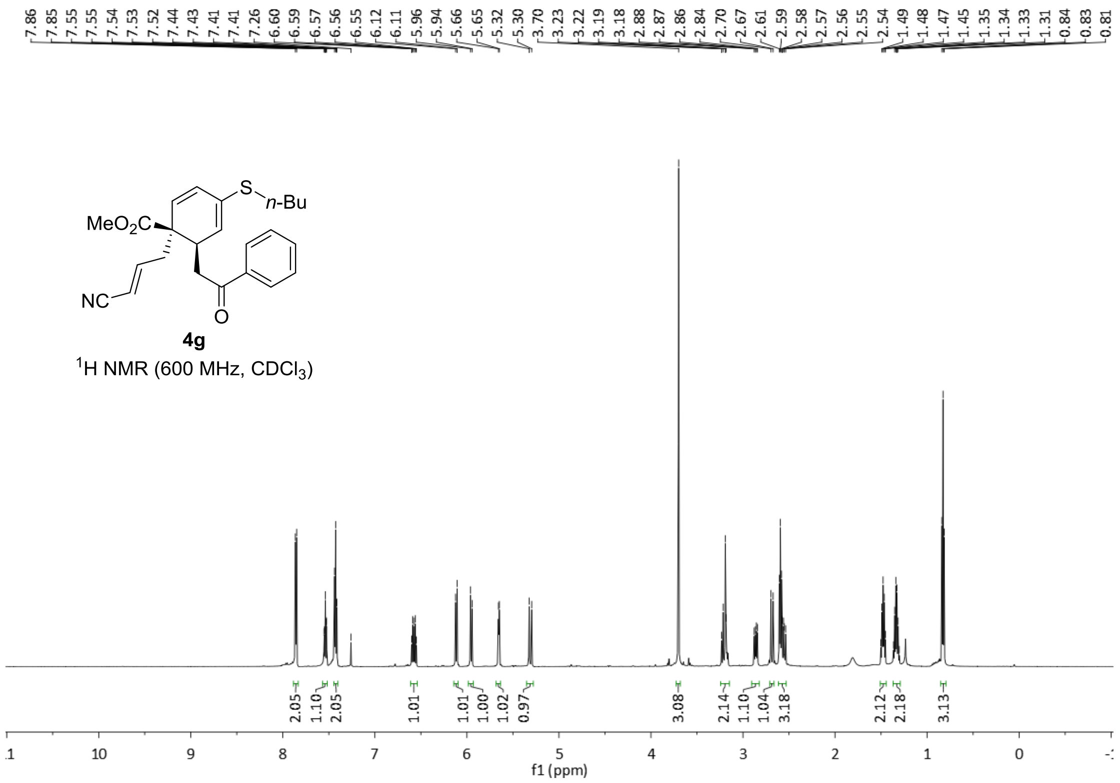

Supplementary Figure 77. ^{1}H NMR spectrum of **4g**

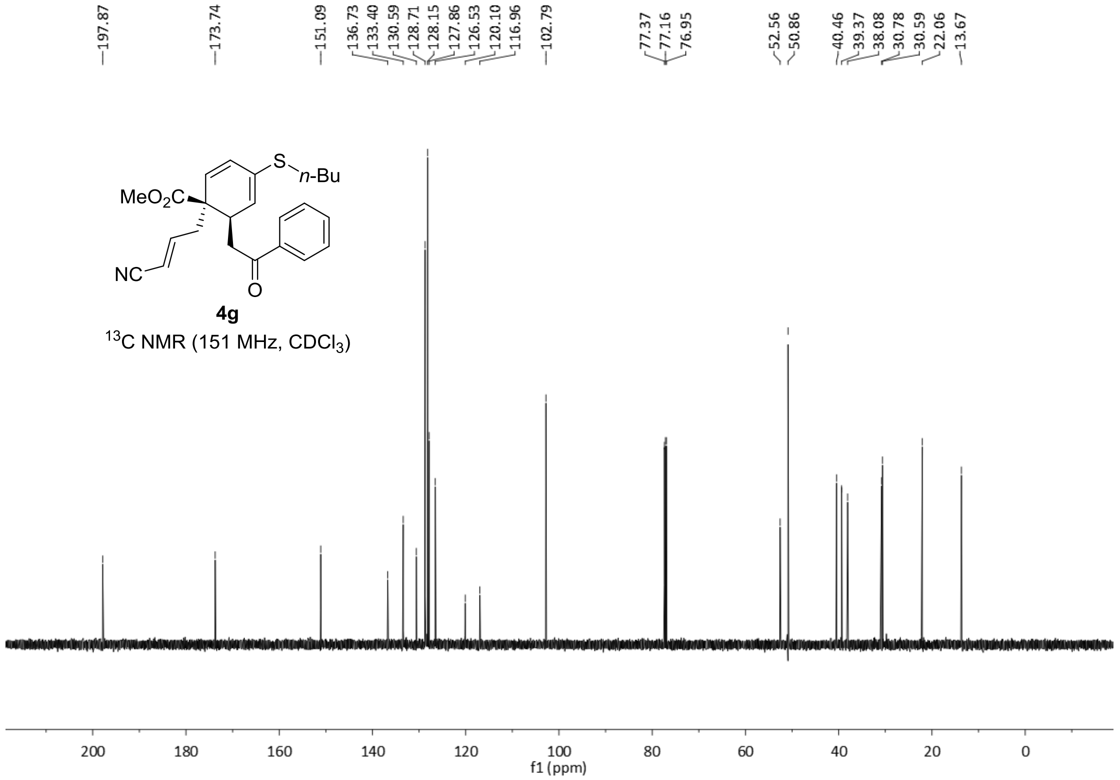

Supplementary Figure 78. ^{13}C NMR spectrum of **4g**

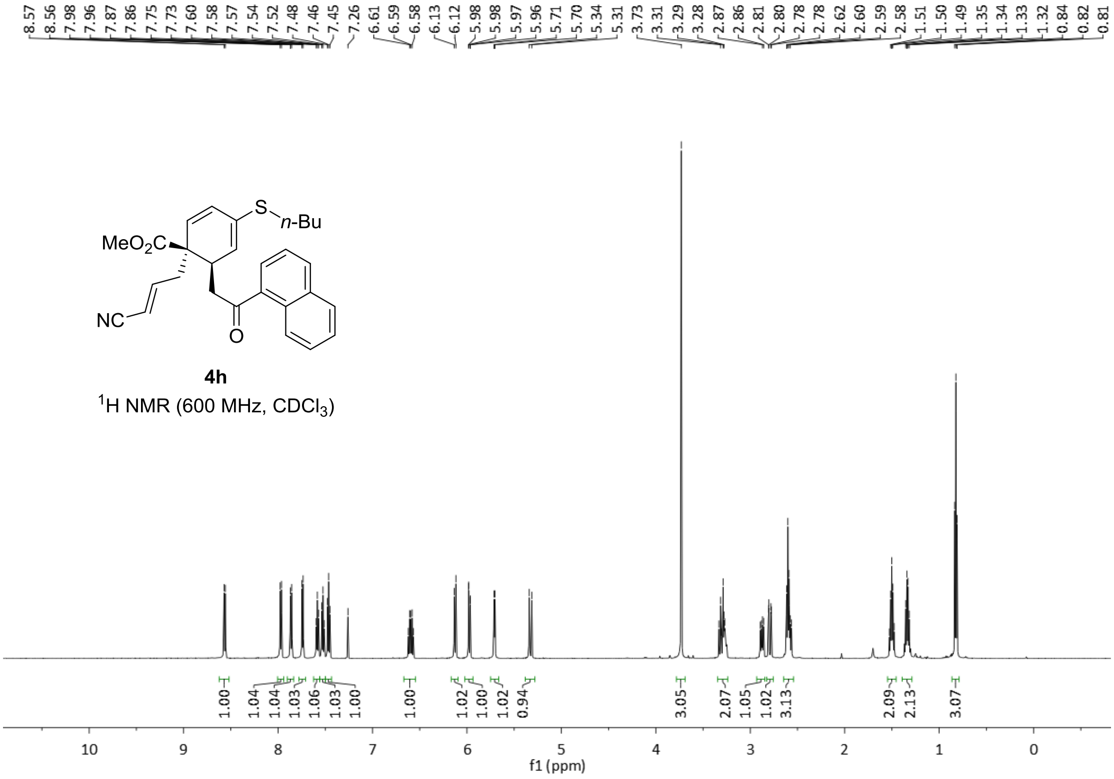

Supplementary Figure 79. ^{1}H NMR spectrum of **4h**

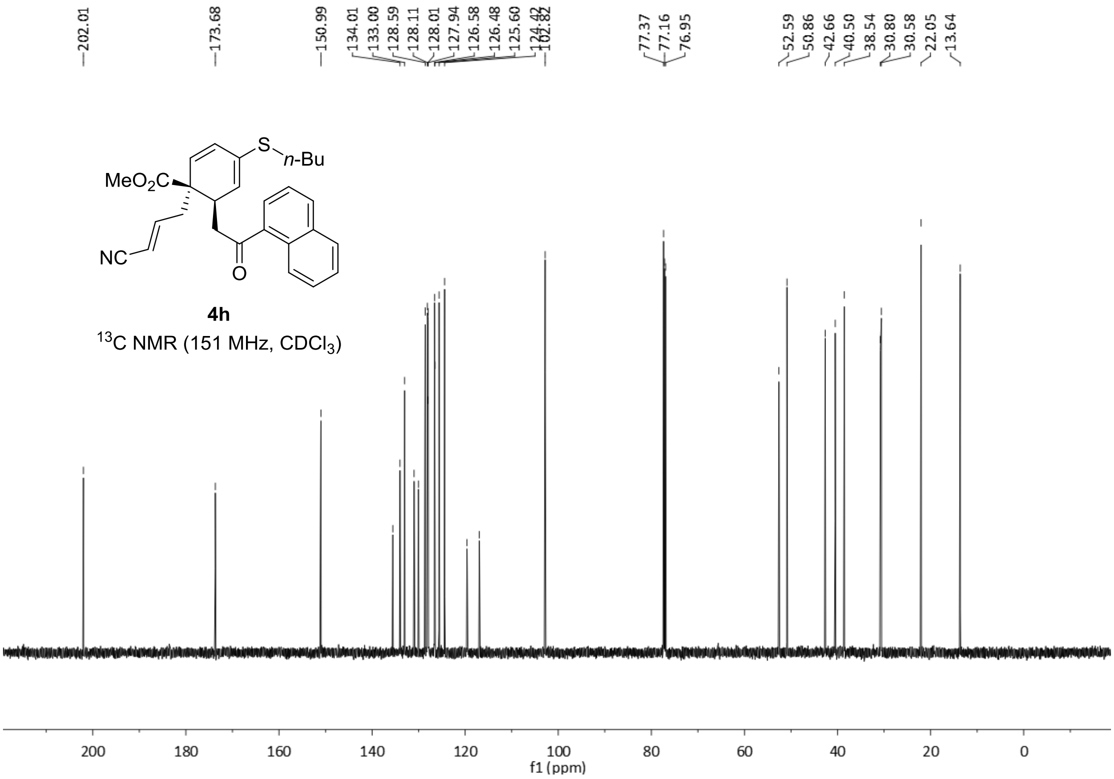

Supplementary Figure 80. ^{13}C NMR spectrum of **4h**

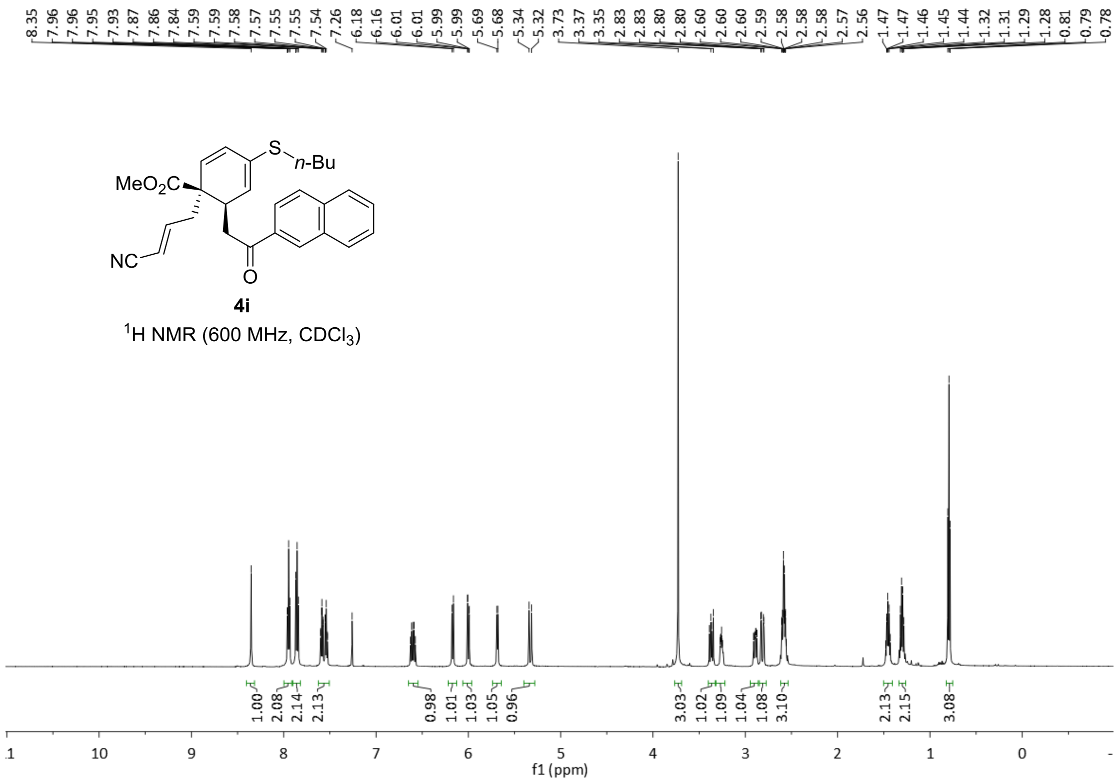

Supplementary Figure 81. ^{1}H NMR spectrum of **4i**

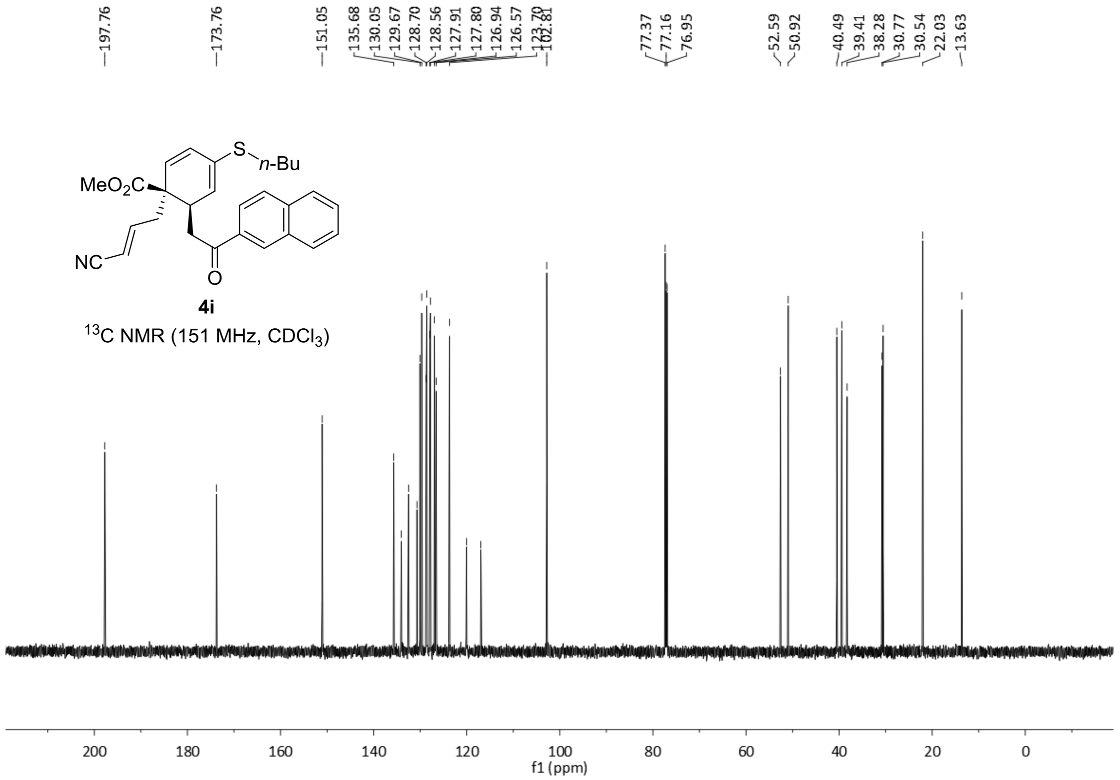

Supplementary Figure 82. ^{13}C NMR spectrum of **4i**

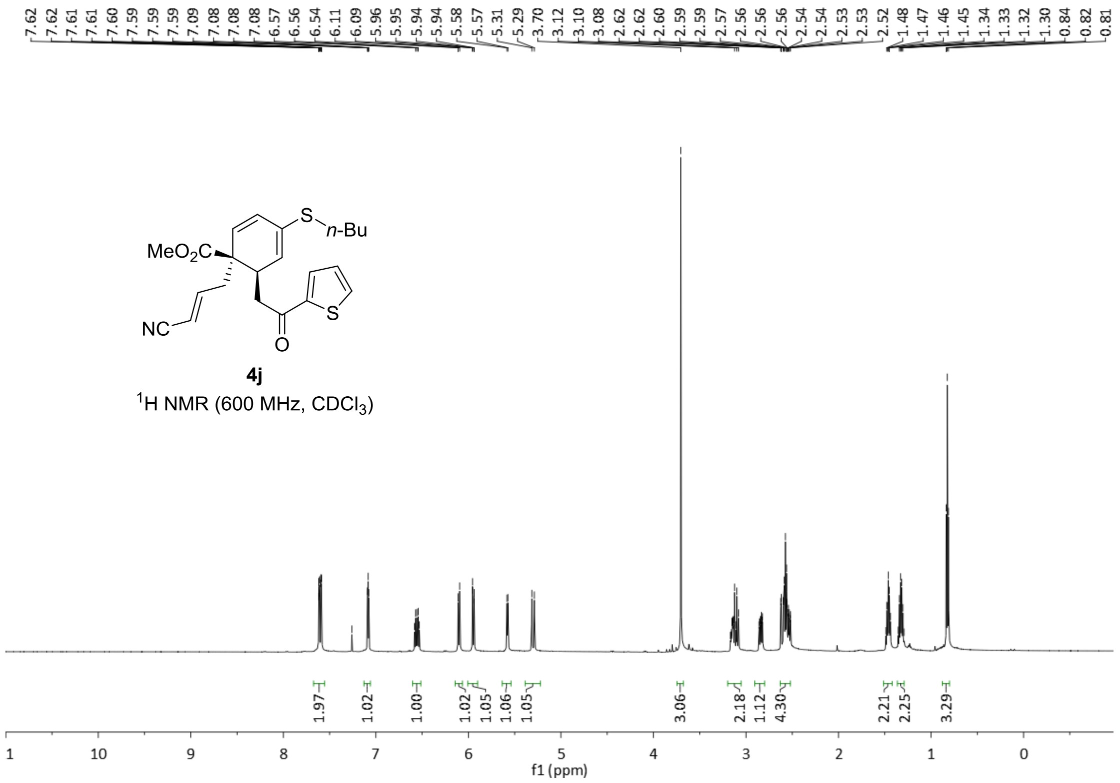

Supplementary Figure 83. ^{1}H NMR spectrum of **4j**

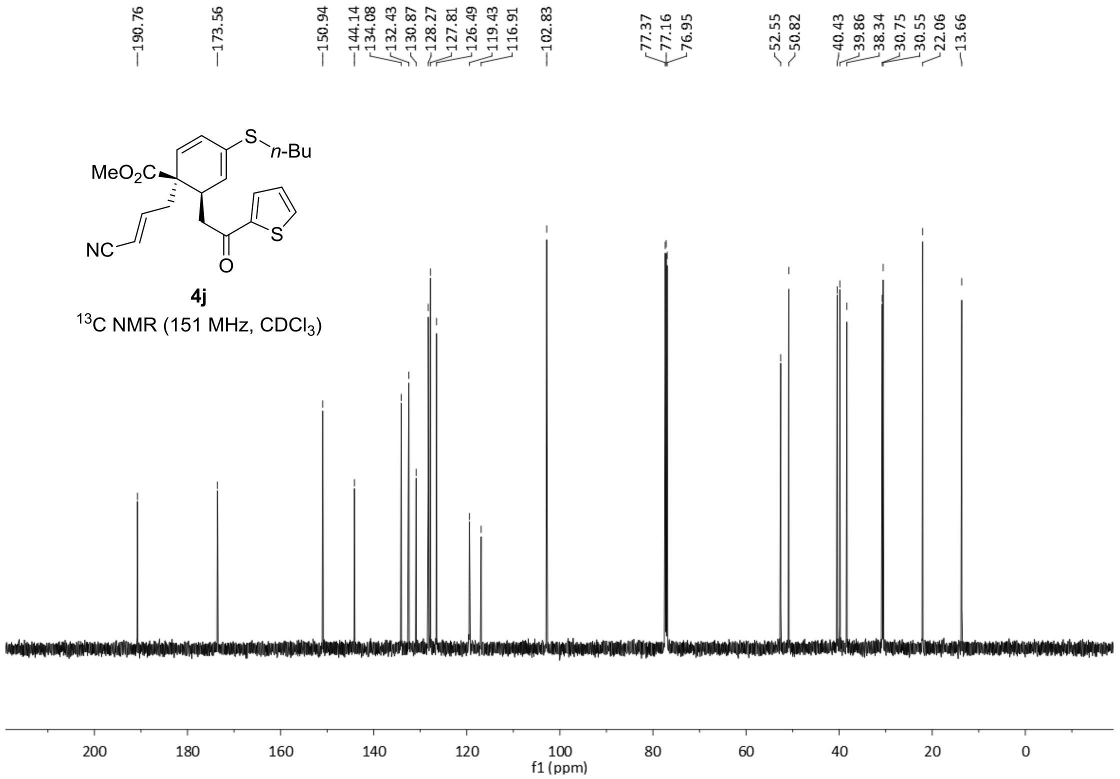

Supplementary Figure 84. ^{13}C NMR spectrum of **4j**

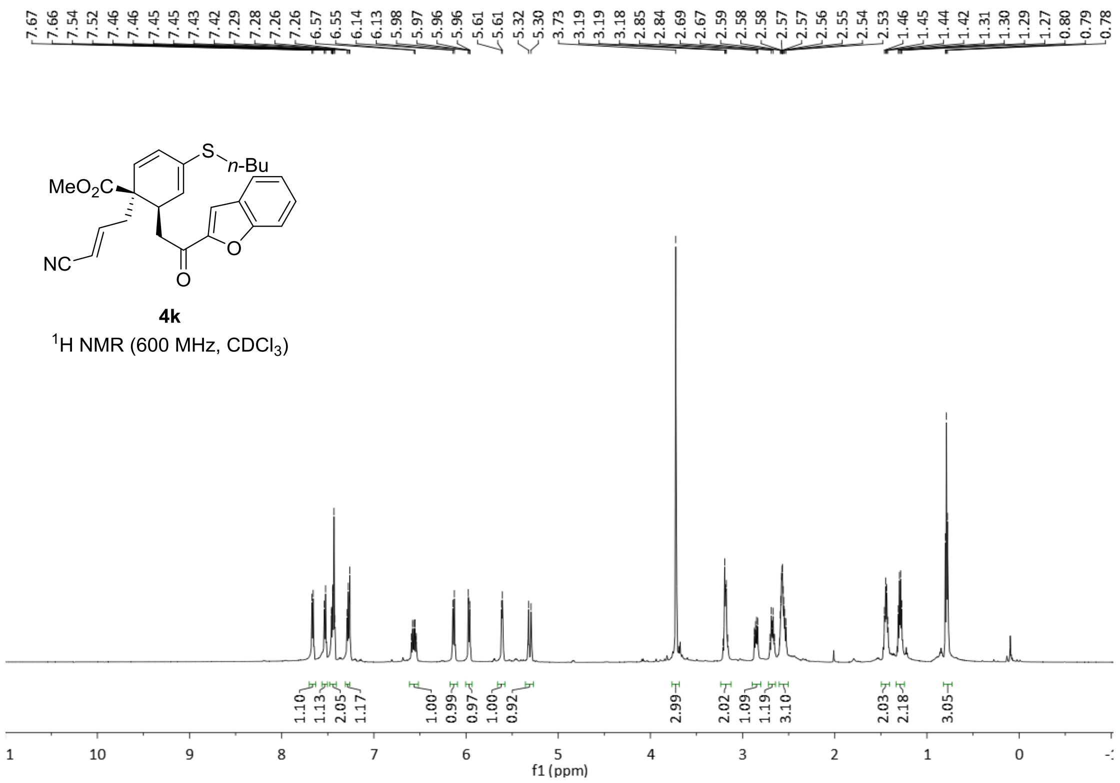

Supplementary Figure 85. ^{1}H NMR spectrum of **4k**

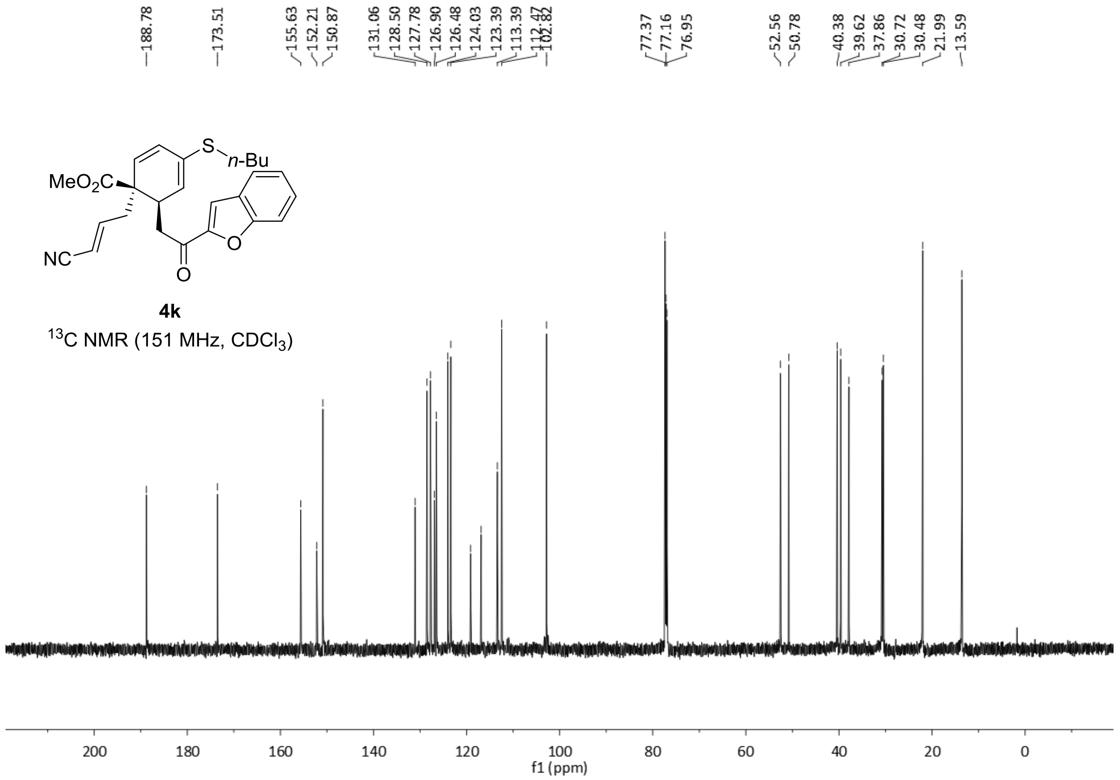

Supplementary Figure 86. ^{13}C NMR spectrum of **4k**

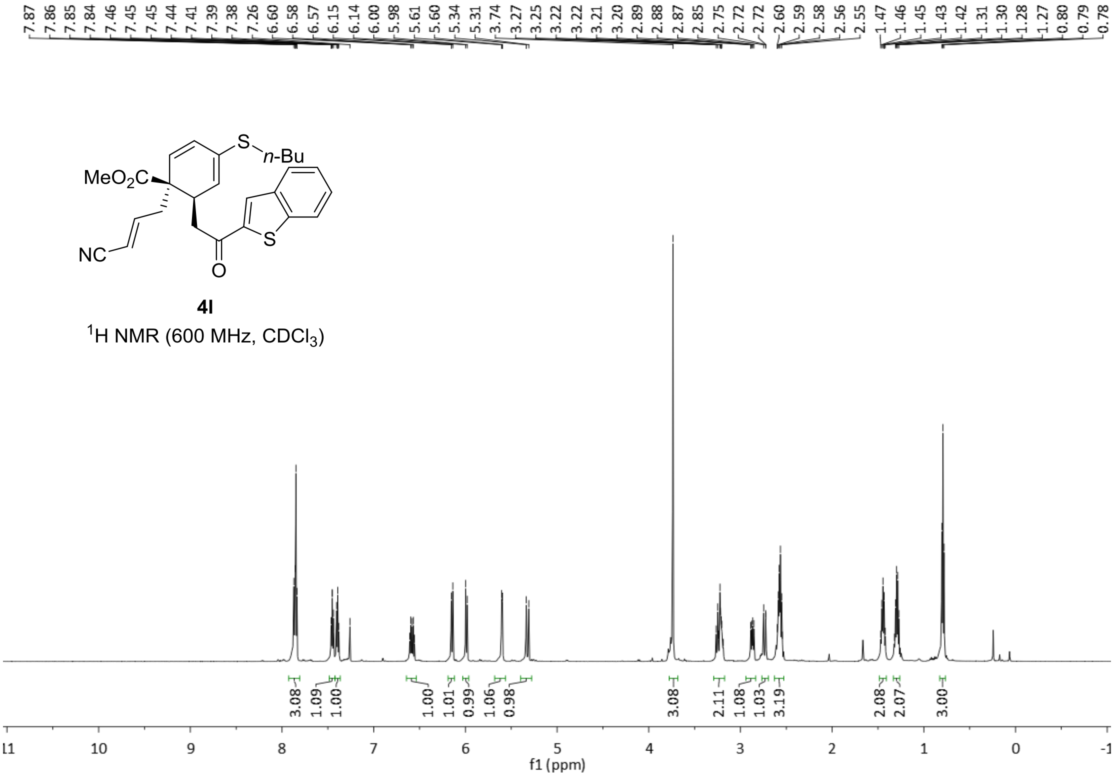

Supplementary Figure 87. ^{1}H NMR spectrum of **4l**

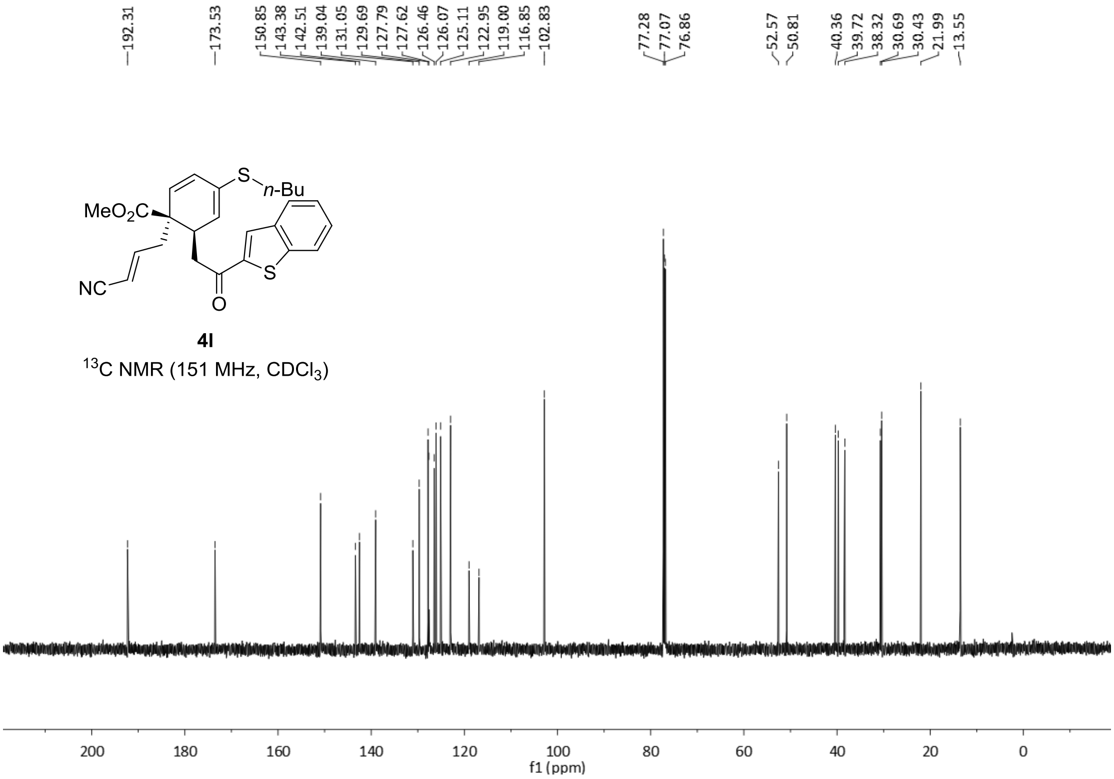

Supplementary Figure 88. ^{13}C NMR spectrum of **4l**

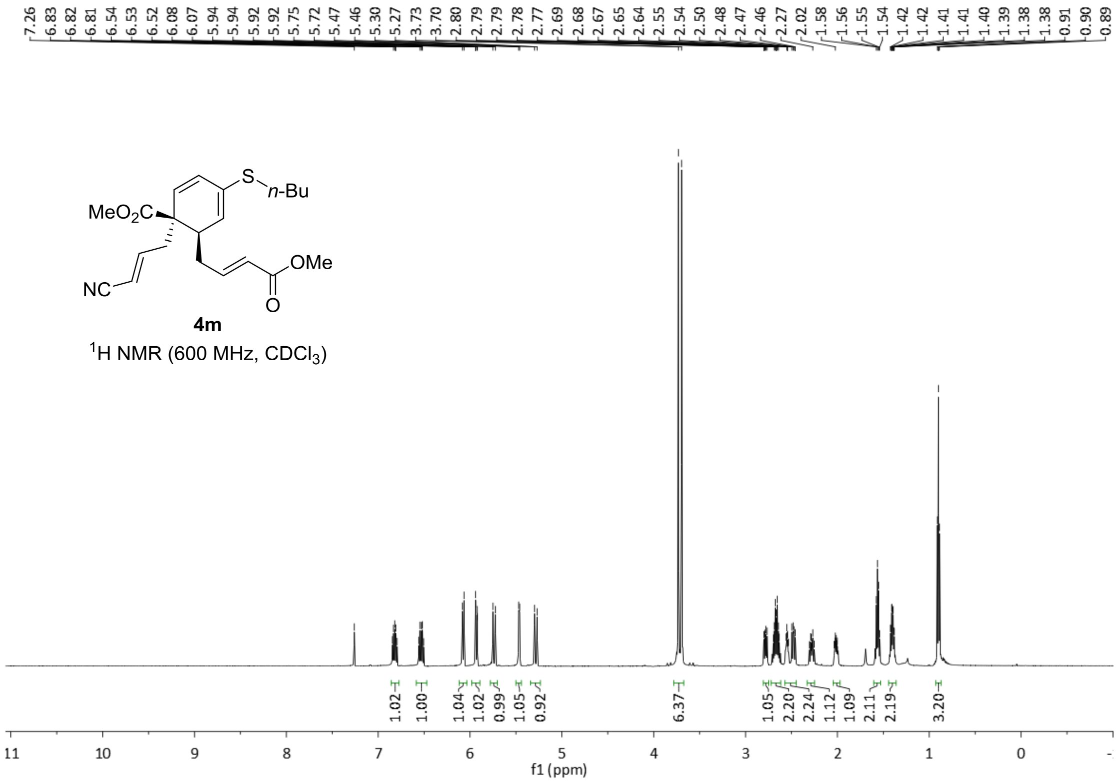

Supplementary Figure 89. ^{1}H NMR spectrum of **4m**

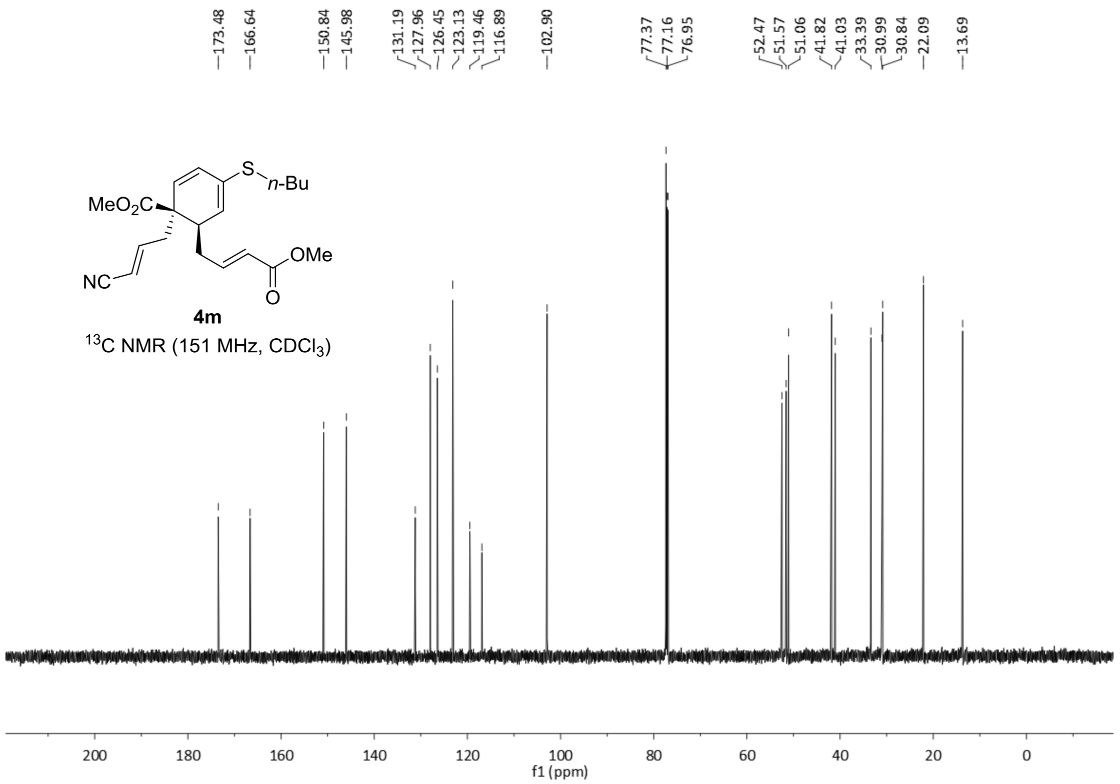

Supplementary Figure 90. ^{13}C NMR spectrum of **4m**

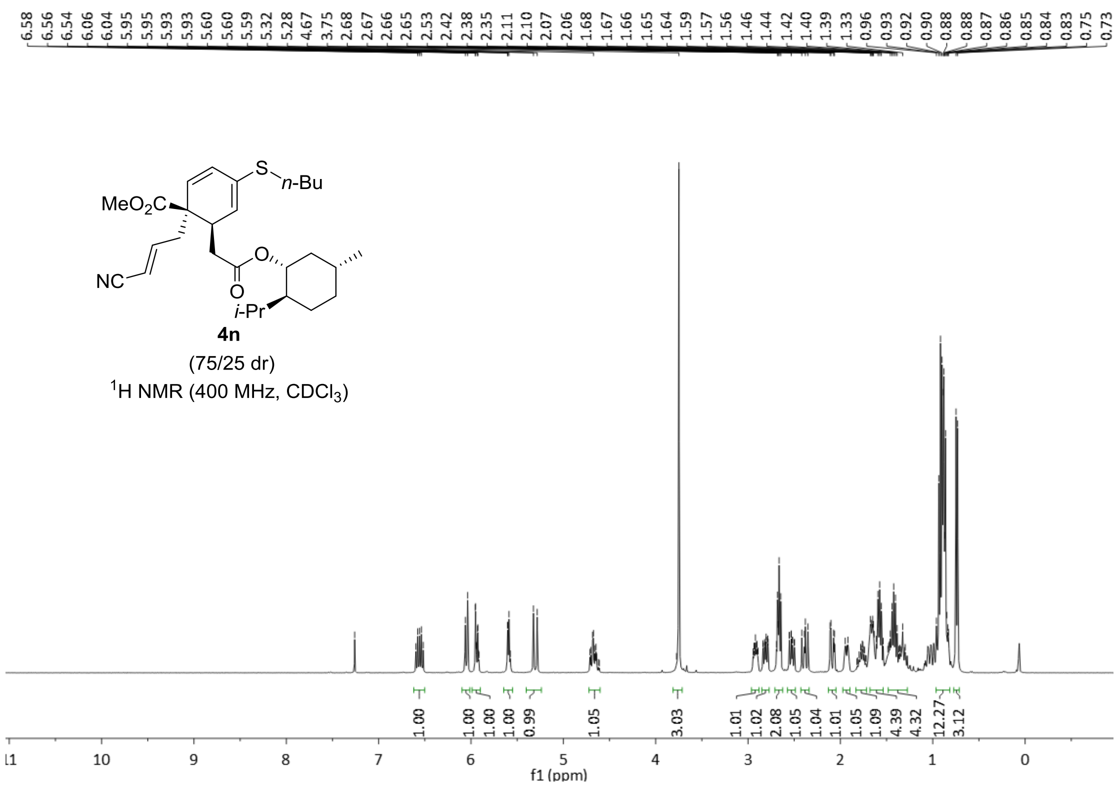

Supplementary Figure 91. ^{1}H NMR spectrum of **4n**

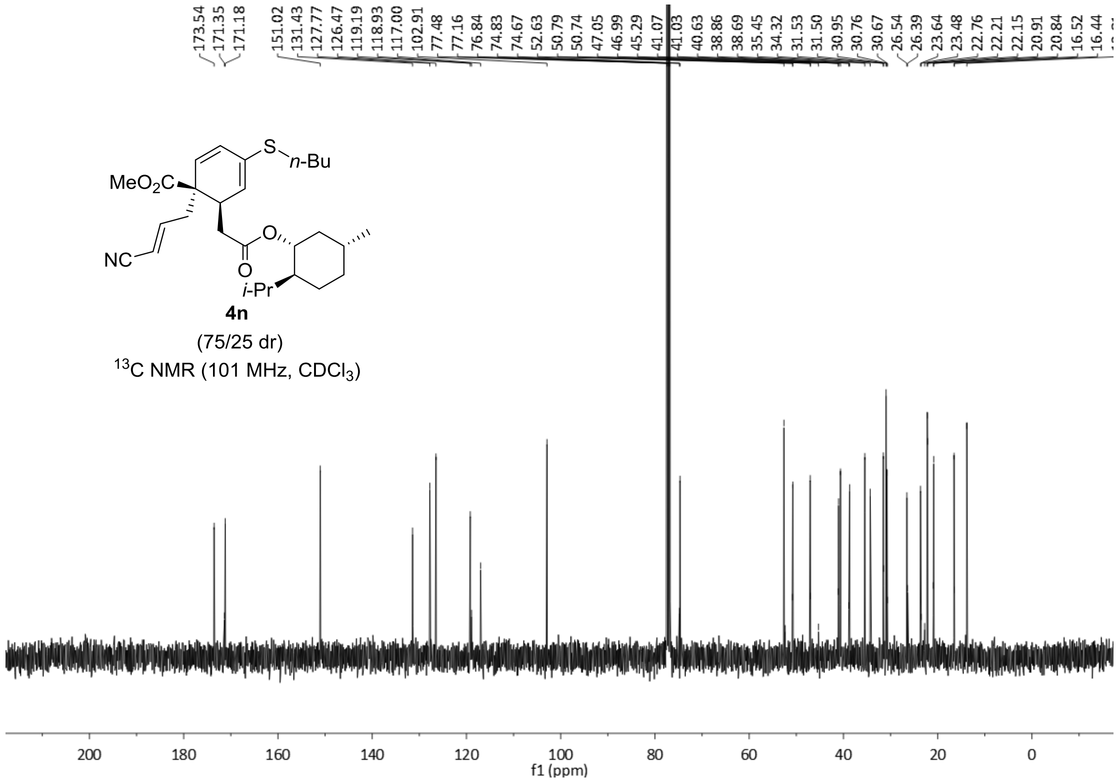

Supplementary Figure 92. ^{13}C NMR spectrum of **4n**

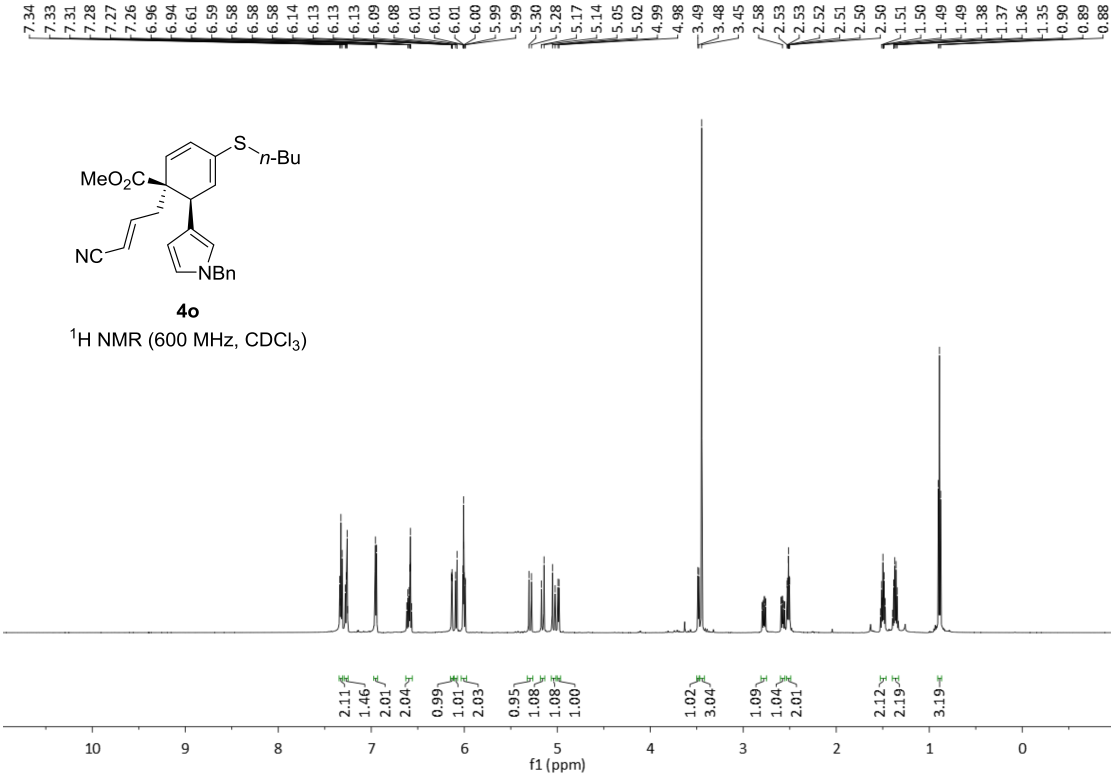

Supplementary Figure 93. ^{1}H NMR spectrum of **4o**

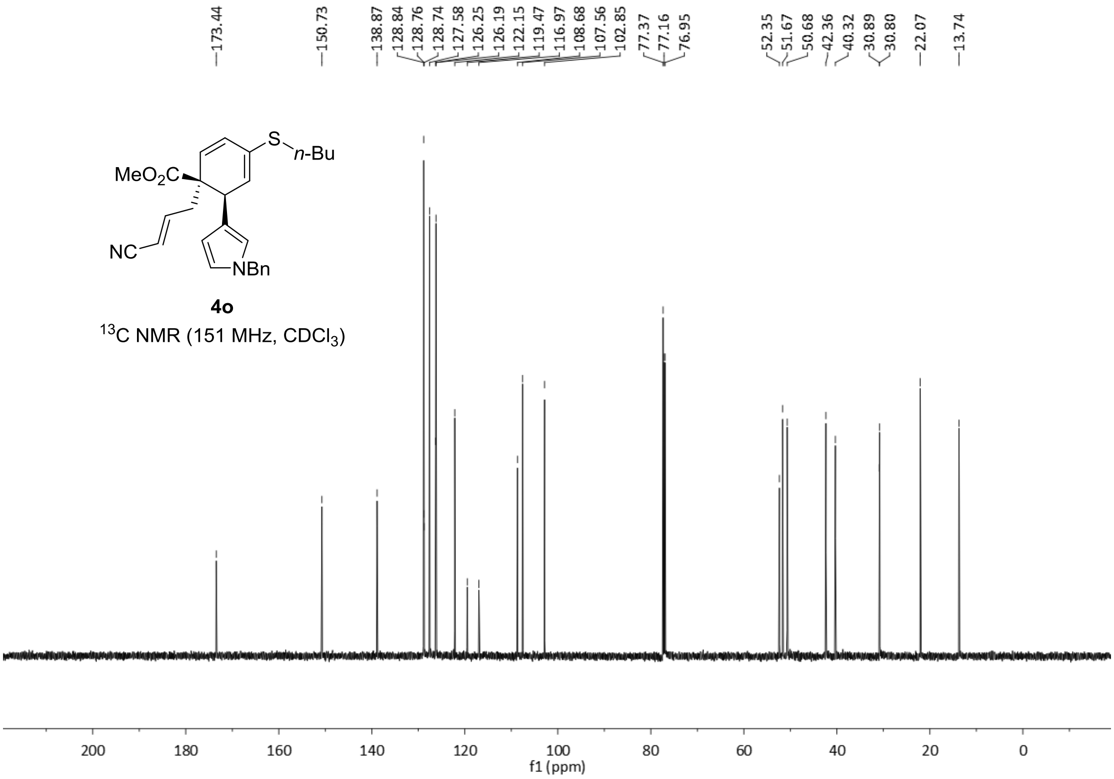

Supplementary Figure 94. ^{13}C NMR spectrum of **4o**

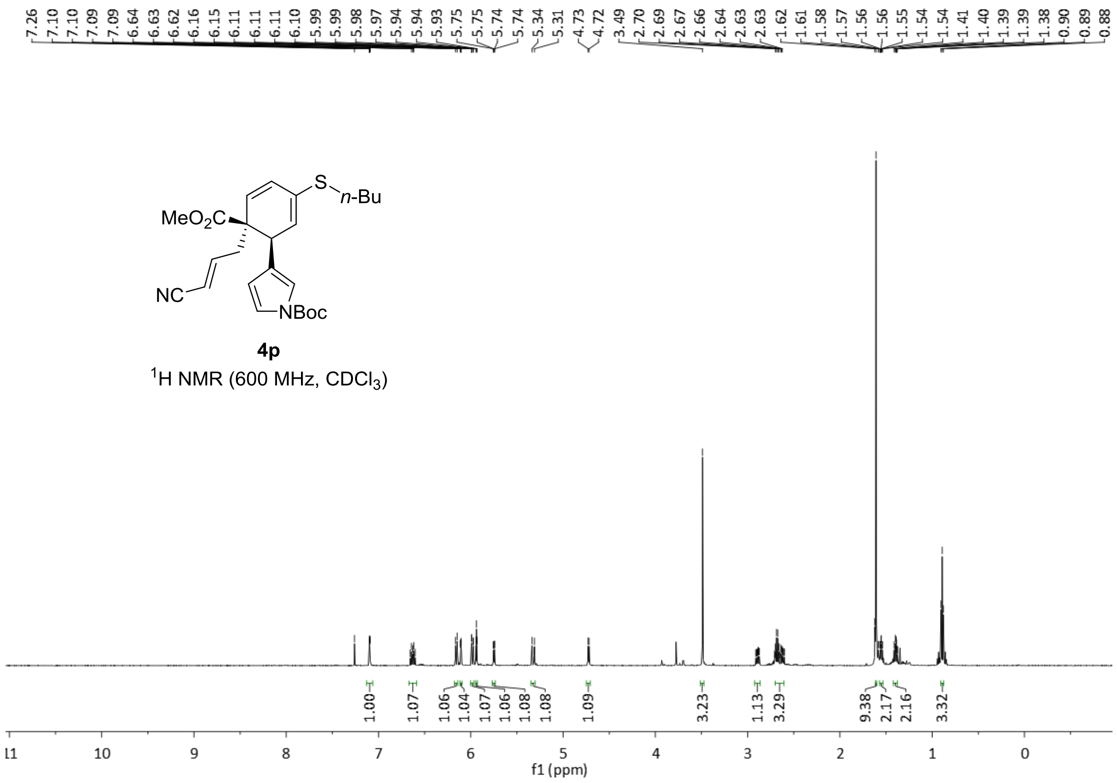

Supplementary Figure 95. ^{1}H NMR spectrum of **4p**

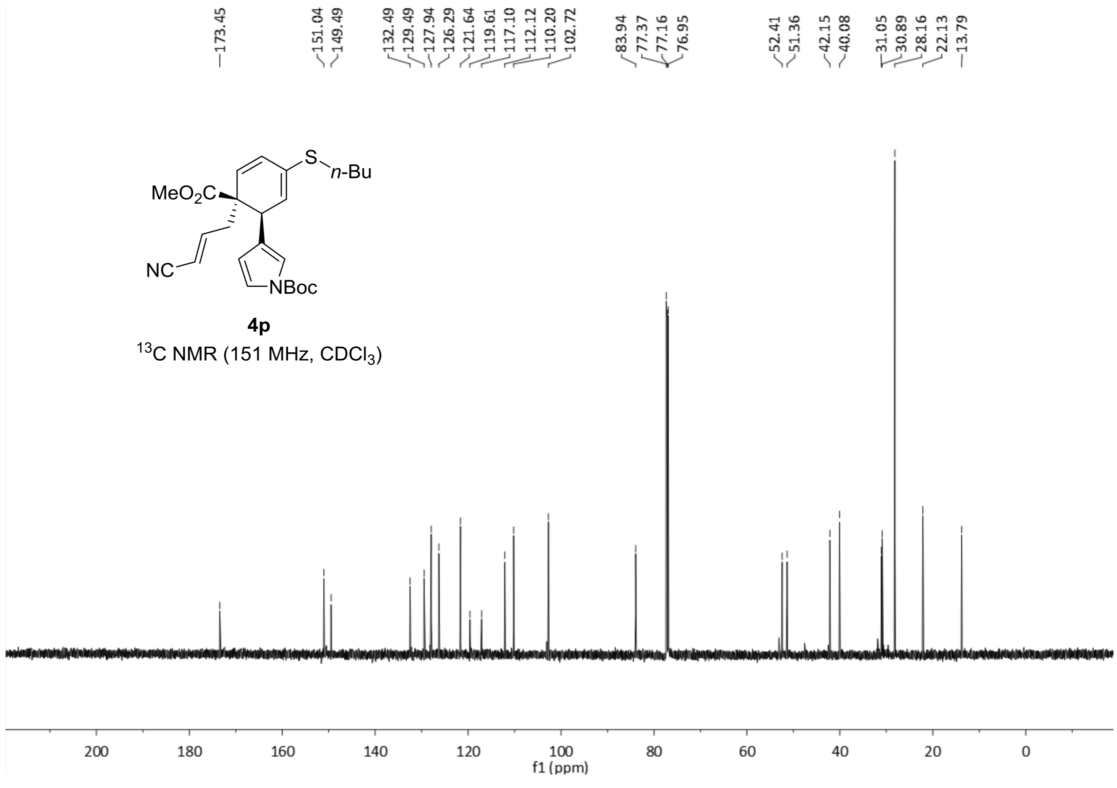

Supplementary Figure 96. ^{13}C NMR spectrum of **4p**

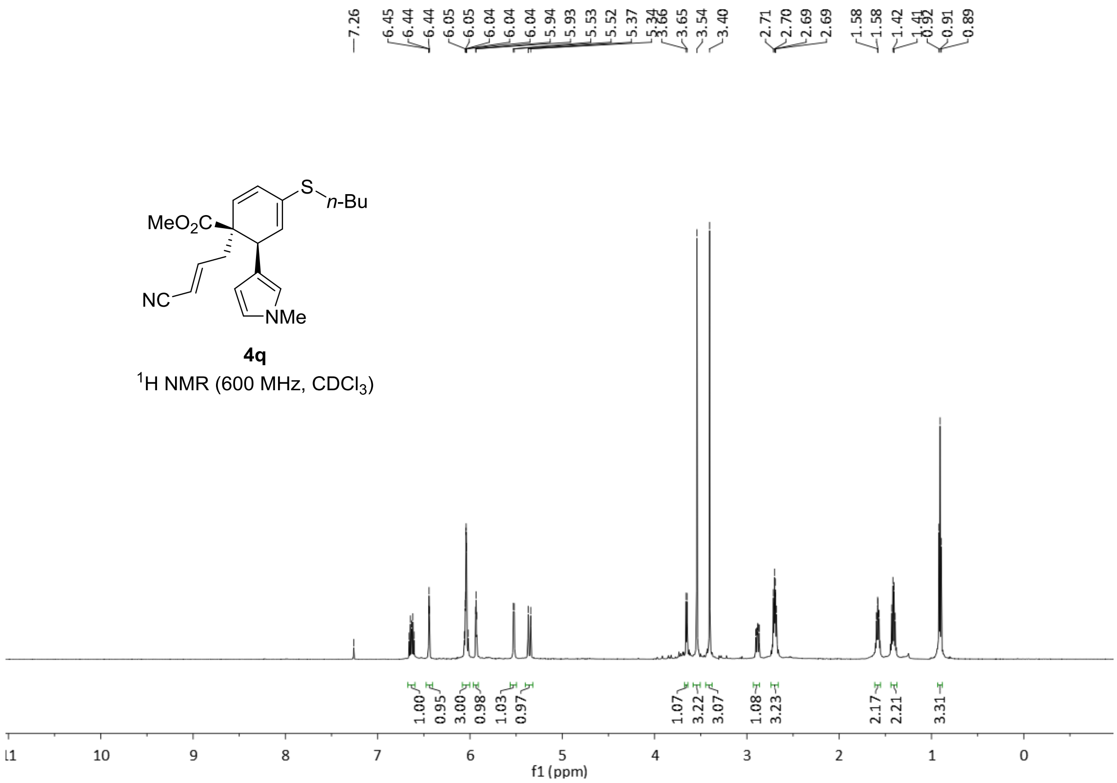

Supplementary Figure 97. ^{1}H NMR spectrum of **4q**

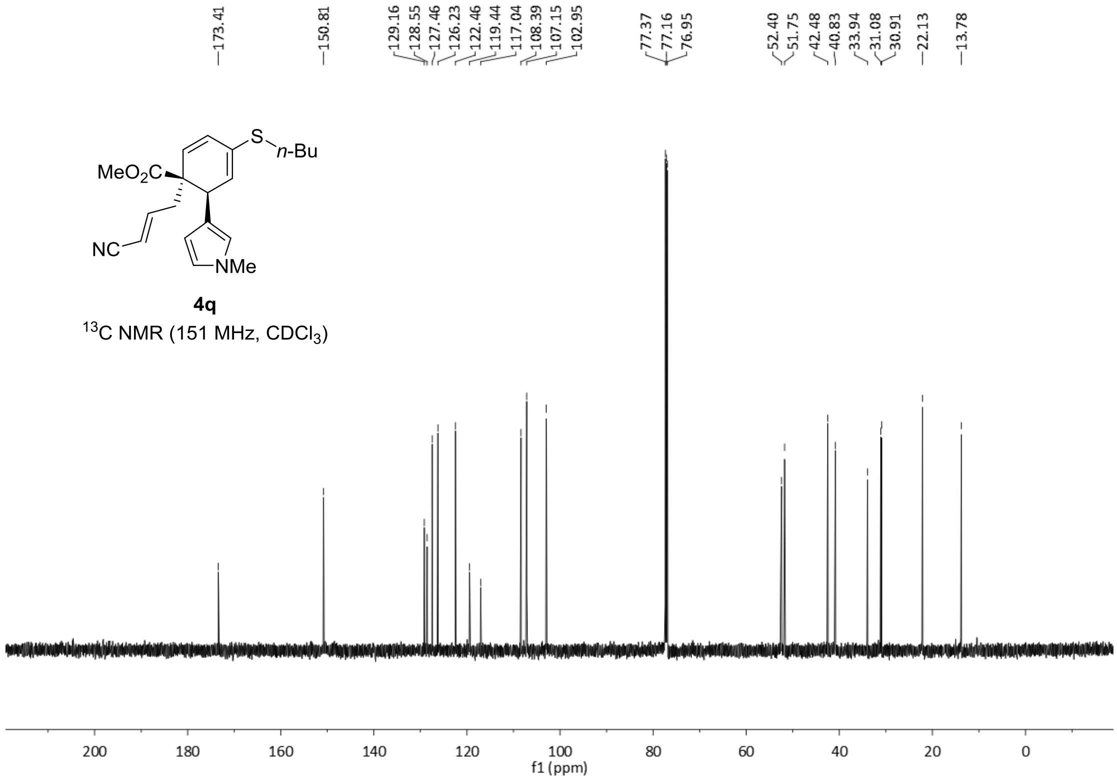

Supplementary Figure 98. ^{13}C NMR spectrum of **4q**

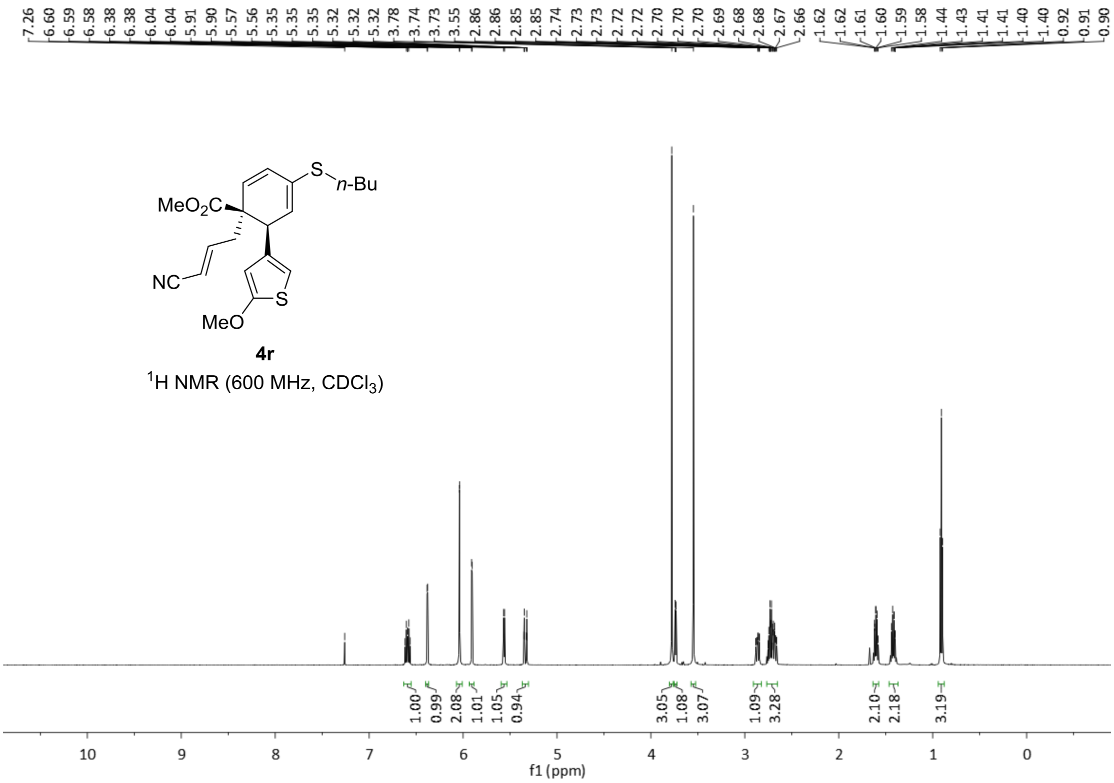

Supplementary Figure 99. ^{1}H NMR spectrum of **4r**

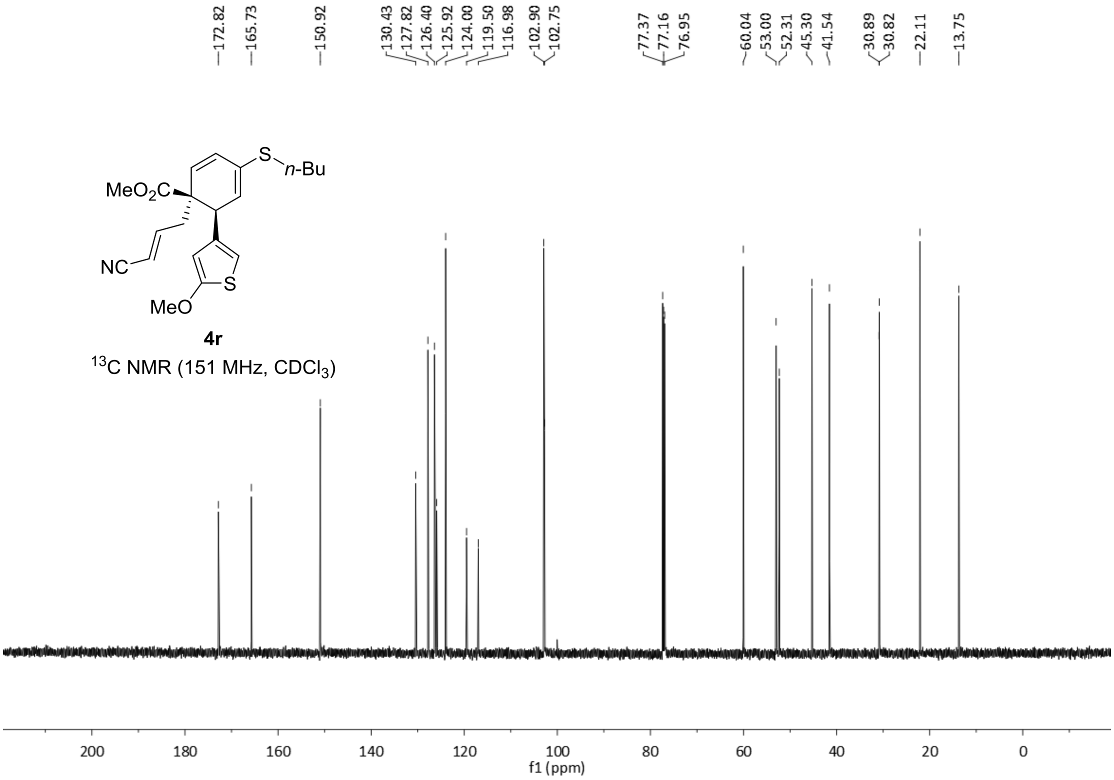

Supplementary Figure 100. ^{13}C NMR spectrum of **4r**

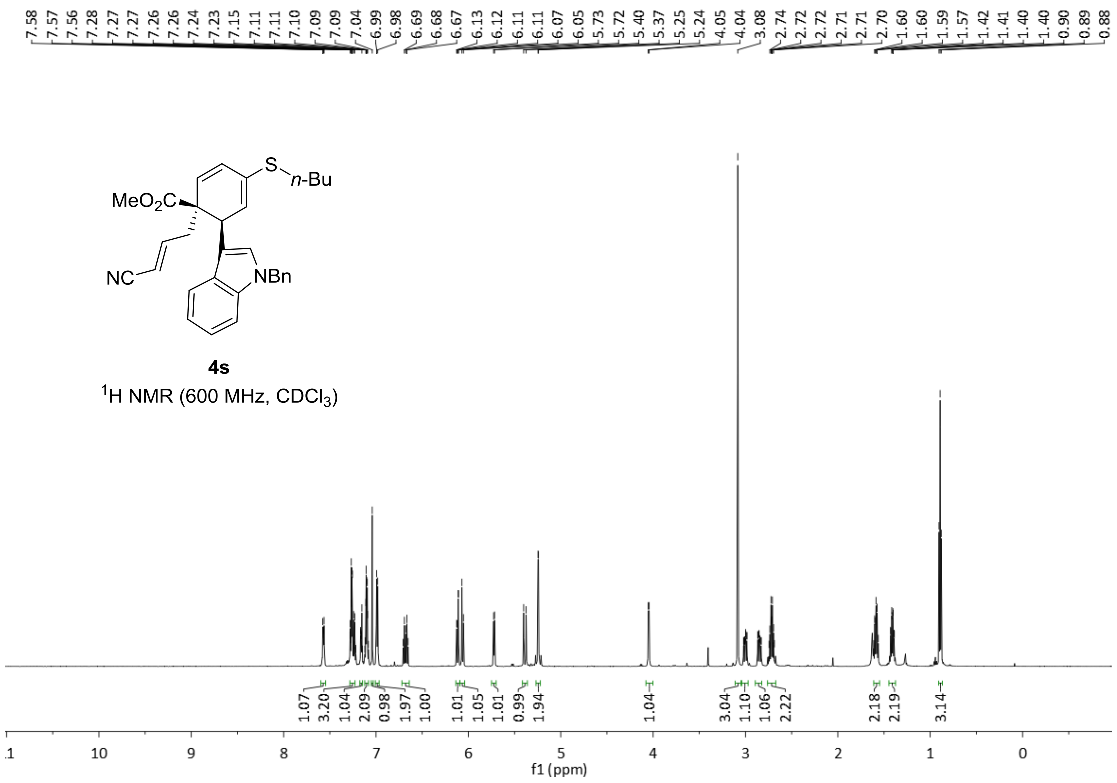

Supplementary Figure 101. ^{1}H NMR spectrum of **4s**

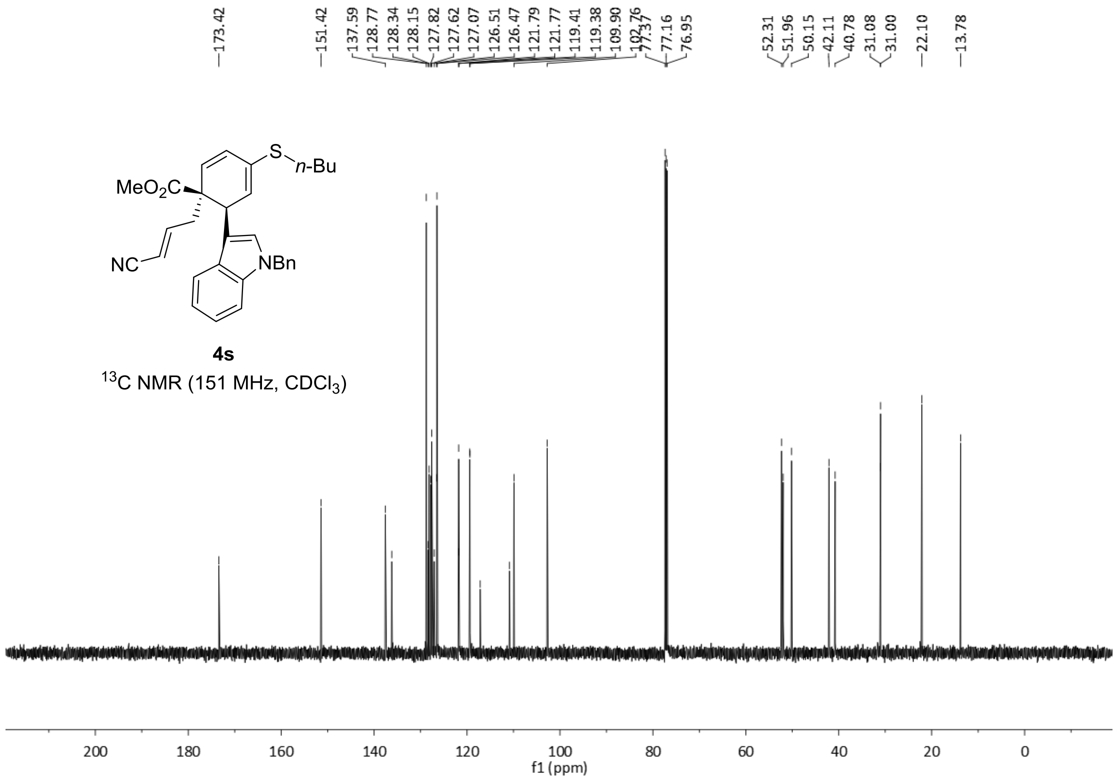

Supplementary Figure 102. ^{13}C NMR spectrum of **4s**

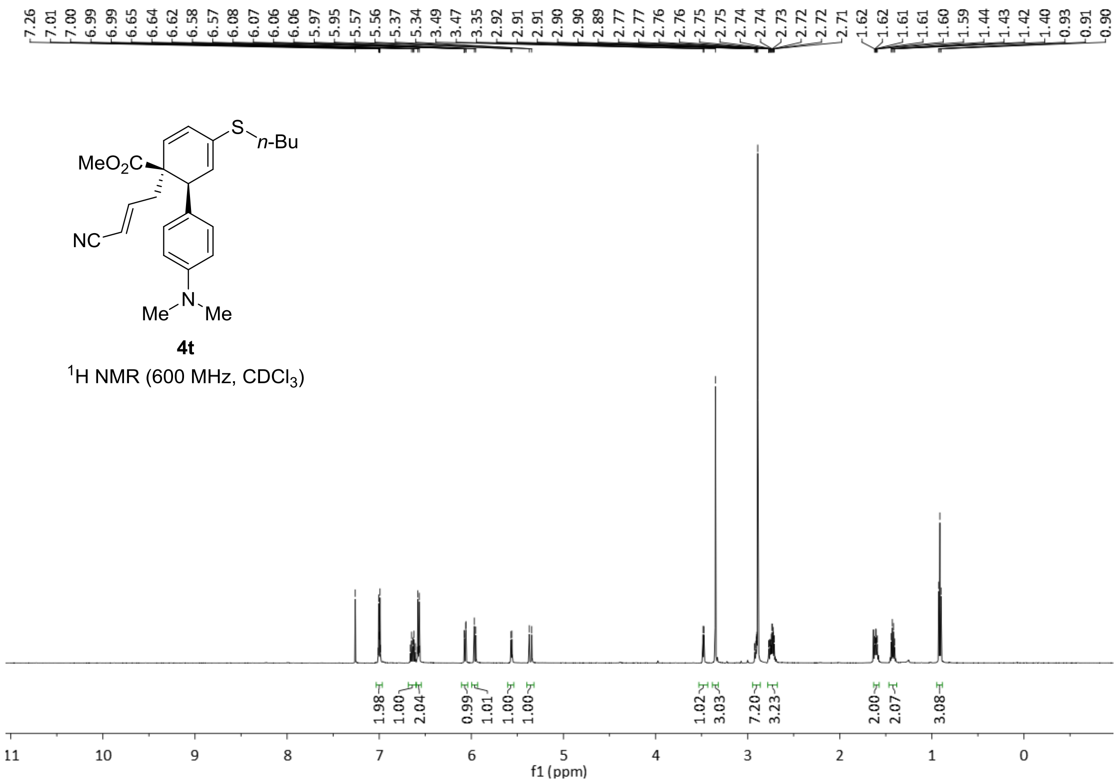

Supplementary Figure 103. ^{1}H NMR spectrum of **4t**

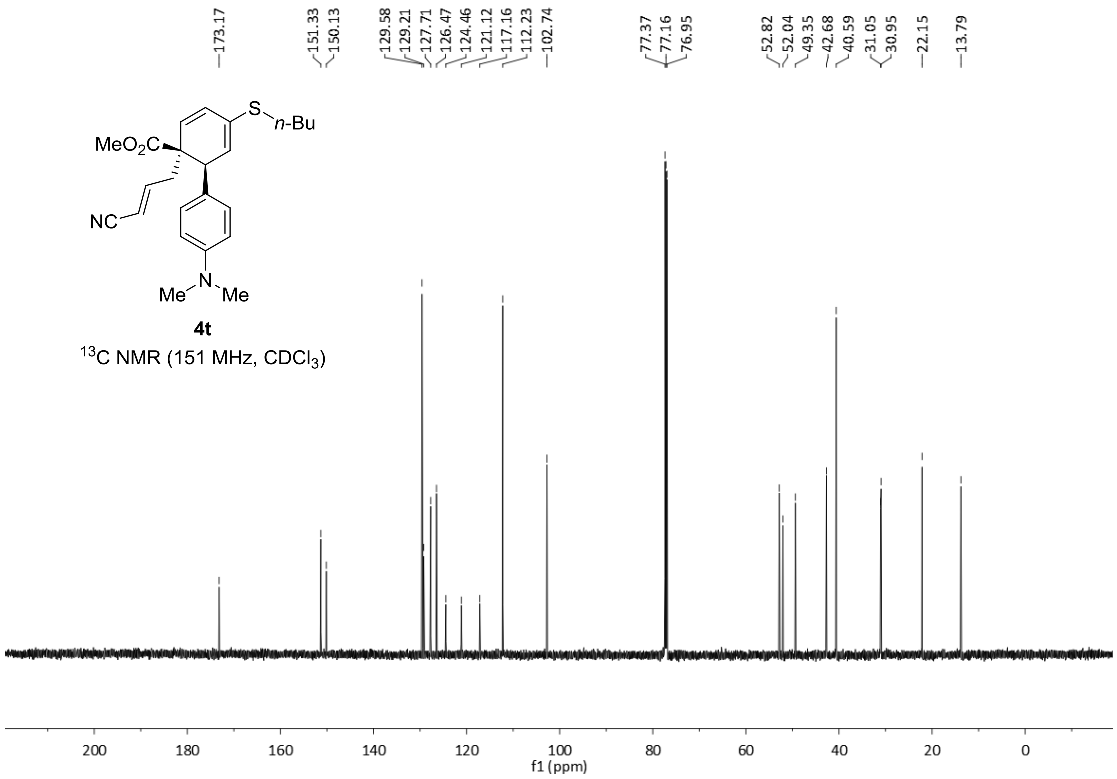

Supplementary Figure 104. ^{13}C NMR spectrum of **4t**

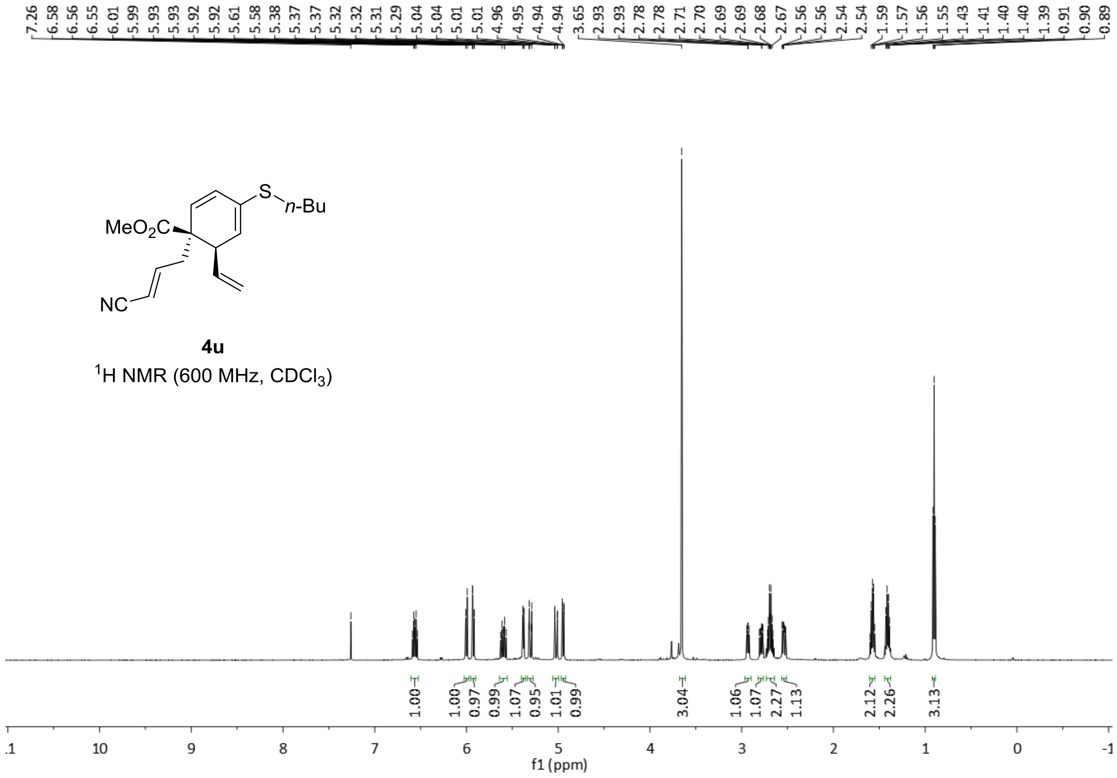

Supplementary Figure 105. ^{1}H NMR spectrum of **4u**

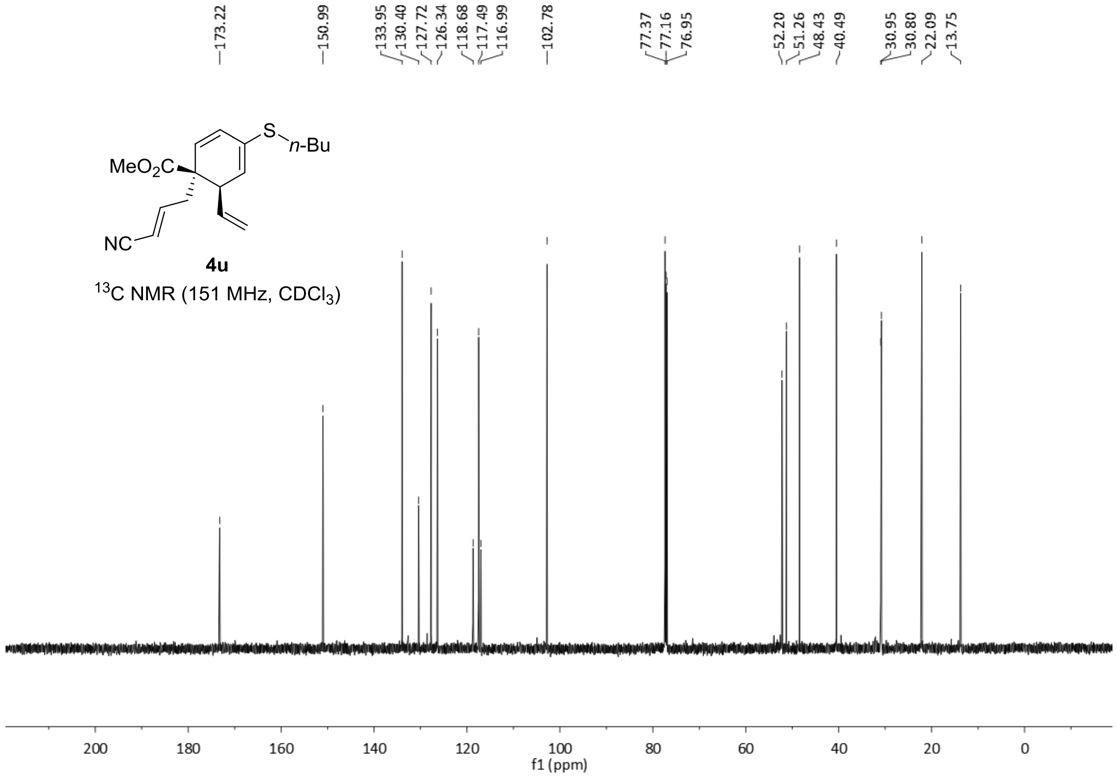

Supplementary Figure 106. ^{13}C NMR spectrum of **4u**

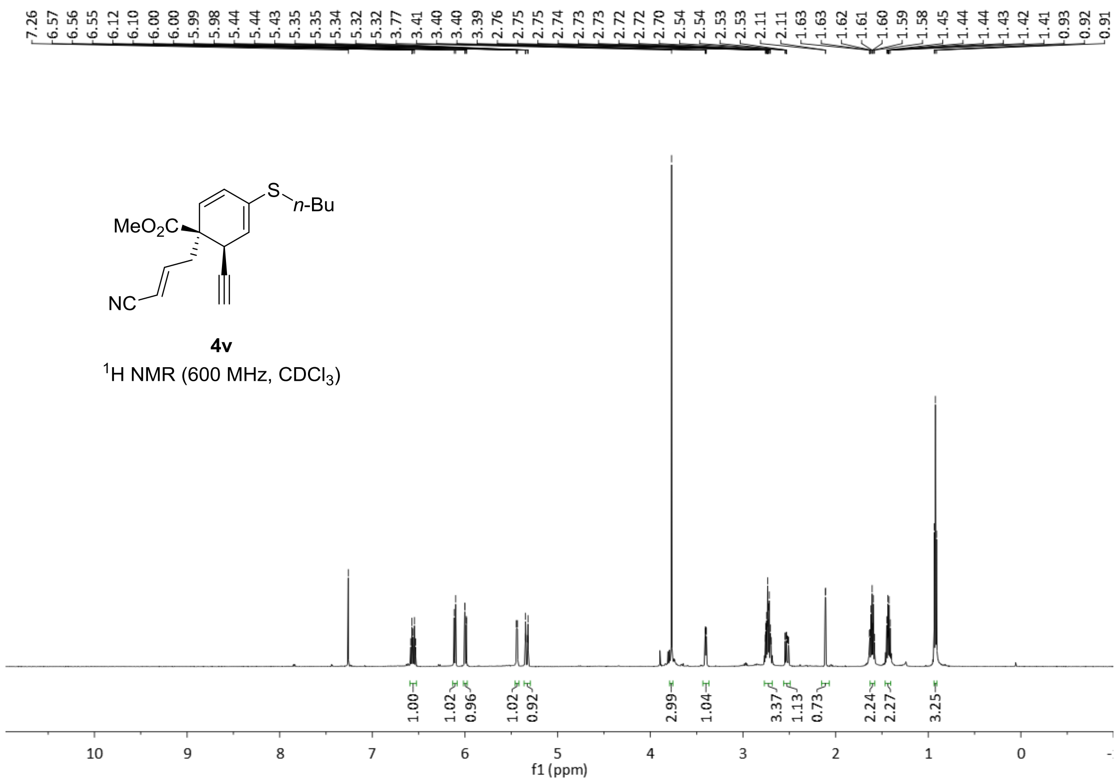

Supplementary Figure 107. ^{1}H NMR spectrum of **4v**

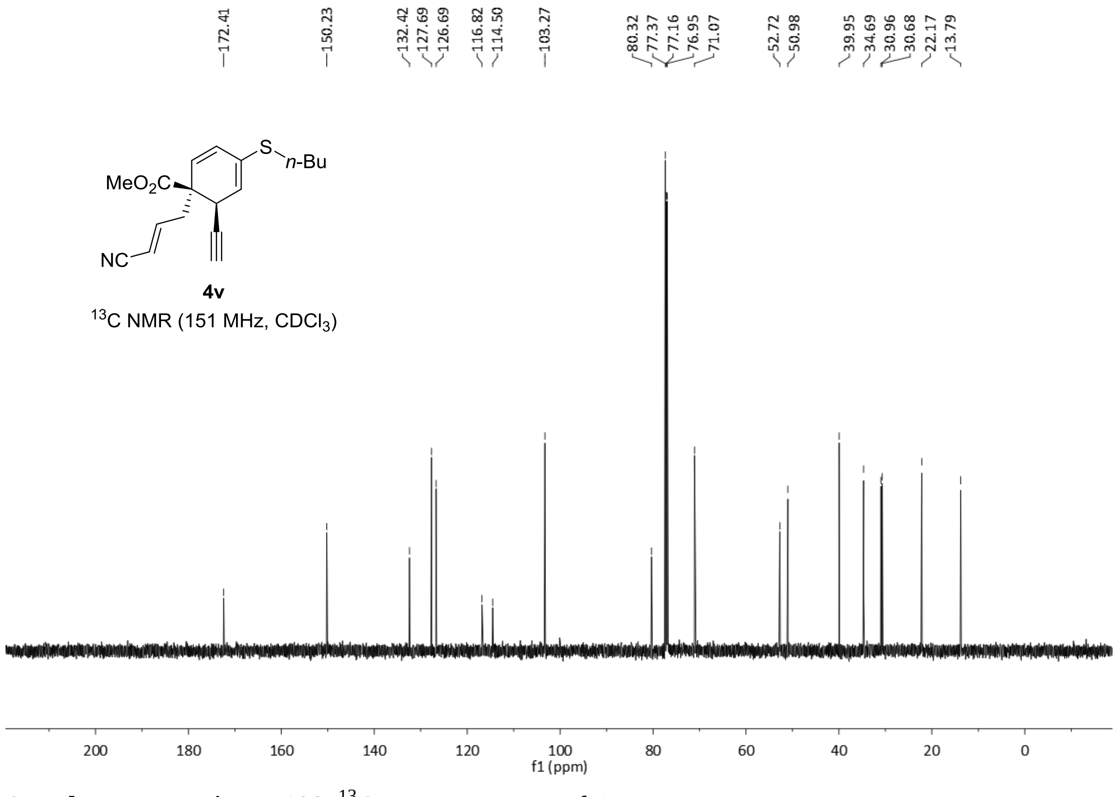

Supplementary Figure 108. ^{13}C NMR spectrum of **4v**

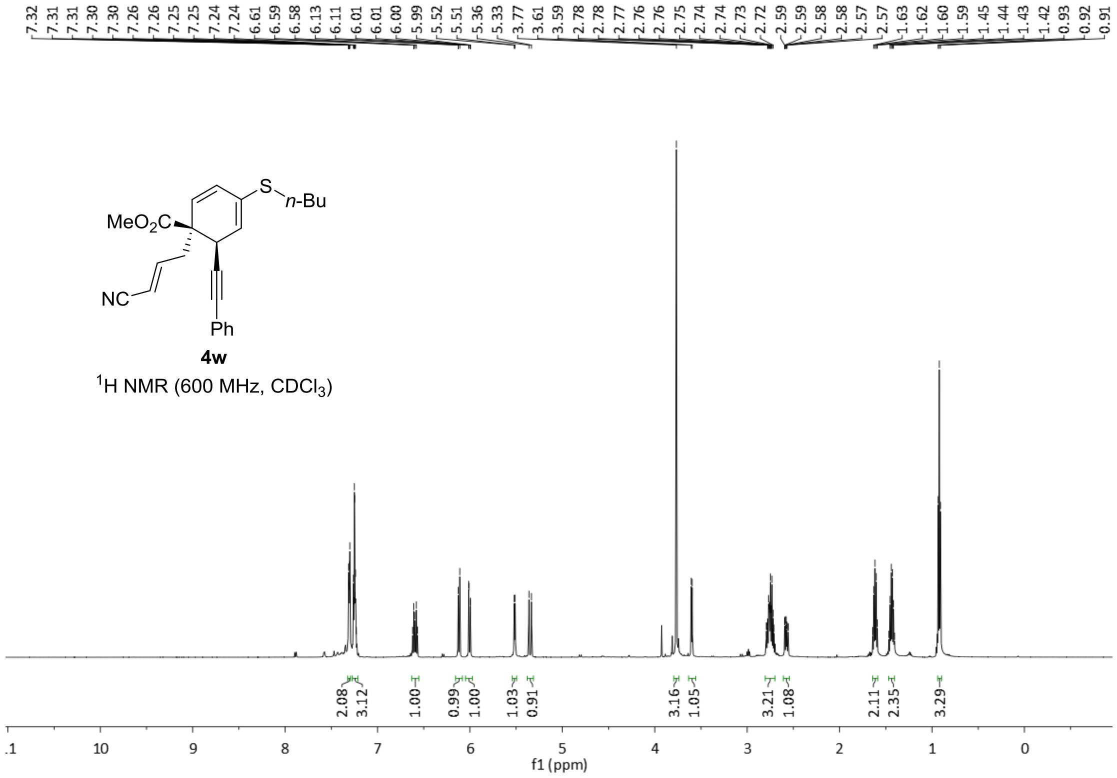

Supplementary Figure 109. ^{1}H NMR spectrum of **4w**

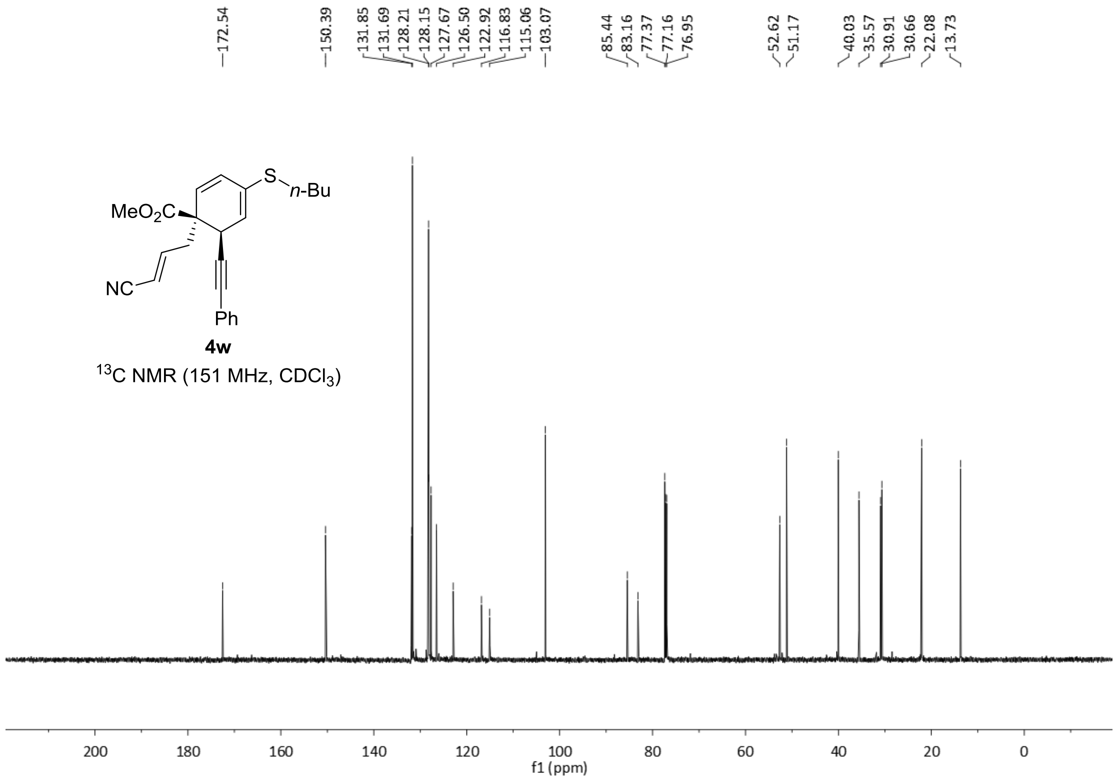

Supplementary Figure 110. ^{13}C NMR spectrum of **4w**

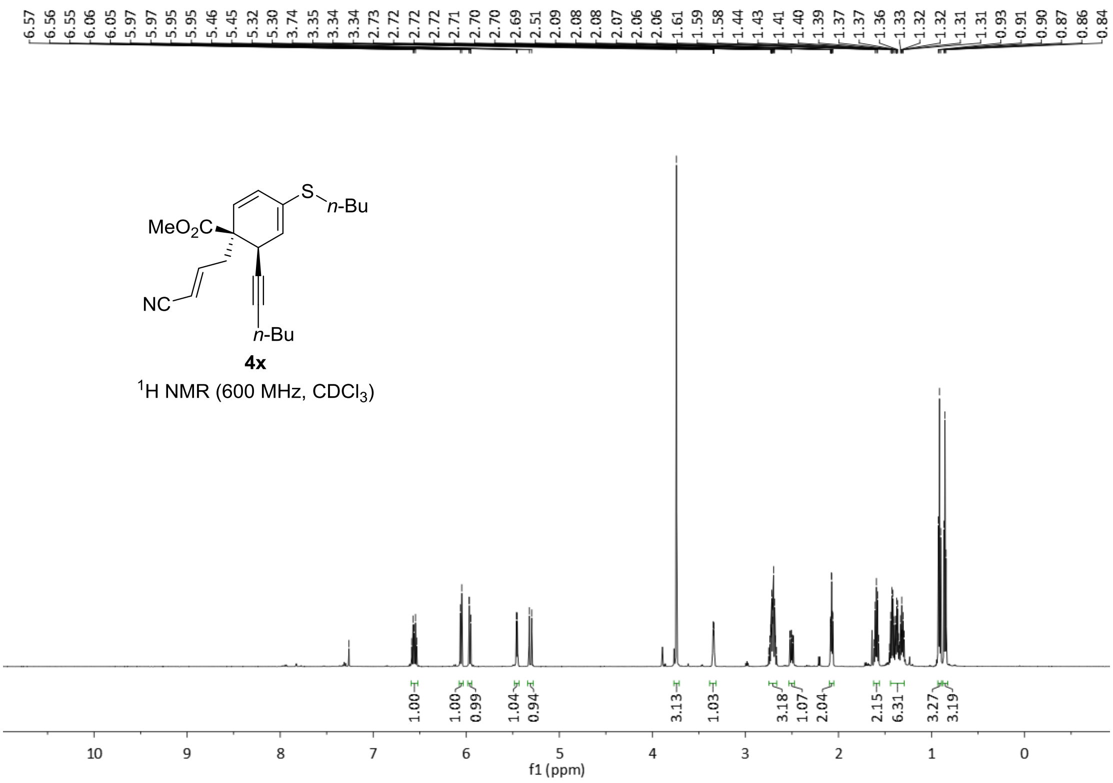

Supplementary Figure 111. ^{1}H NMR spectrum of **4x**

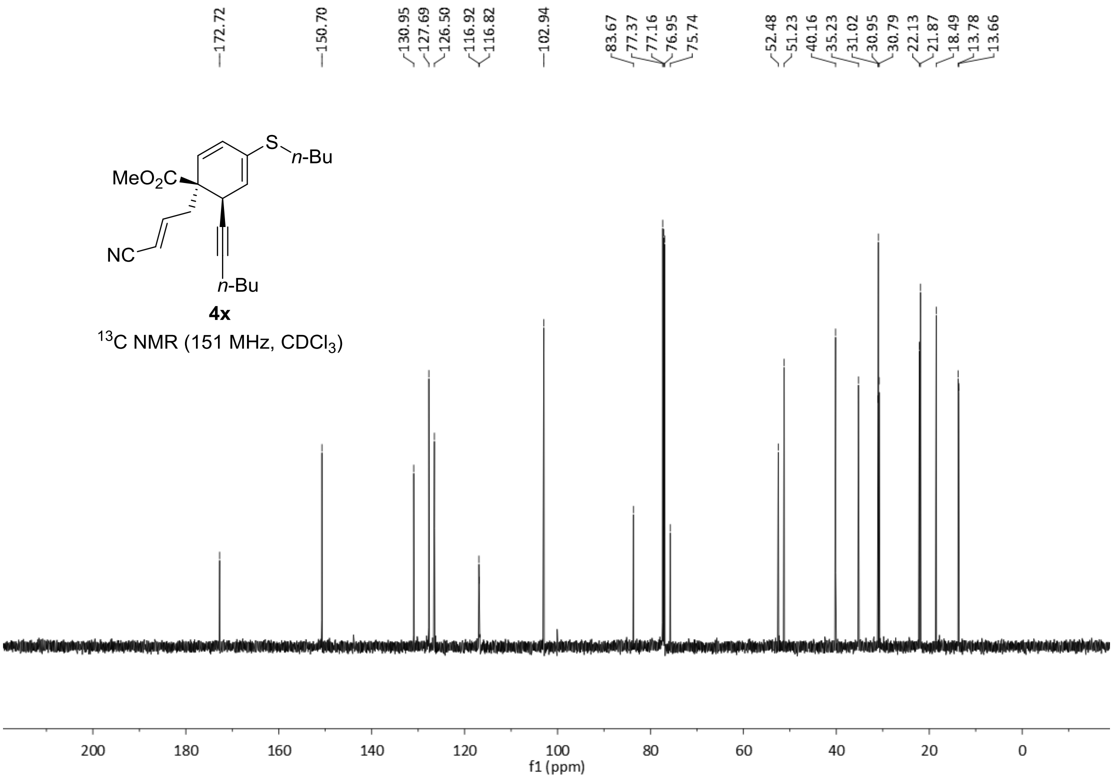

Supplementary Figure 112. ^{13}C NMR spectrum of **4x**

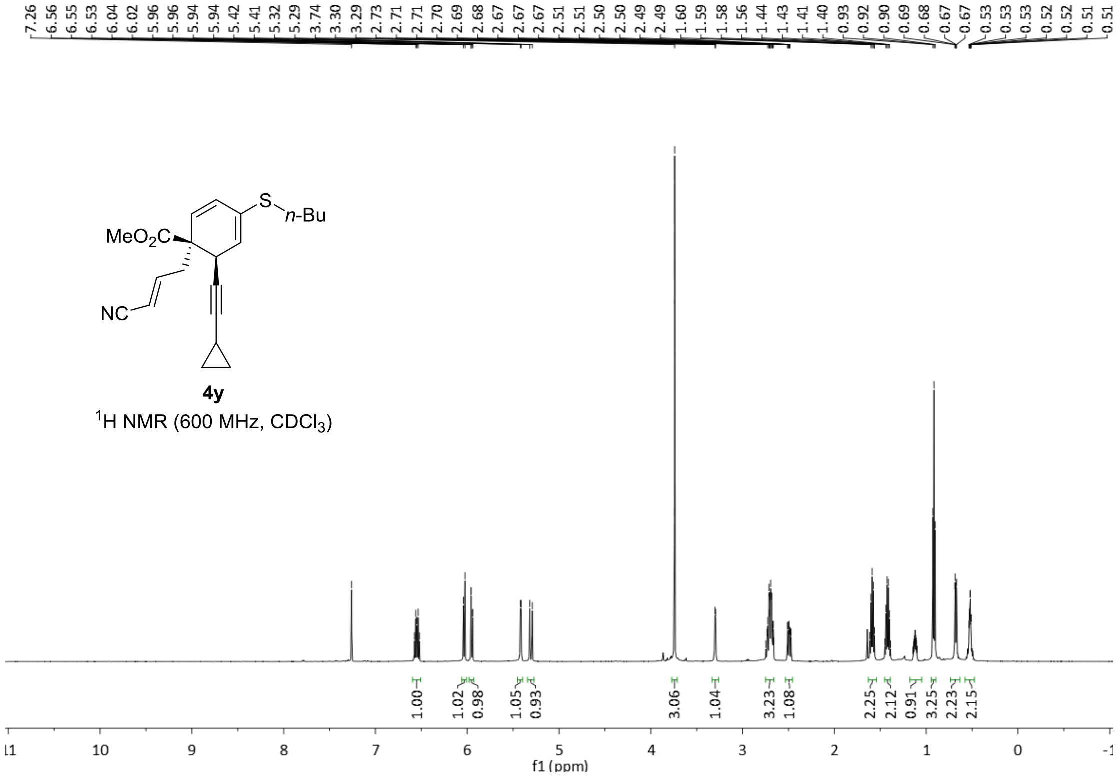

Supplementary Figure 113. ^{1}H NMR spectrum of **4y**

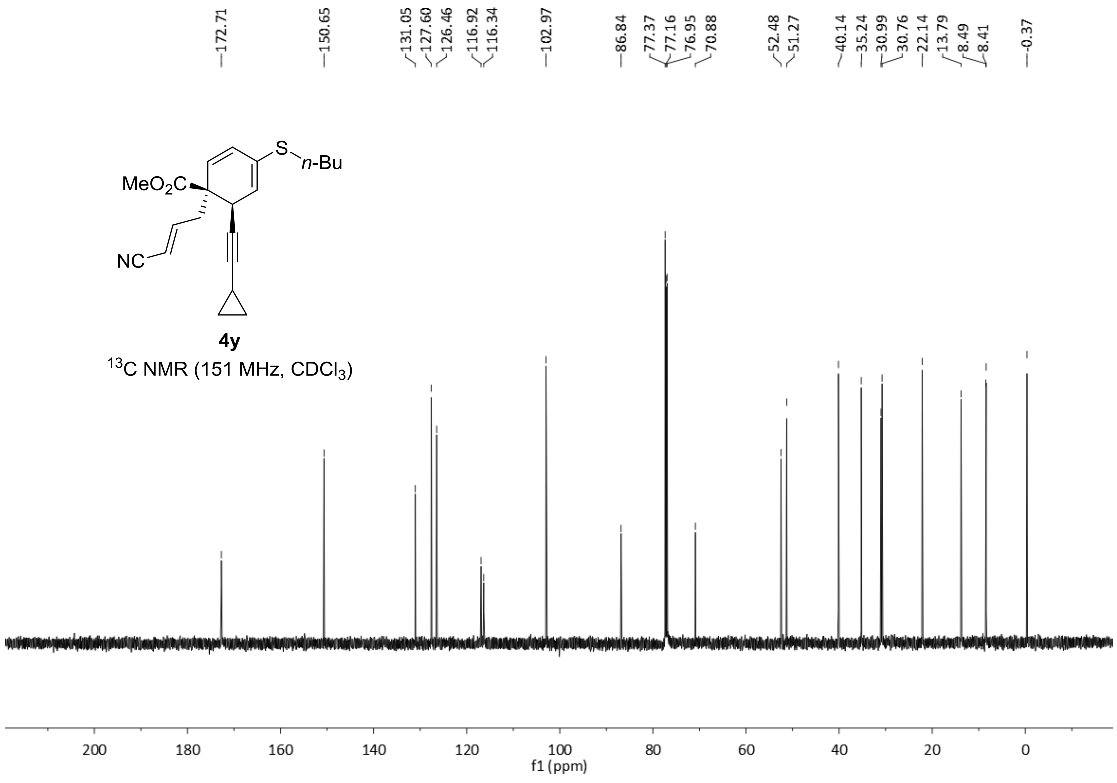

Supplementary Figure 114. ^{13}C NMR spectrum of **4y**

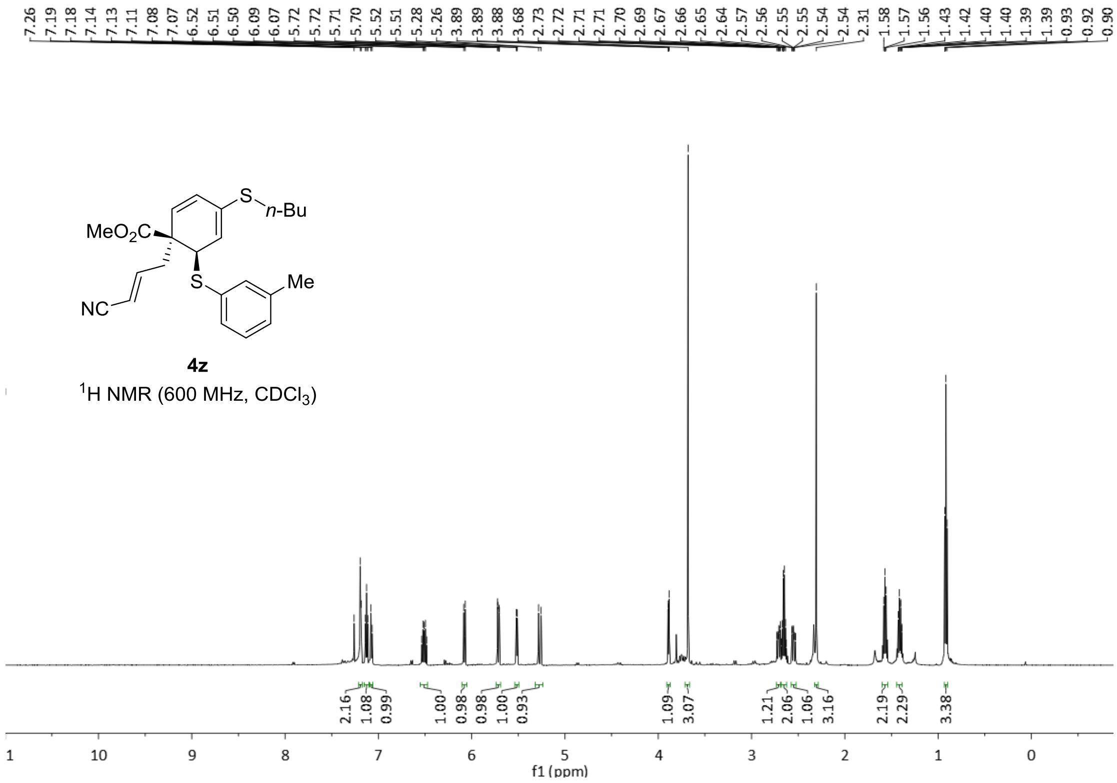

Supplementary Figure 115. ^{1}H NMR spectrum of **4z**

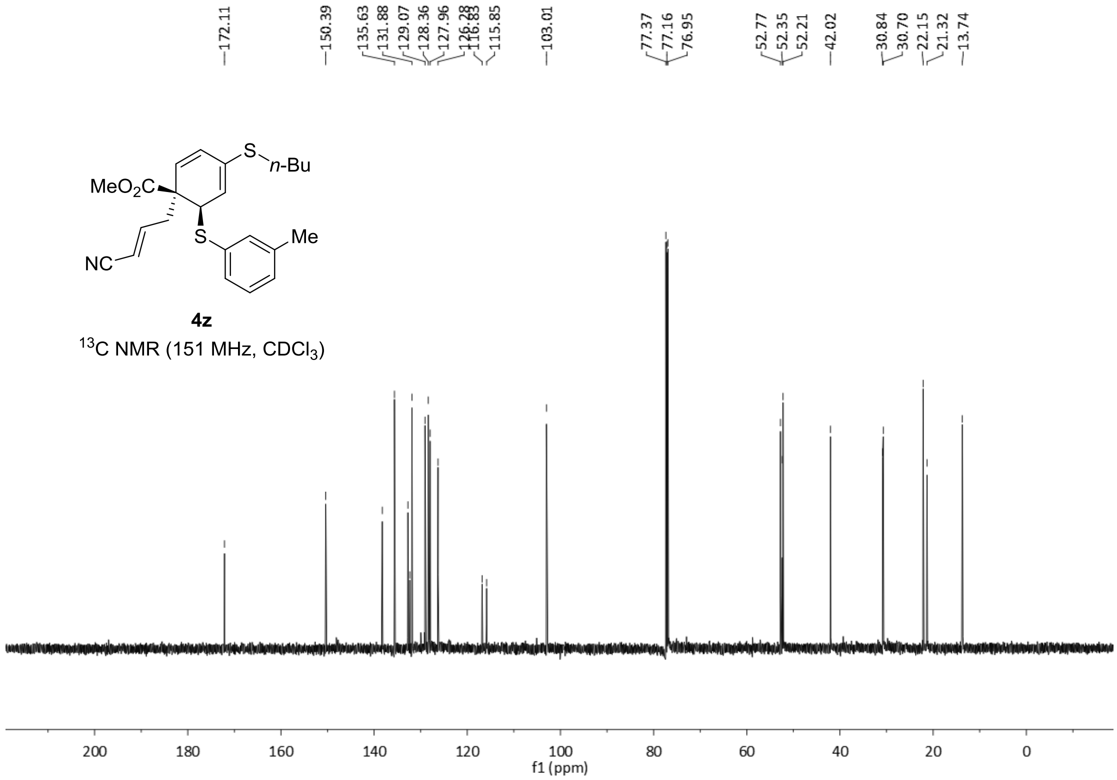

Supplementary Figure 116. ^{13}C NMR spectrum of **4z**

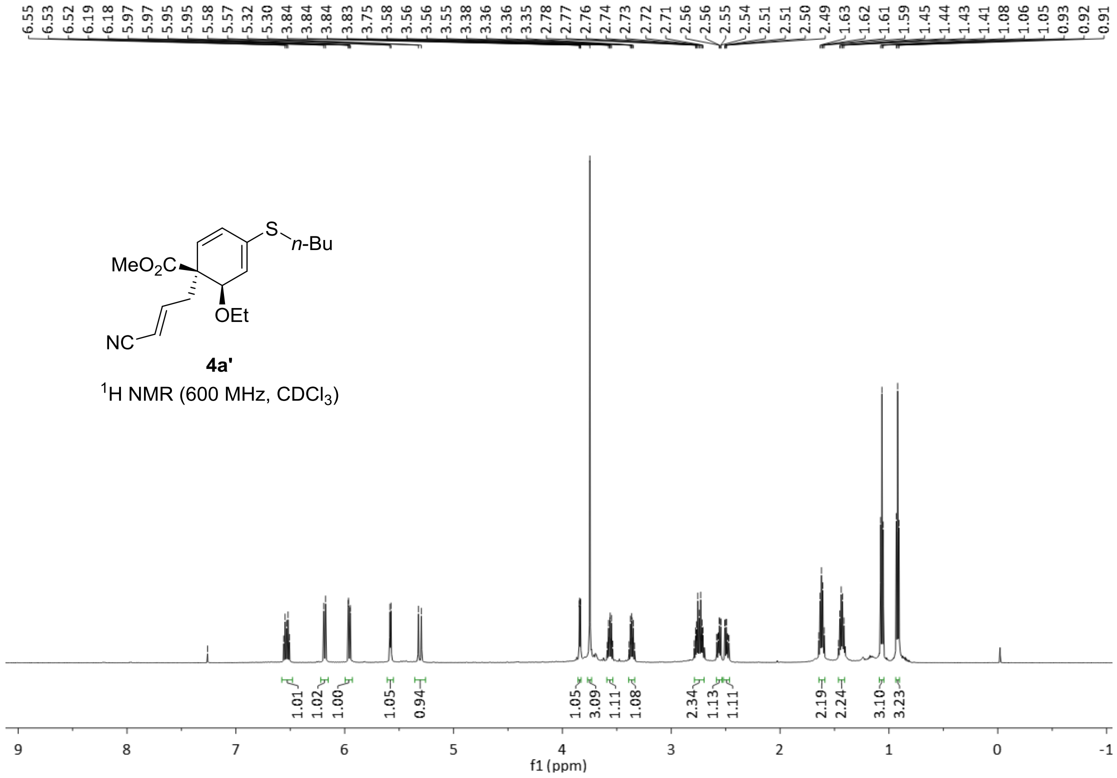

Supplementary Figure 117. ^{1}H NMR spectrum of **4a'**

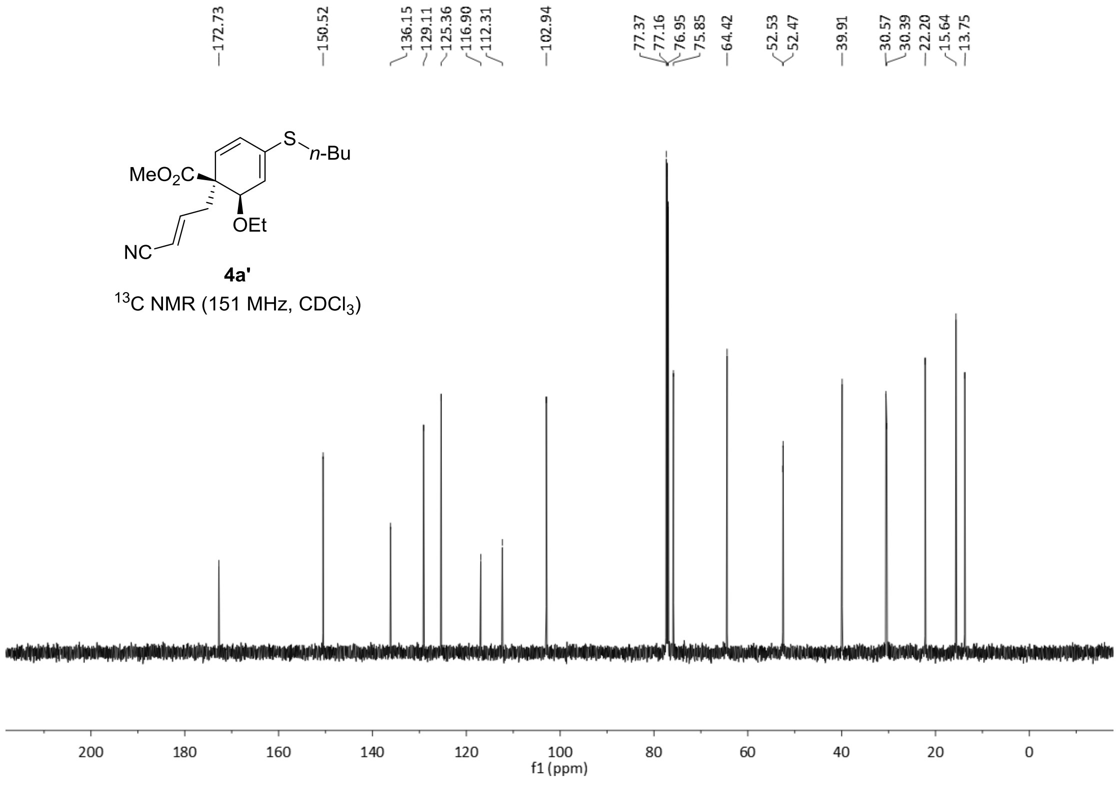

Supplementary Figure 118. ^{13}C NMR spectrum of **4a'**

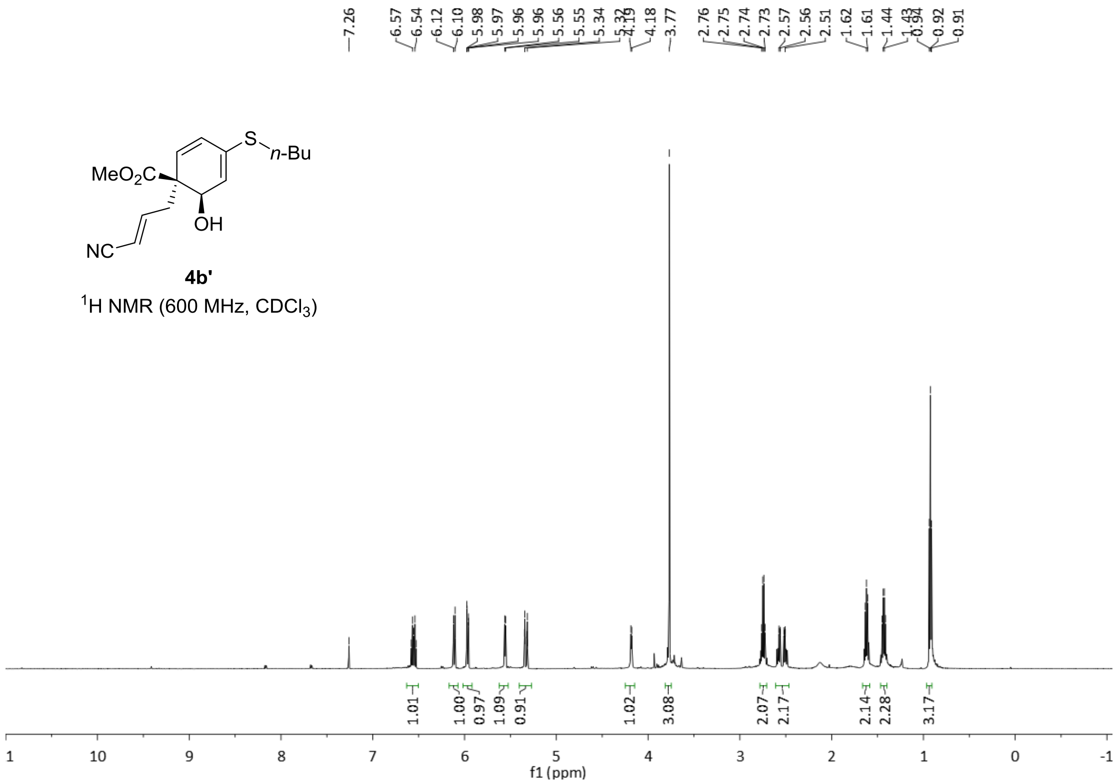

Supplementary Figure 119. ^{1}H NMR spectrum of **4b’**

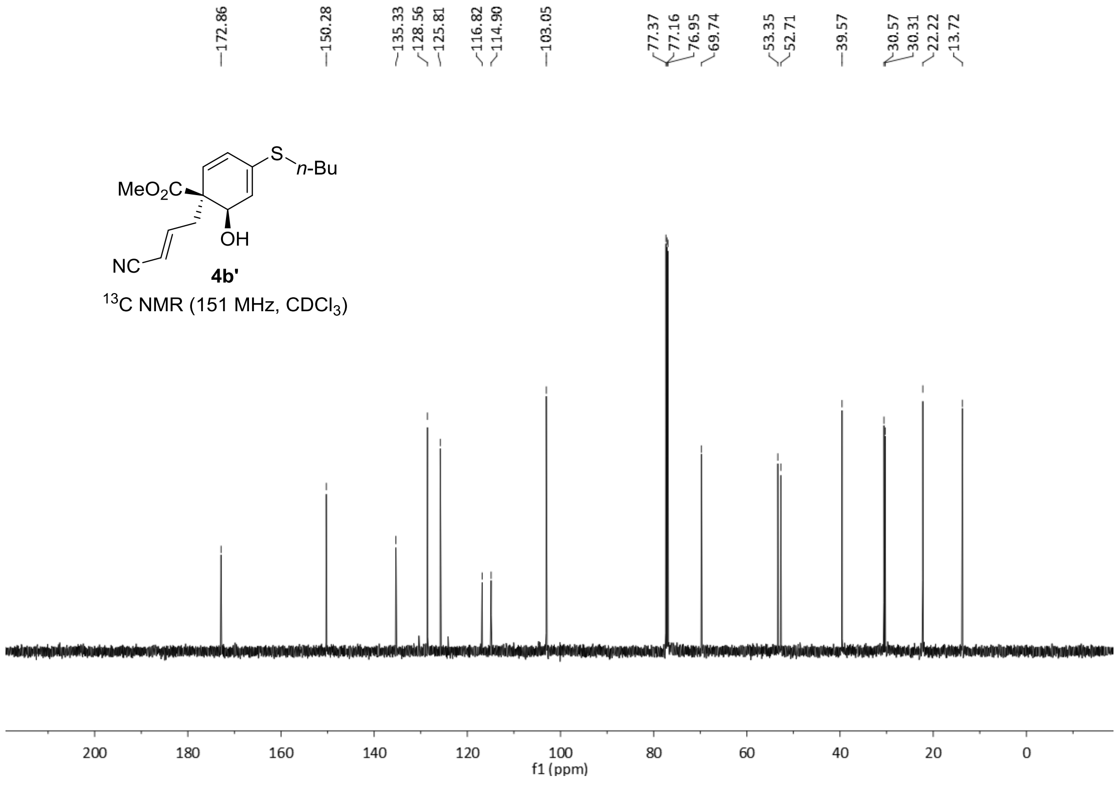

Supplementary Figure 120. ^{13}C NMR spectrum of **4b’**

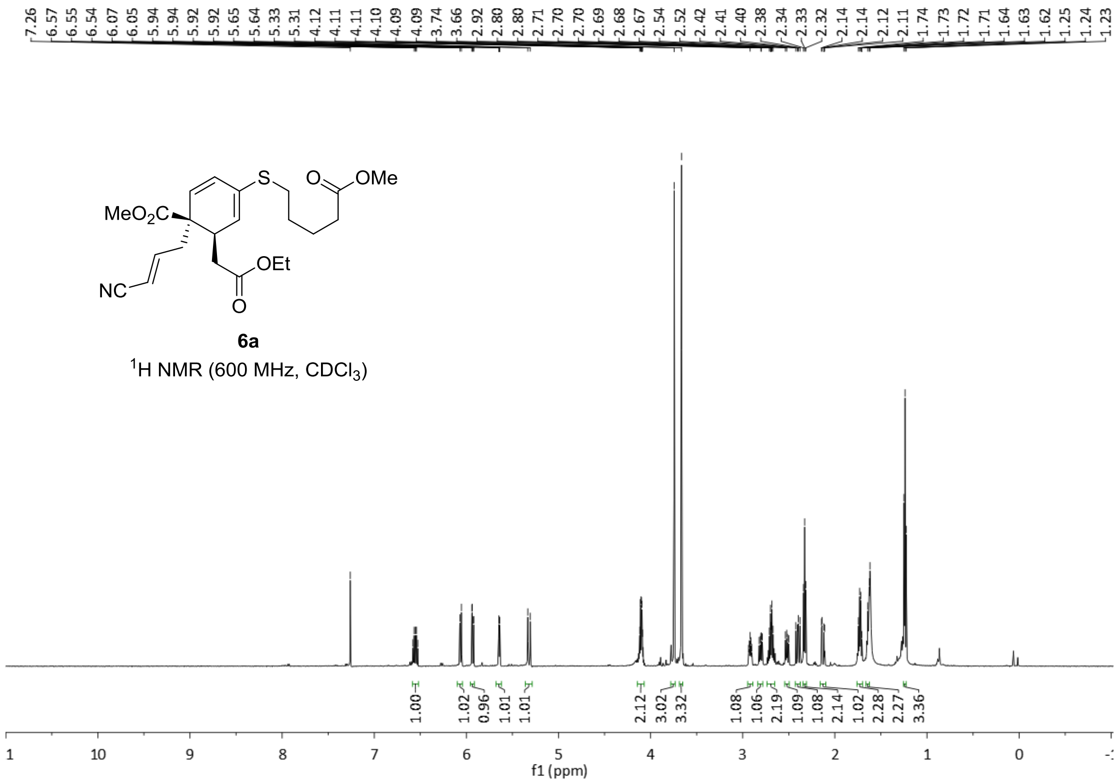

Supplementary Figure 121. ^{1}H NMR spectrum of **6a**

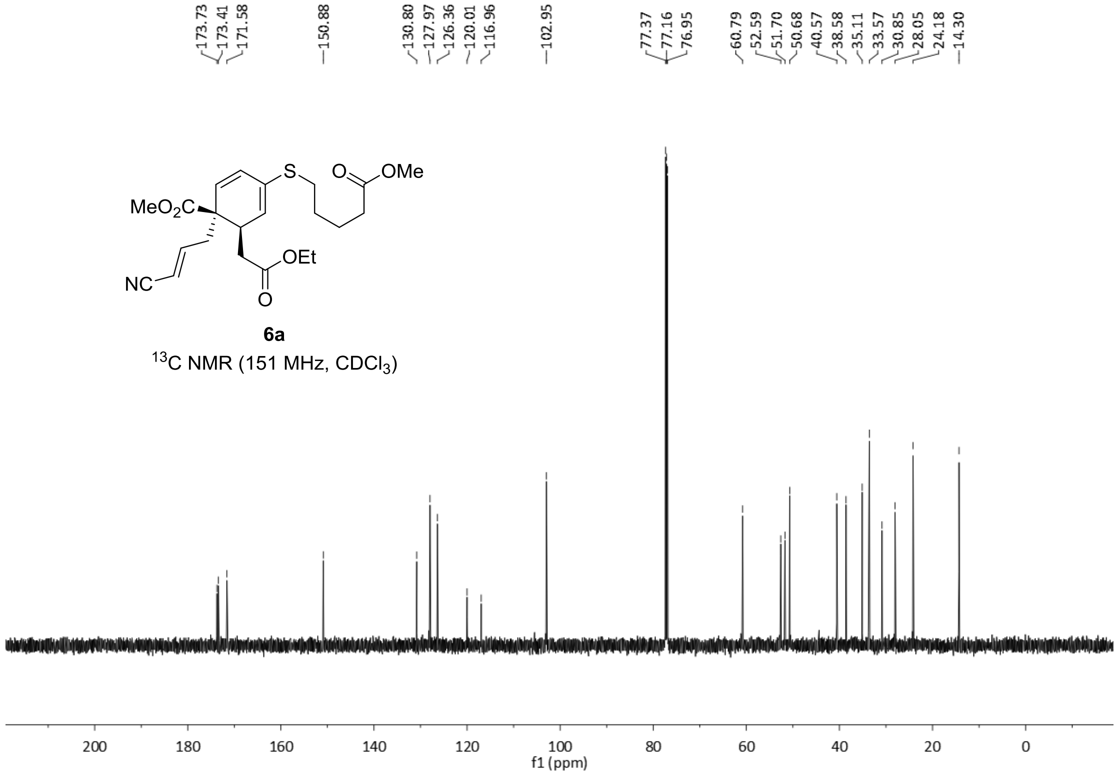

Supplementary Figure 122. ^{13}C NMR spectrum of **6a**

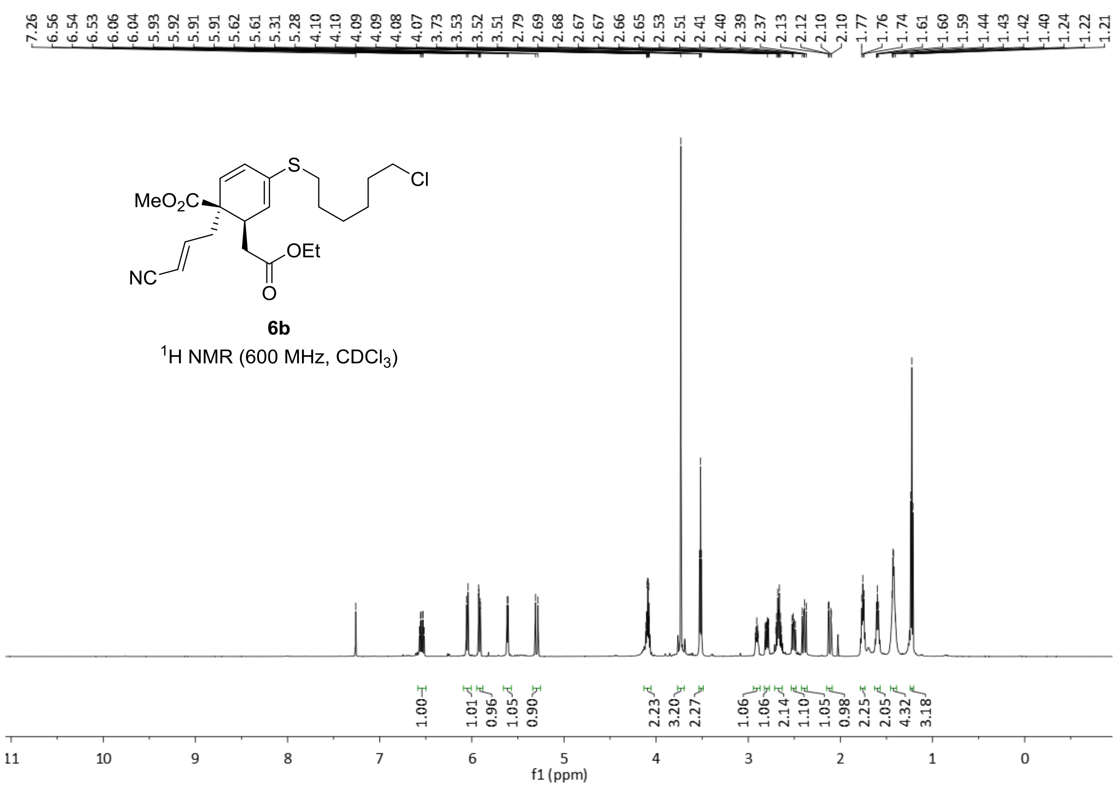

Supplementary Figure 123. ^{1}H NMR spectrum of **6b**

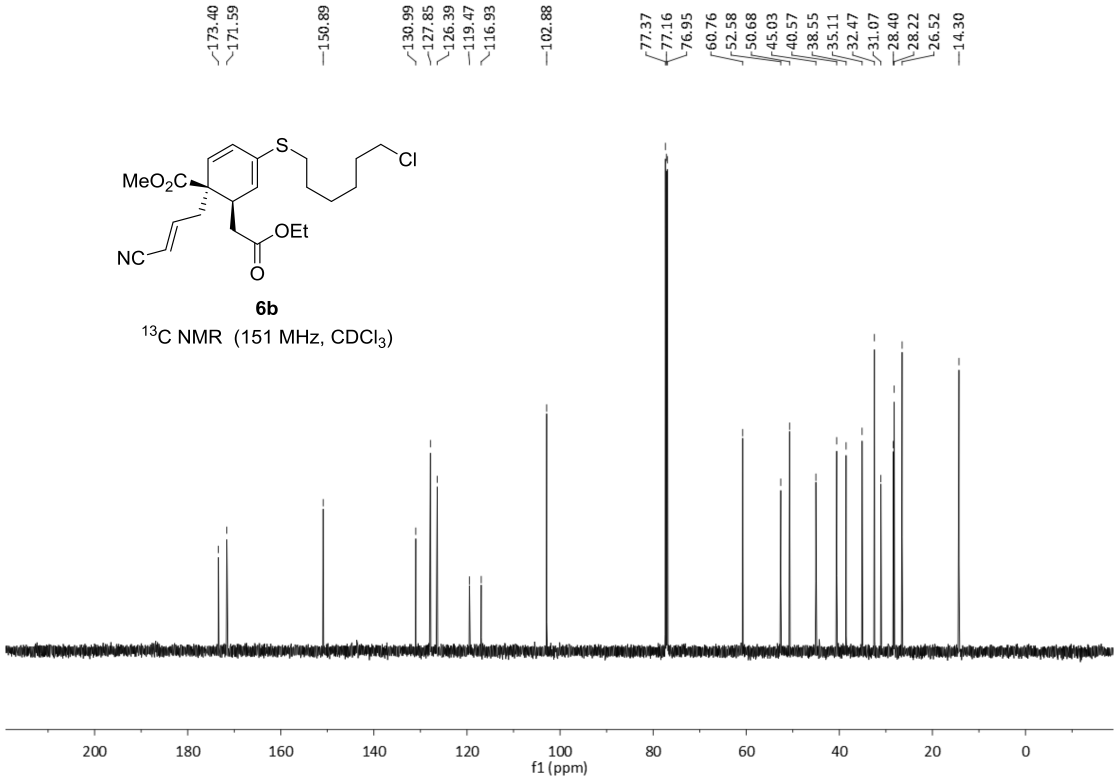

Supplementary Figure 124. ^{13}C NMR spectrum of **6b**

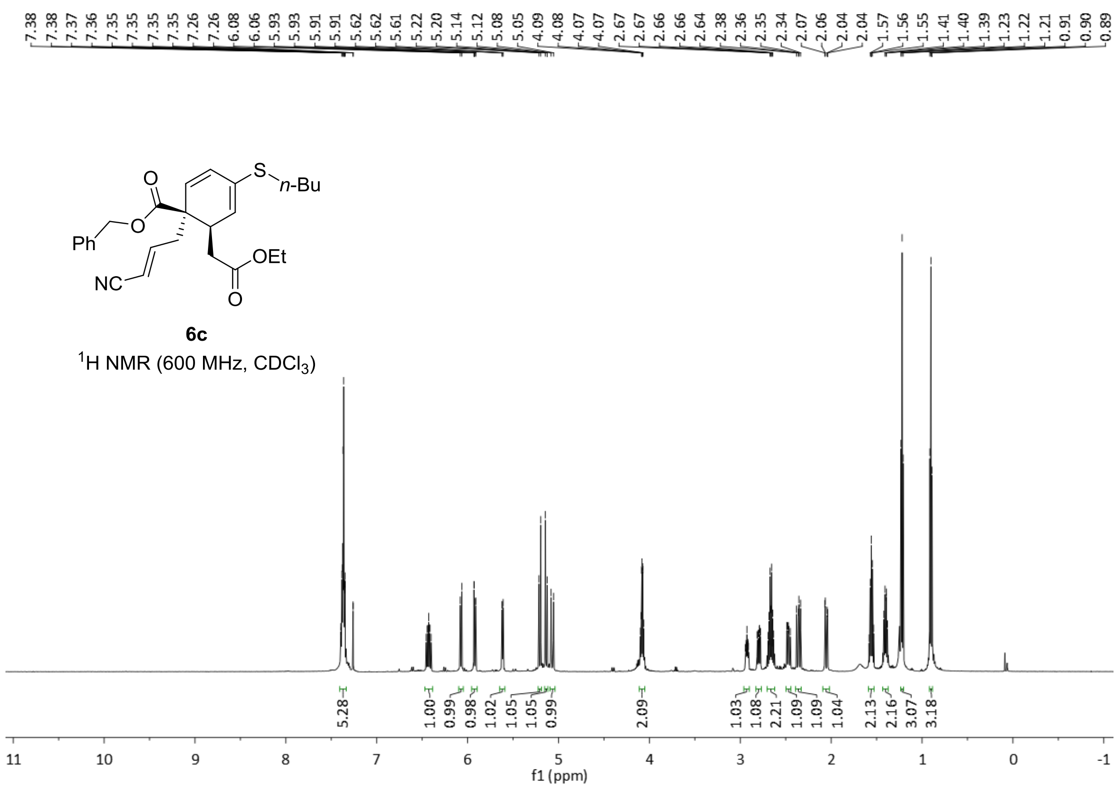

Supplementary Figure 125. ^{1}H NMR spectrum of **6c**

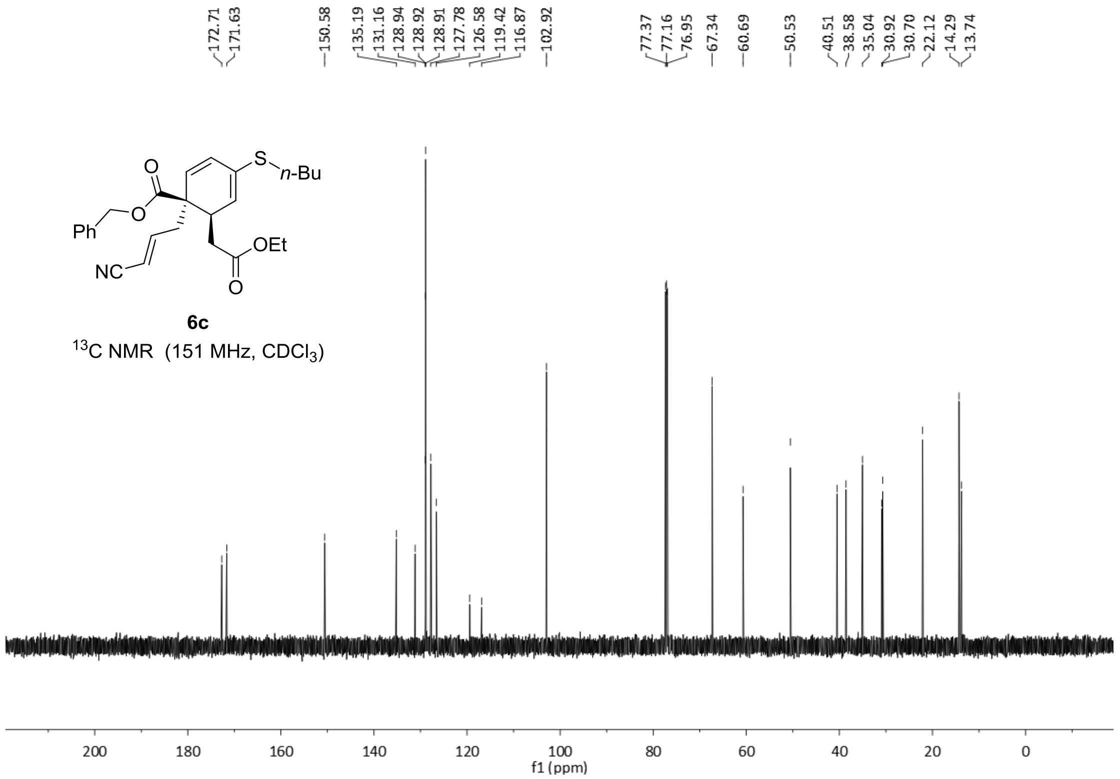

Supplementary Figure 126. ^{13}C NMR spectrum of **6c**

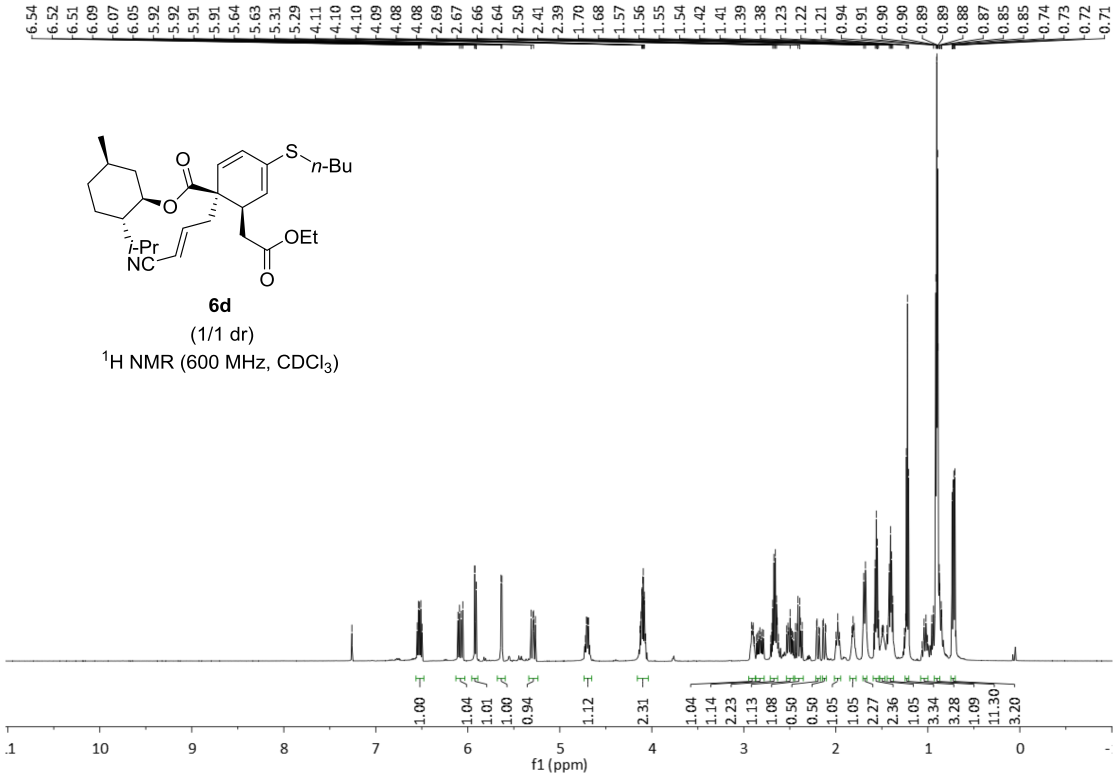

Supplementary Figure 127. ^{1}H NMR spectrum of **6d**

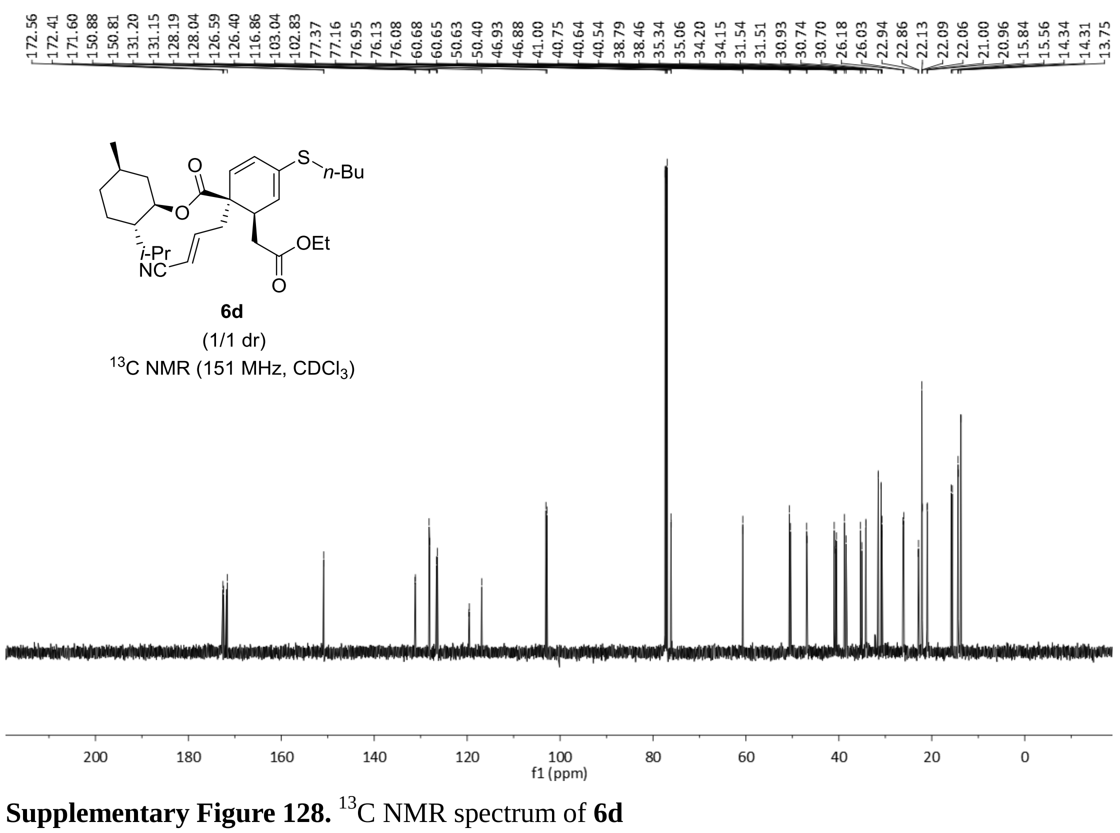

Supplementary Figure 128. ^{13}C NMR spectrum of **6d**

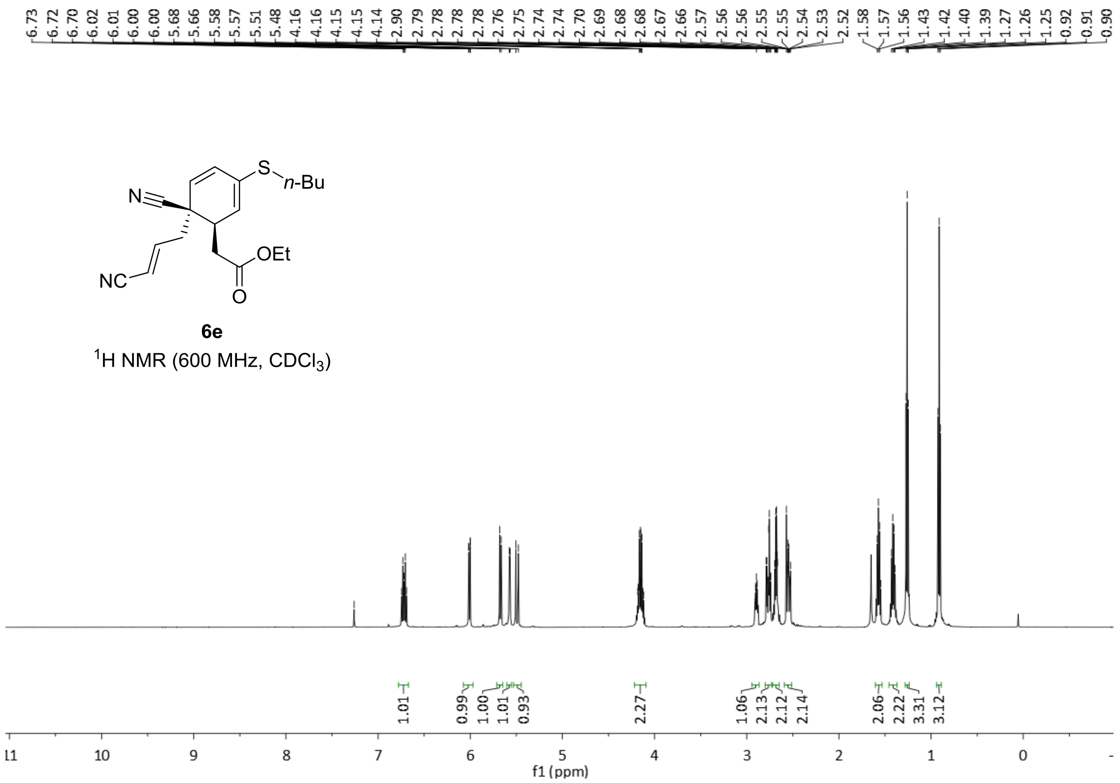

Supplementary Figure 129. ^{1}H NMR spectrum of **6e**

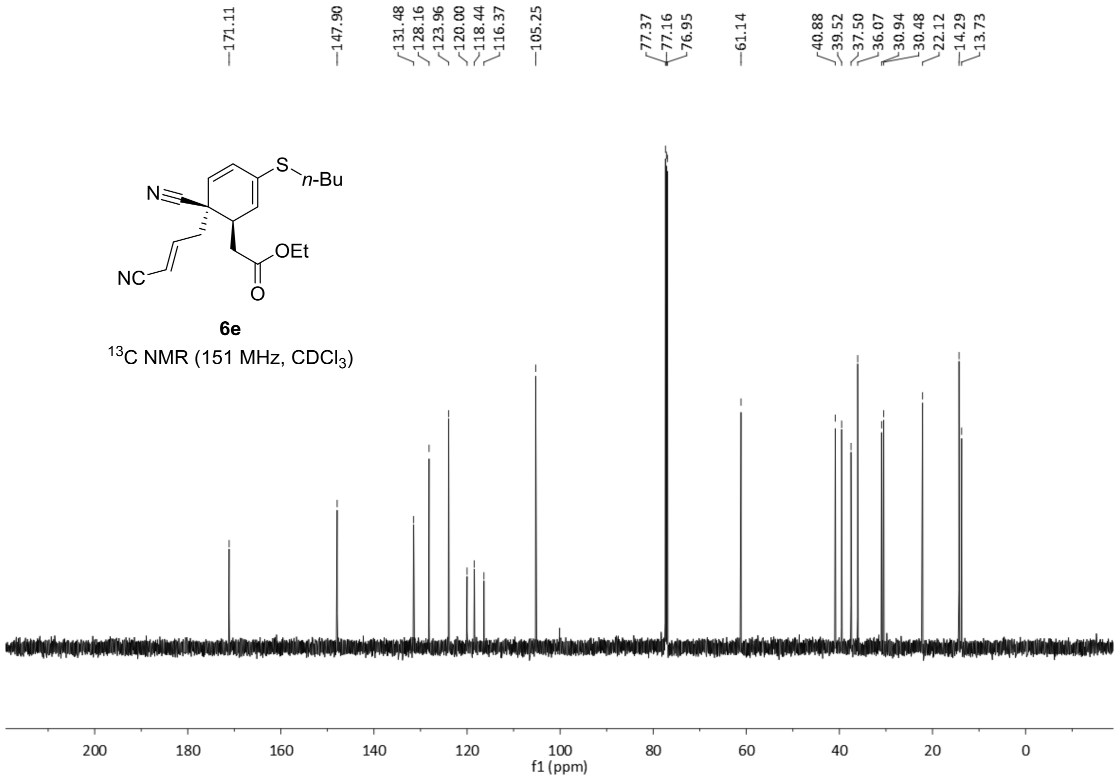

Supplementary Figure 130. ^{13}C NMR spectrum of **6e**

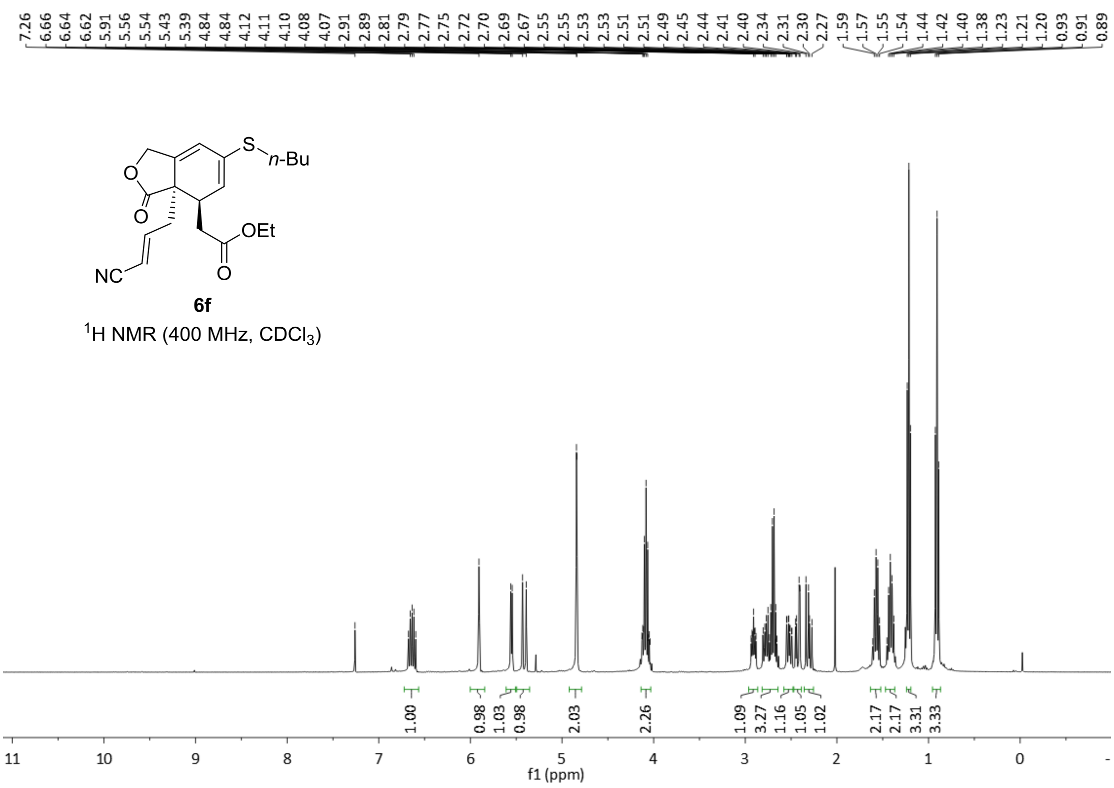

Supplementary Figure 131. ^{1}H NMR spectrum of **6f**

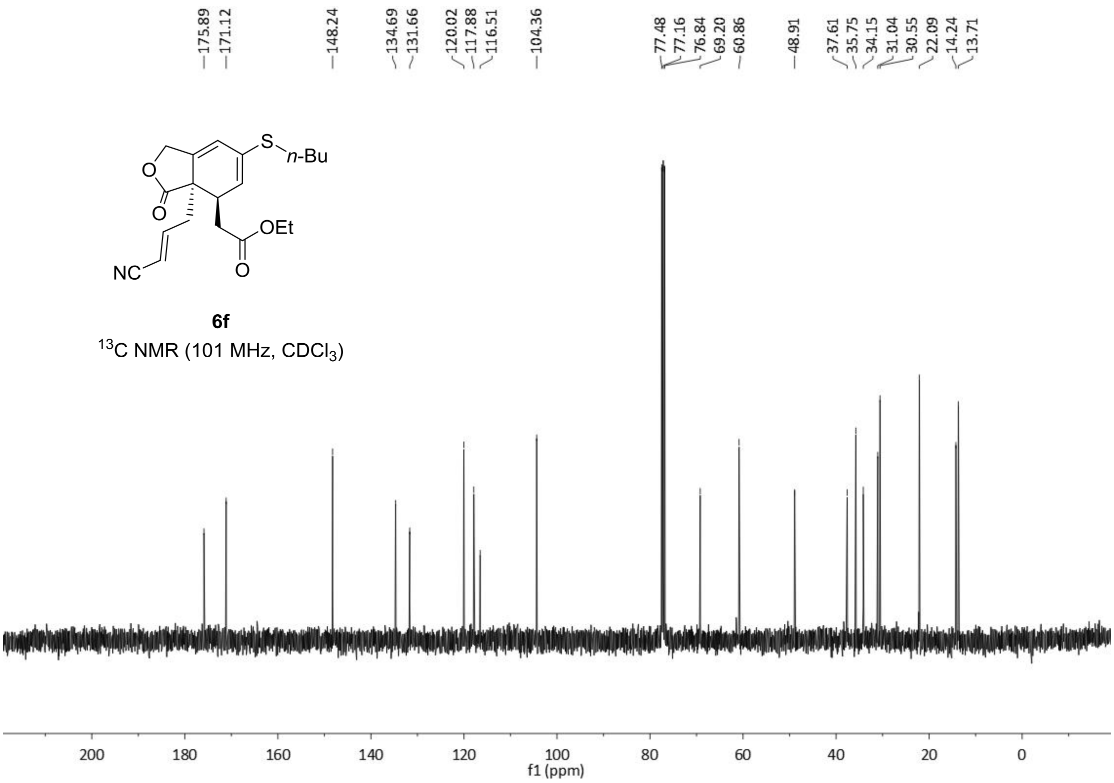

Supplementary Figure 132. ^{13}C NMR spectrum of **6f**

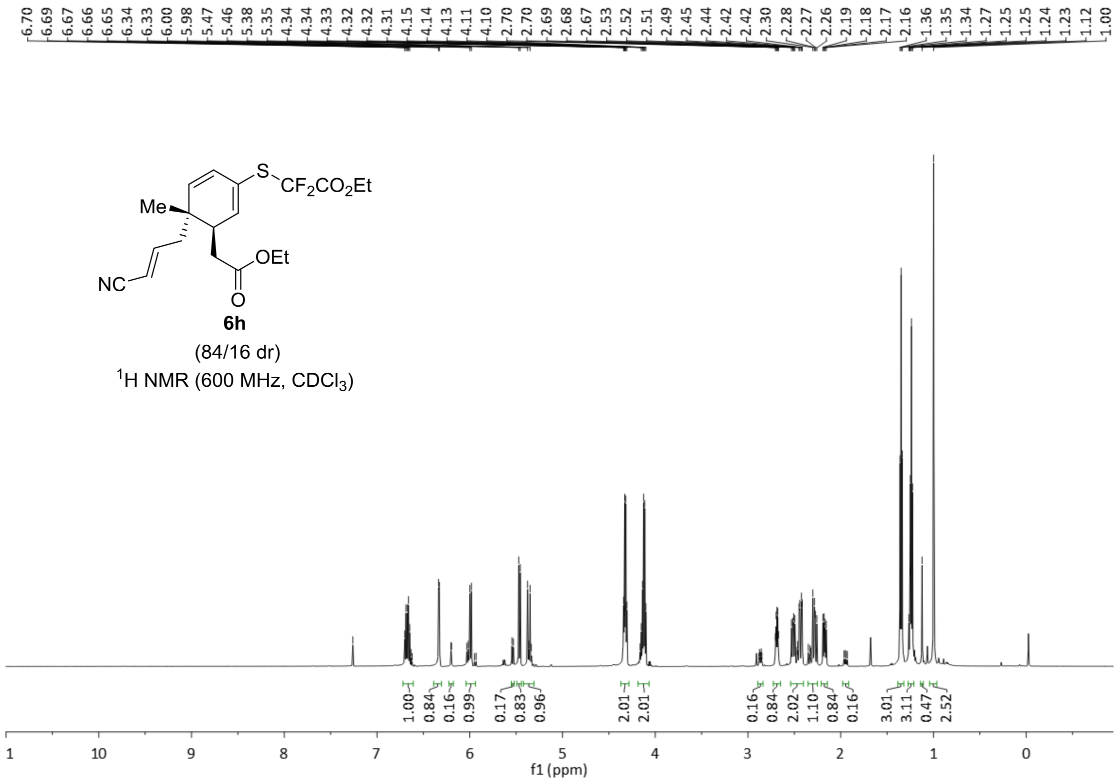

Supplementary Figure 133. ^{1}H NMR spectrum of **6h**

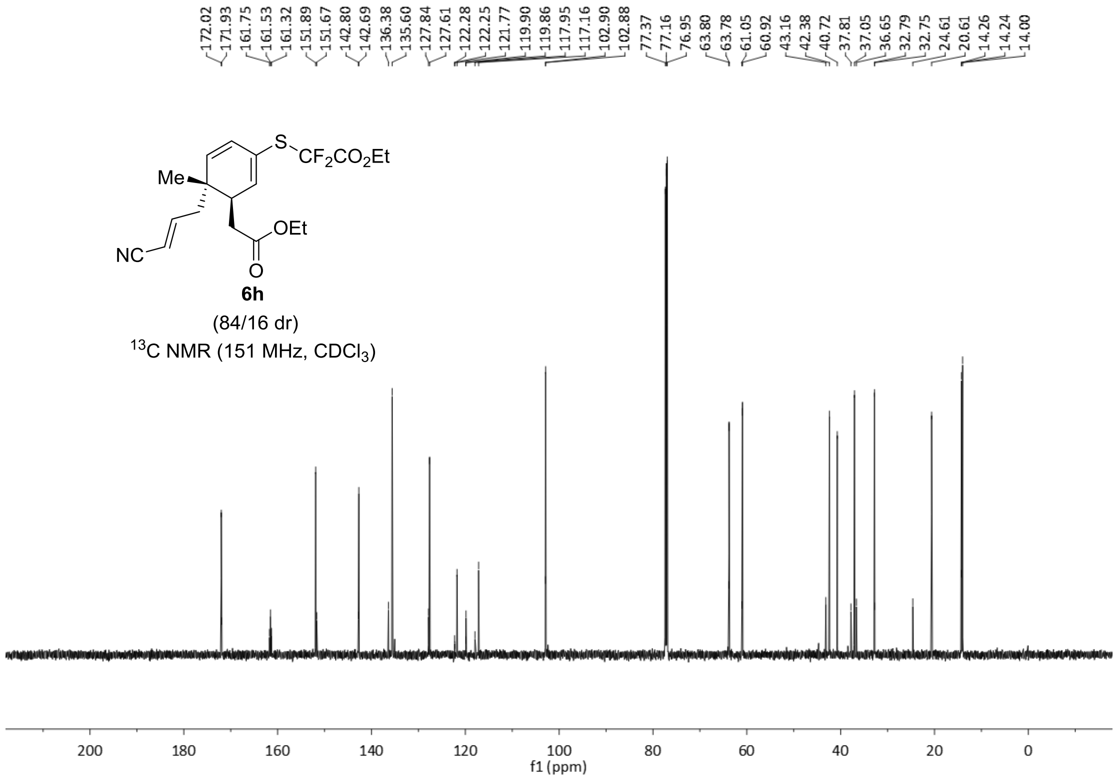

Supplementary Figure 134. ^{13}C NMR spectrum of **6h**

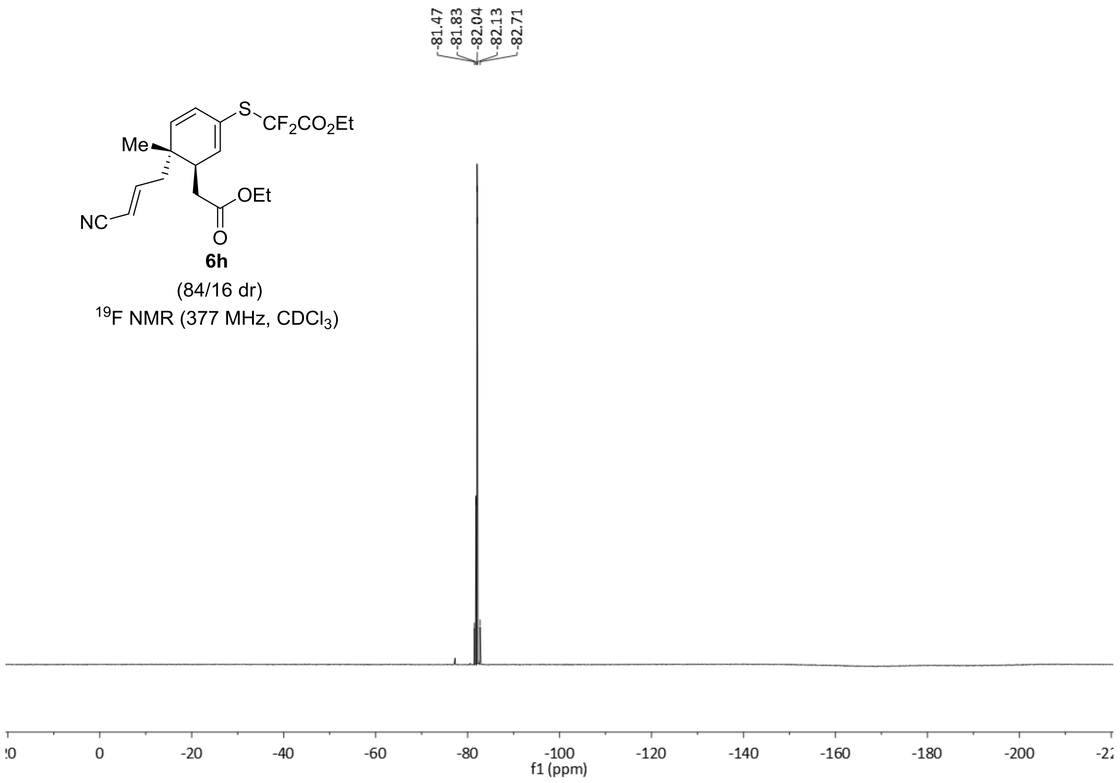

Supplementary Figure 135. ^{19}F NMR spectrum of **6h**

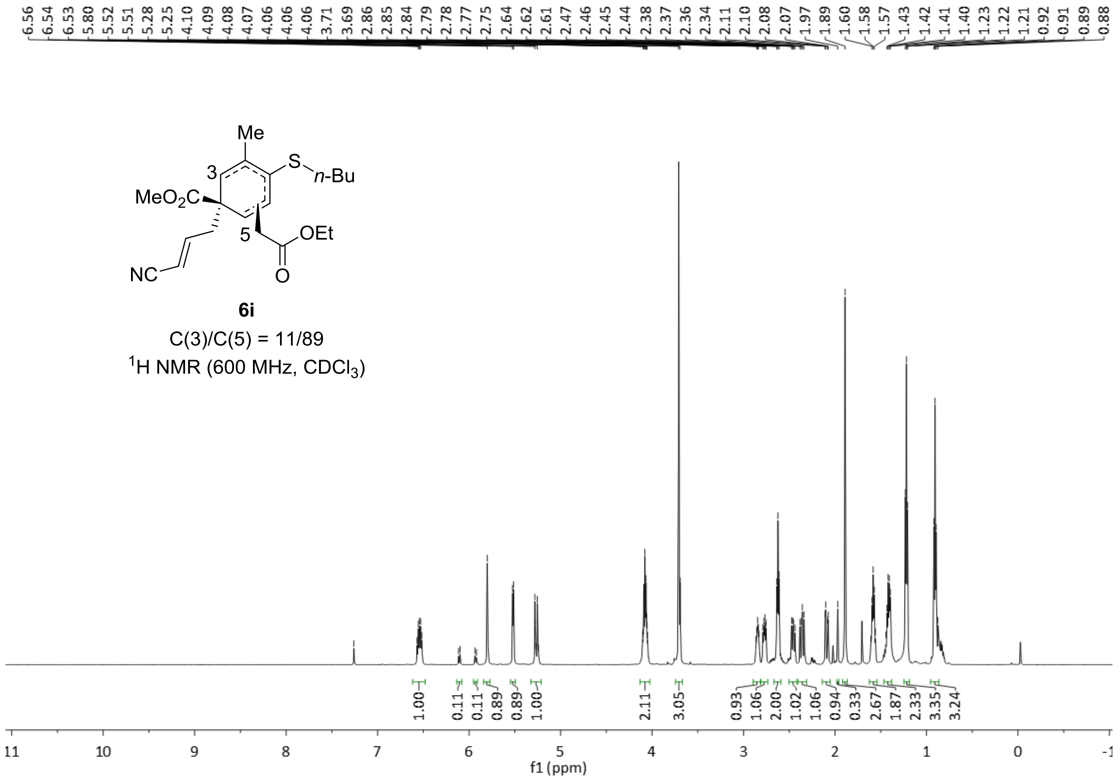

Supplementary Figure 136. ^{1}H NMR spectrum of **6i**

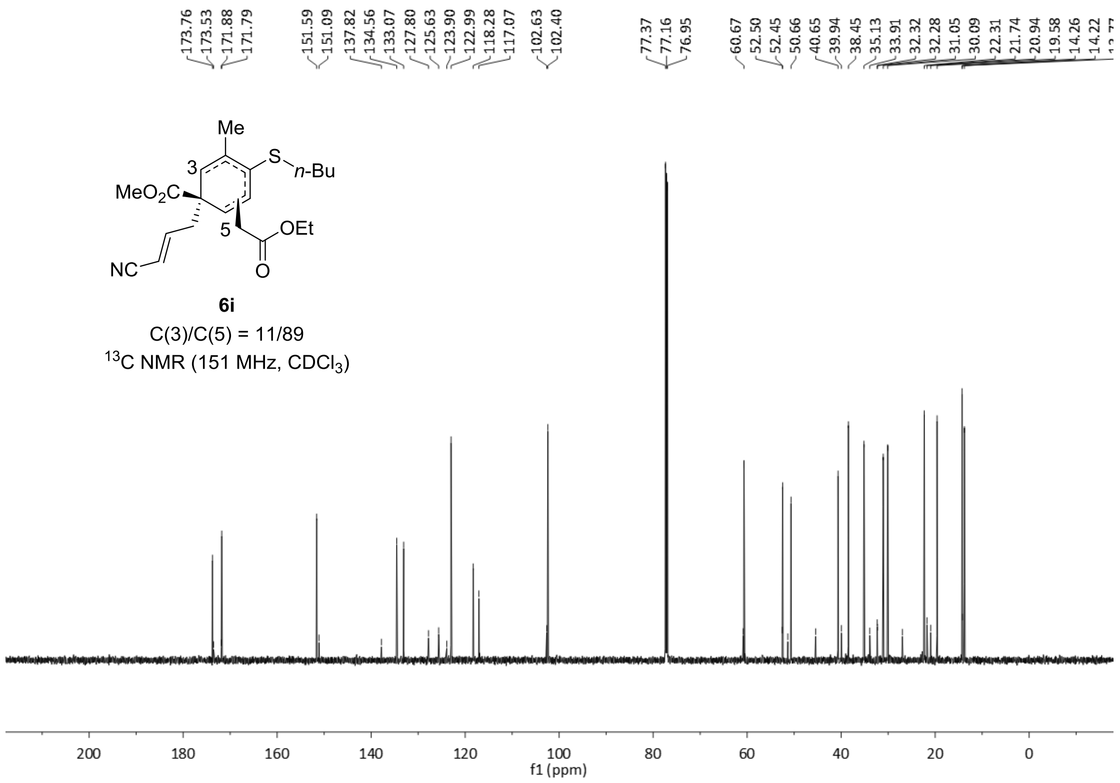

Supplementary Figure 137. ^{13}C NMR spectrum of **6i**

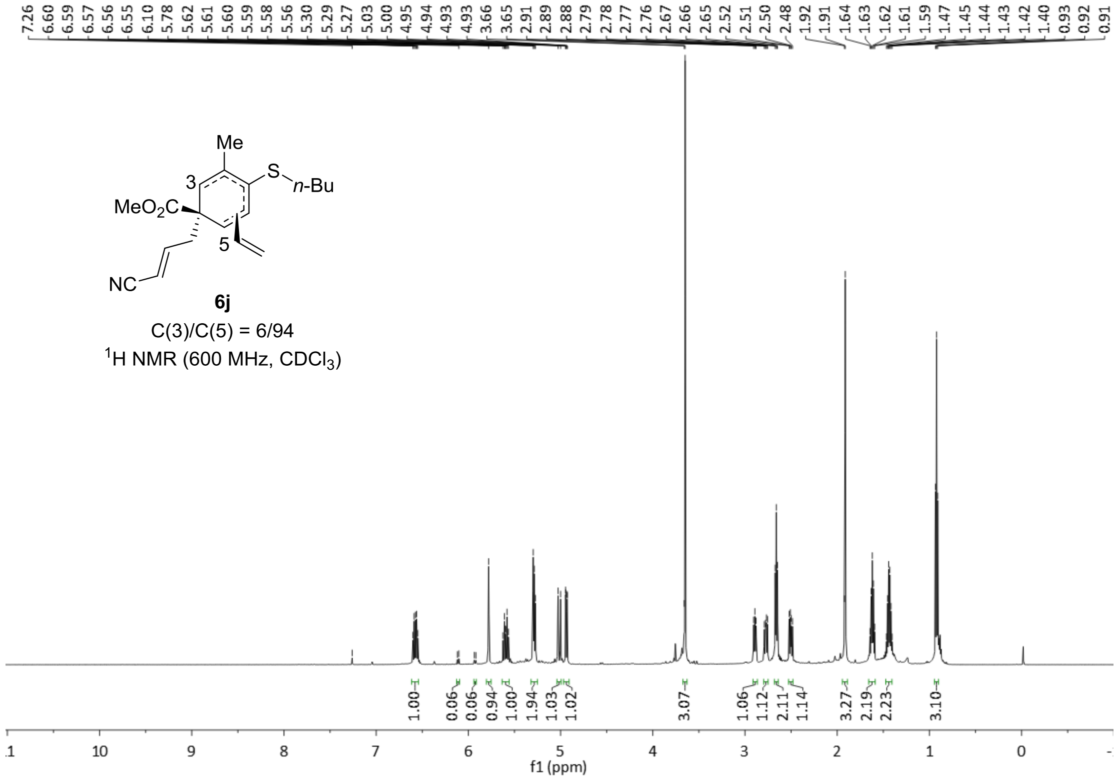

Supplementary Figure 138. ^{1}H NMR spectrum of **6j**

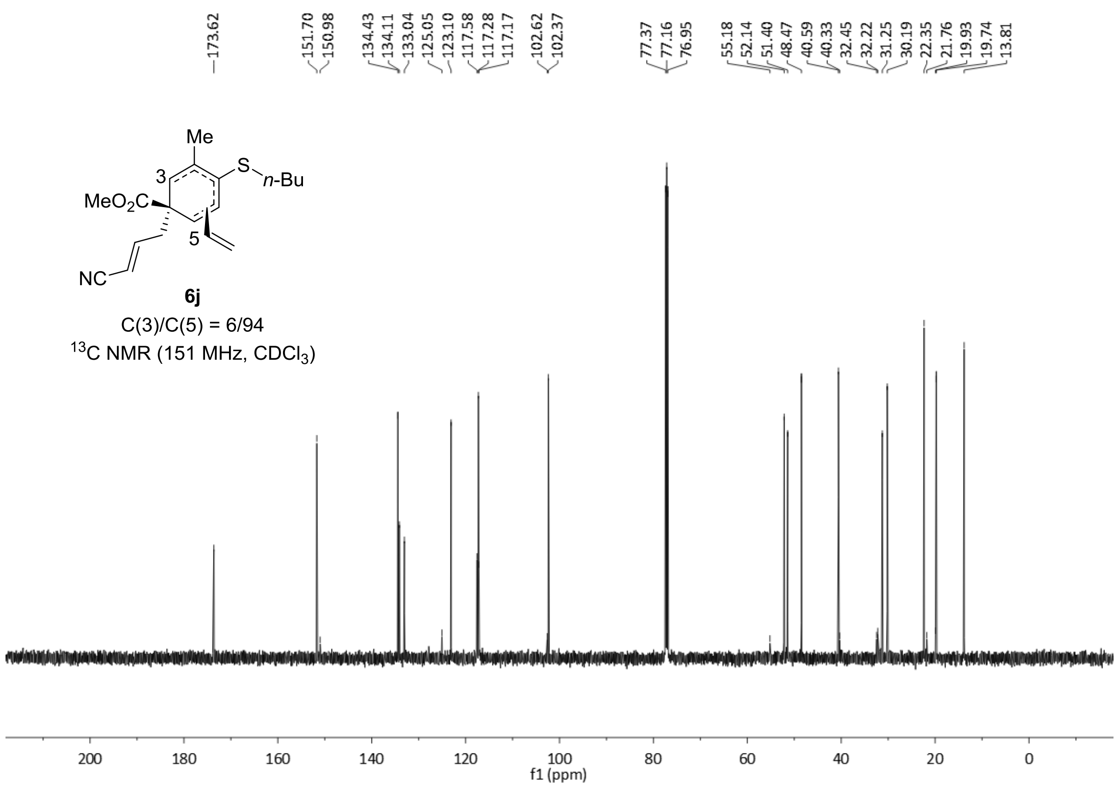

Supplementary Figure 139. ^{13}C NMR spectrum of **6j**

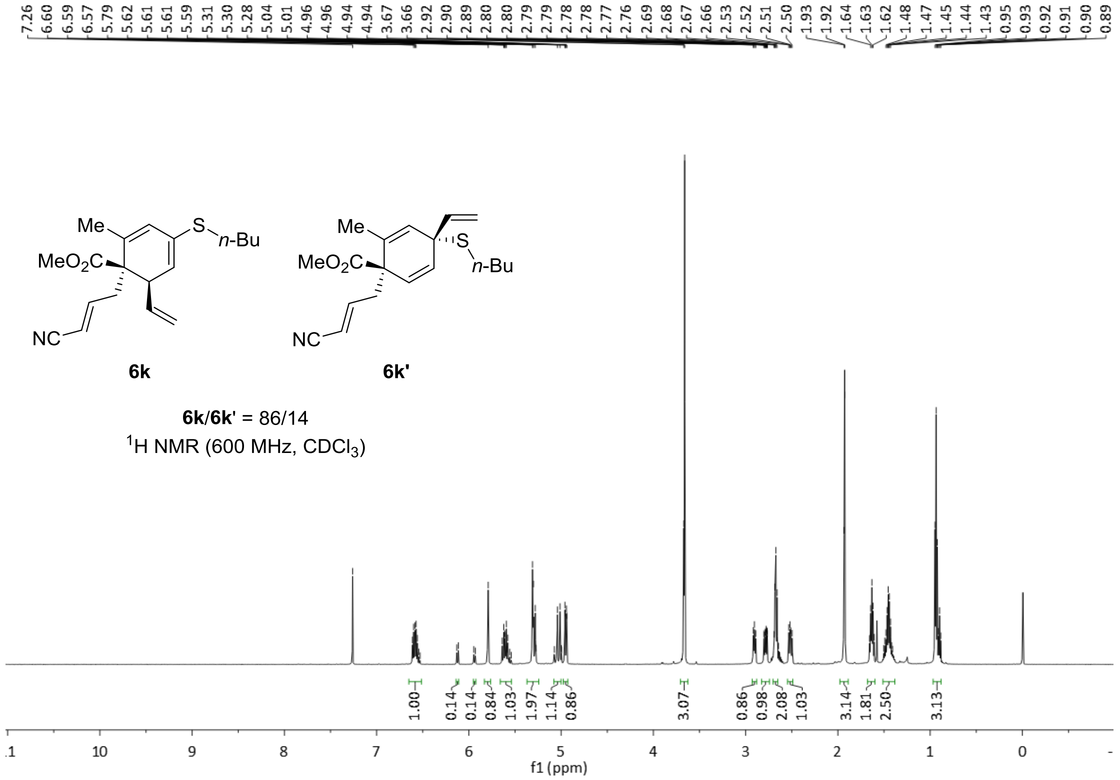

Supplementary Figure 140. ^{1}H NMR spectrum of **6k/6k’**

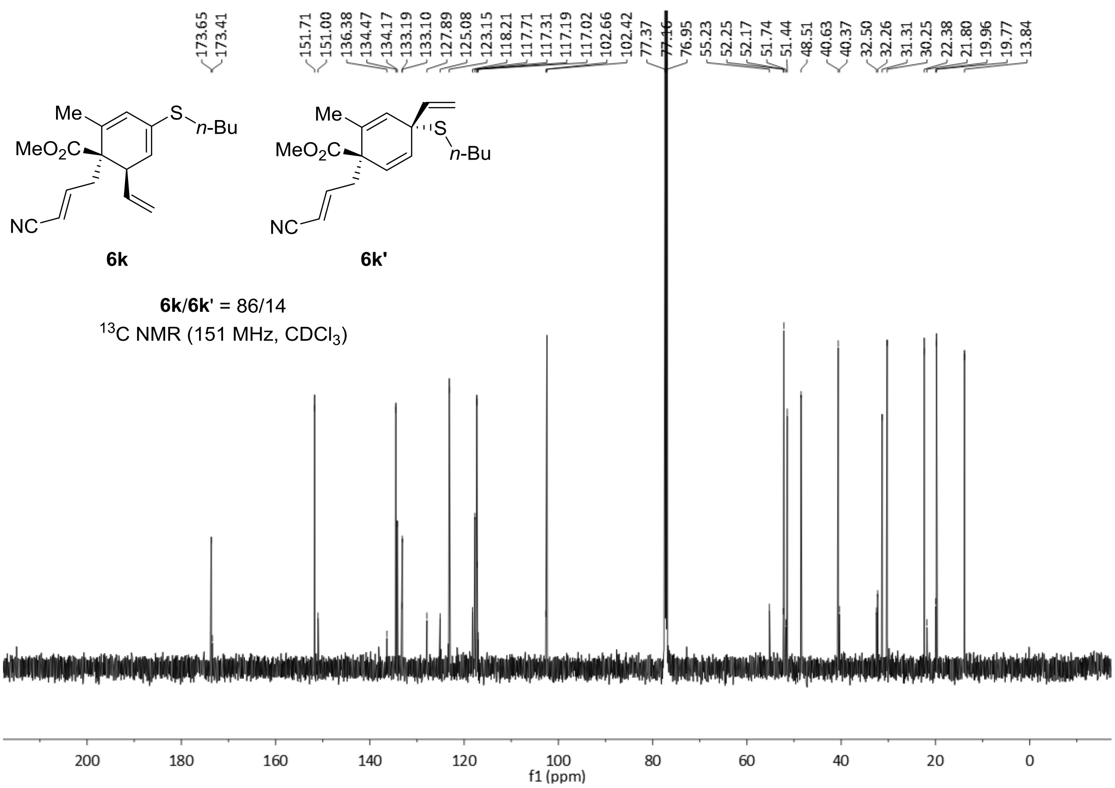

Supplementary Figure 141. ^{13}C NMR spectrum of **6k/6k'**

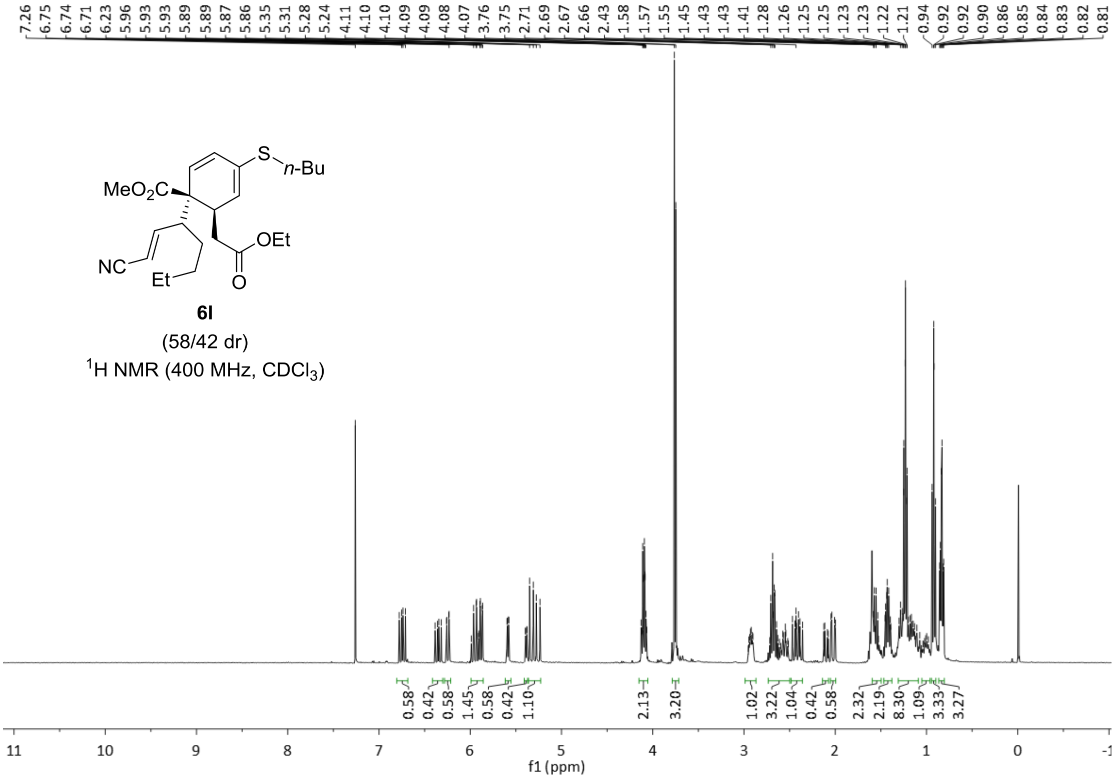

Supplementary Figure 142. ^{1}H NMR spectrum of **6l**

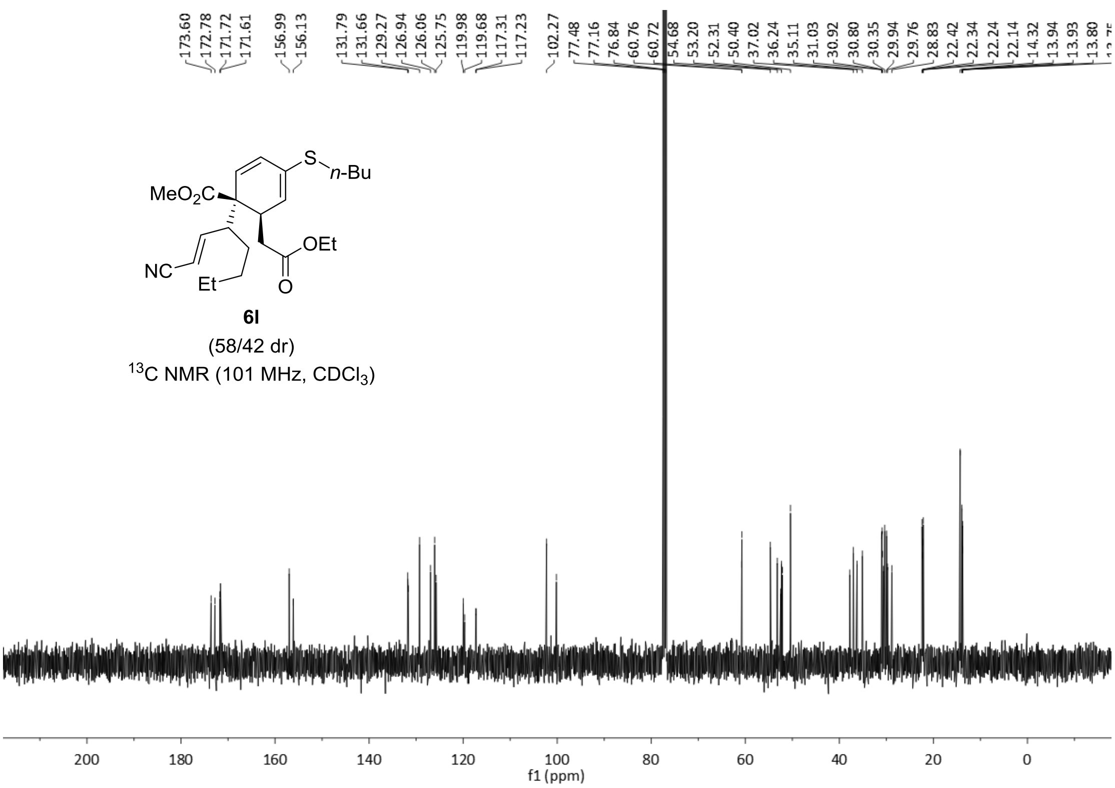

Supplementary Figure 143. ^{13}C NMR spectrum of **6l**

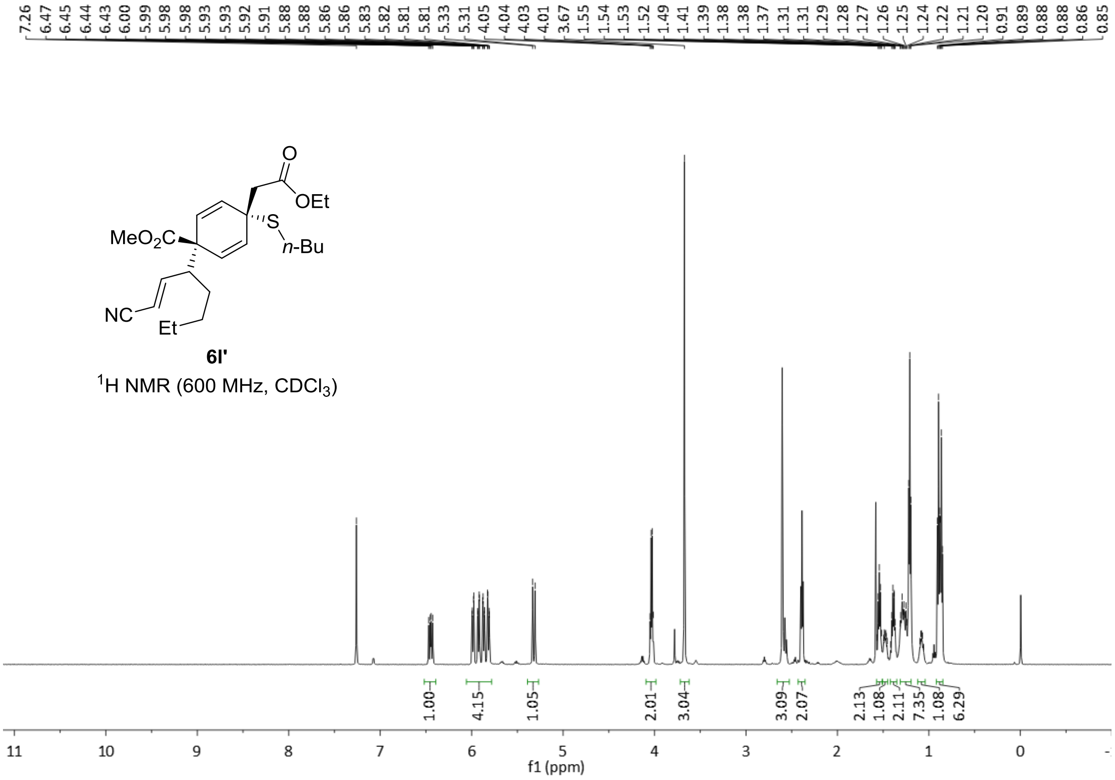

Supplementary Figure 144. ^{1}H NMR spectrum of **6l’**

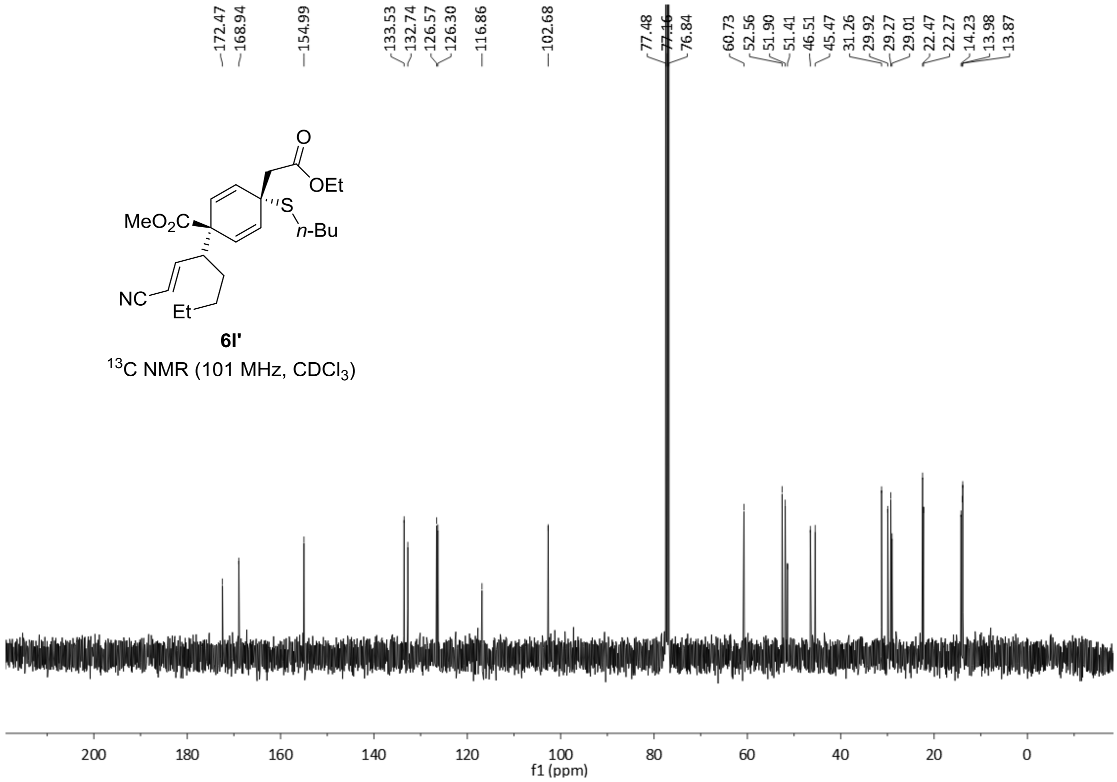

Supplementary Figure 145. ^{13}C NMR spectrum of **6l'**

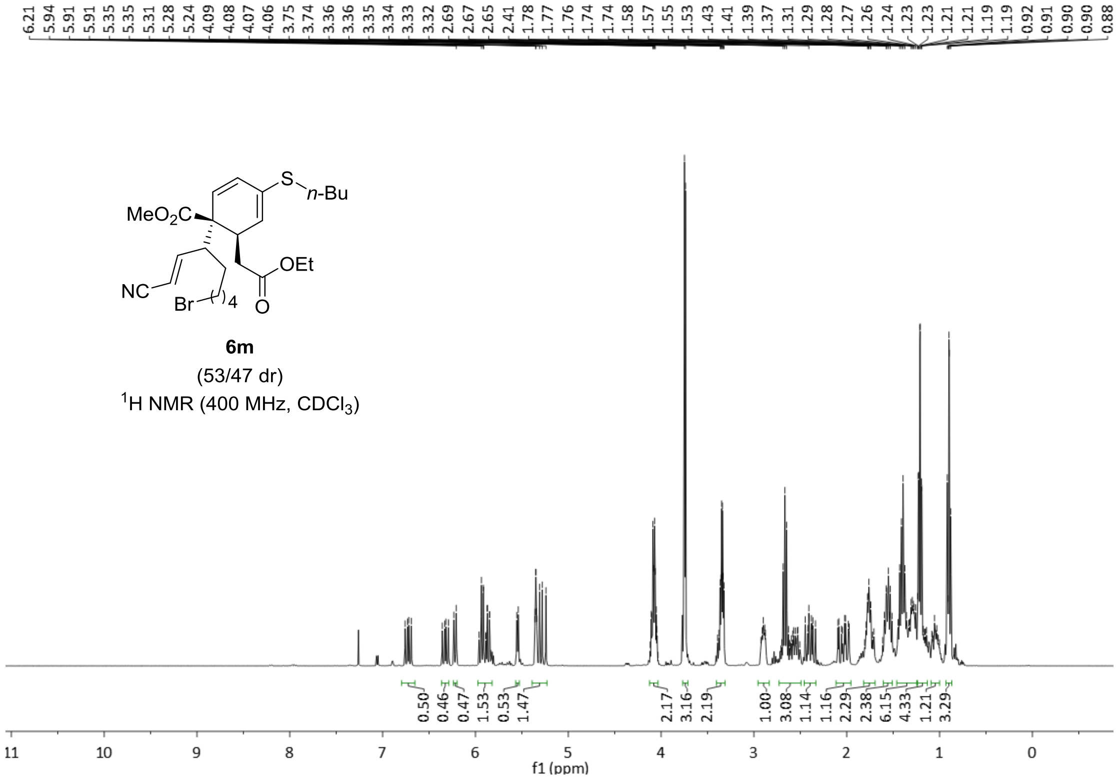

Supplementary Figure 146. ^{1}H NMR spectrum of **6m**

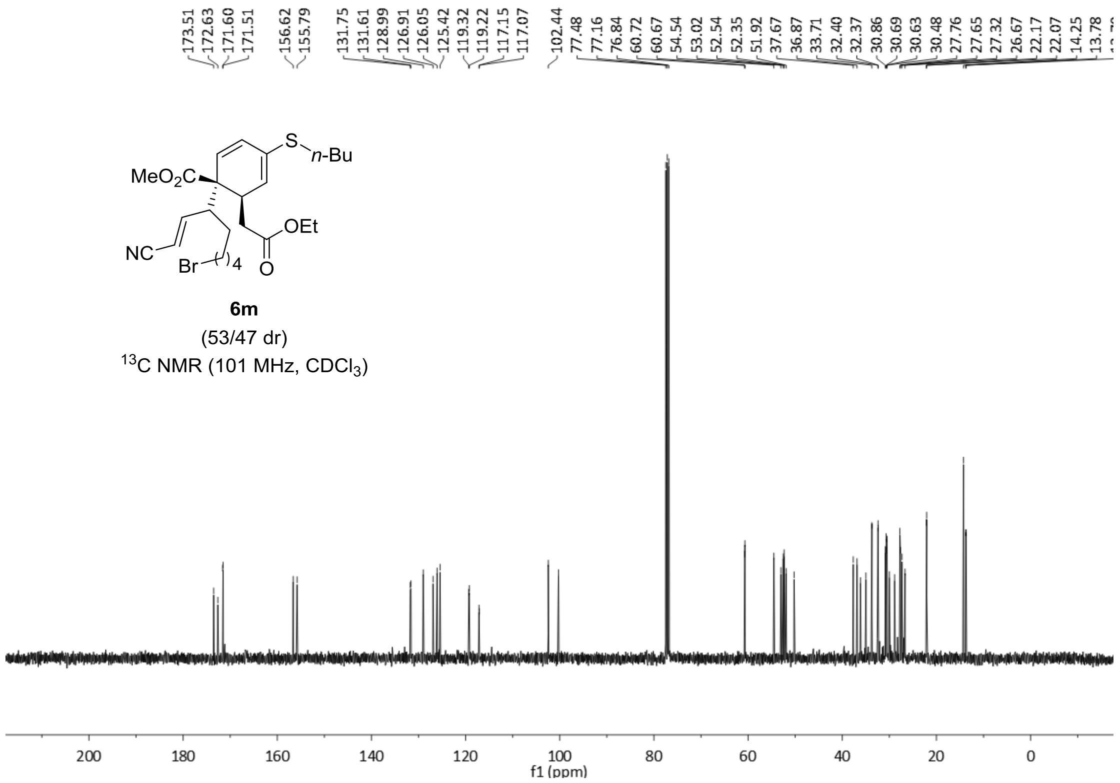

Supplementary Figure 147. ^{13}C NMR spectrum of **6m**

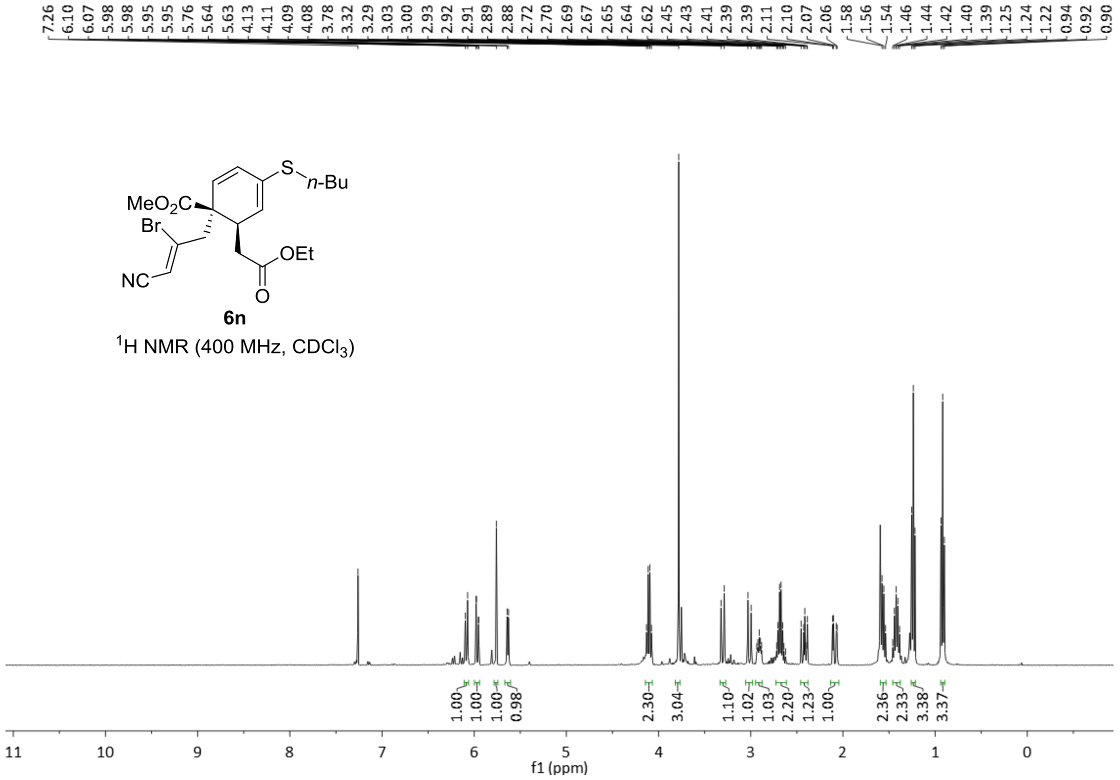

Supplementary Figure 148. ^{1}H NMR spectrum of **6n**

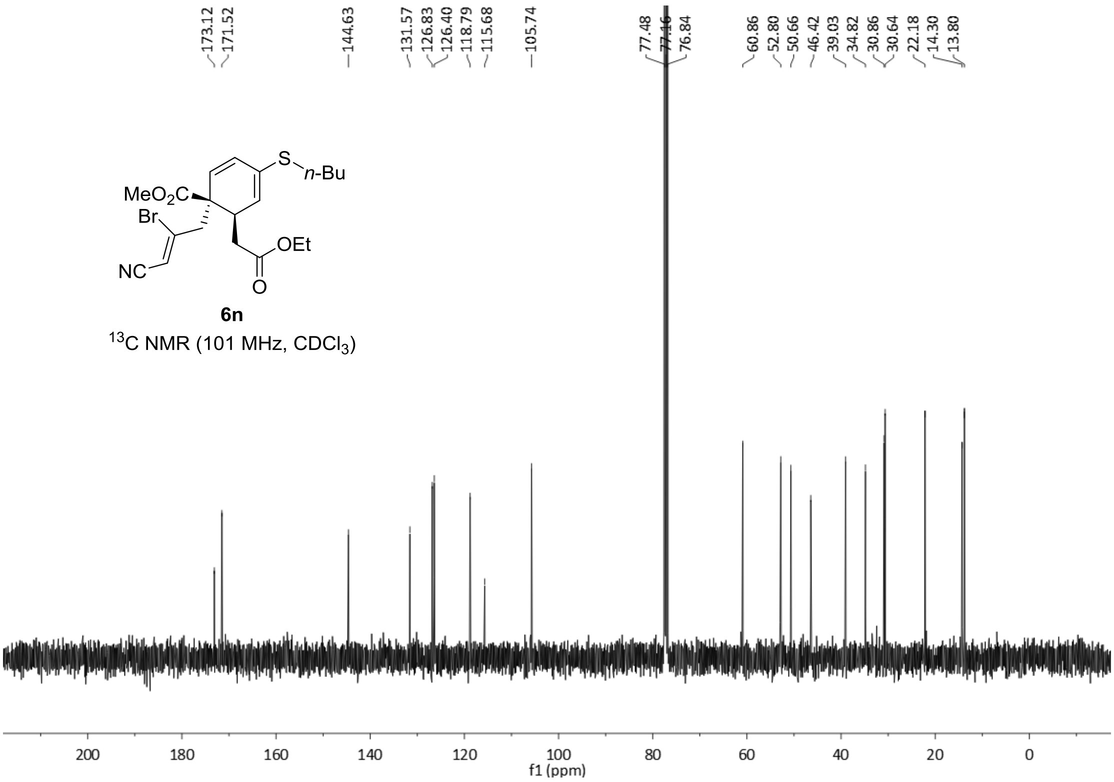

Supplementary Figure 149. ^{13}C NMR spectrum of **6n**

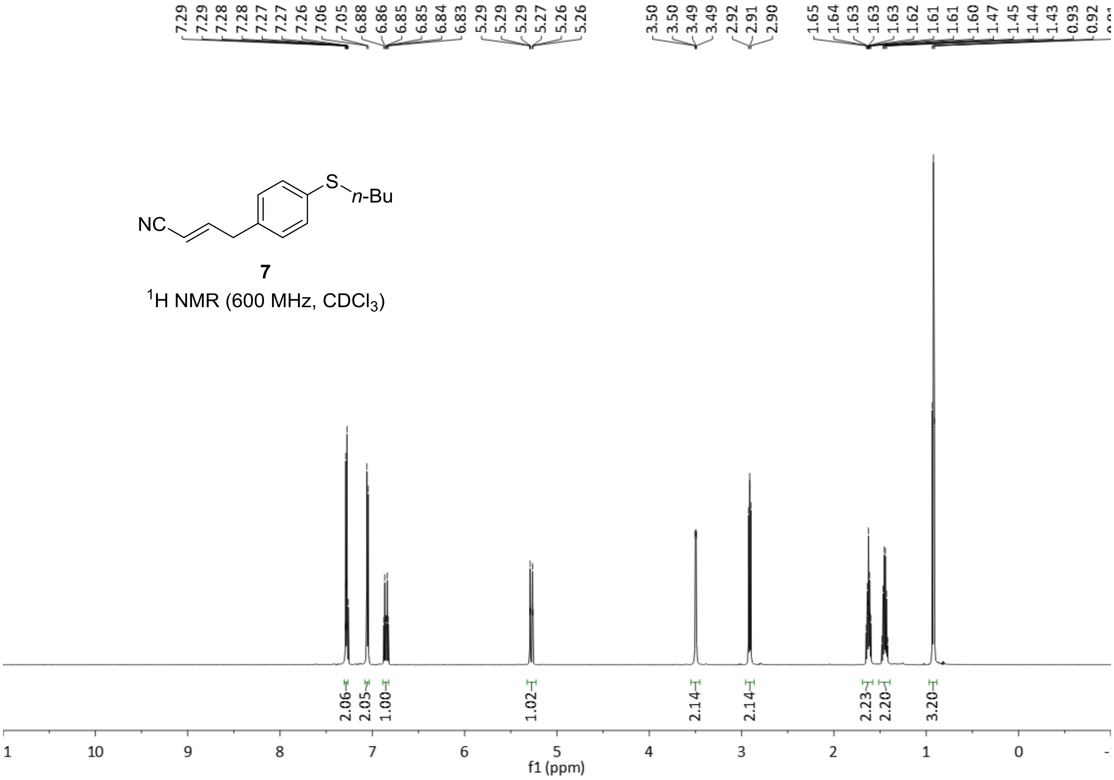

Supplementary Figure 150. ^{1}H NMR spectrum of **7**

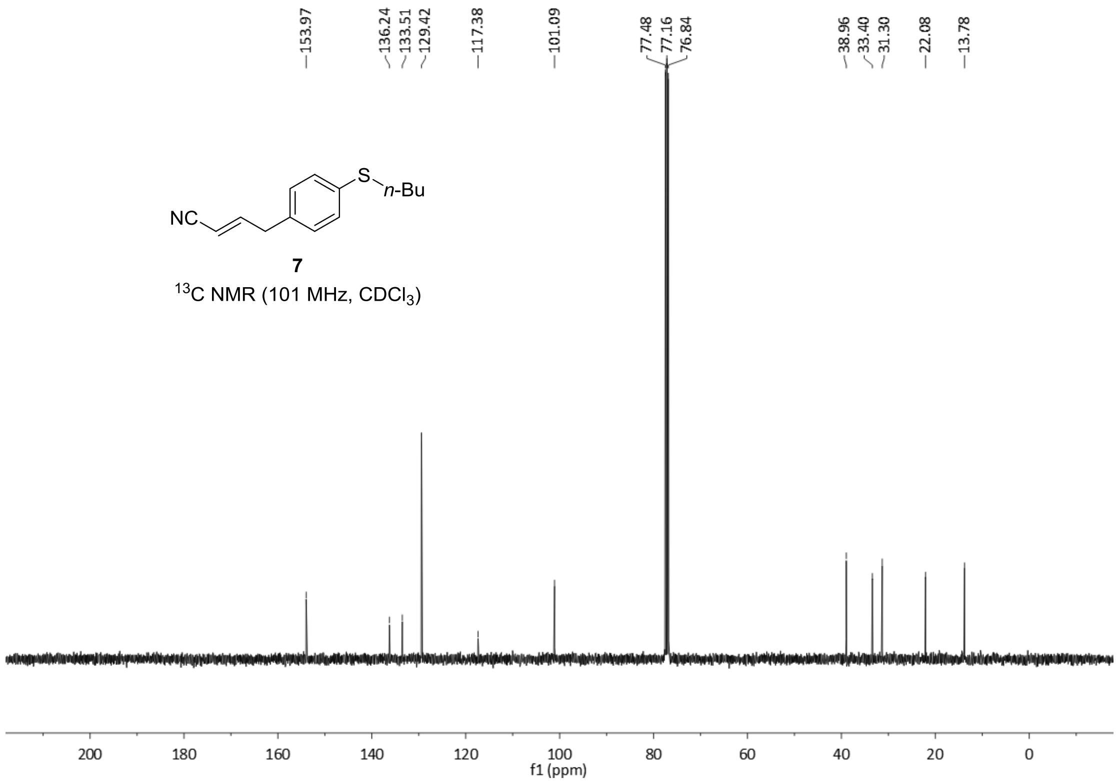

Supplementary Figure 151. ^{13}C NMR spectrum of **7**

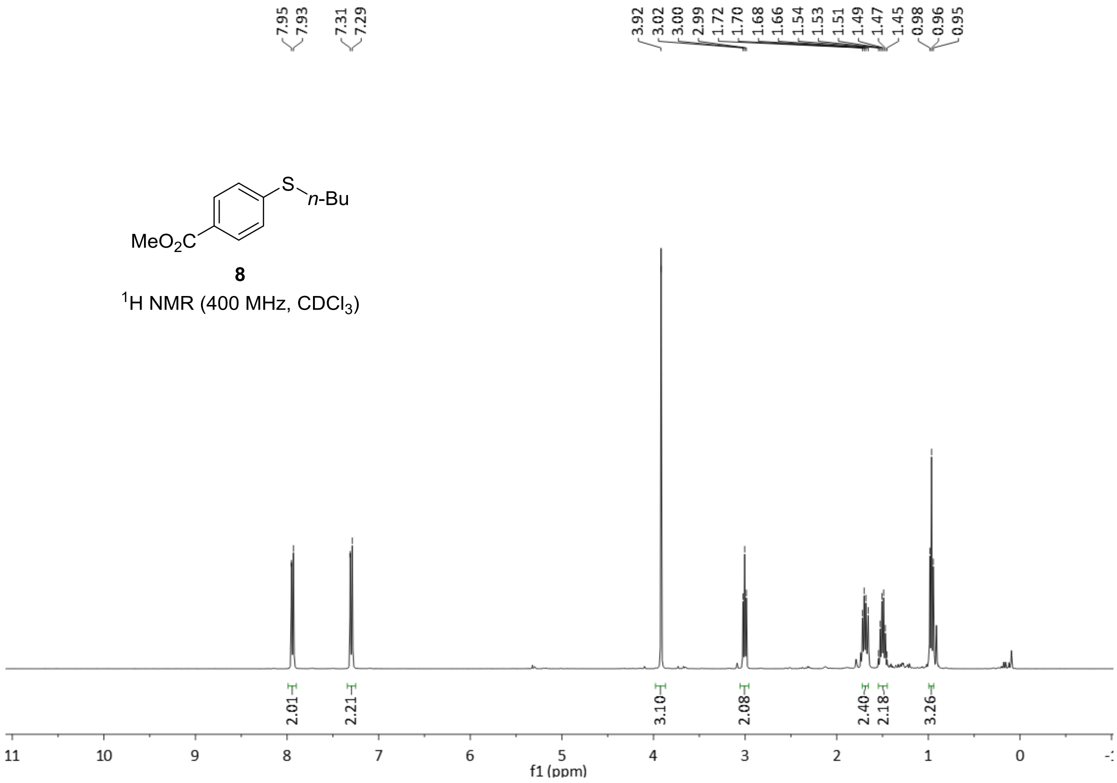

Supplementary Figure 152. ^{1}H NMR spectrum of **8**

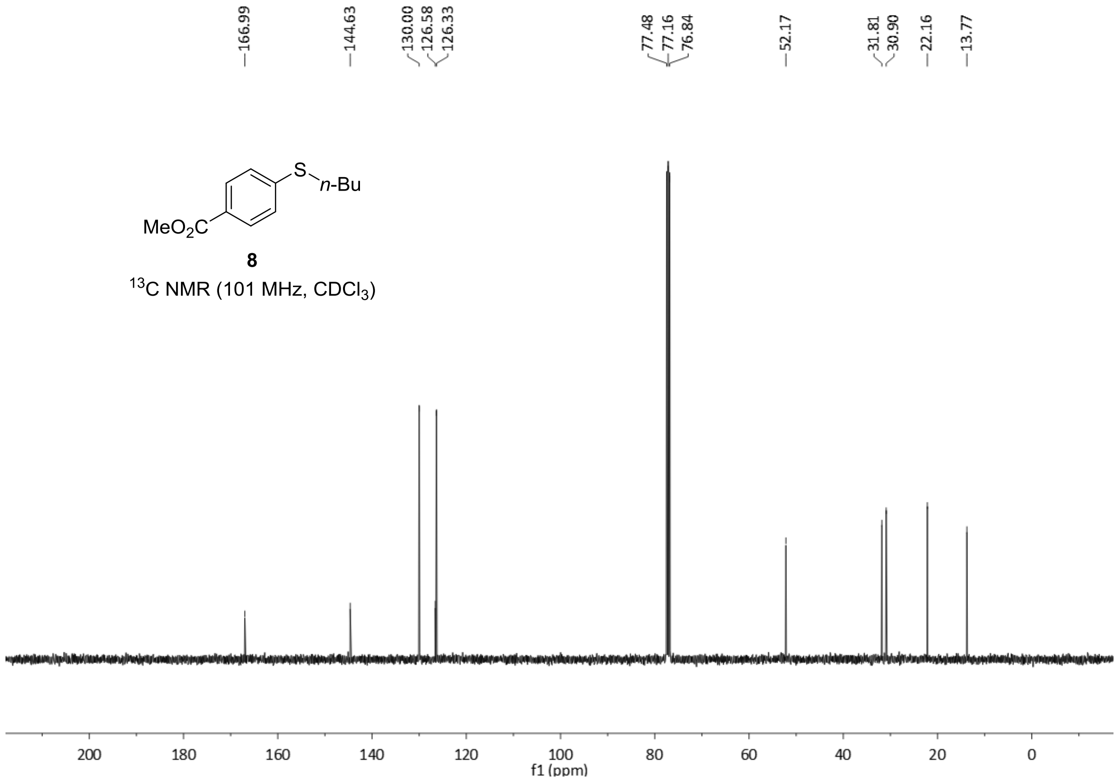

Supplementary Figure 153. ^{13}C NMR spectrum of **8**

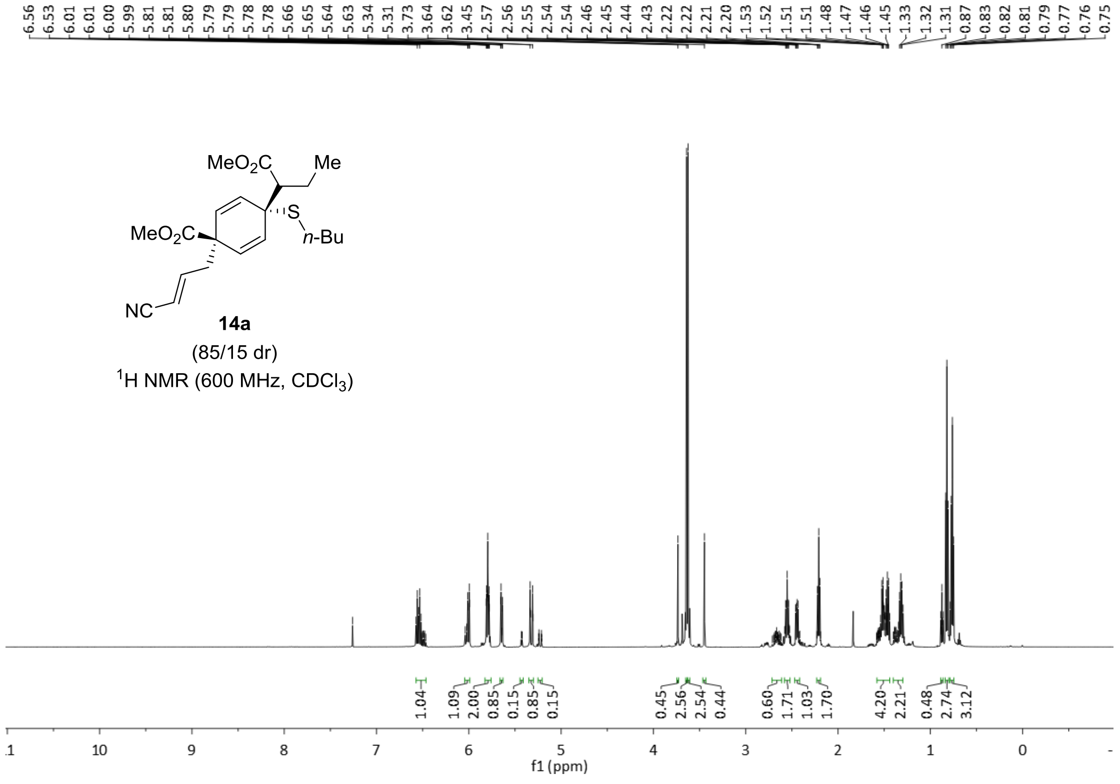

Supplementary Figure 154. ^{1}H NMR spectrum of **14a**

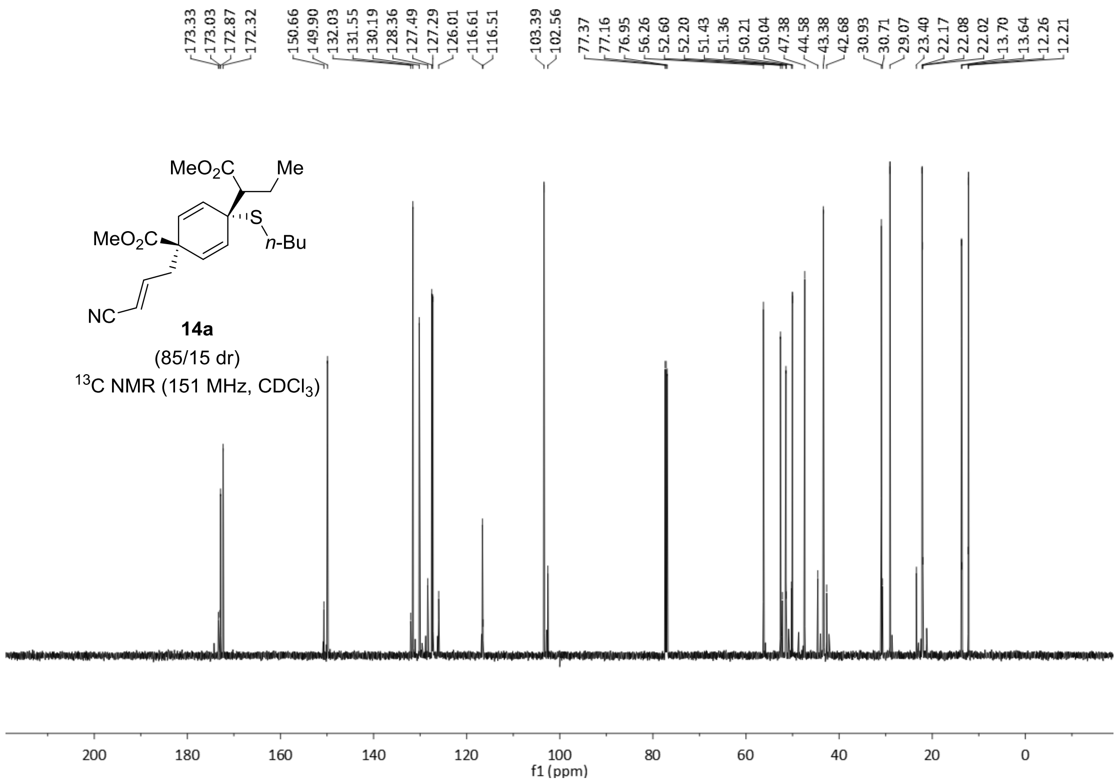

Supplementary Figure 155. ^{13}C NMR spectrum of **14a**

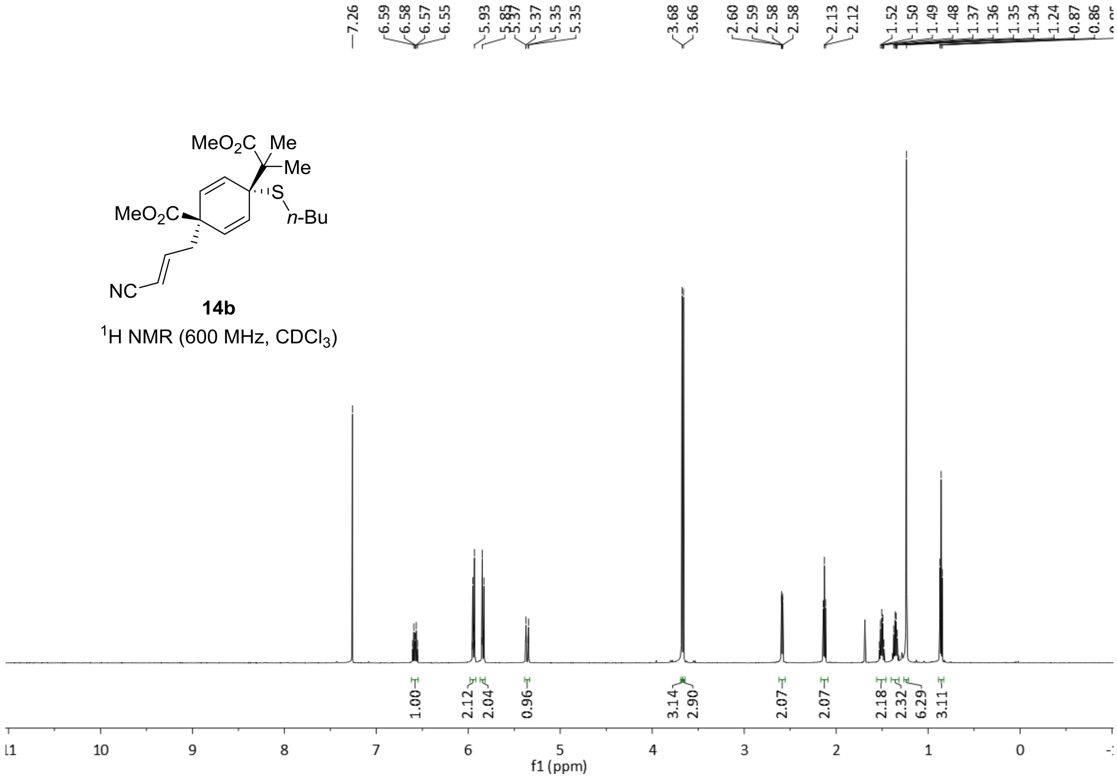

Supplementary Figure 156. ^{1}H NMR spectrum of **14b**

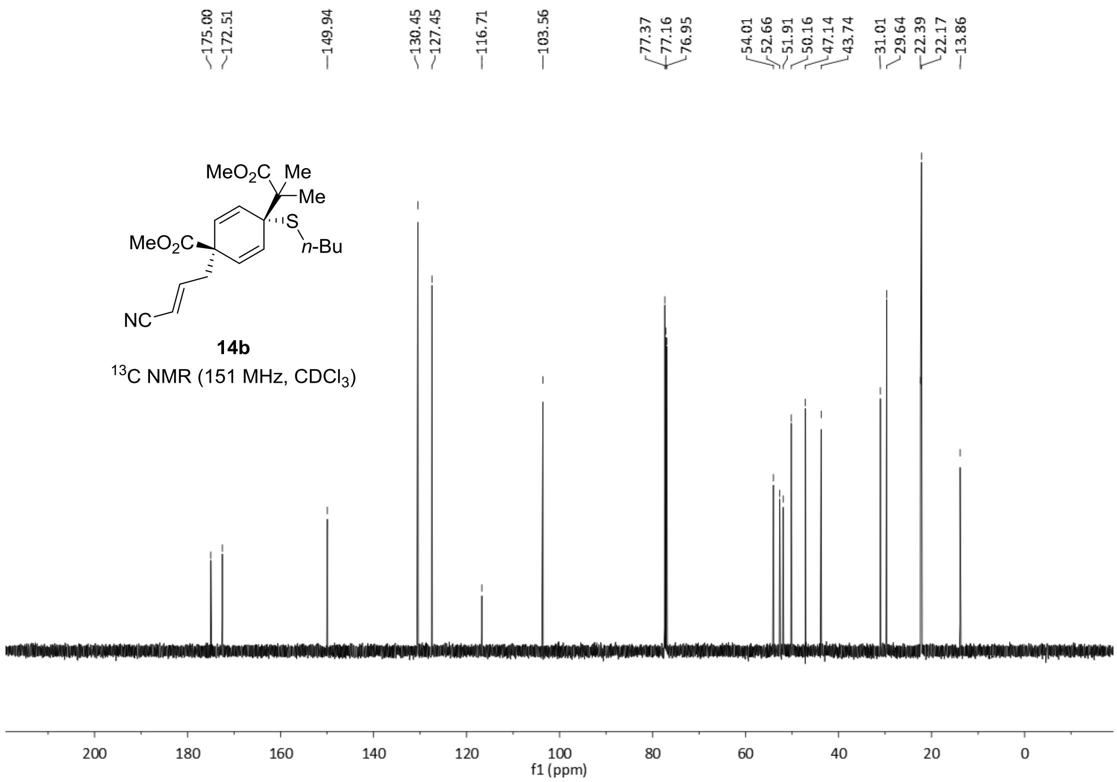

Supplementary Figure 157. ^{13}C NMR spectrum of **14b**

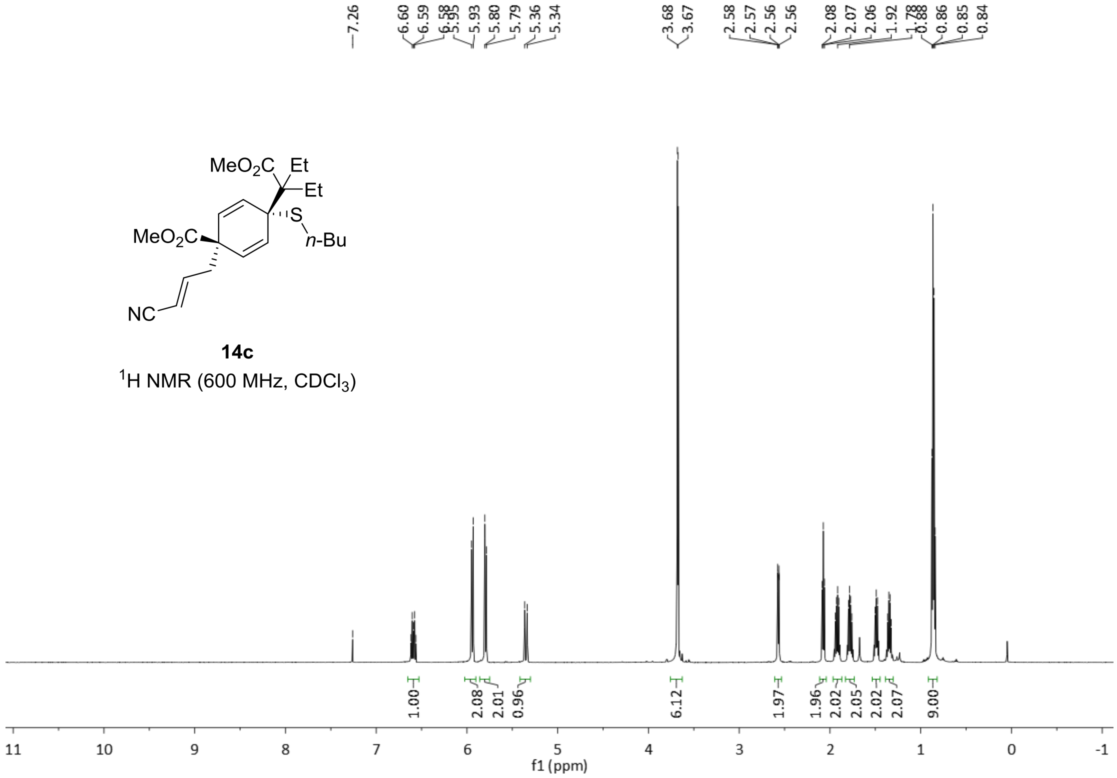

Supplementary Figure 158. ^{1}H NMR spectrum of **14c**

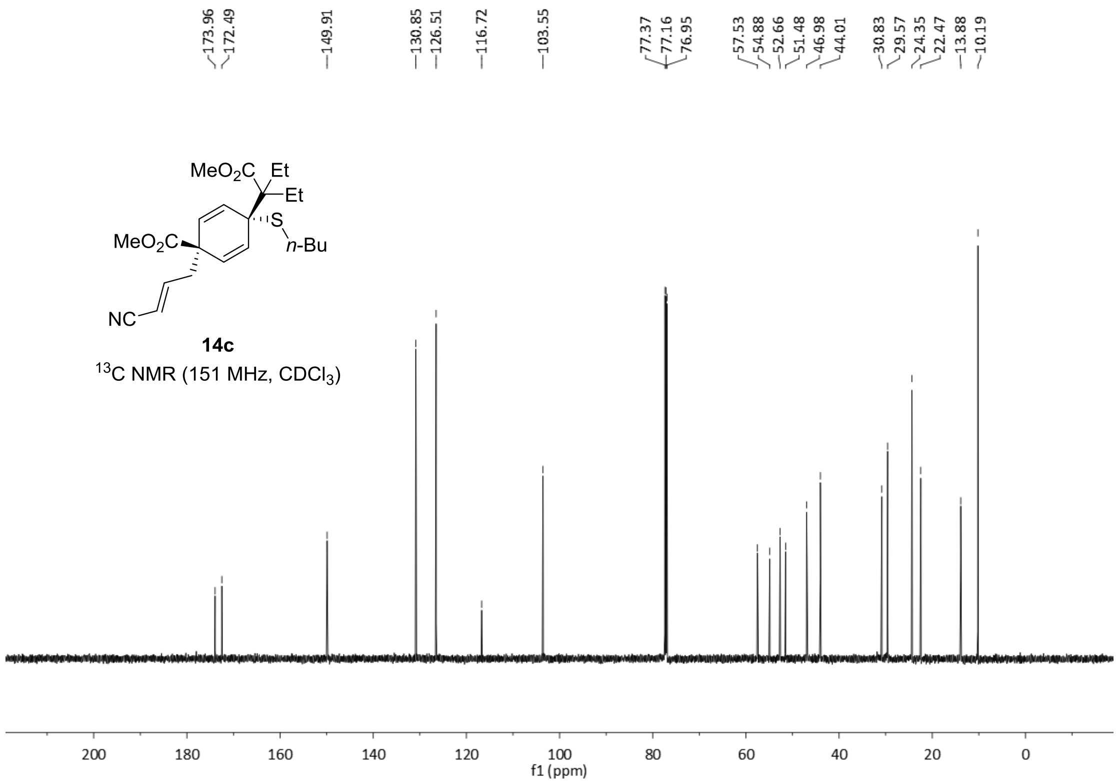

Supplementary Figure 159. ^{13}C NMR spectrum of **14c**

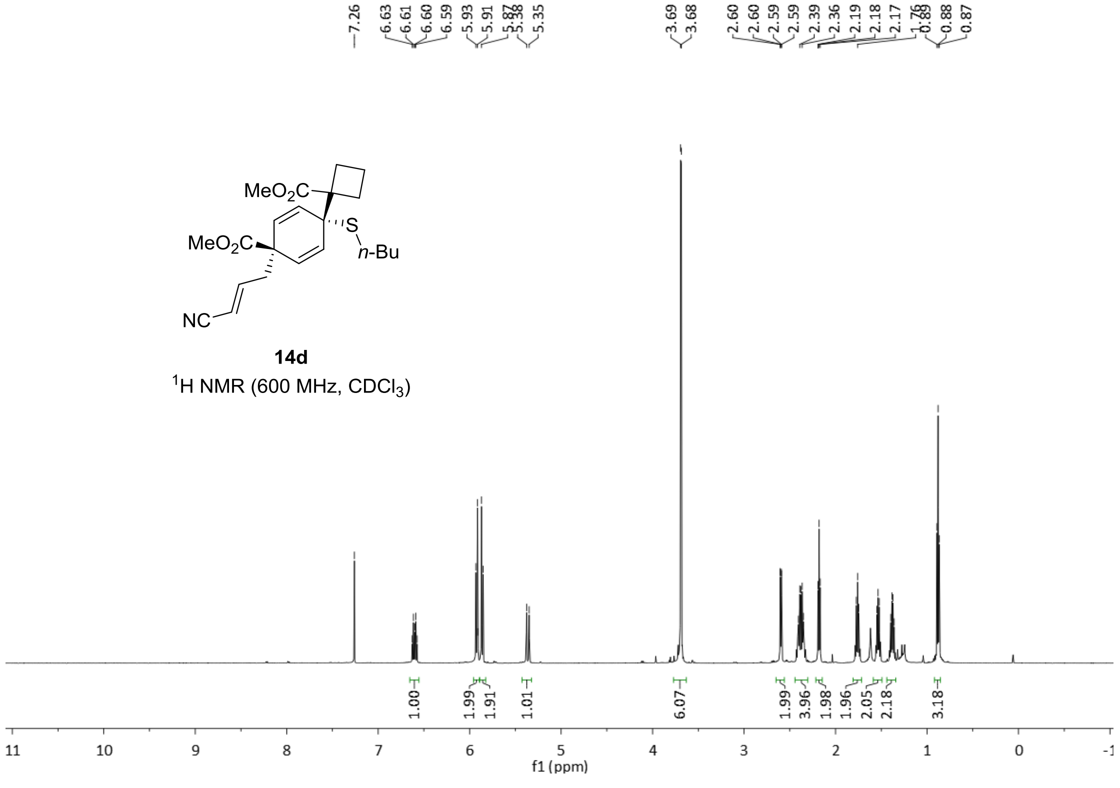

Supplementary Figure 160. ^{1}H NMR spectrum of **14d**

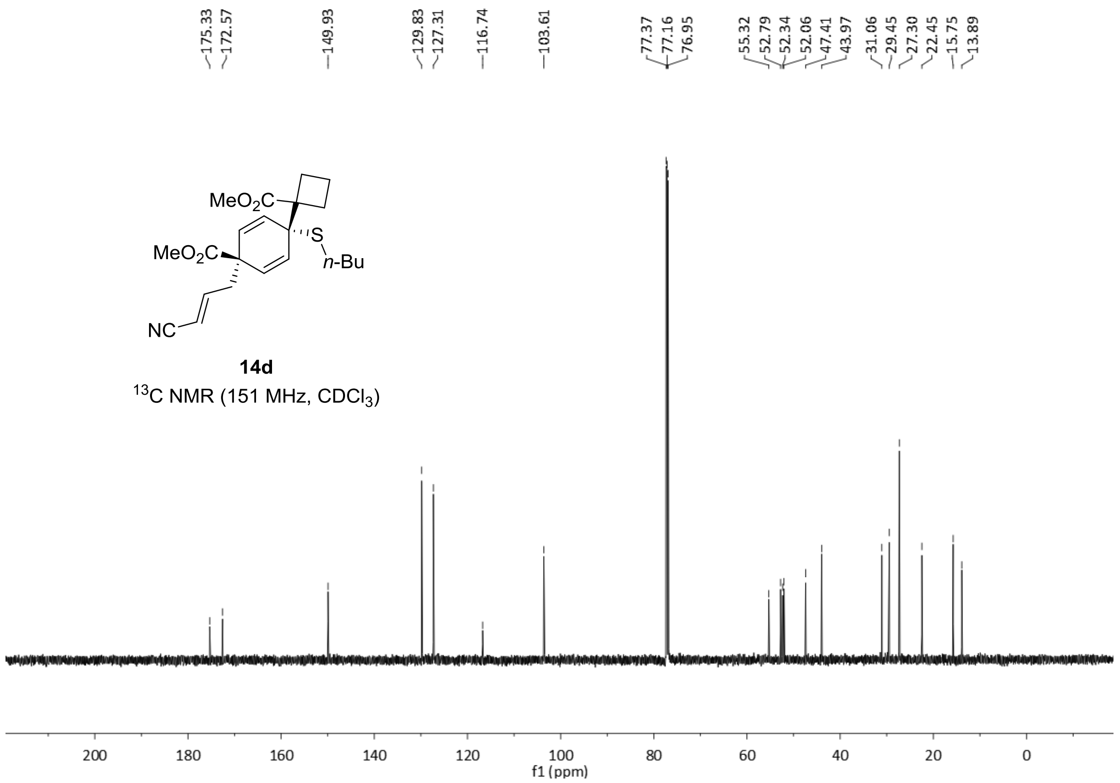

Supplementary Figure 161. ^{13}C NMR spectrum of **14d**

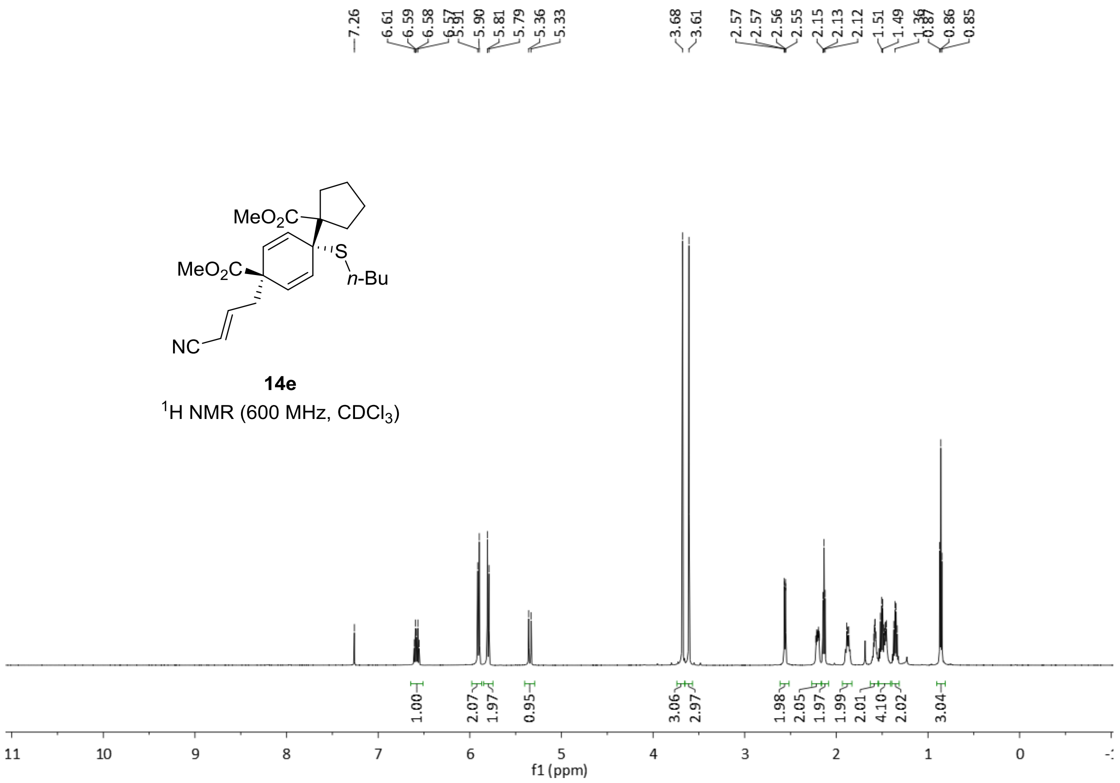

Supplementary Figure 162. ^{1}H NMR spectrum of **14e**

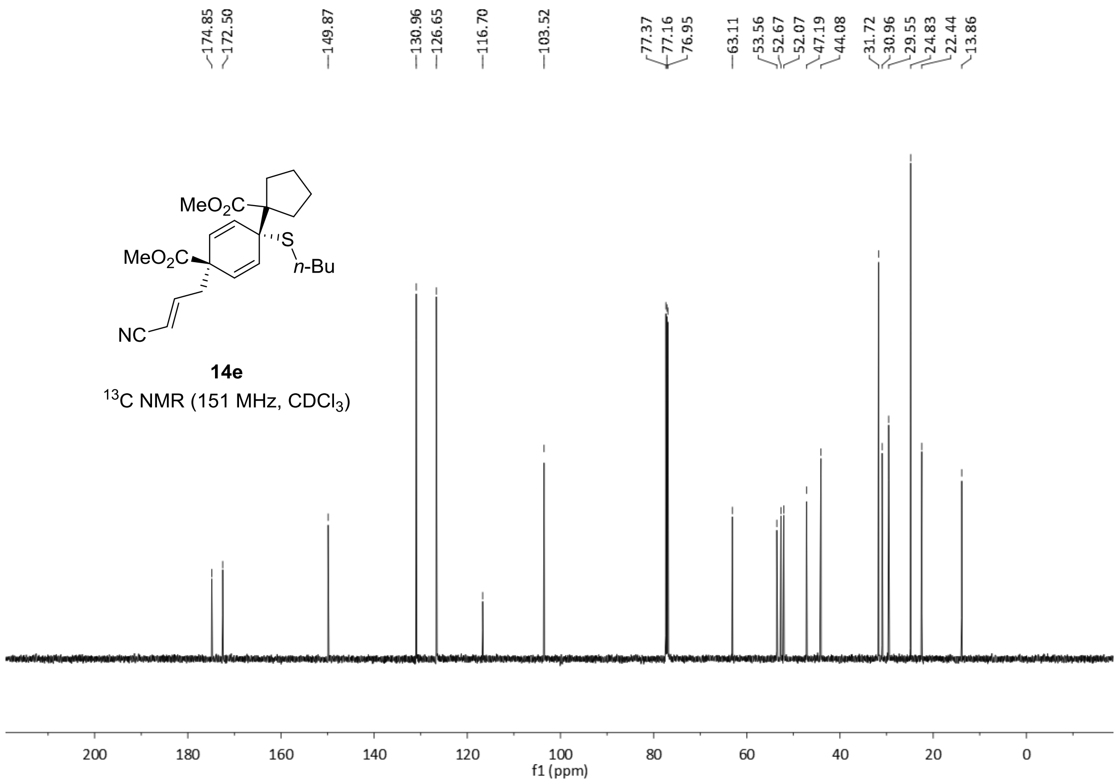

Supplementary Figure 163. ^{13}C NMR spectrum of **14e**

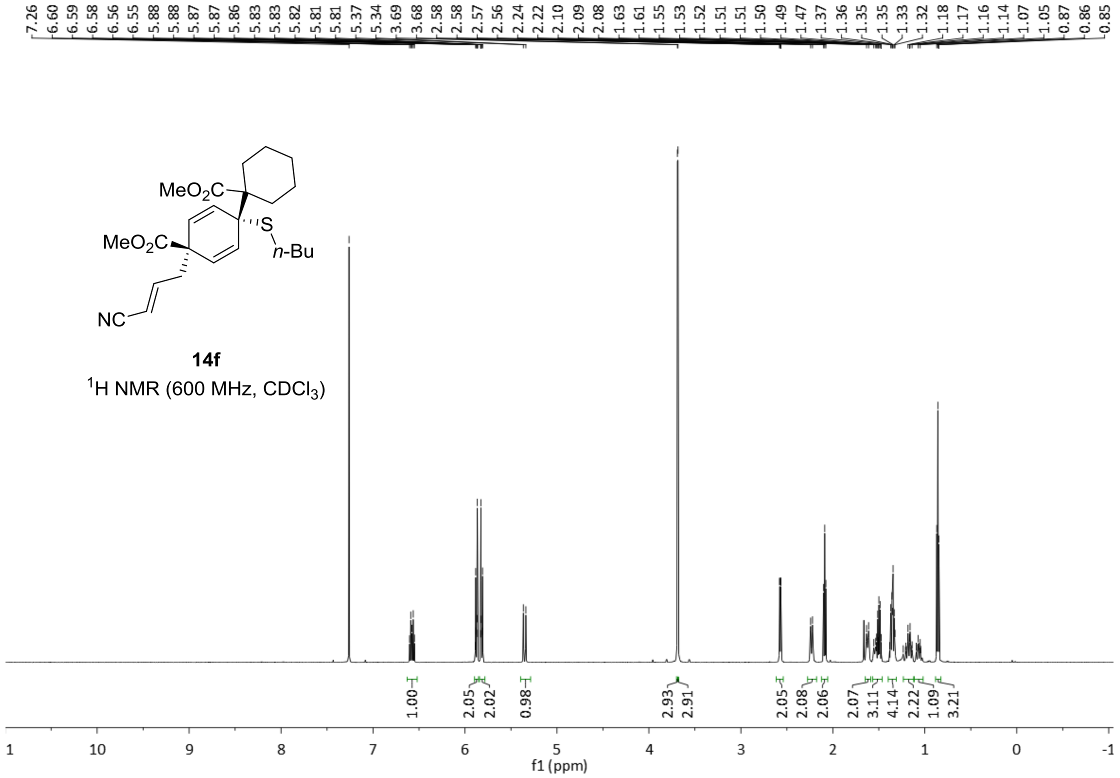

Supplementary Figure 164. ^{1}H NMR spectrum of **14f**

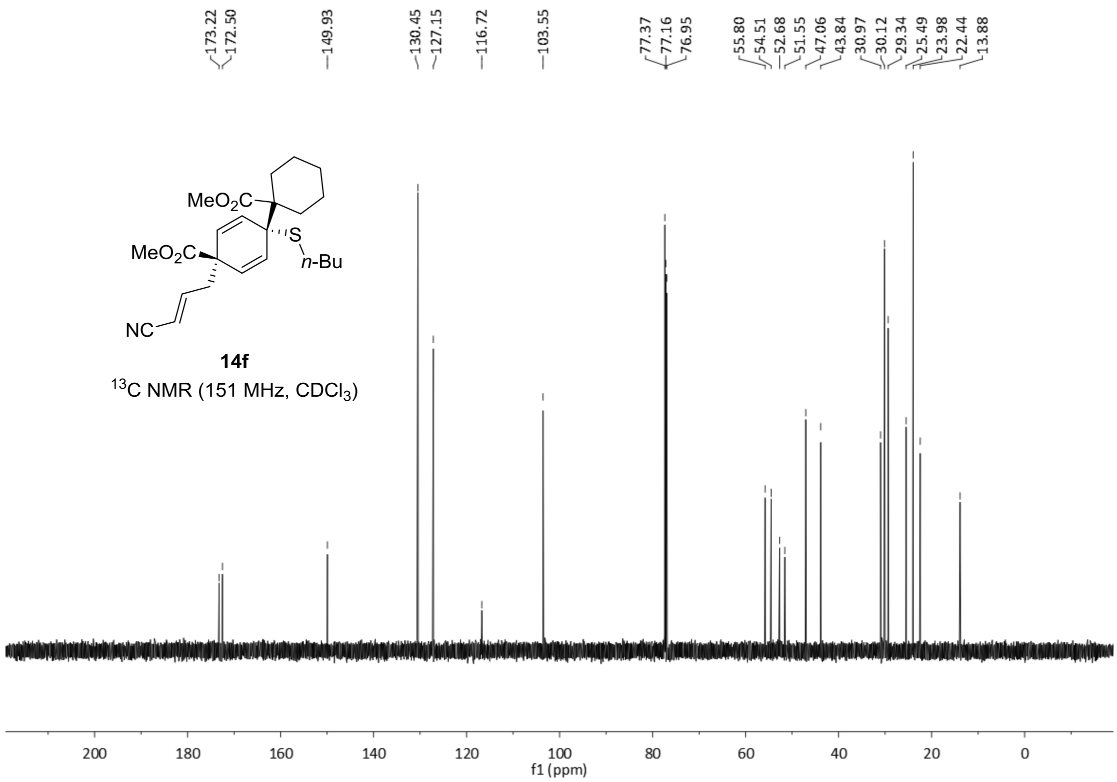

Supplementary Figure 165. ^{13}C NMR spectrum of **14f**

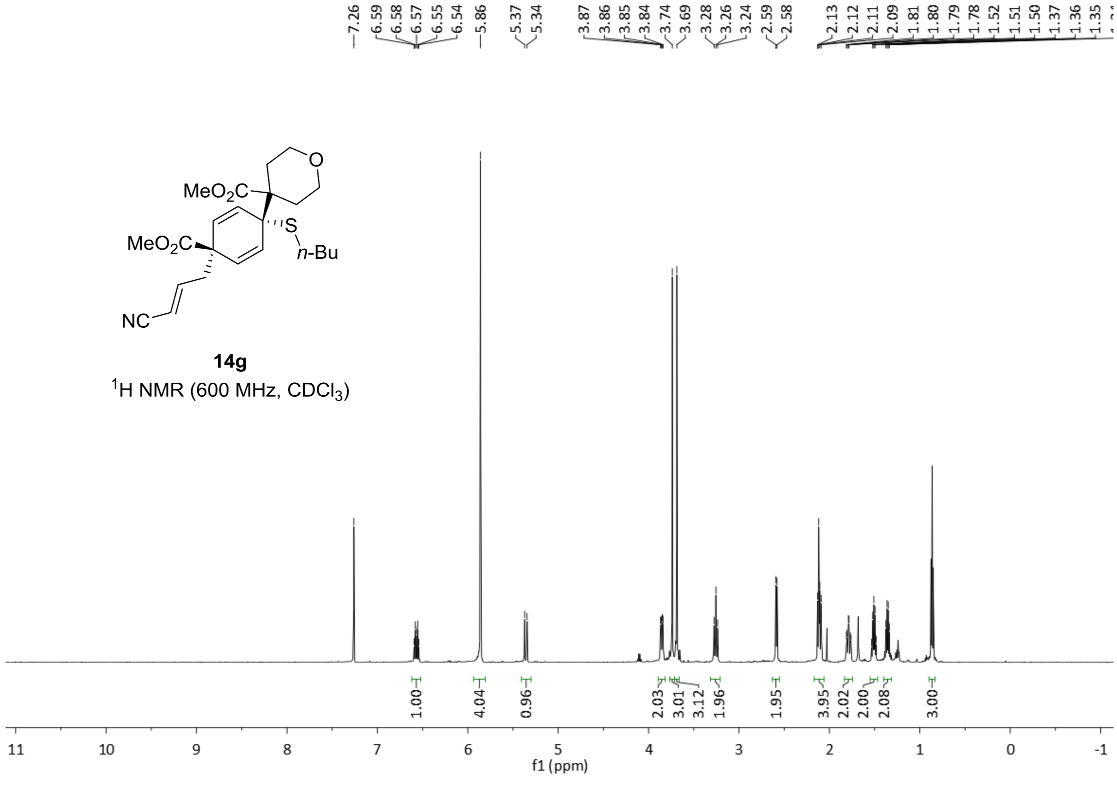

Supplementary Figure 166. ^{1}H NMR spectrum of **14g**

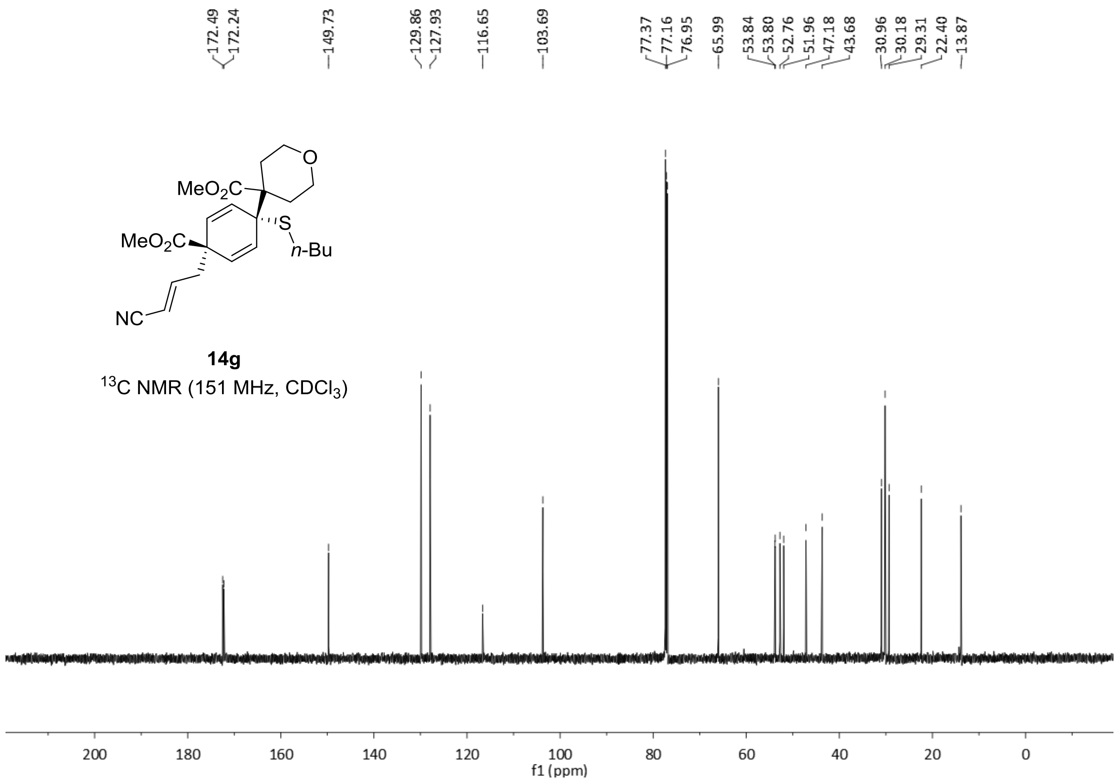

Supplementary Figure 167. ^{13}C NMR spectrum of **14g**

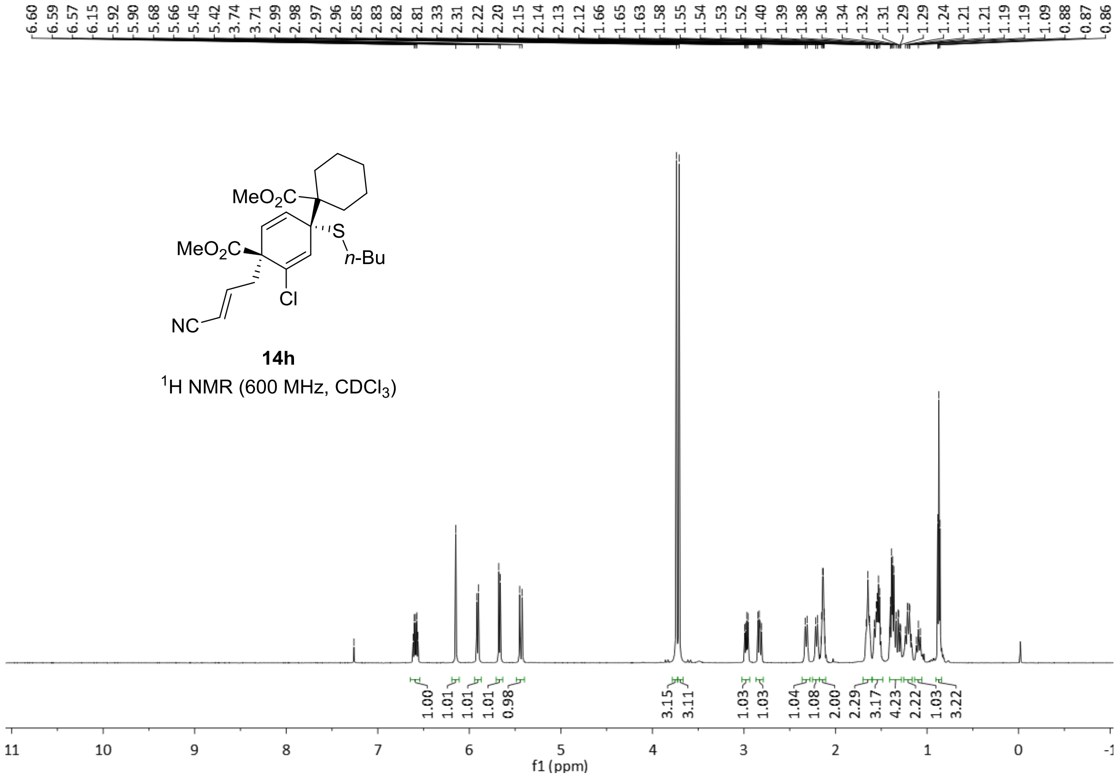

Supplementary Figure 168. ^{1}H NMR spectrum of **14h**

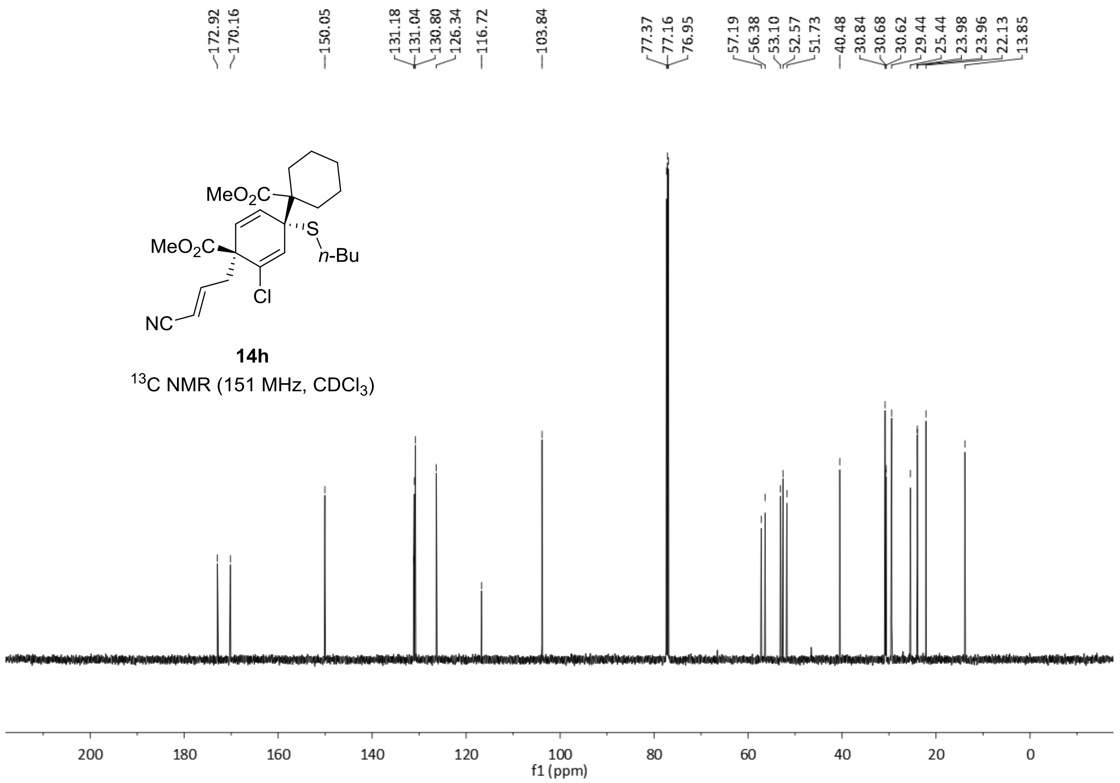

Supplementary Figure 169. ^{13}C NMR spectrum of **14h**

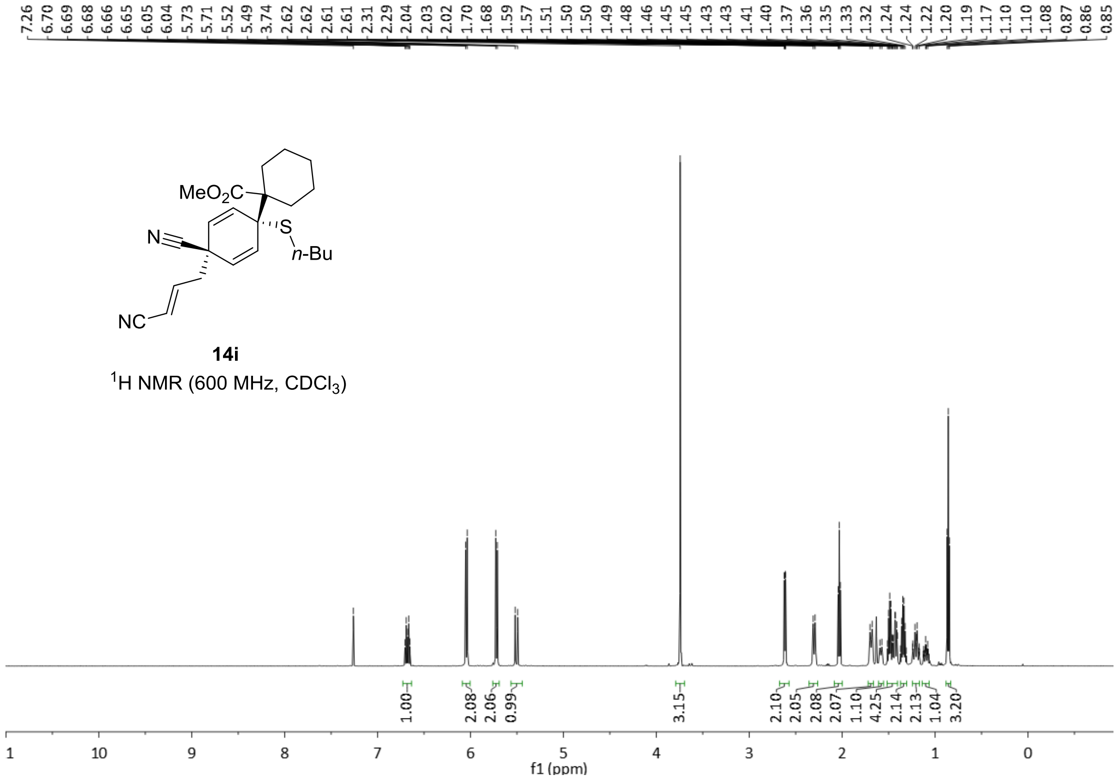

Supplementary Figure 170. ^{1}H NMR spectrum of **14i**

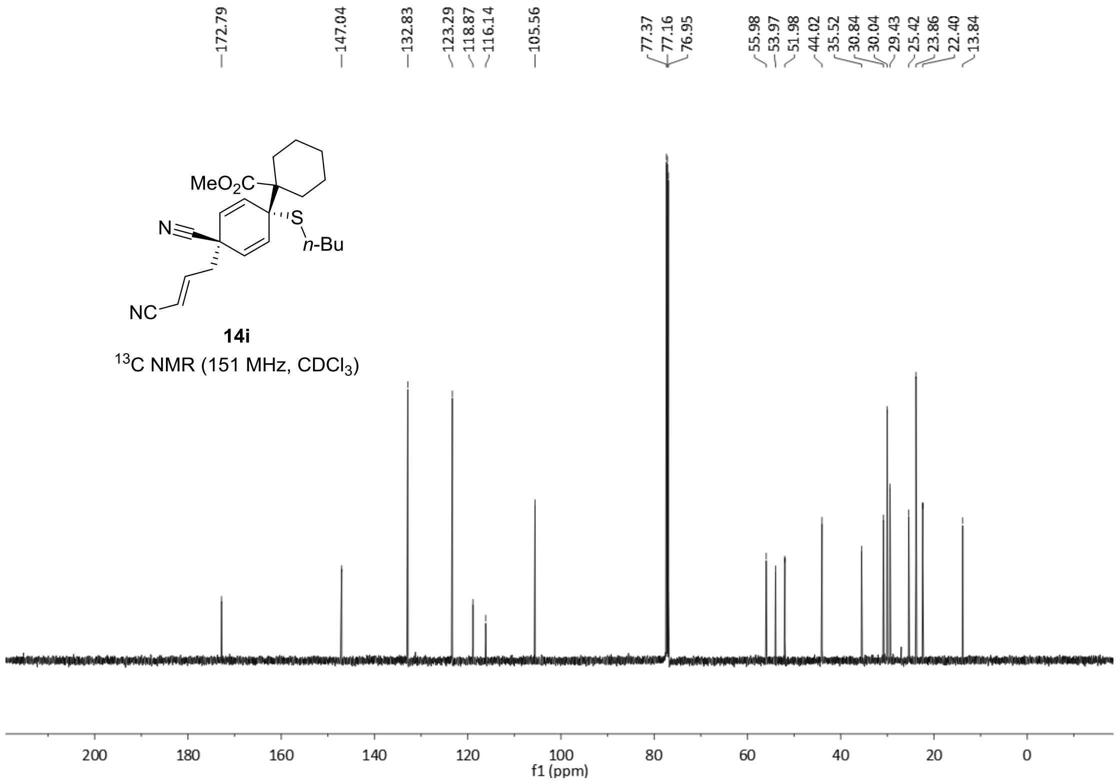

Supplementary Figure 171. ^{13}C NMR spectrum of **14i**

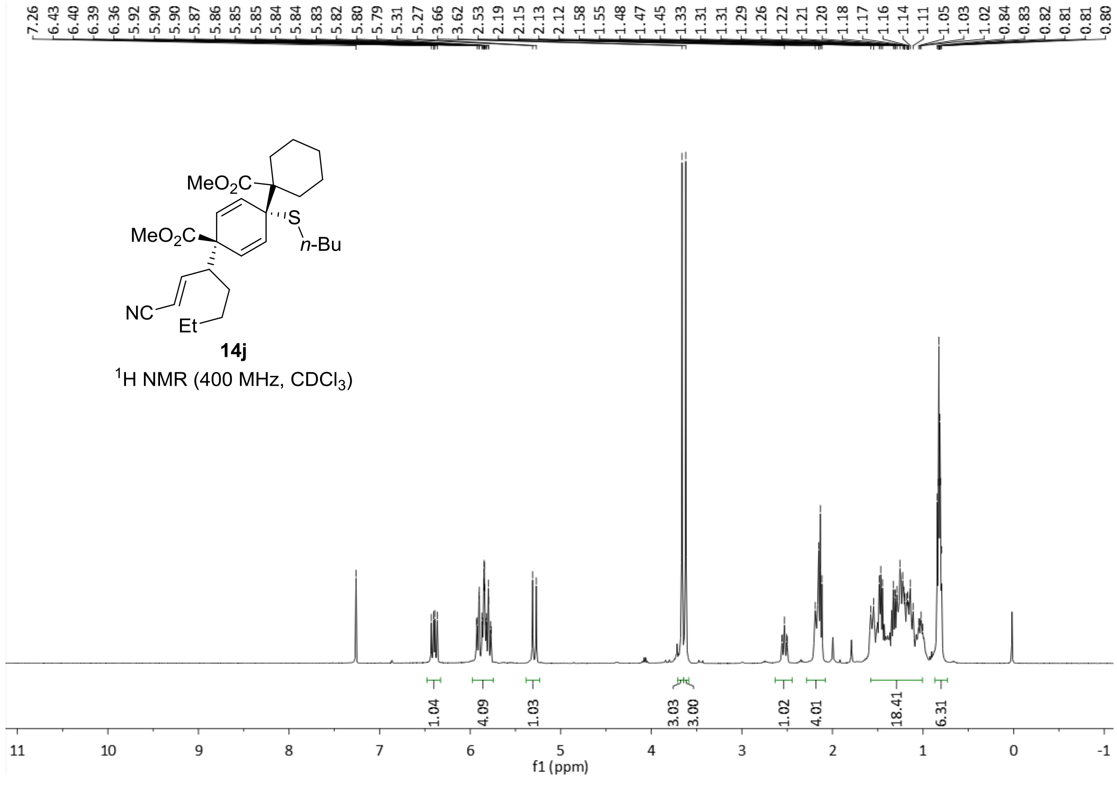

Supplementary Figure 172. ^{1}H NMR spectrum of **14j**

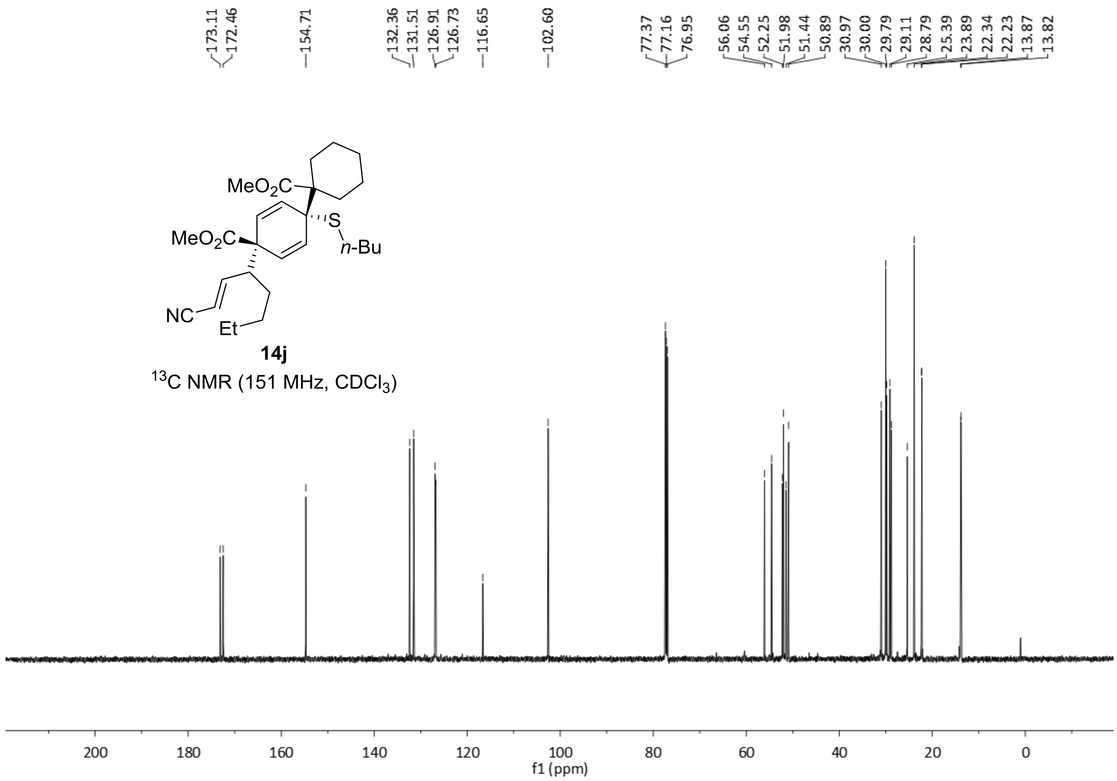

Supplementary Figure 173. ^{13}C NMR spectrum of **14j**

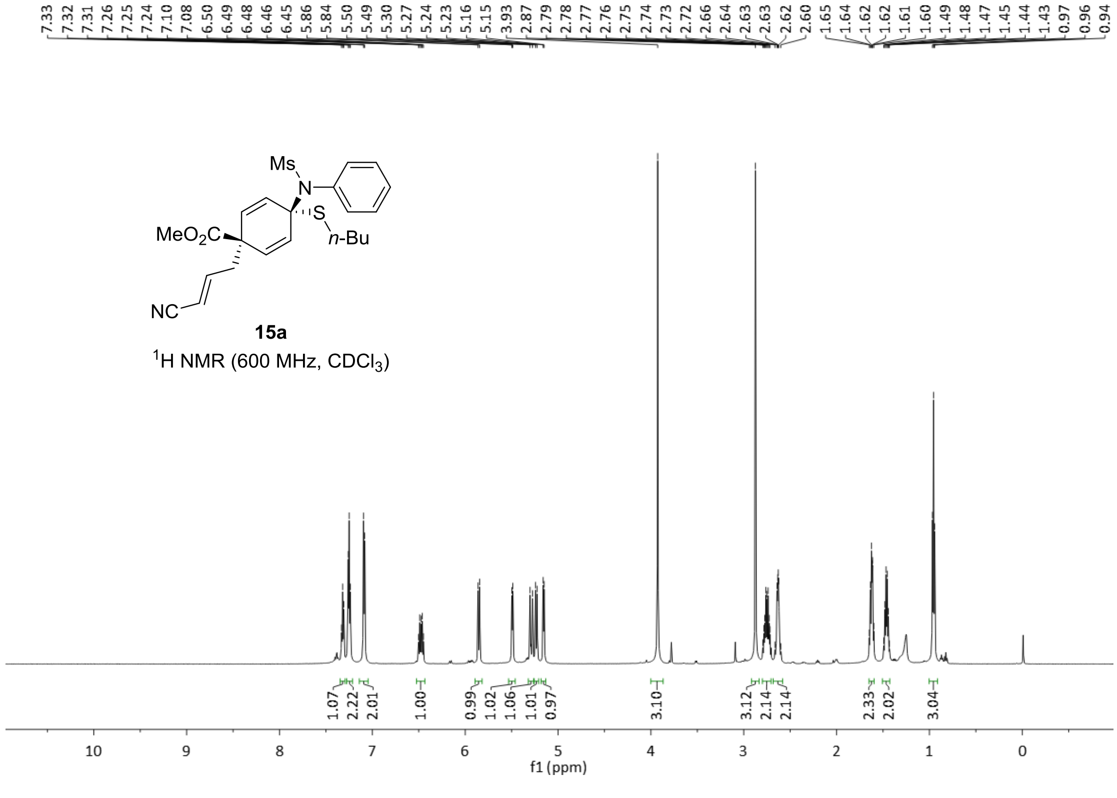

Supplementary Figure 174. ^{1}H NMR spectrum of **15a**

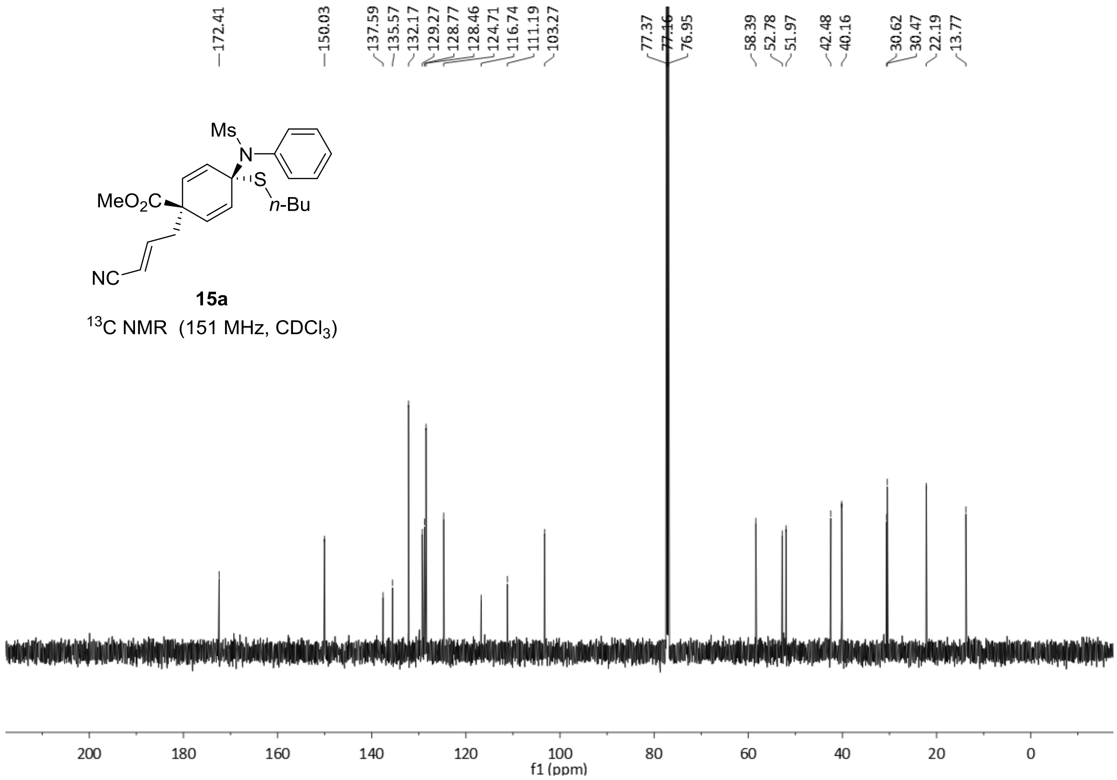

Supplementary Figure 175. ^{13}C NMR spectrum of **15a**

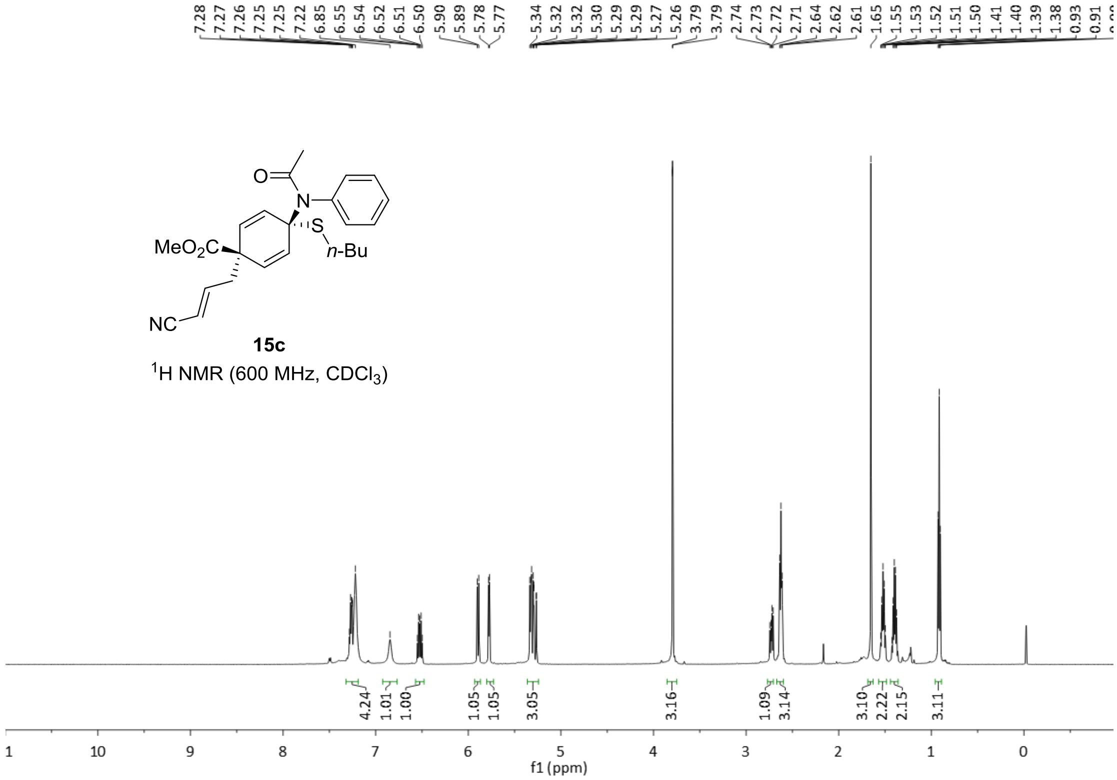

Supplementary Figure 176. ^{1}H NMR spectrum of **15c**

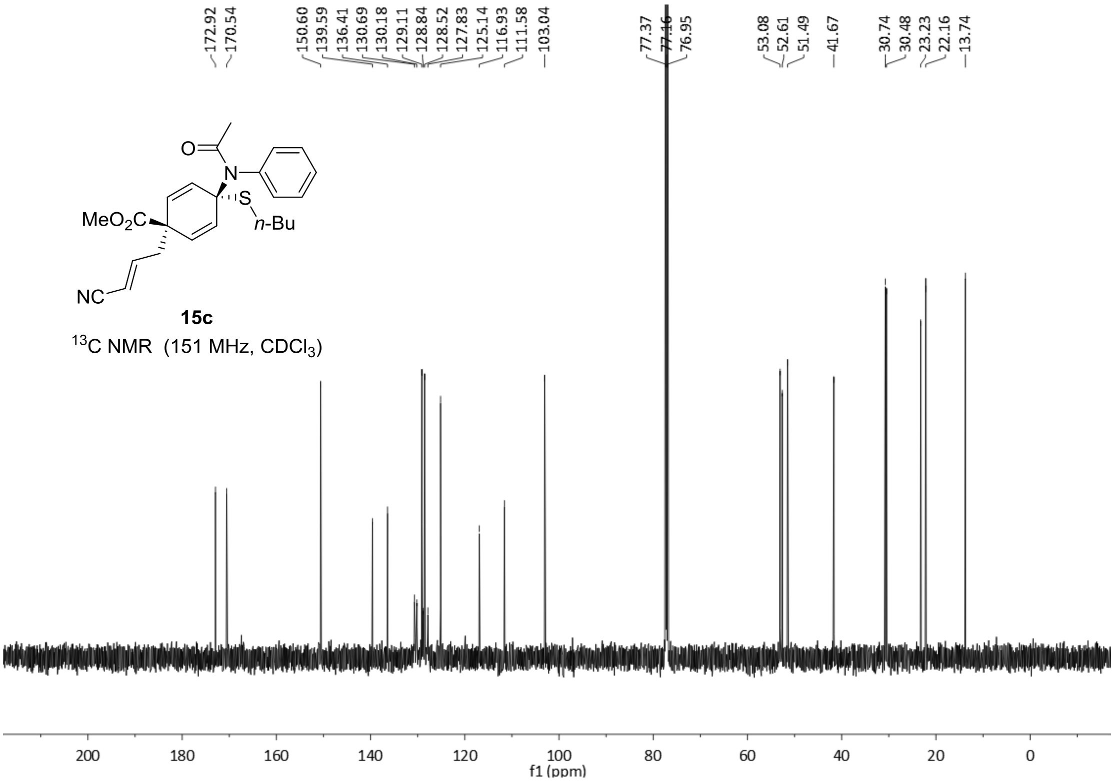

Supplementary Figure 177. ^{13}C NMR spectrum of **15c**

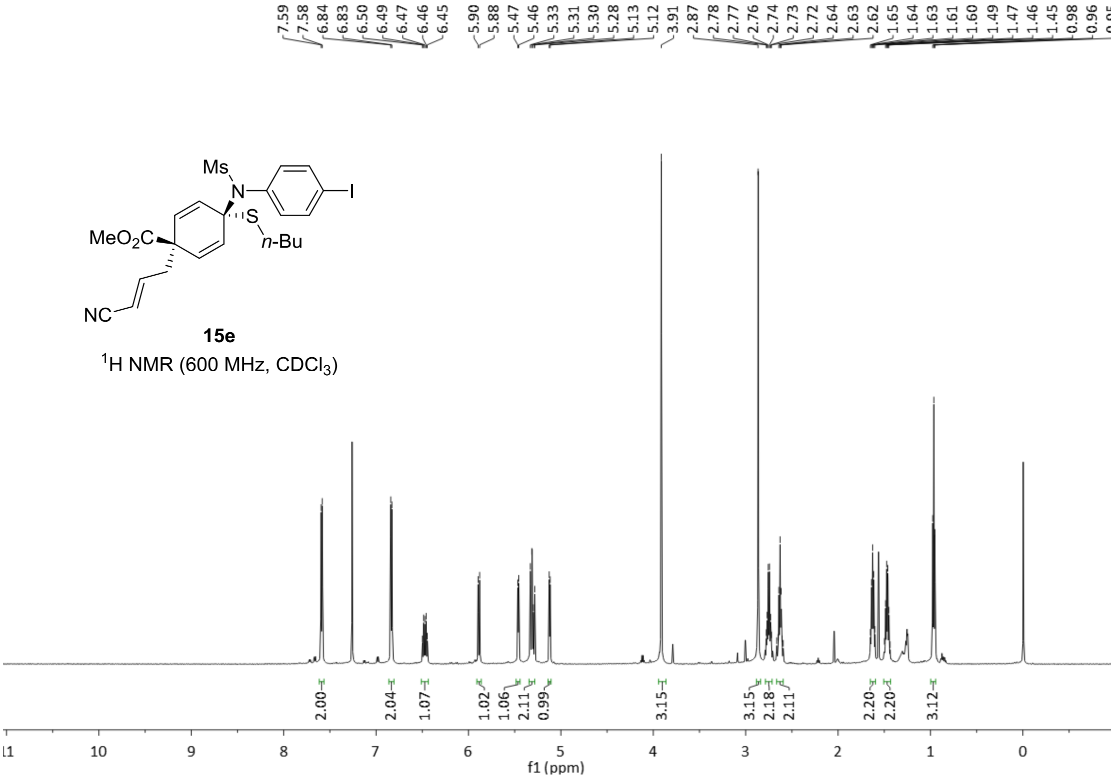

Supplementary Figure 178. ^{1}H NMR spectrum of **15e**

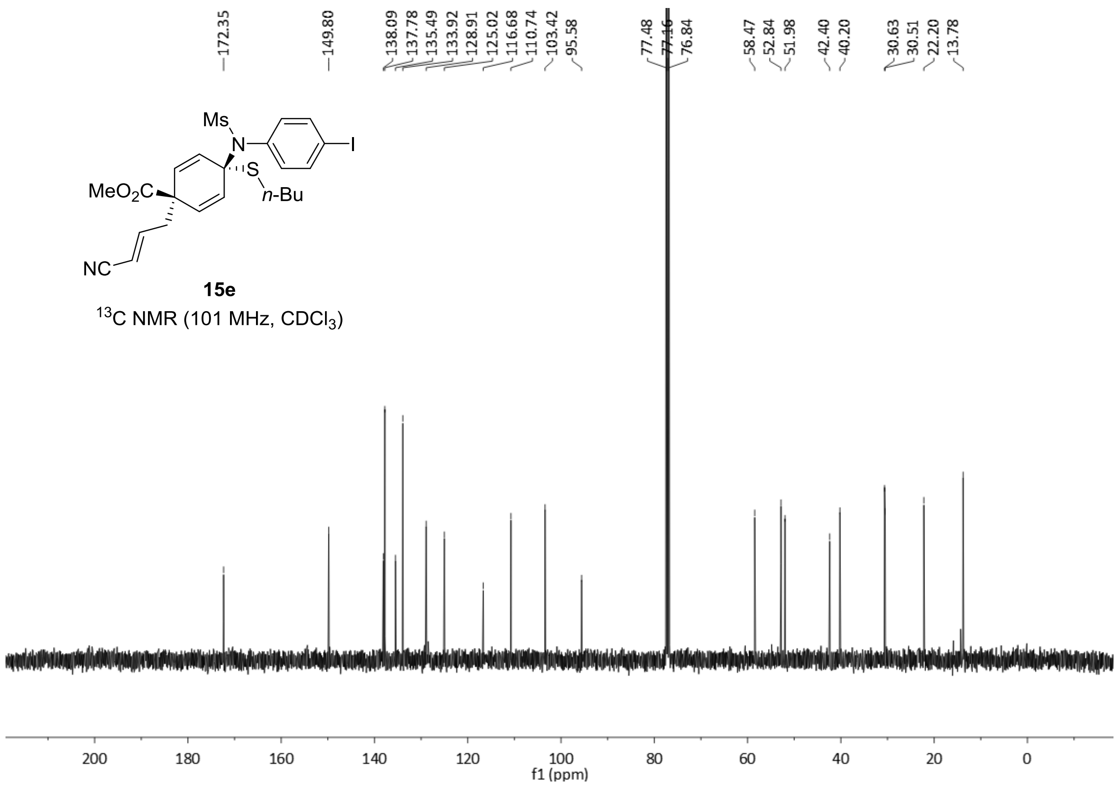

Supplementary Figure 179. ^{13}C NMR spectrum of **15e**

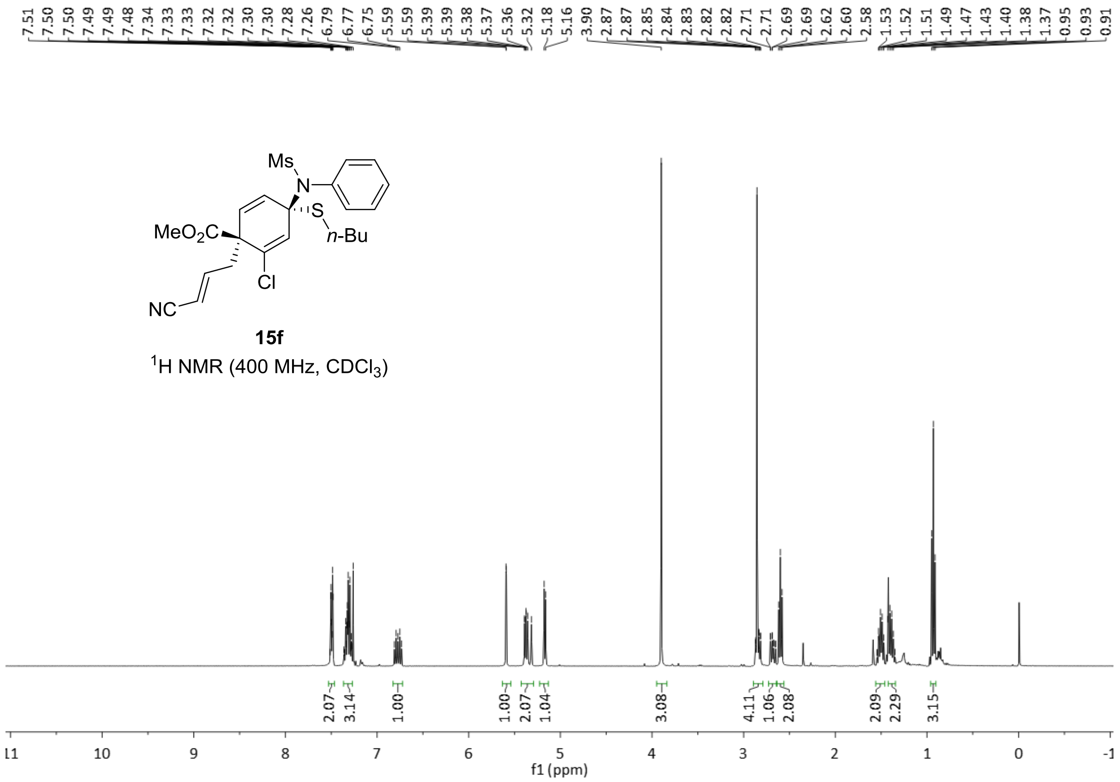

Supplementary Figure 180. ^{1}H NMR spectrum of **15f**

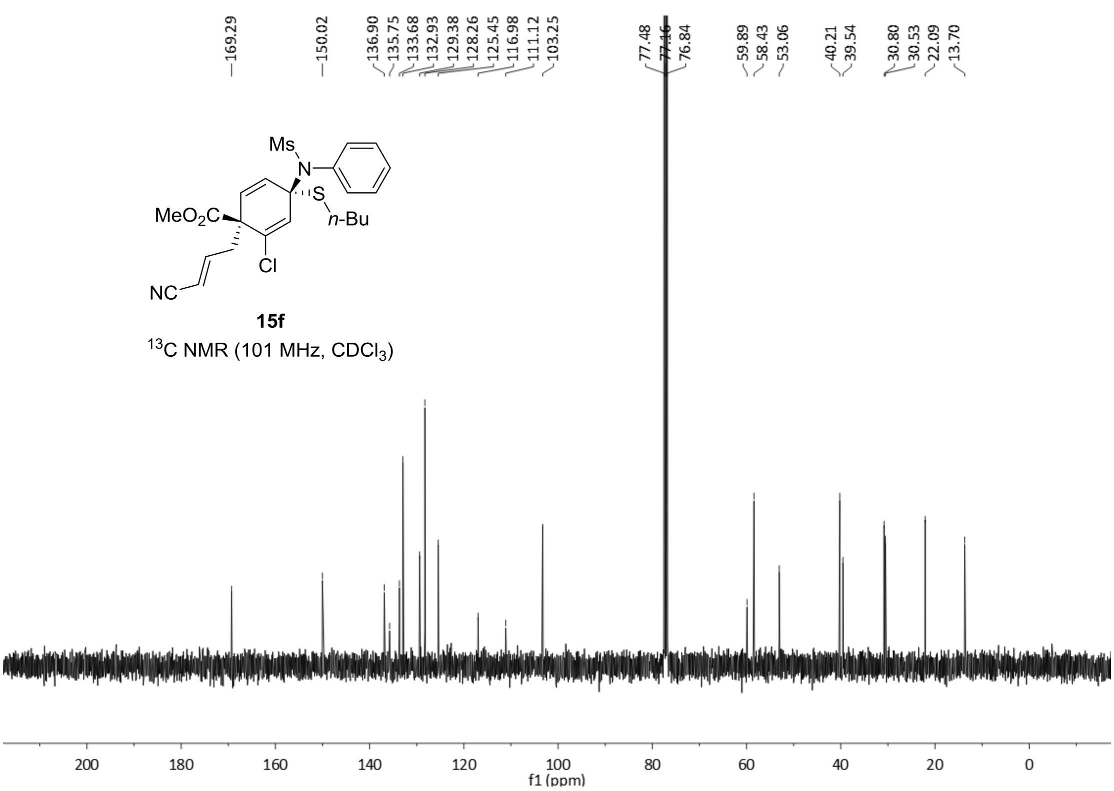

Supplementary Figure 181. ^{13}C NMR spectrum of **15f**

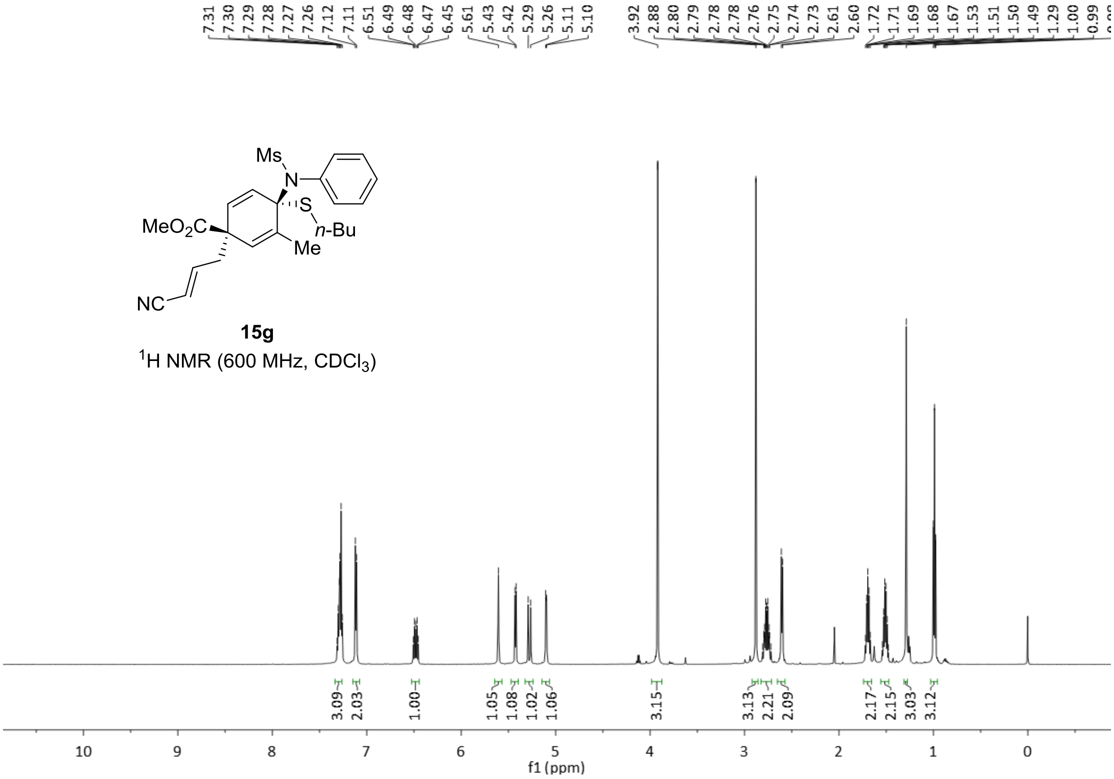

Supplementary Figure 182. ^{1}H NMR spectrum of **15g**

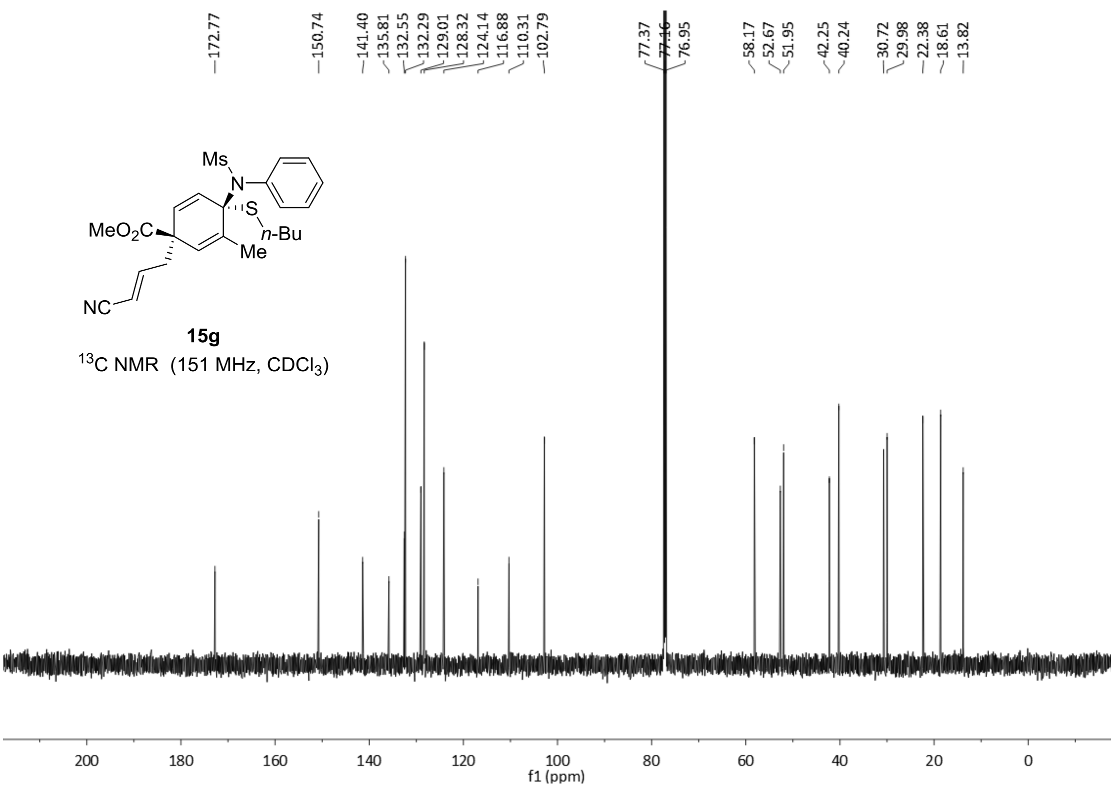

Supplementary Figure 183. ^{13}C NMR spectrum of **15g**

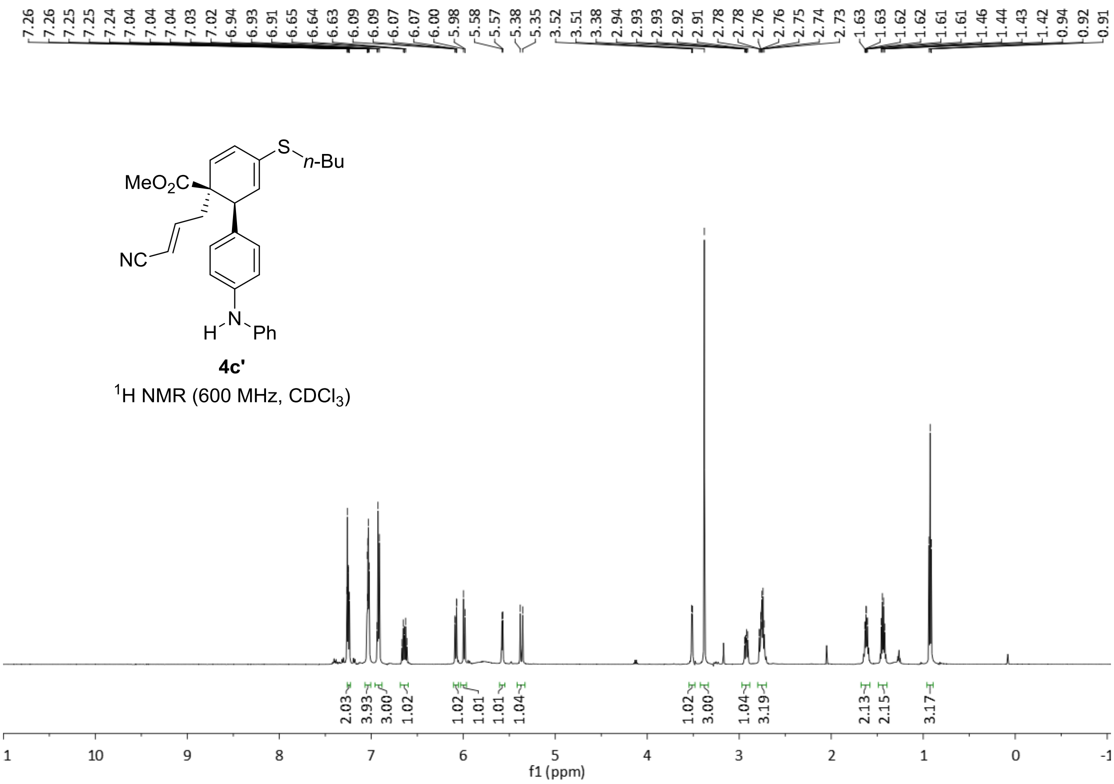

Supplementary Figure 184. ^{1}H NMR spectrum of **4c’**

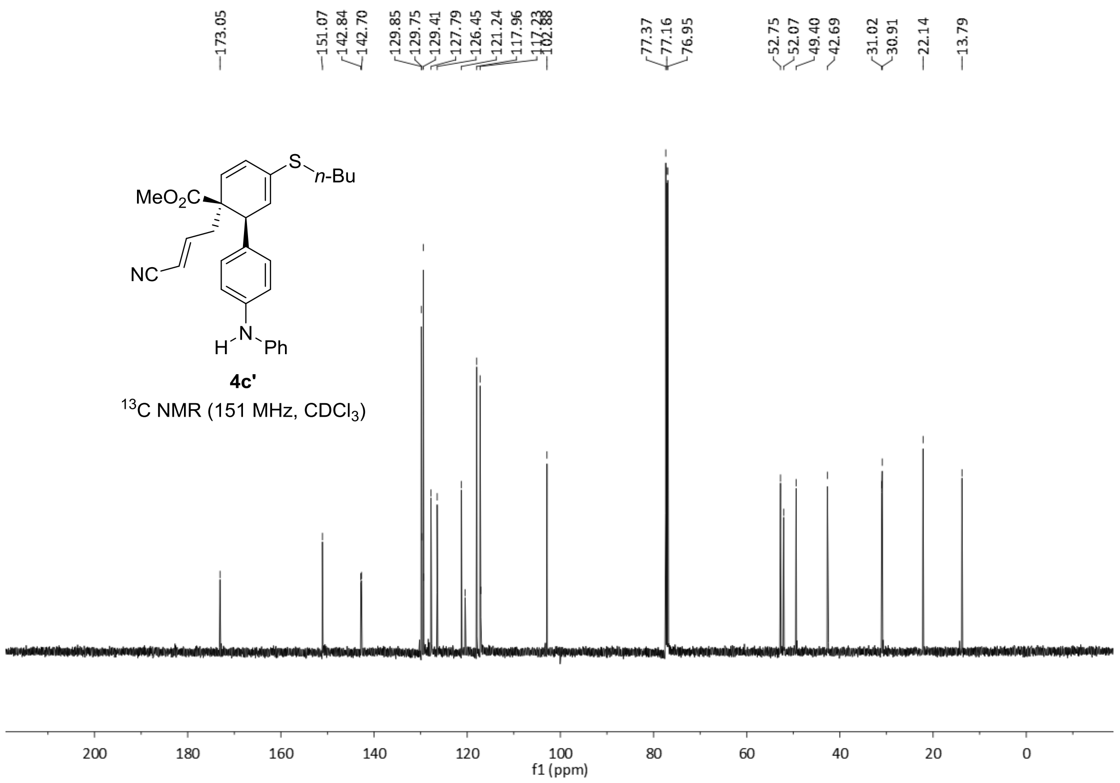

Supplementary Figure 185. ^{13}C NMR spectrum of **4c'**

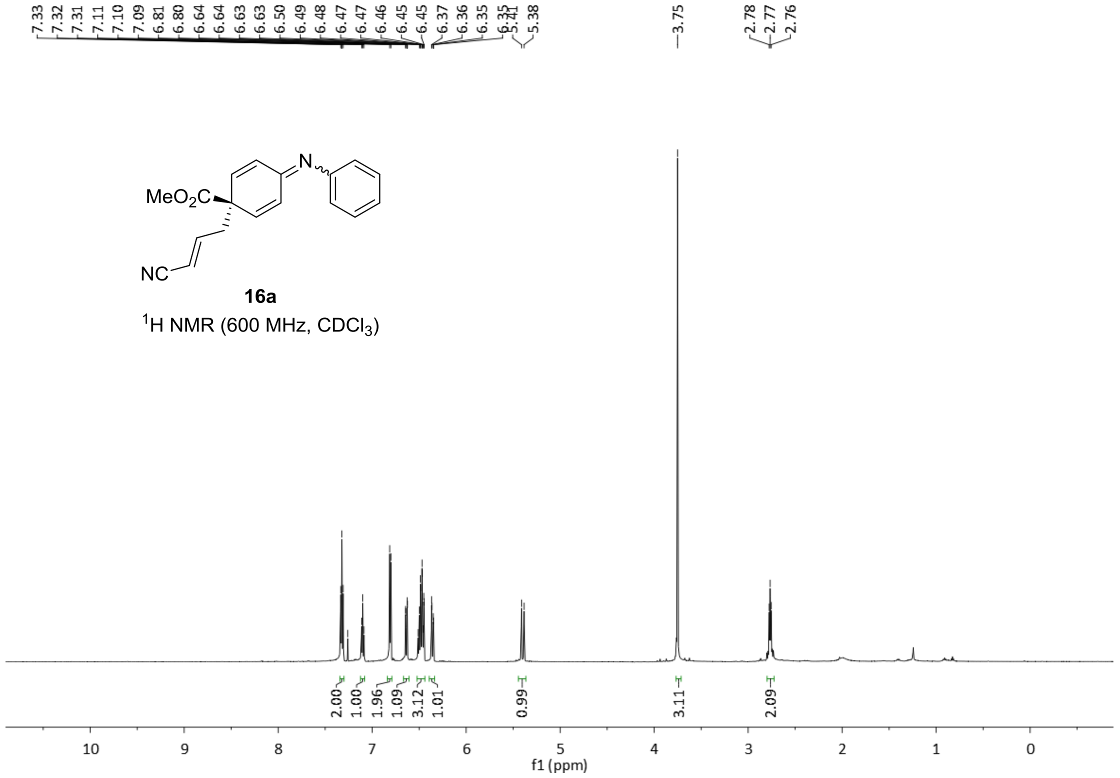

Supplementary Figure 186. ^{1}H NMR spectrum of **16a**

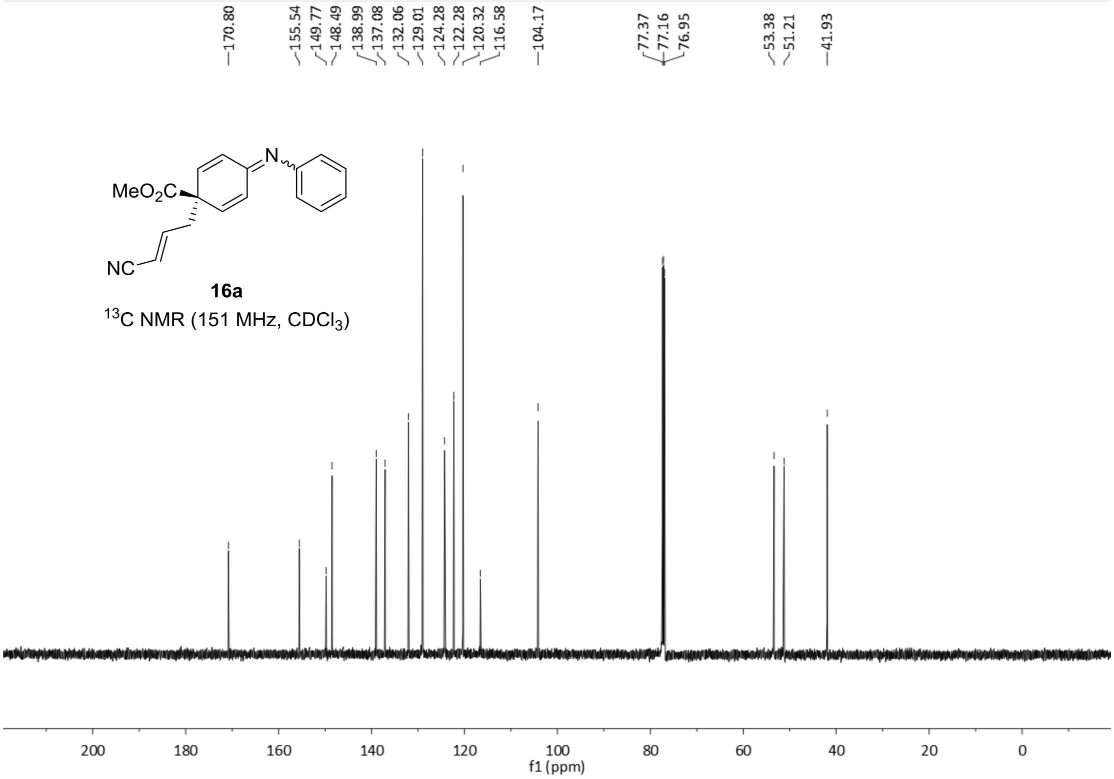

Supplementary Figure 187. ^{13}C NMR spectrum of **16a**

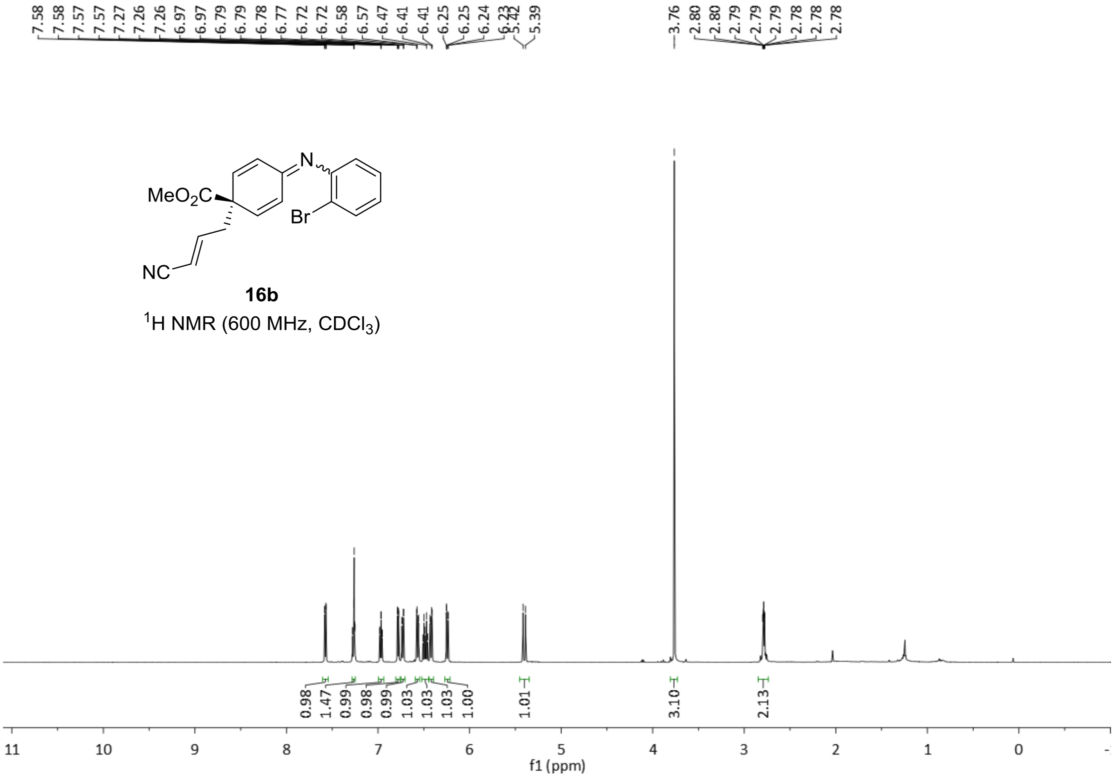

Supplementary Figure 188. ^{1}H NMR spectrum of **16b**

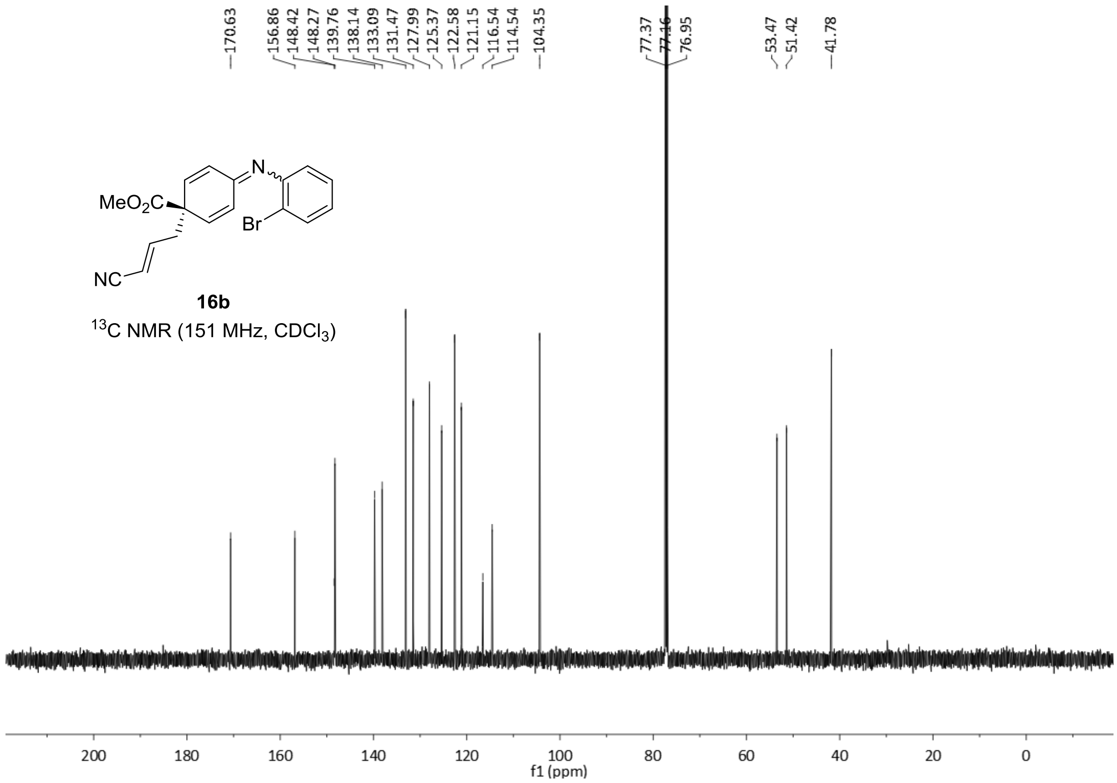

Supplementary Figure 189. ^{13}C NMR spectrum of **16b**

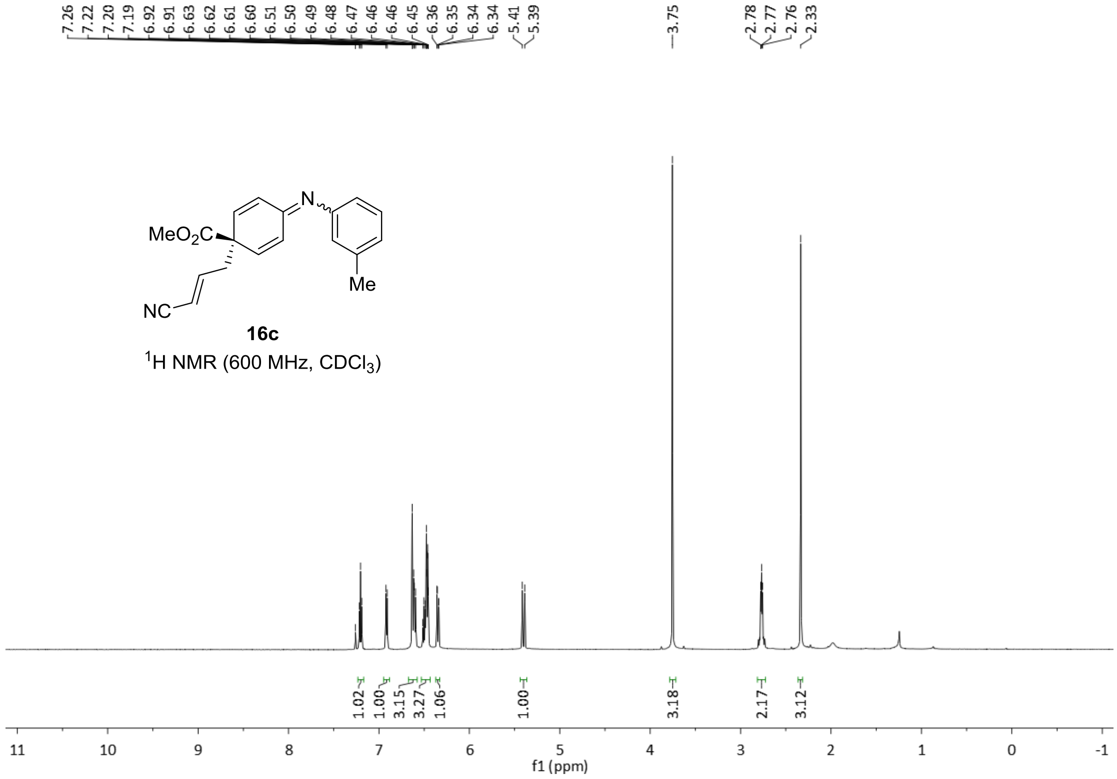

Supplementary Figure 190. ^{1}H NMR spectrum of **16c**

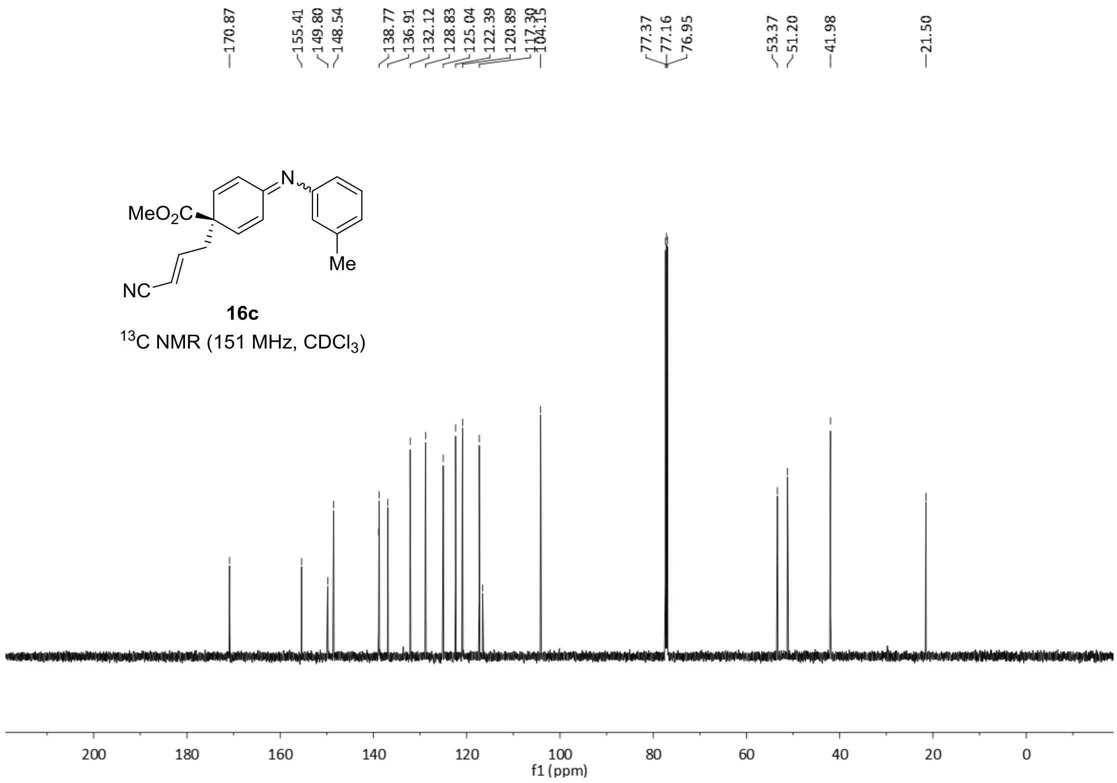

Supplementary Figure 191. ^{13}C NMR spectrum of **16c**

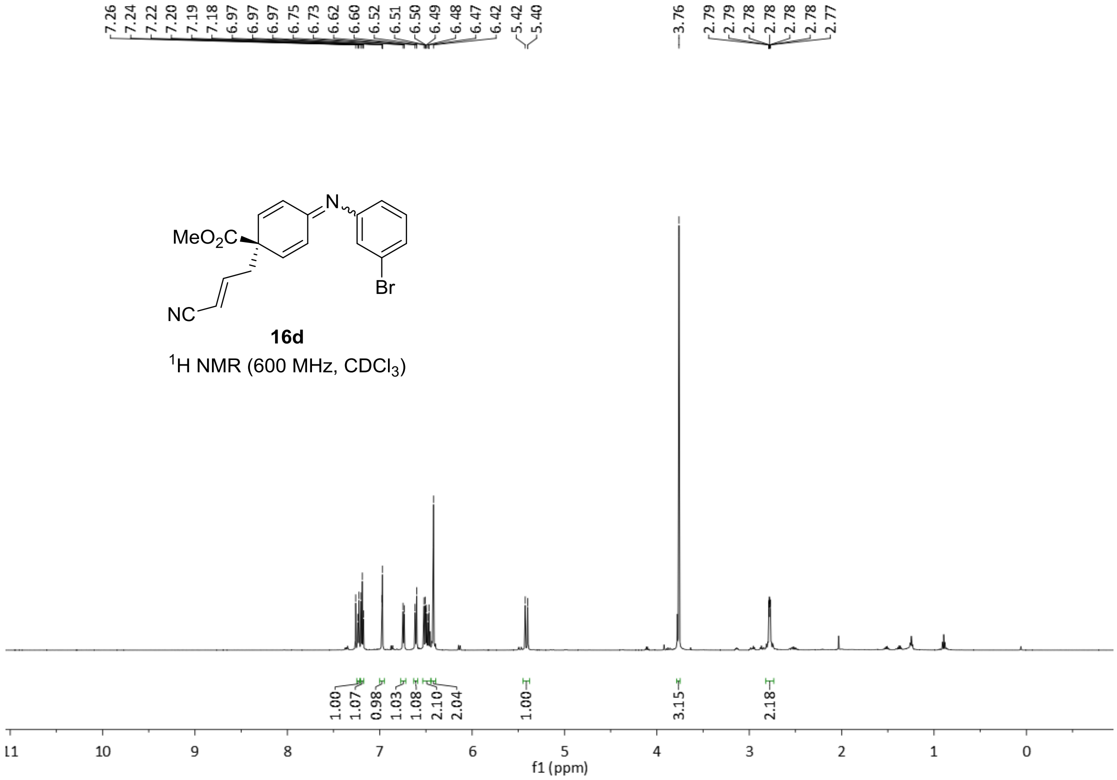

Supplementary Figure 192. ^{1}H NMR spectrum of **16d**

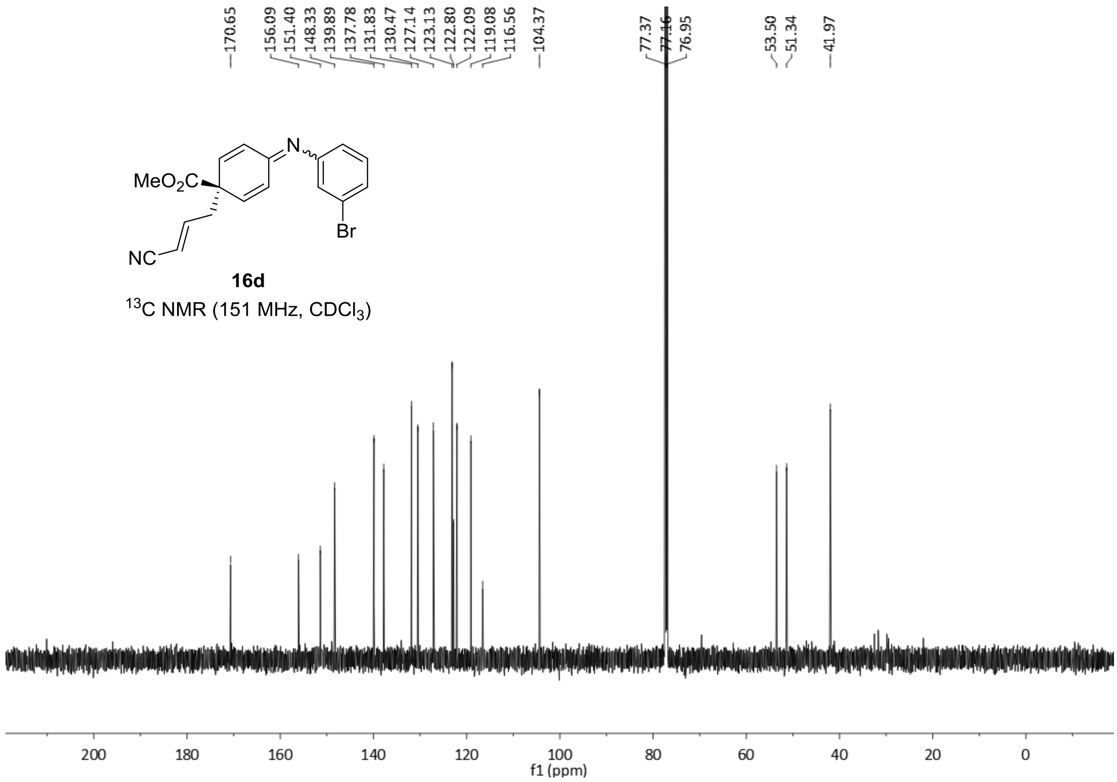

Supplementary Figure 193. ^{13}C NMR spectrum of **16d**

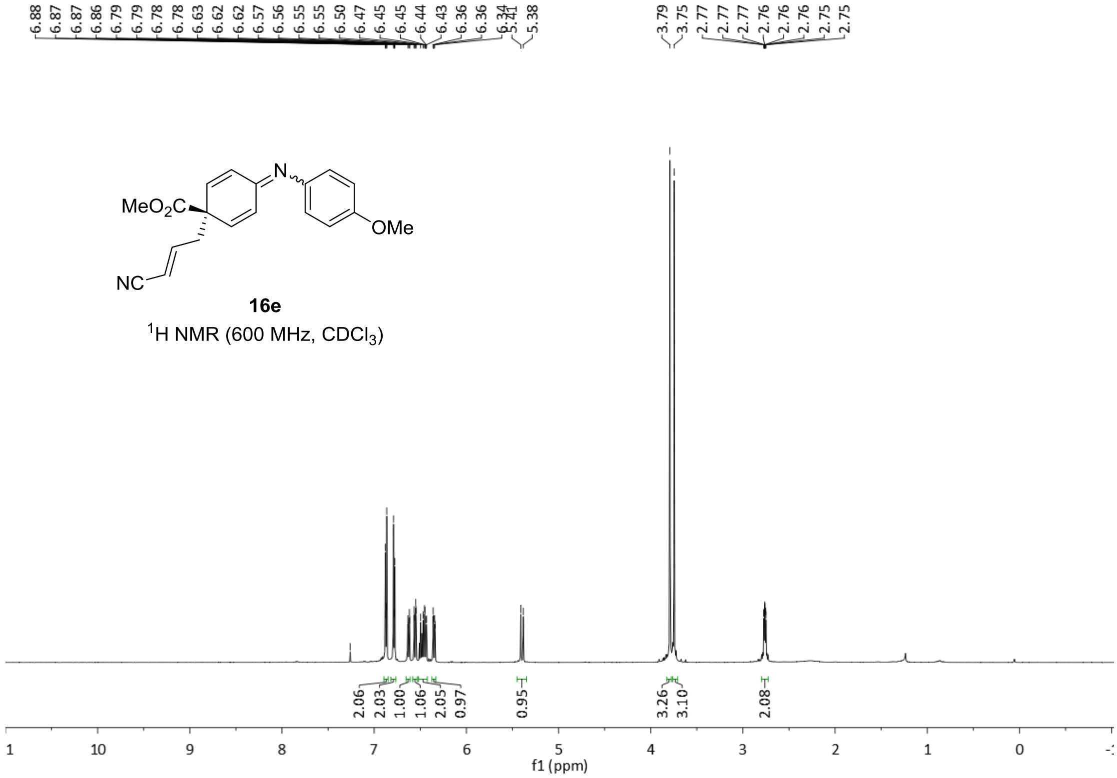

Supplementary Figure 194. ^{1}H NMR spectrum of **16e**

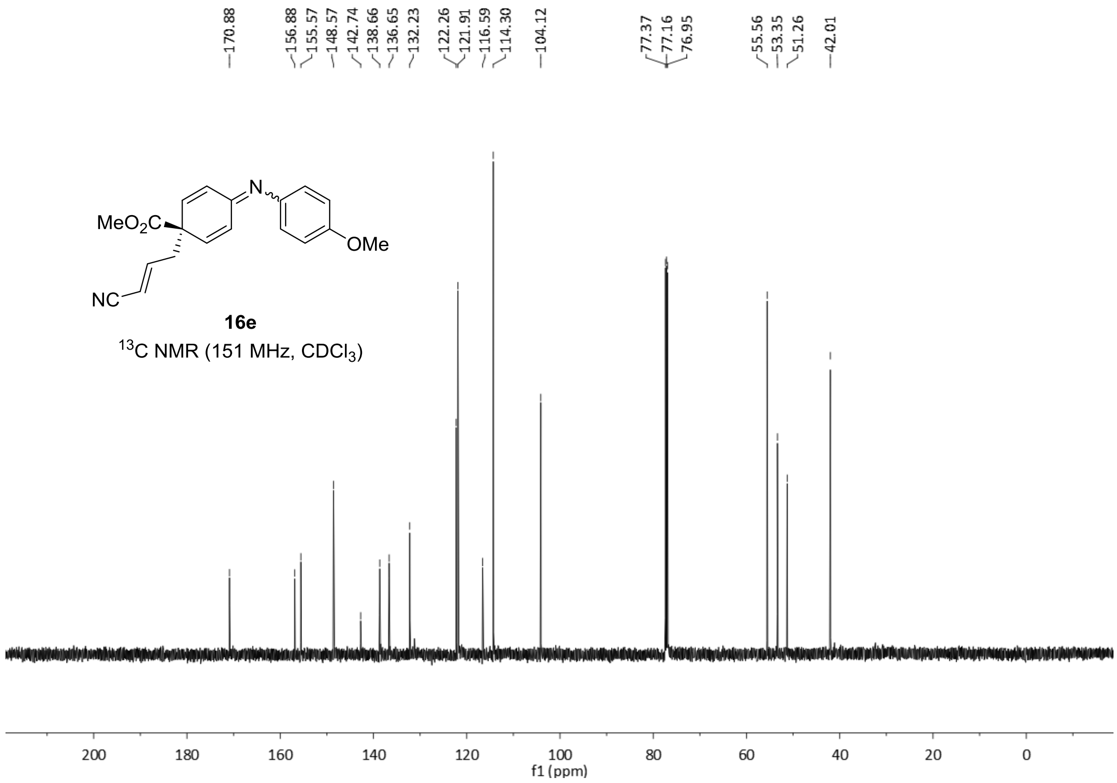

Supplementary Figure 195. ^{13}C NMR spectrum of **16e**

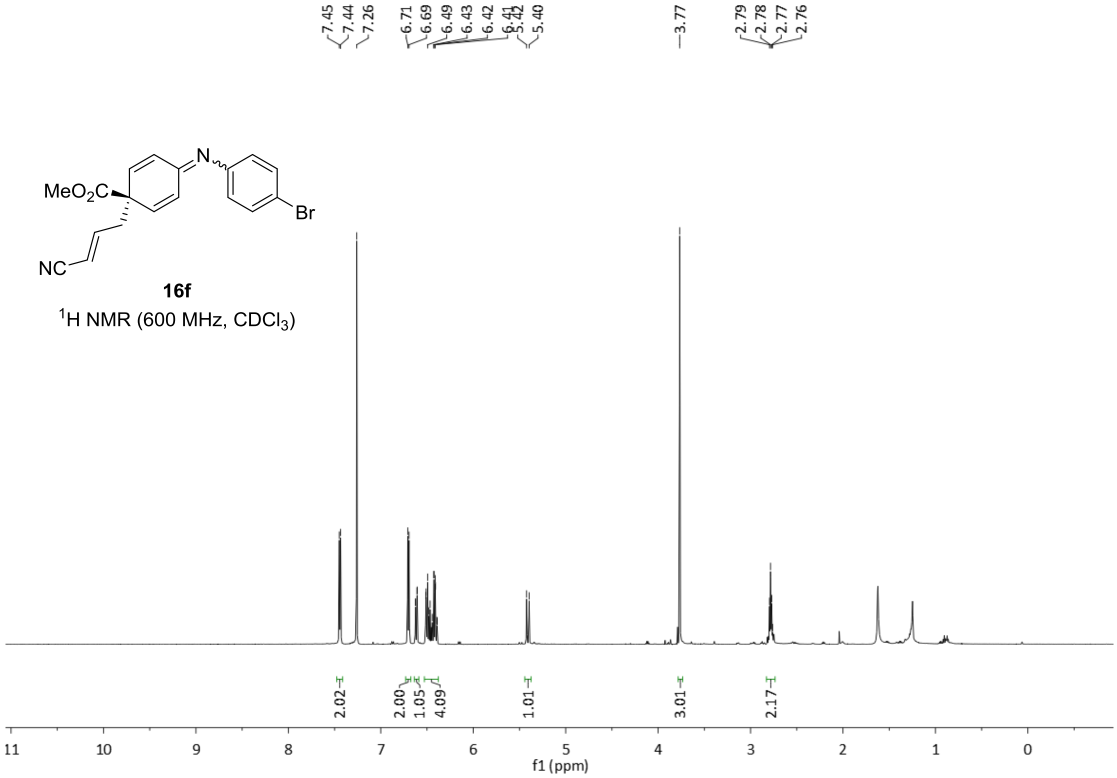

Supplementary Figure 196. ^{1}H NMR spectrum of **16f**

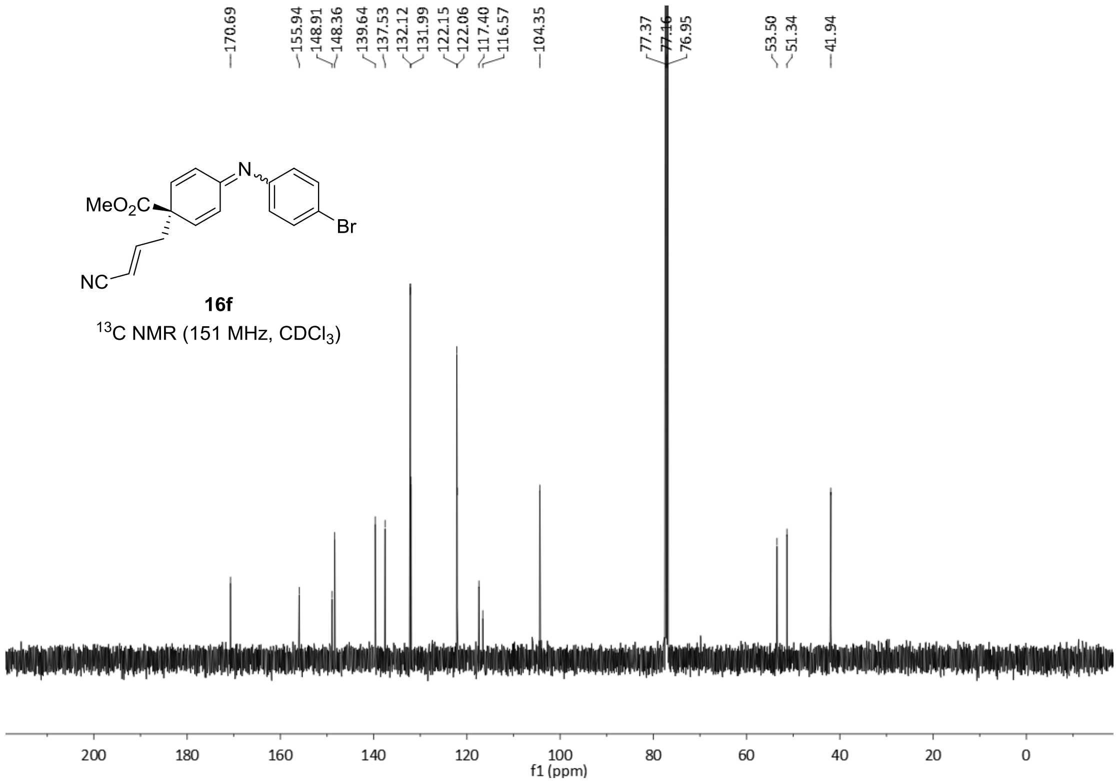

Supplementary Figure 197. ^{13}C NMR spectrum of **16f**

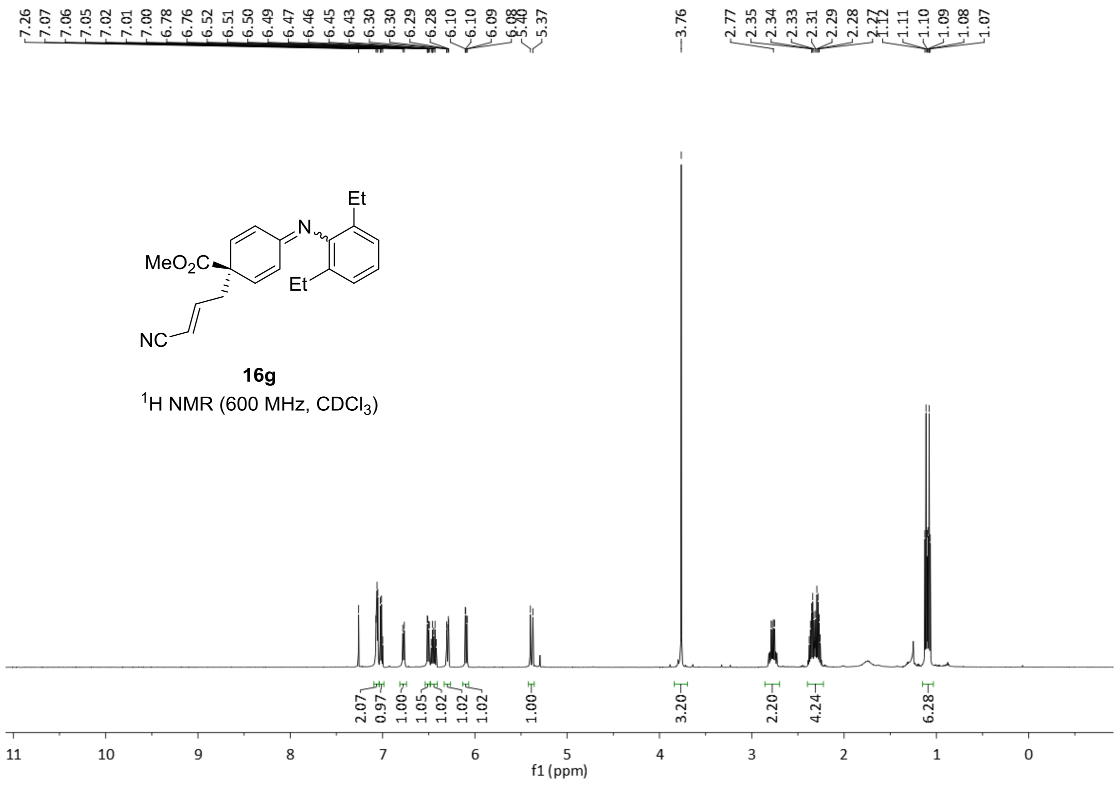

Supplementary Figure 198. ^{1}H NMR spectrum of **16g**

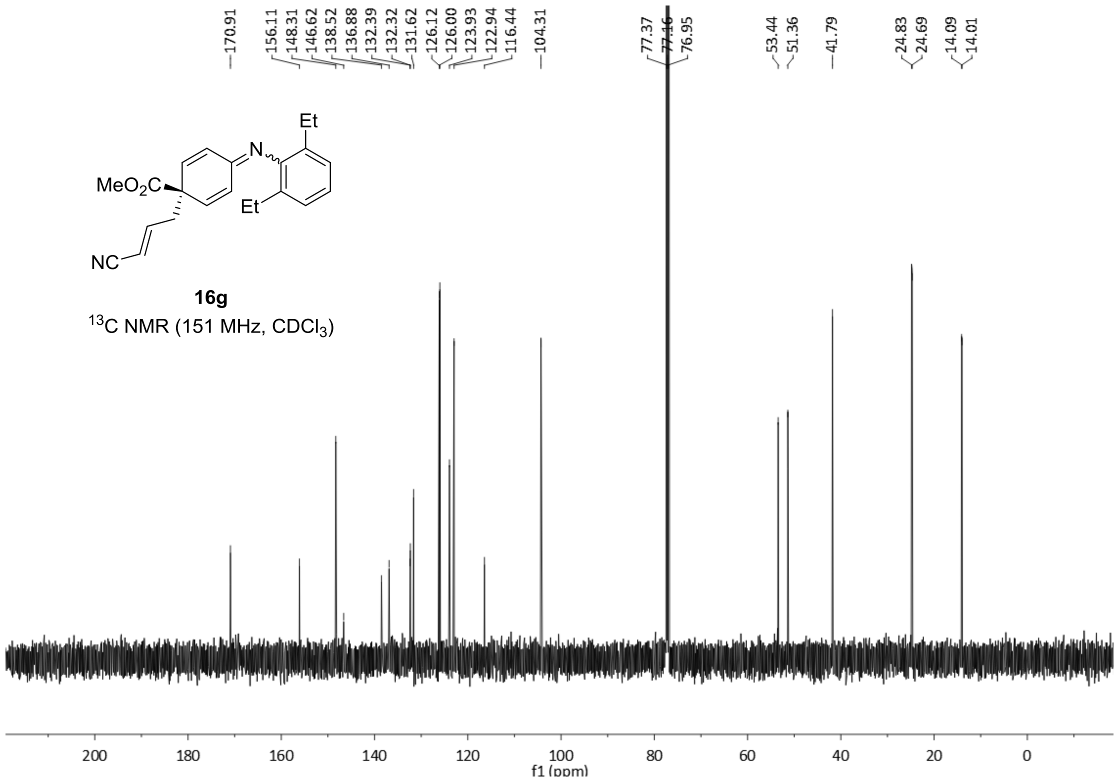

Supplementary Figure 199. ^{13}C NMR spectrum of **16g**

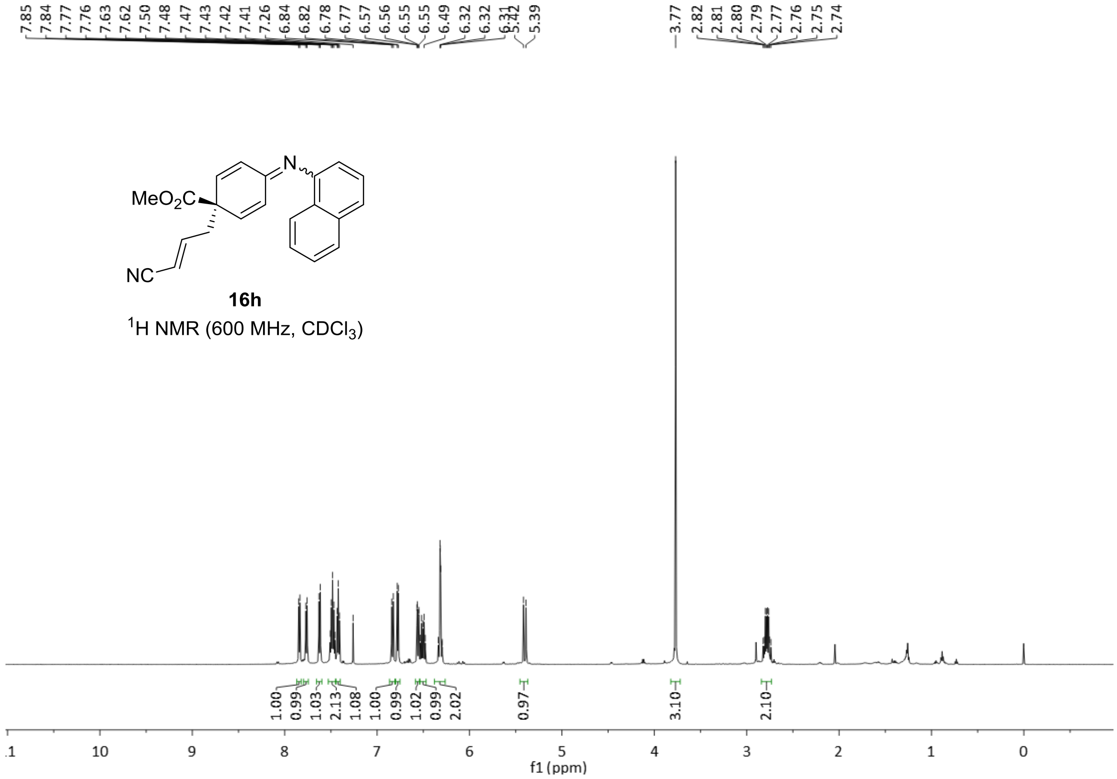

Supplementary Figure 200. ^{1}H NMR spectrum of **16h**

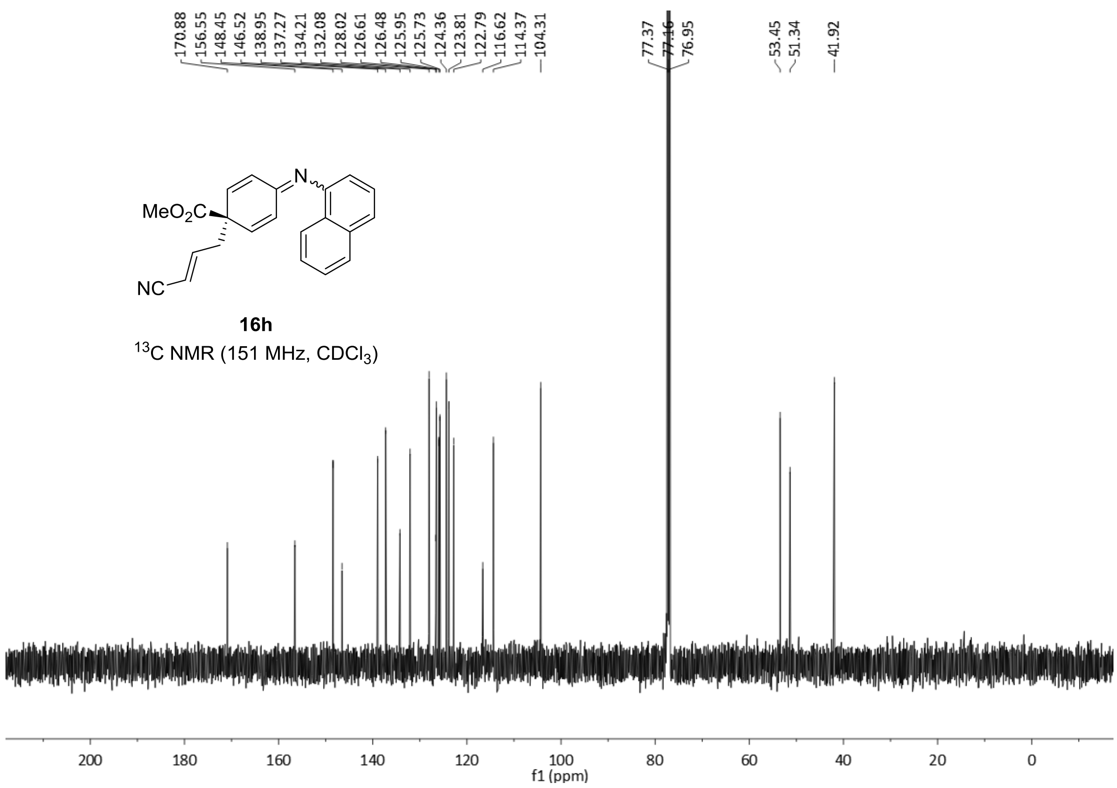

Supplementary Figure 201. ^{13}C NMR spectrum of **16h**

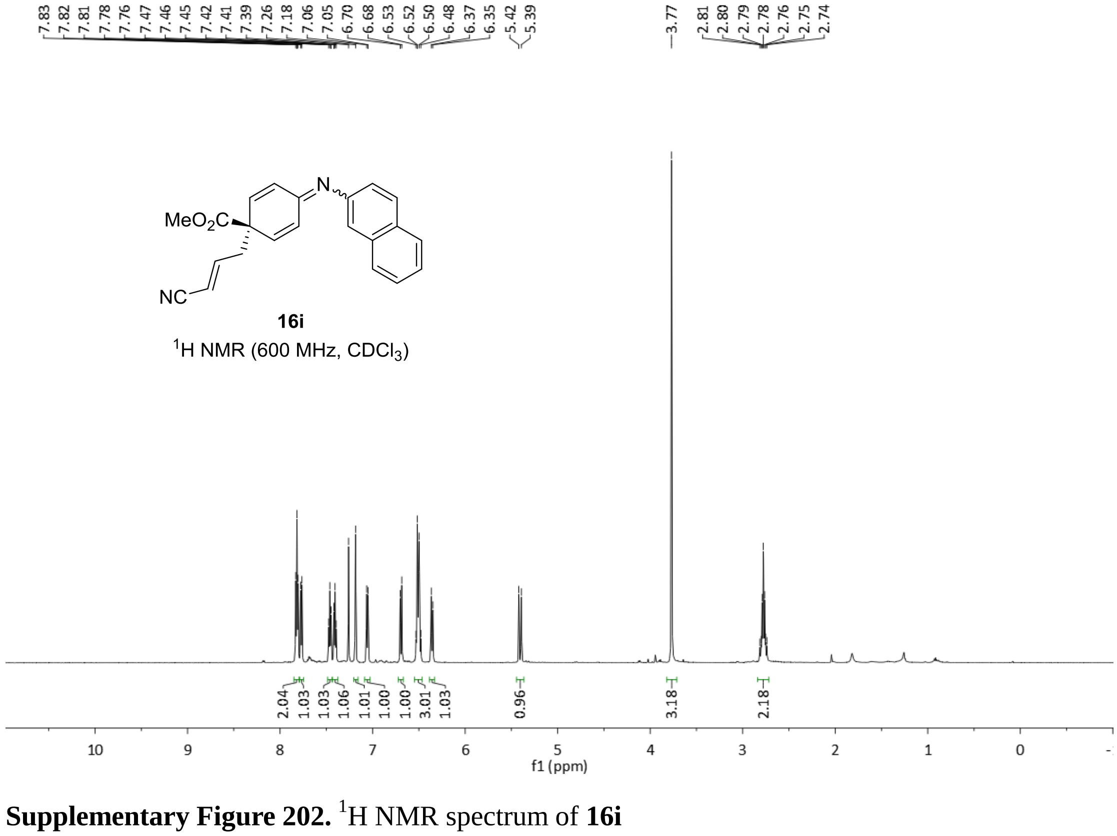

Supplementary Figure 202. ^{1}H NMR spectrum of **16i**

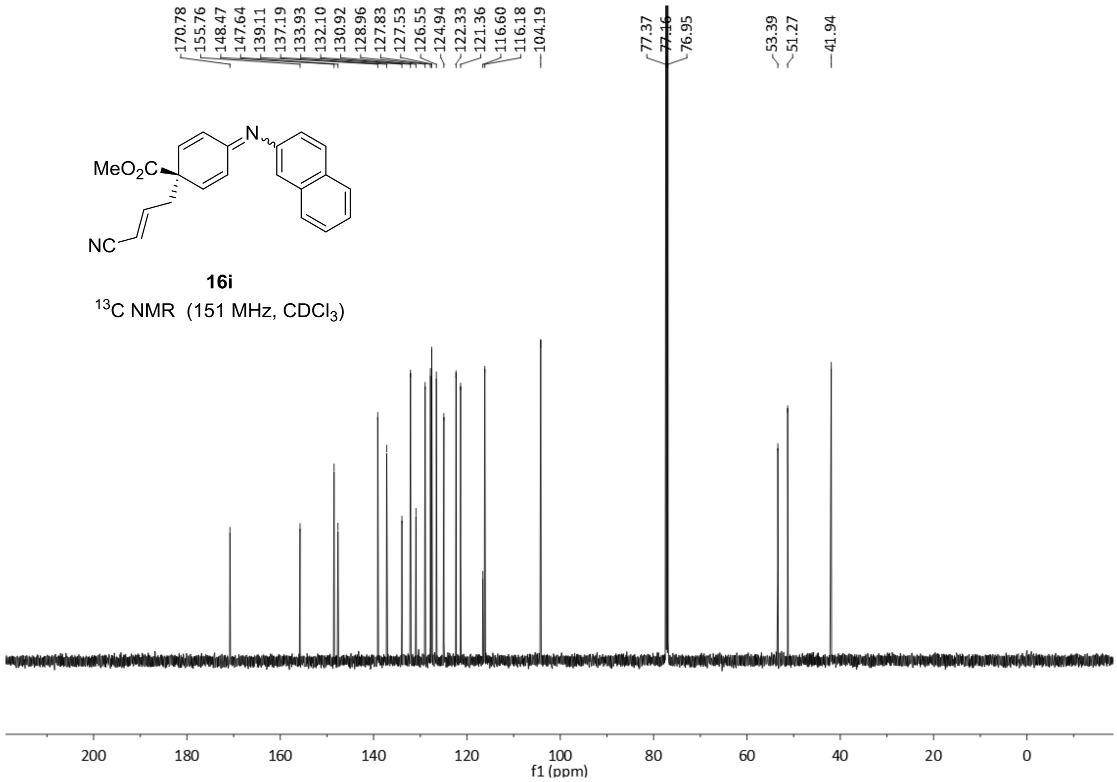

Supplementary Figure 203. ^{13}C NMR spectrum of **16i**

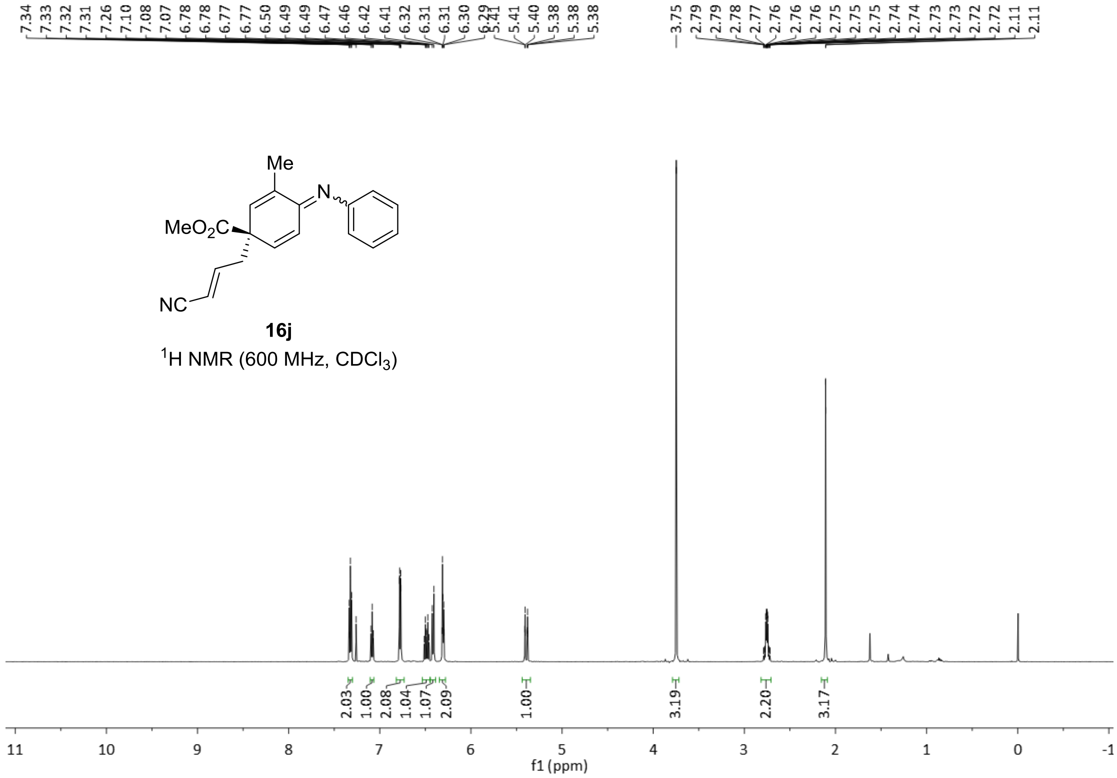

Supplementary Figure 204. ^{1}H NMR spectrum of **16j**

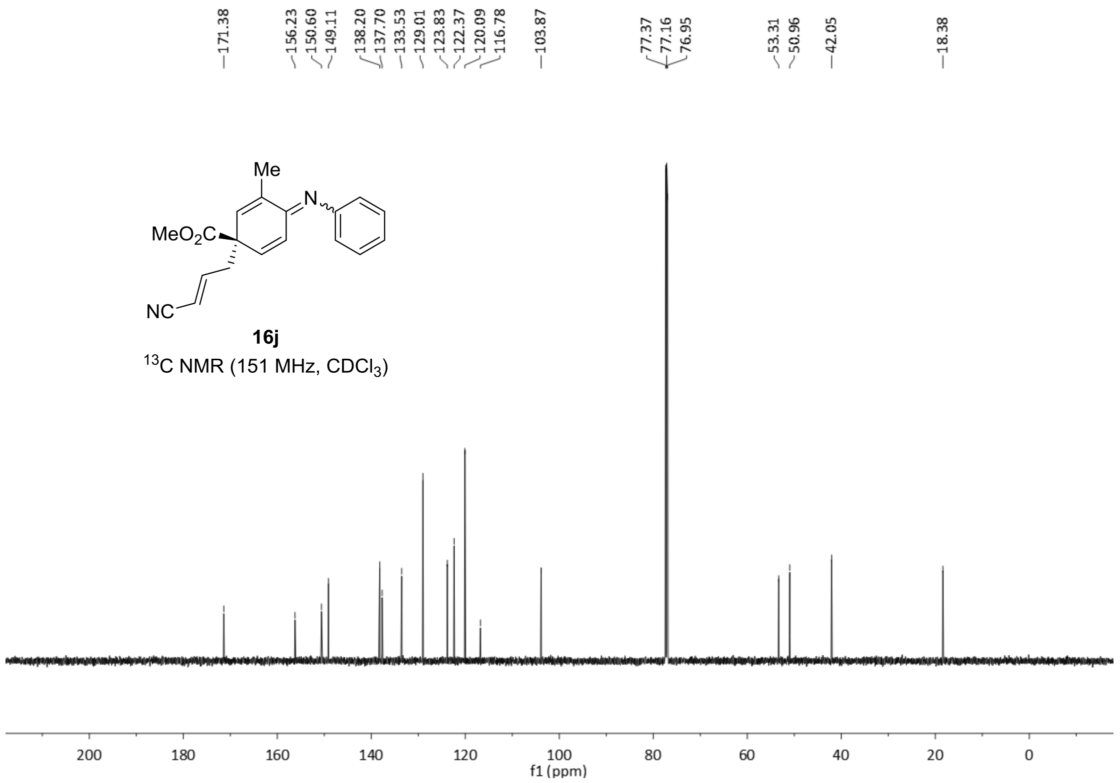

Supplementary Figure 205. ^{13}C NMR spectrum of **16j**

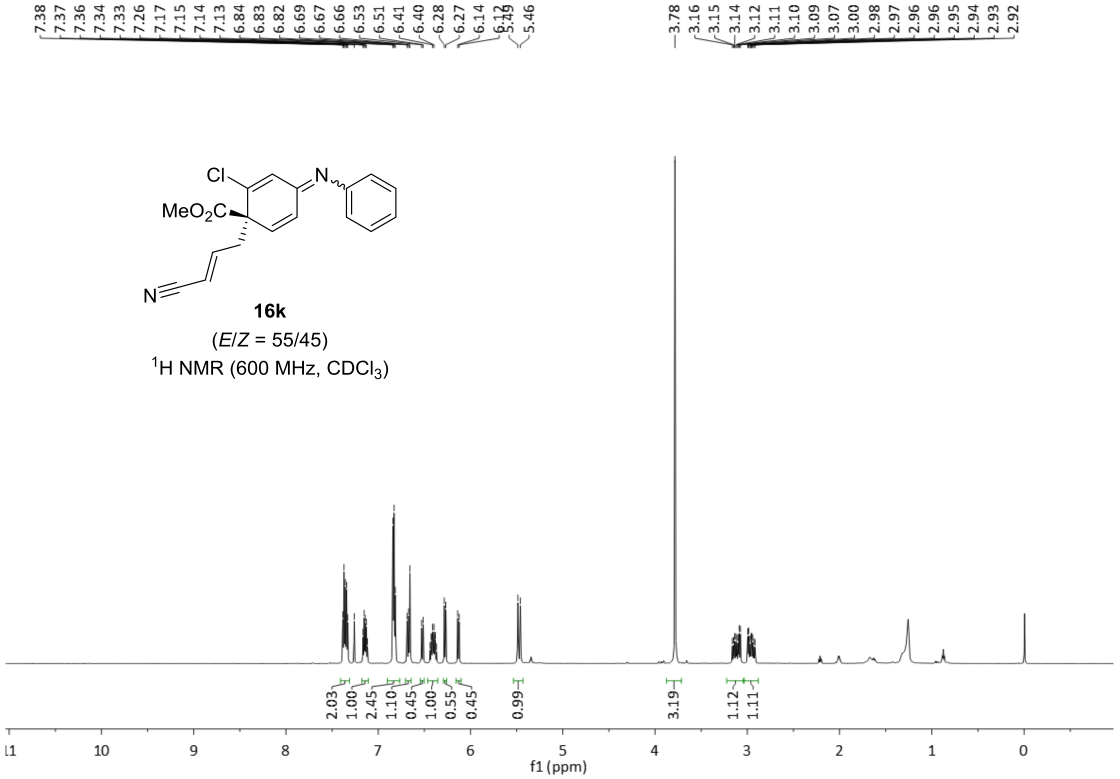

Supplementary Figure 206. ^{1}H NMR spectrum of 1**6k**

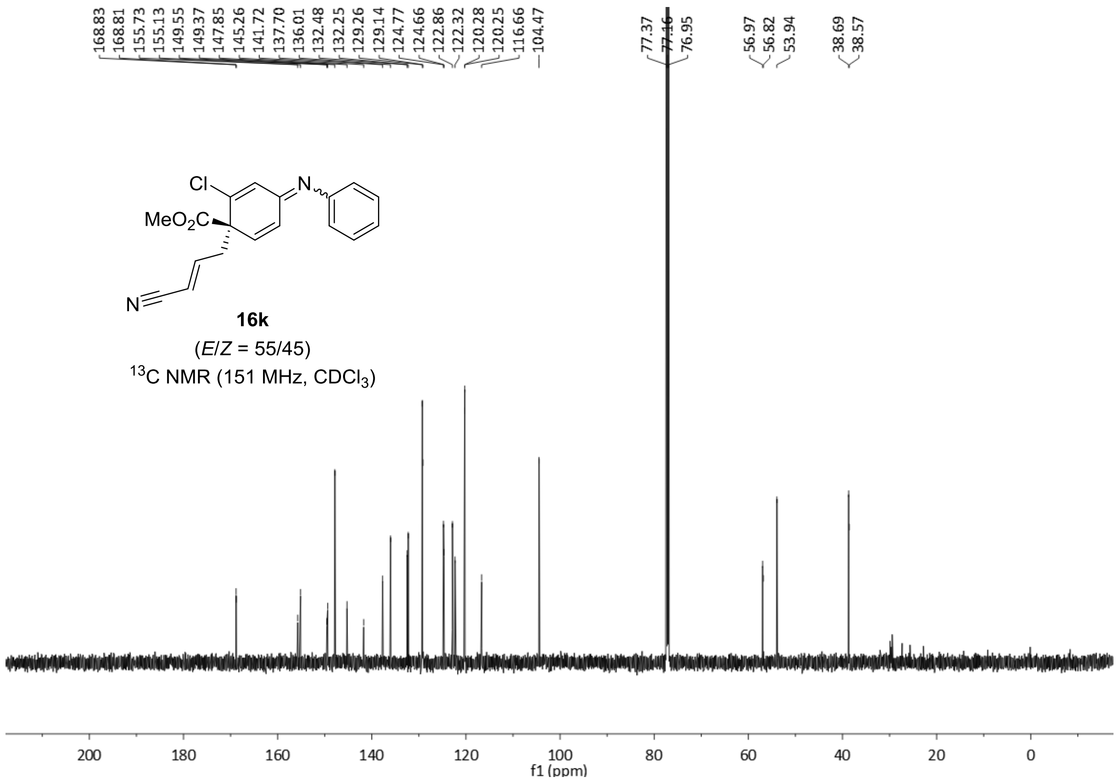

Supplementary Figure 207. ^{13}C NMR spectrum of **16k**

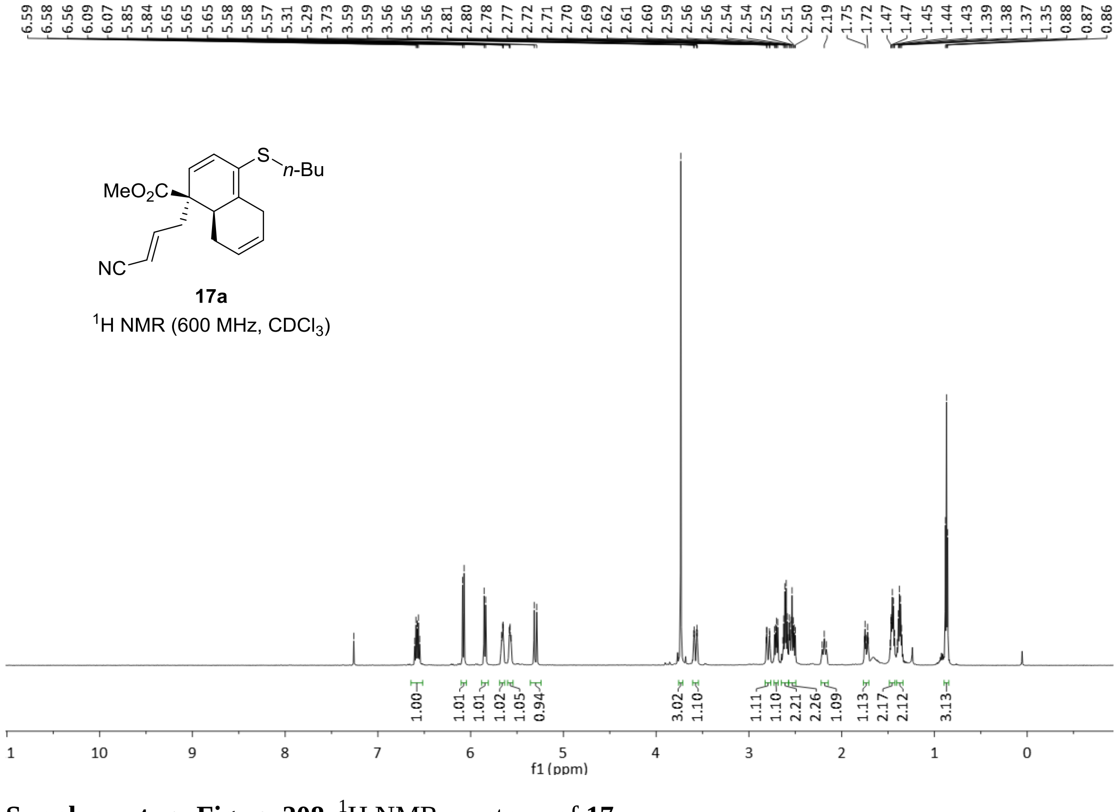

Supplementary Figure 208. ^{1}H NMR spectrum of **17a**

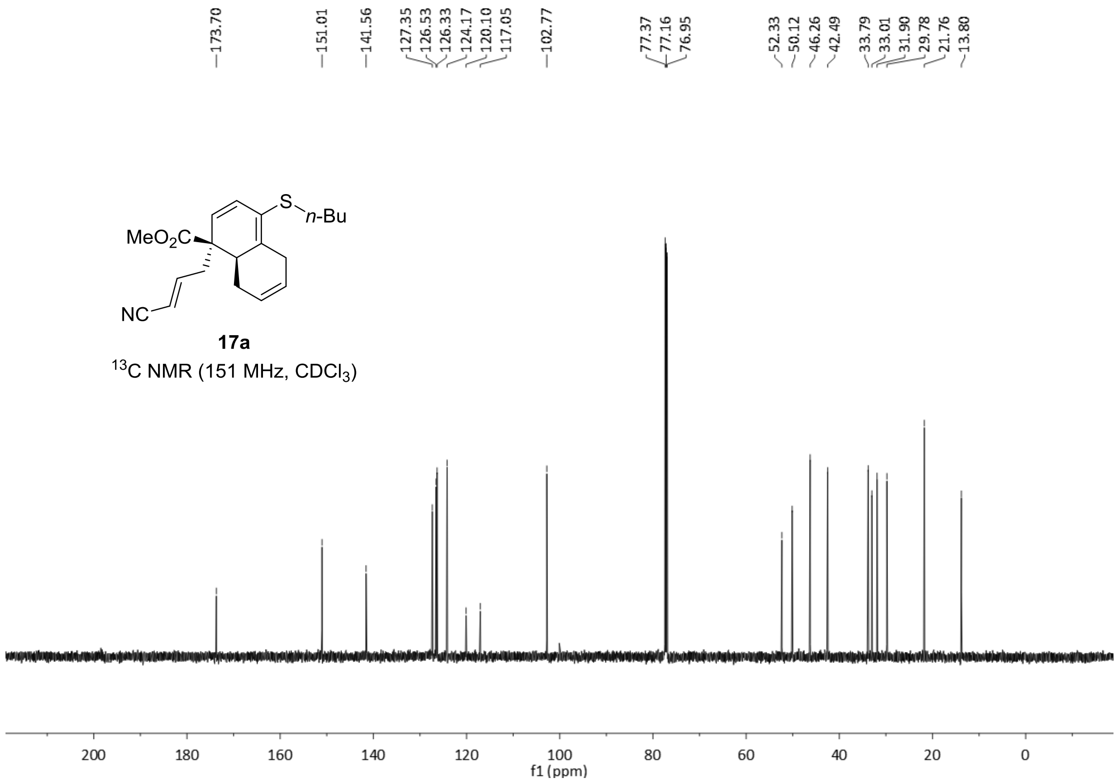

Supplementary Figure 209. ^{13}C NMR spectrum of **17a**

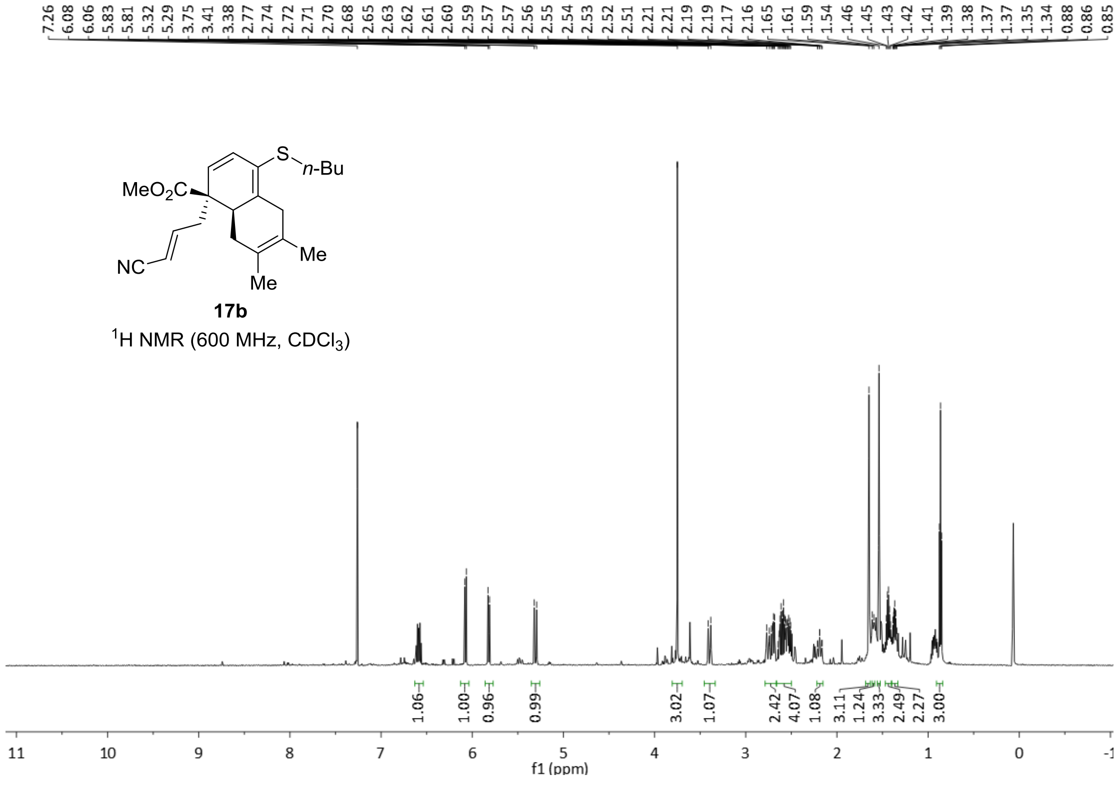

Supplementary Figure 210. ^{1}H NMR spectrum of **17b**

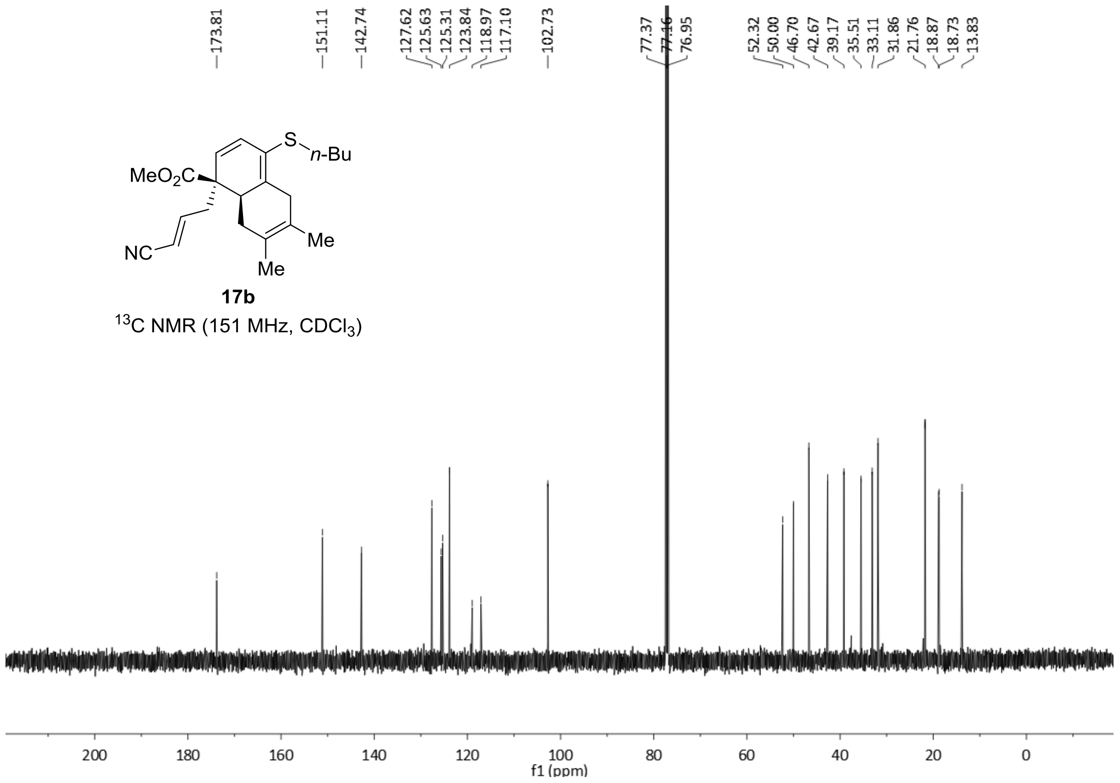

Supplementary Figure 211. ^{13}C NMR spectrum of **17b**

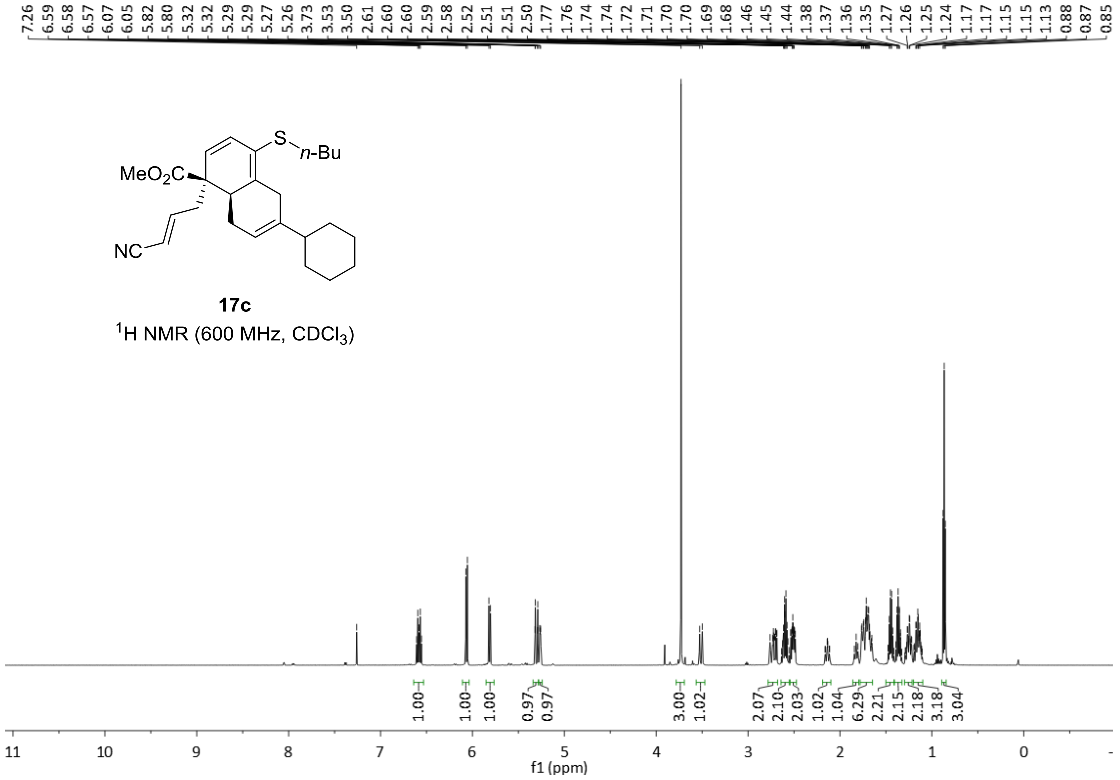

Supplementary Figure 212. ^{1}H NMR spectrum of **17c**

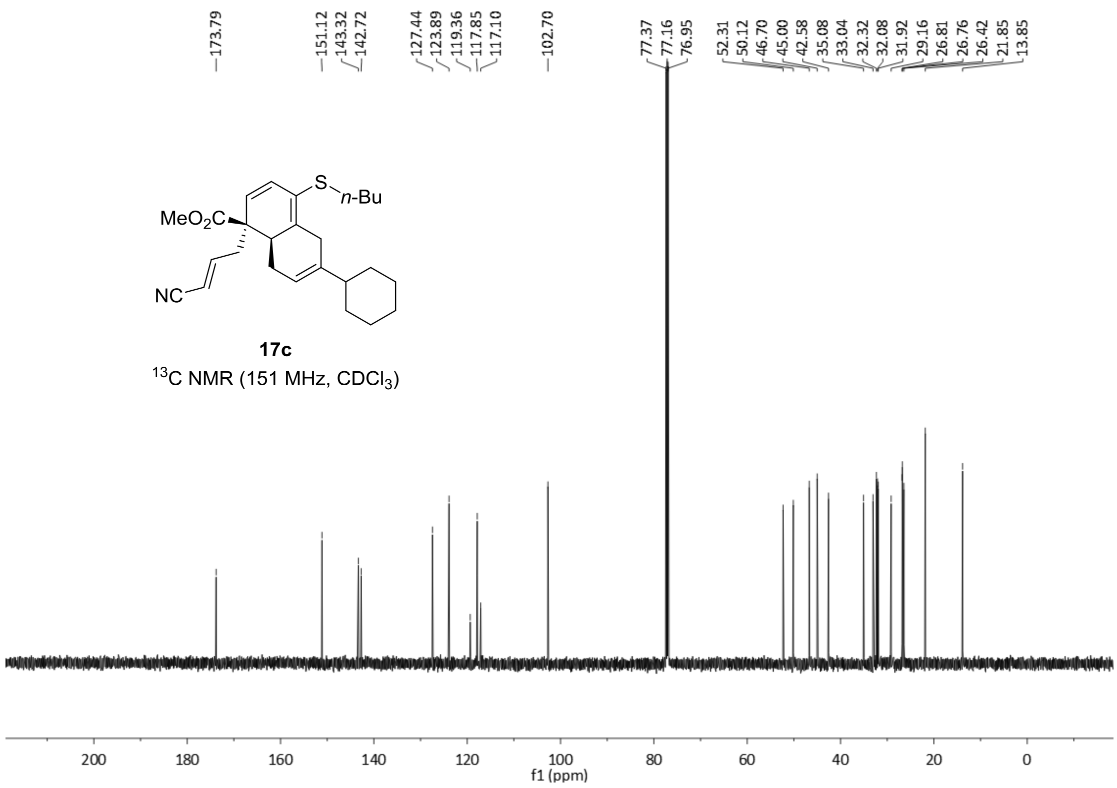

Supplementary Figure 213. ^{13}C NMR spectrum of **17c**

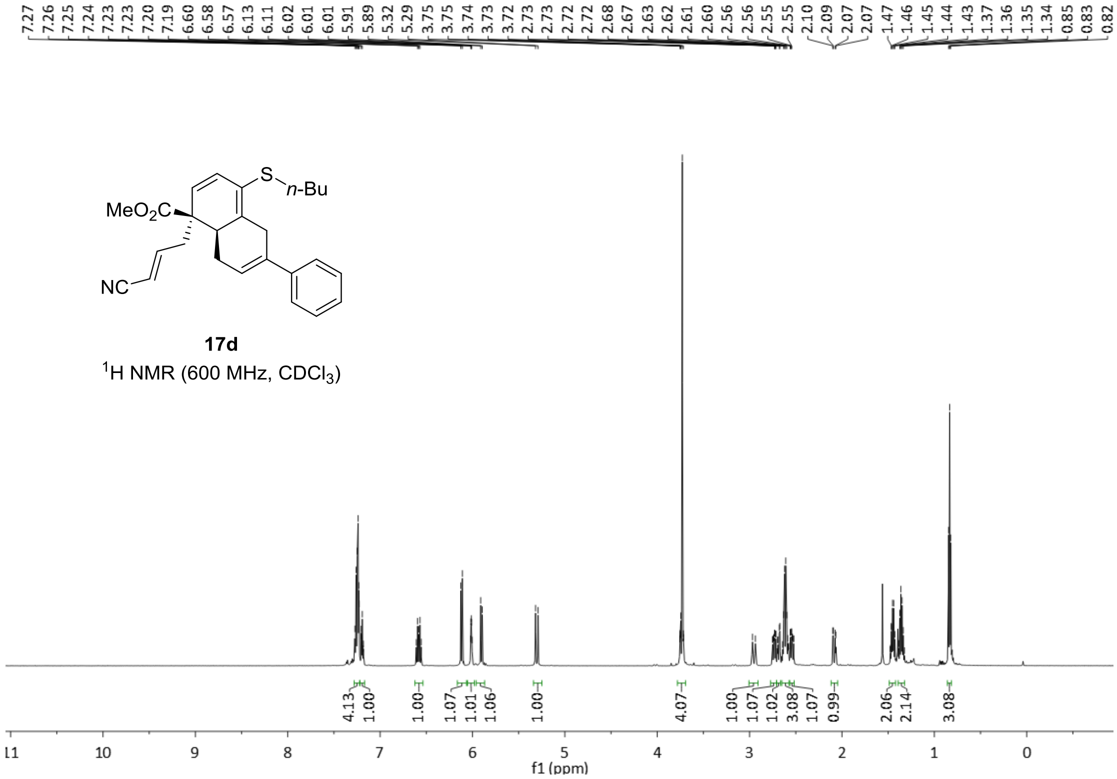

Supplementary Figure 214. ^{1}H NMR spectrum of **17d**

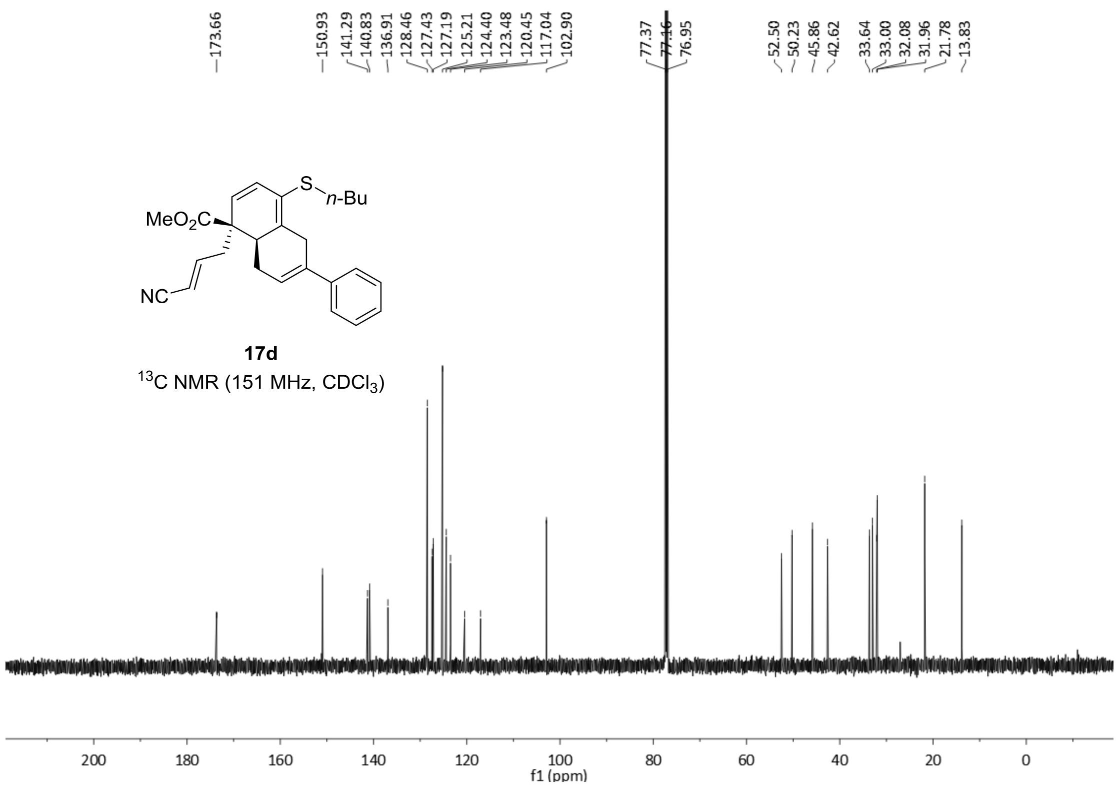

Supplementary Figure 215. ^{13}C NMR spectrum of **17d**

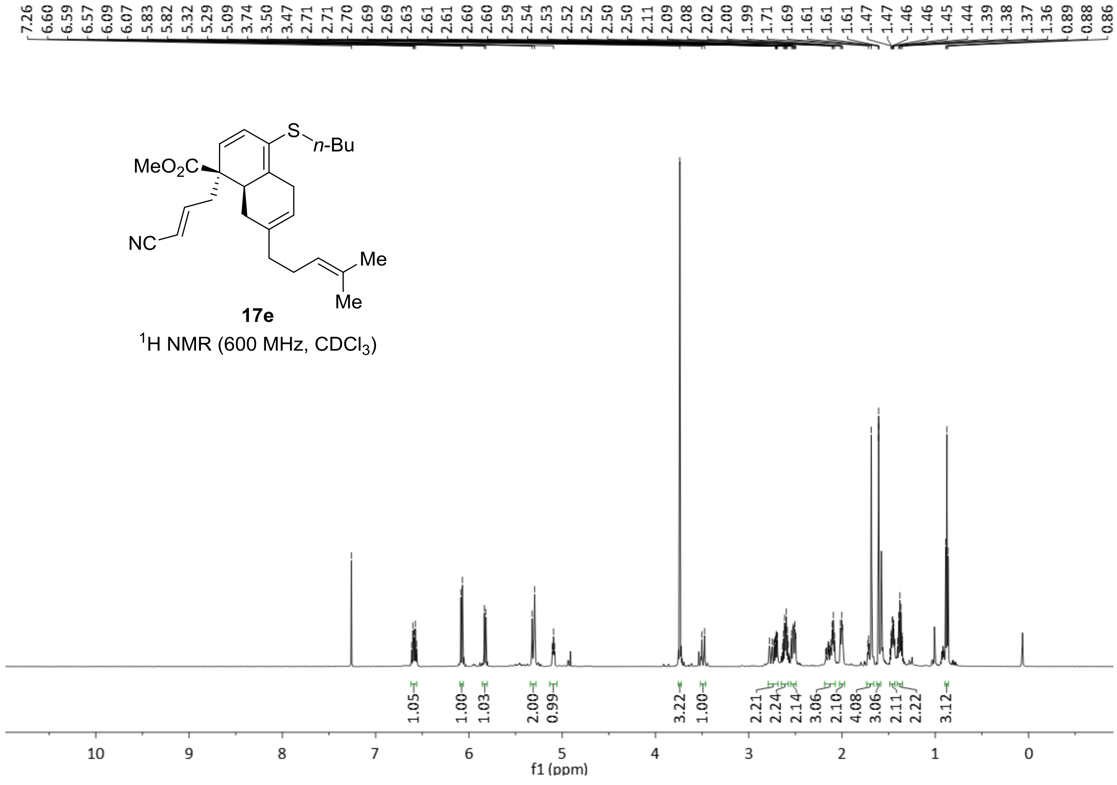

Supplementary Figure 216. ^{1}H NMR spectrum of **17e**

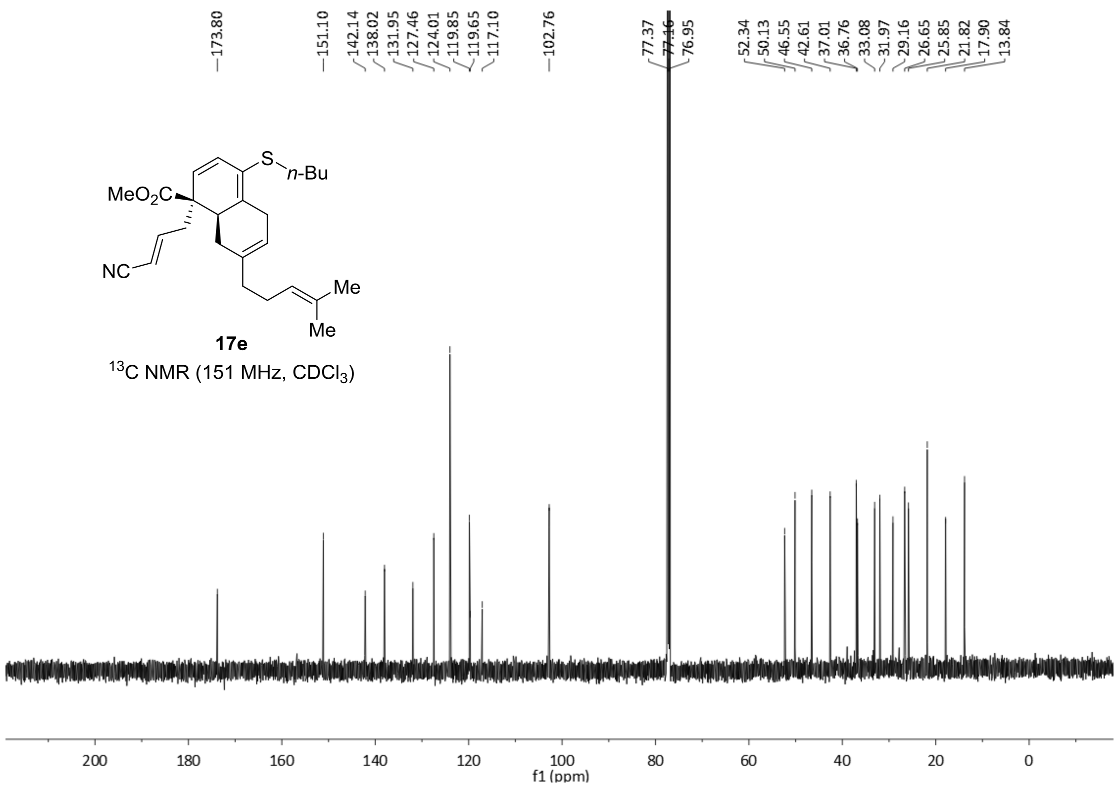

Supplementary Figure 217. ^{13}C NMR spectrum of **17e**

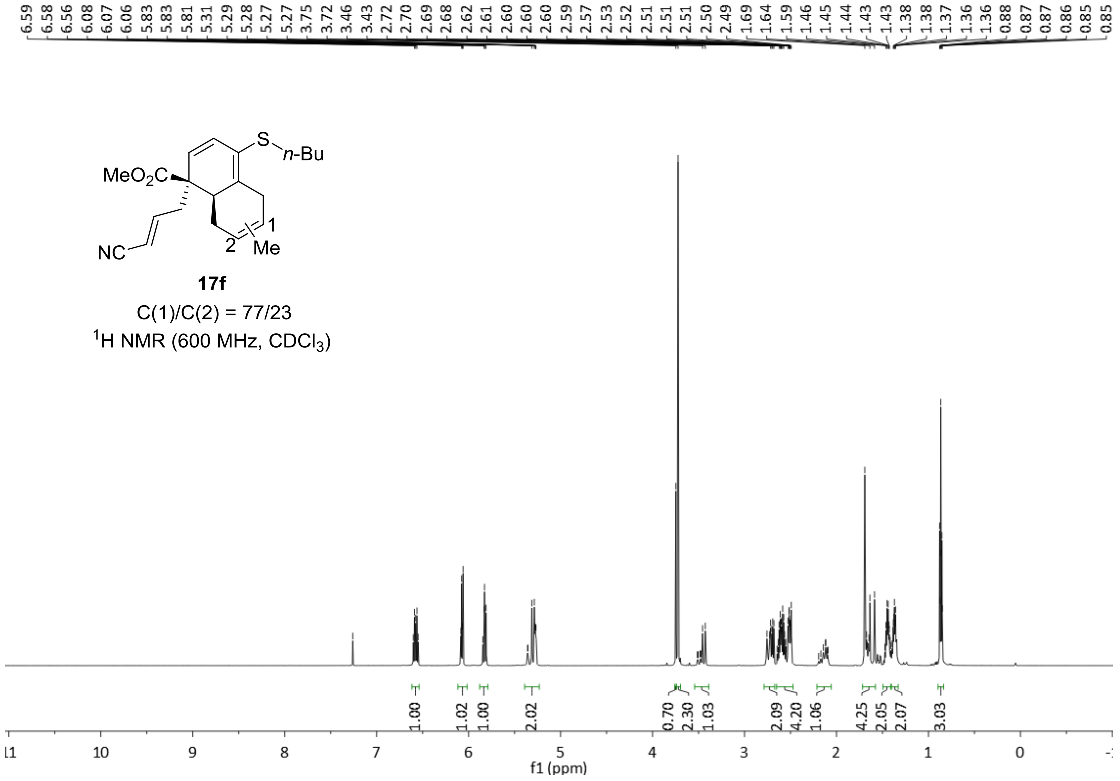

Supplementary Figure 218. ^{1}H NMR spectrum of **17f**

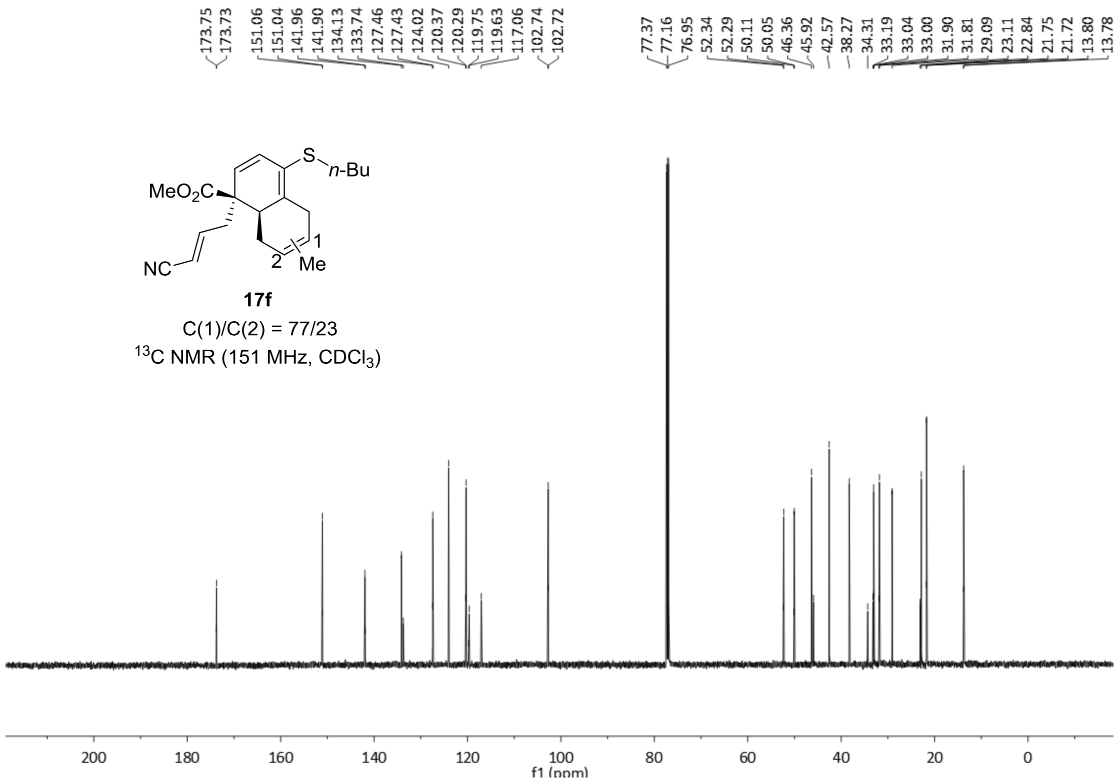

Supplementary Figure 219. ^{13}C NMR spectrum of **17f**

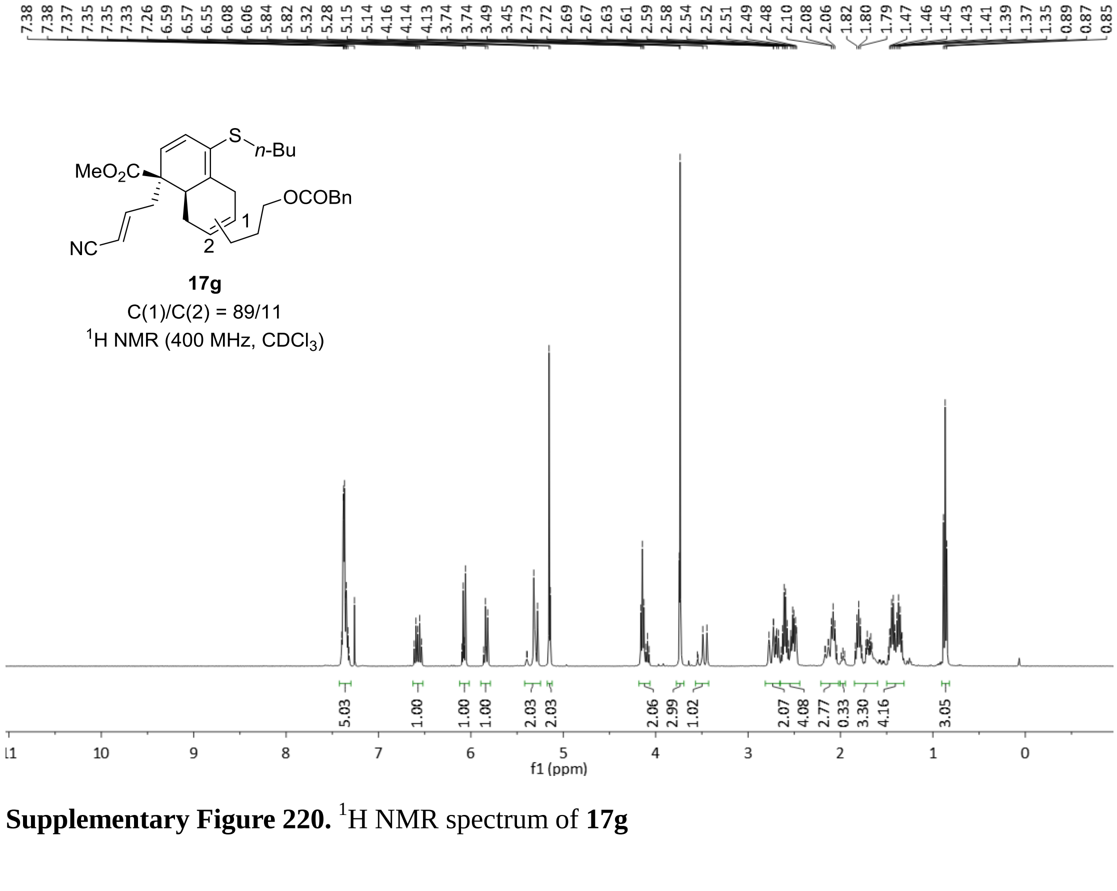

Supplementary Figure 220. ^{1}H NMR spectrum of **17g**

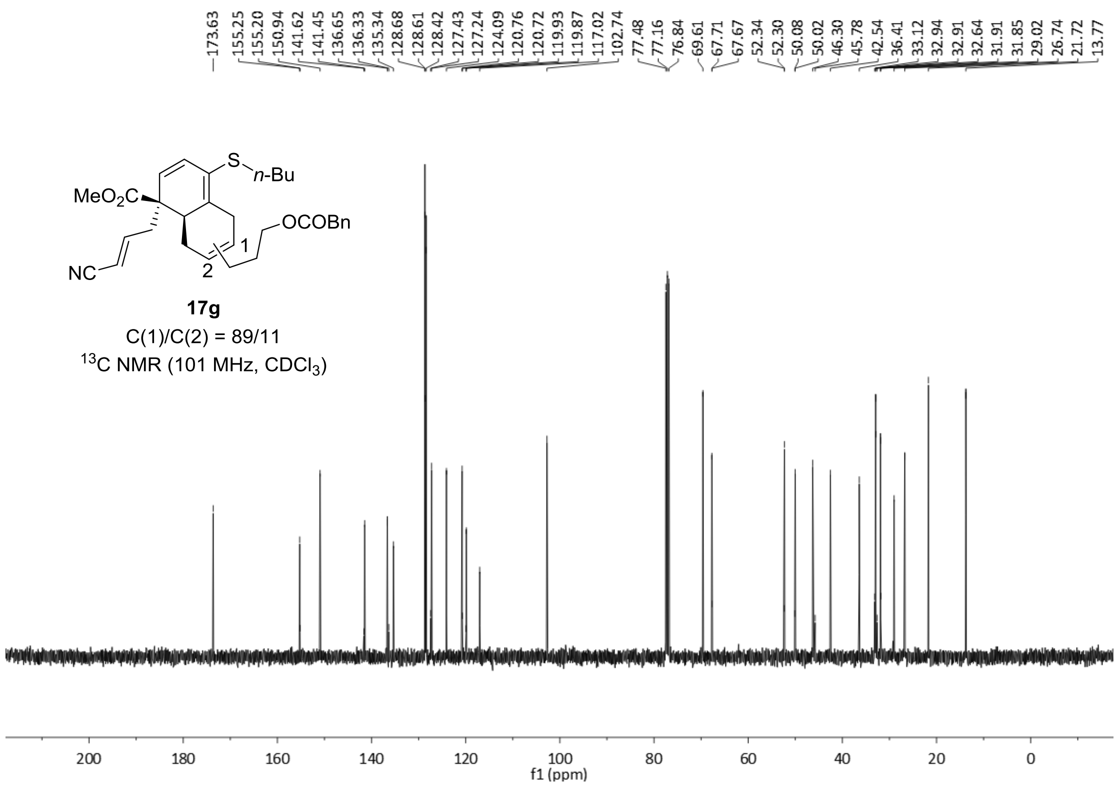

Supplementary Figure 221. ^{13}C NMR spectrum of **17g**

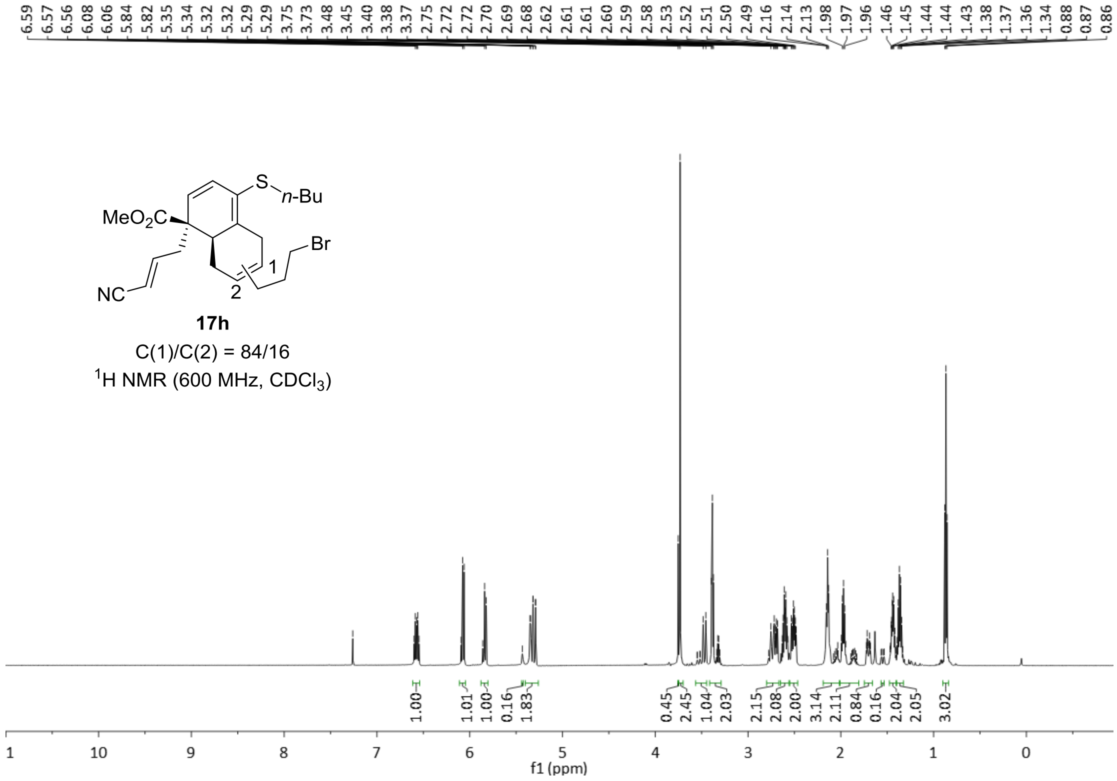

Supplementary Figure 222. ^{1}H NMR spectrum of **17h**

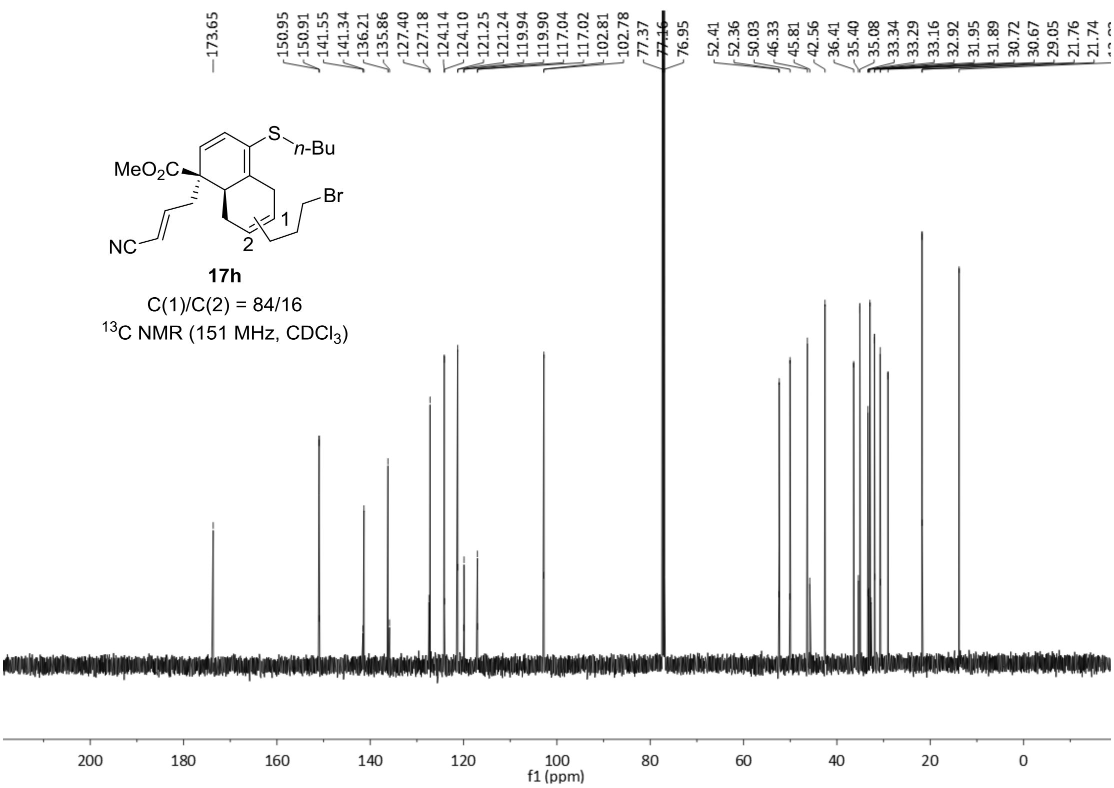

Supplementary Figure 223. ^{13}C NMR spectrum of **17h**

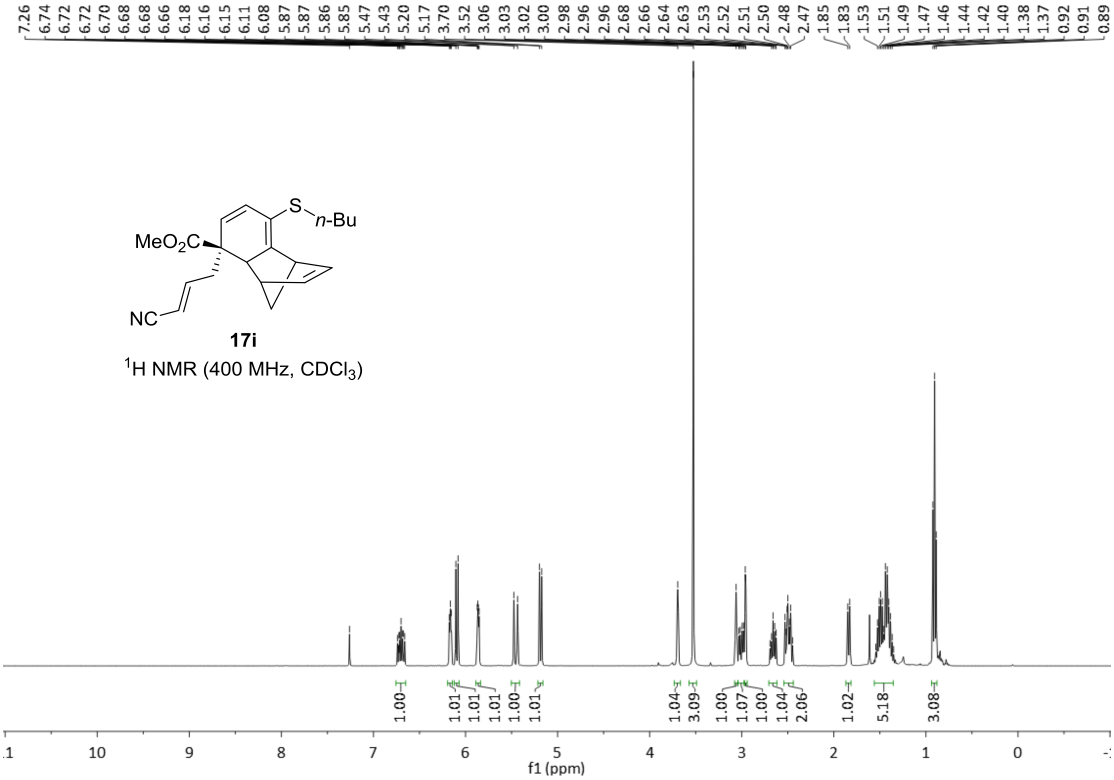

Supplementary Figure 224. ^{1}H NMR spectrum of **17i**

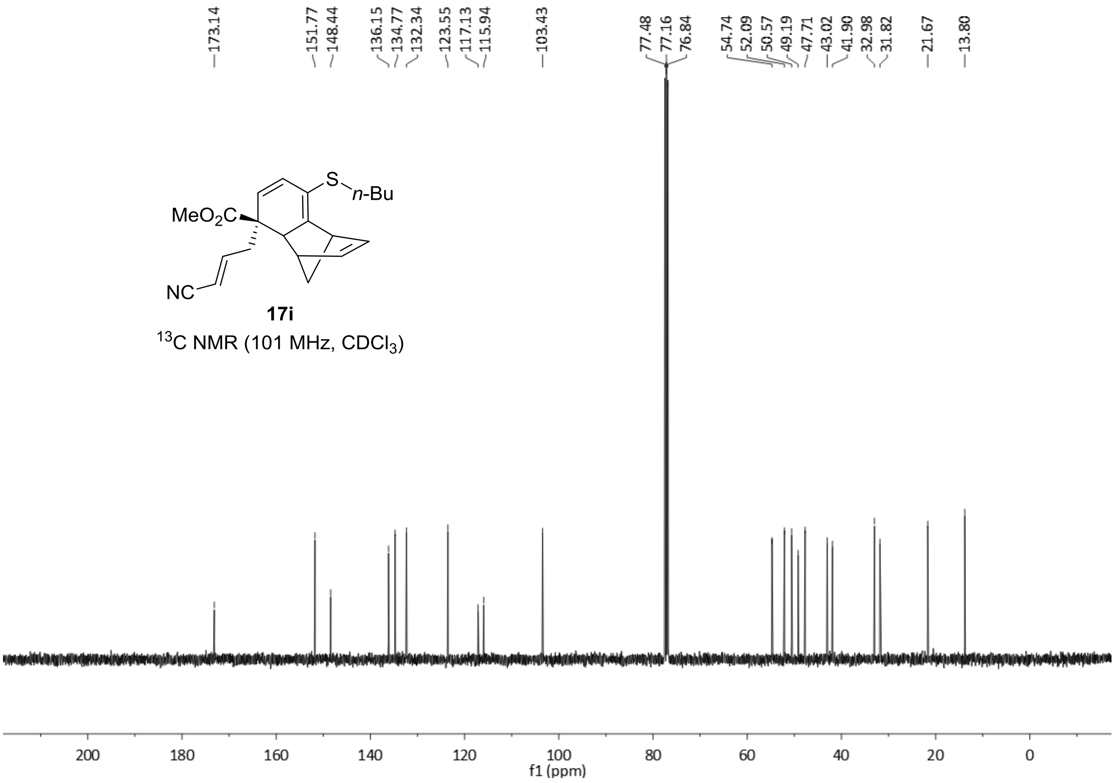

Supplementary Figure 225. ^{13}C NMR spectrum of **17i**

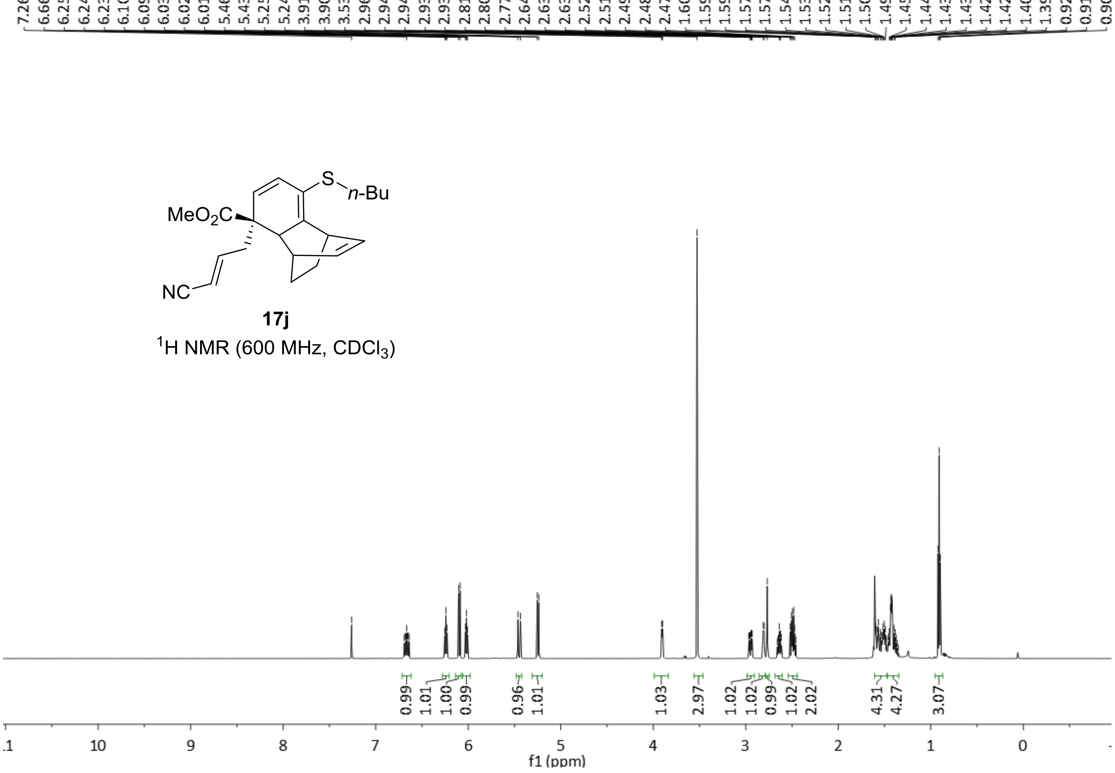

Supplementary Figure 226. ^{1}H NMR spectrum of **17j**

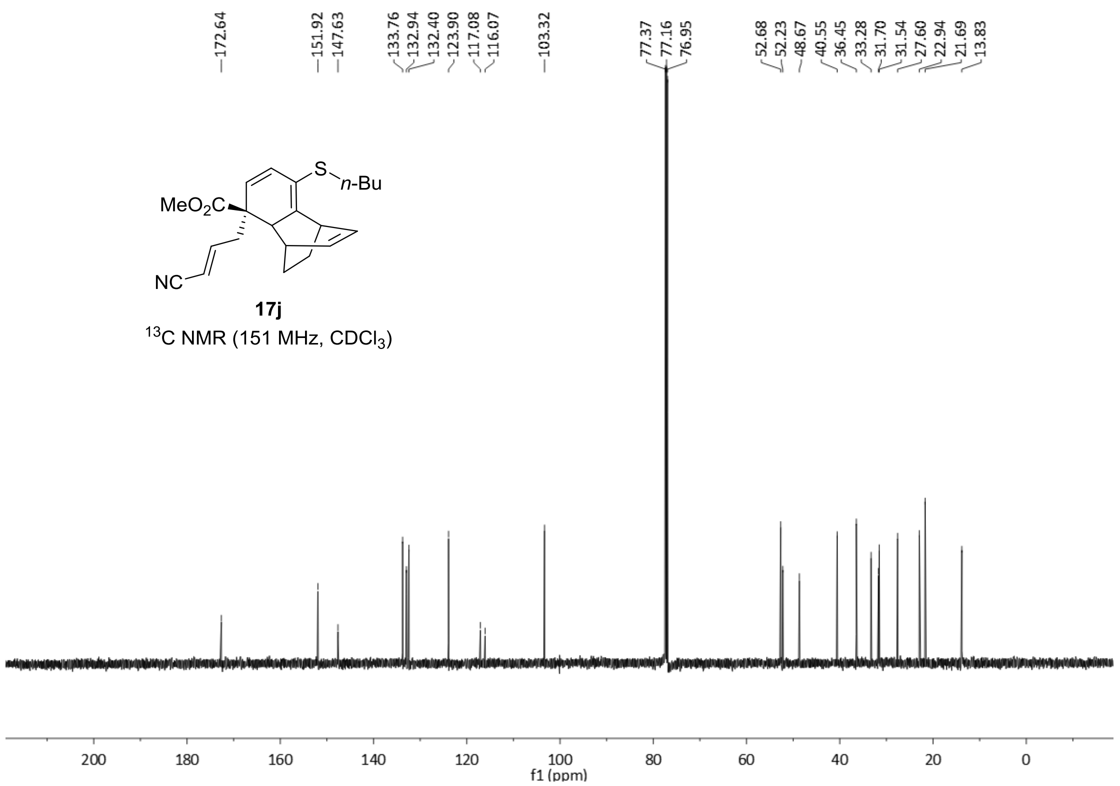

Supplementary Figure 227. ^{13}C NMR spectrum of **17j**

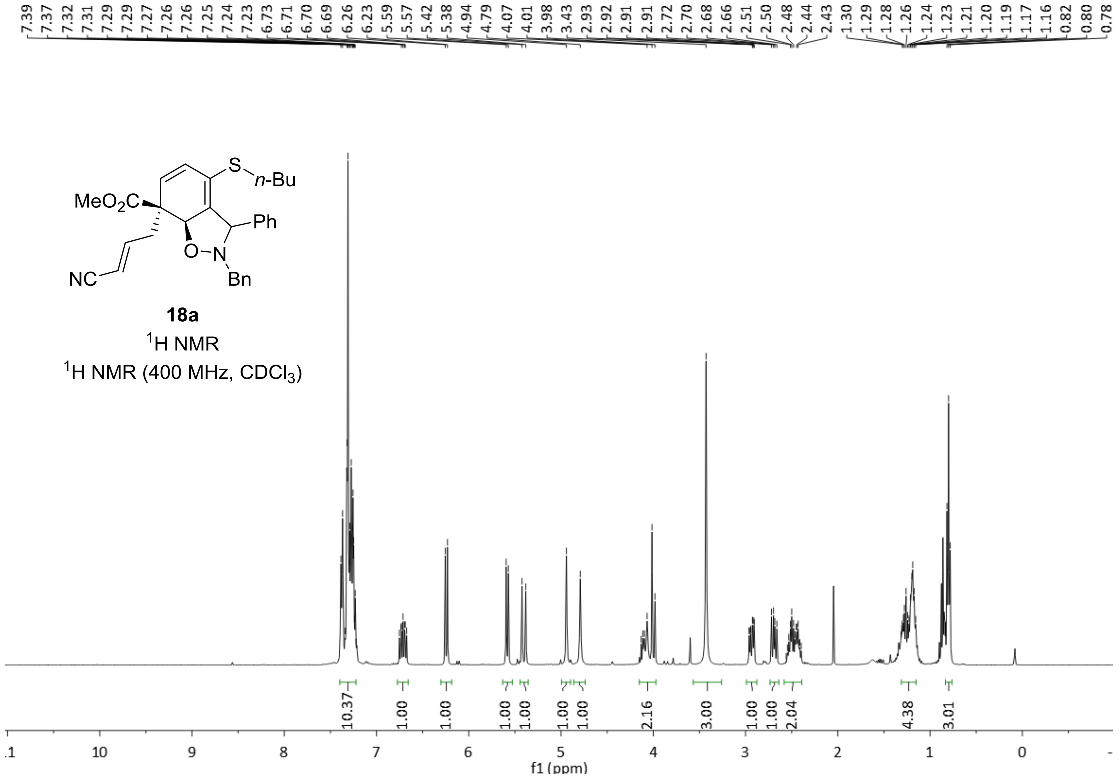

Supplementary Figure 228. ^{1}H NMR spectrum of **18a**

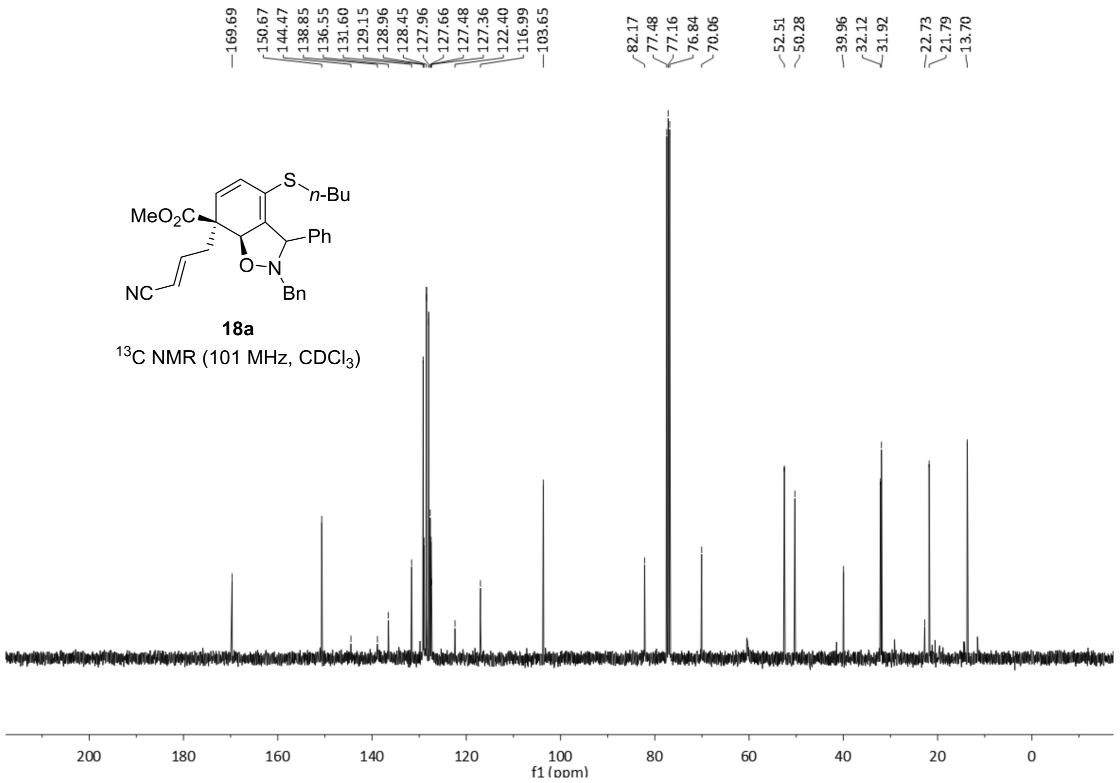

Supplementary Figure 229. ^{13}C NMR spectrum of **18a**

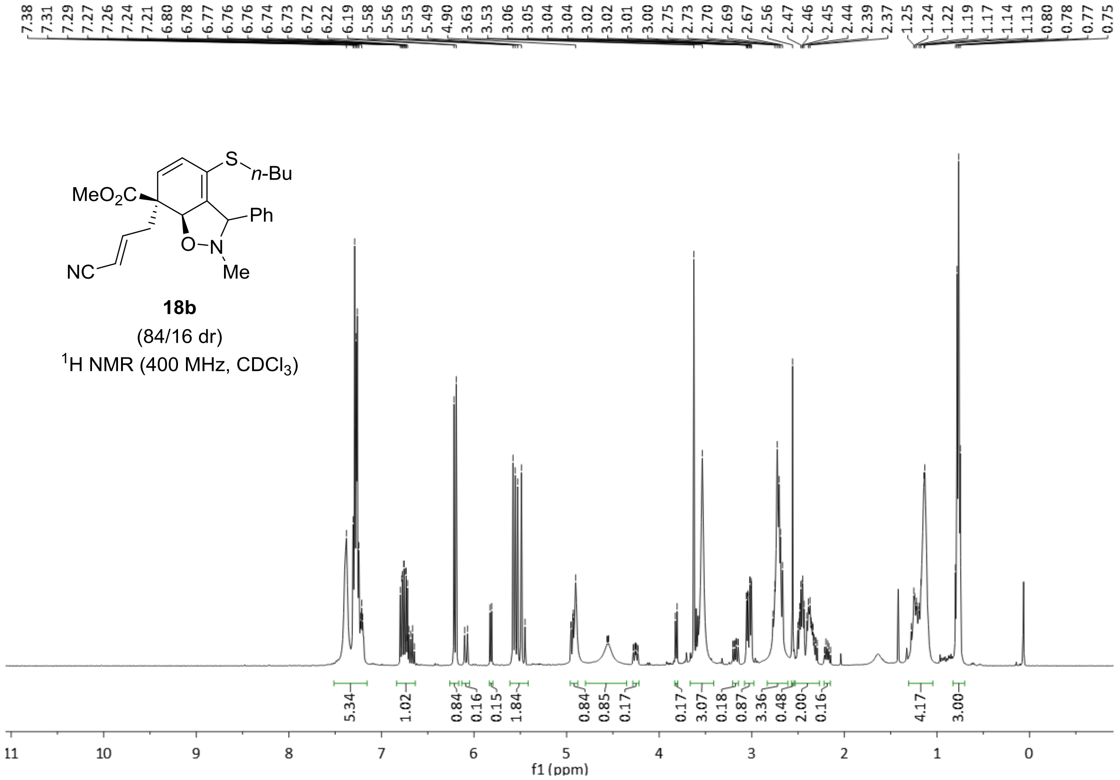

Supplementary Figure 230. ^{1}H NMR spectrum of **18b**

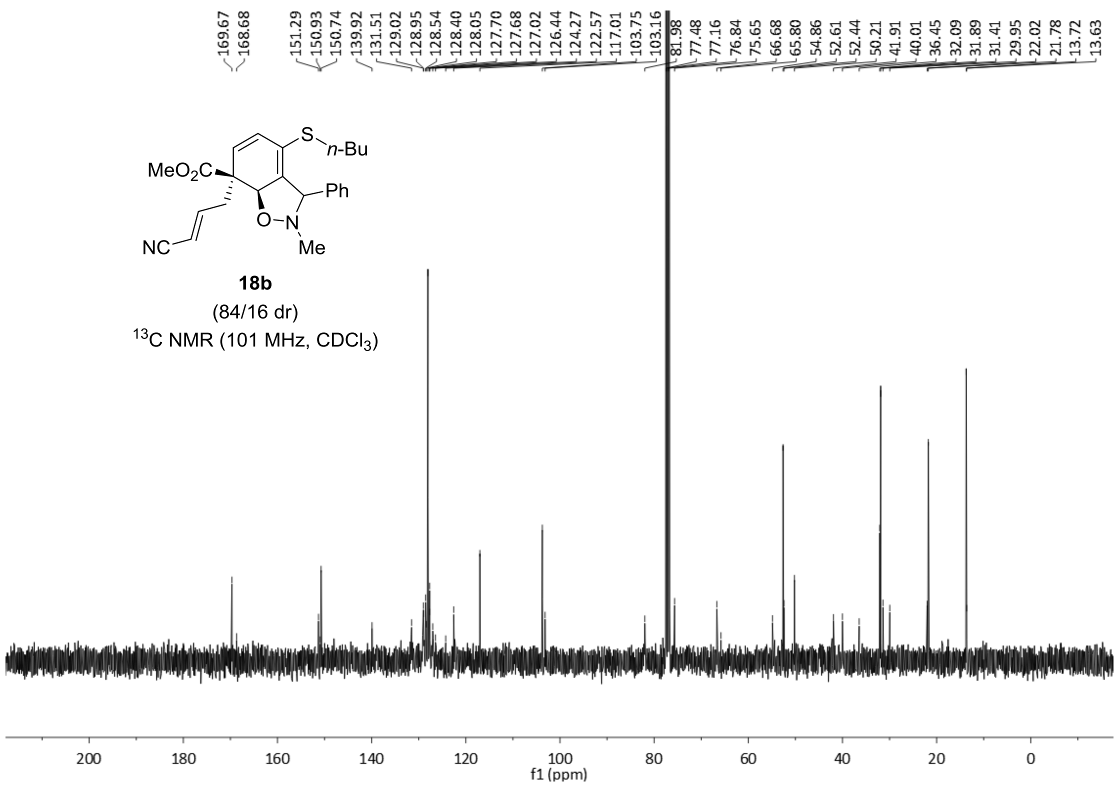

Supplementary Figure 231. ^{13}C NMR spectrum of **18b**

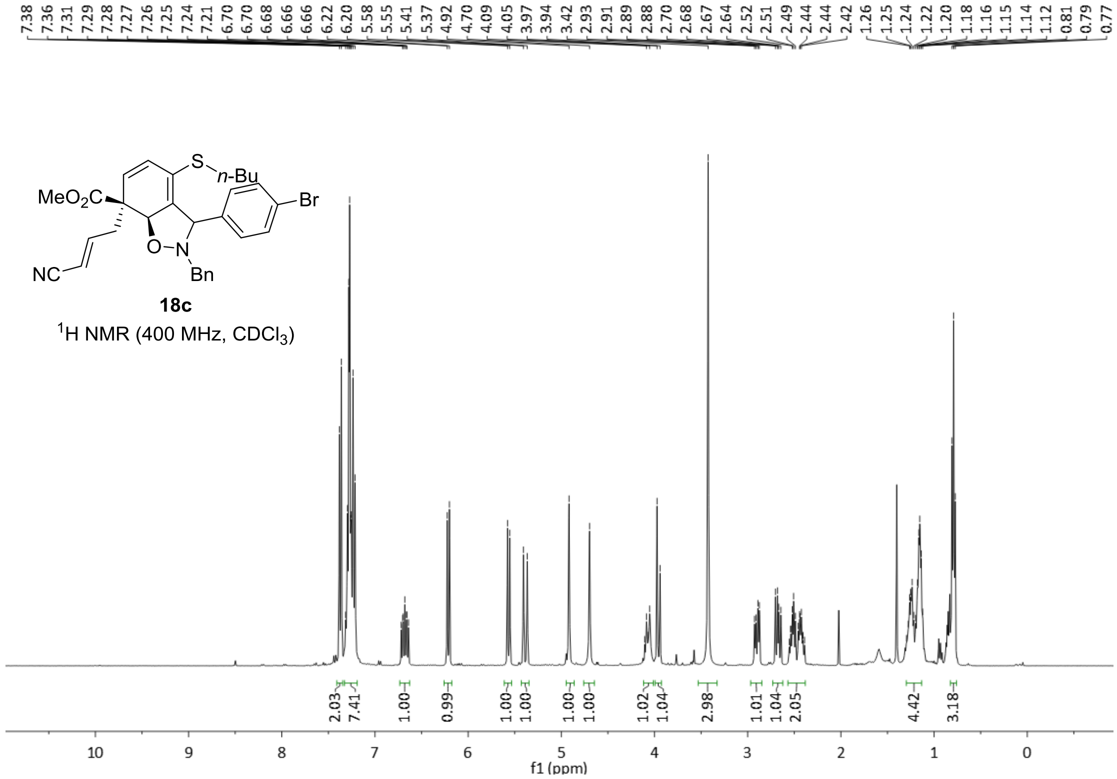

Supplementary Figure 232. ^{1}H NMR spectrum of **18c**

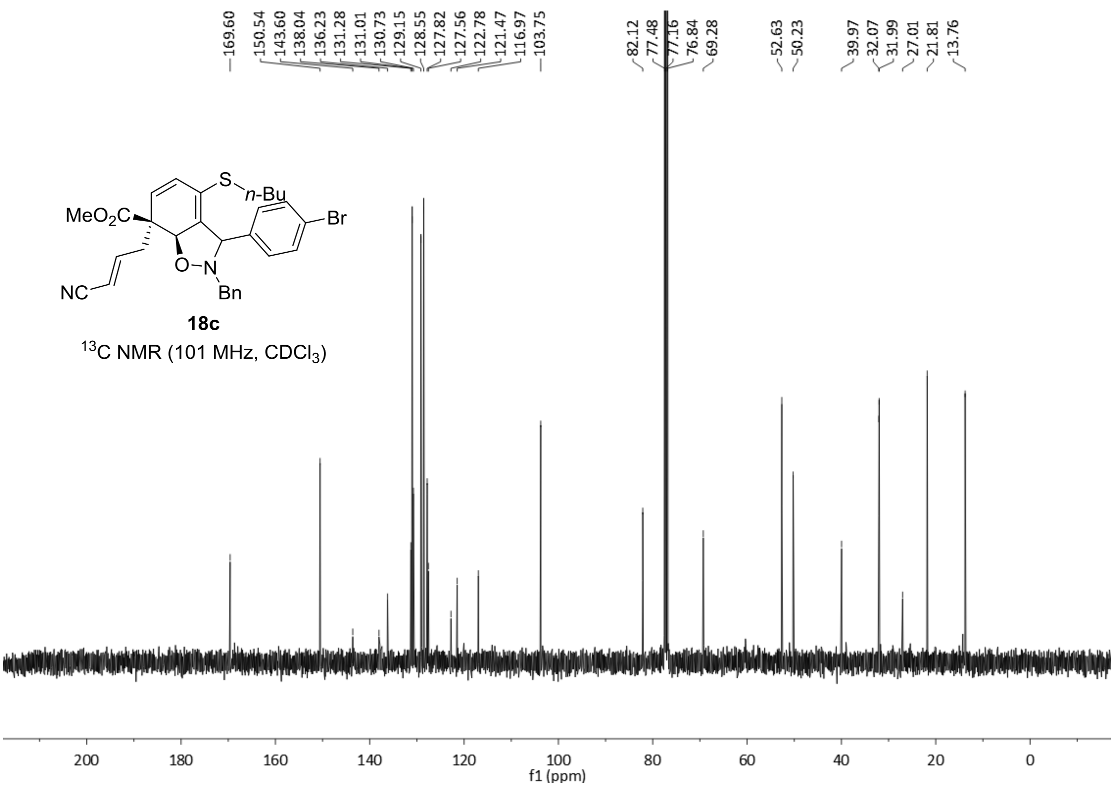

Supplementary Figure 233. ^{13}C NMR spectrum of **18c**

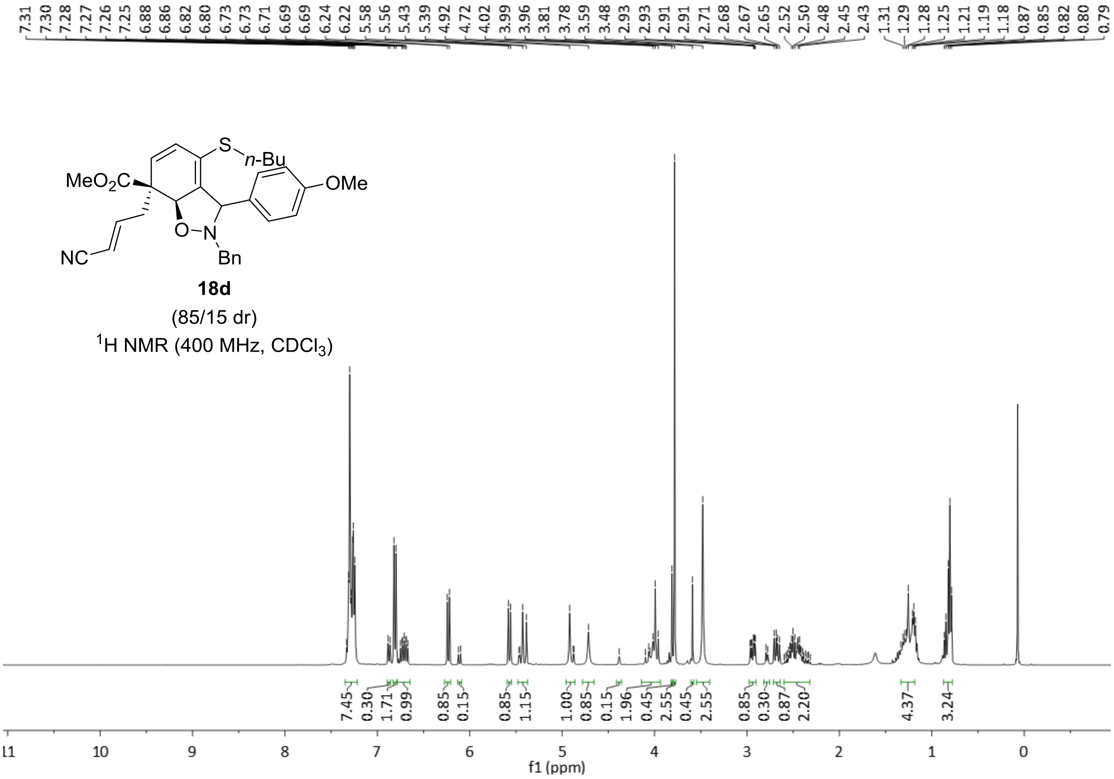

Supplementary Figure 234. ^{1}H NMR spectrum of **18d**

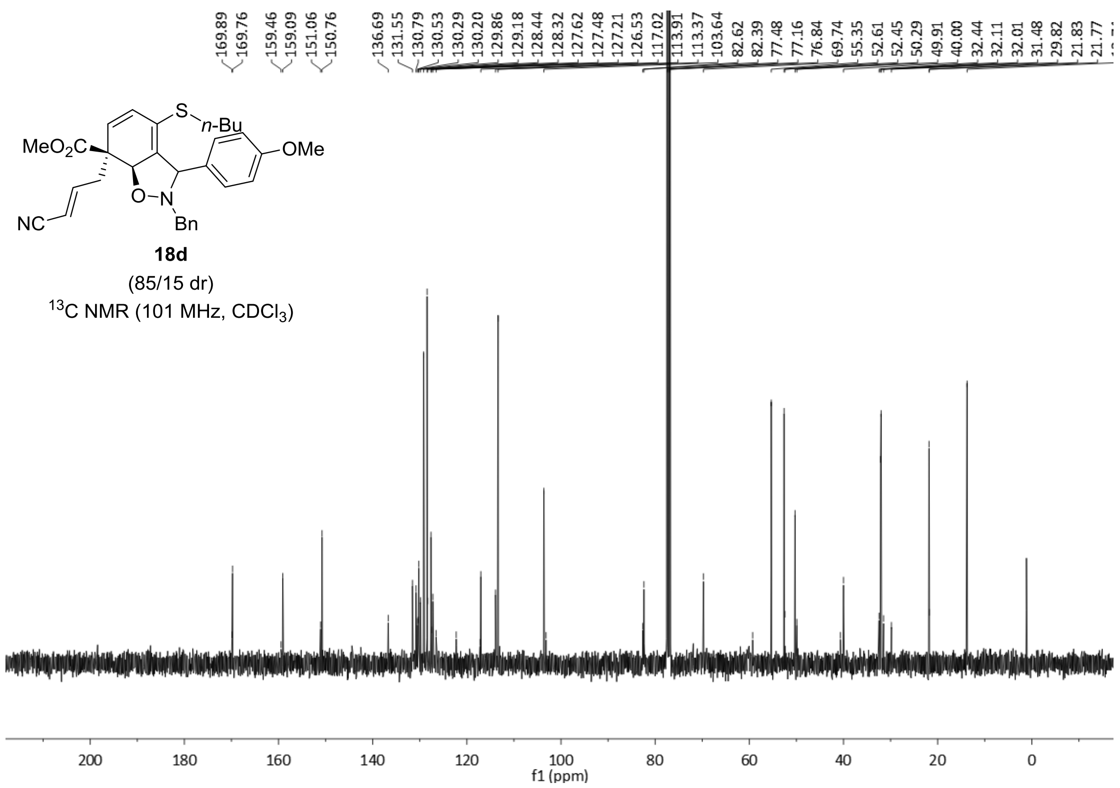

Supplementary Figure 235. ^{13}C NMR spectrum of **18d**

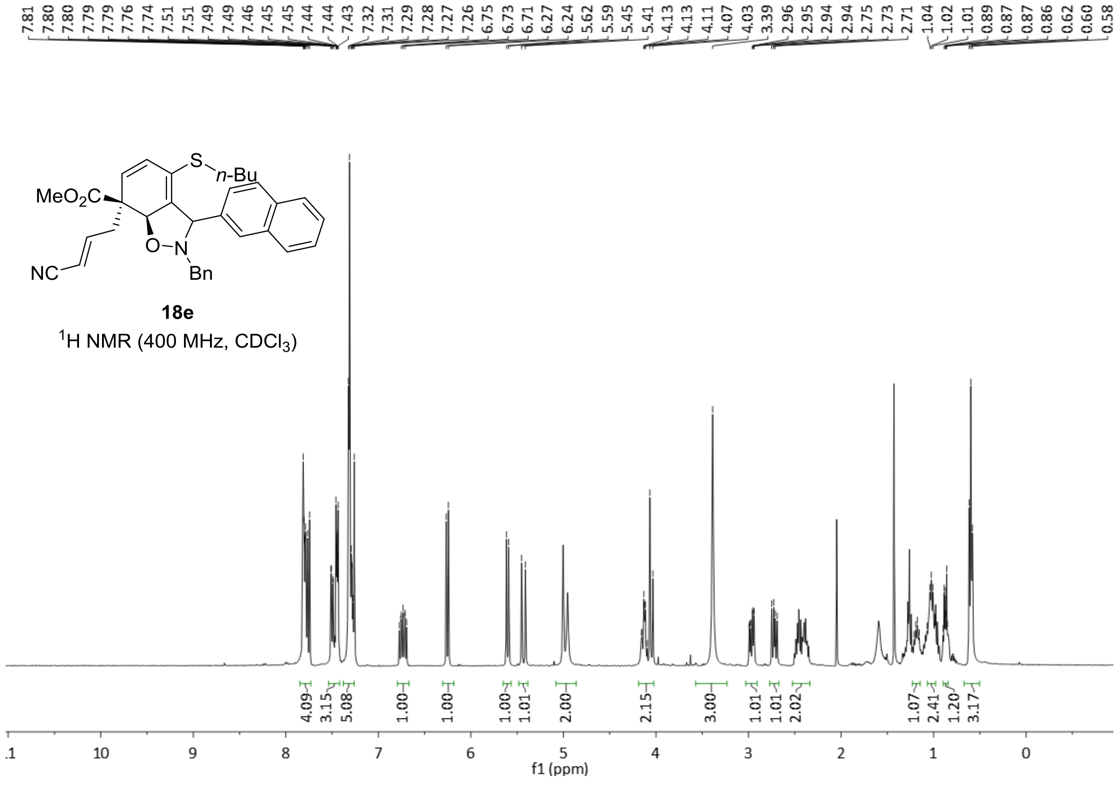

Supplementary Figure 236. ^{1}H NMR spectrum of **18e**

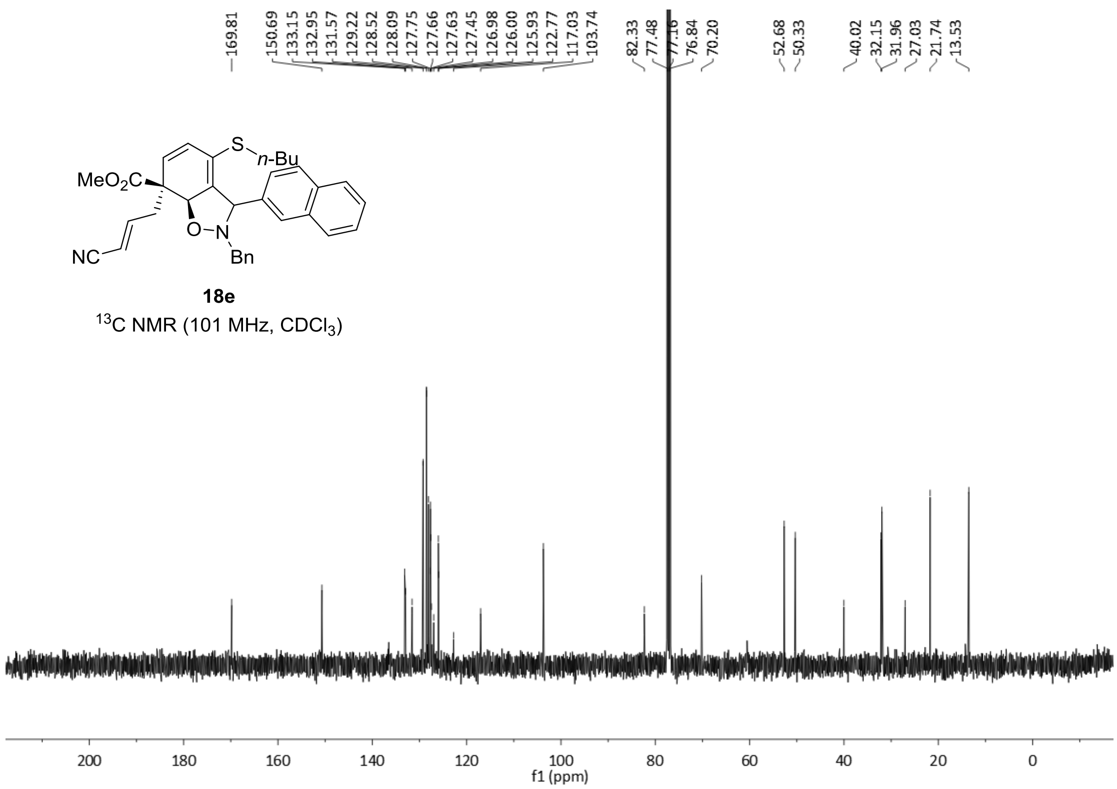

Supplementary Figure 237. ^{13}C NMR spectrum of **18e**

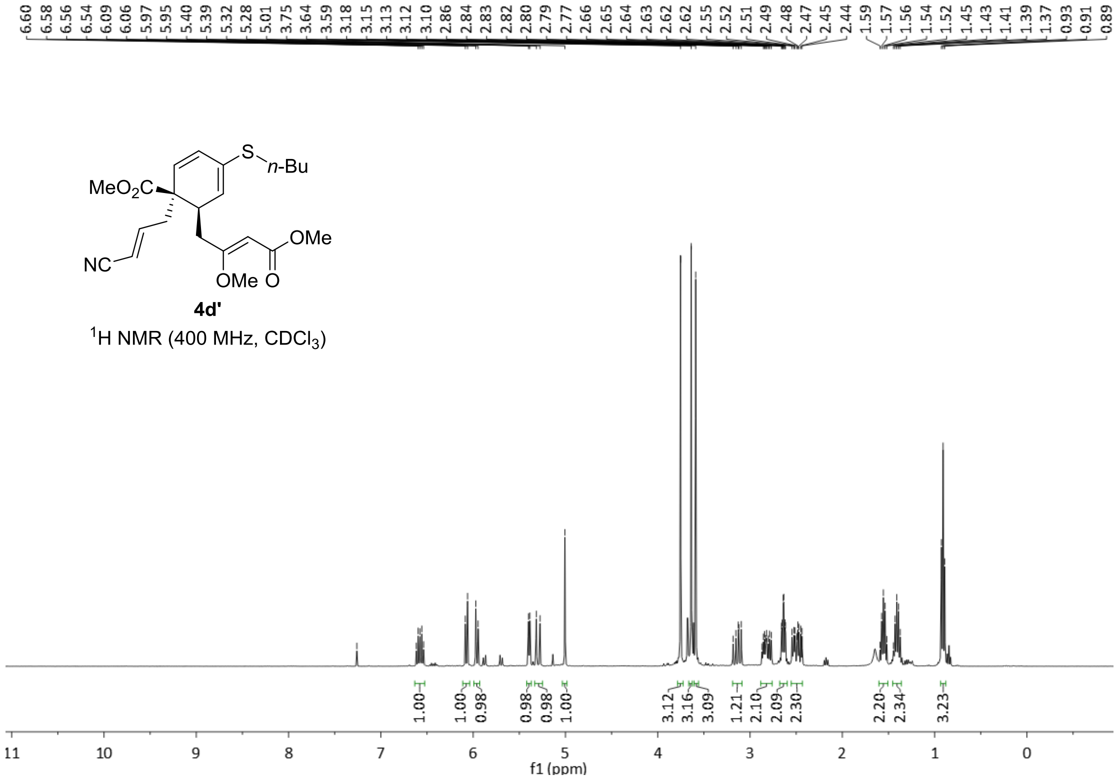

Supplementary Figure 238. ^{1}H NMR spectrum of **4d’**

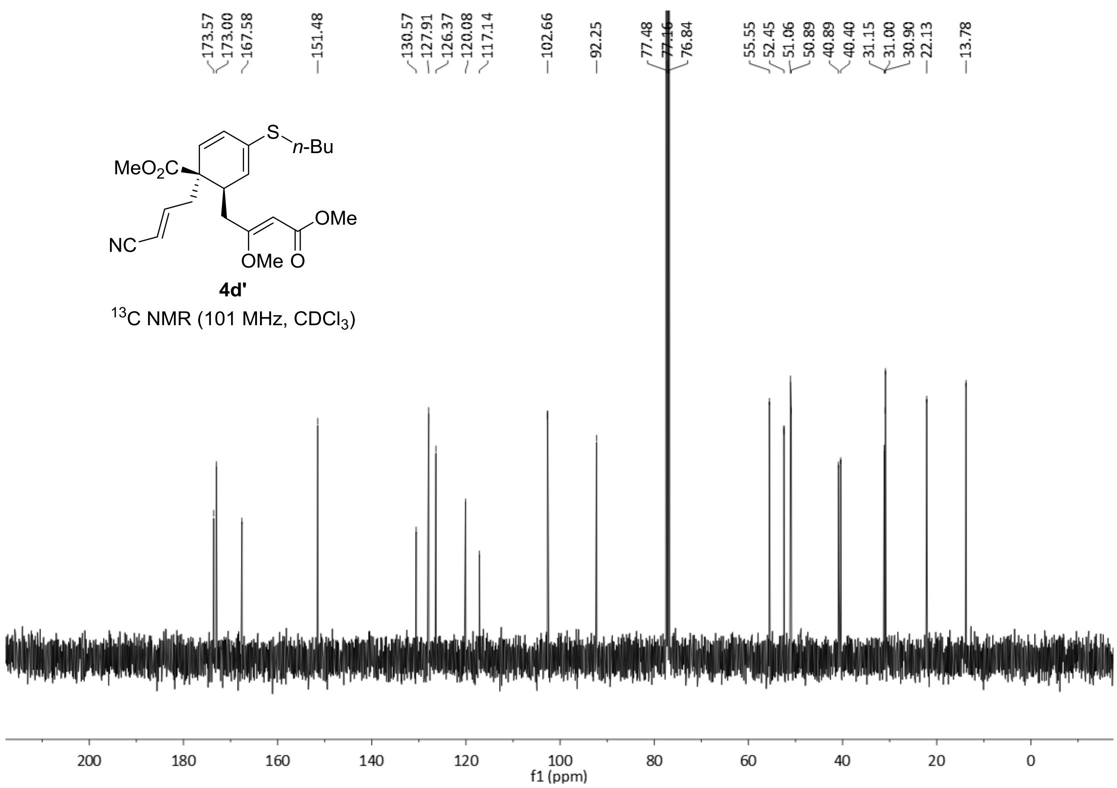

Supplementary Figure 239. ^{13}C NMR spectrum of **4d'**

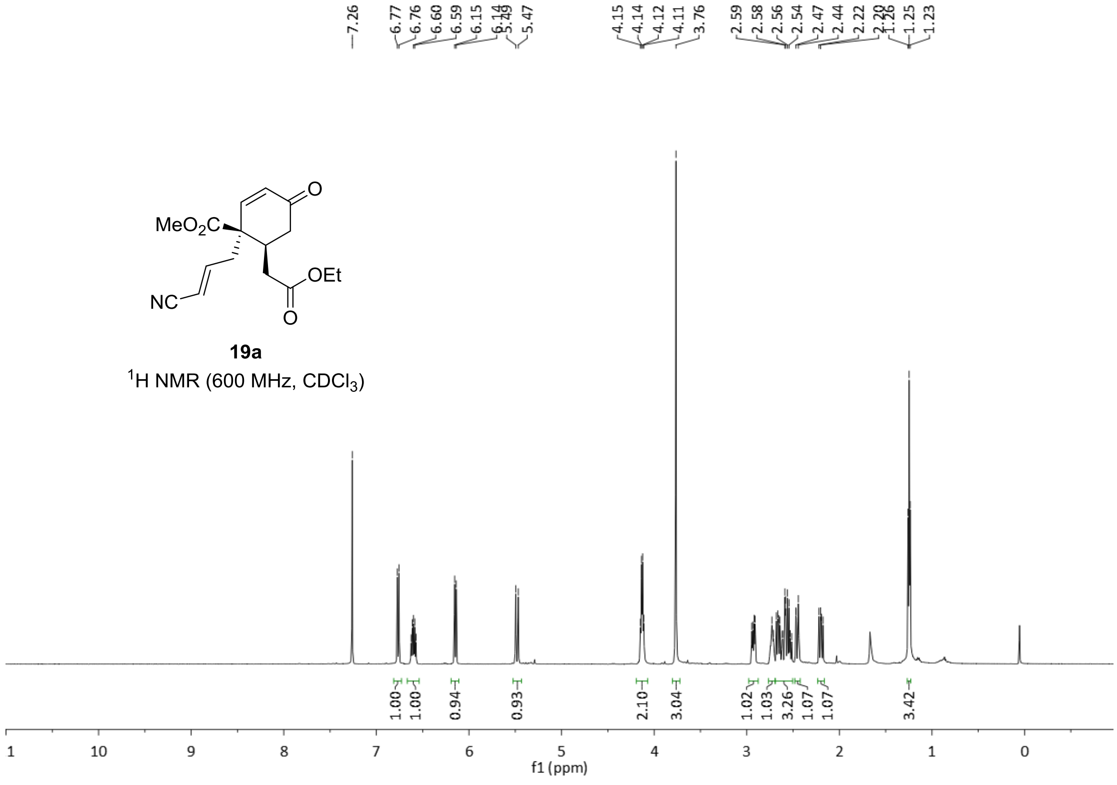

Supplementary Figure 240. ^{1}H NMR spectrum of **19a**

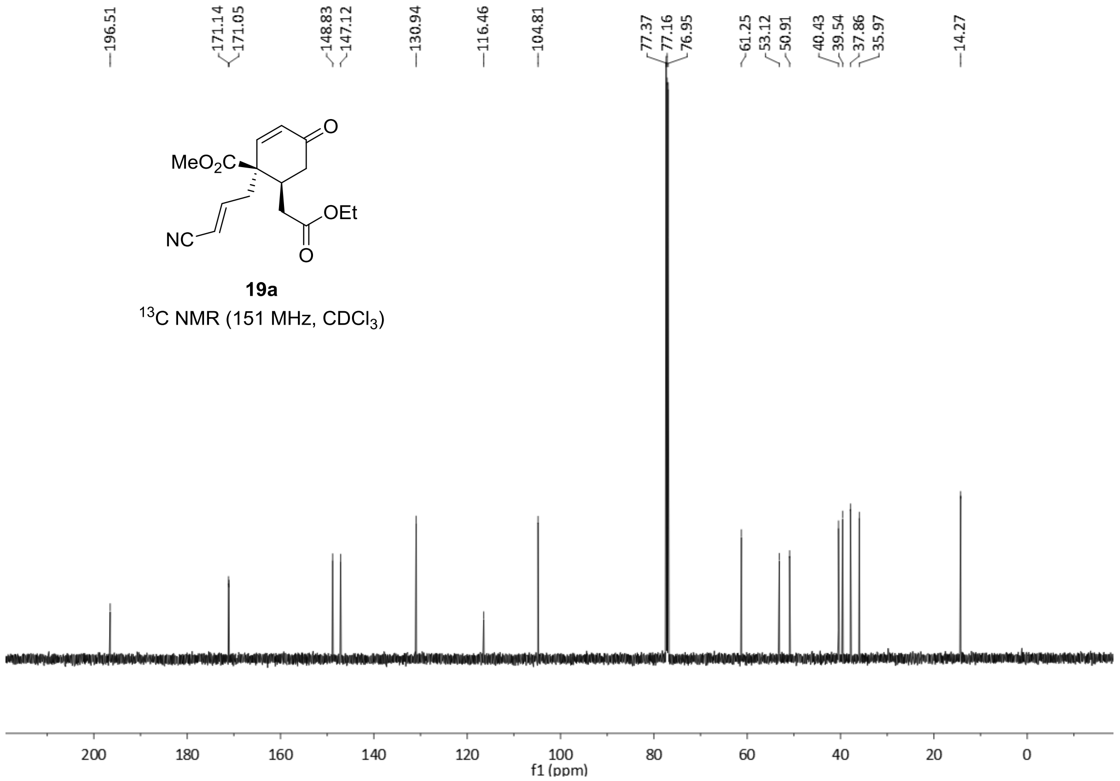

Supplementary Figure 241. ^{13}C NMR spectrum of **19a**

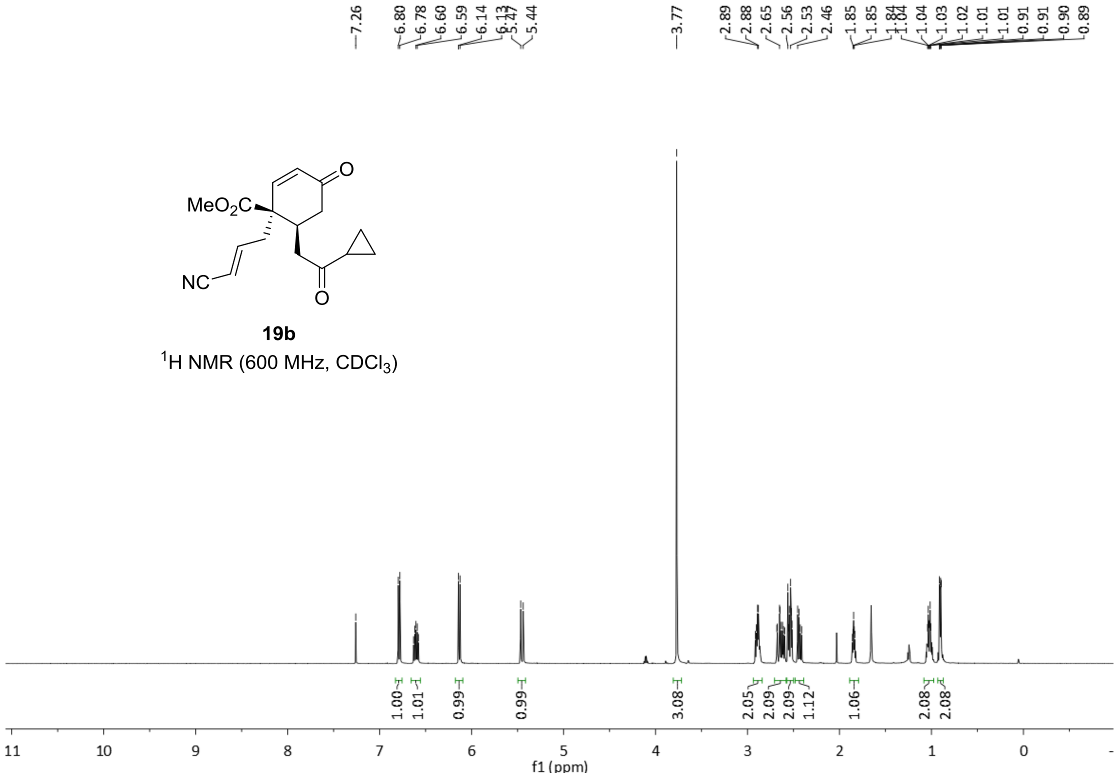

Supplementary Figure 242. ^{1}H NMR spectrum of **19b**

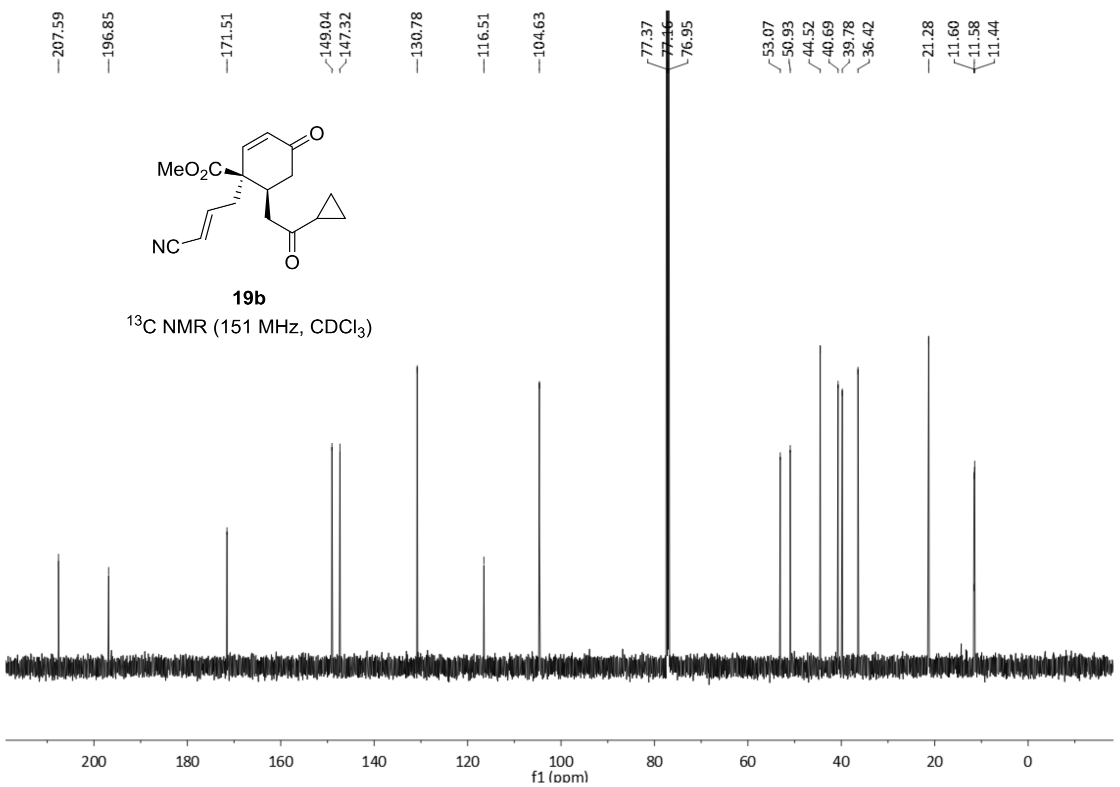

Supplementary Figure 243. ^{13}C NMR spectrum of **19b**

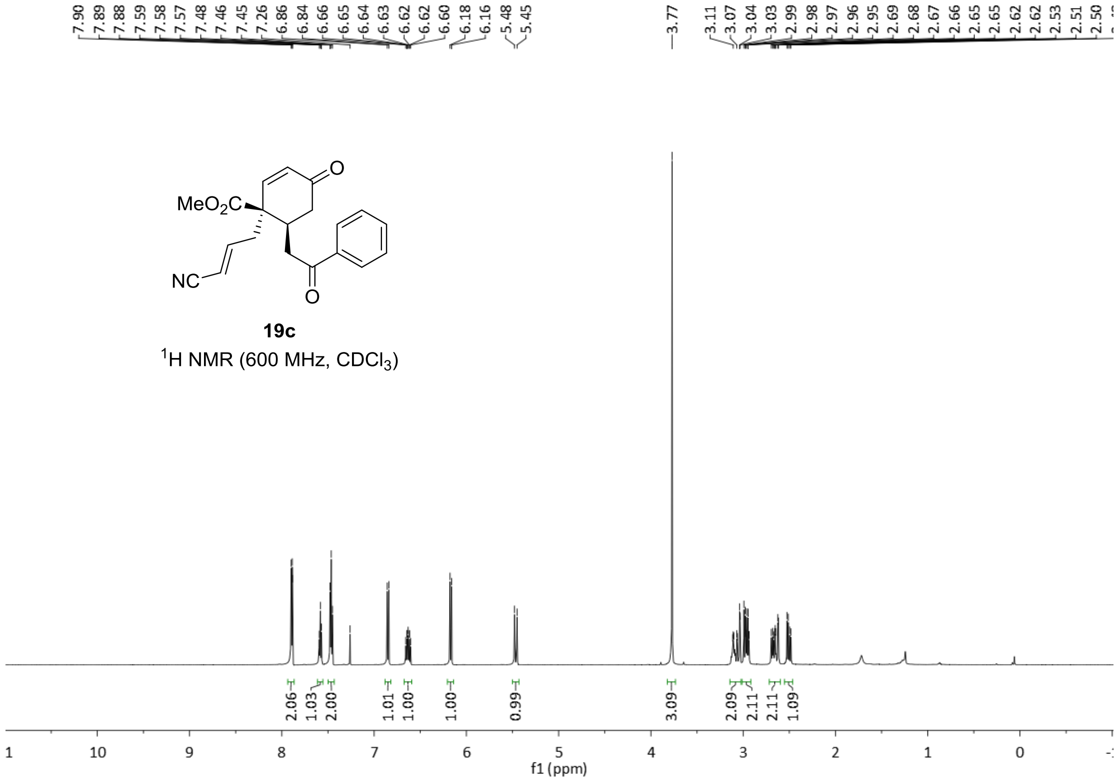

Supplementary Figure 244. ^{1}H NMR spectrum of **19c**

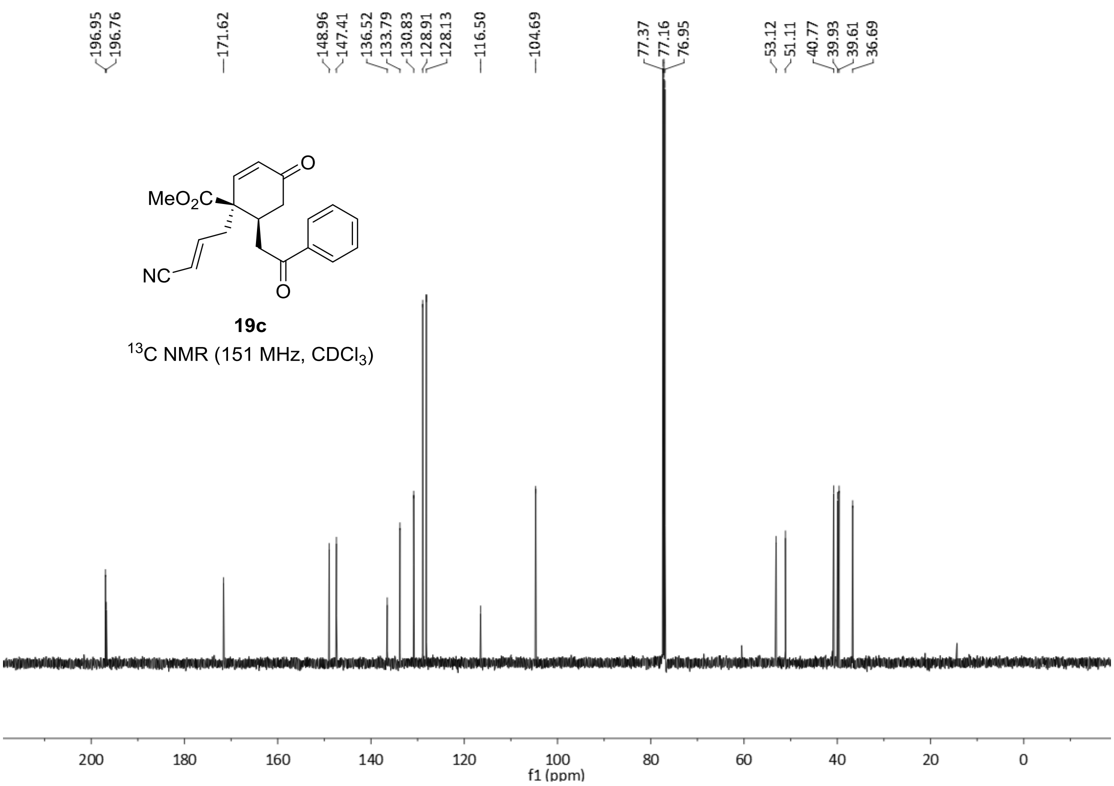

Supplementary Figure 245. ^{13}C NMR spectrum of **19c**

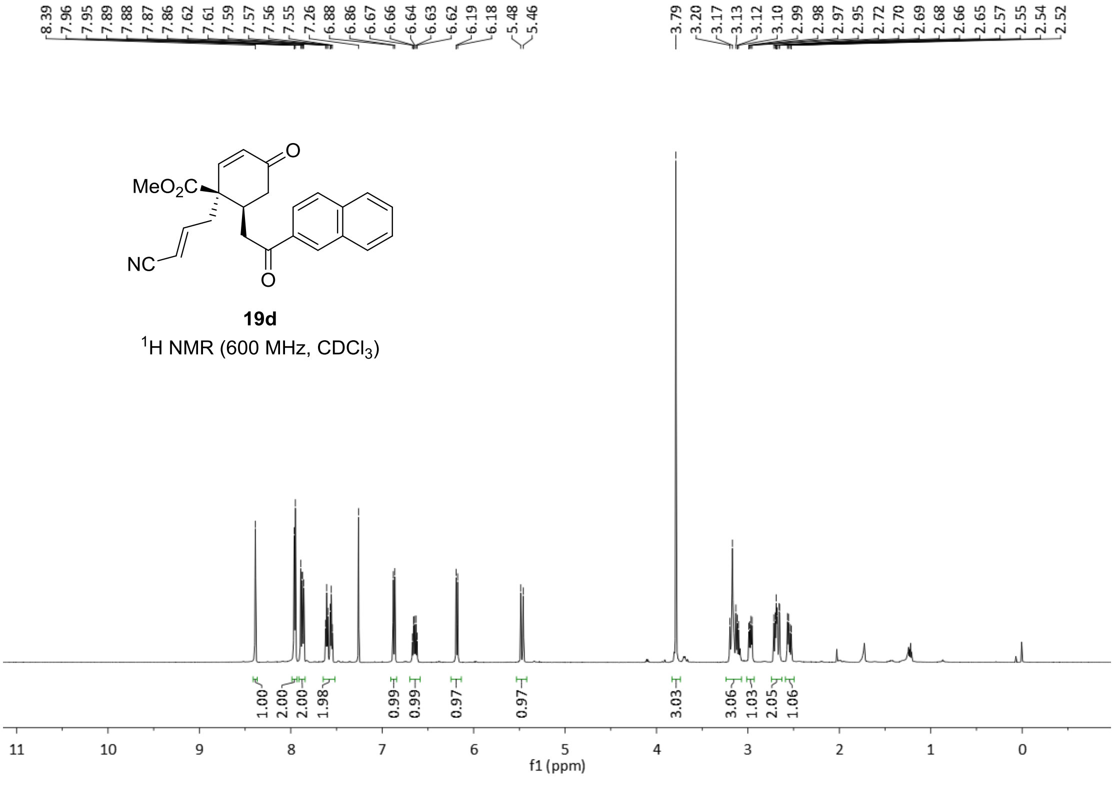

Supplementary Figure 246. ^{1}H NMR spectrum of **19d**

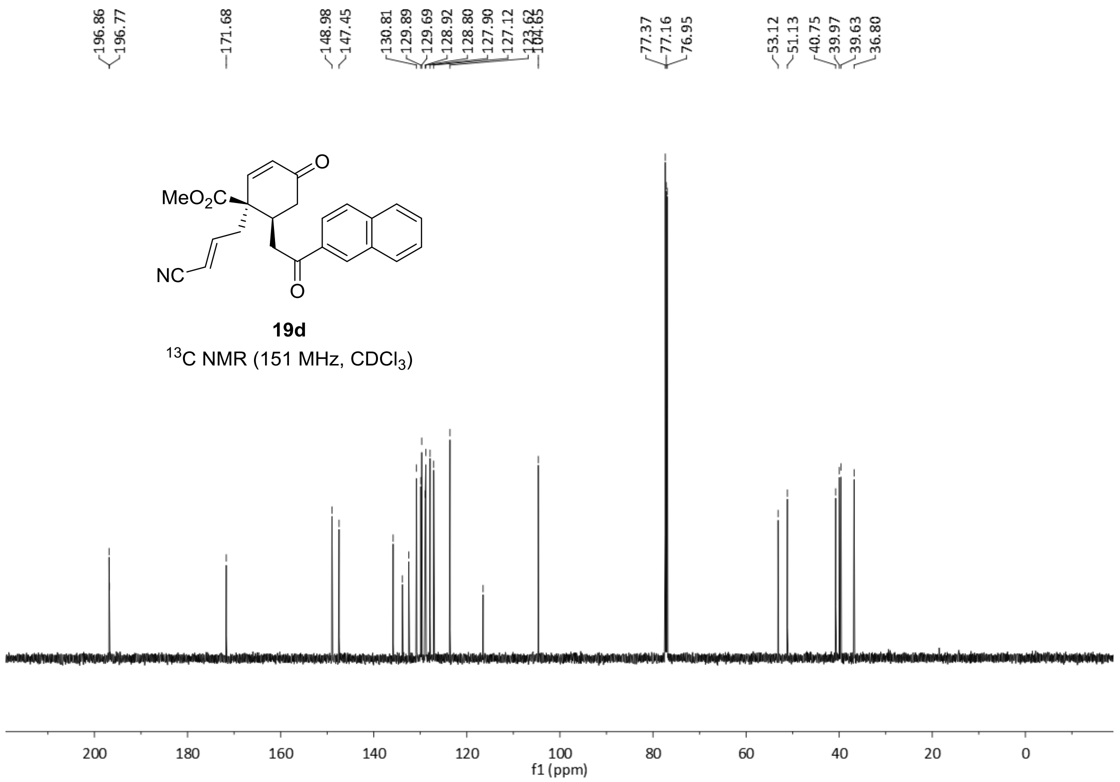

Supplementary Figure 247. ^{13}C NMR spectrum of **19d**

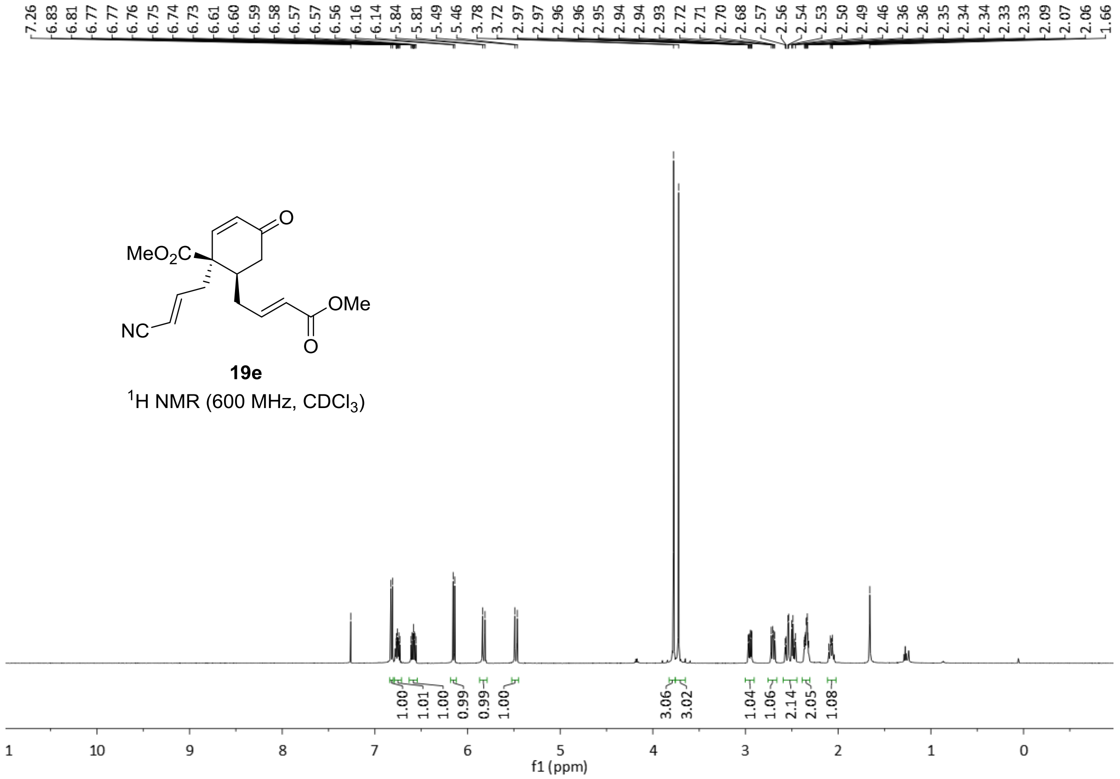

Supplementary Figure 248. ^{1}H NMR spectrum of **19e**

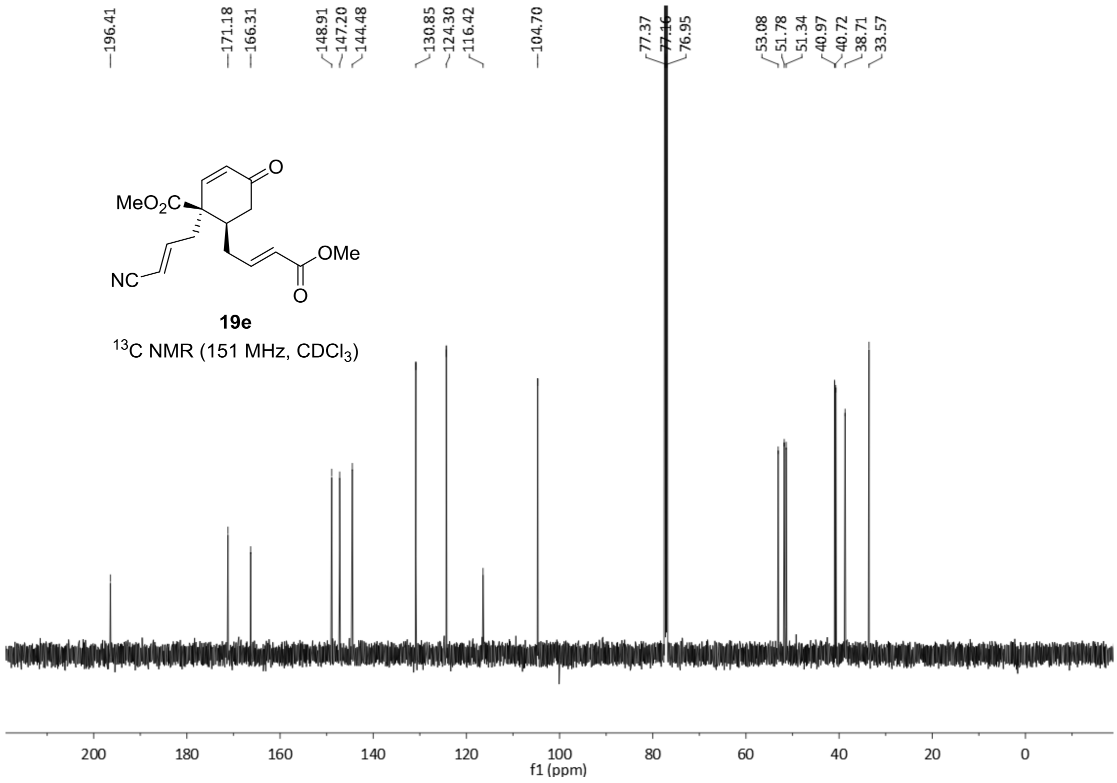

Supplementary Figure 249. ^{13}C NMR spectrum of **19e**

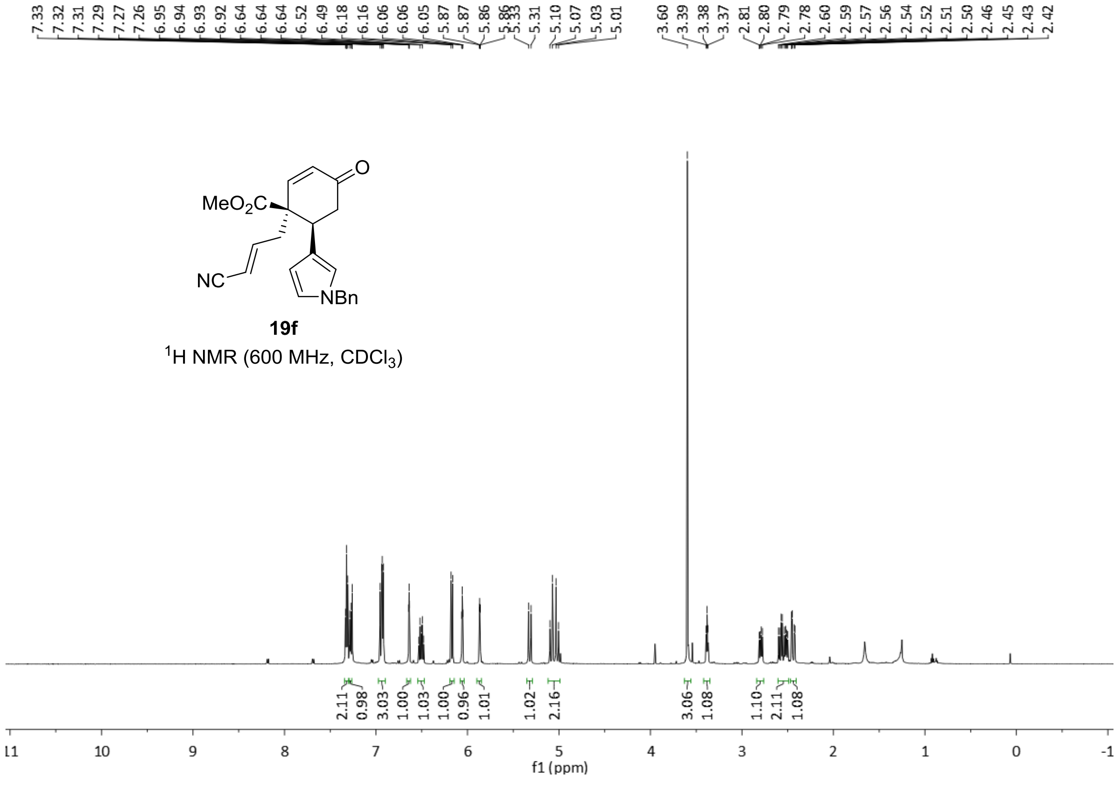

Supplementary Figure 250. ^{1}H NMR spectrum of **19f**

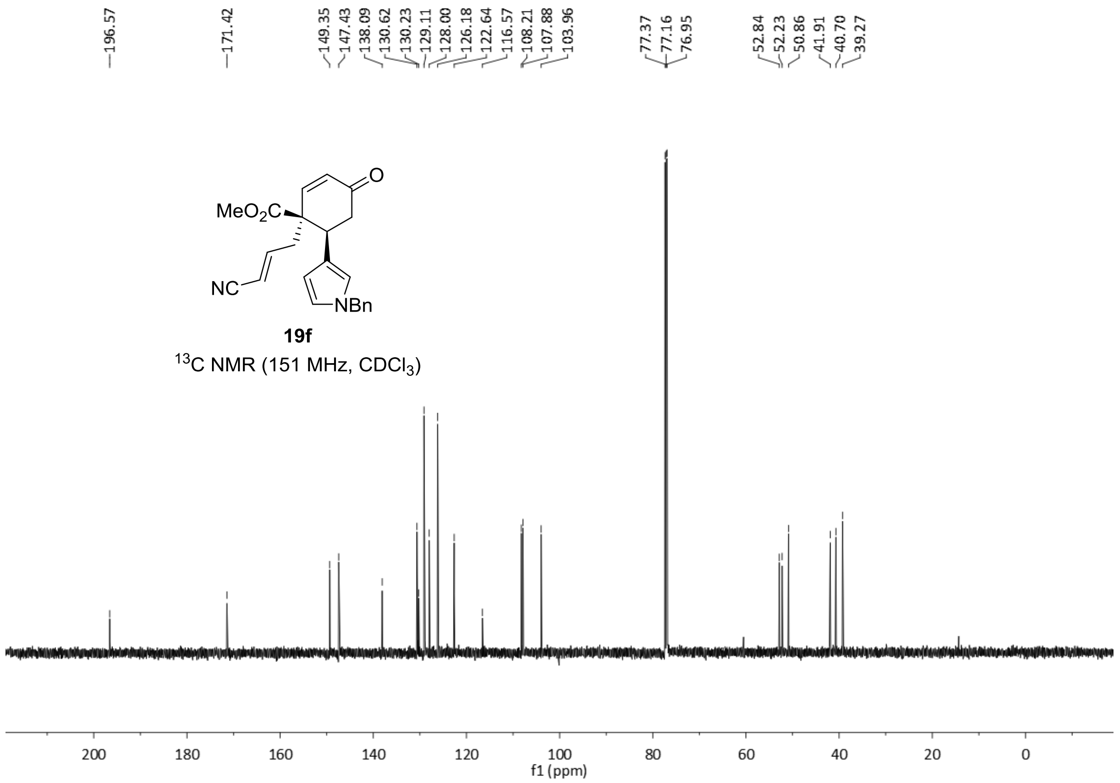

Supplementary Figure 251. ^{13}C NMR spectrum of **19f**

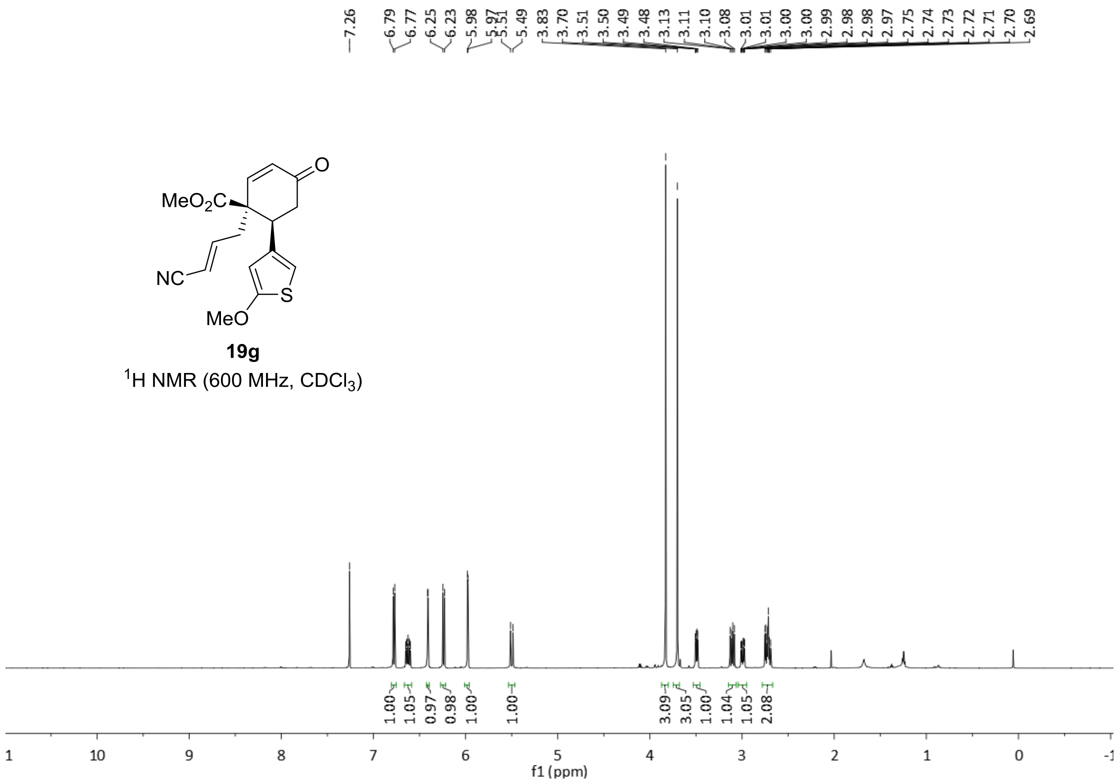

Supplementary Figure 252. ^{1}H NMR spectrum of **19g**

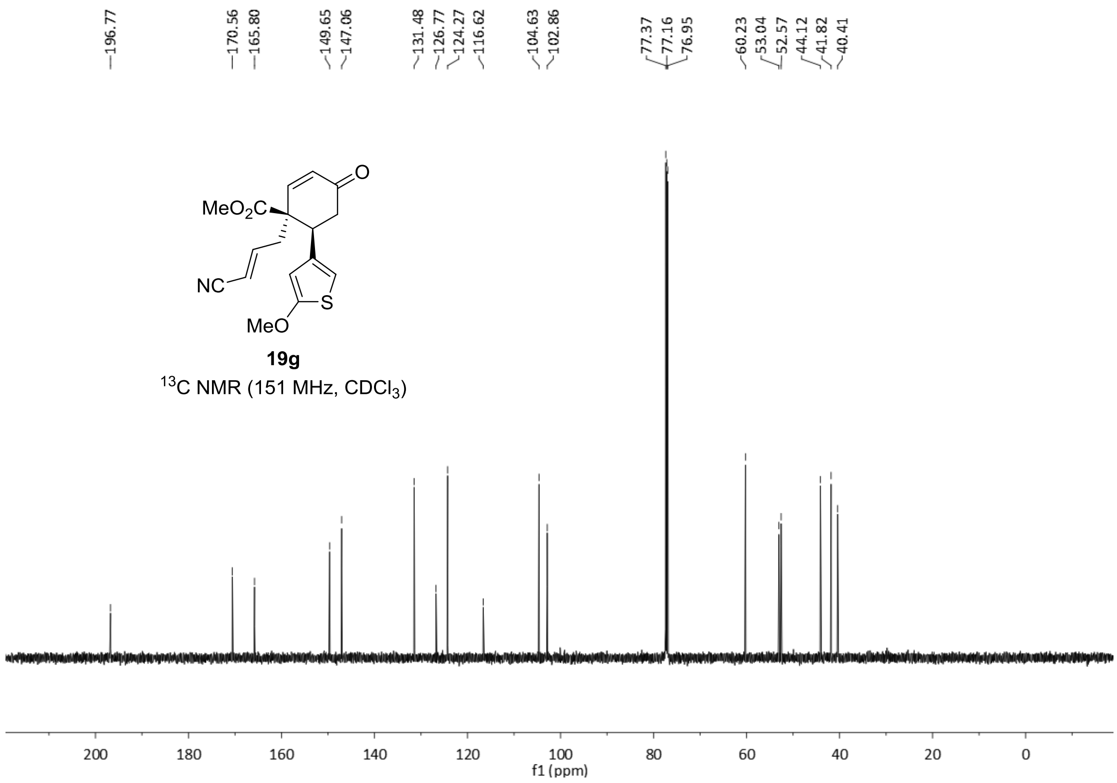

Supplementary Figure 253. ^{13}C NMR spectrum of **19g**

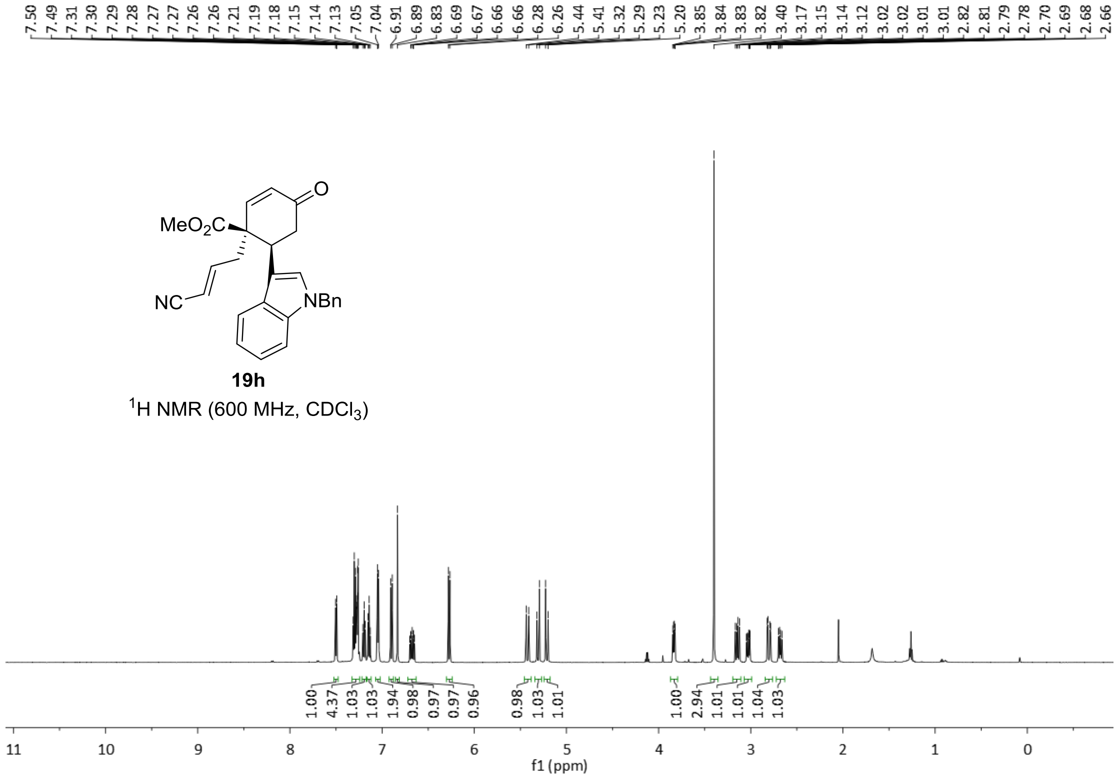

Supplementary Figure 254. ^{1}H NMR spectrum of **19h**

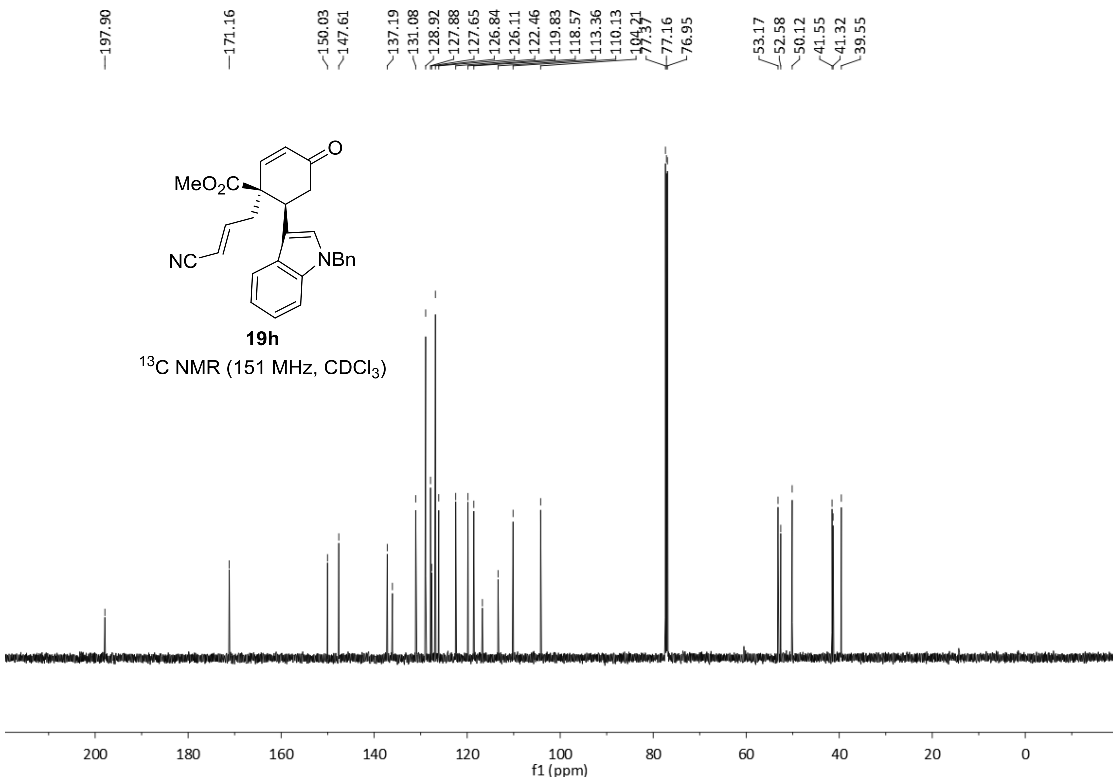

Supplementary Figure 255. ^{13}C NMR spectrum of **19h**

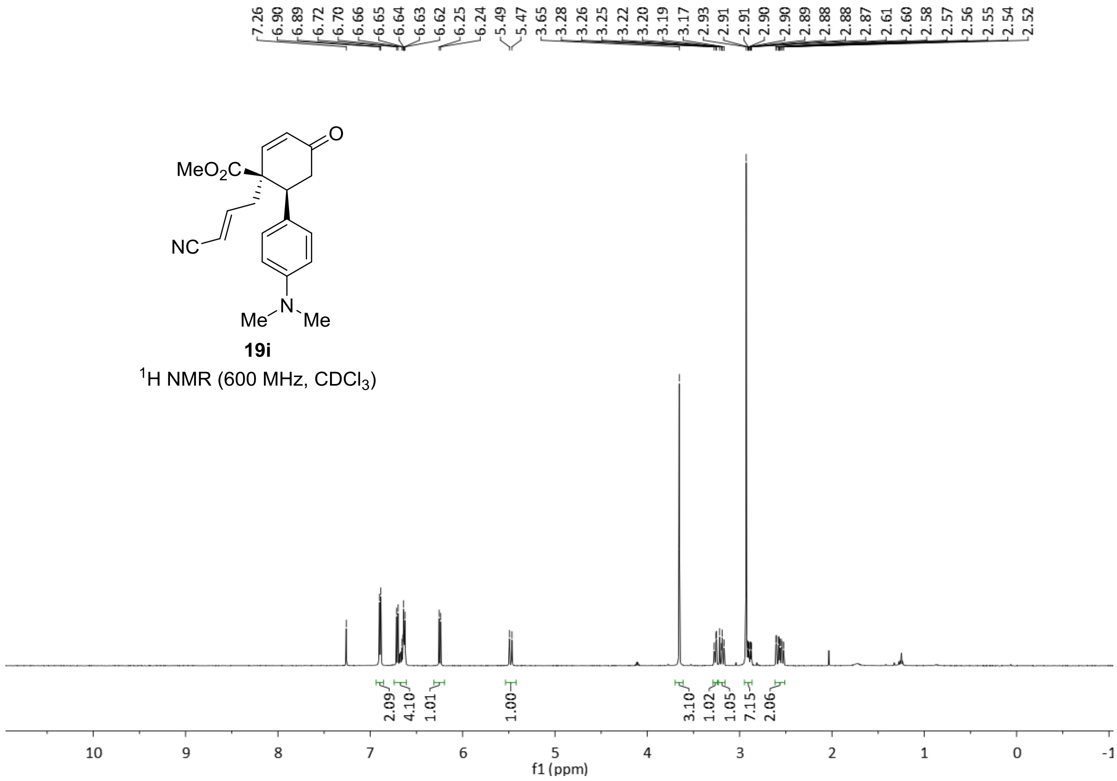

Supplementary Figure 256. ^{1}H NMR spectrum of **19i**

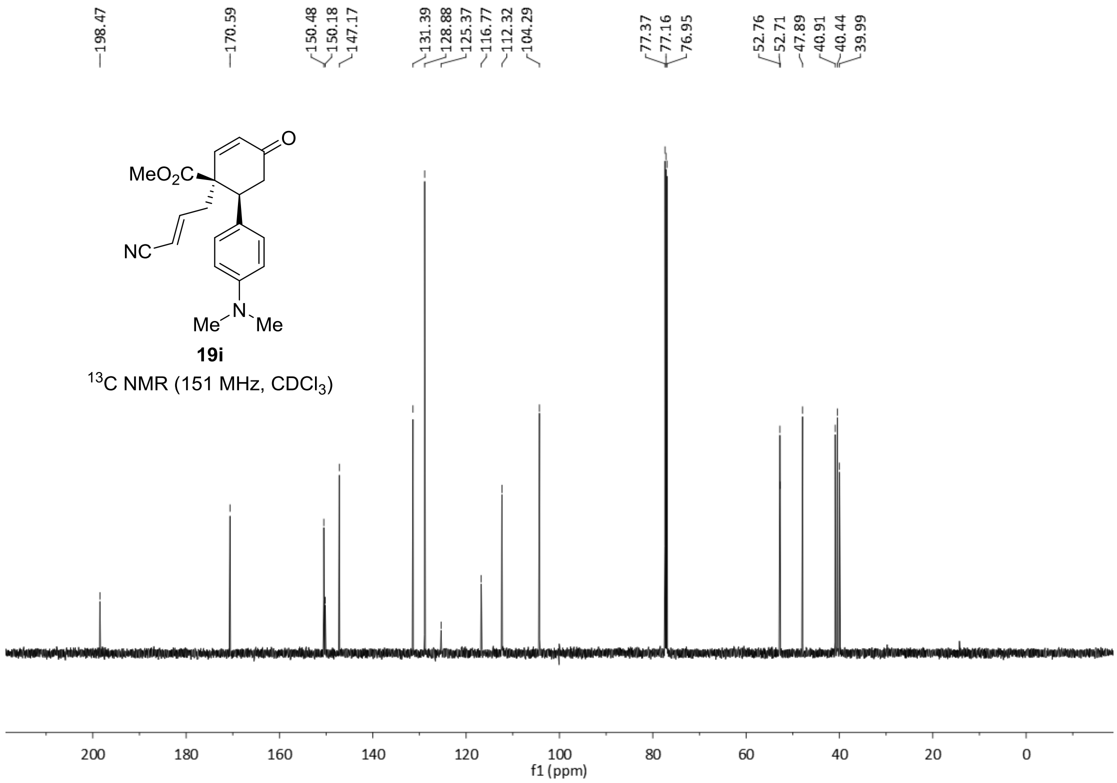

Supplementary Figure 257. ^{13}C NMR spectrum of **19i**

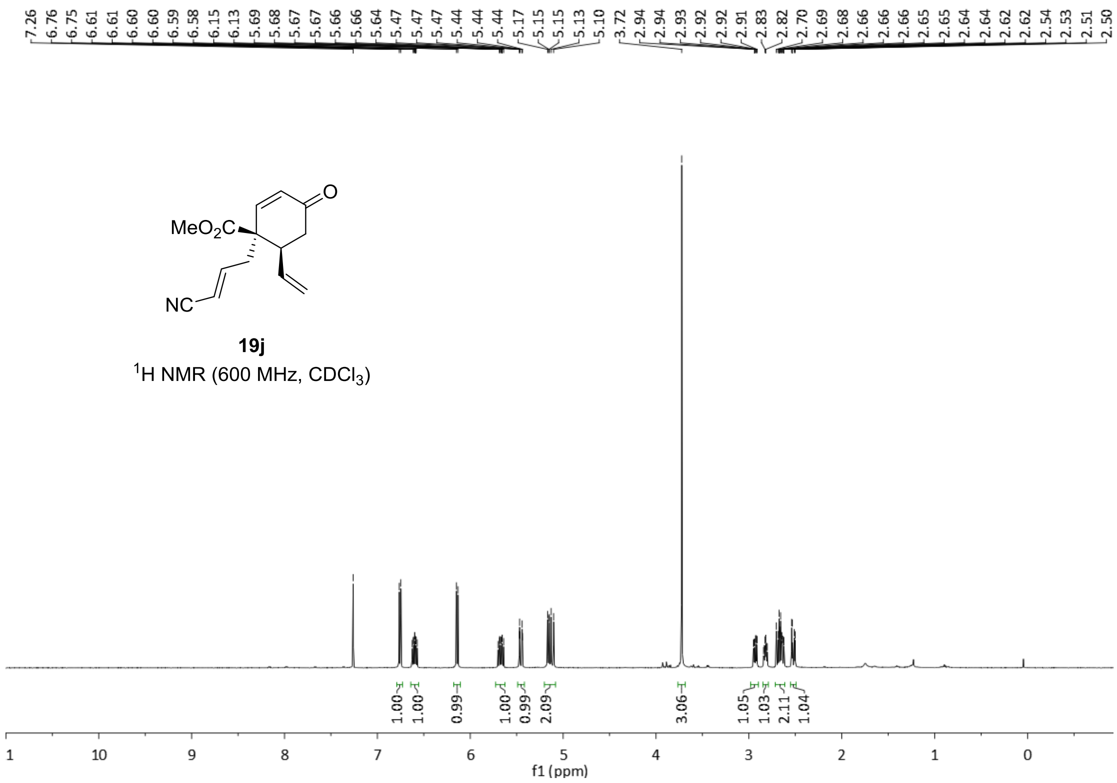

Supplementary Figure 258. ^{1}H NMR spectrum of **19j**

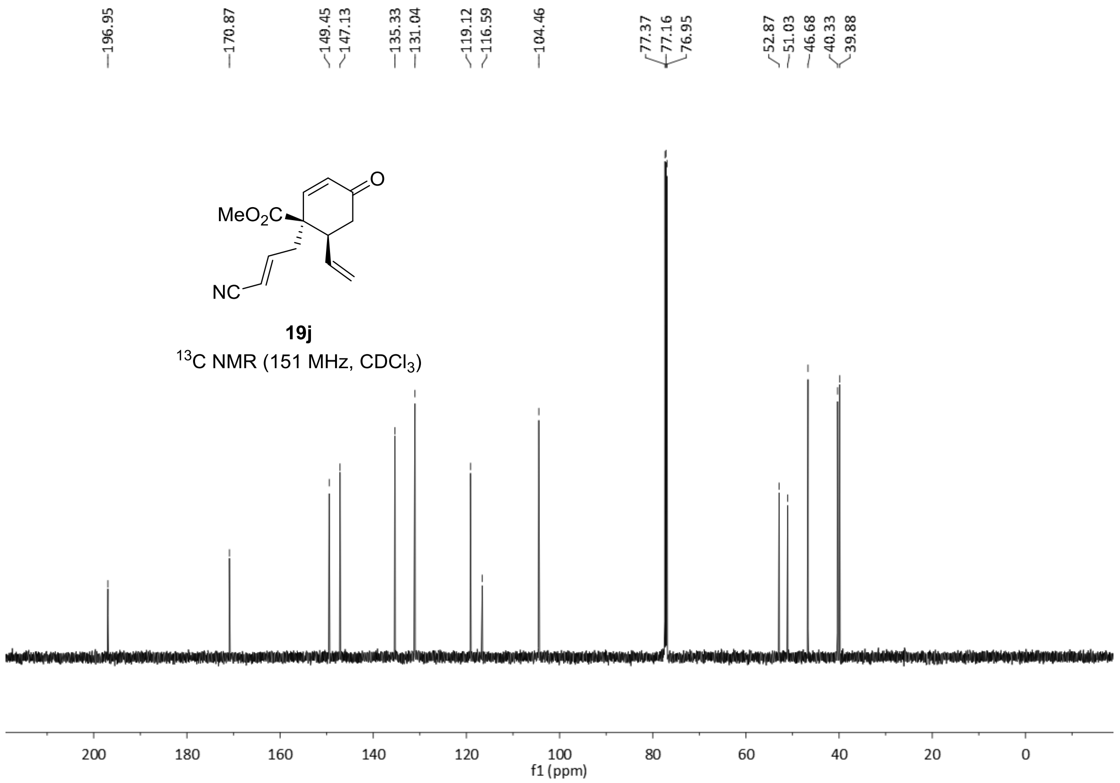

Supplementary Figure 259. ^{13}C NMR spectrum of **19j**

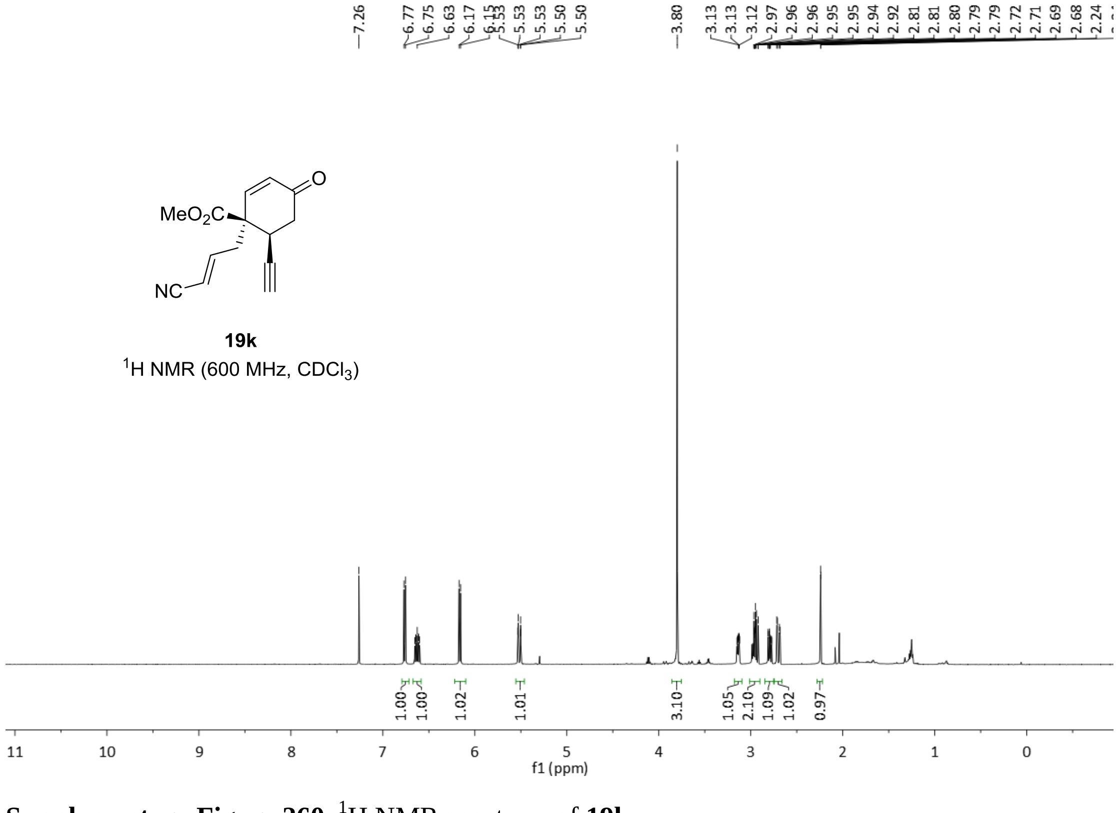

Supplementary Figure 260. ^{1}H NMR spectrum of **19k**

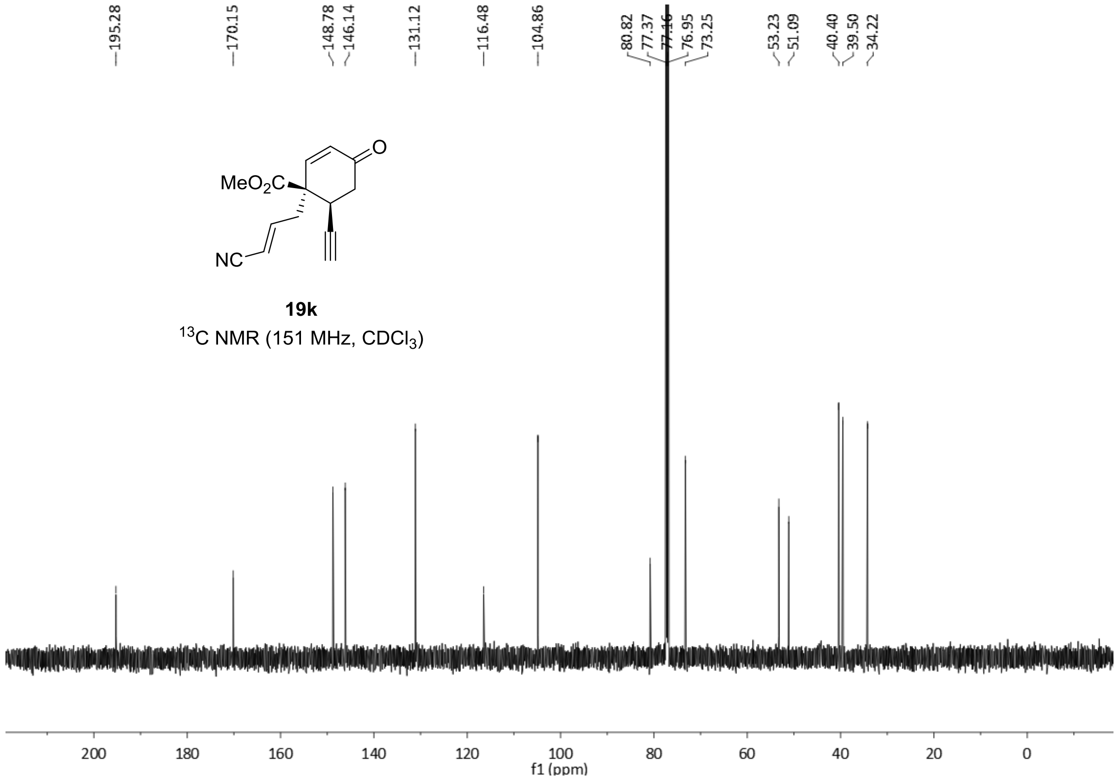

Supplementary Figure 261. ^{13}C NMR spectrum of **19k**

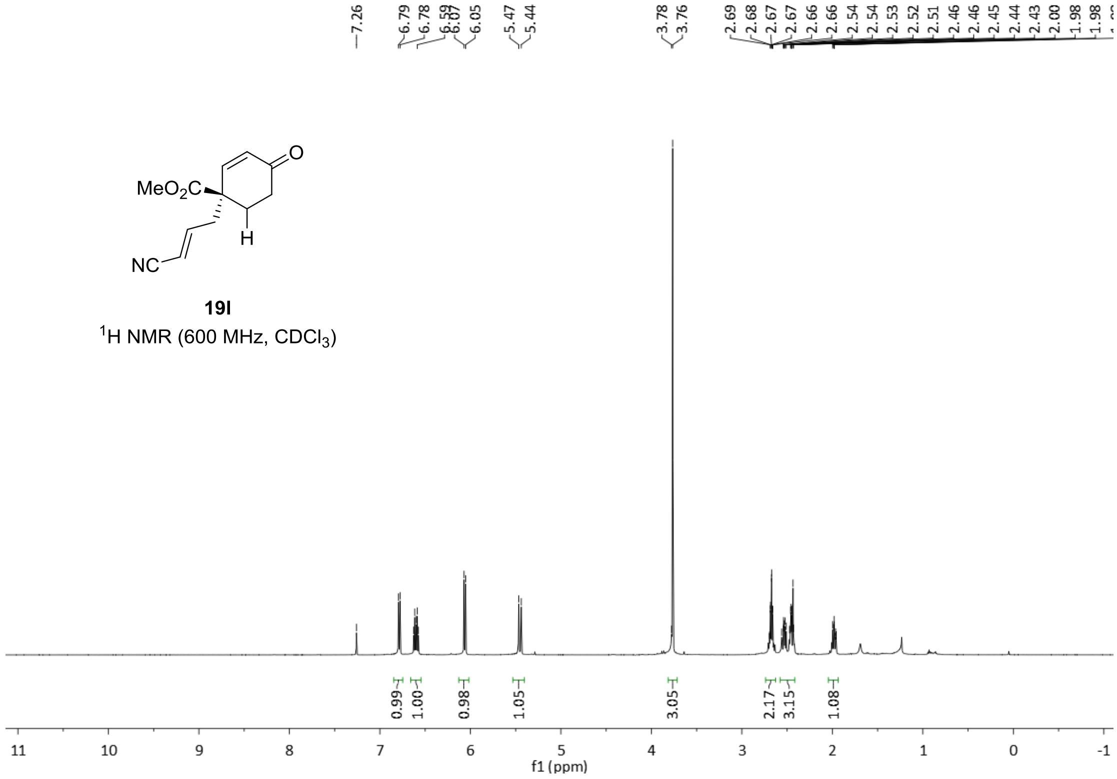

Supplementary Figure 262. ^{1}H NMR spectrum of **19l**

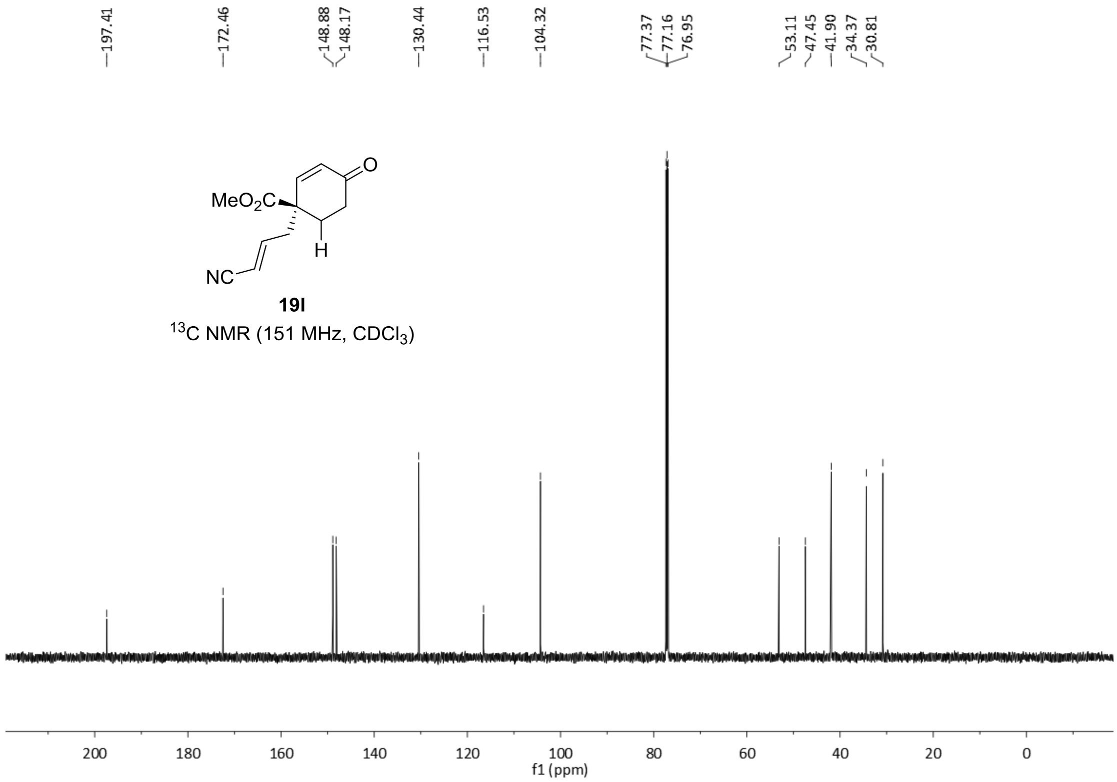

Supplementary Figure 263. ^{13}C NMR spectrum of **19l**

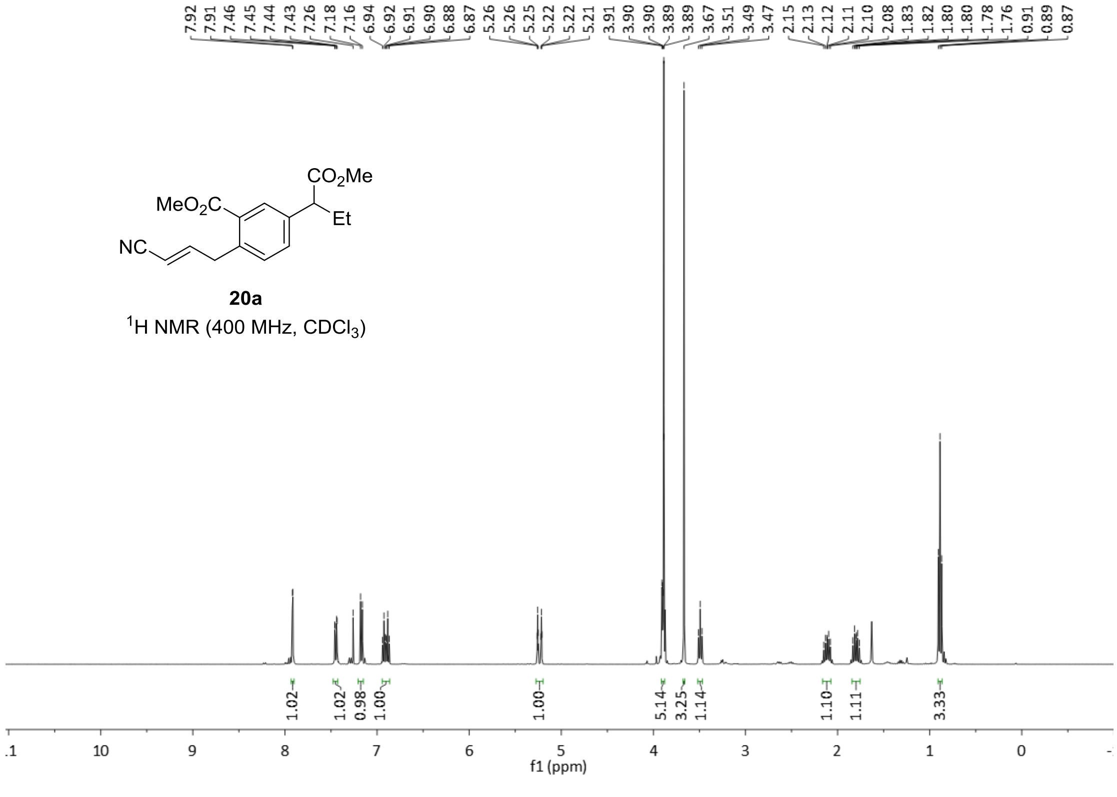

Supplementary Figure 264. ^{1}H NMR spectrum of **20a**

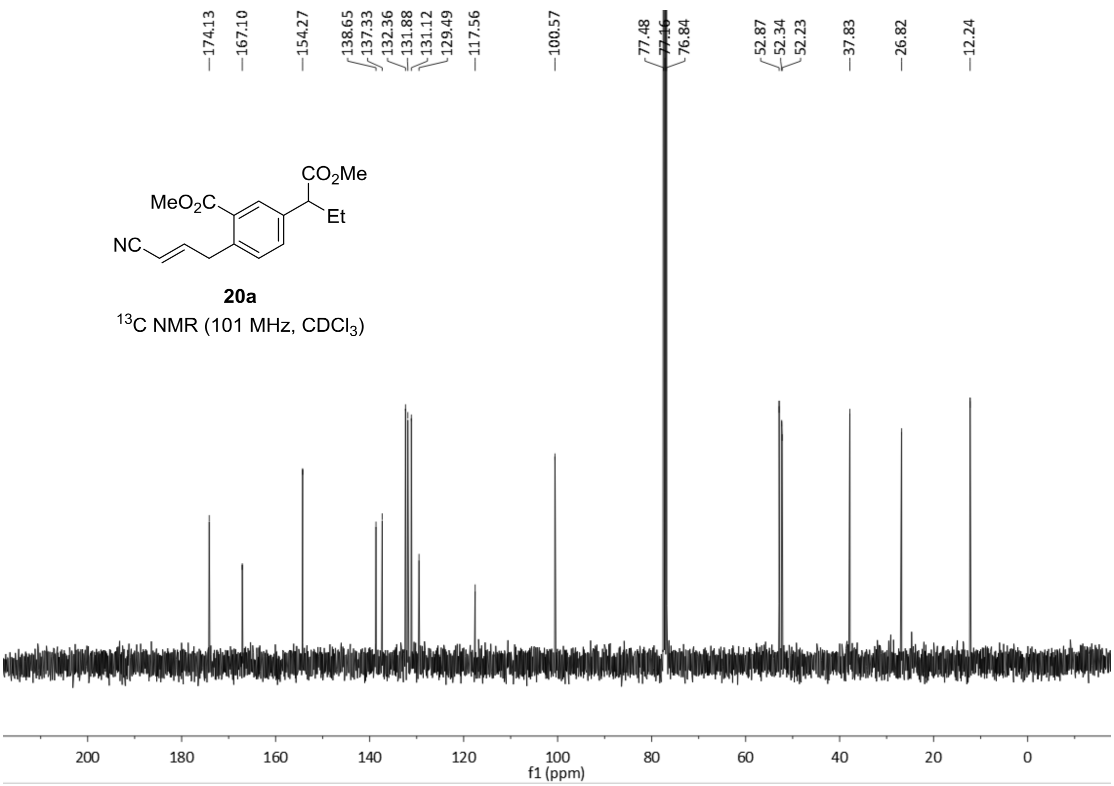

Supplementary Figure 265. ^{13}C NMR spectrum of **20a**

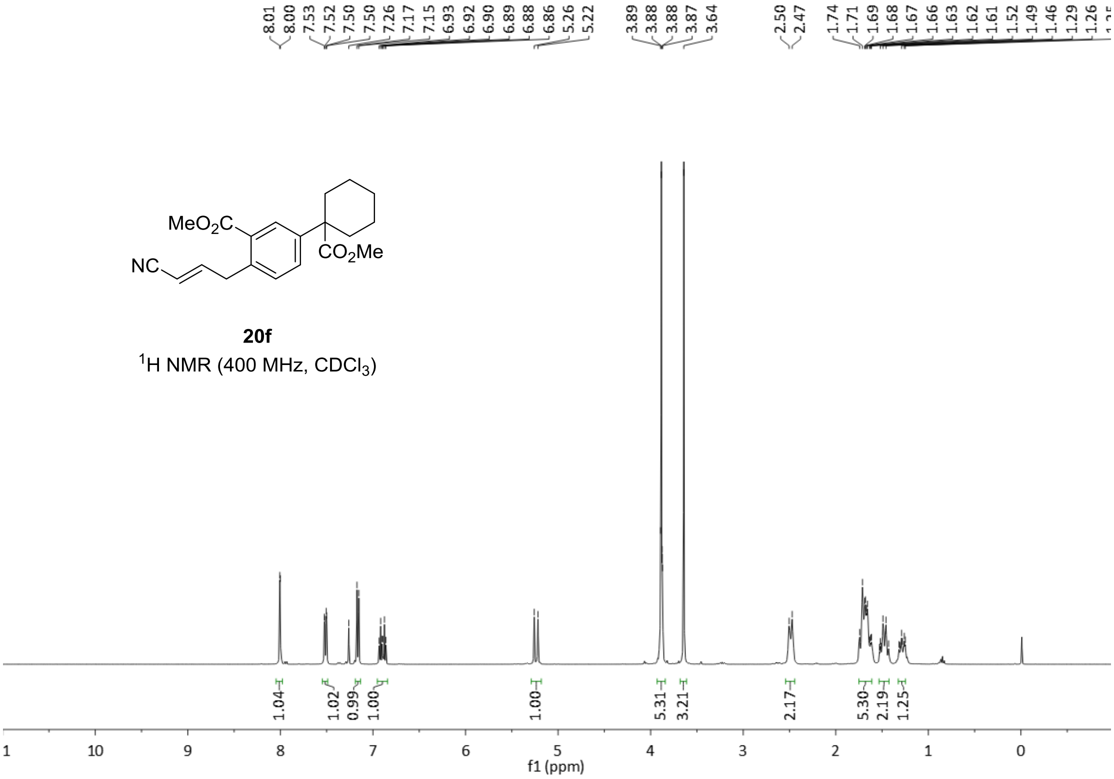

Supplementary Figure 266. ^{1}H NMR spectrum of **20f**

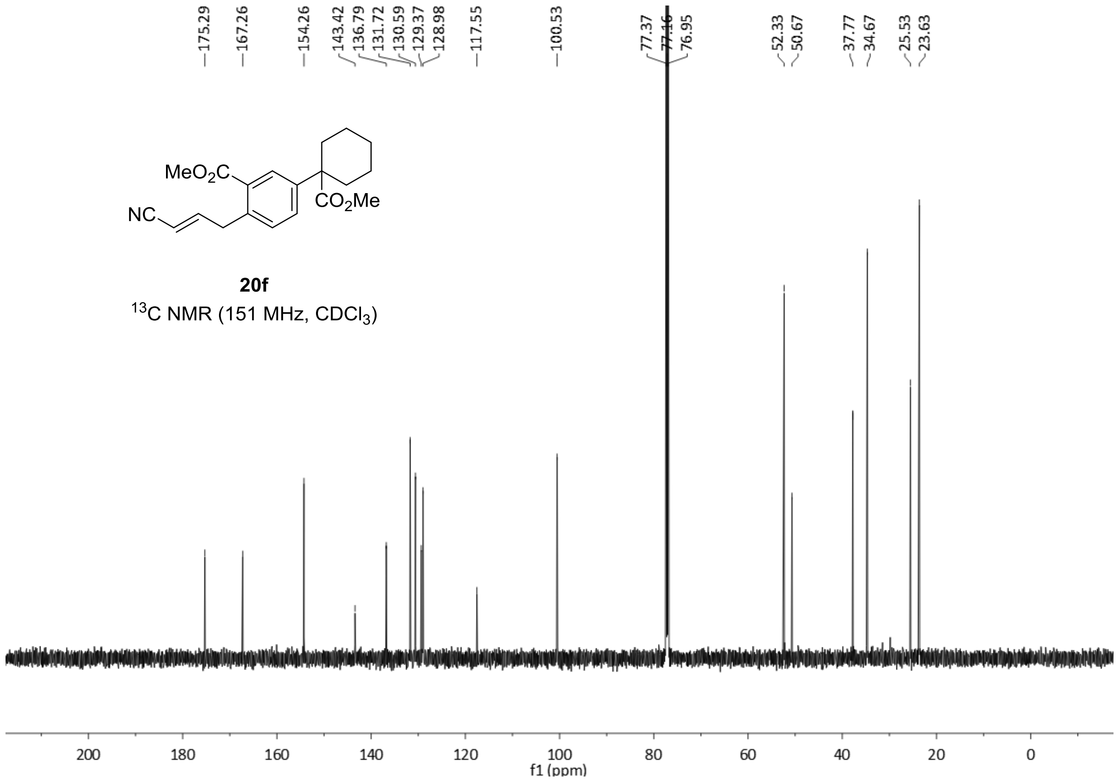

Supplementary Figure 267. ^{13}C NMR spectrum of **20f**

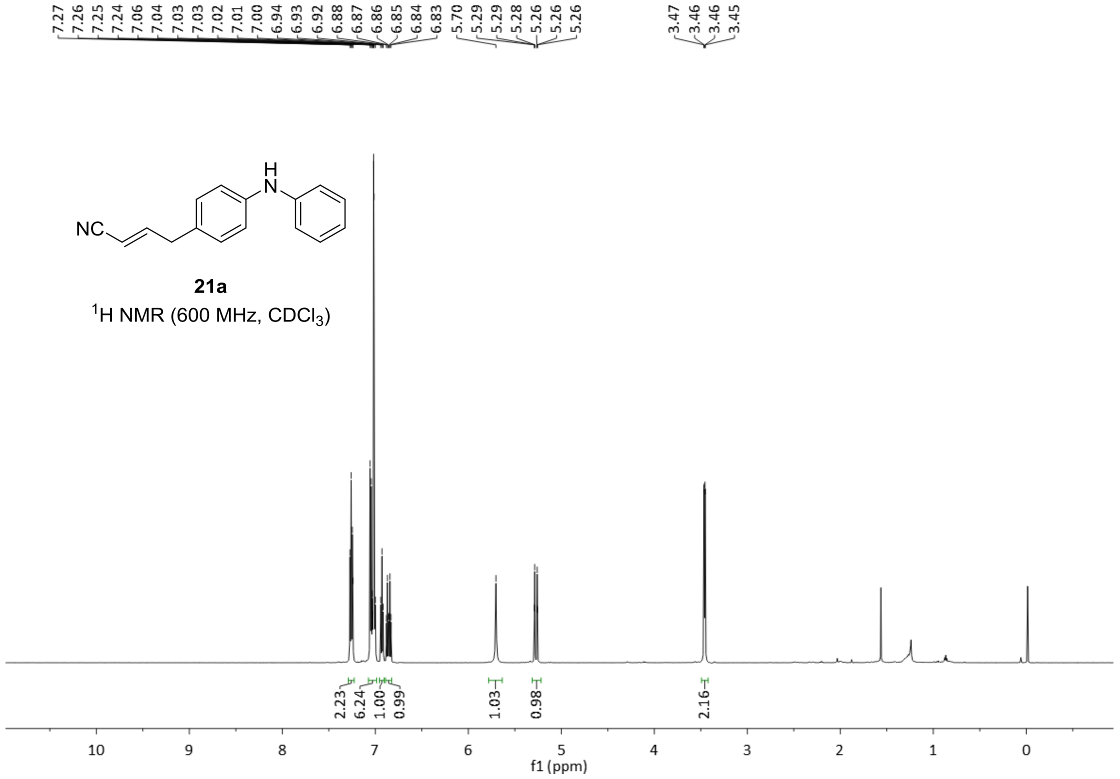

Supplementary Figure 268. ^{1}H NMR spectrum of **21a**

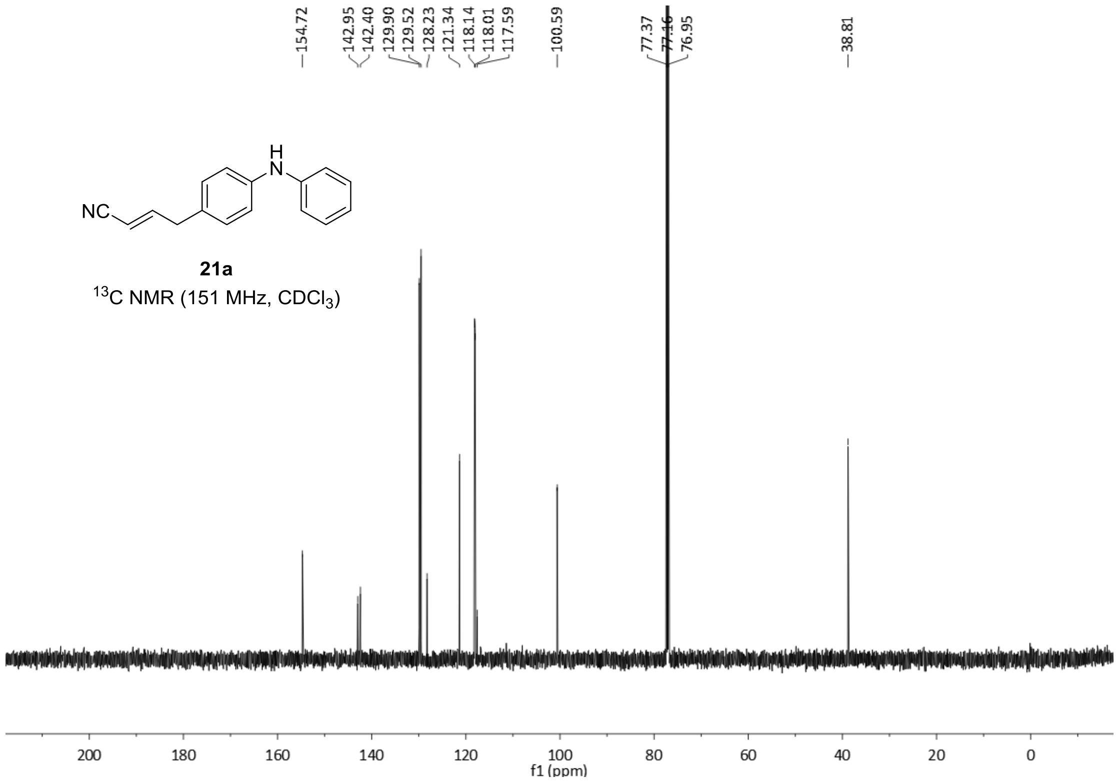

Supplementary Figure 269. ^{13}C NMR spectrum of **21a**

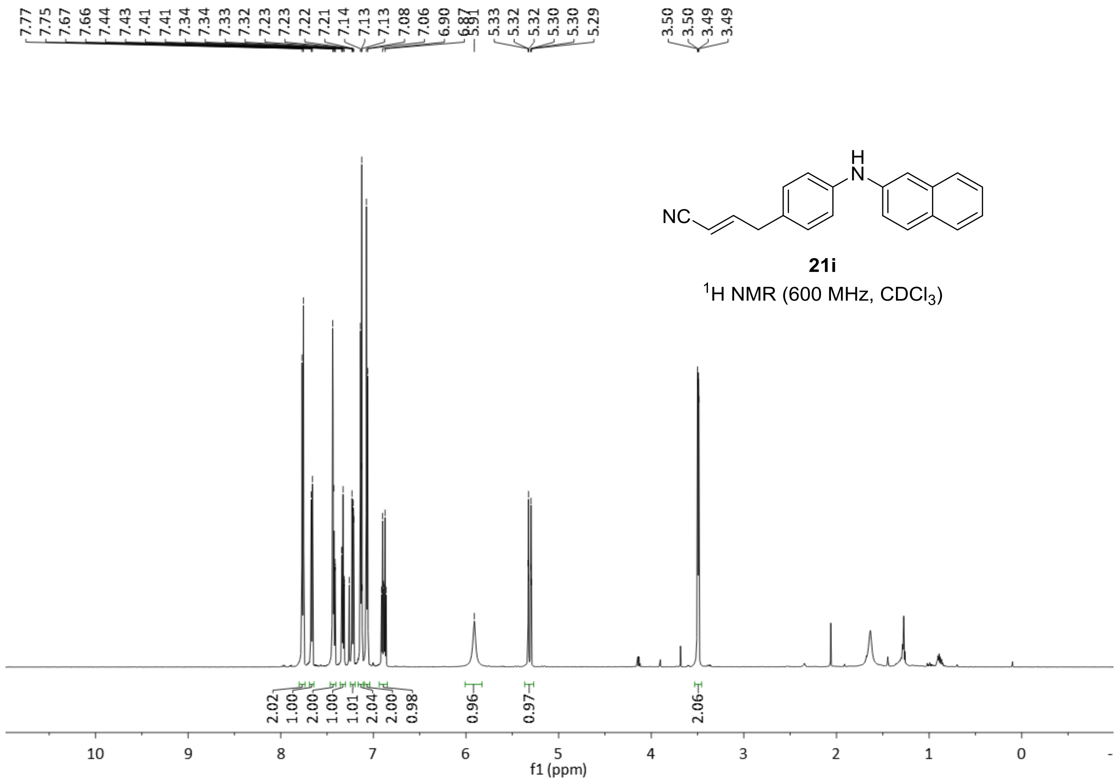

Supplementary Figure 270. ^{1}H NMR spectrum of **21i**

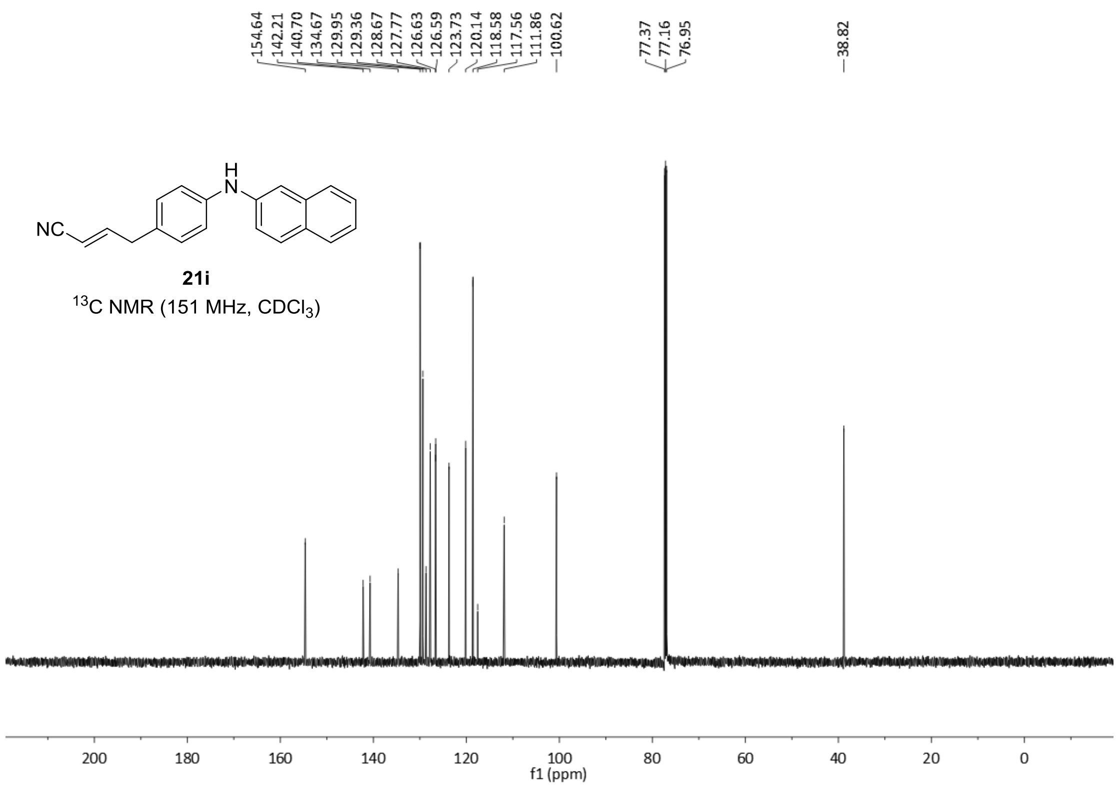

Supplementary Figure 271. ^{13}C NMR spectrum of **21i**

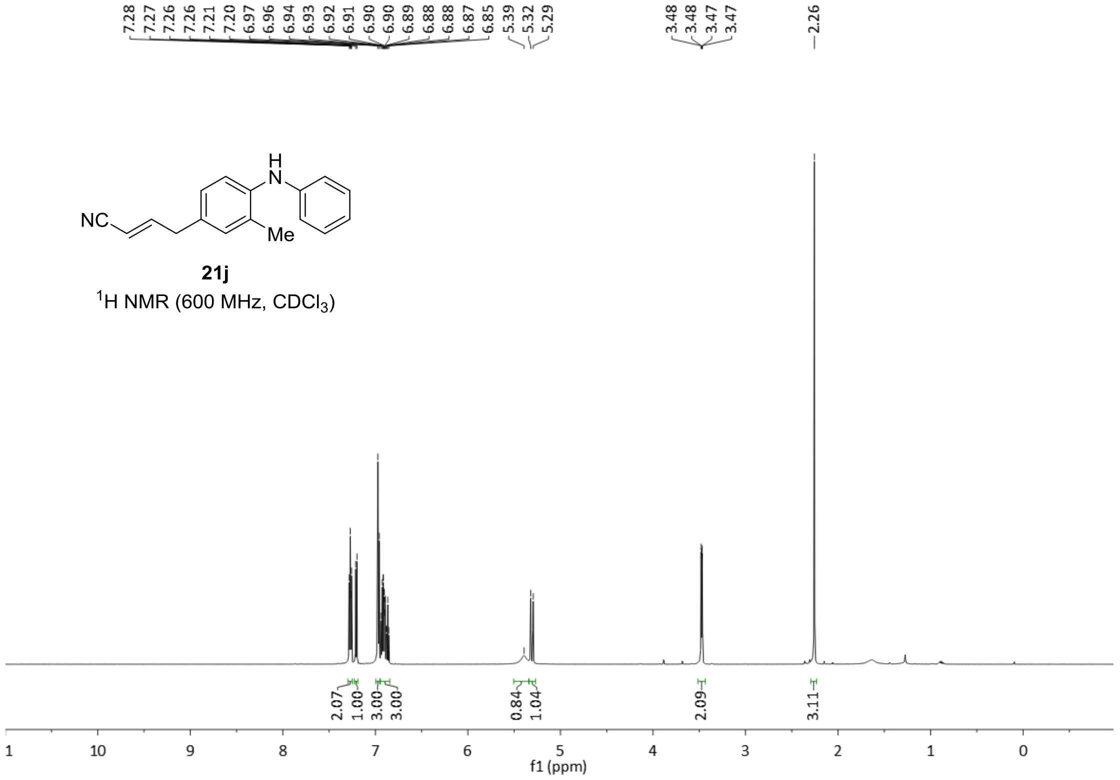

Supplementary Figure 272. ^{1}H NMR spectrum of **21j**

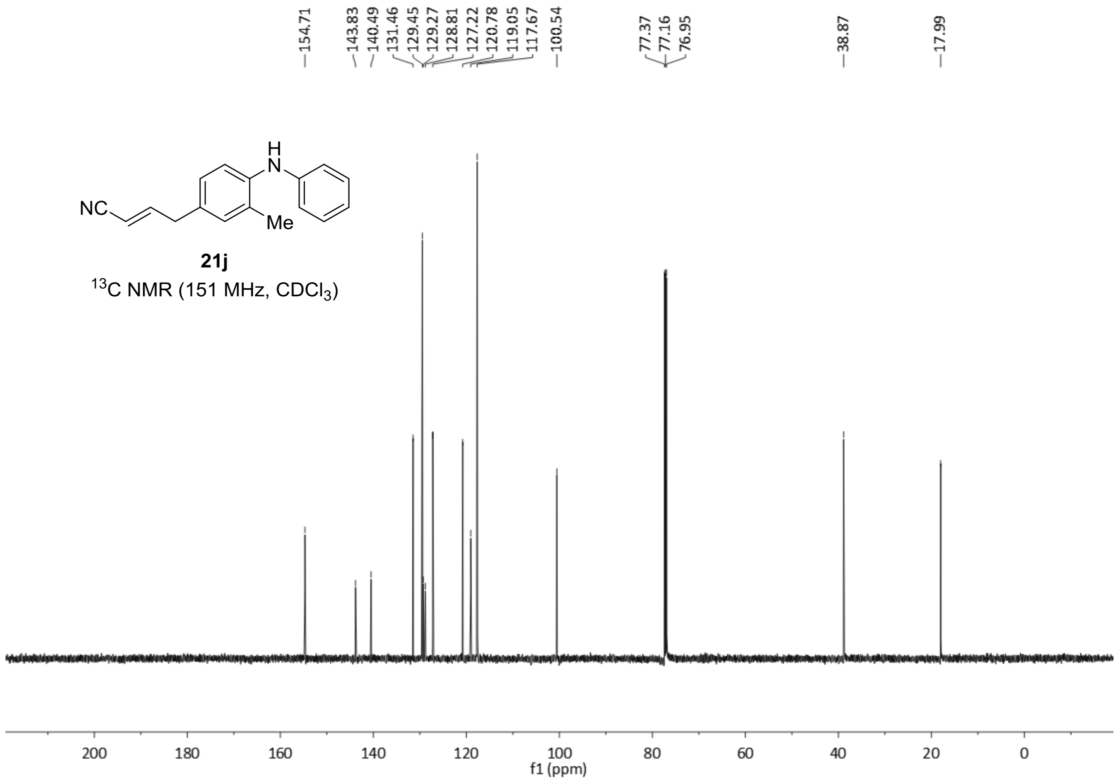

Supplementary Figure 273. ^{13}C NMR spectrum of **21j**

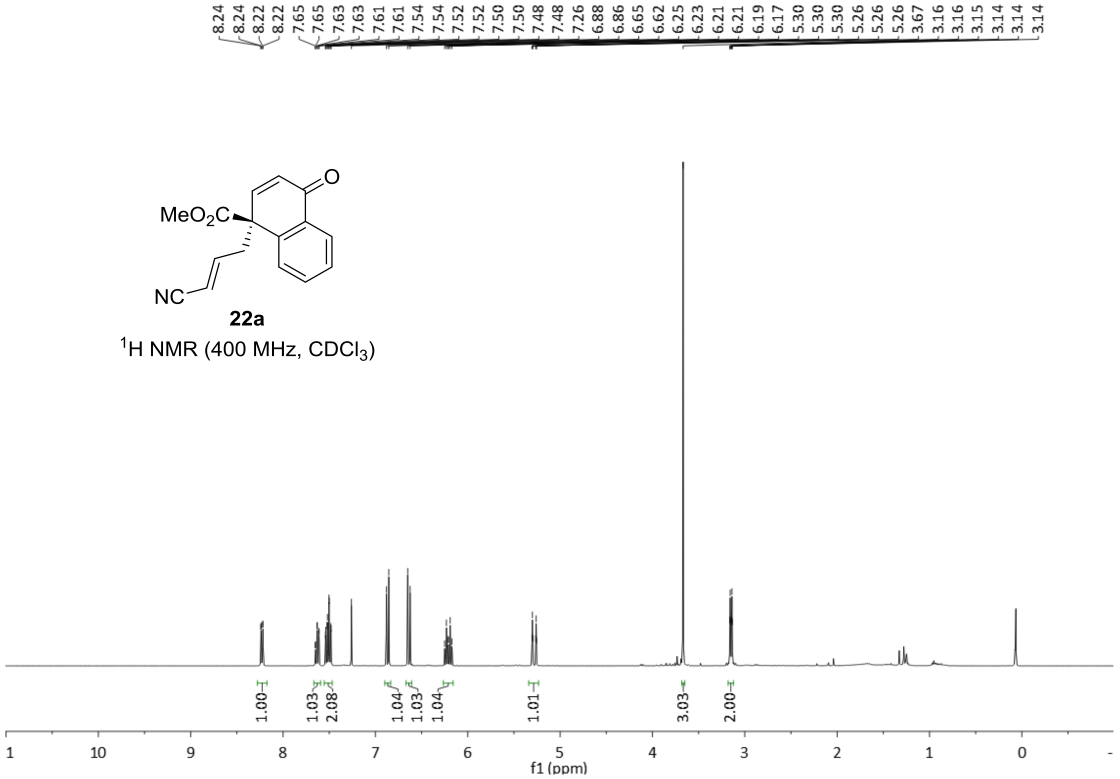

Supplementary Figure 274. ^{1}H NMR spectrum of **22a**

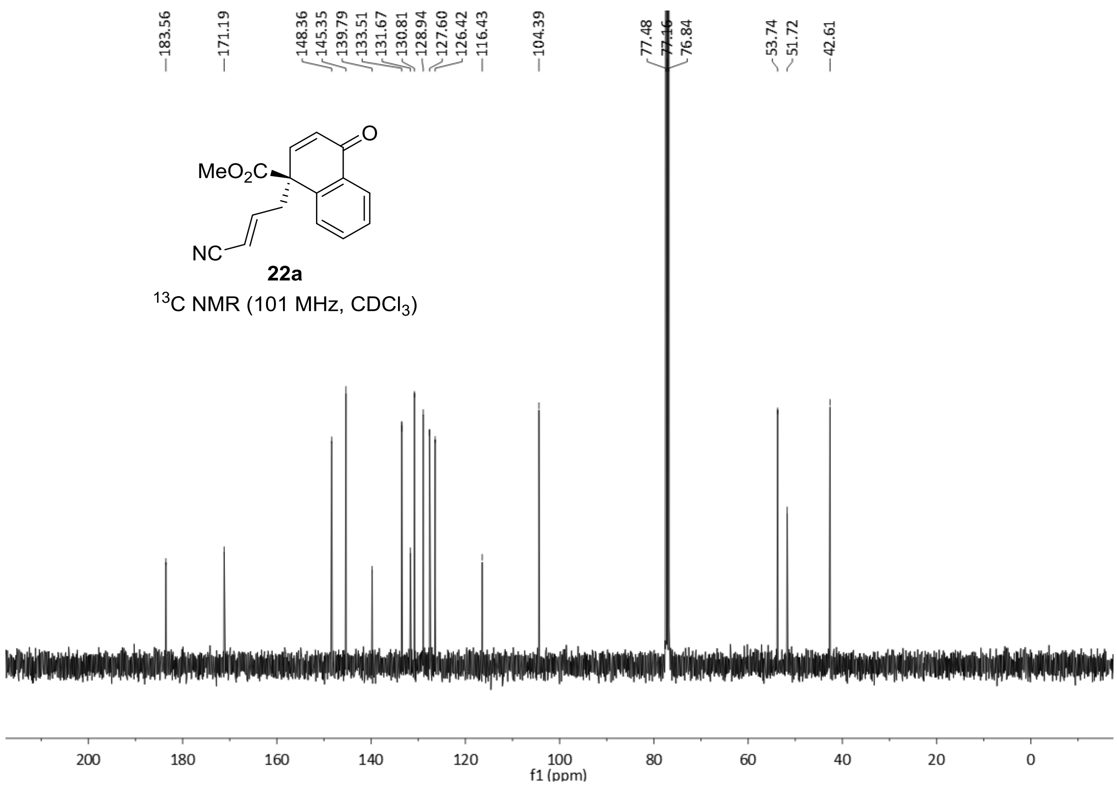

Supplementary Figure 275. ^{13}C NMR spectrum of **22a**

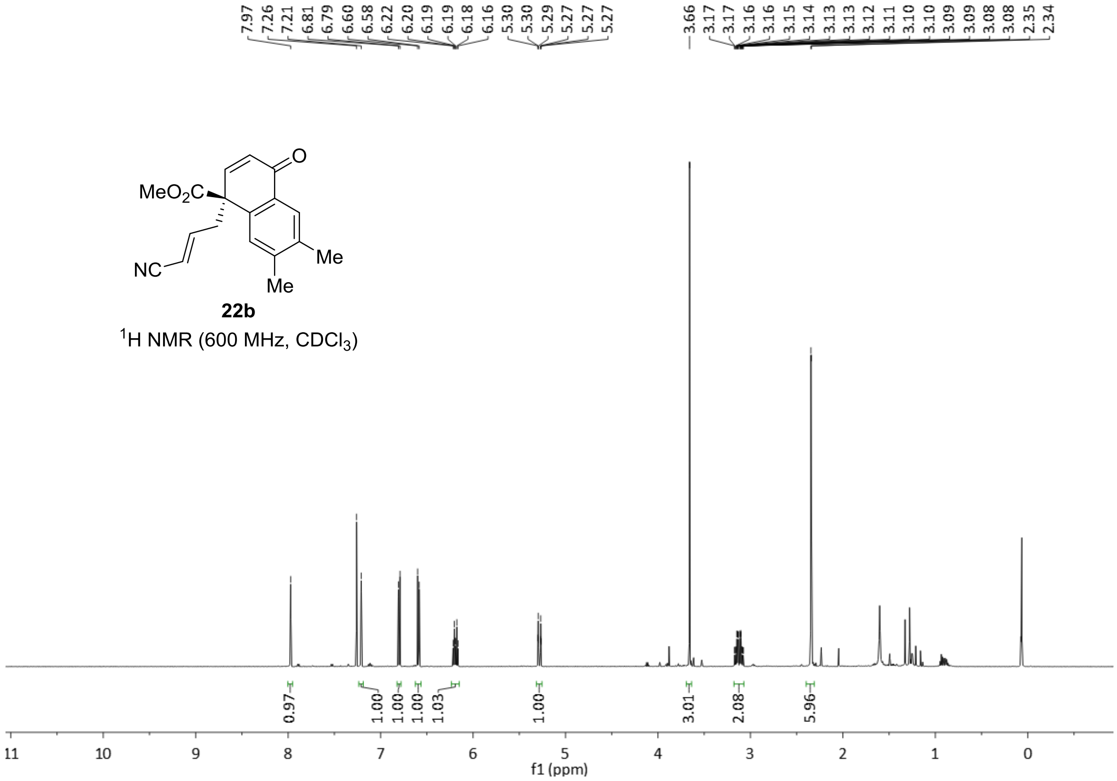

Supplementary Figure 276. ^{1}H NMR spectrum of **22b**

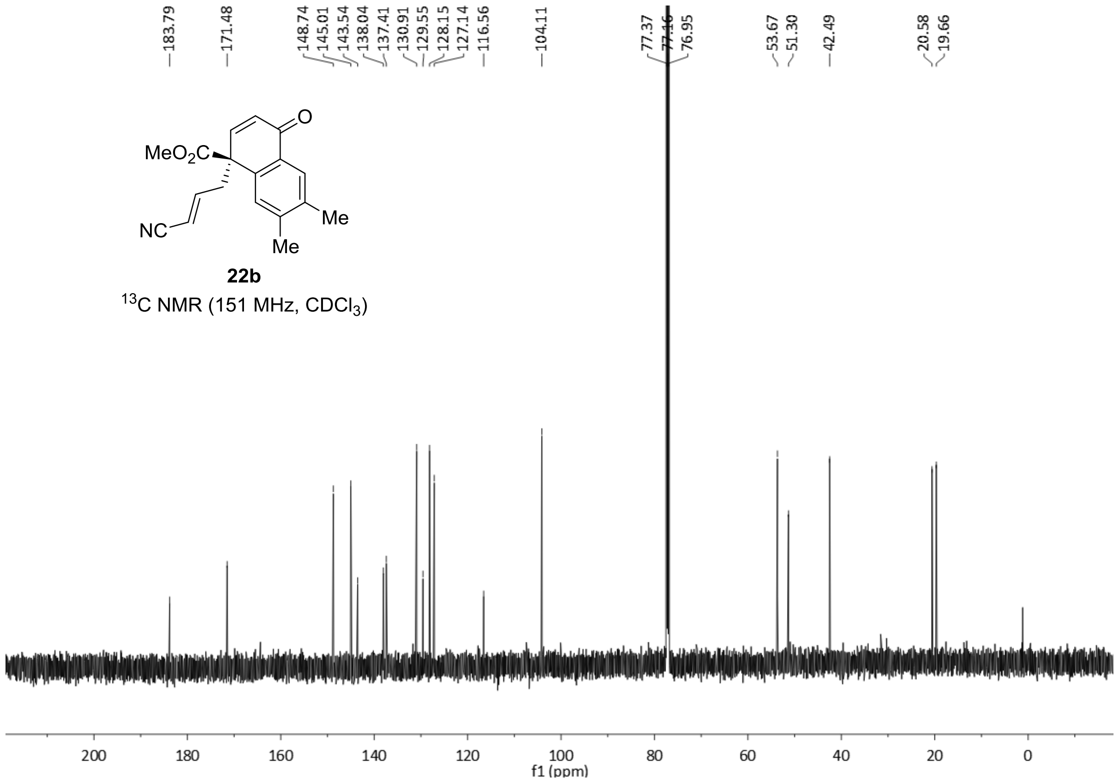

Supplementary Figure 277. ^{13}C NMR spectrum of **22b**

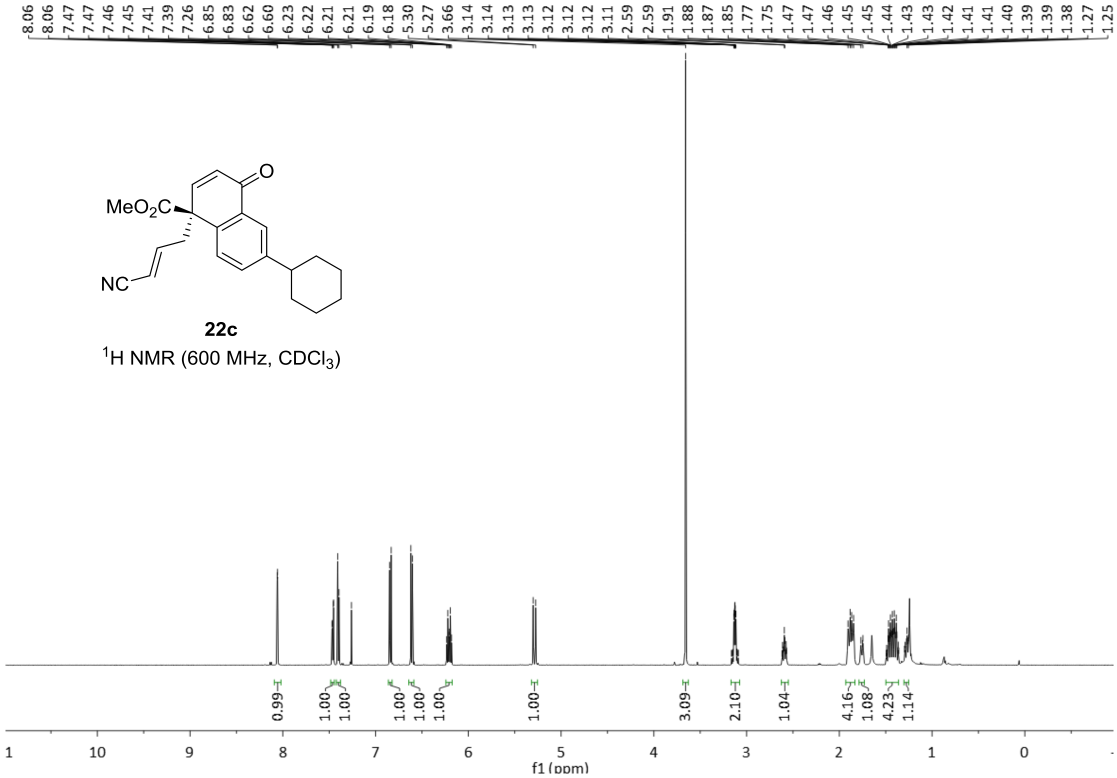

Supplementary Figure 278. ^{1}H NMR spectrum of **22c**

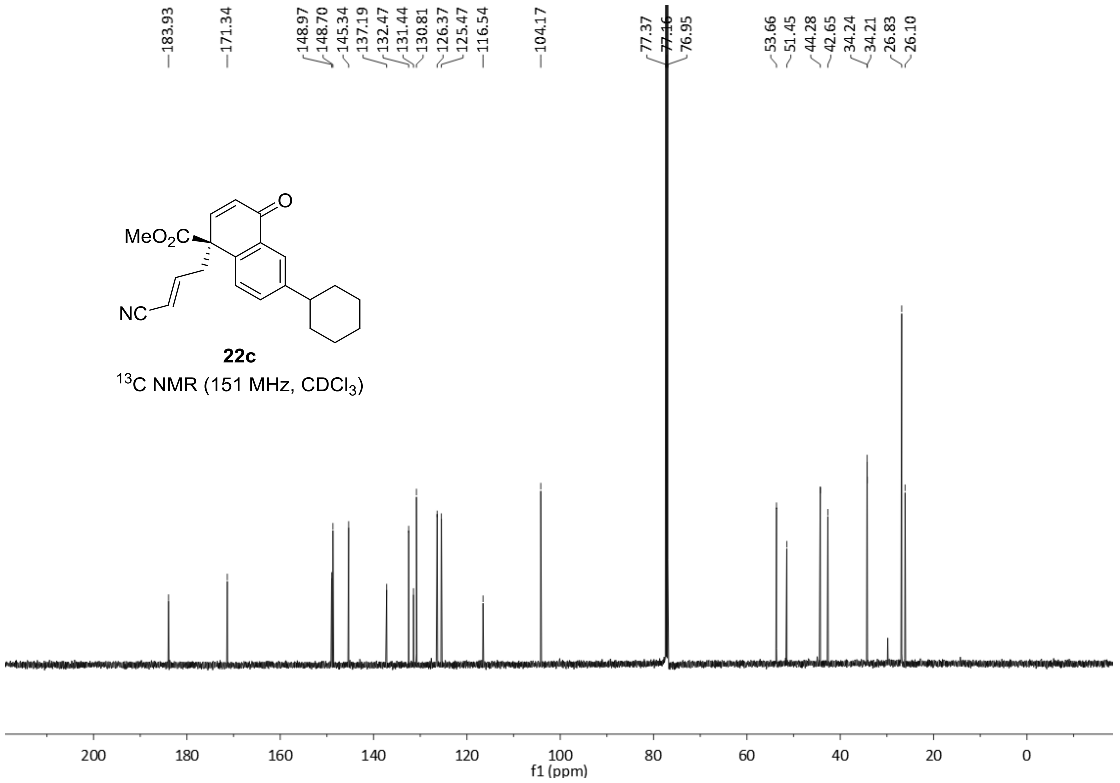

Supplementary Figure 279. ^{13}C NMR spectrum of **22c**

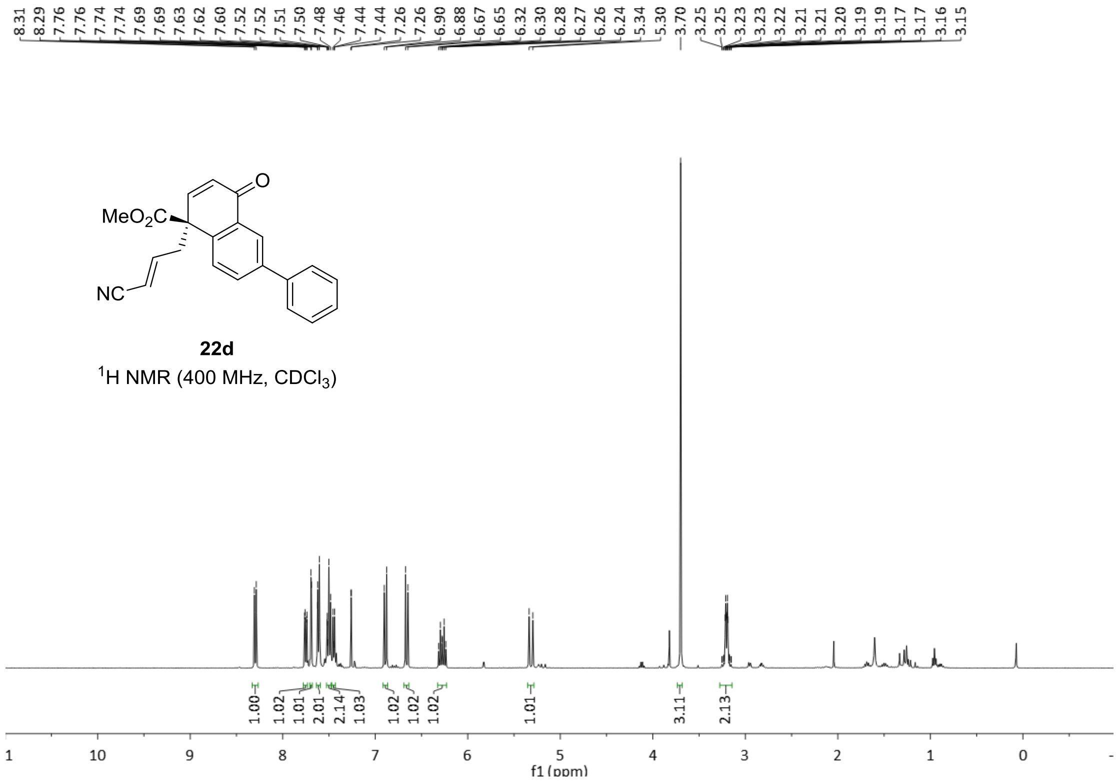

Supplementary Figure 280. ^{1}H NMR spectrum of **22d**

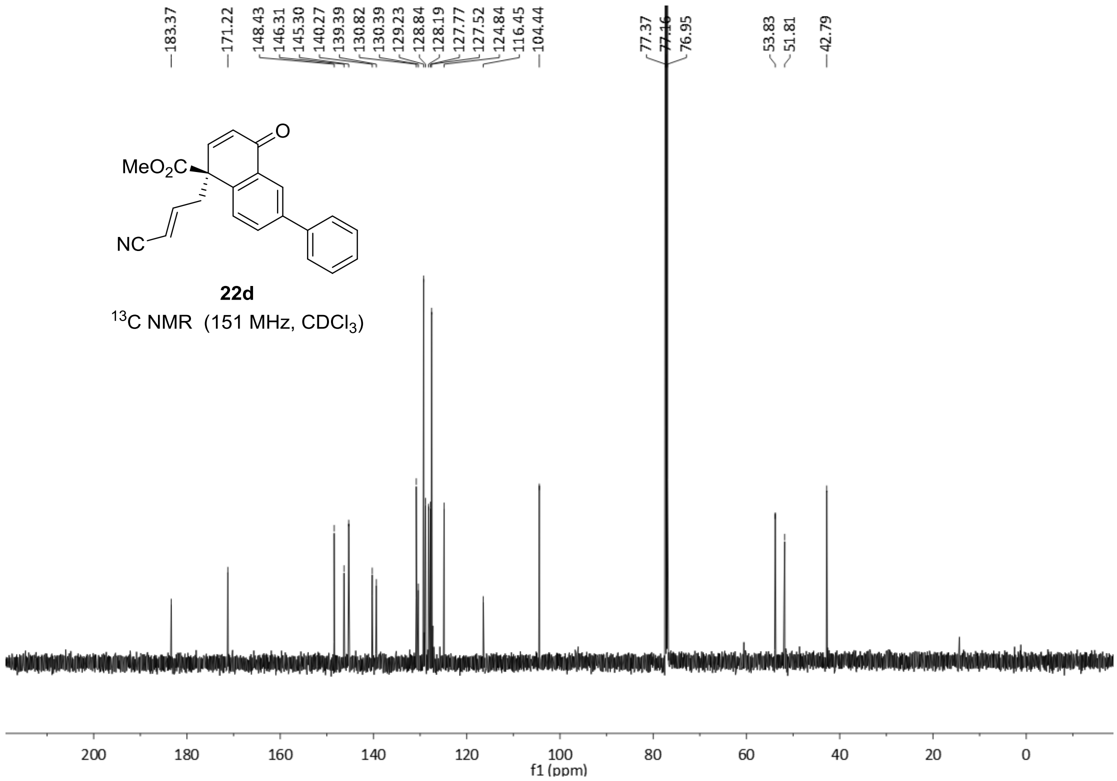

Supplementary Figure 281. ^{13}C NMR spectrum of **22d**

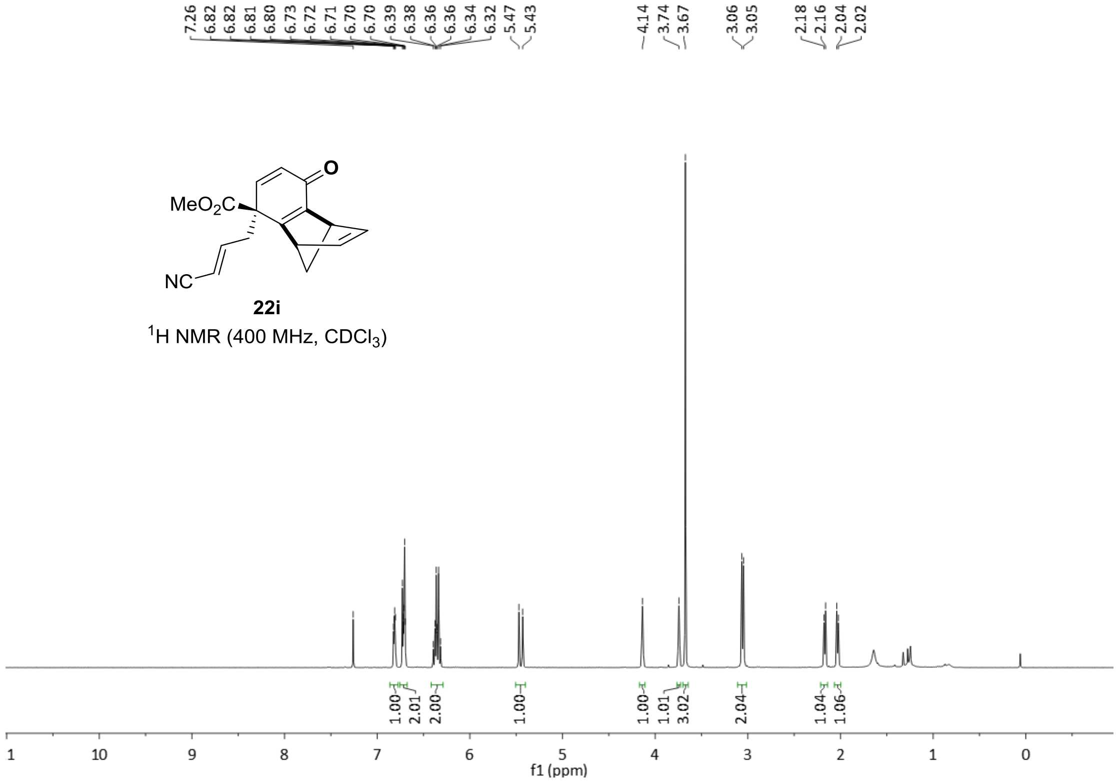

Supplementary Figure 282. ^{1}H NMR spectrum of **22i**

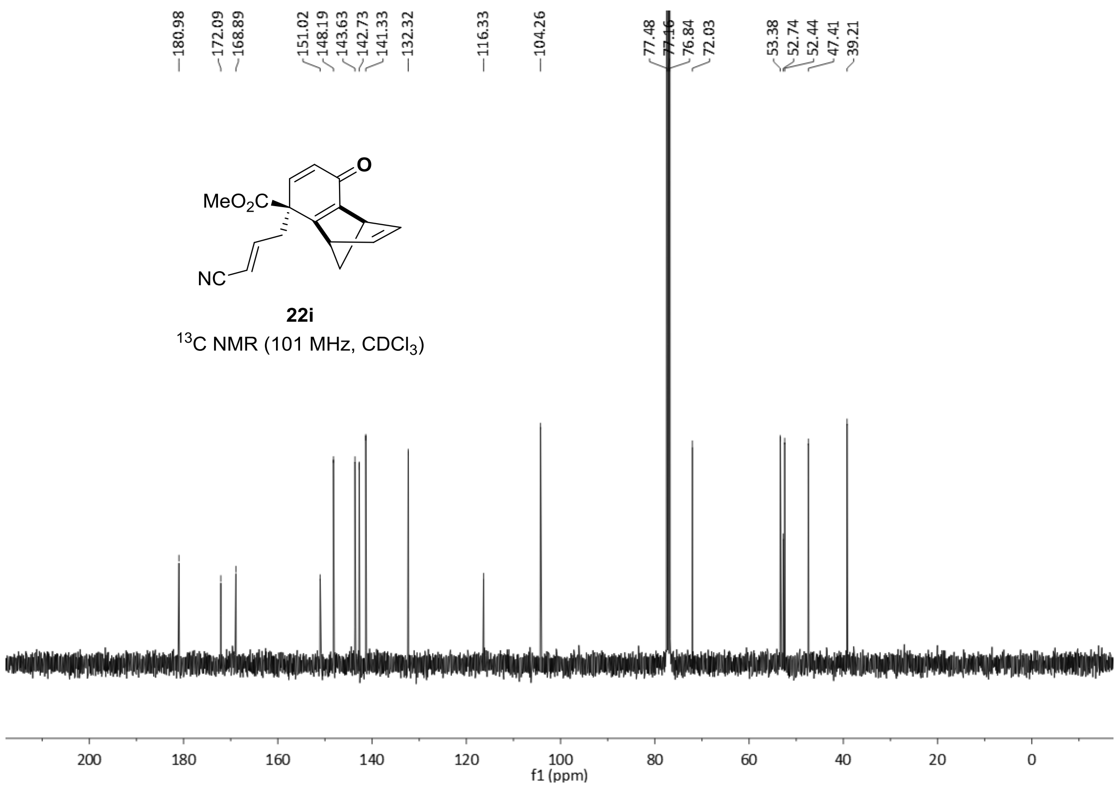

Supplementary Figure 283. ^{13}C NMR spectrum of **22i**

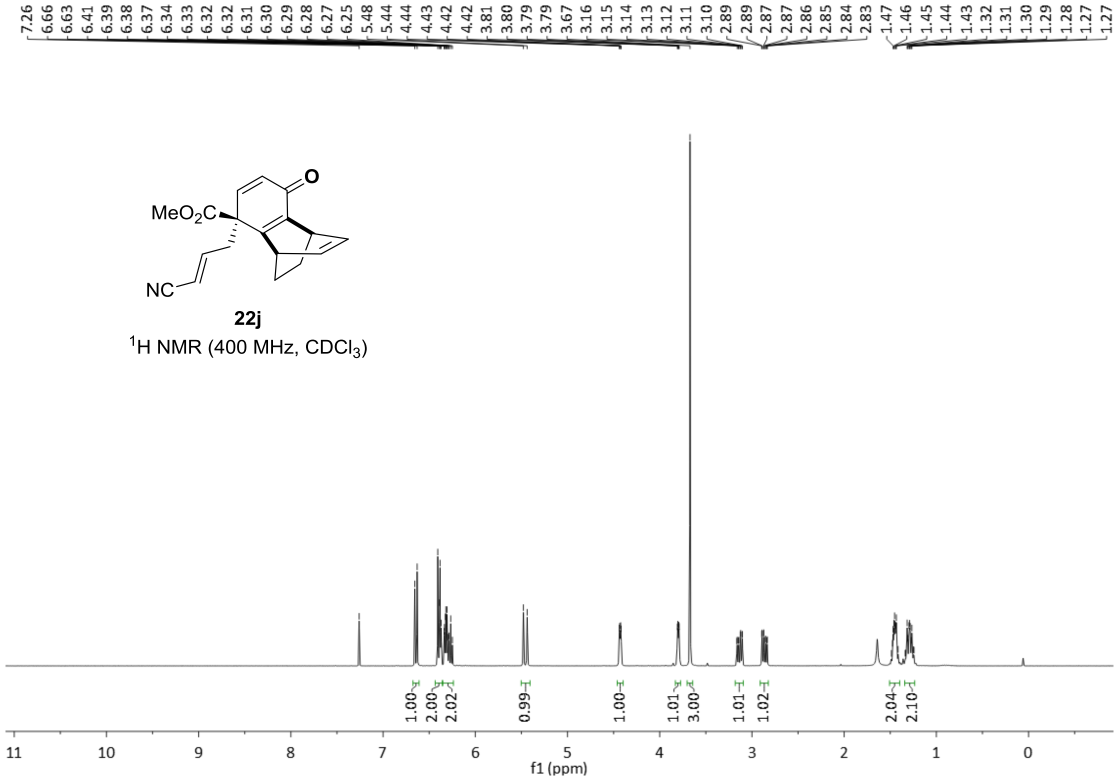

Supplementary Figure 284. ^{1}H NMR spectrum of **22j**

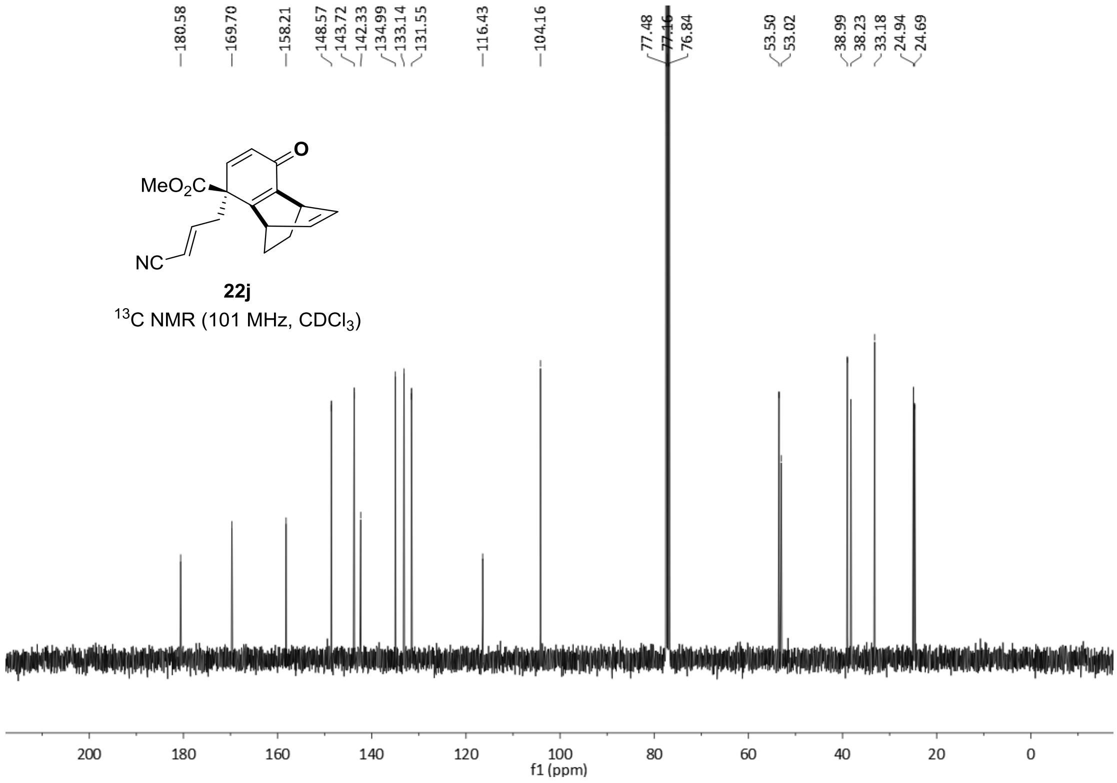

Supplementary Figure 285. ^{13}C NMR spectrum of **22j**

Supplementary References

[1] Chen, M.; Liang, Y.; Dong, T.; Liang, W.; Liu, Y.; Zhang, Y.; Huang, X.; Kong, L.; Wang Z.-X. & Peng, B. Z-selective α-arylation of α,β-unsaturated nitriles via [3,3]-sigmatropic rearrangement. *Angew. Chem. Int. Ed.* **60**, 2339-2345 (2021).

[2] Hugenberg, V. & Haufe, G. Facile synthesis of α,α-difluoroalkyl aryl thioethers and their oxidative desulfurization-fluorination to trifluorides. *J. Fluorine Chem.* **131**, 942-950 (2010).

[3] Tian, Z.-Y. & Zhang, C.-P. Ullmann-type N-arylation of anilines with alkyl(aryl)sulfonium salts. *Chem. Commun.* **55**, 11936-11939 (2019).

[4] Kemme, S. T.; Šmejkal, T. & Breit, B. Combined transition-metal- and organocatalysis: an atom economic C3 homologation of alkenes to carbonyl and carboxylic compounds. *Chem. Eur. J.* **16**, 3423-3433 (2010).

[5] Zhang, L.; He, J.-N.; Liang, Y.; Hu, M.; Shang, L.; Huang, X.; Kong, L.; Wang Z.-X. & Peng, B. Selective [5,5]-sigmatropic rearrangement by assembly of aryl sulfoxides with allyl nitriles. *Angew. Chem. Int. Ed.* **58**, 5316-5320 (2019).

[6] Smith, S. M.; Thacker, N. C. & Takacs, J. M. Efficient amide-directed catalytic asymmetric hydroboration. *J. Am. Chem. Soc.* **130**, 3734-3735 (2008).

[7] Wenzel, A. & Jacobsen, E. Asymmetric catalytic mannich reactions catalyzed by urea derivatives: enantioselective synthesis of *β*-aryl-*β*-amino acids. *J. Am. Chem. Soc.* **124**, 12964-12965 (2002).

[8] Okano, T.; Shimizu, T.; Sumida, K. & Eguchi, S. Two-step synthesis of (γ,γ-difluoroallyl)carbonyl compounds via addition of 1,1,2-Trichloro-2,2-difluoroethyl radical to silyl enol ethers followed by reductive dechlorination. *J. Org. Chem.* **58**, 5163-5166 (1993).

[9] Siegel, D.; Piizzi, G.; Piersanti, G. & Movassaghi, M. Enantioselective total synthesis of (-)-acylfulvene and (-)-irofulven. *J. Org. Chem.* **74**, 9292-9304 (2009).

[10] Chulsky, K. & Dobrovetsky, R. Metal-free catalytic reductive cleavage of enol ethers. *Org. Lett.* **20**, 6804-6807 (2018).

[11] Hu, Y.; Sun, W.; Zhang, T.; Xu, N.; Xu, J.; Lan, Y. & Liu, C. Stereoselective synthesis of

trisubstituted vinylboronates from ketone enolates triggered by 1,3-metalate rearrangement of lithium enolates. *Angew. Chem. Int. Ed.* **58**, 15813-15818 (2019).

[12] Khan, I.; Reed-Berendt, B.; Melen, R. & Morrill, L. FLP-catalyzed transfer hydrogenation of silyl enol ethers. *Angew. Chem. Int. Ed.* **57**, 12356-12359 (2018).

[13] Ratjen, L.; Garcǐa-Garcǐa, P.; Lay, F.; Beck, M. & List, B. Disulfonimide-catalyzed asymmetric vinylogous and bisvinylogous mukaiyama aldol reactions. *Angew. Chem. Int. Ed.* **50**, 754-758 (2011).

[14] Cai, Y.; Roberts, B. P.; Tocher, D. A. & Barnett, S. A. Carbon–carbon bond formation by radical addition–fragmentation reactions of O-alkylated enols. *Org. Biomol. Chem.* **2**, 2517-2529 (2004).

[15] Edwards, J.; Merchant, R.; McClymont, K.; Knouse, K.; Qin, T.; Malins, L.; Vokits, B.; Shaw, S.; Bao, D.-H.; Wei, F.-L.; Zhou, T.; Eastgate, M. & Baran, P. Decarboxylative alkenylation. *Nature* **545**, 213-218 (2017).

[16] Chen, T.-G.; Barton, L.; Lin, Y.; Tsien, J.; Kossler, D.; Bastida, I.; Asai1, S.; Bi, C.; Chen, J.; Shan, M.; Fang, H.; Fang, F.; Choi, H.-W.; Hawkins, L.; Qin, T. & Baran, P. Building C(sp3)-rich complexity by combining cycloaddition and C–C cross-coupling reactions. *Nature* **560**, 350 (2018).

[17] Wang, C.; Wu, L.; Xu, W.; He, F.; Qu, J. & Chen, Y. Palladium-catalyzed secondary benzylic imidoylative reactions. *Org. Lett.* **22**, 6954-6959 (2020).

[18] Chen, Y.-H.; Tüllmann, C.; Ellwart, M. & Knochel, P. Preparation of solid polyfunctional alkynylzinc pivalates with enhanced air and moisture stability for organic synthesis. *Angew. Chem. Int. Ed.* **56**, 9236-9239 (2017).

[19] Liu, X.-G.; Zhou, C.-J.; Lin, E.; Han, X.-L.; Zhang, S.-S.; Li, Q. & Wang, H. Decarboxylative negishi coupling of redox-active aliphatic esters by cobalt catalysis. *Angew. Chem. Int. Ed.* **57**, 13096-13100 (2018).

[20] Stahl, I. Synthese cyclischer dithioacetale von β-ketoestern aus keten-silylacetalen. *Chem. Ber.* **118**, 3159-3165 (1985).

[21] Gatzenmeier, T.; Kaib, P. S. J.; Lingnau, J. B.; Goddard, R. & List, B. The catalytic

asymmetric Mukaiyama–Michael reaction of silyl ketene acetals with α, β-unsaturated methyl esters. *Angew. Chem. Int. Ed.* **57**, 2464-2468 (2018).

[22] Hachiya, I.; Nakamura, K.; Hara, M.; Sato, K. & Shimizu, M. Domino 1,4- and 1,6-addition reactions of ketene silyl acetals to dialkynyl imines promoted by aluminum chloride: synthesis of multifunctionalized β‑ lactams. *J. Org. Chem.* **84**, 14770-14794 (2019).

[23] Fiorito, D.; Folliet, S.; Liu, Y. & Mazet, C. A general nickel-catalyzed kumada vinylation for the preparation of 2-substituted 1,3-dienes. *ACS Catal.* **8**, 1392-1398 (2018).

[24] Cardinale, G.; Laan, J. A. M. & Ward, J. P. The Diels-Alder reaction with 6-methylene-7-octenoic acid, a functionalised butadiene. *Recl. Trav. Chim. Pays-Bas* **106**, 62-64 (1987).

[25] Nicolaou, K. C.; Hale, C. R. H.; Nilewski, C.; Ioannidou, H. A.; ElMarrouni, A.; Nilewski, L. G.; Beabout, K.; Wang, T. T. & Shamoo, Y. Total synthesis of viridicatumtoxin B and analogues thereof: strategy evolution, structural revision, and biological evaluation. *J. Am. Chem. Soc.* **136**, 12137-12160 (2014).

[26] Pagoti, S.; Dutta, D. & Dash J. A magnetoclick imidazolidinone nanocatalyst for asymmetric 1,3-dipolar cycloadditions. *Adv. Synth. Catal.* **355**, 3532-3538 (2013).

[27] Kano, T.; Hashimoto, T. & Maruoka, K. Asymmetric 1,3-dipolar cycloaddition reaction of nitrones and acrolein with a bis-titanium catalyst as chiral lewis acid. *J. Am. Chem. Soc.* **127**, 11926-11927 (2005).

[28] Jin, Y.; Yang, H. & Fu, H. An N-(acetoxy)phthalimide motif as a visible-light pro-photosensitizer in photoredox decarboxylative arylthiation. *Chem. Commun.* **52**, 12909-12912 (2016).

[29] Zhao, Y. & Truhlar, D. G. The M06 suite of density functionals for main grou thermochemistry, thermochemical kinetics, noncovalent interactions, excited states, and transition elements: two new functionals and systematic testing of four M06-class functionals and 12 other functionals. *Theor. Chem. Acc.* **120**, 215-241 (2008).

[30] Zhao, Y. & Truhlar, D. G. Density functionals with broad applicability in chemistry. *Acc. Chem. Res.* **41**, 157-167 (2008).

[31] Valero, R.; Costa, R.; Moreira, I. D. P. R.; Truhlar, D. G. & Illas, F. Performance of the M06 family of exchangecorrelation functionals for predicting magnetic coupling in organic and inorganic molecules. *J. Chem.Phys.* **128**, 114103 (2008).

[32] Marenich, A. V.; Cramer, C. J. & Truhlar, D. G. Universal solvation model based on solute electron density and on a continuum model of the solvent defined by the bulk dielectric constant and atomic surface tensions. *J. Phys. Chem. B* **113**, 6378-6396 (2009).

[33] Gaussian 09, Revision A.01, Frisch, M. J.; Trucks, G. W.; Schlegel, H. B.; Scuseria, G. E.; Robb, M. A.; Cheeseman, J. R.; Scalmani, G.; Barone, V.; Mennucci, B.; Petersson, G. A.; Nakatsuji, H.; Caricato, M.; Li, X.; Hratchian, H. P.; Izmaylov, A. F.; Bloino, J.; Zheng, G.; Sonnenberg, J. L.; Hada, M.; Ehara, M.; Toyota, K.; Fukuda, R.; Hasegawa, J.; Ishida, M.; Nakajima, T.; Honda, Y.; Kitao, O.; Nakai, H.; Vreven, T.; Montgomery, J. A.; Peralta, Jr., J. E.; Ogliaro, F.; Bearpark, M.; Heyd, J. J.; Brothers, E.; Kudin, K. N.; Staroverov, V. N.; Keith, T.; Kobayashi, R.; Normand, J.; Raghavachari, K.; Rendell, A.; Burant, J. C.; Iyengar, S. S.; Tomasi, J.; Cossi, M.; Rega, N.; Millam, J. M.; Klene, M.; Knox, J. E.; Cross, J. B.; Bakken, V.; Adamo, C.; Jaramillo, J.; Gomperts, R.; Stratmann, R. E.; Yazyev, O.; Austin, A. J.; Cammi, R.; Pomelli, C.; Ochterski, J. W.; Martin, R. L.; Morokuma, K.; Zakrzewski, V. G.; Voth, G. A.; Salvador, P.; Dannenberg, J. J.; Dapprich, S.; Daniels, A. D.; Farkas, O.; Foresman, J. B.; Ortiz, J. V.; Cioslowski, J. & Fox, D. J. Gaussian, Inc., Wallingford CT, **2009**.
