# Supplementary material for: Dearomative di- and trifunctionalization of aryl sulfoxides via [5,5]-rearrangement
Source: Nat Commun. 2022 Aug 11;13:4719. doi: 10.1038/s41467-022-32426-6 (PMC9372148; doi:10.1038/s41467-022-32426-6)
Supplement: Supplementary file 2 — Description of Additional Supplementary Files [file 41467_2022_32426_MOESM2_ESM.docx]

Description of Additional Supplementary Files

File Name: Supplementary Data 1

Description: Cartesian coordinates and energies of the optimized structures
