# Supplementary material for: Dearomative di- and trifunctionalization of aryl sulfoxides via [5,5]-rearrangement
Source: Nat Commun. 2022 Aug 11;13:4719. doi: 10.1038/s41467-022-32426-6 (PMC9372148; doi:10.1038/s41467-022-32426-6)
Supplement: Supplementary file 3 — Supplementary data 1 [file 41467_2022_32426_MOESM3_ESM.docx]

**Cartesian Coordinates (in Å), SCF Energies, and Free Energies (in a.u.) at 178.15 K and 1 atm for the Optimized Structures**

BSI=6-31G(d,p)

BSII=6-311++(d,p)

**1a**

M062x/BSI SCF energy: -1090.442995 a.u.

M062x/BSII SCF energy in solution: -1090.65717 a.u.

M062x/BSII free energy in solution: -1090.415829 a.u.

S -1.866677 1.086810 0.775540

O 4.561131 0.127421 -0.902014

O 4.424924 -1.450390 0.686726

C -0.149729 0.576472 0.548499

C 0.609667 1.250638 -0.399378

C 1.941342 0.889076 -0.579908

C 2.490662 -0.136636 0.191306

C 1.715788 -0.799242 1.147633

C 0.388389 -0.439761 1.336531

C -2.619310 -0.379156 -0.000620

C -4.130436 -0.207461 -0.072016

C -4.802475 -1.403890 -0.745149

C -6.315920 -1.235433 -0.831821

C 3.911568 -0.566583 0.036824

H 0.148497 2.046200 -0.976714

H 2.552437 1.400380 -1.315314

H 2.169130 -1.588498 1.737928

H -0.215379 -0.942092 2.087989

H -2.174097 -0.473176 -0.996954

H -2.330309 -1.244649 0.605713

H -4.537119 -0.078504 0.939294

H -4.357519 0.707668 -0.631224

H -4.560864 -2.315957 -0.185851

H -4.383628 -1.532252 -1.750418

H -6.756090 -1.128862 0.165073

H -6.577705 -0.342217 -1.408196

C 5.929852 -0.244559 -1.097944

H 5.996764 -1.280741 -1.436525

H 6.315060 0.429812 -1.860869

H 6.491560 -0.129150 -0.168823

H -6.785977 -2.096251 -1.315489

O -2.104095 2.242787 -0.172461

**Tf_2_O**

M062x/BSI SCF energy: -1847.151554 a.u.

M062x/BSII SCF energy in solution: -1847.550518 a.u.

M062x/BSII free energy in solution: -1847.516397 a.u.

S 1.546831 -0.813331 0.418371

O 1.707822 -1.767285 -0.643703

O 2.128769 -0.946737 1.722148

S -1.191077 -0.636198 -0.508150

O -0.588262 -0.002391 -1.652725

O -1.825740 -1.922634 -0.557624

O -0.078533 -0.633769 0.723979

C -2.329307 0.560817 0.336121

C 1.958649 0.899781 -0.230956

F -3.377839 0.706171 -0.447598

F -1.702439 1.706319 0.491588

F -2.681751 0.058086 1.498817

F 1.008938 1.740201 0.126274

F 2.067939 0.841114 -1.535703

F 3.105088 1.238266 0.318723

**4-ethylmorpholine**

M062x/BSI SCF energy: -366.271919 a.u.

M062x/BSII SCF energy in solution: -366.367918 a.u.

M062x/BSII free energy in solution: -366.191503 a.u.

C 0.420827 -1.291437 -0.164235

C 1.817416 -0.940522 0.321199

C 1.341478 1.327159 0.161757

C -0.069287 1.029535 -0.321888

C -1.869004 -0.588647 -0.237728

C -2.937238 0.371090 0.268810

N -0.524898 -0.247434 0.221902

O 2.242699 0.301062 -0.211211

H 0.446341 -1.419402 -1.263691

H 0.105592 -2.242836 0.278321

H 2.538283 -1.693802 -0.007269

H 1.823668 -0.899900 1.421892

H 1.710805 2.254873 -0.283179

H 1.333578 1.439844 1.257586

H -0.078302 1.014376 -1.429298

H -0.731513 1.833677 0.011521

H -1.903585 -0.636316 -1.343324

H -2.088127 -1.597924 0.129370

H -2.855993 0.494076 1.353019

H -2.862241 1.357497 -0.196427

H -3.930962 -0.023695 0.040364

**s-cis-2a**

M062x/BSI SCF energy: -210.062045 a.u.

M062x/BSII SCF energy in solution: -210.119229 a.u.

M062x/BSII free energy in solution: -210.05465 a.u.

C 1.177039 0.093226 0.000373

N 2.044805 -0.670212 -0.000356

C 0.060233 1.039022 0.000122

H 0.162598 1.686349 0.878223

H 0.162733 1.685905 -0.878399

C -1.289547 0.362069 -0.000054

C -1.500738 -0.948377 0.000255

H -2.127120 1.055302 -0.001243

H -2.510322 -1.346005 -0.000892

H -0.683447 -1.665708 0.000633

**s-trans-2a**

M062x/BSI SCF energy: -210.061237 a.u.

M062x/BSII SCF energy in solution: -210.118499 a.u.

M062x/BSII free energy in solution: -210.053874 a.u.

C 1.356701 0.054794 -0.055403

N 2.349812 -0.527582 -0.157955

C 0.086153 0.775898 0.084095

H 0.226202 1.532518 0.864206

H -0.117691 1.296611 -0.856291

C -1.045622 -0.158458 0.440579

C -2.135060 -0.292439 -0.306418

H -0.930956 -0.717128 1.366733

H -2.941442 -0.956091 -0.010357

H -2.257832 0.258397 -1.235729

**base•HOTf**

M062x/BSI SCF energy: -1328.121561 a.u.

M062x/BSII SCF energy in solution: -1328.435337 a.u.

M062x/BSII free energy in solution: -1328.219856 a.u.

C -2.225201 -0.656686 1.333855

C -1.500472 -1.952777 1.017219

C -1.703482 -1.630421 -1.267230

C -2.440586 -0.311707 -1.097068

C -2.768538 1.598637 0.493632

C -2.502522 2.656999 -0.560093

N -2.053277 0.313139 0.206878

O -1.972331 -2.510125 -0.193766

H -3.299596 -0.825109 1.445303

H -1.820504 -0.187572 2.232327

H -1.694137 -2.674914 1.812796

H -0.418622 -1.768223 0.959868

H -2.044641 -2.112254 -2.185637

H -0.623306 -1.439686 -1.344549

H -3.523834 -0.459522 -1.071381

H -2.171528 0.379999 -1.895959

H -3.830829 1.354729 0.577937

H -2.402062 1.922251 1.470487

H -1.429671 2.768590 -0.741941

H -3.001377 2.435910 -1.505719

H -2.887997 3.611180 -0.194243

H -1.046938 0.537097 0.141830

C 2.470454 -0.525440 -0.116650

S 1.394342 0.935854 0.136430

O 0.523387 0.921461 -1.059096

F 3.223401 -0.367232 -1.202730

F 1.733345 -1.629674 -0.276654

F 3.269363 -0.706568 0.932397

O 0.647489 0.606596 1.367039

O 2.332157 2.051007 0.231917

**TBSOTf**

M062x/BSI SCF energy: -1488.301482 a.u.

M062x/BSII SCF energy in solution: -1488.598508 a.u.

M062x/BSII free energy in solution: -1488.3941 a.u.

Si -1.289410 0.809546 0.051699

C -1.480700 2.254731 -1.100269

H -2.429809 2.771234 -0.921595

H -0.671559 2.976021 -0.948405

H -1.461489 1.932920 -2.145632

C -0.932616 1.328373 1.802125

H 0.038706 1.826264 1.878848

H -1.699143 2.046112 2.115557

H -0.939179 0.486436 2.498066

C -2.647957 -0.485224 -0.094423

C -3.080286 -0.627845 -1.559667

C -2.150869 -1.840825 0.427054

C -3.841520 -0.016287 0.753880

H -3.506968 0.303026 -1.948814

H -2.241359 -0.912972 -2.204836

H -3.847306 -1.407754 -1.648584

H -1.763887 -1.777598 1.450452

H -2.978383 -2.562007 0.428624

H -1.362771 -2.256172 -0.212090

H -4.672955 -0.724649 0.646919

H -3.588119 0.037801 1.818407

H -4.209078 0.968635 0.440870

C 2.653489 0.327744 -0.104816

S 1.244166 -0.840507 0.042677

O 0.150942 0.035491 -0.634517

F 2.322146 1.489331 0.445045

F 2.954631 0.512486 -1.379274

F 3.696204 -0.181381 0.532371

O 1.552161 -1.975303 -0.797462

O 0.997436 -1.012781 1.459357

**TIPSOTf**

M062x/BSI SCF energy: -1606.174817 a.u.

M062x/BSII SCF energy in solution: -1606.497284 a.u.

M062x/BSII free energy in solution: -1606.205466 a.u.

O -1.959614 -2.151509 -0.305085

O -0.338235 -0.399806 0.348553

Si 1.316862 0.077015 -0.085047

C 1.925062 0.571174 1.624205

H 3.010834 0.718147 1.532731

C 1.262530 1.447273 -1.374960

H 1.042355 0.937725 -2.323109

C 2.173780 -1.458961 -0.777384

C 3.651916 -1.497174 -0.353150

C 1.480341 -2.776045 -0.403777

H 4.194855 -0.583243 -0.615632

H 3.748159 -1.641418 0.728608

H 4.162663 -2.333925 -0.842497

H 0.467985 -2.848498 -0.810922

H 2.052799 -3.624253 -0.796448

H 1.407956 -2.902351 0.681959

H 2.138567 -1.355133 -1.871130

C 0.213509 2.548200 -1.184798

H 0.318350 3.296330 -1.979485

H -0.803759 2.156793 -1.238822

H 0.329179 3.070078 -0.230491

C 2.673454 2.053763 -1.481094

H 2.703231 2.799438 -2.283339

H 2.962233 2.562054 -0.553362

H 3.438099 1.302597 -1.706468

C 1.294240 1.883832 2.107020

H 1.614529 2.738226 1.504150

H 0.198814 1.836931 2.074078

H 1.582017 2.084603 3.145552

C 1.674218 -0.549847 2.644132

H 2.157295 -1.490481 2.361831

H 2.062537 -0.259216 3.626827

H 0.602070 -0.744013 2.756101

C -2.818221 0.253821 0.330880

S -1.562286 -0.778851 -0.529852

F -2.978959 -0.191142 1.566484

F -2.426375 1.518999 0.366711

F -3.961398 0.161501 -0.331065

O -1.416482 -0.273365 -1.880651

**n-BuSH**

M062x/BSI SCF energy: -556.529669 a.u.

M062x/BSII SCF energy in solution: -556.597838 a.u.

M062x/BSII free energy in solution: -556.4817 a.u.

S 2.351197 -0.203944 -0.071741

C 0.745931 0.668025 0.042063

C -0.460896 -0.256441 -0.043995

C -1.778865 0.512326 0.046151

C -2.990532 -0.412181 -0.026713

H 0.726571 1.251674 0.966229

H 0.752305 1.375624 -0.792530

H -0.421561 -0.819918 -0.983860

H -0.412305 -0.992528 0.769250

H -1.825211 1.248728 -0.765804

H -1.802208 1.080428 0.984350

H -3.005098 -0.964305 -0.972133

H -2.972139 -1.145852 0.785965

H -3.926955 0.147928 0.048450

H 2.173625 -0.989050 1.002898

**IM1**

M062x/BSI SCF energy: -2186.339786 a.u.

M062x/BSII SCF energy in solution: -2186.786171 a.u.

M062x/BSII free energy in solution: -2186.441374 a.u.

S 1.637139 -1.065834 0.995577

O -4.464900 -2.236876 -1.484068

O -4.894269 -2.664595 0.678137

C -0.064341 -1.423011 0.580190

C -0.904222 -1.745124 1.642608

C -2.230294 -2.048661 1.357375

C -2.688592 -2.004491 0.041935

C -1.831384 -1.661347 -1.006552

C -0.496723 -1.377523 -0.744114

C 2.539661 -2.489240 0.312006

C 4.034772 -2.327753 0.551636

C 4.795009 -3.513789 -0.045714

C 6.295919 -3.394029 0.196830

C -4.129853 -2.337383 -0.199593

H -0.542311 -1.751882 2.665913

H -2.919429 -2.309548 2.153049

H -2.202546 -1.623159 -2.024046

H 0.178595 -1.112725 -1.552631

H 2.121165 -3.353485 0.838206

H 2.274999 -2.538269 -0.748853

H 4.385088 -1.397643 0.090776

H 4.233081 -2.265162 1.627960

H 4.592939 -3.564890 -1.121846

H 4.418133 -4.444497 0.393773

H 6.691380 -2.473025 -0.243037

H 6.519838 -3.377684 1.268088

C -5.830402 -2.551808 -1.789833

H -6.502635 -1.906018 -1.222040

H -5.942988 -2.375657 -2.857800

H -6.038499 -3.596647 -1.550528

H 6.833255 -4.236390 -0.246516

C 1.576539 1.206079 -0.218343

N 2.164809 0.082270 -0.140001

C 2.040820 2.286595 -1.140401

H 2.340025 3.131746 -0.507190

C 3.164777 1.842132 -2.031776

C 4.346503 2.446663 -2.066411

H 2.953244 0.996343 -2.682297

H 5.125545 2.116284 -2.746141

H 4.572234 3.293401 -1.422891

C -0.905759 3.707764 0.646386

S -0.976002 1.920906 0.168190

O -1.881688 1.262362 1.065366

F -0.620745 3.789796 1.927865

F -2.089997 4.224979 0.396347

F 0.021877 4.308550 -0.073881

O -1.054441 1.817530 -1.266040

O 0.535592 1.485938 0.658174

H 1.179890 2.616608 -1.735114

**TS1**

M062x/BSI SCF energy: -2552.616117 a.u.

M062x/BSII SCF energy in solution: -2553.155657 a.u.

M062x/BSII free energy in solution: -2552.6227 a.u.

S -0.549801 2.035675 -0.845501

O -6.178006 -0.850011 1.345602

O -6.722601 -0.684223 -0.826082

C -2.149936 1.294952 -0.520927

C -2.982963 1.081142 -1.614780

C -4.224454 0.498838 -1.388272

C -4.598189 0.131109 -0.096208

C -3.742027 0.346276 0.986038

C -2.504355 0.943760 0.779722

C -0.793302 3.721731 -0.211546

C 0.491865 4.530868 -0.312790

C 0.280774 5.936952 0.252351

C 1.556238 6.769295 0.174216

C -5.942018 -0.505986 0.080534

H -2.672614 1.346976 -2.620583

H -4.905530 0.311444 -2.211158

H -4.040979 0.051169 1.985153

H -1.826062 1.115046 1.610552

H -1.596325 4.133652 -0.830944

H -1.144932 3.607467 0.818359

H 1.287516 4.026386 0.246607

H 0.808278 4.596815 -1.360675

H -0.050216 5.858812 1.294591

H -0.525233 6.433477 -0.300185

H 2.368068 6.293542 0.733604

H 1.887091 6.885435 -0.862673

C -7.445673 -1.474586 1.588212

H -7.526019 -2.399290 1.013343

H -7.470600 -1.686475 2.655489

H -8.258760 -0.800188 1.312706

H 1.401105 7.767642 0.591395

C 0.804460 0.235190 0.394684

N 0.461368 1.481744 0.374621

C 1.853437 -0.303214 1.204596

H 2.858317 -0.312436 0.365811

C 2.329470 0.529570 2.336491

C 2.817359 0.023457 3.469512

H 2.308256 1.607630 2.186109

H 3.199024 0.668451 4.254281

H 2.841856 -1.049109 3.648965

C 0.339798 -3.259063 -0.638146

S -0.749197 -1.859044 -0.123491

O -1.866897 -1.857580 -1.026186

F 0.647232 -3.117171 -1.913014

F -0.333655 -4.375448 -0.446731

F 1.440274 -3.259702 0.095204

O -0.881669 -1.876454 1.310710

O 0.220618 -0.623251 -0.566628

H 1.724414 -1.359918 1.442231

C 4.019137 -1.702786 -1.093838

C 4.450434 -2.664413 0.001080

C 5.659652 -1.014480 1.082212

C 5.253454 0.035154 0.059344

C 3.550492 0.615659 -1.621659

C 3.484302 2.058587 -1.143456

N 3.968516 -0.327246 -0.567537

O 5.721167 -2.304785 0.505704

H 4.730960 -1.751874 -1.930069

H 3.023883 -1.961582 -1.469198

H 4.528163 -3.677690 -0.399619

H 3.712972 -2.663972 0.819131

H 6.653558 -0.782777 1.471549

H 4.943782 -1.008900 1.919652

H 6.024980 0.120054 -0.720564

H 5.146834 1.002158 0.557156

H 4.232693 0.524827 -2.478669

H 2.560682 0.280883 -1.958237

H 2.936199 2.136103 -0.198226

H 4.477779 2.492041 -1.007040

H 2.962374 2.659865 -1.892867

**IM2**

M062x/BSI SCF energy: -2185.889037 a.u.

M062x/BSII SCF energy in solution: -2186.34286 a.u.

M062x/BSII free energy in solution: -2186.012409 a.u.

S 1.106183 1.357158 -0.756971

O -5.227348 0.937904 1.414482

O -5.662704 1.513483 -0.710343

C -0.670482 1.361456 -0.440115

C -1.525304 1.657312 -1.498760

C -2.895582 1.630681 -1.272634

C -3.387057 1.302492 -0.007549

C -2.514373 1.002442 1.040410

C -1.140544 1.032790 0.826231

C 1.496955 3.060318 -0.266470

C 2.977350 3.348193 -0.468049

C 3.314607 4.785180 -0.068509

C 4.795841 5.092420 -0.262667

C -4.870618 1.269409 0.172006

H -1.132519 1.890502 -2.484659

H -3.595527 1.848368 -2.072262

H -2.906840 0.741821 2.016808

H -0.439192 0.779413 1.615593

H 0.861160 3.698938 -0.888033

H 1.191874 3.147030 0.781382

H 3.566665 2.647057 0.133340

H 3.245330 3.186249 -1.519435

H 3.034796 4.944006 0.979780

H 2.708800 5.477288 -0.665486

H 5.416167 4.436666 0.356558

H 5.091864 4.944842 -1.306371

C -6.637851 0.865390 1.651272

H -7.098103 0.139087 0.978571

H -6.748123 0.548105 2.686772

H -7.099739 1.843557 1.501653

H 5.025275 6.126546 0.008523

C 2.141377 -0.743876 0.218217

N 1.782116 0.536162 0.490002

C 3.173132 -1.447379 0.722660

C 4.194377 -0.837625 1.555508

C 5.212414 -1.508776 2.107498

H 4.099798 0.234126 1.721893

H 5.956192 -1.004967 2.715742

H 5.328877 -2.580448 1.965214

C 0.404575 -3.899984 -0.584438

S -0.051145 -2.155626 -0.214525

O -1.111572 -1.802868 -1.127908

F 0.729388 -4.011382 -1.860484

F -0.639237 -4.668042 -0.317896

F 1.428304 -4.260078 0.173340

O -0.196586 -2.043399 1.217746

O 1.302028 -1.465587 -0.721755

H 3.228283 -2.509240 0.508254

**TS2**

M062x/BSI SCF energy: -2185.878388 a.u.

M062x/BSII SCF energy in solution: -2186.337656 a.u.

M062x/BSII free energy in solution: -2186.007644 a.u.

S 1.511411 -1.301664 0.805297

O -4.884950 -1.781480 -1.094535

O -5.187592 -2.030810 1.114896

C -0.254656 -1.457420 0.569595

C -1.035796 -1.630580 1.708129

C -2.409550 -1.765531 1.549693

C -2.971347 -1.715768 0.274412

C -2.170657 -1.525232 -0.853684

C -0.795386 -1.396710 -0.712343

C 2.107985 -2.840085 0.051804

C 3.629694 -2.876941 0.033813

C 4.124975 -4.190794 -0.573177

C 5.646938 -4.237383 -0.656883

C -4.456350 -1.859244 0.166017

H -0.586885 -1.651156 2.696337

H -3.055976 -1.900870 2.410072

H -2.621141 -1.469452 -1.837859

H -0.160241 -1.211319 -1.572048

H 1.677211 -3.641267 0.660892

H 1.678220 -2.873005 -0.954183

H 4.004547 -2.033414 -0.556547

H 4.019696 -2.769453 1.052913

H 3.693569 -4.309530 -1.573957

H 3.757312 -5.028534 0.031015

H 6.028569 -3.426829 -1.285896

H 6.098634 -4.133042 0.334813

C -6.301238 -1.900883 -1.271735

H -6.817541 -1.094019 -0.747785

H -6.471693 -1.827218 -2.344343

H -6.652265 -2.863581 -0.894627

H 5.989602 -5.183714 -1.083833

C 2.149581 0.971407 -0.018919

N 1.952854 -0.222288 -0.430885

C 2.973248 2.005207 0.091476

C 4.407159 1.824615 -0.124475

C 5.288085 2.823533 -0.023280

H 4.740433 0.820307 -0.377594

H 6.346730 2.654935 -0.189788

H 4.974299 3.833745 0.226154

C -0.647529 3.776369 -0.048485

S -0.649365 1.944905 -0.129092

O -1.915092 1.550970 0.473629

F -0.841826 4.186513 1.201098

F -1.614990 4.267190 -0.817481

F 0.520652 4.255100 -0.476714

O -0.422264 1.635468 -1.535214

O 0.538263 1.623459 0.757705

H 2.581827 2.984119 0.337451

**IM3**

M062x/BSI SCF energy:-1224.514701 a.u.

M062x/BSII SCF energy in solution: -1224.751108 a.u.

M062x/BSII free energy in solution: -1224.446129 a.u.

S 1.894615 -0.132142 1.040723

O -4.334194 -1.198010 -1.138806

O -4.747867 -1.085052 1.065599

C 0.194769 -0.474127 0.687936

C -0.647072 -0.530562 1.799984

C -2.007142 -0.723579 1.592446

C -2.499748 -0.851344 0.294853

C -1.645060 -0.782071 -0.809129

C -0.284104 -0.583997 -0.619861

C 2.863312 -0.935629 -0.267919

C 4.351186 -0.749662 0.010443

C 5.173809 -1.421349 -1.091294

C 6.671286 -1.254863 -0.855945

C -3.976669 -1.055581 0.134966

H -0.250705 -0.430796 2.805714

H -2.693173 -0.776538 2.430523

H -2.043339 -0.878554 -1.812346

H 0.378823 -0.522809 -1.476253

H 2.560327 -1.986922 -0.235321

H 2.563516 -0.487568 -1.218871

H 4.590210 0.318830 0.050601

H 4.605010 -1.187611 0.982232

H 4.898483 -0.989433 -2.060558

H 4.918117 -2.486558 -1.133057

H 6.949586 -0.196573 -0.836363

H 6.970892 -1.699186 0.098322

C -5.738560 -1.384526 -1.366845

H -6.295305 -0.513041 -1.016708

H -5.850219 -1.501502 -2.443002

H -6.088865 -2.277379 -0.845499

H 7.247618 -1.739726 -1.648120

C 1.187728 2.270706 0.453312

N 2.173815 1.533777 0.528280

C 0.160134 3.083016 0.353584

H -0.100990 3.656019 1.241783

C -0.613097 3.257806 -0.874984

C -1.649513 4.096955 -0.937519

H -0.311347 2.673489 -1.739787

H -2.215717 4.215867 -1.854975

H -1.954540 4.685158 -0.076350

**TS3**

M062x/BSI SCF energy: -1224.512984 a.u.

M062x/BSII SCF energy in solution: -1224.749374 a.u.

M062x/BSII free energy in solution: -1224.444874 a.u.

S 2.013718 0.221231 1.106617

O -4.150440 -1.497920 -0.717512

O -4.648533 -0.072952 0.945431

C 0.357209 -0.270531 0.915824

C -0.551705 0.399796 1.750049

C -1.914150 0.225917 1.544215

C -2.358719 -0.597172 0.513493

C -1.447347 -1.280426 -0.302478

C -0.086464 -1.107526 -0.119699

C 2.992385 -0.806167 -0.024888

C 4.465775 -0.437870 0.120450

C 5.314239 -1.264522 -0.847002

C 6.796152 -0.926251 -0.725935

C -3.837942 -0.685242 0.289272

H -0.196758 1.044654 2.549343

H -2.638083 0.741457 2.165859

H -1.808367 -1.923916 -1.096449

H 0.614895 -1.615905 -0.772126

H 2.797985 -1.846005 0.256741

H 2.624677 -0.617074 -1.037053

H 4.602899 0.629246 -0.088763

H 4.793787 -0.620979 1.149869

H 4.972369 -1.082083 -1.872549

H 5.156547 -2.330261 -0.643911

H 6.977801 0.128462 -0.954976

H 7.159731 -1.116465 0.288748

C -5.551793 -1.615400 -1.002573

H -5.956510 -0.647300 -1.304996

H -5.628320 -2.332734 -1.817390

H -6.086841 -1.976727 -0.122472

H 7.394722 -1.528101 -1.414694

C 0.954589 2.239029 -0.323157

N 2.076822 1.838376 -0.109706

C -0.345030 2.529598 -0.453552

H -0.762208 3.259682 0.238309

C -1.186183 1.928948 -1.462226

C -2.519918 2.072792 -1.417751

H -0.712675 1.300200 -2.210883

H -3.160120 1.577583 -2.141063

H -2.997572 2.685070 -0.657067

**IM4**

M062x/BSI SCF energy: -1224.551268 a.u.

M062x/BSII SCF energy in solution: -1224.788232 a.u.

M062x/BSII free energy in solution: -1224.480283 a.u.

S 2.148386 -0.251315 1.265115

O -3.580186 -1.951521 -0.879581

O -4.292077 -1.012075 1.034644

C 0.535488 -0.333809 0.768420

C -0.443408 0.064813 1.745538

C -1.750076 0.085351 1.417481

C -2.235790 -0.222471 0.049615

C -1.190151 -0.753095 -0.859498

C 0.120899 -0.778000 -0.530565

C 3.149571 -0.801907 -0.159259

C 4.623375 -0.680629 0.219304

C 5.514209 -1.136363 -0.936761

C 6.995224 -1.005652 -0.596869

C -3.487112 -1.119680 0.146022

H -0.115363 0.337075 2.743667

H -2.500856 0.368632 2.149938

H -1.505415 -1.072128 -1.848961

H 0.852165 -1.133108 -1.245465

H 2.882099 -1.837523 -0.386528

H 2.908704 -0.162623 -1.013163

H 4.856209 0.360109 0.473008

H 4.832818 -1.288615 1.107371

H 5.282106 -0.539178 -1.826579

H 5.278940 -2.178143 -1.184602

H 7.252335 0.032170 -0.362349

H 7.254690 -1.619540 0.271407

C -4.761255 -2.777346 -0.903883

H -5.654139 -2.151164 -0.934635

H -4.679228 -3.373696 -1.810005

H -4.784894 -3.417467 -0.020742

H 7.621236 -1.325683 -1.433983

C -0.337054 4.035928 -0.383856

N 0.535783 4.761343 -0.611468

C -1.413964 3.136848 -0.085321

H -1.978452 3.340796 0.820986

C -1.695907 2.105473 -0.886980

C -2.793278 1.133820 -0.590717

H -1.104281 1.937930 -1.784211

H -3.314948 0.856410 -1.511841

H -3.514911 1.546452 0.118590

**IM1’**

M062x/BSI SCF energy: -2186.335275 a.u.

M062x/BSII SCF energy in solution: -2186.781268 a.u.

M062x/BSII free energy in solution: -2186.436857 a.u.

S -1.694761 -1.025945 -0.982766

O 4.425930 -2.390261 1.339615

O 4.853167 -2.583497 -0.856301

C 0.009854 -1.410686 -0.613607

C 0.838687 -1.649870 -1.705762

C 2.168010 -1.973074 -1.458843

C 2.639850 -2.027717 -0.148818

C 1.792118 -1.772393 0.932443

C 0.455233 -1.470889 0.706681

C -2.591398 -2.420709 -0.232662

C -4.095951 -2.222912 -0.353315

C -4.830884 -3.403048 0.287868

C -6.343976 -3.235209 0.199125

C 4.086795 -2.362410 0.052431

H 0.465225 -1.580401 -2.722565

H 2.849353 -2.170656 -2.279068

H 2.173674 -1.813596 1.945942

H -0.213700 -1.273644 1.539978

H -2.236377 -3.295363 -0.787368

H -2.251792 -2.480632 0.805907

H -4.384744 -1.293097 0.148696

H -4.378492 -2.139890 -1.409274

H -4.526420 -3.488926 1.337525

H -4.528291 -4.331936 -0.209113

H -6.667589 -2.322322 0.708963

H -6.671624 -3.172947 -0.843286

C 5.797401 -2.713532 1.607602

H 6.456116 -1.972092 1.151287

H 5.898708 -2.697882 2.690969

H 6.033915 -3.704076 1.214227

H -6.859461 -4.079856 0.663672

C -1.575896 1.259879 0.232265

N -2.196899 0.159457 0.118359

C -2.008115 2.331449 1.182925

H -1.628325 3.288798 0.812785

C -3.492933 2.439446 1.436279

C -4.477145 2.035626 0.641143

H -3.730243 2.957219 2.362132

H -5.510340 2.218645 0.918614

H -4.303578 1.522466 -0.300147

C 1.117667 3.651241 -0.436783

S 1.011873 1.861548 0.045410

O 1.961595 1.155912 -0.766744

F 0.687275 3.784931 -1.672866

F 2.385547 3.993628 -0.345005

F 0.387126 4.371678 0.391315

O 0.945948 1.755526 1.480347

O -0.470726 1.502513 -0.577329

H -1.484255 2.139515 2.127778

**TS1’**

M062x/BSI SCF energy: -2552.61419 a.u.

M062x/BSII SCF energy in solution: -2553.153829 a.u.

M062x/BSII free energy in solution: -2552.62005 a.u.

S -0.493414 1.974757 -0.888224

O -6.166255 -0.741171 1.404553

O -6.738405 -0.570348 -0.759702

C -2.109360 1.283172 -0.533552

C -2.965694 1.089444 -1.613220

C -4.220182 0.545897 -1.364201

C -4.583597 0.193742 -0.064758

C -3.703829 0.386913 1.002457

C -2.452822 0.947627 0.773948

C -0.681078 3.684975 -0.303034

C 0.622248 4.455810 -0.459168

C 0.459029 5.883765 0.065014

C 1.750083 6.683269 -0.075777

C -5.940623 -0.407729 0.134823

H -2.663363 1.340290 -2.625286

H -4.919924 0.375697 -2.175004

H -3.994437 0.103896 2.007492

H -1.757082 1.101824 1.593630

H -1.485858 4.098512 -0.919382

H -1.012976 3.614699 0.737444

H 1.420199 3.949243 0.095626

H 0.915286 4.480846 -1.515509

H 0.155237 5.846898 1.117636

H -0.350807 6.380461 -0.481779

H 2.565910 6.208826 0.478891

H 2.054802 6.754025 -1.124724

C -7.441829 -1.341586 1.666009

H -7.544254 -2.268808 1.098649

H -7.458417 -1.545644 2.734965

H -8.246326 -0.655348 1.394634

H 1.628586 7.699133 0.308808

C 0.811189 0.174201 0.403240

N 0.509800 1.428061 0.343413

C 1.809280 -0.395622 1.257428

H 2.846181 -0.415260 0.482197

C 2.275677 0.321895 2.474333

C 2.210675 1.622010 2.773878

H 2.771493 -0.341361 3.181570

H 2.633302 1.976027 3.709149

H 1.738408 2.354762 2.130042

C 0.292667 -3.312522 -0.680431

S -0.773081 -1.900981 -0.147322

O -1.882534 -1.858583 -1.059590

F 0.653319 -3.126320 -1.935411

F -0.420234 -4.414278 -0.564015

F 1.362710 -3.377549 0.093826

O -0.921568 -1.940596 1.284878

O 0.230882 -0.682660 -0.563227

H 1.642109 -1.458206 1.431579

C 4.042442 -1.814727 -0.953817

C 4.407389 -2.783079 0.159283

C 5.599485 -1.147752 1.288053

C 5.264769 -0.098347 0.239239

C 3.619689 0.511423 -1.489861

C 3.563383 1.952989 -1.005594

N 3.996896 -0.439392 -0.429310

O 5.658259 -2.443735 0.724492

H 4.787286 -1.879975 -1.760123

H 3.058573 -2.055422 -1.369026

H 4.489562 -3.797287 -0.238173

H 3.630639 -2.773005 0.939922

H 6.578890 -0.937338 1.723425

H 4.846435 -1.120675 2.091928

H 6.069485 -0.042328 -0.509650

H 5.161874 0.875991 0.722895

H 4.320285 0.413777 -2.331402

H 2.633310 0.191692 -1.850673

H 3.020713 2.027760 -0.056910

H 4.560571 2.378397 -0.870155

H 3.042208 2.562269 -1.749256

**IM2’**

M062x/BSI SCF energy: -2185.887882 a.u.

M062x/BSII SCF energy in solution: -2186.339987 a.u.

M062x/BSII free energy in solution: -2186.00588 a.u.

S -0.437175 -1.364116 -0.566706

O 5.625204 1.111459 0.917576

O 6.005067 0.612286 -1.236852

C 1.260496 -0.756417 -0.447651

C 2.059147 -0.819782 -1.587198

C 3.366737 -0.359176 -1.506893

C 3.850167 0.163202 -0.305055

C 3.028842 0.230392 0.821990

C 1.718121 -0.231246 0.753466

C -0.116248 -3.142517 -0.381134

C -1.416747 -3.929900 -0.441395

C -1.161667 -5.423097 -0.231698

C -2.453854 -6.231435 -0.291299

C 5.264455 0.644241 -0.279546

H 1.670857 -1.214262 -2.522095

H 4.020625 -0.391247 -2.371722

H 3.412029 0.643637 1.748169

H 1.053464 -0.181169 1.611617

H 0.567265 -3.405882 -1.194386

H 0.395929 -3.253831 0.579984

H -2.098047 -3.559111 0.332566

H -1.901630 -3.770009 -1.412169

H -0.674769 -5.571369 0.739480

H -0.461143 -5.783990 -0.994342

H -3.159578 -5.896907 0.475712

H -2.941521 -6.119834 -1.265079

C 6.976157 1.577812 1.013197

H 7.148105 2.389836 0.303811

H 7.095353 1.934183 2.034868

H 7.673202 0.762334 0.809104

H -2.262883 -7.295891 -0.130618

C -2.010485 -0.032159 0.962637

N -1.132488 -1.055095 0.875692

C -2.425457 0.599274 2.082381

C -1.884496 0.396538 3.419496

C -1.015379 -0.535000 3.842747

H -2.253613 1.112657 4.152382

H -0.694509 -0.541549 4.880008

H -0.625674 -1.301979 3.184288

C -2.832791 3.043543 -0.291916

S -2.117921 1.554547 -1.127705

O -2.756719 1.471526 -2.417829

F -4.077080 2.787656 0.078110

F -2.820999 4.031244 -1.171727

F -2.107959 3.375144 0.759909

O -0.681680 1.639426 -0.978905

O -2.705058 0.360570 -0.251979

H -3.236663 1.308993 1.973659

**TS2’**

M062x/BSI SCF energy: -2185.875958 a.u.

M062x/BSII SCF energy in solution: -2186.335133 a.u.

M062x/BSII free energy in solution: -2186.005152 a.u.

S 1.566941 -1.100314 0.817462

O -4.729261 -2.189226 -1.169241

O -5.038579 -2.424533 1.040775

C -0.175859 -1.400014 0.558115

C -0.956479 -1.628027 1.687336

C -2.309743 -1.888223 1.509791

C -2.852716 -1.904633 0.225551

C -2.055253 -1.651808 -0.893044

C -0.699129 -1.398311 -0.732196

C 2.306958 -2.586741 0.087575

C 3.822947 -2.536169 0.220340

C 4.470186 -3.727622 -0.486434

C 5.988817 -3.704009 -0.348253

C -4.314153 -2.197353 0.098234

H -0.523687 -1.597926 2.682527

H -2.954578 -2.074805 2.361674

H -2.492828 -1.651420 -1.884629

H -0.065744 -1.172705 -1.583712

H 1.867377 -3.429603 0.630059

H 1.973775 -2.605150 -0.955142

H 4.198706 -1.601951 -0.214168

H 4.099880 -2.539491 1.281118

H 4.191331 -3.711705 -1.546551

H 4.070995 -4.658871 -0.067556

H 6.405445 -2.786185 -0.775823

H 6.287017 -3.747431 0.704134

C -6.122696 -2.463524 -1.359136

H -6.728710 -1.709173 -0.853192

H -6.287999 -2.426220 -2.434359

H -6.370414 -3.452396 -0.967823

H 6.446816 -4.552814 -0.862991

C 2.069250 1.201142 -0.016703

N 1.935849 -0.001987 -0.426396

C 2.834945 2.277801 0.093581

C 4.281962 2.211222 -0.158113

C 4.911209 1.284567 -0.886308

H 4.855487 3.013002 0.301802

H 5.989768 1.310150 -1.001825

H 4.369822 0.493812 -1.398537

C -0.884862 3.831141 0.034895

S -0.780621 2.004130 -0.070165

O -2.012308 1.527875 0.543089

F -1.093984 4.214570 1.290546

F -1.883769 4.275370 -0.722178

F 0.250883 4.381579 -0.394064

O -0.555123 1.728429 -1.483282

O 0.434955 1.742279 0.798314

H 2.387543 3.214829 0.395647

**IM3’**

M062x/BSI SCF energy: -1224.512246 a.u.

M062x/BSII SCF energy in solution: -1224.747498 a.u.

M062x/BSII free energy in solution: -1224.440264 a.u.

S -1.004064 -0.146737 1.055516

O 5.244146 -0.012109 -1.298957

O 5.705494 0.223309 0.885489

C 0.731283 -0.100358 0.684488

C 1.597115 0.037574 1.766512

C 2.962005 0.101093 1.508473

C 3.426962 0.015092 0.197657

C 2.540033 -0.134624 -0.872761

C 1.173965 -0.190346 -0.634483

C -1.678649 1.259050 0.105240

C -3.170290 1.407523 0.368634

C -3.775012 2.460338 -0.562550

C -5.273627 2.611444 -0.324671

C 4.909020 0.087123 -0.014257

H 1.221631 0.091154 2.783499

H 3.673772 0.209875 2.319201

H 2.917314 -0.206865 -1.886098

H 0.471832 -0.316223 -1.451998

H -1.102988 2.125870 0.445935

H -1.444494 1.051695 -0.944113

H -3.689316 0.456493 0.196006

H -3.335507 1.688334 1.414320

H -3.591027 2.166475 -1.602860

H -3.268418 3.419960 -0.408365

H -5.788405 1.657158 -0.479544

H -5.478228 2.940285 0.698850

C 6.650210 0.059971 -1.573139

H 7.174372 -0.755402 -1.071074

H 6.746588 -0.032165 -2.653040

H 7.052084 1.016351 -1.233180

H -5.707929 3.345257 -1.008351

C -2.609150 -1.929197 0.174382

N -1.481330 -1.453218 -0.009873

C -3.787318 -2.499798 0.261624

C -4.976338 -2.041179 -0.473438

C -4.946995 -1.222650 -1.528333

H -5.918930 -2.431177 -0.102145

H -5.864107 -0.921237 -2.023037

H -4.010829 -0.849865 -1.939803

H -3.859489 -3.351583 0.934119

**TS3’**

M062x/BSI SCF energy: -1224.50525 a.u.

M062x/BSII SCF energy in solution: -1224.741314 a.u.

M062x/BSII free energy in solution: -1224.435805 a.u.

S 1.830667 0.132659 0.964326

O -4.343253 -1.903461 -0.561029

O -4.858584 -0.011102 0.533314

C 0.155280 -0.253112 0.704048

C -0.758220 0.710487 1.164570

C -2.122421 0.509636 0.963318

C -2.563470 -0.623885 0.291737

C -1.645062 -1.566299 -0.194307

C -0.287110 -1.383617 -0.001308

C 2.782648 -1.162601 0.120467

C 4.266171 -0.822048 0.225903

C 5.108067 -1.883240 -0.483156

C 6.599315 -1.581270 -0.379834

C -4.039075 -0.794988 0.111633

H -0.411243 1.586505 1.706988

H -2.844803 1.231846 1.328215

H -2.000649 -2.444407 -0.721382

H 0.413124 -2.122078 -0.372894

H 2.552671 -2.109326 0.618247

H 2.445018 -1.196271 -0.919756

H 4.452883 0.159416 -0.224565

H 4.559071 -0.764316 1.280760

H 4.809335 -1.935081 -1.536764

H 4.895617 -2.865196 -0.043921

H 6.834314 -0.611389 -0.829407

H 6.921010 -1.553265 0.666022

C -5.744201 -2.141328 -0.755468

H -6.196662 -1.313470 -1.304609

H -5.809929 -3.062554 -1.331187

H -6.243820 -2.257384 0.208371

H 7.191974 -2.342938 -0.893236

C 1.009774 1.965967 -1.011175

N 2.081369 1.499653 -0.764296

C -0.258622 2.409508 -1.185613

H -0.886742 1.808361 -1.840344

C -0.764433 3.642540 -0.635823

C -0.022237 4.469645 0.122311

H -1.799832 3.872540 -0.863324

H -0.446783 5.382854 0.525091

H 1.021471 4.260162 0.346438

**IM4’**

M062x/BSI SCF energy: -1224.537683 a.u.

M062x/BSII SCF energy in solution: -1224.774461 a.u.

M062x/BSII free energy in solution: -1224.465598 a.u.

S 1.995267 0.381836 0.591413

O -3.878548 -2.450267 -0.391883

O -4.593892 -0.688530 0.808524

C 0.411104 0.003491 0.166899

C -0.644903 0.992438 0.566526

C -2.004377 0.401308 0.688157

C -2.311795 -0.783863 0.121818

C -1.288991 -1.527726 -0.563442

C 0.031205 -1.157439 -0.540192

C 3.023828 -0.952157 -0.113725

C 4.473498 -0.713402 0.298998

C 5.373012 -1.820040 -0.252773

C 6.830440 -1.618574 0.148496

C -3.719396 -1.284824 0.226411

H -0.374450 1.532360 1.480106

H -2.773011 0.988259 1.182272

H -1.569436 -2.439942 -1.081554

H 0.771393 -1.799806 -1.000219

H 2.652127 -1.902707 0.278559

H 2.909656 -0.929581 -1.201001

H 4.813918 0.258905 -0.075383

H 4.550355 -0.690154 1.392314

H 5.286732 -1.842619 -1.345383

H 5.016016 -2.790119 0.113233

H 7.209949 -0.660875 -0.221397

H 6.941743 -1.625352 1.237388

C -5.203165 -3.004459 -0.335457

H -5.919350 -2.309794 -0.777515

H -5.160604 -3.928451 -0.908179

H -5.478720 -3.208076 0.700906

H 7.464280 -2.410695 -0.258725

C 0.490331 2.826948 -0.743570

N 1.494159 3.385012 -0.868873

C -0.782648 2.121376 -0.577403

H -0.998859 1.596027 -1.514558

C -1.904645 3.085089 -0.248194

C -1.727496 4.289324 0.282612

H -2.900383 2.713514 -0.475108

H -2.580251 4.924363 0.498869

H -0.740986 4.685707 0.510779

**5**

M062x/BSI SCF energy: -1224.165581 a.u.

M062x/BSII SCF energy in solution: -1224.406441 a.u.

M062x/BSII free energy in solution: -1224.112097 a.u.

S -2.074107 0.564670 0.088352

O 3.831402 -2.650959 -0.169447

O 4.604533 -0.562539 0.111254

C -0.414883 -0.052098 0.078081

C 0.639312 0.876602 0.221872

C 1.951848 0.427242 0.185806

C 2.245055 -0.927613 0.027690

C 1.204339 -1.845245 -0.102004

C -0.114517 -1.410038 -0.079997

C -3.095143 -0.941681 0.015632

C -4.565055 -0.543142 0.114150

C -5.477672 -1.766354 0.027566

C -6.951438 -1.389084 0.137809

C 3.676188 -1.333153 -0.001591

H 2.771466 1.132733 0.281727

H 1.420600 -2.900644 -0.225767

H -0.906370 -2.140285 -0.192540

H -2.902489 -1.459370 -0.928662

H -2.818601 -1.598049 0.845949

H -4.742913 -0.019125 1.061092

H -4.815194 0.156068 -0.693149

H -5.213783 -2.470965 0.825649

H -5.296606 -2.284361 -0.922121

H -7.158408 -0.894586 1.092407

H -7.239955 -0.701350 -0.663673

C 5.186209 -3.108516 -0.220707

H 5.696237 -2.899064 0.722239

H 5.130482 -4.182667 -0.390417

H 5.722230 -2.621480 -1.037837

H -7.594594 -2.270877 0.070696

C -0.010948 2.961406 -0.901109

N -0.272698 3.428670 -1.925387

C 0.346442 2.367729 0.398931

H -0.540804 2.470444 1.039908

C 1.474232 3.127556 1.064447

C 2.155666 4.132383 0.527493

H 1.700136 2.775439 2.068578

H 2.944668 4.624852 1.086551

H 1.953190 4.499900 -0.475416

**3a**

M062x/BSI SCF energy: -834.039153 a.u.

M062x/BSII SCF energy in solution: -834.207544 a.u.

M062x/BSII free energy in solution: -833.923359 a.u.

C 1.912001 2.385943 -0.674760

C 1.639377 1.093219 -0.482167

H 1.140450 3.051302 -1.038603

H 2.903676 2.766570 -0.469909

C 2.494115 -1.122122 -0.342176

C 3.696005 -1.812998 0.261183

H 1.563006 -1.566004 0.024361

H 2.498586 -1.196608 -1.436539

H 3.682505 -2.874934 0.002839

H 4.624377 -1.374690 -0.113487

H 3.680867 -1.720924 1.350661

O 2.565444 0.255438 0.045363

O 0.444712 0.523671 -0.768422

Si -0.796741 0.502808 0.403229

C -1.501507 2.230146 0.540477

H -2.411963 2.234939 1.150000

H -0.784427 2.910329 1.011343

H -1.754859 2.634227 -0.444742

C -0.054999 -0.041793 2.033174

H 0.823108 0.565427 2.278492

H -0.779877 0.083455 2.844649

H 0.256577 -1.091310 2.017156

C -2.044808 -0.733007 -0.288609

C -2.698169 -0.150092 -1.548988

C -1.329213 -2.042514 -0.646408

C -3.123712 -1.013099 0.766757

H -3.261229 0.763655 -1.328240

H -1.953250 0.088437 -2.316719

H -3.400812 -0.874916 -1.981515

H -0.851545 -2.498320 0.229260

H -2.050184 -2.770137 -1.042569

H -0.558929 -1.884300 -1.408747

H -3.866653 -1.716837 0.368206

H -2.700467 -1.460954 1.673283

H -3.660373 -0.102685 1.059187

**(3a)IM5**

M062x/BSI SCF energy: -2058.61083 a.u.

M062x/BSII SCF energy in solution: -2059.015115 a.u.

M062x/BSII free energy in solution: -2058.406635 a.u.

S 0.148792 2.616552 0.829122

O -2.840626 -2.830064 -0.706892

O -3.956589 -2.378101 1.190868

C -0.961138 1.394487 0.454460

C -2.008698 1.180652 1.414619

C -2.933508 0.223659 1.200420

C -3.008174 -0.546456 -0.065264

C -1.834058 -0.355051 -0.952444

C -0.885954 0.582440 -0.723735

C 1.494905 2.431413 -0.386924

C 2.641556 3.355684 0.010416

C 3.797338 3.237994 -0.983423

C 4.953478 4.164025 -0.620429

C -3.308306 -2.024945 0.239717

H -2.007854 1.758067 2.333063

H -3.696184 0.017143 1.945838

H -1.794925 -0.951972 -1.859185

H -0.076836 0.716370 -1.431476

H 1.802734 1.380608 -0.356487

H 1.112689 2.678239 -1.381639

H 2.290561 4.394087 0.043649

H 2.993219 3.099338 1.018058

H 3.431438 3.473150 -1.990303

H 4.145686 2.197913 -1.009633

H 4.628762 5.209325 -0.609248

H 5.349245 3.925550 0.371927

C -3.120793 -4.229108 -0.519668

H -4.198777 -4.398217 -0.530914

H -2.643845 -4.738428 -1.354475

H -2.702931 -4.568895 0.429932

H 5.771818 4.074027 -1.339692

C -4.558313 3.707874 -1.331989

N -4.428475 4.777409 -1.755277

C -4.721276 2.386840 -0.797275

H -5.310972 2.301326 0.111721

C -4.170902 1.326297 -1.396550

C -4.308804 -0.063402 -0.861494

H -3.587709 1.461362 -2.304956

H -4.454041 -0.770033 -1.683963

H -5.151834 -0.143227 -0.170340

C -0.522584 -2.206073 1.528351

C 0.417263 -1.250019 1.495772

H -0.615705 -2.924088 0.724359

H -1.173129 -2.279013 2.389702

C 1.739291 0.236008 2.795340

C 1.558072 1.074483 4.038919

H 2.444138 -0.587120 2.962431

H 2.116176 0.839971 1.961076

H 2.512616 1.523472 4.325011

H 0.837626 1.877823 3.859183

H 1.197812 0.462514 4.869511

O 0.451798 -0.303536 2.459105

O 1.337428 -1.054078 0.535048

Si 1.966197 -2.236533 -0.537654

C 0.699664 -2.570138 -1.876448

H 1.098673 -3.294029 -2.596263

H -0.234779 -2.979194 -1.478233

H 0.461569 -1.654865 -2.428539

C 2.380241 -3.766795 0.452846

H 1.508680 -4.158659 0.983422

H 2.754039 -4.554166 -0.209820

H 3.157768 -3.553760 1.194357

C 3.524397 -1.427837 -1.242725

C 3.176357 -0.461023 -2.382854

C 4.265210 -0.672285 -0.130584

C 4.438377 -2.532244 -1.798142

H 2.735255 -0.988114 -3.235862

H 2.471445 0.319029 -2.070742

H 4.085232 0.042248 -2.739424

H 4.515158 -1.327597 0.712576

H 5.206661 -0.260220 -0.518035

H 3.667865 0.158930 0.261427

H 5.319597 -2.082955 -2.274474

H 4.794146 -3.202160 -1.008449

H 3.932866 -3.141673 -2.557467

**(3a)TS4A**

M062x/BSI SCF energy: -2058.604355 a.u.

M062x/BSII SCF energy in solution: -2059.007497 a.u.

M062x/BSII free energy in solution: -2058.399202 a.u.

S 2.630280 -1.199126 0.577503

O -0.480754 3.695975 -2.332825

O -1.809950 3.715031 -0.523761

C 1.691066 0.140131 0.054458

C 2.104522 1.021146 -1.019157

C 1.446796 2.176559 -1.236946

C 0.315987 2.608049 -0.364586

C -0.289198 1.461687 0.377568

C 0.482558 0.374810 0.728370

C 4.093672 -1.238477 -0.514374

C 4.927677 -2.462105 -0.144746

C 6.185200 -2.544699 -1.009955

C 7.029236 -3.768907 -0.670530

C -0.786710 3.394323 -1.078305

H 2.964868 0.766961 -1.625831

H 1.792435 2.866934 -2.000125

H -1.217439 1.664030 0.904757

H 0.113404 -0.343068 1.455303

H 4.662773 -0.316136 -0.368680

H 3.762137 -1.301438 -1.554689

H 4.328693 -3.371294 -0.278988

H 5.213630 -2.414600 0.912810

H 5.894698 -2.573331 -2.067144

H 6.777604 -1.632630 -0.869842

H 6.464647 -4.692203 -0.835204

H 7.340727 -3.750101 0.378700

C -1.479565 4.444045 -3.049770

H -2.411783 3.877301 -3.088768

H -1.075132 4.587016 -4.049530

H -1.649475 5.404964 -2.561347

H 7.930880 -3.812097 -1.287278

C 3.037964 2.547013 3.643755

N 3.935665 2.166838 4.268428

C 1.919979 3.013391 2.876013

H 1.020617 3.265118 3.431145

C 1.998009 3.127546 1.546278

C 0.868403 3.641246 0.714615

H 2.924764 2.865268 1.040094

H 0.022873 3.935863 1.341119

H 1.206149 4.520047 0.151904

C -1.659640 0.710930 -1.612123

C -1.465798 -0.573186 -1.195115

H -2.541734 1.263568 -1.318997

H -1.049401 1.074426 -2.429589

C -0.205119 -2.600669 -1.251762

C 0.913706 -3.142841 -2.108566

H -1.137812 -3.144830 -1.420723

H 0.036237 -2.647243 -0.184068

H 1.118480 -4.178847 -1.827375

H 1.829621 -2.561423 -1.972505

H 0.637956 -3.116039 -3.165538

O -0.390236 -1.220045 -1.630649

O -2.176471 -1.221366 -0.292834

Si -3.794481 -0.920246 0.245004

C -3.802064 0.652971 1.254525

H -4.770129 0.760129 1.757472

H -3.647368 1.551606 0.648808

H -3.030932 0.622035 2.031568

C -4.875443 -0.817210 -1.272081

H -4.511782 -0.063431 -1.976450

H -5.897836 -0.543624 -0.991499

H -4.915235 -1.777067 -1.796782

C -4.160331 -2.438368 1.298413

C -3.360724 -2.382508 2.607655

C -3.787738 -3.713910 0.530844

C -5.663531 -2.455395 1.616028

H -3.642294 -1.519993 3.221427

H -2.280823 -2.331837 2.423268

H -3.551770 -3.286425 3.200484

H -4.309706 -3.782032 -0.430751

H -4.065148 -4.597837 1.119689

H -2.710987 -3.765209 0.338078

H -5.898544 -3.306845 2.267527

H -6.268283 -2.558045 0.708192

H -5.985167 -1.546231 2.137763

**(3a)TS4B**

M062x/BSI SCF energy: -2058.60756 a.u.

M062x/BSII SCF energy in solution: -2059.010904 a.u.

M062x/BSII free energy in solution: -2058.402175 a.u.

S 0.184177 2.756525 0.901825

O -3.496405 -2.522236 -0.713807

O -4.419852 -1.867402 1.225704

C -0.936880 1.510159 0.548185

C -2.096441 1.451686 1.418085

C -3.081188 0.569375 1.180222

C -3.070618 -0.300099 -0.027405

C -1.715900 -0.425771 -0.637978

C -0.778471 0.575560 -0.487300

C 1.615955 2.389459 -0.165990

C 2.775544 3.296735 0.233574

C 4.029956 2.966689 -0.575628

C 5.192968 3.888914 -0.224664

C -3.733666 -1.647966 0.259031

H -2.142392 2.120290 2.272043

H -3.953491 0.514890 1.824077

H -1.606086 -1.146996 -1.442553

H 0.111117 0.565547 -1.105594

H 1.874879 1.334209 -0.016634

H 1.332149 2.546890 -1.210612

H 2.498235 4.345617 0.074631

H 2.988889 3.175833 1.302990

H 3.802322 3.042653 -1.646133

H 4.311782 1.922923 -0.387445

H 4.942328 4.932989 -0.437180

H 5.444510 3.814964 0.838270

C -4.089240 -3.820360 -0.537212

H -5.175676 -3.732027 -0.484586

H -3.793787 -4.399216 -1.409881

H -3.711671 -4.281336 0.377743

H 6.087110 3.634299 -0.799840

C -3.325924 4.075093 -1.694784

N -2.877752 5.064887 -2.094726

C -3.878790 2.855951 -1.180638

H -4.610676 2.954005 -0.383191

C -3.497800 1.667159 -1.660632

C -4.026864 0.375737 -1.127350

H -2.762079 1.621207 -2.459858

H -4.136279 -0.350745 -1.936766

H -4.997674 0.516811 -0.643717

C -0.911816 -2.283930 0.951117

C 0.169144 -1.513486 1.252310

H -0.884115 -2.986018 0.129900

H -1.702187 -2.357234 1.687830

C 1.307994 -0.093475 2.799442

C 0.932883 0.640726 4.064079

H 2.037685 -0.886906 2.988288

H 1.727184 0.586759 2.051264

H 1.819014 1.125930 4.480586

H 0.182998 1.408862 3.855323

H 0.529700 -0.050802 4.807642

O 0.094966 -0.683486 2.289828

O 1.270030 -1.383762 0.532264

Si 1.927409 -2.477362 -0.636760

C 0.787642 -2.542253 -2.118509

H 1.317040 -3.009741 -2.957246

H -0.115797 -3.132458 -1.937872

H 0.491283 -1.536843 -2.437739

C 2.110724 -4.128450 0.210363

H 1.159252 -4.467836 0.631031

H 2.451426 -4.886160 -0.503014

H 2.842132 -4.077020 1.023588

C 3.580405 -1.674038 -1.061065

C 3.384472 -0.552632 -2.091043

C 4.220314 -1.100291 0.210188

C 4.503257 -2.747504 -1.660238

H 2.991546 -0.936801 -3.038623

H 2.696588 0.222663 -1.731693

H 4.345378 -0.065889 -2.304241

H 4.357508 -1.867447 0.981738

H 5.210144 -0.685241 -0.021528

H 3.607118 -0.300225 0.641292

H 5.454786 -2.293390 -1.965109

H 4.729905 -3.538565 -0.937536

H 4.065734 -3.216822 -2.550010

**(3a)TS4C**

M062x/BSI SCF energy: -2058.603632 a.u.

M062x/BSII SCF energy in solution: -2059.006695 a.u.

M062x/BSII free energy in solution: -2058.398622 a.u.

S -2.892990 -1.198804 -0.874747

O 1.275406 2.979496 1.992405

O 2.233853 2.621844 -0.006958

C -1.648047 -0.163433 -0.292591

C -0.829664 0.444031 -1.325539

C -0.091833 1.534312 -1.061854

C -0.150647 2.251437 0.246602

C -0.932039 1.520763 1.293311

C -1.662972 0.423012 1.028790

C -3.745794 -1.789092 0.623526

C -4.774378 -2.841882 0.220367

C -5.539024 -3.349050 1.442822

C -6.573507 -4.406294 1.071495

C 1.267655 2.609696 0.713883

H -0.822371 -0.005302 -2.314329

H 0.526004 1.984430 -1.834065

H -0.956132 1.960262 2.286373

H -2.248377 -0.041090 1.813662

H -2.994198 -2.213347 1.297036

H -4.230648 -0.939570 1.111010

H -5.481400 -2.414676 -0.501093

H -4.271779 -3.680719 -0.275232

H -6.032526 -2.502471 1.934958

H -4.827079 -3.763074 2.166933

H -7.305668 -4.005974 0.362969

H -6.097134 -5.274464 0.604974

C 2.530684 3.469828 2.493867

H 2.828234 4.361486 1.938973

H 2.354441 3.711327 3.540066

H 3.305661 2.706121 2.399733

H -7.116430 -4.755343 1.953940

C -3.941513 3.872283 -2.194428

N -5.024663 3.768133 -2.589759

C -2.594263 4.005686 -1.721145

H -1.897066 4.511811 -2.383193

C -2.228869 3.528915 -0.527153

C -0.836779 3.658494 0.001701

H -2.962641 3.025507 0.099374

H -0.850814 4.181476 0.964333

H -0.212339 4.225304 -0.695684

C -0.026240 -1.971484 0.347109

C 0.979041 -1.131279 0.722919

H -0.062231 -2.365175 -0.657641

H -0.637285 -2.420076 1.120017

C 2.218129 -0.105482 2.488706

C 1.920323 0.406064 3.877745

H 2.984525 -0.889000 2.506068

H 2.558888 0.686620 1.818011

H 2.827474 0.837950 4.308368

H 1.145846 1.177331 3.846366

H 1.584616 -0.407787 4.525010

O 0.995957 -0.678459 1.977228

O 1.862970 -0.575073 -0.080533

Si 2.704641 -1.148431 -1.477737

C 3.257005 0.423744 -2.303373

H 4.015369 0.208371 -3.064265

H 2.416696 0.915031 -2.804261

H 3.677165 1.127436 -1.580229

C 1.605561 -2.155217 -2.606946

H 0.637771 -1.676303 -2.784859

H 2.109646 -2.237629 -3.577181

H 1.427796 -3.170339 -2.240198

C 4.135029 -2.177573 -0.804621

C 5.135989 -1.261320 -0.087426

C 3.615923 -3.234795 0.179531

C 4.830465 -2.880903 -1.980341

H 5.586270 -0.536270 -0.774217

H 4.660333 -0.703407 0.728349

H 5.949768 -1.856499 0.347022

H 2.862520 -3.889237 -0.274029

H 4.445633 -3.871110 0.514513

H 3.172632 -2.780650 1.073582

H 5.699157 -3.444547 -1.615910

H 4.163475 -3.591256 -2.481642

H 5.194829 -2.168442 -2.729916

**(3a)TS4a**

M062x/BSI SCF energy: -2058.597309 a.u.

M062x/BSII SCF energy in solution: -2059.001076 a.u.

M062x/BSII free energy in solution: -2058.394886 a.u.

S 1.359596 2.608776 0.781102

O 2.933049 -1.297267 2.028487

O 1.706358 -3.151135 2.338683

C 1.277692 0.944762 0.377632

C 2.227628 0.299660 -0.499884

C 2.279179 -1.045316 -0.551573

C 1.380795 -1.904633 0.273465

C 0.157269 -1.155370 0.719674

C 0.229055 0.199471 0.947672

C 2.809730 3.260094 -0.116384

C 2.946942 4.742786 0.216545

C 4.152843 5.351654 -0.498464

C 4.318753 6.832204 -0.172936

C 2.039270 -2.202790 1.675138

H 2.916256 0.898063 -1.083571

H 3.028883 -1.541449 -1.163242

H -0.633766 -1.740623 1.181236

H -0.565607 0.707951 1.486410

H 3.695263 2.702763 0.201149

H 2.657936 3.117968 -1.190263

H 2.035415 5.275564 -0.081242

H 3.059214 4.871004 1.299762

H 4.034735 5.218738 -1.580519

H 5.057453 4.802910 -0.210256

H 3.429727 7.399731 -0.465918

H 4.472498 6.982323 0.900424

C 3.508855 -1.476535 3.337124

H 4.013721 -2.442255 3.392744

H 4.220726 -0.662647 3.455488

H 2.728162 -1.420912 4.097721

H 5.177780 7.259152 -0.697444

C 3.937751 -5.348175 -1.840853

N 4.887238 -6.001745 -1.949620

C 2.762517 -4.533981 -1.724259

H 2.251160 -4.289080 -2.651326

C 2.323578 -4.113597 -0.533000

C 1.090493 -3.278104 -0.374046

H 2.867986 -4.394065 0.366813

H 0.622818 -3.123610 -1.345260

H 0.382557 -3.803308 0.276934

C -1.139883 -1.329566 -1.524921

C -1.610332 -0.078287 -1.272930

H -1.699223 -2.207321 -1.231855

H -0.323920 -1.422589 -2.228994

C -1.436410 2.288159 -1.549762

C -0.549623 3.215603 -2.346116

H -2.463429 2.297727 -1.924452

H -1.453524 2.551388 -0.486505

H -0.903351 4.243408 -2.233208

H 0.484567 3.165261 -1.994490

H -0.569994 2.953379 -3.406621

O -0.893440 0.960456 -1.698822

O -2.660380 0.241918 -0.537564

Si -4.012642 -0.730700 -0.074943

C -3.436040 -2.007215 1.163255

H -4.306330 -2.475386 1.637913

H -2.853168 -2.807969 0.697524

H -2.835397 -1.554763 1.958816

C -4.716022 -1.506396 -1.619070

H -3.969255 -2.108050 -2.145723

H -5.550911 -2.166360 -1.360679

H -5.088497 -0.747162 -2.313847

C -5.161769 0.542155 0.703591

C -4.534245 1.091313 1.992167

C -5.403091 1.696779 -0.277838

C -6.500981 -0.133984 1.032714

H -4.384237 0.304327 2.739662

H -3.565762 1.568424 1.800388

H -5.192860 1.848160 2.437696

H -5.850696 1.349382 -1.216003

H -6.093568 2.426428 0.164910

H -4.472391 2.221081 -0.519686

H -7.181881 0.592213 1.494905

H -6.993913 -0.519951 0.133219

H -6.380790 -0.964167 1.738372

**(3a)TS4b**

M062x/BSI SCF energy: -2058.599144 a.u.

M062x/BSII SCF energy in solution: -2059.003937 a.u.

M062x/BSII free energy in solution: -2058.395376 a.u.

S 0.938240 -3.069741 0.497671

O -3.619159 0.033245 -1.961329

O -3.894653 -2.038027 -1.131236

C -0.320997 -1.914819 0.429870

C -0.320548 -0.787142 -0.407132

C -1.391418 0.078677 -0.372698

C -2.710953 -0.347896 0.199642

C -2.571935 -1.452032 1.197222

C -1.448928 -2.178385 1.304799

C 2.179470 -2.480268 -0.699747

C 3.419887 -3.362525 -0.593716

C 4.478490 -2.939604 -1.612151

C 5.736864 -3.795981 -1.515121

C -3.472756 -0.916468 -1.043608

H 0.544502 -0.535259 -1.008192

H -1.405962 0.934486 -1.039868

H -3.434504 -1.689504 1.813931

H -1.377899 -2.992102 2.020342

H 1.740908 -2.527966 -1.701010

H 2.420034 -1.439317 -0.462440

H 3.834181 -3.293075 0.419672

H 3.148584 -4.411555 -0.763164

H 4.733835 -1.885083 -1.450265

H 4.055855 -3.011499 -2.621459

H 6.189870 -3.716179 -0.521757

H 5.508246 -4.851089 -1.695941

C -4.417781 -0.330814 -3.101968

H -5.418648 -0.624293 -2.779073

H -4.461749 0.560238 -3.724796

H -3.946263 -1.154352 -3.640603

H 6.483899 -3.483921 -2.249849

C -7.336783 0.730419 0.026845

N -8.460401 0.452261 0.046938

C -5.947640 1.086020 -0.014762

H -5.672690 1.878773 -0.705203

C -5.046999 0.473106 0.760492

C -3.589774 0.824280 0.733297

H -5.368742 -0.320927 1.432075

H -3.422138 1.703491 0.105347

H -3.262690 1.054247 1.749615

C -0.639899 1.884632 1.393174

C 0.532533 1.205265 1.496935

H -0.750920 2.707113 0.699896

H -1.374124 1.744282 2.172770

C 1.906485 -0.358788 2.668772

C 1.702714 -1.342650 3.796167

H 2.313297 -0.845714 1.776169

H 2.583330 0.451435 2.956288

H 2.655514 -1.817282 4.043480

H 1.322634 -0.836453 4.686755

H 0.993282 -2.123067 3.506982

O 0.612011 0.194485 2.362222

O 1.602811 1.352101 0.732811

Si 2.023735 2.682752 -0.286215

C 1.763130 4.264619 0.670533

H 2.315239 5.079393 0.188620

H 0.711742 4.560627 0.713563

H 2.134772 4.173010 1.696259

C 0.952747 2.598685 -1.816303

H -0.102069 2.768988 -1.579352

H 1.255392 3.385072 -2.517051

H 1.044887 1.639486 -2.336970

C 3.844479 2.357884 -0.651288

C 4.694218 2.752575 0.565130

C 4.065990 0.870774 -0.958435

C 4.264985 3.199405 -1.866000

H 4.621036 3.823819 0.781799

H 4.395007 2.203264 1.465580

H 5.750697 2.524091 0.373978

H 3.440640 0.522493 -1.790569

H 5.113275 0.698086 -1.240324

H 3.848878 0.249769 -0.081709

H 5.339786 3.077271 -2.051262

H 3.735834 2.893234 -2.775045

H 4.080345 4.269239 -1.710959

**(3a)TS4c**

M062x/BSI SCF energy: -2058.599986 a.u.

M062x/BSII SCF energy in solution: -2059.004081 a.u.

M062x/BSII free energy in solution: -2058.393778 a.u.

S 1.598801 -2.155626 1.167589

O -4.618767 -2.598058 -0.601497

O -4.309178 -2.031491 1.550635

C 0.122695 -1.680187 0.450082

C -0.692030 -0.808814 1.279994

C -2.002700 -0.655106 1.035814

C -2.699163 -1.301741 -0.116961

C -1.827526 -2.259671 -0.869631

C -0.515844 -2.414015 -0.627758

C 2.469827 -3.100185 -0.122762

C 3.893663 -3.352913 0.364854

C 4.767376 -3.920911 -0.751686

C 6.185707 -4.209275 -0.270909

C -3.957625 -2.021663 0.397998

H -0.220476 -0.304388 2.118151

H -2.604000 -0.034183 1.694126

H -2.302892 -2.828965 -1.663416

H 0.072758 -3.101829 -1.223546

H 1.944231 -4.039624 -0.310550

H 2.462794 -2.482772 -1.026914

H 4.332273 -2.415159 0.728947

H 3.874676 -4.047332 1.213076

H 4.792434 -3.205603 -1.583132

H 4.310673 -4.839389 -1.139994

H 6.662860 -3.299789 0.109425

H 6.182056 -4.947394 0.537523

C -5.844621 -3.255106 -0.236473

H -6.526879 -2.541847 0.229620

H -6.261937 -3.634760 -1.166881

H -5.639972 -4.075060 0.454239

H 6.807443 -4.600893 -1.080398

C -6.443959 1.567185 -0.238403

N -7.216194 2.289434 0.232898

C -5.497800 0.667552 -0.832627

H -5.896336 -0.052992 -1.542159

C -4.195366 0.728164 -0.533383

C -3.193303 -0.204419 -1.139153

H -3.837423 1.467273 0.181168

H -2.315503 0.356433 -1.473478

H -3.627057 -0.723618 -1.996973

C 0.839670 0.045269 -1.262552

C 1.806165 0.538795 -0.440799

H -0.158917 0.451456 -1.229465

H 1.128097 -0.608330 -2.075340

C 4.092067 0.522417 0.309169

C 5.392438 0.132090 -0.354356

H 3.998786 1.607744 0.384892

H 3.991785 0.093383 1.311030

H 6.229409 0.460501 0.266893

H 5.463558 -0.951668 -0.484836

H 5.480555 0.606558 -1.335098

O 3.025674 0.007949 -0.517307

O 1.613912 1.400727 0.543903

Si 0.757616 2.906369 0.540375

C 0.646650 3.292686 2.357626

H 0.275321 4.310524 2.517287

H -0.043062 2.605624 2.858708

H 1.623698 3.207680 2.842269

C -0.945830 2.710249 -0.208769

H -1.455477 1.840692 0.219833

H -1.538538 3.598399 0.039094

H -0.930856 2.610955 -1.298445

C 1.812154 4.140259 -0.427528

C 2.997422 4.609413 0.426776

C 2.333258 3.506211 -1.724884

C 0.932930 5.352370 -0.775656

H 2.663607 5.133267 1.328872

H 3.636994 3.775981 0.739773

H 3.621586 5.304385 -0.149888

H 1.522688 3.115477 -2.351748

H 2.873412 4.258190 -2.314872

H 3.033047 2.684882 -1.528225

H 1.537832 6.116421 -1.280793

H 0.111658 5.081678 -1.448089

H 0.500372 5.817967 0.118327

**4a**

M062x/BSI SCF energy: -1531.735671 a.u.

M062x/BSII SCF energy in solution: -1532.064009 a.u.

M062x/BSII free energy in solution: -1531.648718 a.u.

S -2.271612 -2.099802 1.151196

O 3.298149 1.313775 -0.822928

O 3.556114 1.033811 1.386662

C -0.846964 -1.102799 0.817790

C 0.229503 -1.247468 1.816072

C 1.457135 -0.781321 1.568166

C 1.781171 -0.162109 0.228331

C 0.551467 0.596537 -0.326518

C -0.694314 -0.259515 -0.216449

C -3.421534 -1.586857 -0.160860

C -4.735205 -2.349569 -0.022775

C -5.747153 -1.919790 -1.084600

C -7.055458 -2.697060 -0.981393

C 2.966815 0.785866 0.361479

H 0.004034 -1.740164 2.758514

H 2.253457 -0.875125 2.299648

H 0.738797 0.819248 -1.381737

H -1.472256 -0.095363 -0.952699

H -3.586482 -0.507281 -0.075600

H -2.960396 -1.790391 -1.132632

H -4.546290 -3.426616 -0.111671

H -5.159546 -2.180618 0.974634

H -5.308780 -2.061946 -2.080026

H -5.944402 -0.845898 -0.979733

H -6.886421 -3.769684 -1.121419

H -7.518121 -2.558321 0.001357

C 4.432181 2.190063 -0.809712

H 5.317632 1.658684 -0.454015

H 4.570207 2.513797 -1.840007

H 4.240886 3.049806 -0.163866

H -7.773903 -2.369717 -1.738156

C 5.802284 -2.479464 -0.598453

N 6.778522 -3.002804 -0.260011

C 4.602771 -1.823881 -1.030410

H 4.682178 -1.207290 -1.921920

C 3.447091 -1.971800 -0.370969

C 2.189270 -1.277528 -0.784116

H 3.415001 -2.593351 0.522187

H 1.363131 -1.994784 -0.833702

H 2.317877 -0.822971 -1.770294

C 0.320469 1.933777 0.394587

C -0.807909 2.712078 -0.240315

H 1.207750 2.579183 0.342375

H 0.096849 1.779800 1.454685

C -2.298787 4.521116 0.048373

C -3.638740 3.835083 0.219988

H -2.242956 5.439311 0.635073

H -2.098753 4.752650 -1.000327

H -4.436936 4.496905 -0.127336

H -3.676908 2.911903 -0.362905

H -3.818792 3.599906 1.272118

O -1.229101 3.698878 0.557145

O -1.260822 2.498863 -1.343491

**9d**

M062x/BSI SCF energy: -1029.271799 a.u.

M062x/BSII SCF energy in solution: -1029.479221 a.u.

M062x/BSII free energy in solution: -1029.074689 a.u.

C 1.468034 -0.286699 -0.229787

C 2.050478 -2.506130 0.379921

H 2.899070 -2.645848 -0.295990

H 2.409010 -2.154565 1.352909

O 1.101849 -1.597439 -0.161822

O 0.383141 0.507481 -0.330513

Si -1.205328 -0.015450 0.023324

C -2.145045 1.624068 -0.021942

H -3.205822 1.394697 0.160351

C -1.235488 -0.844326 1.728246

H -1.000151 -1.900545 1.535988

C -1.804204 -1.221501 -1.313032

C -3.322809 -1.149034 -1.525119

C -1.056279 -1.049299 -2.641054

H -3.882696 -1.301577 -0.595873

H -3.624817 -0.179209 -1.935874

H -3.651549 -1.917568 -2.235038

H 0.020425 -1.196275 -2.516240

H -1.411481 -1.777467 -3.380722

H -1.208913 -0.051353 -3.066745

H -1.565152 -2.222477 -0.927407

C -0.166604 -0.298555 2.683280

H -0.183150 -0.845018 3.634330

H 0.842833 -0.388074 2.267615

H -0.333313 0.759349 2.911954

C -2.629389 -0.780548 2.368334

H -2.650313 -1.336777 3.313170

H -2.920267 0.251945 2.593162

H -3.402059 -1.208973 1.720378

C -1.664644 2.585774 1.073000

H -1.839816 2.192222 2.078718

H -0.591367 2.786251 0.974206

H -2.186388 3.548026 1.000256

C -2.023576 2.298989 -1.396037

H -2.430581 1.682456 -2.203212

H -2.562201 3.254414 -1.407769

H -0.974834 2.509223 -1.634731

C 2.699437 0.228406 -0.214392

C 4.134210 -0.275390 -0.215867

C 3.115554 1.683304 -0.263378

C 4.595787 1.209900 -0.212787

H 4.427510 -0.829143 -1.113917

H 4.447385 -0.854895 0.658210

H 2.838257 2.212130 -1.181819

H 2.793858 2.293071 0.588201

H 5.197335 1.499825 -1.076359

H 5.123255 1.503904 0.696735

H 1.527837 -3.455551 0.501349

**(9d)IM5**

M062x/BSI SCF energy: -2253.839891 a.u.

M062x/BSII SCF energy in solution: -2254.284062 a.u.

M062x/BSII free energy in solution: -2253.554315 a.u.

S -0.203946 1.891527 0.489105

O -4.414427 -2.402574 -1.331835

O -5.159192 -2.468947 0.788492

C -1.490676 0.842220 0.206857

C -2.062098 0.219528 1.377100

C -3.246301 -0.415288 1.289309

C -4.034701 -0.478370 0.024693

C -3.271937 -0.023574 -1.170817

C -2.085983 0.614592 -1.083351

C 0.305007 2.524743 -1.142264

C 1.318794 3.643496 -0.919444

C 1.847114 4.170660 -2.252952

C 2.910733 5.245176 -2.054082

C -4.601424 -1.903831 -0.117811

H -1.535088 0.291248 2.324158

H -3.697163 -0.871240 2.167075

H -3.744970 -0.151502 -2.140284

H -1.598431 0.985288 -1.976532

H 0.744206 1.687741 -1.694475

H -0.576894 2.900848 -1.667076

H 0.849255 4.460187 -0.358673

H 2.155728 3.272126 -0.316882

H 1.012017 4.573068 -2.838981

H 2.266463 3.337276 -2.829860

H 2.511862 6.092913 -1.487870

H 3.768858 4.847126 -1.502216

C -4.869892 -3.754015 -1.527622

H -5.942848 -3.820449 -1.340827

H -4.644004 -3.989531 -2.565554

H -4.332245 -4.425234 -0.855112

H 3.274939 5.623075 -3.013110

C -4.512173 3.947297 1.443216

N -4.205182 5.062990 1.478943

C -4.884202 2.562442 1.409198

H -5.089759 2.091089 2.366910

C -4.965724 1.896257 0.252879

C -5.319966 0.444548 0.184974

H -4.747826 2.409478 -0.681101

H -5.961600 0.250367 -0.680165

H -5.836627 0.116535 1.090403

C 0.906566 -1.237689 0.281641

C 0.219970 -2.415060 2.222918

H -0.862228 -2.375086 2.069849

H 0.617595 -3.345210 1.803647

O 0.871707 -1.289866 1.639044

O 1.923524 -0.465446 -0.148234

Si 3.525366 -0.720636 0.441831

C 4.584105 0.029848 -0.929317

H 5.611230 0.084040 -0.542066

C 3.754663 -2.576857 0.726523

H 3.103724 -2.799011 1.586321

C 3.806239 0.183289 2.088134

C 5.240441 0.721829 2.208448

C 2.791238 1.298651 2.356635

H 6.000142 -0.047558 2.033876

H 5.420737 1.531202 1.491362

H 5.411582 1.132106 3.210614

H 1.772636 0.905557 2.391247

H 3.003238 1.791671 3.313535

H 2.827353 2.071356 1.578432

H 3.667841 -0.583988 2.864380

C 3.335223 -3.497748 -0.426240

H 3.346791 -4.544167 -0.097878

H 2.327732 -3.275738 -0.791332

H 4.024116 -3.419874 -1.272081

C 5.201404 -2.868949 1.151951

H 5.337433 -3.937398 1.357174

H 5.910889 -2.598716 0.359868

H 5.485008 -2.322693 2.057723

C 4.599274 -0.800223 -2.217762

H 5.112945 -1.755969 -2.082893

H 3.582668 -1.010380 -2.571468

H 5.118095 -0.258740 -3.018169

C 4.109601 1.459965 -1.225922

H 4.128446 2.096663 -0.334687

H 4.743507 1.931470 -1.986828

H 3.082351 1.448629 -1.609571

C 0.064863 -1.820571 -0.583679

C -1.157730 -2.714482 -0.526634

C 0.103732 -1.822482 -2.096699

C -1.156574 -2.734030 -2.078854

H -2.044752 -2.250685 -0.078541

H -1.016387 -3.688842 -0.049437

H -0.040104 -0.846119 -2.573170

H 0.997774 -2.284321 -2.531388

H -2.046959 -2.285823 -2.524778

H -1.002003 -3.722979 -2.513865

H 0.436604 -2.369833 3.290482

**(9d)TS4A**

M062x/BSI SCF energy: -2253.830784 a.u.

M062x/BSII SCF energy in solution: -2254.272491 a.u.

M062x/BSII free energy in solution: -2253.543436 a.u.

S 3.739923 -1.330894 0.471689

O 0.158946 3.502984 -2.124438

O -1.391448 2.909723 -0.612360

C 2.636471 -0.073563 0.033845

C 3.007790 1.026156 -0.841538

C 2.160901 2.054186 -1.030824

C 0.859647 2.131181 -0.304810

C 0.396554 0.806296 0.213613

C 1.349218 -0.148362 0.560156

C 5.283414 -0.963141 -0.431818

C 6.297151 -2.060274 -0.118784

C 7.621103 -1.797578 -0.836851

C 8.651439 -2.885122 -0.551170

C -0.262984 2.867774 -1.038063

H 3.972096 1.023257 -1.334596

H 2.447137 2.896591 -1.652203

H -0.563316 0.835022 0.723345

H 1.063095 -0.988827 1.185918

H 5.657296 0.012556 -0.109958

H 5.065333 -0.935950 -1.502994

H 5.897456 -3.033129 -0.429278

H 6.470343 -2.110137 0.962866

H 7.439254 -1.731527 -1.916397

H 8.012926 -0.822879 -0.522366

H 8.289416 -3.864436 -0.880286

H 8.865529 -2.950036 0.520448

C -0.820683 4.326502 -2.782176

H -1.628608 3.709796 -3.181226

H -0.289037 4.824799 -3.590028

H -1.227670 5.054485 -2.078459

H 9.592948 -2.684547 -1.069633

C 2.794636 1.861011 4.152336

N 3.598689 1.499633 4.903058

C 1.787849 2.301618 3.231401

H 0.779453 2.396760 3.624807

C 2.087605 2.571151 1.956324

C 1.063091 3.057146 0.984840

H 3.113472 2.464458 1.609812

H 0.086183 3.149735 1.465978

H 1.359980 4.042506 0.604864

C -1.368426 -0.888088 -0.711628

C -0.200080 -2.958828 -0.446616

H 0.816279 -2.587472 -0.592869

H -0.603507 -3.352792 -1.382118

O -1.062120 -1.929532 0.064008

O -2.480593 -0.291049 -0.347210

Si -3.763545 -1.049485 0.552871

C -5.091906 0.273161 0.362968

H -5.962392 -0.048489 0.952507

C -4.136884 -2.695230 -0.299598

H -3.444426 -3.423021 0.146725

C -3.245103 -1.317270 2.354434

C -4.458004 -1.198849 3.290913

C -2.105085 -0.401730 2.819392

H -5.282292 -1.856500 2.994349

H -4.844392 -0.174197 3.315833

H -4.176973 -1.467138 4.315632

H -1.173566 -0.624510 2.289644

H -1.914361 -0.545893 3.889639

H -2.339970 0.658502 2.669139

H -2.880623 -2.352857 2.406251

C -3.891385 -2.685063 -1.813545

H -4.112019 -3.670160 -2.240893

H -2.851388 -2.448281 -2.068071

H -4.528460 -1.956304 -2.324417

C -5.569106 -3.149483 0.021649

H -5.763410 -4.140968 -0.402966

H -6.309525 -2.460997 -0.400680

H -5.750341 -3.212795 1.100106

C -5.529025 0.444577 -1.098087

H -6.001925 -0.456815 -1.499381

H -4.673560 0.691778 -1.737959

H -6.252115 1.263997 -1.186916

C -4.591096 1.614328 0.921975

H -4.361969 1.559454 1.990799

H -5.352795 2.391669 0.788794

H -3.685522 1.942618 0.398941

C -0.611316 -0.363791 -1.734482

C 0.567632 -0.844663 -2.558581

C -1.095802 0.523215 -2.864235

C 0.289482 0.330310 -3.529768

H 1.546802 -0.873809 -2.068308

H 0.390037 -1.821925 -3.019537

H -1.447433 1.531098 -2.645028

H -1.889275 0.010005 -3.421996

H 0.949692 1.180516 -3.334037

H 0.296290 0.100729 -4.595917

H -0.200986 -3.736743 0.315878

**(9d)TS4B**

M062x/BSI SCF energy: -2253.834649 a.u.

M062x/BSII SCF energy in solution: -2254.276922 a.u.

M062x/BSII free energy in solution: -2253.546779 a.u.

S -4.137027 0.339234 1.090304

O 1.468012 -2.920968 -0.582639

O 0.475887 -4.378556 0.810480

C -2.756862 -0.632152 0.755405

C -2.829701 -2.001118 1.241509

C -1.884997 -2.889803 0.893986

C -0.805916 -2.525838 -0.065714

C -0.565573 -1.054326 -0.153869

C -1.625062 -0.181665 0.077770

C -3.758293 1.935281 0.296684

C -4.945035 2.876503 0.477910

C -4.671062 4.228357 -0.181452

C -5.839253 5.193684 -0.012221

C 0.448155 -3.376019 0.140534

H -3.658493 -2.287757 1.881611

H -1.926304 -3.920546 1.231826

H 0.257693 -0.749399 -0.795368

H -1.529687 0.853612 -0.226663

H -2.856348 2.350123 0.758555

H -3.567096 1.747532 -0.764068

H -5.843663 2.428823 0.037397

H -5.144668 3.022217 1.546393

H -4.468337 4.073553 -1.247993

H -3.762224 4.663155 0.252388

H -6.752146 4.784799 -0.456925

H -6.040486 5.385139 1.046639

C 2.646681 -3.745507 -0.581284

H 2.402347 -4.739135 -0.962314

H 3.357271 -3.247340 -1.238006

H 3.054071 -3.833006 0.428030

H -5.633066 6.153583 -0.493386

C -4.833654 -1.982051 -2.472704

N -5.741904 -1.383733 -2.870088

C -3.715836 -2.724868 -1.967931

H -3.937026 -3.681574 -1.502180

C -2.469974 -2.247078 -2.064263

C -1.284342 -2.985741 -1.537422

H -2.299087 -1.283059 -2.537036

H -0.427206 -2.837835 -2.199056

H -1.495188 -4.056544 -1.462710

C 1.255997 0.444156 1.023328

C -0.037069 2.250597 1.898324

H -0.962022 1.702395 2.089197

H 0.569844 2.292932 2.806159

O 0.713230 1.651903 0.831418

O 2.242835 0.191383 0.185641

Si 3.238798 1.384691 -0.589296

C 4.462789 0.241181 -1.449416

H 5.234865 0.871510 -1.913848

C 4.007090 2.450707 0.774501

H 3.365675 3.339195 0.862559

C 2.220559 2.441960 -1.781610

C 3.138615 3.077757 -2.837291

C 1.039671 1.720294 -2.440917

H 3.953632 3.652799 -2.384432

H 3.588266 2.320054 -3.488707

H 2.570766 3.762938 -3.477148

H 0.319683 1.368016 -1.695529

H 0.507830 2.402154 -3.115436

H 1.360275 0.860043 -3.037472

H 1.806978 3.251400 -1.164228

C 4.039687 1.754710 2.141703

H 4.517332 2.401091 2.887268

H 3.036434 1.518582 2.514375

H 4.607631 0.818703 2.106382

C 5.416067 2.921894 0.379665

H 5.811532 3.617487 1.128545

H 6.112624 2.078358 0.317962

H 5.434031 3.437333 -0.586412

C 5.140155 -0.700193 -0.442827

H 5.733993 -0.160054 0.301263

H 4.394064 -1.297525 0.094394

H 5.812183 -1.395549 -0.959995

C 3.772304 -0.574872 -2.552451

H 3.420767 0.054943 -3.374962

H 4.465773 -1.311419 -2.976470

H 2.907931 -1.122891 -2.155645

C 0.838268 -0.516339 1.906942

C -0.143034 -0.572580 3.061833

C 1.628037 -1.687841 2.453946

C 0.443909 -1.963162 3.413135

H -1.208844 -0.530353 2.811719

H 0.056653 0.179039 3.831960

H 1.948458 -2.487428 1.787267

H 2.515048 -1.325797 2.988690

H -0.189183 -2.777038 3.047790

H 0.691914 -2.140645 4.460207

H -0.265545 3.260437 1.559289

**(9d)TS4C**

M062x/BSI SCF energy: -2253.835968 a.u.

M062x/BSII SCF energy in solution: -2254.279473 a.u.

M062x/BSII free energy in solution: -2253.549551 a.u.

S -0.038939 1.717530 0.225008

O -5.535767 -1.740543 -1.109417

O -4.450338 -2.787676 0.554110

C -1.217939 0.502531 -0.041562

C -1.739181 -0.094723 1.182251

C -2.990510 -0.571688 1.238704

C -3.961957 -0.451553 0.105026

C -3.307619 -0.005372 -1.171108

C -2.048100 0.458560 -1.233094

C 0.373806 2.421722 -1.404789

C 1.184125 3.696051 -1.169227

C 1.769857 4.222495 -2.478553

C 2.608312 5.477854 -2.263121

C -4.663320 -1.801489 -0.108427

H -1.090786 -0.128538 2.054226

H -3.367639 -1.005805 2.160666

H -3.928496 0.000303 -2.063222

H -1.654081 0.830639 -2.171826

H 0.953911 1.675500 -1.956120

H -0.544266 2.651533 -1.950712

H 0.541088 4.457169 -0.712452

H 2.000238 3.500536 -0.462474

H 0.955200 4.431366 -3.182537

H 2.386767 3.437690 -2.934387

H 2.011482 6.276464 -1.811016

H 3.452255 5.271905 -1.596086

C -6.237320 -2.962431 -1.396586

H -6.833838 -3.263952 -0.534115

H -6.879078 -2.739362 -2.246455

H -5.526016 -3.751481 -1.647274

H 3.010726 5.852858 -3.207928

C -4.067434 3.864467 2.144249

N -3.618267 4.917584 2.317124

C -4.621099 2.557231 1.940654

H -5.115289 2.100287 2.793434

C -4.524506 1.943430 0.757090

C -5.083086 0.581234 0.498652

H -4.014273 2.442000 -0.065326

H -5.796511 0.623309 -0.331056

H -5.603430 0.203826 1.384605

C 0.920797 -1.327699 0.139797

C 0.123385 -2.866449 1.786797

H -0.937771 -2.704348 1.589845

H 0.483469 -3.747660 1.249978

O 0.900839 -1.717390 1.416532

O 1.949873 -0.552686 -0.134421

Si 3.526493 -0.802692 0.578458

C 4.607673 0.188117 -0.604596

H 5.619498 0.195463 -0.174356

C 3.842973 -2.668733 0.566826

H 3.336451 -3.057899 1.461985

C 3.589759 -0.133154 2.347661

C 4.992825 0.394231 2.688088

C 2.530907 0.929846 2.658418

H 5.780323 -0.345077 2.508461

H 5.234448 1.285014 2.097428

H 5.044715 0.678960 3.745125

H 1.518778 0.545384 2.508257

H 2.618184 1.258913 3.700853

H 2.647668 1.816473 2.025019

H 3.387320 -1.000982 2.991819

C 3.280557 -3.414831 -0.649973

H 3.481456 -4.488804 -0.559081

H 2.195580 -3.295634 -0.752961

H 3.740414 -3.076850 -1.584010

C 5.346640 -2.944950 0.725546

H 5.536395 -4.023853 0.759570

H 5.919238 -2.538556 -0.115980

H 5.752075 -2.512905 1.646506

C 4.680249 -0.428339 -2.006917

H 5.151173 -1.415568 -2.001898

H 3.680127 -0.533504 -2.444561

H 5.265218 0.213344 -2.676424

C 4.103035 1.635204 -0.693217

H 4.094405 2.138524 0.279446

H 4.731920 2.228565 -1.368049

H 3.081485 1.652271 -1.090106

C -0.014363 -1.571422 -0.832684

C -1.126961 -2.577924 -1.028536

C 0.200945 -1.392085 -2.322225

C -0.986475 -2.364770 -2.559114

H -2.096223 -2.292863 -0.598314

H -0.901426 -3.591599 -0.688326

H 0.073900 -0.382388 -2.720636

H 1.172016 -1.772775 -2.658353

H -1.858040 -1.892974 -3.016382

H -0.730200 -3.264234 -3.119948

H 0.286712 -2.992360 2.856126

**(9d)TS4a**

M062x/BSI SCF energy: -2253.824644 a.u.

M062x/BSII SCF energy in solution: -2254.267275 a.u.

M062x/BSII free energy in solution: -2253.536423 a.u.

S 3.782215 -1.218640 -0.866286

O 1.636906 4.268355 -0.391870

O 0.822890 2.821395 -1.904853

C 2.710281 -0.041992 -0.193600

C 1.373472 -0.099666 -0.572994

C 0.470371 0.849771 -0.101484

C 0.977628 2.150157 0.454581

C 2.347625 2.007194 1.054368

C 3.160829 0.987978 0.731562

C 5.412358 -0.892209 -0.110594

C 6.410150 -1.889496 -0.694525

C 7.791720 -1.717568 -0.063554

C 8.807342 -2.698319 -0.641111

C 1.136924 3.097285 -0.776517

H 1.014415 -0.915435 -1.193540

H -0.504925 0.886408 -0.576780

H 2.690842 2.798393 1.715248

H 4.159017 0.938419 1.148668

H 5.713378 0.134010 -0.337051

H 5.328947 -1.018402 0.972507

H 6.055950 -2.913311 -0.523396

H 6.481144 -1.748510 -1.779666

H 7.711942 -1.859776 1.020924

H 8.136211 -0.688672 -0.222676

H 8.491389 -3.733153 -0.474079

H 8.921349 -2.553445 -1.720159

C 1.765149 5.254793 -1.431778

H 0.795403 5.437556 -1.898252

H 2.129911 6.153619 -0.938629

H 2.478142 4.913551 -2.184282

H 9.789944 -2.568708 -0.179409

C -2.803791 4.814437 -0.300613

N -3.794950 5.067860 -0.842444

C -1.573133 4.507805 0.369767

H -0.898116 5.339346 0.556901

C -1.285511 3.255171 0.741250

C 0.001231 2.887468 1.416144

H -1.999667 2.461561 0.527623

H -0.194007 2.241593 2.274293

H 0.504708 3.788090 1.775331

C -1.468974 -0.477774 1.111489

C -2.924509 0.757382 2.575571

H -2.911969 -0.044680 3.317963

H -2.247620 1.563022 2.870321

O -2.585153 0.237411 1.283914

O -1.545440 -1.256469 0.047687

Si -3.052272 -1.845323 -0.603222

C -2.427036 -3.138449 -1.821890

H -3.319244 -3.584412 -2.286654

C -3.988712 -0.433925 -1.444960

H -4.623326 0.000918 -0.660487

C -4.034324 -2.554377 0.850794

C -5.021961 -3.627964 0.368590

C -3.161604 -3.103092 1.987160

H -5.689208 -3.260098 -0.417913

H -4.492723 -4.502994 -0.024943

H -5.650715 -3.971521 1.197904

H -2.493593 -2.344747 2.412815

H -3.791766 -3.473111 2.804452

H -2.537328 -3.939022 1.653856

H -4.616675 -1.711928 1.251749

C -3.079417 0.680857 -1.975473

H -3.673776 1.461650 -2.464506

H -2.519591 1.159246 -1.166112

H -2.355953 0.310701 -2.709818

C -4.908464 -0.962884 -2.556262

H -5.512331 -0.147266 -2.970083

H -4.332279 -1.393534 -3.382483

H -5.600707 -1.731424 -2.196465

C -1.576325 -2.501556 -2.930837

H -2.142220 -1.783849 -3.531812

H -0.708740 -1.976408 -2.512892

H -1.194422 -3.272594 -3.610213

C -1.636443 -4.255033 -1.127603

H -2.245369 -4.812289 -0.409486

H -1.258537 -4.971622 -1.866270

H -0.769496 -3.848417 -0.593353

C -0.299304 -0.439453 1.839718

C 0.054534 -0.011582 3.264150

C 0.622603 -1.633942 1.937276

C 0.579015 -1.452509 3.474491

H -0.727866 0.349926 3.930730

H 0.882540 0.702782 3.264675

H 0.259075 -2.571259 1.508390

H 1.621825 -1.420942 1.540002

H -0.184238 -2.086497 3.930453

H 1.522053 -1.561799 4.012826

H -3.934744 1.150216 2.468854

**(9d)TS4b**

M062x/BSI SCF energy: -2253.82931 a.u.

M062x/BSII SCF energy in solution: -2254.271854 a.u.

M062x/BSII free energy in solution: -2253.540383 a.u.

S 4.071643 -2.291115 0.427117

O -1.593848 -2.160108 -1.897743

O -0.400147 -4.051687 -1.669998

C 2.356549 -2.191906 0.409723

C 1.675551 -3.335423 1.003130

C 0.345982 -3.461991 0.896400

C -0.493845 -2.448711 0.185469

C 0.234760 -1.159182 -0.076887

C 1.626066 -1.137250 -0.129833

C 4.624058 -0.748401 -0.367425

C 6.147844 -0.756547 -0.450505

C 6.668654 0.531637 -1.087550

C 8.189157 0.534504 -1.207878

C -0.799514 -3.001331 -1.242901

H 2.268178 -4.095647 1.502551

H -0.151645 -4.340335 1.298594

H -0.308677 -0.421934 -0.658941

H 2.119726 -0.265046 -0.540818

H 4.179252 -0.697658 -1.365539

H 4.266408 0.095977 0.230832

H 6.575205 -0.867918 0.553161

H 6.478850 -1.618897 -1.041309

H 6.341623 1.388791 -0.486823

H 6.217495 0.651538 -2.079823

H 8.660837 0.433959 -0.225034

H 8.533877 -0.297191 -1.830650

C -2.040837 -2.605415 -3.189807

H -2.540180 -3.572121 -3.099279

H -2.736783 -1.844636 -3.538127

H -1.192198 -2.689516 -3.871155

H 8.548739 1.463223 -1.658999

C -4.739838 -4.516919 -0.202795

N -5.436830 -5.431644 -0.336033

C -3.884791 -3.375266 -0.050845

H -4.183436 -2.473662 -0.580204

C -2.783440 -3.423991 0.706492

C -1.876689 -2.242518 0.872269

H -2.520535 -4.350148 1.214053

H -1.696231 -2.069406 1.936712

H -2.343079 -1.351912 0.443537

C -0.890873 0.981299 1.183678

C -2.927828 0.739612 2.435202

H -2.975148 -0.330263 2.650297

H -2.449350 1.276140 3.258062

O -2.229210 0.988683 1.209543

O -0.413364 1.762348 0.227433

Si -1.308470 3.088651 -0.460092

C 0.042032 3.931427 -1.467712

H -0.442464 4.760977 -2.003627

C -1.974953 4.118407 0.979010

H -2.931806 3.652684 1.255475

C -2.713985 2.436014 -1.547674

C -2.995941 3.392148 -2.716725

C -2.487351 1.006421 -2.056216

H -3.191409 4.416933 -2.383138

H -2.152741 3.426426 -3.415055

H -3.874458 3.058511 -3.280860

H -2.426737 0.287318 -1.234108

H -3.319199 0.699071 -2.702028

H -1.567801 0.919808 -2.646869

H -3.602118 2.421604 -0.900452

C -1.072846 4.106708 2.219862

H -1.529221 4.689270 3.028687

H -0.904194 3.094109 2.605786

H -0.091634 4.546610 2.013417

C -2.266621 5.556936 0.525878

H -2.736813 6.128333 1.334348

H -1.346282 6.081940 0.246560

H -2.943424 5.589979 -0.334862

C 1.157400 4.508324 -0.586141

H 0.795090 5.288957 0.089320

H 1.620052 3.723608 0.024069

H 1.946860 4.948804 -1.206608

C 0.640782 2.971477 -2.506837

H -0.103519 2.600445 -3.217965

H 1.426843 3.473665 -3.082885

H 1.098069 2.103041 -2.018164

C -0.022931 0.241214 1.947080

C -0.138675 -0.510637 3.267305

C 1.374717 0.705401 2.296818

C 1.038955 0.359992 3.770070

H 0.179105 -1.551093 3.143984

H -1.074075 -0.486996 3.825753

H 2.149994 0.034809 1.904553

H 1.626569 1.738549 2.044738

H 1.814340 -0.141956 4.351550

H 0.676473 1.236032 4.312280

H -3.935077 1.123379 2.276815

**(9d)TS4c**

M062x/BSI SCF energy: -2253.83228 a.u.

M062x/BSII SCF energy in solution: -2254.276613 a.u.

M062x/BSII free energy in solution: -2253.546463 a.u.

S 0.690623 2.376278 -1.050776

O -5.188589 1.334744 -0.073485

O -4.196966 0.566546 -1.937666

C -0.440678 1.424569 -0.166763

C -0.803103 0.162405 -0.790475

C -2.017615 -0.378589 -0.605560

C -3.091413 0.267104 0.216222

C -2.588928 1.465587 0.966339

C -1.374377 2.010827 0.776042

C 0.726077 3.998188 -0.211265

C 1.801571 4.855753 -0.872039

C 1.843989 6.248950 -0.244660

C 2.920017 7.126542 -0.875357

C -4.205925 0.741435 -0.746371

H -0.081241 -0.319430 -1.443648

H -2.275400 -1.293829 -1.131628

H -3.288247 1.939401 1.650235

H -1.105439 2.919025 1.303105

H -0.259059 4.462315 -0.310094

H 0.952307 3.849319 0.848635

H 2.780097 4.371280 -0.763334

H 1.601038 4.944344 -1.946709

H 2.025188 6.153635 0.832870

H 0.862346 6.724000 -0.358815

H 3.912465 6.682224 -0.749037

H 2.743429 7.251606 -1.948372

C -6.318883 1.742187 -0.863289

H -6.748445 0.878870 -1.375227

H -7.032626 2.164312 -0.158574

H -6.013997 2.491085 -1.596208

H 2.937029 8.120507 -0.420308

C -6.608584 -2.875322 -0.175908

N -7.202140 -3.701519 -0.728474

C -5.886472 -1.846196 0.514808

H -6.483121 -1.114268 1.053193

C -4.549481 -1.803266 0.499987

C -3.777240 -0.733487 1.208730

H -3.994353 -2.559706 -0.051502

H -3.001242 -1.187449 1.831416

H -4.444602 -0.156760 1.854099

C 1.800583 -0.064727 0.805528

C 3.429743 1.667830 1.017861

H 2.675342 2.434909 1.203669

H 3.868091 1.330595 1.960348

O 2.873776 0.549587 0.315322

O 1.573398 -1.228414 0.225451

Si 2.802347 -2.274959 -0.420932

C 1.808219 -3.857274 -0.648049

H 2.481222 -4.589692 -1.117286

C 4.183674 -2.378904 0.866923

H 4.837345 -1.519086 0.662595

C 3.454325 -1.585472 -2.059498

C 3.917830 -2.714024 -2.993495

C 2.457222 -0.666294 -2.773739

H 4.660304 -3.369157 -2.525957

H 3.075941 -3.340112 -3.309848

H 4.373003 -2.297755 -3.899494

H 2.197232 0.196997 -2.154491

H 2.885925 -0.286360 -3.708753

H 1.530921 -1.191797 -3.032800

H 4.333071 -0.981105 -1.794056

C 3.705741 -2.270775 2.320574

H 4.560214 -2.319414 3.005748

H 3.184030 -1.327891 2.521434

H 3.027617 -3.087788 2.587564

C 5.008997 -3.657950 0.657946

H 5.862677 -3.682698 1.344814

H 4.409539 -4.554743 0.850812

H 5.405726 -3.734922 -0.359831

C 1.317230 -4.434469 0.685992

H 2.141512 -4.723261 1.344626

H 0.694755 -3.708059 1.221718

H 0.704425 -5.327113 0.513401

C 0.614806 -3.623761 -1.586738

H 0.922232 -3.305331 -2.587348

H 0.030551 -4.544719 -1.699330

H -0.056349 -2.857115 -1.179308

C 0.914883 0.403071 1.742298

C 0.939166 1.453212 2.846386

C -0.100755 -0.446695 2.456333

C -0.129657 0.609057 3.592356

H 0.616141 2.459440 2.565542

H 1.893194 1.534294 3.373490

H -1.046341 -0.574633 1.914115

H 0.263023 -1.439740 2.740034

H -1.092075 1.110806 3.710065

H 0.199756 0.235296 4.562494

H 4.208274 2.062511 0.367032

**14d**

M062x/BSI SCF energy: -1609.085581 a.u.

M062x/BSII SCF energy in solution: -1609.430609 a.u.

M062x/BSII free energy in solution: -1608.981136 a.u.

S 2.648203 0.627778 -0.274719

N -5.237334 3.630084 -2.414516

C 4.655168 4.706744 0.805172

H 5.475383 4.719959 1.528422

H 4.025674 5.582349 0.996399

H 5.087952 4.820334 -0.194345

C 3.844138 3.418588 0.906080

H 3.435370 3.313237 1.919167

H 4.503390 2.557279 0.747053

C 2.694373 3.382716 -0.099697

H 3.093087 3.411923 -1.121494

H 2.084268 4.286867 0.023061

C 1.757464 2.186480 0.056081

H 0.924395 2.261456 -0.649221

H 1.349729 2.152007 1.071519

C 1.254895 -0.605522 -0.090741

C 0.210156 -0.292817 -1.122827

H 0.521004 -0.389201 -2.160690

C -1.034506 0.080338 -0.834190

H -1.739022 0.277428 -1.638823

C -1.559121 0.239286 0.567455

C -0.515794 -0.105158 1.605274

H -0.833443 -0.031320 2.642943

C 0.722938 -0.492295 1.308997

H 1.419212 -0.730118 2.109770

C -2.718229 -0.744207 0.767809

C -4.449460 -1.403566 2.203950

H -4.877252 -1.055660 3.142545

H -4.057313 -2.416378 2.315433

H -5.201885 -1.387691 1.412654

C -2.053576 1.695161 0.817484

H -2.341226 1.779809 1.871155

H -1.213637 2.371450 0.633688

C -3.222470 2.071697 -0.035286

H -4.158306 1.537245 0.127952

C -3.168548 3.020164 -0.977328

H -2.261157 3.581805 -1.182022

C -4.316729 3.352479 -1.769041

C 1.846248 -2.031358 -0.329955

C 0.695894 -3.010186 -0.166346

C -1.071516 -4.117209 -1.230482

H -1.469175 -4.186982 -2.242100

H -1.814879 -3.685105 -0.558906

H -0.776535 -5.106279 -0.872292

C 3.085744 -2.399268 0.529971

H 3.335244 -1.701267 1.331800

H 2.993978 -3.401887 0.952172

C 2.701349 -2.213967 -1.614377

H 2.449891 -3.137406 -2.138943

H 2.675593 -1.386837 -2.327596

C 3.969607 -2.334519 -0.737339

H 4.598855 -1.442510 -0.770337

H 4.592546 -3.210797 -0.926917

O -2.993263 -1.652114 0.019797

O -3.385436 -0.491923 1.898101

O 0.367728 -3.502582 0.889307

O 0.073496 -3.260910 -1.320818

**11a**

M062x/BSI SCF energy: -287.495631 a.u.

M062x/BSII SCF energy in solution: -287.56874 a.u.

M062x/BSII free energy in solution: -287.466417 a.u.

C -0.938201 0.000223 -0.011197

C -0.221598 1.206777 -0.005978

C 1.168818 1.200443 0.003724

C 1.877659 -0.000287 0.007976

C 1.168503 -1.200713 0.003730

C -0.222015 -1.206446 -0.005896

H -0.767489 2.146662 -0.010218

H 1.702788 2.146480 0.010134

H 2.962481 -0.000165 0.016617

H 1.701828 -2.147093 0.009965

H -0.768385 -2.146070 -0.009898

N -2.328434 0.000121 -0.078086

H -2.765422 -0.836669 0.287164

H -2.765757 0.836023 0.288691

**(11a)IM5**

M062x/BSI SCF energy: -1512.063726 a.u.

M062x/BSII SCF energy in solution: -1512.370578 a.u.

M062x/BSII free energy in solution: -1511.947343 a.u.

S -2.601111 -0.224651 -1.023628

O 3.346999 0.571783 1.648812

O 3.867527 1.000829 -0.496502

C -0.999650 0.051528 -0.520757

C -0.562542 -0.006391 0.844982

C 0.672142 0.434347 1.174220

C 1.557940 1.106697 0.187321

C 1.156486 0.876003 -1.222934

C -0.078693 0.447719 -1.551352

C -3.492171 -0.785741 0.465485

C -4.931218 -1.106945 0.070594

C -5.734096 -1.584325 1.280554

C -7.173942 -1.923235 0.908402

C 3.058914 0.849567 0.383645

H -1.217803 -0.406951 1.608705

H 1.001619 0.410018 2.208761

H 1.871337 1.159224 -1.990437

H -0.384297 0.370178 -2.590279

H -3.457881 0.009683 1.215150

H -2.982805 -1.674742 0.849949

H -4.939041 -1.883209 -0.704154

H -5.405974 -0.215529 -0.356509

H -5.244303 -2.464756 1.714086

H -5.722918 -0.804344 2.051290

H -7.207457 -2.715666 0.153957

H -7.688025 -1.048347 0.497831

C 4.745895 0.460966 1.958330

H 5.219774 -0.271428 1.303921

H 4.791567 0.137661 2.996550

H 5.229944 1.432256 1.837329

H -7.737298 -2.264931 1.780763

C -1.931852 4.129645 -0.546711

N -3.041185 4.459968 -0.521810

C -0.561856 3.707831 -0.594928

H -0.048540 3.838682 -1.544009

C 0.036336 3.176203 0.476632

C 1.449056 2.690860 0.452147

H -0.520421 3.065205 1.403836

H 2.027507 3.178676 -0.336414

H 1.930128 2.877273 1.416648

C 1.317697 -2.425703 -0.593795

C 2.130752 -2.195424 -1.719520

C 3.508878 -2.107438 -1.580589

C 4.106452 -2.228313 -0.324948

C 3.302754 -2.419874 0.796745

C 1.918696 -2.506565 0.673974

H 1.667124 -2.116188 -2.699463

H 4.122658 -1.943595 -2.461154

H 5.185414 -2.169480 -0.224954

H 3.754243 -2.506229 1.781235

H 1.296737 -2.684549 1.547451

N -0.058358 -2.509404 -0.729955

H -0.533340 -3.017471 0.007053

H -0.385456 -2.780333 -1.650197

**(11a)TS4A**

M062x/BSI SCF energy: -1512.059794 a.u.

M062x/BSII SCF energy in solution: -1512.365083 a.u.

M062x/BSII free energy in solution: -1511.939517 a.u.

S -2.049010 0.791572 -0.674421

O 3.906672 -0.776929 1.920536

O 4.050115 -1.570314 -0.173459

C -0.420595 0.422645 -0.255369

C 0.499241 0.302290 -1.293867

C 1.780130 -0.162202 -1.002561

C 2.365369 0.091466 0.351104

C 1.315132 0.093405 1.417368

C 0.012502 0.244862 1.124957

C -2.989115 0.703182 0.886696

C -4.467150 0.908600 0.566786

C -5.314807 0.840465 1.837027

C -6.797462 1.053163 1.549195

C 3.522363 -0.865934 0.653685

H 0.173606 0.391985 -2.324441

H 2.495588 -0.288199 -1.810349

H 1.644566 0.035999 2.450495

H -0.715716 0.272913 1.926041

H -2.826013 -0.283572 1.330085

H -2.628531 1.477469 1.569402

H -4.610964 1.881140 0.080621

H -4.801664 0.139833 -0.140568

H -4.962225 1.598234 2.546886

H -5.165656 -0.134217 2.317543

H -6.971266 2.034476 1.096312

H -7.176859 0.294005 0.857694

C 5.040435 -1.584310 2.286238

H 5.911948 -1.292028 1.698106

H 5.209270 -1.391102 3.343493

H 4.817918 -2.639294 2.117828

H -7.389651 0.997564 2.466597

C 1.560394 4.658898 -1.200846

N 0.855767 5.551974 -1.416825

C 2.441042 3.556362 -0.946960

H 3.339316 3.505259 -1.555615

C 2.157492 2.650362 -0.005311

C 3.070712 1.513833 0.321683

H 1.246449 2.753090 0.581545

H 3.489722 1.665680 1.322888

H 3.899728 1.468111 -0.391355

C 0.029427 -2.567408 -0.957587

C -0.609047 -2.465693 -2.197664

C -1.991569 -2.583743 -2.273777

C -2.747206 -2.797899 -1.120162

C -2.107234 -2.908815 0.113631

C -0.724591 -2.781620 0.200794

H -0.019222 -2.290269 -3.093491

H -2.482532 -2.502817 -3.238333

H -3.826688 -2.888940 -1.185073

H -2.684718 -3.093879 1.014272

H -0.221756 -2.849849 1.161863

N 1.409651 -2.317205 -0.861629

H 1.834221 -2.718542 -0.029143

H 1.940293 -2.605102 -1.680368

**(11a)TS4B**

M062x/BSI SCF energy: -1512.062779 a.u.

M062x/BSII SCF energy in solution: -1512.368407 a.u.

M062x/BSII free energy in solution: -1511.942856 a.u.

S 1.680446 1.523185 1.048209

O -3.749974 -1.704141 -0.965212

O -3.972422 -1.546996 1.263559

C 0.146936 0.887029 0.624869

C -0.854856 0.929492 1.679518

C -2.100458 0.484344 1.451307

C -2.531365 0.017503 0.103195

C -1.383537 -0.286477 -0.803939

C -0.153717 0.325987 -0.617381

C 2.756392 1.004228 -0.328572

C 4.214393 1.109391 0.104655

C 5.142677 0.643258 -1.016636

C 6.608250 0.677875 -0.596527

C -3.497257 -1.164119 0.223329

H -0.570373 1.311091 2.655606

H -2.855026 0.510031 2.231971

H -1.634520 -0.683541 -1.783370

H 0.591502 0.264052 -1.399402

H 2.506104 -0.036155 -0.558568

H 2.550472 1.632634 -1.199484

H 4.456640 2.142667 0.380475

H 4.374379 0.488152 0.994940

H 4.990853 1.276680 -1.898789

H 4.862581 -0.376816 -1.309706

H 6.908544 1.689524 -0.305323

H 6.785597 0.017384 0.258576

C -4.645500 -2.830867 -0.958341

H -5.628325 -2.524480 -0.596645

H -4.704229 -3.164609 -1.992168

H -4.247959 -3.621936 -0.319541

H 7.262123 0.355113 -1.411202

C -1.746079 4.618374 -0.401125

N -1.094614 5.543630 -0.645798

C -2.548796 3.473281 -0.083413

H -3.125324 3.523827 0.836514

C -2.574101 2.402778 -0.883981

C -3.383827 1.184504 -0.573380

H -1.978685 2.395776 -1.793478

H -3.811950 0.773975 -1.491596

H -4.195748 1.420209 0.120641

C 0.465384 -2.501408 0.049087

C 1.067562 -2.142613 1.257858

C 2.455030 -2.170066 1.376958

C 3.249148 -2.538480 0.292283

C 2.644669 -2.871189 -0.921641

C 1.259719 -2.845346 -1.050628

H 0.445128 -1.851642 2.100513

H 2.914432 -1.901353 2.323414

H 4.329957 -2.564619 0.390028

H 3.254584 -3.154146 -1.773950

H 0.785117 -3.104608 -1.993392

N -0.932065 -2.406759 -0.089520

H -1.308243 -2.997918 -0.826998

H -1.422852 -2.584128 0.785062

**(11a)TS4C**

M062x/BSI SCF energy: -1512.06174 a.u.

M062x/BSII SCF energy in solution: -1512.367644 a.u.

M062x/BSII free energy in solution: -1511.944834 a.u.

S -2.588235 -0.176975 -1.026195

O 3.341029 0.376141 1.650573

O 3.879089 0.756207 -0.497806

C -0.936974 -0.072516 -0.525620

C -0.536171 -0.003173 0.865618

C 0.672306 0.480588 1.202598

C 1.597991 1.113011 0.214702

C 1.174701 0.911863 -1.202894

C -0.031180 0.430483 -1.540676

C -3.479749 -0.783072 0.446261

C -4.925076 -1.076619 0.053748

C -5.729821 -1.567758 1.257004

C -7.175016 -1.883685 0.885502

C 3.066140 0.695945 0.390250

H -1.203226 -0.383380 1.630810

H 0.966505 0.519186 2.247730

H 1.865714 1.253636 -1.968501

H -0.333037 0.364667 -2.582035

H -3.435679 -0.018245 1.226276

H -2.981211 -1.690982 0.798662

H -4.947068 -1.834968 -0.738075

H -5.390032 -0.169991 -0.352229

H -5.248632 -2.461805 1.672328

H -5.706788 -0.802990 2.042569

H -7.221079 -2.662336 0.117476

H -7.680861 -0.995928 0.492759

C 4.725683 0.121186 1.936119

H 5.111205 -0.657048 1.275805

H 4.756734 -0.203746 2.974383

H 5.305009 1.037309 1.803319

H -7.738725 -2.234287 1.754176

C -1.514278 4.592470 -0.488623

N -2.582455 5.038874 -0.474194

C -0.191496 4.038238 -0.517542

H 0.411319 4.267113 -1.392173

C 0.261981 3.283840 0.488728

C 1.628014 2.676539 0.496733

H -0.382475 3.082813 1.341485

H 2.267460 3.134912 -0.262970

H 2.090751 2.819022 1.479025

C 1.071992 -2.371008 -0.618451

C 1.890486 -2.260727 -1.753496

C 3.268362 -2.365024 -1.622101

C 3.844909 -2.559553 -0.366094

C 3.033046 -2.643337 0.763330

C 1.649635 -2.543085 0.646994

H 1.433051 -2.118207 -2.728724

H 3.896229 -2.295022 -2.504443

H 4.922496 -2.647141 -0.270970

H 3.476897 -2.789298 1.743558

H 1.009776 -2.625701 1.521089

N -0.310920 -2.222010 -0.742809

H -0.854119 -2.677845 -0.012389

H -0.678673 -2.442592 -1.665483

**(11a)TS4a**

M062x/BSI SCF energy: -1512.053677 a.u.

M062x/BSII SCF energy in solution: -1512.359518 a.u.

M062x/BSII free energy in solution: -1511.933788 a.u.

S 2.418933 -0.652743 1.163393

O -2.384590 -1.968080 1.469885

O -3.702844 -0.279817 2.143505

C 0.768152 -0.301219 0.821201

C 0.087438 0.517274 1.719079

C -1.182272 0.984416 1.384513

C -2.042485 0.182008 0.439691

C -1.231971 -0.642333 -0.507484

C 0.084703 -0.842589 -0.344818

C 3.040446 -1.535977 -0.308110

C 4.549552 -1.709957 -0.161360

C 5.137941 -2.423957 -1.378109

C 6.649967 -2.590489 -1.267198

C -2.817206 -0.719615 1.453277

H 0.606639 0.936287 2.574355

H -1.731057 1.585815 2.104157

H -1.757474 -1.101604 -1.341026

H 0.633538 -1.442606 -1.059737

H 2.542407 -2.506180 -0.385468

H 2.808861 -0.931768 -1.190121

H 5.023411 -0.727233 -0.047480

H 4.774234 -2.284459 0.745436

H 4.891626 -1.854163 -2.282415

H 4.662712 -3.406394 -1.485873

H 7.146515 -1.618290 -1.184389

H 6.915618 -3.177430 -0.382222

C -3.051951 -2.836327 2.405971

H -4.115380 -2.892672 2.167319

H -2.579273 -3.809077 2.288932

H -2.919242 -2.460846 3.421853

H 7.057183 -3.102019 -2.143526

C -5.534627 -0.722366 -2.681328

N -6.410589 -1.353786 -3.098696

C -4.443698 0.064383 -2.182967

H -3.819001 0.547933 -2.929260

C -4.224948 0.196486 -0.870401

C -3.106748 1.025344 -0.315004

H -4.885085 -0.301837 -0.163123

H -3.515899 1.757740 0.389903

H -2.617662 1.555431 -1.135185

C 0.697194 2.737530 -0.073824

C 1.090441 2.172999 -1.290587

C 2.443026 2.023853 -1.578195

C 3.407584 2.416525 -0.650326

C 3.010437 2.975583 0.564200

C 1.660363 3.132440 0.857743

H 0.333768 1.848793 -2.000377

H 2.744358 1.598596 -2.530687

H 4.462224 2.296067 -0.876245

H 3.754850 3.290233 1.288712

H 1.344251 3.564198 1.803879

N -0.669670 2.785116 0.266605

H -0.876966 3.484632 0.976709

H -1.274815 2.934526 -0.536666

**(11a)TS4b**

M062x/BSI SCF energy: -1512.056595 a.u.

M062x/BSII SCF energy in solution: -1512.363085 a.u.

M062x/BSII free energy in solution: -1511.93801 a.u.

S -2.140124 -1.621275 -1.274500

O 3.197181 -0.916045 1.662724

O 3.045231 -2.220520 -0.162484

C -0.645811 -0.942216 -0.789930

C 0.352880 -0.823949 -1.840000

C 1.588708 -0.383649 -1.558037

C 1.998600 -0.002573 -0.172181

C 0.829404 0.150241 0.753421

C -0.366463 -0.496811 0.505621

C -3.221192 -1.361759 0.169903

C -4.676707 -1.539560 -0.247259

C -5.605162 -1.313618 0.945670

C -7.075225 -1.421442 0.555670

C 2.797033 -1.204363 0.428852

H 0.082163 -1.108508 -2.851922

H 2.343907 -0.339673 -2.338553

H 1.056994 0.472090 1.764587

H -1.111017 -0.549032 1.288593

H -2.940371 -2.070496 0.954069

H -3.052536 -0.339155 0.522046

H -4.917182 -0.821040 -1.041007

H -4.837227 -2.543952 -0.657416

H -5.404793 -0.321642 1.370953

H -5.371336 -2.044202 1.729410

H -7.331830 -0.679421 -0.207600

H -7.301299 -2.411997 0.148255

C 4.062125 -1.891047 2.273031

H 4.974415 -1.998669 1.682278

H 4.290138 -1.500694 3.262805

H 3.551040 -2.852243 2.346242

H -7.727270 -1.255578 1.417424

C 6.700049 0.417207 -0.378390

N 7.744693 0.122923 -0.781118

C 5.411141 0.781243 0.135807

H 5.359219 1.001839 1.198850

C 4.338754 0.846269 -0.660122

C 2.977127 1.211655 -0.149100

H 4.441096 0.610959 -1.717758

H 2.564431 2.000940 -0.782508

H 3.044123 1.582697 0.876792

C -1.247334 2.325033 0.185601

C -2.067049 2.486053 1.308563

C -3.449558 2.407450 1.175093

C -4.027910 2.149051 -0.069252

C -3.207675 1.957896 -1.179916

C -1.822891 2.036573 -1.055819

H -1.614703 2.691279 2.275394

H -4.078585 2.552400 2.048166

H -5.107363 2.092739 -0.169611

H -3.645174 1.746403 -2.151046

H -1.179826 1.886128 -1.920078

N 0.151154 2.353831 0.313125

H 0.611610 2.690714 -0.527132

H 0.476086 2.877651 1.121417

**(11a)TS4c**

M062x/BSI SCF energy: -1512.055799 a.u.

M062x/BSII SCF energy in solution: -1512.362063 a.u.

M062x/BSII free energy in solution: -1511.935737 a.u.

S 1.900968 -1.008357 1.424387

O -4.155308 -1.812087 -0.885524

O -4.135882 -1.513707 1.340937

C 0.415350 -0.592125 0.676358

C -0.562699 -0.006182 1.592902

C -1.863944 0.029425 1.282715

C -2.406446 -0.479110 -0.016182

C -1.395889 -1.255443 -0.806417

C -0.092005 -1.292340 -0.500401

C 2.927605 -1.667176 0.063526

C 4.392583 -1.393703 0.386636

C 5.293377 -1.790924 -0.781227

C 6.756319 -1.463662 -0.502552

C -3.654060 -1.334586 0.250842

H -0.203621 0.395927 2.535822

H -2.577071 0.446043 1.987487

H -1.763665 -1.787526 -1.679584

H 0.597496 -1.857583 -1.117038

H 2.726703 -2.735405 -0.052000

H 2.641375 -1.142185 -0.851396

H 4.520086 -0.324383 0.595896

H 4.687539 -1.941584 1.289852

H 4.960487 -1.257133 -1.680927

H 5.178023 -2.861819 -0.987453

H 6.883715 -0.391229 -0.318344

H 7.116622 -1.999003 0.381676

C -5.356893 -2.590308 -0.753165

H -6.143773 -1.989736 -0.293247

H -5.634285 -2.876056 -1.765881

H -5.164973 -3.474760 -0.143181

H 7.394362 -1.739243 -1.346541

C -6.343381 2.105945 -0.146100

N -7.222916 2.673107 0.348698

C -5.261846 1.399078 -0.769345

H -5.512511 0.803971 -1.643598

C -4.014017 1.479760 -0.295034

C -2.875372 0.730160 -0.914139

H -3.808973 2.086058 0.584975

H -2.010956 1.388740 -1.049702

H -3.170563 0.331053 -1.887744

C 2.306281 1.652130 -0.336390

C 3.156847 1.408478 -1.421678

C 4.501912 1.746039 -1.332185

C 5.010418 2.318968 -0.164168

C 4.164028 2.547631 0.918311

C 2.815332 2.209727 0.840713

H 2.753474 0.973371 -2.332837

H 5.156017 1.566710 -2.180015

H 6.060233 2.586463 -0.101956

H 4.551825 2.989942 1.830176

H 2.148491 2.391111 1.679555

N 0.952533 1.265675 -0.390945

H 0.330899 1.899639 0.108070

H 0.608747 1.116505 -1.337972

**(11a)IM6**

M062x/BSI SCF energy: -1512.076072 a.u.

M062x/BSII SCF energy in solution: -1512.3818 a.u.

M062x/BSII free energy in solution: -1511.954178 a.u.

S -2.559164 -0.097252 -0.960234

O 3.272244 0.280865 1.688483

O 3.631900 0.175157 -0.527240

C -0.812160 -0.417856 -0.529954

C -0.482205 -0.141218 0.902215

C 0.589427 0.558608 1.264905

C 1.566665 1.158420 0.290079

C 1.101296 1.039425 -1.134660

C 0.030030 0.347029 -1.507627

C -3.492146 -0.836250 0.429789

C -4.972112 -0.870945 0.054745

C -5.816648 -1.454541 1.187115

C -7.301051 -1.482496 0.837905

C 2.931683 0.462065 0.412032

H -1.142482 -0.563504 1.654283

H 0.794264 0.722319 2.319686

H 1.689529 1.565928 -1.881806

H -0.264868 0.295949 -2.553485

H -3.343922 -0.228107 1.325460

H -3.130807 -1.851871 0.626723

H -5.110506 -1.468119 -0.854424

H -5.317520 0.145370 -0.172173

H -5.468055 -2.470573 1.409940

H -5.660154 -0.862459 2.096894

H -7.481339 -2.080395 -0.061299

H -7.677654 -0.472323 0.648222

C 4.588461 -0.248514 1.906727

H 4.711055 -1.193513 1.375226

H 4.672954 -0.398346 2.981635

H 5.339760 0.464666 1.560849

H -7.891561 -1.913378 1.651019

C -0.655861 5.311918 -0.533522

N -1.617595 5.942687 -0.668290

C 0.542879 4.541263 -0.375910

H 1.406141 4.865389 -0.950615

C 0.586013 3.483769 0.441272

C 1.817624 2.658397 0.638246

H -0.308149 3.194139 0.991808

H 2.631700 3.038631 0.012779

H 2.136018 2.713674 1.685973

C 0.850194 -2.376777 -0.648963

C 1.642630 -2.427045 -1.789014

C 2.972144 -2.814561 -1.660054

C 3.488775 -3.133685 -0.406470

C 2.676759 -3.076248 0.724871

C 1.342018 -2.696237 0.611020

H 1.225365 -2.171126 -2.758840

H 3.603095 -2.860323 -2.540853

H 4.527831 -3.432252 -0.311116

H 3.076645 -3.333169 1.700366

H 0.696838 -2.661088 1.483259

N -0.545734 -1.942940 -0.785813

H -1.139542 -2.482542 -0.142383

H -0.884339 -2.160483 -1.733079

**(11a)IM7**

M062x/BSI SCF energy: -1878.380736 a.u.

M062x/BSII SCF energy in solution: -1878.781938 a.u.

M062x/BSII free energy in solution: -1878.161746 a.u.

S 1.189712 1.945495 -0.013534

O -4.689554 -0.236357 -1.408380

O -3.875061 -1.506227 0.253049

C 0.238000 0.389641 -0.364098

C -0.586593 0.180241 0.873071

C -1.912391 0.276665 0.903648

C -2.756605 0.581219 -0.302742

C -1.940396 0.627454 -1.570469

C -0.611781 0.563684 -1.592440

C 2.387690 2.011122 -1.394070

C 3.035521 3.393542 -1.438039

C 4.082011 3.489002 -2.548156

C 4.737438 4.865213 -2.604220

C -3.819482 -0.515299 -0.434551

H -0.029468 -0.056168 1.777204

H -2.440982 0.111278 1.838508

H -2.496907 0.756906 -2.495405

H -0.082513 0.629011 -2.540967

H 3.163176 1.248940 -1.251406

H 1.867500 1.810357 -2.336083

H 2.261647 4.154053 -1.593517

H 3.508494 3.612010 -0.471926

H 3.606605 3.265654 -3.511080

H 4.848732 2.720213 -2.390874

H 3.993692 5.646556 -2.790082

H 5.238680 5.099864 -1.659510

C -5.741704 -1.195034 -1.585833

H -6.324808 -1.289059 -0.667159

H -6.361251 -0.809081 -2.393316

H -5.325653 -2.167955 -1.854949

H 5.484235 4.916782 -3.401282

C -6.661570 1.826470 1.962091

N -7.393807 1.803294 2.858908

C -5.768516 1.851967 0.840414

H -6.224724 1.781081 -0.143570

C -4.444438 1.958903 1.005246

C -3.486126 1.955111 -0.145348

H -4.034876 2.022473 2.011373

H -2.712865 2.715507 0.001681

H -4.016344 2.170145 -1.076880

C 0.682964 -1.968291 -0.980202

C 1.049848 -2.454728 -2.237618

C 0.595619 -3.695682 -2.675323

C -0.250908 -4.453651 -1.870510

C -0.626721 -3.965666 -0.620611

C -0.156585 -2.735627 -0.166214

H 1.694562 -1.851911 -2.873161

H 0.897345 -4.061397 -3.651823

H -0.614294 -5.416772 -2.213430

H -1.284356 -4.549701 0.015619

H -0.442767 -2.378670 0.816755

N 1.223756 -0.714050 -0.529634

H 2.601267 -0.835417 0.876594

H 1.886312 -0.415809 -1.246174

C 2.488451 -1.375155 2.862894

C 2.383612 0.018783 3.459118

C 4.302078 0.740959 2.387447

C 4.522363 -0.602095 1.712540

C 3.401043 -2.715670 1.007107

C 4.116072 -2.724269 -0.330679

N 3.223521 -1.330174 1.558747

O 3.666748 0.583445 3.639428

H 3.056687 -2.034160 3.524733

H 1.503167 -1.807386 2.669337

H 1.906525 -0.047996 4.438699

H 1.778619 0.670342 2.811143

H 5.271234 1.211969 2.562056

H 3.701329 1.395191 1.737401

H 5.167438 -1.243371 2.319920

H 4.963154 -0.467809 0.724224

H 3.949288 -3.283101 1.763406

H 2.393924 -3.128766 0.916881

H 3.673967 -2.007307 -1.029618

H 5.181712 -2.508671 -0.230473

H 4.012421 -3.718978 -0.769929

**
(11a)TS5**

M062x/BSI SCF energy: -1878.364139 a.u.

M062x/BSII SCF energy in solution: -1878.764539 a.u.

M062x/BSII free energy in solution: -1878.148375 a.u.

S 0.776247 -0.927477 0.373840

O -5.307277 0.061768 1.453203

O -4.929606 1.734629 0.003725

C -0.482189 1.337588 1.325534

C -1.042647 1.546856 -0.003850

C -2.266827 1.094927 -0.299205

C -3.095328 0.277339 0.642926

C -2.530271 0.229773 2.031560

C -1.303503 0.668510 2.327871

C 1.974152 -1.077185 1.746625

C 2.073657 -2.486274 2.325544

C 3.095403 -2.589071 3.457559

C 3.193728 -3.999672 4.031401

C -4.536179 0.799080 0.655383

H -0.453311 2.069679 -0.745789

H -2.681378 1.275087 -1.286741

H -3.141018 -0.246157 2.793358

H -0.900166 0.571592 3.331801

H 2.968153 -0.749099 1.415228

H 1.674643 -0.381312 2.545710

H 1.084496 -2.796728 2.685718

H 2.343286 -3.187081 1.523721

H 2.826601 -1.884208 4.255247

H 4.078849 -2.273714 3.084622

H 2.229153 -4.327793 4.432537

H 3.491611 -4.716223 3.258672

C -6.691813 0.440429 1.498843

H -7.133252 0.373001 0.502575

H -7.167155 -0.265568 2.177093

H -6.791827 1.459528 1.877055

H 3.928909 -4.055244 4.839397

C -5.700442 -1.959859 -2.588839

N -6.225302 -2.047624 -3.617209

C -5.059922 -1.858933 -1.309711

H -5.620650 -2.230600 -0.455800

C -3.831595 -1.342959 -1.185148

C -3.144992 -1.217739 0.137979

H -3.309044 -0.981390 -2.068701

H -2.107250 -1.562920 0.072828

H -3.669566 -1.807885 0.893543

C 1.546925 2.707102 1.010784

C 2.890886 2.370682 1.179956

C 3.864516 3.058157 0.460143

C 3.497472 4.069172 -0.426404

C 2.154455 4.412118 -0.572374

C 1.173031 3.744048 0.155506

H 3.162033 1.576251 1.869774

H 4.910490 2.799989 0.592492

H 4.258282 4.600511 -0.988723

H 1.866091 5.218934 -1.238053

H 0.131995 4.039982 0.080721

N 0.583322 1.989818 1.773391

H 2.023776 -0.644791 -1.205001

H 0.863536 1.780722 2.729082

C 2.645495 -1.367349 -3.085094

C 3.142757 -2.710145 -2.574202

C 4.487138 -1.728415 -0.963409

C 4.070587 -0.326888 -1.380194

C 2.280503 1.013450 -2.405831

C 0.904744 1.025980 -3.047427

N 2.707829 -0.357037 -1.989410

O 4.458081 -2.609882 -2.067295

H 3.270369 -1.001321 -3.905797

H 1.609758 -1.451236 -3.417436

H 3.159957 -3.424435 -3.399791

H 2.461844 -3.086707 -1.795330

H 5.511337 -1.702282 -0.585977

H 3.825341 -2.100148 -0.165196

H 4.757661 0.071282 -2.132869

H 4.030356 0.352202 -0.523695

H 2.287854 1.608071 -1.487525

H 3.046400 1.403191 -3.082663

H 0.914046 0.599699 -4.052678

H 0.178529 0.480492 -2.435913

H 0.570348 2.063713 -3.128968

**(11a)IM8**

M062x/BSI SCF energy: -955.541916 a.u.

M062x/BSII SCF energy in solution: -955.778552 a.u.

M062x/BSII free energy in solution: -955.485254 a.u.

O -3.229972 -1.739009 0.263394

O -2.295981 -0.528589 1.911021

C 1.555165 -0.838143 -0.513786

C 1.013609 0.318620 0.168658

C -0.315328 0.456077 0.290838

C -1.303629 -0.491369 -0.303399

C -0.671705 -1.689731 -0.935660

C 0.654564 -1.832944 -1.062467

C -2.316601 -0.918378 0.771421

H 1.687723 1.065657 0.567304

H -0.714284 1.319563 0.815910

H -1.343912 -2.442782 -1.337045

H 1.086386 -2.697425 -1.557409

C -4.262415 -2.156615 1.174737

H -4.805497 -1.286433 1.548175

H -4.920328 -2.801531 0.595990

H -3.823975 -2.704110 2.010637

C -5.074826 2.316573 -0.208305

N -5.718718 3.171534 0.232604

C -4.288290 1.247777 -0.752967

H -4.828417 0.351939 -1.049032

C -2.962533 1.360806 -0.889342

C -2.120138 0.250029 -1.436703

H -2.464116 2.276595 -0.578712

H -1.395433 0.635546 -2.159512

H -2.749235 -0.495386 -1.929248

C 3.917693 -0.211850 -0.175599

C 4.890752 0.205909 -1.080859

C 5.953812 0.974792 -0.619718

C 6.040552 1.313850 0.729636

C 5.068105 0.874981 1.625661

C 4.001713 0.100144 1.179877

H 4.804972 -0.060190 -2.129874

H 6.712547 1.312455 -1.317652

H 6.871379 1.915224 1.083755

H 5.142155 1.124626 2.678748

H 3.255063 -0.272062 1.874363

N 2.844845 -1.022218 -0.669488

H 3.126719 -1.829282 -1.225689

**16a**

M062x/BSI SCF energy: -955.088821 a.u.

M062x/BSII SCF energy in solution: -955.332912 a.u.

M062x/BSII free energy in solution: -955.05385 a.u.

O -3.205190 -1.738340 0.212444

O -2.199553 -0.642197 1.893028

C 1.633536 -0.841446 -0.593483

C 1.052025 0.301489 0.132557

C -0.270110 0.447956 0.261420

C -1.278556 -0.480993 -0.352825

C -0.642404 -1.670698 -1.022492

C 0.678850 -1.809441 -1.154160

C -2.256765 -0.952929 0.728604

H 1.724501 1.037443 0.559156

H -0.666376 1.296787 0.812911

H -1.322737 -2.409672 -1.437335

H 1.109415 -2.661262 -1.672013

C -4.194532 -2.202334 1.142221

H -4.721412 -1.355704 1.587582

H -4.880223 -2.817302 0.562198

H -3.724103 -2.793680 1.930119

C -5.028722 2.340290 -0.090155

N -5.655809 3.190050 0.384833

C -4.265641 1.278740 -0.679444

H -4.819562 0.391570 -0.975422

C -2.942923 1.384721 -0.853951

C -2.119011 0.280363 -1.439665

H -2.431870 2.292325 -0.539107

H -1.411115 0.680720 -2.171558

H -2.766310 -0.445896 -1.938302

C 3.881476 -0.241317 -0.234322

C 4.818134 0.354358 -1.088103

C 5.851732 1.123674 -0.565142

C 5.984521 1.284480 0.813783

C 5.071289 0.668595 1.667097

C 4.023768 -0.089547 1.151465

H 4.715782 0.210664 -2.159517

H 6.563108 1.591767 -1.238929

H 6.798249 1.876973 1.219529

H 5.172569 0.776864 2.742883

H 3.316337 -0.579901 1.814477

N 2.884620 -1.061930 -0.796390

**TS5**

M062x/BSI SCF energy: -2186.335281 a.u.

M062x/BSII SCF energy in solution: -2186.781294 a.u.

M062x/BSII free energy in solution: -2186.435165 a.u.

S -1.694040 -1.020255 -0.989187

O 4.417855 -2.397620 1.348906

O 4.850884 -2.590971 -0.845897

C 0.008976 -1.409066 -0.616032

C 0.839744 -1.651929 -1.705893

C 2.167848 -1.977537 -1.455507

C 2.636703 -2.031087 -0.144376

C 1.786984 -1.772621 0.934579

C 0.451239 -1.468689 0.705366

C -2.594573 -2.415380 -0.244075

C -4.098587 -2.214618 -0.366266

C -4.836488 -3.393446 0.273926

C -6.349167 -3.222542 0.183938

C 4.082437 -2.368623 0.060772

H 0.468760 -1.583477 -2.723652

H 2.850544 -2.177821 -2.273950

H 2.166110 -1.813425 1.949008

H -0.219117 -1.269841 1.537181

H -2.240283 -3.289514 -0.800063

H -2.256592 -2.477915 0.794905

H -4.386100 -1.284501 0.135921

H -4.379903 -2.130568 -1.422456

H -4.533155 -3.480143 1.323849

H -4.535331 -4.322850 -0.222958

H -6.671372 -2.309064 0.693617

H -6.675879 -3.159550 -0.858721

C 5.787724 -2.724295 1.620914

H 6.449665 -1.983464 1.168307

H 5.885353 -2.710821 2.704648

H 6.023461 -3.714634 1.226570

H -6.866711 -4.066185 0.648024

C -1.572395 1.261141 0.233149

N -2.195528 0.162296 0.115497

C -1.997588 2.330183 1.189587

H -1.638557 3.290490 0.806233

C -3.476684 2.419352 1.480590

C -4.475934 2.020062 0.702015

H -3.696939 2.918681 2.420633

H -5.503800 2.187753 1.007622

H -4.320344 1.524674 -0.251796

C 1.117005 3.653316 -0.445952

S 1.017171 1.863591 0.035747

O 1.962007 1.158488 -0.782557

F 0.687714 3.785636 -1.682582

F 2.383305 4.001030 -0.352337

F 0.382037 4.370271 0.381315

O 0.961358 1.757404 1.471038

O -0.468888 1.504702 -0.578509

H -1.447913 2.148958 2.121805

**TS6**

M062x/BSI SCF energy: -2185.877208 a.u.

M062x/BSII SCF energy in solution: -2186.331783 a.u.

M062x/BSII free energy in solution: -2186.001074 a.u.

S -1.186607 -1.282271 -0.840150

O 5.153737 -1.158213 1.357421

O 5.559053 -1.864899 -0.733300

C 0.588459 -1.353173 -0.521706

C 1.433419 -1.702488 -1.571529

C 2.801584 -1.751993 -1.335095

C 3.301771 -1.443558 -0.068647

C 2.439910 -1.083091 0.969559

C 1.068283 -1.038940 0.745068

C -1.639301 -2.950662 -0.283641

C -3.132489 -3.187144 -0.454373

C -3.527555 -4.573632 0.055160

C -5.019729 -4.837645 -0.118656

C 4.781734 -1.515628 0.126578

H 1.035485 -1.923715 -2.558100

H 3.492857 -2.019038 -2.127316

H 2.839927 -0.838461 1.947130

H 0.375493 -0.741045 1.526555

H -1.035089 -3.639837 -0.881878

H -1.327729 -3.003953 0.764720

H -3.681596 -2.417570 0.099764

H -3.403721 -3.090858 -1.513022

H -3.255034 -4.657959 1.113871

H -2.950377 -5.336489 -0.481062

H -5.613832 -4.092844 0.420511

H -5.304956 -4.792358 -1.174701

C 6.560922 -1.218636 1.616023

H 7.102790 -0.570818 0.924069

H 6.688970 -0.872375 2.640079

H 6.922278 -2.244161 1.512571

H -5.294568 -5.825683 0.260687

C -2.150959 0.883799 0.046893

N -1.833734 -0.398839 0.376735

C -3.218898 1.598962 0.413548

C -4.363086 0.973690 1.120305

C -4.495990 0.950547 2.444102

H -5.137643 0.520010 0.499236

H -5.359014 0.489667 2.916300

H -3.745458 1.389263 3.096810

C -0.218650 3.982690 -0.459048

S 0.128429 2.207724 -0.123110

O 1.267743 1.853361 -0.935309

F -0.326468 4.175901 -1.762217

F 0.785545 4.699494 0.019971

F -1.344419 4.337273 0.140963

O 0.124882 2.041341 1.311807

O -1.183472 1.588700 -0.791236

H -3.245808 2.657937 0.178016

**TS7**

M062x/BSI SCF energy: -1224.505422 a.u.

M062x/BSII SCF energy in solution: -1224.741608 a.u.

M062x/BSII free energy in solution: -1224.436066 a.u.

S 1.802680 -0.332309 1.082306

O -4.475674 -0.566519 -1.200046

O -4.793114 -1.607561 0.764311

C 0.082425 -0.535442 0.687219

C -0.711320 -1.136622 1.662529

C -2.065605 -1.305928 1.396741

C -2.598341 -0.861616 0.188199

C -1.788874 -0.248785 -0.773381

C -0.432562 -0.082933 -0.529572

C 2.685459 -1.046808 -0.337364

C 4.191246 -0.968191 -0.119014

C 4.922253 -1.579387 -1.316916

C 6.435084 -1.547335 -1.128165

C -4.067879 -1.060897 -0.033633

H -0.286973 -1.468747 2.604836

H -2.717351 -1.774052 2.126133

H -2.217046 0.095485 -1.707769

H 0.201765 0.391464 -1.272814

H 2.326460 -2.080118 -0.383877

H 2.357294 -0.500300 -1.226437

H 4.495851 0.076369 0.007284

H 4.461311 -1.510265 0.794282

H 4.648127 -1.030183 -2.225024

H 4.585034 -2.613196 -1.455973

H 6.793834 -0.520453 -1.007310

H 6.732008 -2.113845 -0.240031

C -5.876214 -0.714086 -1.475645

H -6.465096 -0.191336 -0.719493

H -6.032443 -0.268943 -2.456250

H -6.149479 -1.770710 -1.486030

H 6.946184 -1.982843 -1.990790

C 1.287216 2.172597 0.870312

N 2.193229 1.334152 0.781874

C 0.386112 3.115822 0.919435

H 0.228794 3.601202 1.881921

C -0.410106 3.517202 -0.277663

C -0.144967 4.615901 -0.976130

H -1.244492 2.869761 -0.539872

H -0.757975 4.892471 -1.828407

H 0.685451 5.267810 -0.720213

**TS8**

M062x/BSI SCF energy: -1224.523731 a.u.

M062x/BSII SCF energy in solution: -1224.759378 a.u.

M062x/BSII free energy in solution: -1224.453275 a.u.

S -2.026601 0.098386 -1.285125

O 4.185186 -1.602705 0.441114

O 4.676449 0.213381 -0.788411

C -0.380433 -0.093312 -0.833237

C 0.547532 0.847953 -1.392423

C 1.927943 0.614015 -1.291083

C 2.389960 -0.377732 -0.453479

C 1.465696 -1.218514 0.224280

C 0.107199 -1.095965 0.027676

C -2.945188 -0.909748 -0.074008

C -4.431252 -0.589790 -0.206088

C -5.257638 -1.425820 0.771282

C -6.748084 -1.122051 0.660830

C 3.869669 -0.543009 -0.298434

H 0.180600 1.601158 -2.085614

H 2.630659 1.263908 -1.803044

H 1.843509 -1.995056 0.881256

H -0.568963 -1.792899 0.508055

H -2.750699 -1.967364 -0.271816

H -2.575845 -0.650433 0.922869

H -4.593701 0.477196 -0.012137

H -4.765298 -0.789985 -1.231457

H -4.912950 -1.230783 1.794008

H -5.079054 -2.489810 0.575029

H -6.951459 -0.069849 0.883757

H -7.115180 -1.326634 -0.349944

C 5.590862 -1.827149 0.629383

H 6.041172 -0.980573 1.151225

H 5.666242 -2.731144 1.230319

H 6.081272 -1.966867 -0.335635

H -7.329435 -1.731495 1.357939

C -0.923173 2.589506 0.394907

N -2.064937 2.773188 0.310255

C 0.462922 2.273623 0.488545

H 1.141018 2.921563 -0.060882

C 0.927393 1.519524 1.590076

C 2.264298 1.351378 1.771812

H 0.205159 1.023089 2.230257

H 2.648293 0.750044 2.590932

H 2.985516 1.856572 1.133239

**TS9**

M062x/BSI SCF energy: -1224.521986 a.u.

M062x/BSII SCF energy in solution: -1224.757055 a.u.

M062x/BSII free energy in solution: -1224.450609 a.u.

S -2.454230 -0.924804 1.132664

O 1.804901 2.871875 -0.569420

O 0.374930 2.353984 1.123351

C -0.868693 -0.433498 0.589217

C 0.210937 -0.706696 1.487214

C 1.509085 -0.404264 1.173772

C 1.814397 0.249848 -0.045083

C 0.727291 0.559891 -0.922236

C -0.597989 0.173043 -0.625031

C -3.533178 -0.460944 -0.260007

C -4.972422 -0.837724 0.080134

C -5.916669 -0.488603 -1.070204

C -7.364505 -0.844489 -0.748685

C 1.050038 2.149516 0.183427

H -0.007077 -1.191309 2.435361

H 2.314618 -0.656997 1.855950

H 0.954029 0.960343 -1.907215

H -1.373937 0.399445 -1.345433

H -3.447206 0.617075 -0.426743

H -3.195532 -0.992192 -1.155023

H -5.035927 -1.911859 0.292302

H -5.287408 -0.308759 0.987625

H -5.595638 -1.021041 -1.973693

H -5.837188 0.583037 -1.290302

H -7.468504 -1.915300 -0.546006

H -7.713770 -0.301837 0.135518

C 1.876534 4.289899 -0.199668

H 2.259544 4.369052 0.817474

H 2.565202 4.724762 -0.918519

H 0.880809 4.723655 -0.287456

H -8.028413 -0.594475 -1.580627

C 5.463257 -2.685395 -0.343232

N 5.922652 -3.726158 -0.557432

C 4.905054 -1.394785 -0.060604

H 5.384767 -0.823714 0.729291

C 3.855716 -0.927055 -0.743234

C 3.255578 0.424592 -0.474262

H 3.399937 -1.528335 -1.527151

H 3.294502 1.026036 -1.386873

H 3.821055 0.935495 0.310356

**IM5**

M062x/BSI SCF energy: -1224.534469 a.u.

M062x/BSII SCF energy in solution: -1224.769638 a.u.

M062x/BSII free energy in solution: -1224.465629 a.u.

S -2.550709 -1.795773 0.120720

O 1.740959 2.909813 -0.436551

O 0.005469 2.645734 0.967637

C -0.970027 -1.032040 -0.044803

C 0.165330 -1.864870 0.158583

C 1.481914 -1.417432 0.074948

C 1.762645 -0.095314 -0.204815

C 0.627173 0.816506 -0.484265

C -0.738610 0.285547 -0.317366

C -3.685737 -0.385453 -0.085271

C -5.120805 -0.884448 0.057878

C -6.123087 0.257870 -0.106324

C -7.563166 -0.225893 0.030265

C 0.747631 2.232309 0.117687

H 0.000826 -2.913028 0.403622

H 2.284805 -2.122215 0.259740

H 0.725362 0.971468 -1.586082

H -1.544996 0.998529 -0.452218

H -3.458434 0.362569 0.680810

H -3.528835 0.055545 -1.074703

H -5.319184 -1.657025 -0.694761

H -5.254397 -1.349119 1.042035

H -5.979220 0.726924 -1.087159

H -5.917510 1.029301 0.645649

H -7.795931 -0.979944 -0.728562

H -7.731717 -0.678026 1.012944

C 1.945771 4.241587 0.078161

H 2.147870 4.200809 1.149469

H 2.805547 4.631234 -0.462346

H 1.060726 4.850221 -0.112126

H -8.272340 0.597996 -0.086162

C 6.184030 -1.869610 0.205314

N 6.991116 -2.681583 0.035123

C 5.188289 -0.861276 0.428902

H 5.285744 -0.279116 1.341008

C 4.202840 -0.655230 -0.448567

C 3.170447 0.415724 -0.273320

H 4.141895 -1.261244 -1.350365

H 3.246127 1.136546 -1.095196

H 3.357956 0.989783 0.646290

**TS10**

M062x/BSI SCF energy: -1224.521277 a.u.

M062x/BSII SCF energy in solution: -1224.756486 a.u.

M062x/BSII free energy in solution: -1224.449564 a.u.

S 2.135196 0.216600 1.207544

O -4.098617 -1.476248 -0.666671

O -4.585695 -0.318502 1.201528

C 0.455146 -0.119318 0.848376

C -0.501425 0.619144 1.609640

C -1.857979 0.409528 1.469174

C -2.317194 -0.475618 0.490363

C -1.380636 -1.153637 -0.335637

C 0.006707 -1.023878 -0.098155

C 3.034861 -0.847352 0.035306

C 4.532981 -0.614901 0.210992

C 5.344269 -1.501115 -0.733731

C 6.844408 -1.276032 -0.574825

C -3.791761 -0.741537 0.397036

H -0.145463 1.329974 2.351613

H -2.571338 0.935772 2.093907

H -1.731545 -1.939350 -0.996009

H 0.688639 -1.620487 -0.691980

H 2.780253 -1.891630 0.240275

H 2.721956 -0.592764 -0.982249

H 4.768413 0.438772 0.019051

H 4.821223 -0.827613 1.247531

H 5.045139 -1.296048 -1.768947

H 5.102667 -2.552765 -0.537379

H 7.109256 -0.237165 -0.796652

H 7.165025 -1.491427 0.449580

C -5.493122 -1.800445 -0.814191

H -6.080687 -0.886034 -0.911806

H -5.560064 -2.398731 -1.720239

H -5.835244 -2.370469 0.051209

H 7.417728 -1.919552 -1.247638

C 0.205184 3.361041 -0.553161

N 1.106355 4.067324 -0.386734

C -0.910647 2.481768 -0.737983

H -1.835106 2.755162 -0.234445

C -0.820303 1.382310 -1.521469

C -1.941308 0.503493 -1.637849

H 0.116297 1.118772 -2.004192

H -1.951058 -0.246533 -2.423767

H -2.914308 0.892549 -1.353595
